# High yield derivation of enriched glutamatergic neurons from suspension-cultured mouse ESCs for neurotoxicology research

**DOI:** 10.1186/1471-2202-13-127

**Published:** 2012-10-24

**Authors:** Kyle S Hubbard, Ian M Gut, Megan E Lyman, Kaylie M Tuznik, Mariano T Mesngon, Patrick M McNutt

**Affiliations:** 1 United States Army Medical Research Institute of Chemical Defense, 3100 Ricketts Point Rd, Aberdeen Proving Ground, MD 21010, USA; 2 Department of Clinical Investigations, Madigan Army Medical Center, 9040 Jackson Avenue, Fort Lewis, WA 98431, USA

**Keywords:** Mouse embryonic stem cells, Neurotoxicity, *In vitro* modeling, BoNT, LTX, Glutamate

## Abstract

**Background:**

Recently, there has been a strong emphasis on identifying an *in vitro* model for neurotoxicity research that combines the biological relevance of primary neurons with the scalability, reproducibility and genetic tractability of continuous cell lines. Derived neurons should be homotypic, exhibit neuron-specific gene expression and morphology, form functioning synapses and consistently respond to neurotoxins in a fashion indistinguishable from primary neurons. However, efficient methods to produce neuronal populations that are suitable alternatives to primary neurons have not been available.

**Methods:**

With the objective of developing a more facile, robust and efficient method to generate enriched glutamatergic neuronal cultures, we evaluated the neurogenic capacity of three mouse embryonic stem cell (ESC) lines (R1, C57BL/6 and D3) adapted to feeder-independent suspension culture. Neurogenesis and neuronal maturation were characterized as a function of time in culture using immunological, genomic, morphological and functional metrics. The functional responses of ESNs to neurotropic toxins with distinctly different targets and mechanisms of toxicity, such as glutamate, α-latrotoxin (LTX), and botulinum neurotoxin (BoNT), were also evaluated.

**Results:**

Suspension-adapted ESCs expressed markers of pluripotency through at least 30 passages, and differentiation produced 97×10^6 ^neural progenitor cells (NPCs) per 10-cm dish. Greater than 99% of embryonic stem cell-derived neurons (ESNs) expressed neuron-specific markers by 96 h after plating and rapidly developed complex axodendritic arbors and appropriate compartmentalization of neurotypic proteins. Expression profiling demonstrated the presence of transcripts necessary for neuronal function and confirmed that ESN populations were predominantly glutamatergic. Furthermore, ESNs were functionally receptive to all toxins with sensitivities and responses consistent with primary neurons.

**Conclusions:**

These findings demonstrate a cost-effective, scalable and flexible method to produce a highly enriched glutamatergic neuron population. The functional characterization of pathophysiological responses to neurotropic toxins and the compatibility with multi-well plating formats were used to demonstrate the suitability of ESNs as a discovery platform for molecular mechanisms of action, moderate-throughput analytical approaches and diagnostic screening. Furthermore, for the first time we demonstrate a cell-based model that is sensitive to all seven BoNT serotypes with EC_50 _values comparable to those reported in primary neuron populations. These data providing compelling evidence that ESNs offer a neuromimetic platform suitable for the evaluation of molecular mechanisms of neurotoxicity.

## Background

The use of live animals or primary neurons for neurotoxicity research is complicated by technical, ethical and economic considerations. An alternative strategy is the use of a cultured cell line that can be induced to exhibit neuronal characteristics. To date, the most common of these “neurogenic” cell lines are human and mouse neuroblastomas, which often exhibit poor sensitivity to neurotoxins, are comprised of heterogeneous phenotypes and may not form functioning synapses, making it questionable how accurately they model neuronal mechanisms of pathogenesis [1-4]. An effective cell-based model system for neurotoxin research would combine the biological relevance of primary neurons with the flexibility of continuous cell lines. Such a model would exhibit normal neurogenic progression, faithfully recapitulate the full range of interactions between primary neurons and neurotoxins, be compatible with modern cell and molecular techniques and facilitate moderate-throughput screening applications [5].

Early efforts to differentiate ESCs into neurons produced cells with morphological and functional characteristics similar to primary neurons [6-10]. These cultures also contained a high percentage of glial cells and multiple neuron subtypes, which significantly detracted from their suitability as a basic research tool or therapeutic screening platform. Recently, a variation of the 4/4 method (so named because it involves withdrawal of leukocyte inhibitory factor (LIF) from ESC aggregates for 4 days followed by supplementation with retinoic acid (RA) for 4 d) was described with significantly improved neuronal yield and purity [11,12]. Widespread application of this protocol was limited by several factors: custom media formulations were required; multiple manipulations were needed to isolate ESCs from feeder cell populations; and neurons were not produced at yields amenable to moderate-throughput screening.

Here we demonstrate that the robust production of enriched glutamatergic neurons from suspension-cultured mouse ESCs using a variant of the 4/4 method offers a facile neuron model that exhibits high sensitivity to a variety of biological neurotoxins and replicates stages of neurogenesis observed in dissociated primary neuron cultures. We developed a facile, cost-effective procedure that enables the rapid production of large numbers of highly enriched glutamatergic ESNs from continuous suspension cultures of murine ESCs. Immunological, genomic, morphological and functional evaluations suggest that ESN maturation and behavior recapitulate those described in primary neurons. We further demonstrate that ESNs appropriately respond to different neurotropic toxins (glutamate, α-latrotoxin [LTX], and BoNT/A, /B, /C, /D, /F and /G) at doses similar to primary neurons.

These data provide compelling evidence that suspension culture-derived ESNs comprise a novel cell model for neurotoxicity research that combines the verisimilitude of primary neurons with the flexibility of continuous cell lines. The identification of a robust, scalable and sensitive neuron-based research platform amenable to genetic modification and compatible with moderate-throughput techniques should enable detailed biochemical and molecular approaches that are not feasible in neurogenic cells or primary neurons. We anticipate this platform to expedite the identification and validation of novel therapeutic approaches to a variety of neurotoxins as well as the elucidation of molecular aspects of pathogenesis following intoxication.

## Results

### Suspension-adapted ESCs remain pluripotent and mitotically active

**Figure 1:**
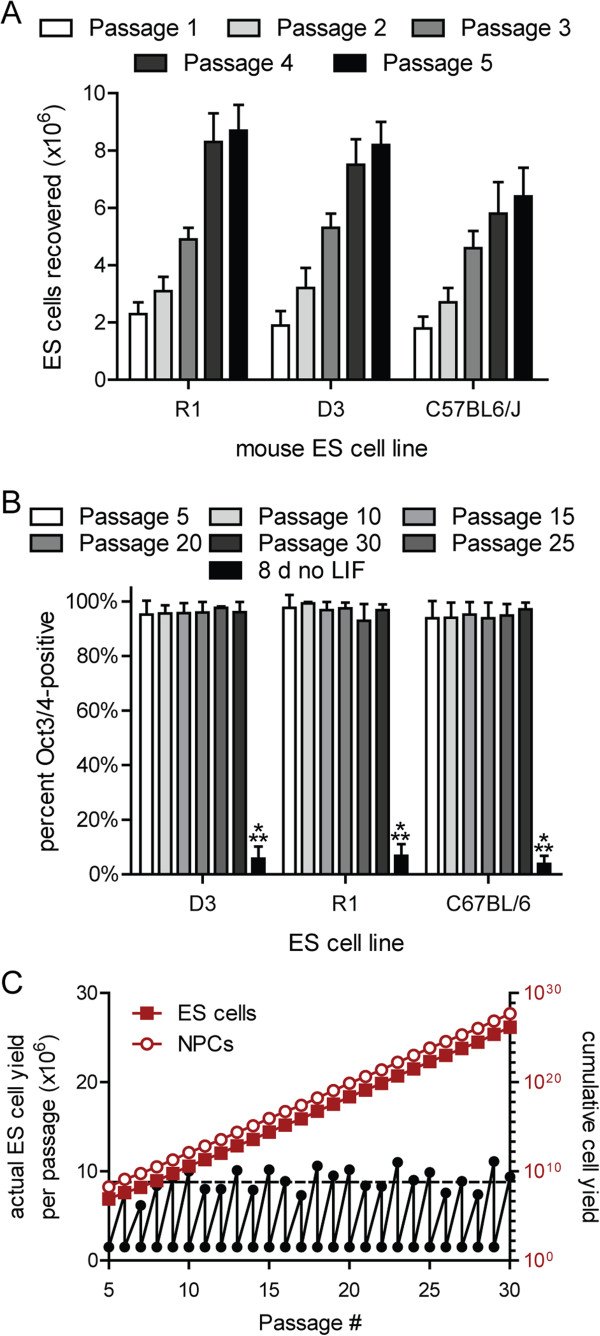
**Suspension-adapted ESNs remain mitotically stable and express markers of pluripotency. A**. ESC yields during the first five passages after transition to suspension culture. **B**. Summary of flow cytometry data demonstrating no substantive change in Oct3/4 expression in three different mESC lines across 25 passages in suspension culture (n=6 for each ESC line). LIF indicates leukocyte inhibitor factor. **A**, **B**. Error bars indicate standard deviation. **C**. Black, actual yields for a representative R1 ESC culture between 5–30 passages after suspension adaptation. Theoretical yields of ESCs (red, filled) and NPCs (red, not filled) over 25 passages are also presented.

ESCs are traditionally maintained on feeder cells in the presence of LIF and fetal calf serum to preserve germline competency [13]. To adapt ESC lines to feeder cell-free suspension culture, R1, D3 and C57BL/6 ESCs that had been co-cultured with mitotically inactivated mouse embryonic fibroblasts were dissociated and cultivated in bacterial dishes until suspended aggregates developed. Mitotic rates of the suspension-adapted ESCs stabilized by five passages (Figure 1A) and mean doubling times were 18.7 ± 2.4 h, 19.2 ± 2.1 h and 23.8 ± 1.9 h over 30 passages (R1, D3 and C57BL/6, respectively). Expression of the pluripotency marker Oct3/4 did not vary through 30 passages, whereas withdrawal of LIF resulted in a loss of Oct3/4 immunoreactivity within 8 d (Figure 1B). Theoretical estimates of cumulative yield over 25 passages from a single dish were 10^26^ ESCs (Figure 1C). 

**Figure 2:**
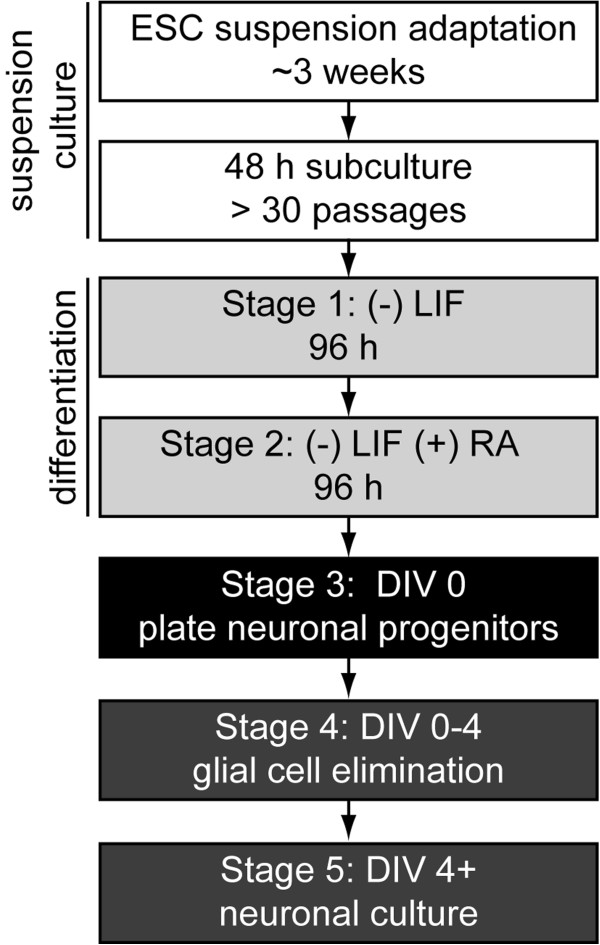
**Timeline from production of suspension-adapted ESCs to neuronal maturation.** LIF: leukocyte inhibitory factor. RA: retinoic acid. The presence or absence of RA or LIF is marked by a + or –.

**Figure 3:**
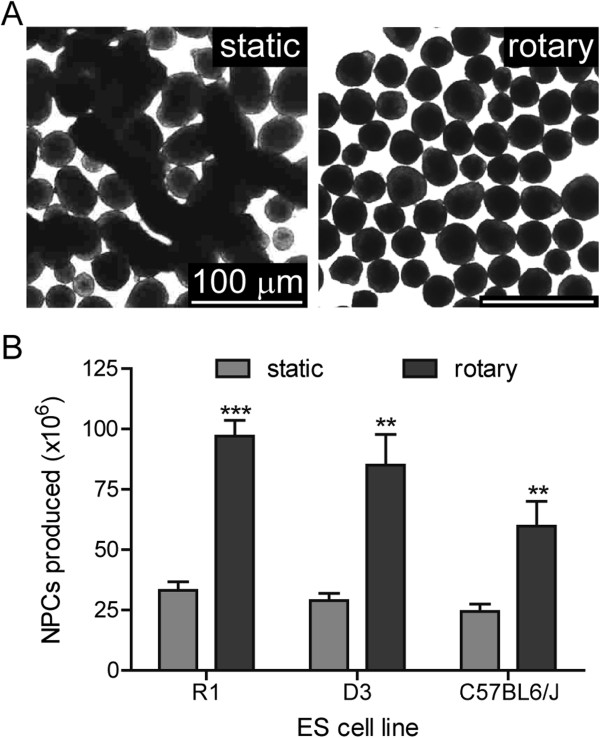
**Rotary conditions increase NPC yield during differentiation. A**. Bright-field images of ESCs differentiated under static (left) or rotary conditions (right; 45 rpm). Rotary conditions produced spherical aggregates without agglomeration. **B**. Average NPC yields for each cell line at DIV 0. Rotary culture of cell aggregates improves neural progenitor cell recovery approximately three-fold (P < .001, n=5).

Suspension-adapted ESCs were differentiated into neurons using a modified 4/4 protocol, with increased RA concentrations and incubation under rotary conditions in low-attachment dishes (Figure 2) [6,11,14]. We initially observed that differentiation under static conditions resulted in large, agglomerated complexes by DIV 0 (Figure 3A). Hypothesizing that agglomeration might limit recovery of NPCs at DIV 0, NPC yields were compared between static differentiation conditions versus differentiation on a rotary shaker at 45 RPM. Rotary conditions eliminated agglomeration and increased average yield by 290% to 97 × 10^6^ NPCs per 10-cm dish (Figure 3A-B). 

No differences were observed in NPC yield between 5–30 passages in any ESC line. In one instance, an R1 culture maintained for 65 passages produced 87 × 10^6^ NPCs, which underwent normal neuronal development, indicating that extended periods in suspension culture may not interfere with neurogenic competence.

### Differentiated cells express transcriptional, morphological and immunological markers of neurogenesis

**Figure 4:**
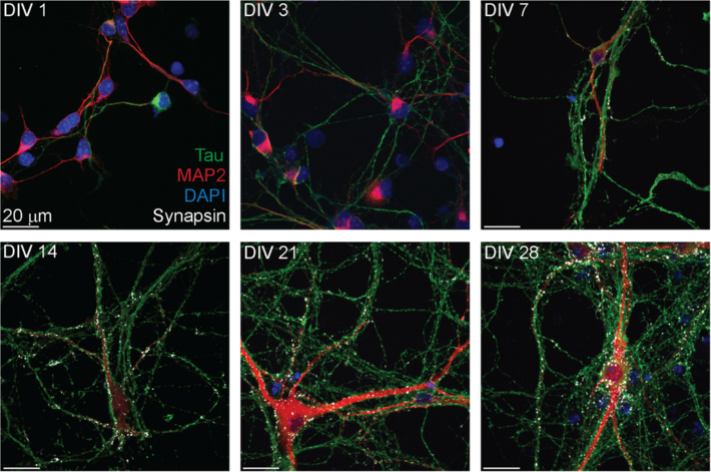
**Immunological characterization of neuron maturation.** ESNs were evaluated at indicated days after plating (DIV) for localization of the dendritic marker MAP2 (red), the axonal marker MAP-tau (green) and presynaptic marker synapsin 1 (white).

**Figure 5:**
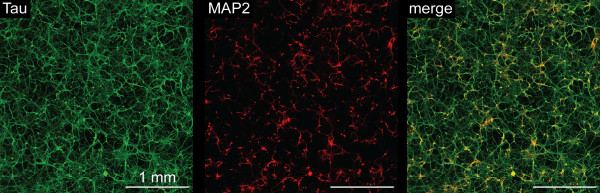
**ESNs develop large, complex axodendritic arbors by DIV 21.** The extent of neuronal network complexity and development was imaged over approximately 9 cm^2^ at DIV 21 by wide-field fluorescent microscopy. Red, dendritic marker MAP2; green, axonal marker MAP-tau. The apposition of mature dendritic and axon processes is demonstrated as co-localized staining (yellow).

Neuronal maturation was characterized between DIV 1–28 using the dendritic marker MAP2, the axonal marker MAP-tau and the pre-synaptic marker synapsin-1 (Figure 4). Although MAP2 was uniformly present at DIV 1, MAP-tau expression was not widely observed in the majority of neurons until DIV 3. Axonal arborization increased extensively between DIV 3–14, while dendritic extension occurred predominantly after DIV 14 (Figure 4). Weak synapsin puncta were widely dispersed along axons at DIV 7, primarily in the absence of proximal dendrites, but as of DIV 14, synapsin-1 staining accumulated at axodendritic interfaces. By DIV 21, an extensive “lawn” of neurites was apparent, with robust axonal arborization and elongated dendrites appearing in close proximity to single or fasciculated axons (Figures 4–5). Greater than 99% of surviving cells expressed neuron-specific markers at DIV 7, and less than one GFAP^+^ glial cell was observed per mm^2^ at DIV 21 (averaged across 25 mm^2^). Glial cells that did survive were most often observed at regions of high neuron density (not shown), suggesting that a supportive microenvironment (e.g., cell-cell contact) may be permissive for glial persistence in the absence of serum.

**Figure 6:**
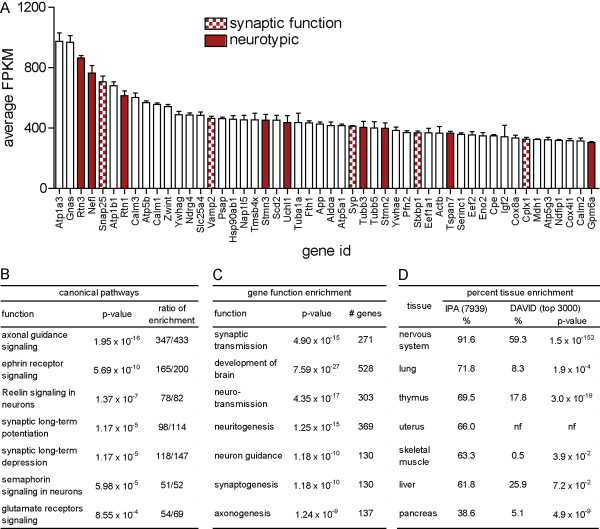
**Characterization of RNA-seq data. A**. Quantification of the fifty most abundant transcripts expressed in DIV 14 ESNs (n = 5 cultures). Neurotypic (red) and synaptic function (checked red) genes are indicated. Error bars indicate standard deviations. **B**. IPA analysis of over-represented canonical pathways, indicating p-values and the number of mapped and identified transcripts identified within each pathway. **C**. IPA analysis of over-represented gene functions, indicating number of mapped and identified transcripts associated with each function. **D**. Tissue-specific gene enrichment from 7939 mapped and identified transcripts with FPKM ≫ 3.0 using Ingenuity Pathway Analysis (IPA). Right: Tissue-specific enrichment among the 3000 most abundant genes determined using DAVID [16,17]. nf, not found.

A presumptive neuronal phenotype was further evaluated by expression profiling at DIV 14. An average of 9,963 ± 42 nuclear mRNA transcripts were detected at a single copy or higher (Additional file 1: Table S1), of which about 1,800 were present in high abundance (≫ 30 FPKM) [15]. Overall, ESNs expressed a broad range of neurotypic genes (Additional file 2: Table S2), and 28% of the most abundant transcripts coded for neuron-specific proteins (Figure 6A). Gene expression was highly enriched for a neuronal phenotype and strongly associated with neuron-specific canonical pathways and functions (Figure 6B-D). ESNs expressed high levels of glutamatergic markers (vGluT2; 100.8 FPKM), with low-to-moderate levels of GABAergic markers (13.9 FPKM and less) and virtually no markers (< 0.4 FPKM) of cholinergic, serotonergic, dopaminergic or motor neuron differentiation. A variety of neurotransmitter receptors were expressed, including those for glycine, GABA, acetylcholine (muscarinic and nicotinic) and glutamate (metabotropic and ionotropic). ESNs also expressed transcripts essential for synaptic activity and electrochemical signal propagation, including subunits of the neuronal N- and P/Q-type voltage-dependent Ca^2+^ channels (VDCCs), SNARE proteins, synaptic vesicle-associated proteins, Na^+^/K^+^ pumps, and a large number of voltage-gated Na^+^, K^+^ and Cl^-^ channels (summarized in Additional file 2: Table S2). 

### Depolarizing stimuli evoke reversible Ca^2+^ uptake

**Figure 7:**
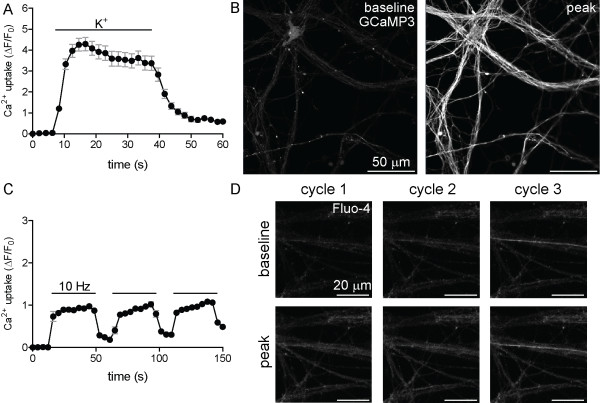
**Depolarization elicits reversible Ca**^**2+ **^**influx.** Ca^2+^ uptake was evaluated following membrane depolarization by KEB (**A**, **B**) or three cycles of electrical field stimulation (10 Hz, 30 sec) (**C**, **D**). Time-lapse imaging of Ca^2+^ uptake (**A**, **C**) under depolarizing conditions, measured using the fluorescent reporter GCaMP3 (**A**; genetically encoded) or Fluo-4 (**C**). Solid black bars in A and C indicate the duration of treatment. Baseline and peak fluorescent intensities are demonstrated by the increase in GCaMP3 (**B**) or Fluo-4 (**D**) fluorescence. Error bars indicate standard error (**A**, **C**).

The expression of gated ion channels and pumps important in maintaining and altering membrane polarity suggested that application of depolarizing stimuli might elicit Ca^2+^ uptake. We altered the plasma membrane potential using indirect (elevated K^+^) and direct (three cycles of electrical field stimulation, 300 pulses per cycle at 10 Hz) methods and found that both methods of depolarization elicited reversible Ca^2+^ influxes (Figure 7). The functional verification of Ca^2+^ uptake in response to classical depolarizing stimuli confirms intracellular recordings and neurotransmitter release assays [11,14], and suggests that ESNs may be sensitive to neurotoxic stimuli whose mechanism of action involves dysregulation of electrochemical signal propagation. 

### ESNs are sensitive to glutamatergic excitotoxicity

**Figure 8:**
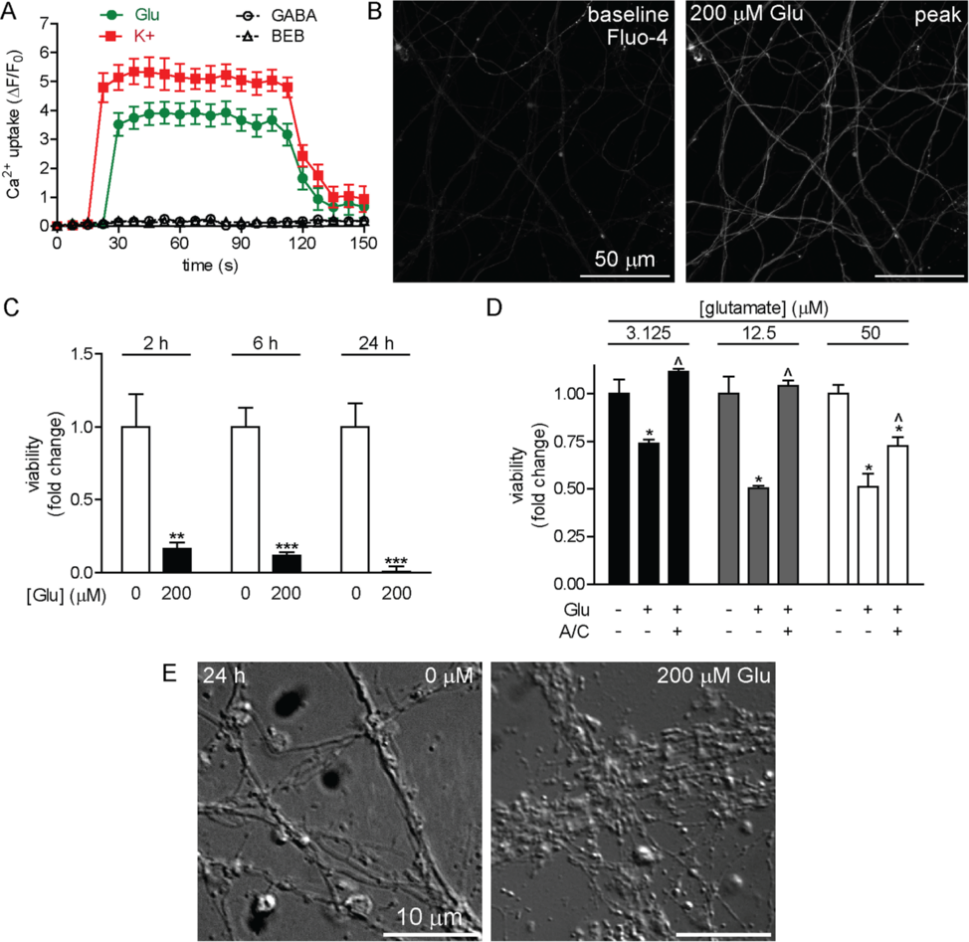
**ESNs exposed to glutamate exhibit acute and chronic sequelae mediated by GluRs. A**. Fluo-4 measurements of Ca^2+^ uptake following the addition of glu (green), GABA (solid, black) or vehicle control (open, black; n ≥ 19 for all samples). K^+^ (red) was used as a positive control. Error bars indicate standard error. **B**. Representative images of Ca^2+^ uptake in ESNs incubated with Fluo-4 and treated with 200 μM glu. **C**. Glu toxicity (200 μM) in ESNs is time-dependent (n=6 for each condition). Data are expressed as fold change relative to control populations. Error bars indicate standard deviation. **D**. Co-administration of ionotropic GluR antagonists APV and CNQX (A/C) blocks glutamate-induced neuron death at 2 h. * indicates P < 0.05 between control and indicated condition. ^ indicates P < 0.05 between glutamate and glutamate plus A/C. (+) and (−) indicate the presence or absence of glutamate or APV/CNQX, respectively. Data are expressed as fold change relative to 0 μM glutamate. **E**. Neurite degeneration and varicosity formation 24 h after exposure to 200 μM glutamate.

Glutamatergic excitotoxicity has been attributed to the pathologic internalization of Ca^2+^ through post-synaptic NMDA receptors, compounded by activation of VDCCs in response to excitatory post-synaptic currents (EPSCs) from AMPA and KA receptors [18,19]. In primary neuron cultures, neurotoxicity has been reported over a wide range of glutamate (glu) doses and exposure durations [20-23]. ESNs express transcripts for NMDA, KA and AMPA receptor subunits and the NMDA-associated protein Grina at high levels (Additional file 1: Table S1), suggesting that ESNs may be functionally sensitive to glu treatment. Treatment of ESNs with 200 μM glu resulted in acute levels of Ca^2+^ uptake similar to those caused by K^+^, whereas the inhibitory neurotransmitter GABA or vehicle controls had no effect (Figure 8A-B). Glu treatment resulted in significant time- (2–24 h, Figure 8C) and dose-dependent (3.125-200 μM; Figure 8D) toxicity, further confirmed by morphological evidence of neurite degeneration 24 h after a 200 μM treatment (Figure 8E). Co-administration of the GluR antagonists APV and CNQX afforded complete protection against toxicity after a 2 h exposure to 3.125 and 12.5 μM glu, and 50% protection against 50 μM glu. These results indicate that ESNs undergo a time- and dose-dependent glu toxicity that is mediated by ionotropic GluRs. 

### Neuron viability and Ca^2+^ influx following exposure to alpha-latrotoxin (LTX)

**Figure 9:**
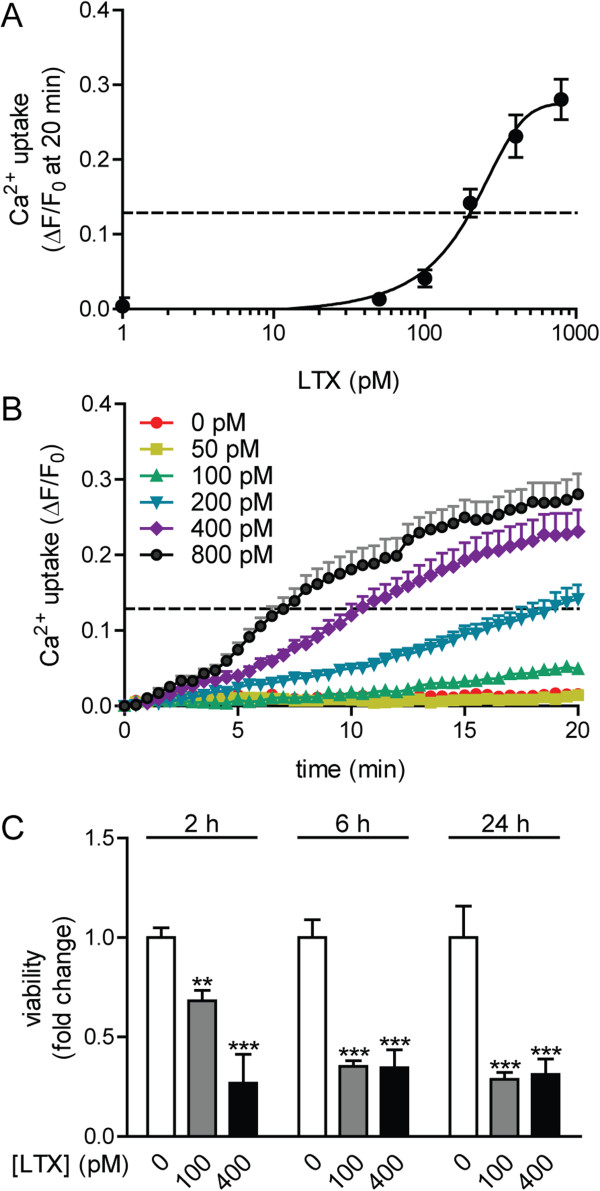
**LTX treatment results in acute Ca**^**2+ **^**influx and loss of metabolic activity. A**. Intracellular Ca^2+^ levels correlate to LTX dose at 20 min after exposure (n = 8). **B**. Longitudinal characterization of Ca^2+^ uptake over a 20 min period after LTX addition (n = 8). **A**, **B**. Dotted line indicates the EC_50_ (representing the dose of LTX that resulted in 50% of the maximum fluorescence) of 174.9 pM. Error bars indicate the standard error. **C**. Dose-dependent reduction in ESN viability 30 min after LTX addition (n = 8). The data are expressed as fold-change relative to 0 μM glutamate. Error bars indicate standard deviations.

Unlike glu exposure, which induces EPSCs in post-synaptic compartments, LTX forms Ca^2+^-permissive pores in the presynaptic membrane that result in fulminant neurotransmitter release and activation of non-synaptic Ca^2+^-sensitive intracellular pathways [24]. ESNs express transcripts of known LTX receptors (PTPRS, neurexin 1–3 and latrophilin 1–3; Additional file 1: Table S1), and LTX treatment of ESNs evokes unregulated Ca^2+^ influx, followed by morphological and biochemical indicators of neurotoxicity [25]. To better characterize LTX toxicity, we evaluated dose-dependent relationships between LTX, onset and magnitude of Ca^2+^ uptake and neuron cell death. The EC_50_ for Ca^2+^ uptake at 20 min was determined to be 174.9 pM (95% C.I. [68.5, 281.3]; Figure 9A), with positive correlations between dose, onset and magnitude of Ca^2+^ uptake (Figure 9B). We also demonstrated a strong correlation between LTX dose and inhibition of metabolic activity after a 30 min exposure (Figure 9C). These findings illustrate the compatibility of ESNs with moderate-throughput screening approaches, confirm that ESN sensitivity and response to LTX are similar to those of primary neurons, and demonstrate that ESNs offer a novel platform with which to screen for therapeutics that prevent LTX toxicity using multi-well formats [26,27]. 

### ESNs are a biologically relevant model of BoNT intoxication

**Figure 10:**
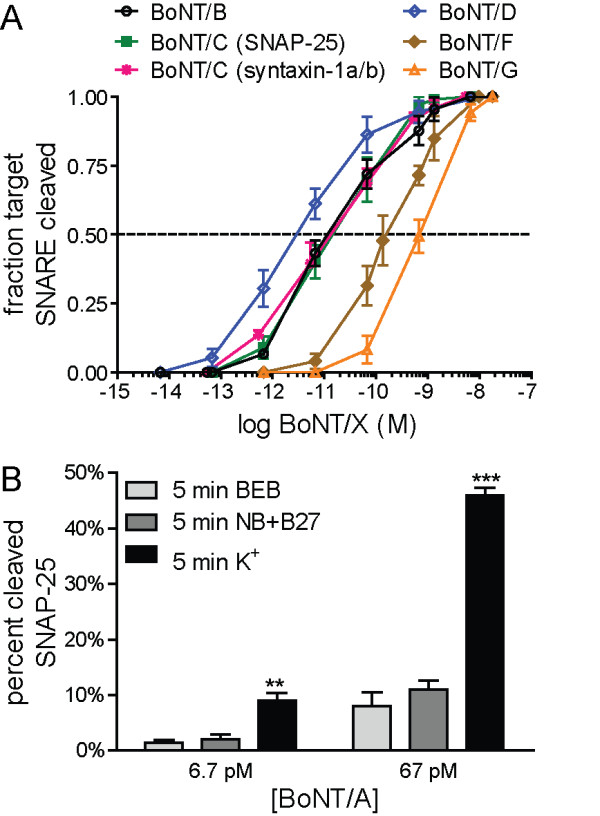
**ESNs exhibit sensitive and specific responses to BoNTs. A**. Log:lin plot calculated from densitometric measurements of SNARE cleavage after a 24 h exposure to 0–17,800 pM BoNT/B, BoNT/C, BoNT/D, BoNT/F and BoNT/G (n ≥ 5 for all time points). **B**. Fractional cleavage of SNAP-25 after exposing ESNs to 6.7 or 67 pM BoNT/A under depolarizing (K^+^) or basal (BEB or NB+B27) conditions (n = 5). Error bars indicate standard error.

ESNs strongly express transcripts and protein for proteolytic targets (SNAP25, VAMP-2 and syntaxin) of the seven BoNT serotypes and known protein receptors for presynaptic uptake (SV2 or synaptotagmin; Additional file 2: Table S2) [28]). We have shown that ESNs are sensitive to femtomolar concentrations of BoNT/A and /E [14]. To determine whether ESNs are a suitable model for the remaining serotypes, we evaluated cleavage of the target SNARE proteins after a 24 h exposure to BoNTs /B, /C, /D, /F and /G (Figure 10A and Additional file 3: Figure S1). ESNs exhibited similar or improved sensitivities to all BoNT serotypes tested compared to primary mouse spinal cord or cerebellar granule cell neurons, and several orders of magnitude improved sensitivity over neuroblastoma cells (Table 1) [29]. BoNT/C is the only holotoxin that targets multiple SNARE proteins; interestingly, we found that intoxication of ESNs with BoNT/C resulted in cleavage of SNAP-25 as well as syntaxin-1 with roughly equivalent EC_50_ values. 

**Table 1:** Sensitivities of primary neurons, SH-SY5Y human neuroblastomas and ESN to the seven classical BoNT serotypes

**BoNT serotype**	**Target SNARE protein**	**Primary neurons EC**_**50**_**(pM)**	**SH-SY5Y EC**_**50**_**(pM)**	**ESNs EC**_**50**_**(pM)**	**95% confidence intervals**	
					**-**	**+**
BoNT/A	SNAP-25	0.4^A^	5562	0.80	0.74	0.86
		10^B^				
BoNT/B	VAMP2	100^B^	41,650	22.50	12.91	39.30
BoNT/C	SNAP-25	13^A^	nr	14.24	9.68	20.93
	Syntaxin-1	nr	nr	7.51	3.06	18.42
BoNT/D	VAMP2	nr	2560	3.09	2.49	3.83
BoNT/E	SNAP-25	36^A^	nr	66.73	52.12	85.44
		43^B^				
BoNT/F	VAMP2	1350^B^	≫300,000	157.82	133.25	186.92
BoNT/G	VAMP2	nr	nr	689.76	615.90	772.48

Notes: Primary neurons’ values are compiled from two sources. Annotation by (**A**) represents 50% inhibition of neurotransmitter release in rat cerebellar neuron cultures exposed to BoNTs for 24 h (a 90% correlation was shown between neurotransmitter release and SNARE integrity) [29], whereas annotation by (**B**) represents cleavage of 50% of SNAP-25 protein in fetal mouse spinal cord cultures exposed to BoNT for 48 h [30]. SH-SY5Y neuroblastomas’ values indicate inhibition of 50% of noradrenalin release in SH-SY5Y human neuroblastomas cells after 72 h incubation [2]. ESN values represent 50% cleavage of SNARE proteins after a 24 h tonic intoxication. ESN EC_50_ values for BoNT/B, /C, /D, /F and /G are from this manuscript, while BoNT/A and /E are from McNutt et al. (2011). nr, not reported.

BoNT holotoxin binds to pre-synaptic receptors and is internalized via synaptic endocytosis. Following cell entry, the proteolytically active light chain is released to the synaptic compartment through pores formed by the heavy chain in the endosome membrane [28]. BoNT uptake and/or activation has been reported to be enhanced following intoxication under depolarizing conditions (presumably the consequence of an accelerated rate of synaptic endocytosis) [28]. We found that a 5 min intoxication of ESNs by BoNT/A in depolarizing media increased SNAP-25 cleavage by five-fold after 24 h as compared to two different basal media (Figure 10B).

## Discussion

### ESC lines remain mitotically active, pluripotent and neurogenic following adaptation to feeder-independent suspension culture

With the aim of developing a more facile, robust and efficient method for the production of homogenous neuronal cultures from mouse ESC lines, we evaluated the neurogenic capacity of ESCs adapted to feeder-independent suspension culture and differentiated under rotary conditions. Mitotic rates of suspension-adapted R1, D3 and C57BL/6 ESC lines were stabilized by five passages, and all ESC lines remained mitotically active and expressed the pluripotency marker Oct3/4 in the absence of feeder cells. Germline competence was not evaluated, so it is unclear whether these culture conditions restrict non-neurogenic developmental fates. Adaptation to suspension culture streamlined and economized ESC culture and neuronal differentiation by obviating the need for feeder cells and allowing the initiation of neuronal differentiation directly from suspension cultures every 48 h. Suspension-cultured ESCs remained neurogenic, and differentiated neurons exhibited a multitude of neurotypic characteristics when maintained in relatively inexpensive, commercially available media. We found that the simple addition of a rotary step during differentiation significantly increased neuron yield to levels sufficient for moderate-throughput screening techniques. These improvements resulted in the generation of sufficient NPCs from one 10-cm dish to plate 776 cm^2^ of tissue culture surface (25 × 96-well dishes) at 125,000 cells per cm^2^.

### Morphological and immunological characterization of neurogenesis

The neurotypic character of ESNs was confirmed using morphology, transcriptional profiling, immunocytochemistry and functional characterization. Temporal changes in MAP2, MAP-tau and synapsin-1 expression and localization demonstrated a characteristic progression through the first 4 stages of neurogenesis, as described by Dotti et al. (1988) in embryonic hippocampal neurons, culminating in a dense axodendritic arbor with punctate localization of synapsin-1 at axodendritic interfaces. As soon as DIV 1, a majority of ESNs produced short MAP2^+^ processes that were indistinguishable from one another, characteristic of development stage (DS) 2 neurons [31,32]. Between DIV 1–3 most ESNs underwent rapid extension of minor neurites and increased expression of axonal markers, characteristic of DS 3 (Figure 4). Axons then underwent rapid extension and arborization (DIV 3–21), accompanied by the onset of dendritic outgrowth and synaptogenesis at DS 4 (DIV 7–14). The earliest appearance of synapsin accumulation at axodendritic interfaces appeared between DIV 7–14, consistent with previous demonstrations of characteristic pre- and post-synaptic architectures at DIV 12 [14].

It should be noted that the plating density is important in producing a neuronal population with few contaminating, non-neuronal cells. Not only does serum withdrawal at DIV 0 initiate the final stages of neuronal differentiation, but it also eliminates the β3-tubulin non-expressing cells, which are likely to be glial cells [14]. Glia that survive beyond DIV 4 tend to be localized to regions of high neuron density. Thus, if NPCs are plated at high density, then increased numbers of glia survive serum-starvation. If unchecked, they can proliferate and eventually overwhelm the ESN culture. We have found that glial contamination due to overplating can be suppressed without detectibly altering neuron viability or function by addition of 5-Fluoro-deoxyuridine/uridine (10 μM each) from DIV 8–12.

During synaptogenesis, presynaptic proteins are bundled and transported along axons in mobile precursors of presynaptic sites known as cytoplasmic transport packets (CTPs) [33,34]. Because synapsin-1 has been identified as a component of CTPs, the abundant distribution of weak synapsin-1 puncta along axons in the absence of proximal MAP2^+^ processes at DIV 7 may represent the presence of CTPs [35]. Interestingly, mobile puncta have been demonstrated to undergo synaptic vesicle recycling in the absence of post-synaptic partners [33,36], possibly accounting for the observation that ESNs are highly sensitive to BoNT/A at DIV 6, prior to the formation of established synapses [14].

### Transcriptional characterization of neurogenesis

Expression profiling and gene enrichment analyses of RNA isolated from DIV 14 ESNs were consistent with immunocytological evidence of neuronal phenotype. ESNs express a large set of neurotypic transcripts, including the post-mitotic nuclear marker NeuN, and abundant transcripts indicative of a vGluT2^+^/vGluT1^-^ glutamatergic neuron subtype, with a smaller fraction expressing GABAergic genes. These findings corroborate previous reports indicating that ≫ 95% of ESNs are vGluT2^+^ and release glutamate in Ca^2+^-dependent fashion under depolarizing stimuli [14]. Consistent with evidence of synaptogenesis, transcripts of genes essential to synapse formation and function are also abundant. For example, SNAP-25, VAMP2 and syntaxin 1a/b are all expressed in the top 2% of all transcripts, emphasizing the importance of neurotransmitter release to neuronal function. ESNs also express a large number of genes essential for neurotransmitter reception and intracellular electrochemical signal propagation, providing a mechanistic basis for previous demonstrations of Ca^2+^-dependent, K^+^-evoked glutamate release and tetrodotoxin-sensitive measurements of action potentials at DIV 12 [11,14].

### Functional characterization of ESN responses to neuron-specific stimuli

A relevant neuron-based research platform must exhibit functional responses and sensitivities to neurotropic stimuli that are consistent with primary neurons. We first demonstrated that two classical methods of membrane depolarization (elevated K^+^ and field stimulation) rapidly induced reversible Ca^2+^ uptake, confirming that ESNs functionally express a wide range of voltage-gated ion channels. ESNs exposed to glutamate or LTX exhibited acute Ca^2+^ influx followed by cell death at doses and timescales similar to those reported in primary neuron populations [20-23,37,38]. These data suggest that ESNs offer a scalable and genetically tractable neuron-based model system that is amenable to characterization of excitotoxic mechanisms and novel identification of therapeutic targets. Furthermore, the ability to evaluate multiple metrics of toxicity in multi-well formats will facilitate moderate-throughput screening for antagonists of NMDA receptor activity, antagonists of LTX pore formation/function or small molecules that mitigate acute or chronic aspects of excitotoxic progression.

Unlike the excitogenic neurotoxins, the BoNTs inhibit neurotransmitter release by cleaving SNARE proteins with exquisite sensitivity. Since intoxication by BoNT holotoxins is highly specific and critically dependent on the function of a large set of neuron-specific proteins within the presynaptic compartment, the sensitivity of a cell population is therefore a reflection of presynaptic function [28]. In combination with previous work, we have now shown that ESNs are sensitive to all seven classical BoNT serotypes, with EC_50_ values that are similar to those of primary neuron cultures and several orders of magnitude improved over neurogenic cell lines (Table 1). To our knowledge, this is the first time that a cell line has been shown to be responsive to all serotypes, including the dual-specificity of serotype /C. These data indicate that ESNs functionally express the necessary receptors, co-factors and substrates for the internalization and activation of all of the BoNT serotypes, and suggest that BoNT is trafficked and processed in a fashion similar to primary neurons.

Although the general mode of action is understood for each of these neurotoxins, efforts to precisely characterize cellular mechanisms of action have been complicated by the lack of a neuromimetic model system that is amenable to modern molecular and biochemical techniques. For the first time, we demonstrate a single model system that is highly sensitive to these neurotoxins at physiologically meaningful concentrations, providing compelling evidence that ESNs are a biologically relevant, functional model for neurotoxicity research. Given that no other neurogenic model has demonstrated comparable sensitivity and neuronal homogeneity to the botulinum neurotoxins [14], ESNs appear to be a novel and relevant model in which to conduct basic research, evaluate therapeutic candidates, determine potencies and diagnose the presence of multiple BoNT serotypes.

## Conclusions

We demonstrate a variant of the 4/4 neuronal differentiation process that results in high yields of enriched neurons, which respond to a variety of neurotropic stimuli in a sensitive and reproducible manner. The robust production of a highly enriched population that exhibits morphological and functional evidence of a neuronal phenotype resolves problems that have hindered moderate-throughput drug screening approaches based on primary neurons (reproducibility, yield and cost) and neurogenic cells (verisimilitude, homogeneity and sensitivity). ESNs do not suffer from allelic variability and are genetically tractable, allowing the interrogation of the role of specific genes in neurogenesis. Repeated production of large quantities of ESNs is relatively simple and offers a highly reductionist model amenable to basic investigations of developmental and functional questions. Unlike dissociated primary cultures derived from neonatal or postnatal CNS tissues, ESN production does not involve disruption of previously established neuronal interactions. Neurogenesis is synchronized and neuron populations are highly enriched, reducing intra- and inter-experimental variability. We anticipate that the ability to recapitulate pathophysiological responses in a relatively homogeneous, scalable and genetically tractable neuron model system will facilitate new approaches to neurotoxicity and neurogenesis research, including detailed investigation into molecular mechanisms of action, “omics”-based discovery and diagnostic screening.

Although more work is needed to characterize neurogenesis and neuron maturation, the apparent similarities between ESNs and dissociated primary neuron cultures suggest that ESNs may also serve as a model system to explore mechanistic aspects of neuron development, synaptogenesis, neurotransmission and plasticity. The same features that make ESNs a productive model for neurotoxicity make them attractive platforms for mechanistic exploration of neurogenic mechanisms.

## Methods

### Reagents

R1, D3 and C57BL/6 ESC lines were obtained from ATCC [39-41]. Pure botulinum holotoxin serotypes /A (2.5 × 10^8^ LD_50_/mg), /B (1.1 × 10^8^ LD_50_/mg), /C (3.5 × 10^7^ LD_50_/mg), /D (0.9 × 10^8^ LD_50_/mg), /F (0.2 × 10^8^ LD_50_/mg) and /G (1.2 × 10^7^ LD_50_/mg) were obtained from Metabiologics (Madison, WI) at 1 mg/mL in Ca^2+^/Mg^2+^-free phosphate buffered saline, pH 7.4 (PBS), and stored at −30°C. In the case of BoNT/G, toxin was first activated by a 60 min incubation at 37°C in 0.05 M sodium phosphate buffer (pH 6.5), 0.3 mg/mL TPCK-treated trypsin (Sigma-Aldrich, St Louis, MO) and 10% glycerol. Activated toxin was diluted 1:1 with soybean trypsin inhibitor and stored at −30°C until use. α-Latrotoxin (LTX; Sigma-Aldrich) was resuspended to 300 nM in H_2_O and stored at −20°C. Mono-sodium glutamate (Sigma-Aldrich) and γ-aminobutyric acid (GABA; Sigma-Aldrich) were resuspended to 20 mM in PBS and stored at 4°C. Solutions were diluted to the indicated concentrations in basal electrophysiologic buffer (BEB; 10 mM glucose, 1 mM MgCl_2_, 10 mM HEPES, 2 mM CaCl_2_, 3 mM KCl, 136 mM NaCl and 0.1% BSA, pH 7.4, 310 ± 10 mOsm; Sigma-Aldrich). R-2-amino-5-phosphonopentanoate (AVP, 50 μM, Sigma) and 6-cyano-7-nitroquinoxaline-2,3-dione (CNQX, 10 μM, Sigma) were prepared in BEB and added 1 h prior to and concurrent with the addition of glutamate. Fluo-4 (Life Technologies, Carlsbad, CA) was prepared per the manufacturer’s instructions. Neurons were maintained in BEB during time-lapse imaging. High potassium electrophysiologic buffer (KEB) was prepared similarly to BEB, except with substitutions of 60 mM KCl and 79 mM NaCl. Electrical field stimulation (1 msec, 100 mV pulses at 10 Hz) of neurons on 18-mm cover slips was applied via a field stimulation perfusion chamber (PC-49FS; Warner Instruments, Hamden, CT) with a Grass S88 stimulator (Grass Medical Instruments, Quincy, MA).

### Suspension adaptation and continuous mESC culture

R1, D3 and C57BL/6 cell lines that had been previously maintained in adherent culture with mouse embryonic fibroblasts (MEFs) were thawed and maintained at 37°C at 5% CO_2_ in 90% relative humidity in 10-cm bacterial plates in 10 mL ESM (Knockout DMEM supplemented with 100 uM β-mercaptoethanol, 15% ES qualified fetal calf serum [ATCC], 0.1mM nonessential amino acids, 2.0 mM L-glutamine and 5000 units/mL penicillin/streptomycin [Life Technologies] and 1000 units/mL recombinant mouse leukemia inhibitory factor [LIF; Chemicon International, Temecula, CA]). Alternatively, ESCs were maintained in commercially prepared complete ESC medium (Millipore, Billerica, MA). Cells were observed daily and passaged once aggregates became clearly visible (typically 4–8 days). Cells that were adherent or failed to divide during adaptation were discarded. Surviving aggregates were trypsinized, and 1.5 × 10^6^ cells were inoculated into a fresh 10-cm bacterial dish with 10 mL ESM. ESCs were subcultured every 48 h. For passaging, aggregates were allowed to settle by gravity, washed once with 0.5 mL PBS and dissociated for 3 min at 37°C with 0.5 ml of TrypLE Express (Life Technologies). Dissociation was terminated with 0.5 mL of ESM, cells were gently triturated and 1.5 × 10^6^ mESCs were transferred to a fresh 10-cm dish.

### Generation of GCaMP3-expressing ESCs

Suspension-adapted R1 ESCs were stably transfected with a genetically encoded Ca^2+^ construct (GCaMP3) driven by the synthetic CAG promoter (Addgene plasmid 22692, Cambridge, MA) [42]. Five μL Lipofectamine 2000, 5 μg plasmid and 5 μL PLUS reagent were prepared per manufacturer’s instructions (Invitrogen) in a total volume of 100 μL DMEM and added to 100 μL of 1 × 10^6^ ESCs/mL in DMEM for 10 min at 37°C. Suspensions were transferred to 10 mL ESM in a bacterial dish and returned to an incubator. Media was changed at 1 d and G418 selection (250 μg/mL; Sigma-Aldrich) started at 2 d. Transient transfection rates exceeded 40%, as estimated from basal fluorescence levels at 2d. Media was subsequently changed every 4 d until G418-resistant ESC aggregates developed. Stably transfected ES cell aggregates (20–50 per transfected population) were isolated and cultured as above until differentiation.

### Neuronal differentiation

A modified 4/4 protocol was employed to differentiate ESCs into neural progenitor cells [11,14]. Following routine sub-passaging, 3.5 × 10^6^ dissociated ESCs were transferred to 25 mL of differentiation medium (ESM modified with 10% ES qualified fetal calf serum and without LIF) in a 10-cm ultra-low attachment culture dish (Corning, Lowell, MA). Differentiating aggregates were maintained on a rotary shaker at 45 rpm at 37°C, 5% CO2 and 90% relative humidity. Complete media changes were conducted at 48 h intervals, with the addition of 6 μM retinoic acid (Sigma-Aldrich) at 4 and 6 days after starting differentiation.

On DIV 0, aggregates were dissociated with TrypLE Express for 5 min at 37°C. Trypsinization was halted by adding 5 mL of 1% soybean trypsin inhibitor (Life Technologies), the aggregates were gently dissociated by triturating with a 10 mL pipet, and the cell suspension was filtered through a 40-μm cell strainer (Thermo Scientific). Cells were pelleted for 5 min at 300 × g, washed in N2 medium (Neurobasal-A medium with 1x N2 vitamins, 2 mM glutamine and antibiotics [Life Technologies]) and counted. Cells were plated at 125,000 (coverslips) or 150,000 cells/cm^2^ (dishes) in N2 medium. Complete washes were conducted at 4 h and 24 h to remove residual serum, gliotrophic factors secreted by glial cells and non-adherent cells, and at 48 h after plating (DIV 2), N2 was replaced with B27 medium (Neurobasal-A supplemented with antibiotics, 2 mM glutamine and 1x B27 vitamins [Life Technologies]). Subsequently, cells underwent full medium changes with B27 on DIV 4 and 8, and then 50% media changes with B27 every fifth day. Glial cell elimination by serum-starvation starting at DIV 0 resulted in the loss of roughly 30% of plated cells between DIV 2–4 [14].

Tissue culture treated dishes ranging in size from 10 cm to 24-well plates were prepared by coating with 0.5 μg/mL poly-D-lysine (PDL, Sigma-Aldrich) for at least 3 h, followed by two quick washes with sterile ddH_2_0 and storage in N2 at 37°C until plating. Coverslips (18 mm, Thermo Fisher Scientific, Waltham, MA) were coated with 200–300 μL of 0.5 μg/mL PDL for 24 h at 37°C followed by 5 μg/mL laminin (Sigma-Aldrich) in Knockout DMEM for 3 h and transferred coated-side up to 12-well dishes. All characterization of differentiated neurons was conducted exclusively with neurons derived from R1 ESCs between 5–30 passages.

### Immunoblotting

ESN cultures were lysed with 250 μL denaturing cell extraction buffer (Life Technologies) and clarified by centrifugation through a Qiashredder (Qiagen, Valencia, CA); total protein concentration was determined by bicinchoninic acid (BCA) analysis (Thermo Scientific, Rockford, IL). Fifteen micrograms of total protein was separated on a 10% (BoNT/B, BoNT/D, BoNT/F or BoNT/G) or 12% (BoNT/A or /C) Nupage gel (Life Technologies) with MOPS running buffer. Gels were transferred to PVDF and probed for SNARE proteins with a mouse anti-SNAP-25 antibody (SMI81; Covance, Gaithersburg, MD), a mouse anti-VAMP2 antibody (Synaptic Systems, Gottingen, Germany), and a mouse anti-Syntaxin-1 (Synaptic Systems) diluted 1:1000 in TBS Superblock with 0.05% Tween-20 (TBST; Life Technologies). Proteins were visualized with goat anti-mouse Alexa-488 labeled antibodies diluted 1:2500 in TBST and imaged with a Versadoc MP4000 (Bio-Rad, Hercules, CA).

### Time-lapse microscopy

Images were collected on a Zeiss LSM-700 confocal microscope with a constant-temperature environmental chamber. For Fluo-4 staining, ESNs on 18-mm coverslips were stained and loaded in a perfusion chamber as previously described [25]. Zen 2009 (Carl Zeiss, Inc, Oberkochen, Germany) was used to determine the mean fluorescence intensity over a fixed area using Fluo-4 and GCaMP3 as indicators for the presence of cytosolic Ca^2+^. Ca^2+^ uptake was induced with the application of KEB or LTX at indicated quantities or by field stimulation. To elicit action potentials by field stimulation, electrical current was applied through platinum wires located in the microscope field of view, using 1 msec, 100 mV pulses. Neurons were stimulated with 3 cycles of approximately 300 action potentials at 10 Hz, with a 10 sec rest. The data were normalized to ΔF/F_0_ via the following equation: *y* = (*F*_*Δ* min_ − *F*_0  min_)/*F*_0  min_.

### Plate reader based quantification of Ca^2+^ uptake

ESNs were plated in either 24- or 48-well dishes, stained and mounted as previously described and maintained at 25°C [25]. Ca^2+^ uptake was induced with the application of chemical stimulation at indicated concentrations. Changes in fluorescence were monitored with a Synergy MX plate reader (BioTek Instruments, Inc., Winooski, VT) with excitation of 490/10 nm and emission of 520/10 nm. The data were normalized via the following equation: *y* = (*F*_*x*_ − *F*_0_)/*F*_0_.

### Immunocytochemistry

Coverslips were fixed with 4% paraformaldehyde for 15 min at room temperature and blocked and permeabilized for 10 min in PBS with 0.1% saponin and 3% bovine serum albumin (BSA) (PBSS). Coverslips were incubated for 1 h with anti-MAP-Tau and anti-MAP2 primary antibodies (Synaptic Systems, Gottingen, Germany) diluted 1:1000 and 1:500, respectively, in PBSS, washed three times with PBSS and incubated for 1 h with goat anti-mouse or anti-rabbit Alexa-labeled secondaries (Invitrogen) diluted 1:500 in PBSS. Coverslips were washed three times in PBSS and incubated for 1 h with anti-synapsin 1-Oyster 650 primary antibody (Synaptic Systems) diluted 1:500 in PBSS. Coverslips were washed three times in PBS and mounted with Prolong Gold DAPI mounting media (Life Technologies). Images were collected with a Zeiss LSM 700 confocal microscope or Zeiss LSM 510 confocal microscope and analyzed with Zen 2009 or 2008, respectively.

### Differential interference contrast (DIC)/bright field microscopy

For evaluation of neuronal morphology, images were collected on a Zeiss LSM 700 confocal microscope with a constant temperature chamber and analyzed with AxioVision LE release 4.8.1. For evaluation of aggregate morphology, images were collected on a Zeiss AxioObserver.A1 epi-fluorescence microscope and analyzed with AxioVision release 4.7.1.

### Flow cytometry

mESCs were collected during routine subculture and washed twice with PBS. Cells were fixed with paraformaldehyde, permeabilized and stained for Oct3/4 expression or with appropriate isotype controls using the Human and Mouse Pluripotent Stem Cell Analysis Kit (BD Biosciences, San Jose, CA). Stained cells were analyzed on a BD FACSAria II flow cytometer using manufacturer-specified laser excitation wavelength and emission filter sets. At least 10,000 gated events were recorded and analyzed for each sample. Density plots and fluorescence intensity histograms were generated using FCS Express, and experimental samples were normalizing to isotype controls.

### Quantitation of ESN viability

ESNs were plated in either 24- or 48-well dishes and maintained under indicated conditions. PrestoBlue (Invitrogen) was added per manufacturer’s protocols in fresh NBA-B27, and ESNs were incubated for 45 min at 37°C. Metabolic conversion of PrestoBlue was measured using an excitation of 535 nm and emission of 595 nm with a Synergy MX plate reader.

### Expression profiling

RNA was harvested from 10-cm dishes of DIV 14 ESNs (n=5) using RNeasy mini kit (Qiagen) and submitted to Expression Analysis (Durham, NC) for RNA sequencing (2 × 25 paired-end, on an Illumina HiSeq 2000 [San Diego, CA]). Transcriptome data were aligned using UCSC’s mouse knowngene transcriptome with Tophat. Unaligned reads were then aligned to the genome using BWA and merged with the Tophat alignments [43]. Transcript abundances were determined using RSEM v1.1.13 and normalized by calculating the fragments of exon per million mapped reads (FPKM) [44,45]. Normalized data were analyzed for enrichment of canonical pathways, gene function and tissue expression by Ingenuity Pathway Analysis (Ingenuity Systems, Redwood City, California). The most abundant 3000 transcripts were also analyzed for gene functional classification and tissue enrichment using Database for Annotation, Visualization, and Integrated Discovery (DAVID) [16,17].

### Statistics

BoNT and LTX EC_50_ values were calculated using a four -parameter sigma model from average dose response values determined from densitometry of western blot images and presented as median values with 95% confidence intervals (C.I.). Multiple comparisons were performed using one-way analysis of variance (ANOVA) to determine significance. Differences among means were determined and calculated with the Student's t-test. * indicates a *P* < 0.05. ** indicates a *P* < 0.01. *** indicates a *P* < 0.001. IPA and DAVID generated the p-values adjusted for multiple hypotheses.

## Abbreviations

ESN: Embryonic stem cell-derived neuron; ESC: Embryonic stem cell; NPC: Neural progenitor cell; BoNT: Botulinum neurotoxin; LTX: α-latrotoxin; LIF: Leukocyte inhibitory factor; RA: Retinoic acid; FPKM: Fragments per kilobase of transcript per million mapped reads; Glu: Glutamate.

## Competing interests

The authors declare that they have no competing interests.

## Authors’ contributions

PMM conceived the project. PMM, KSH, MTM and IMG designed experiments. PMM, IMG, MEL, KSH, MTM and KMT performed experiments and analyzed data. PMM, IMG and KSH wrote the manuscript. All authors read and approved the final manuscript.

## Supplementary Material

Additional file 1: Table S1 Normalized transcript expression for DIV 14 ESNs. Gene names, average FPKMs, standard deviations and coefficient of variations for DIV 14 ESN samples (n=5). Click here for file

Additional file 2: Table S2 Summary of neurotypic gene expression in DIV 14 ESNs. Representative transcripts involved in neuron function, neurogenesis or neurotoxin function are included. Highlighted sequences represent abundant transcripts, defined as an average reads per kilobase of exon per million mapped sequences (FPKM) exceeding 30. Click here for file

Additional file 3: Figure S1 Representative immunoblots demonstrating dose-dependent proteolysis of target specific SNARE proteins 24 h after exposure. Click here for file
