# Supplementary material for: High yield derivation of enriched glutamatergic neurons from suspension-cultured mouse ESCs for neurotoxicology research
Source: BMC Neurosci. 2012 Oct 24;13:127. doi: 10.1186/1471-2202-13-127 (PMC3573964; doi:10.1186/1471-2202-13-127)
Supplement: Additional file 1: Table S1 — Normalized transcript expression for DIV 14 ESNs. Gene names, average FPKMs, standard deviations and coefficient of variations for DIV 14 ESN samples (n=5). [file 1471-2202-13-127-S1.pdf]

Average RPKMs for all transcripts

RKPM: reads per kilobase of exon per million mapped sequences

Gene name	Av RKPM	SD	CoV
40603	11.67626	0.48	4.1%
40604	14.62324	1.31	8.9%
40605	2.737656	0.21	7.8%
40606	8.5183	0.56	6.5%
40607	35.36966	1.28	3.6%
40608	83.4831	3.37	4.0%
40609	14.68758	0.92	6.2%
40610	10.712166	0.62	5.8%
40611	14.50206	1.03	7.1%
40612	0.06923812	0.02	23.5%
40613	2.908098	0.53	18.1%
40787	0.06023374	0.03	45.3%
40788	24.73104	1.95	7.9%
40789	117.9616	7.04	6.0%
40790	9.558842	1.79	18.7%
40791	110.3324	6.26	5.7%
40792	37.93056	2.00	5.3%
40793	85.82438	1.35	1.6%
40794	17.90124	1.01	5.6%
40795	19.03982	1.56	8.2%
40796	1.504728	0.19	12.4%
40797	36.03812	2.59	7.2%
40798	0	0.00	
40800	2.392	0.18	7.6%
40801	71.90914	3.73	5.2%
40817	0.987742	2.19	221.2%
0610006I08Rik	32.01456	4.00	12.5%
0610007C21Rik	27.3846	0.95	3.5%
0610007L01Rik	19.01198	0.76	4.0%
0610007P08Rik	2.648974	0.17	6.3%
0610007P14Rik	28.26806	2.93	10.4%
0610007P22Rik	9.939696	0.83	8.3%
0610008C08Rik	1.1400194	0.30	26.5%
0610008F07Rik	0	0.00	
0610009B22Rik	14.63654	1.13	7.8%
0610009D07Rik	20.81844	1.30	6.2%
0610009K11Rik	9.497214	0.34	3.5%
0610009O03Rik	6.088442	0.85	13.9%
0610009O20Rik	12.75618	0.23	1.8%
0610010D20Rik	0.05401106	0.05	92.2%
0610010D24Rik	0.23117952	0.24	104.8%
0610010E21Rik	5.070526	0.52	10.3%

0610010F05Rik	20.66784	0.98	4.7%
0610010K06Rik	0	0.00	
0610010K14Rik	9.889694	1.41	14.3%
0610010O12Rik	19.91588	1.56	7.8%
0610011F06Rik	8.04009	0.15	1.9%
0610011L14Rik	9.380354	0.48	5.1%
0610012D14Rik	0.3637354	0.14	39.1%
0610012D17Rik	4.80818	0.90	18.7%
0610012G03Rik	19.5748	1.43	7.3%
0610012H03Rik	0.0047451	0.01	223.6%
0610013E23Rik	1.934256	0.28	14.7%
0610025P10Rik	18.77406	0.42	2.2%
0610030E20Rik	1.861344	0.27	14.7%
0610031J06Rik	12.1006	0.92	7.6%
0610037D15Rik	0.8553186	0.18	21.2%
0610037L13Rik	13.5498	0.66	4.9%
0610037P05Rik	20.44222	1.75	8.5%
0610038D11Rik	6.131326	0.65	10.6%
0610038F07Rik	21.1709	0.89	4.2%
0610040J01Rik	0.2318094	0.02	9.7%
0710005I19Rik	12.177524	1.47	12.1%
0710008K08Rik	15.4478	0.49	3.2%
0910001A06Rik	60.53192	3.34	5.5%
0910001L09Rik	23.1696	1.04	4.5%
1100001E04Rik	15.30468	0.86	5.6%
1100001G20Rik	0	0.00	
1100001H23Rik	0.06120732	0.02	25.5%
1110001A07Rik	7.177454	0.77	10.7%
1110001A16Rik	6.049946	0.58	9.6%
1110001D15Rik	0.03658288	0.01	14.8%
1110001J03Rik	22.8624	1.08	4.7%
1110002B05Rik	19.54778	0.92	4.7%
1110002E23Rik	43.70396	2.78	6.4%
1110002H13Rik	0.01619152	0.02	94.5%
1110002N22Rik	3.10692	0.37	11.7%
1110003E01Rik	41.88292	1.69	4.0%
1110004E09Rik	6.723898	0.78	11.6%
1110004F10Rik	34.26432	3.87	11.3%
1110005A03Rik	2.26168	0.15	6.8%
1110005A23Rik	3.816438	0.74	19.3%
1110006G06Rik	12.433106	5.99	48.2%
1110006O17Rik	0.03219452	0.02	69.1%
1110007A13Rik	5.807704	0.22	3.8%
1110007C09Rik	3.927842	0.68	17.3%
1110007L15Rik	11.54428	0.68	5.9%
1110007M04Rik	8.82549	0.85	9.6%
1110008F13Rik	31.15504	2.39	7.7%

1110008I14Rik	0	0.00	
1110008J03Rik	6.245366	0.63	10.1%
1110008L16Rik	2.418618	0.13	5.4%
1110008P14Rik	48.51764	4.77	9.8%
1110012D08Rik	4.589816	0.23	4.9%
1110012J17Rik	4.496836	0.17	3.7%
1110012L19Rik	6.918752	0.45	6.5%
1110012M11Rik	9.201792	0.68	7.4%
1110012N22Rik	0.1772018	0.05	27.2%
1110013L07Rik	15.05462	0.43	2.9%
1110014J01Rik	7.06459	0.56	7.9%
1110014K08Rik	1.678074	0.17	10.3%
1110014N23Rik	21.93458	1.22	5.6%
1110015M06Rik	0	0.00	
1110017D15Rik	0.3522858	0.09	26.6%
1110017I16Rik	0.02260084	0.03	142.0%
1110018G07Rik	22.45992	1.31	5.9%
1110018H23Rik	0	0.00	
1110018J18Rik	3.357416	0.66	19.7%
1110018J23Rik	0.1500428	0.03	22.3%
1110018M03Rik	0.6893744	0.14	20.5%
1110019J04Rik	15.27464	1.89	12.4%
1110019K23Rik	5.63765	0.28	4.9%
1110019N10Rik	12.46444	1.04	8.4%
1110020C03Rik	0.08147936	0.05	57.4%
1110020G09Rik	7.24562	0.36	4.9%
1110020P15Rik	79.82052	5.30	6.6%
1110021J02Rik	7.944344	1.24	15.6%
1110028A07Rik	0.02034882	0.02	94.3%
1110028C15Rik	2.111648	0.27	12.9%
1110029E03Rik	6.447544	0.38	5.9%
1110031B06Rik	188.6826	2.94	1.6%
1110031I02Rik	4.640134	0.13	2.8%
1110032A03Rik	11.06716	0.78	7.1%
1110032A04Rik	3.847072	0.41	10.6%
1110032A13Rik	12.29006	1.01	8.2%
1110032E23Rik	1.94055	0.21	10.7%
1110032O16Rik	7.362976	1.01	13.7%
1110033F04Rik	0	0.00	
1110033J19Rik	15.65934	2.16	13.8%
1110033M05Rik	9.875628	0.52	5.3%
1110034A24Rik	1.319254	0.17	12.9%
1110034B05Rik	5.428918	0.40	7.4%
1110034G24Rik	3.522136	0.20	5.6%
1110036O03Rik	3.62982	0.27	7.5%
1110037F02Rik	8.642296	0.40	4.6%
1110038D17Rik	5.337024	0.61	11.5%

1110038F14Rik	6.605574	0.63	9.5%
1110039B18Rik	20.06066	0.53	2.7%
1110049B09Rik	0.04198018	0.04	89.7%
1110049F12Rik	45.05524	2.60	5.8%
1110051B16Rik	0	0.00	
1110051M20Rik	19.9846	4.29	21.5%
1110054O05Rik	3.94987	0.19	4.9%
1110054P19Rik	0	0.00	
1110057K04Rik	12.68052	0.38	3.0%
1110058L19Rik	8.835994	0.37	4.1%
1110059E24Rik	15.43236	0.75	4.8%
1110059G10Rik	1.962244	0.18	9.2%
1110059H15Rik	2.3429852	1.61	68.7%
1110061O04Rik	0.1934406	0.07	37.1%
1110064P04Rik	1.0300064	0.61	59.5%
1110067D22Rik	31.48432	1.82	5.8%
1110067I12Rik	1.537228	0.18	12.0%
1190002A17Rik	0.7582484	0.18	23.9%
1190002H23Rik	0.2379762	0.08	32.5%
1190002N15Rik	5.956218	0.20	3.3%
1190003J15Rik	0.0154409	0.02	140.8%
1190003M12Rik	0.12587772	0.06	44.4%
1190005F20Rik	7.57087	0.35	4.7%
1190005I06Rik	1.4194672	0.41	28.9%
1190005P17Rik	2.575862	0.27	10.4%
1190017O12Rik	5.843518	0.43	7.4%
1190020J12Rik	0.2949146	0.08	27.0%
1200002N14Rik	0.6087352	0.23	38.0%
1200003C05Rik	29.28896	1.89	6.5%
1200003I07Rik	7.087236	0.13	1.9%
1200004M23Rik	2.902572	0.23	7.9%
1200009F10Rik	7.860752	0.69	8.8%
1200009I06Rik	0.1615962	0.02	12.5%
1200009O22Rik	5.552574	0.53	9.6%
1200011I18Rik	8.365534	0.30	3.6%
1200011M11Rik	6.43495	0.31	4.9%
1200011O22Rik	3.092196	0.21	6.9%
1200013B08Rik	0.004136656	0.01	137.4%
1200013P24Rik	41.40306	2.09	5.0%
1200014J11Rik	5.719202	0.24	4.1%
1200014M14Rik	12.43544	0.24	1.9%
1200015A19Rik	37.7796	1.40	3.7%
1200015F23Rik	17.69728	0.85	4.8%
1200015N20Rik	6.263466	0.48	7.6%
1200016B10Rik	1.306862	0.09	6.7%
1300001I01Rik	32.31458	0.76	2.3%
1300002K09Rik	0.01484772	0.01	92.3%

1300003B13Rik	7.442572	0.43	5.7%
1300007F04Rik	0.05440566	0.03	57.6%
1300010F03Rik	13.10772	0.78	5.9%
1300010M03Rik	18.43024	0.58	3.2%
1300012G16Rik	17.07374	1.40	8.2%
1300014I06Rik	2.900172	0.17	5.7%
1300017J02Rik	0.02417306	0.02	64.4%
1300018I05Rik	23.66636	1.10	4.6%
1300018I17Rik	15.30768	0.32	2.1%
1300018J18Rik	8.113044	0.24	2.9%
1500001A10Rik	5.973948	0.50	8.4%
1500001M20Rik	4.194662	0.29	6.8%
1500002O20Rik	4.65949	0.49	10.5%
1500003O03Rik	26.5808	1.27	4.8%
1500003O22Rik	4.897882	0.34	6.9%
1500005A01Rik	8.706684	0.59	6.8%
1500005I02Rik	0.9168604	0.06	6.1%
1500010J02Rik	11.509	0.89	7.7%
1500011B03Rik	57.1211	2.50	4.4%
1500011H22Rik	29.67984	2.14	7.2%
1500012F01Rik	11.75096	0.92	7.8%
1500015O10Rik	3.274808	0.88	27.0%
1500016O10Rik	0.12042764	0.05	44.8%
1500019G21Rik	34.9358	2.07	5.9%
1500031I19Rik	77.65288	6.03	7.8%
1500031L02Rik	12.95436	0.49	3.7%
1500032D16Rik	79.7215	3.86	4.8%
1500032L24Rik	89.31526	8.07	9.0%
1500034J01Rik	36.01696	3.60	10.0%
1500035H01Rik	11.66006	0.33	2.9%
1500041B16Rik	18.05466	2.36	13.0%
1500041N16Rik	7.097336	0.22	3.0%
1520401A03Rik	0.016259634	0.01	49.1%
1600002H07Rik	2.103546	0.88	41.9%
1600002K03Rik	1.849022	0.20	10.6%
1600002O04Rik	5.170354	0.24	4.6%
1600012F09Rik	1.87956	0.37	19.4%
1600012H06Rik	9.921056	2.45	24.7%
1600012P17Rik	0	0.00	
1600014C10Rik	12.69996	0.24	1.9%
1600014C23Rik	0.04102774	0.07	166.6%
1600014E20Rik	0.00415768	0.01	223.6%
1600014K23Rik	0	0.00	
1600015H20Rik	0.3262128	0.11	35.2%
1600015I10Rik	0	0.00	
1600016N20Rik	0.10771272	0.05	42.2%
1600021P15Rik	20.96076	2.01	9.6%

1600027N09Rik	3.12152	0.36	11.5%
1600029D21Rik	0.04316328	0.03	67.4%
1600029I14Rik	0	0.00	
1700001C02Rik	0	0.00	
1700001C19Rik	0.05964266	0.02	37.5%
1700001F09Rik	0.0123066	0.02	137.6%
1700001G17Rik	0.1585662	0.08	52.9%
1700001J03Rik	0.01658234	0.02	140.6%
1700001K19Rik	0.206083	0.07	32.2%
1700001L19Rik	1.2215938	0.24	19.7%
1700001O22Rik	0.07041344	0.07	101.2%
1700001P01Rik	0.02479292	0.04	152.9%
1700003E16Rik	2.5264	0.25	9.8%
1700003F12Rik	0.04388062	0.03	77.3%
1700003M02Rik	0.07189868	0.03	42.3%
1700006A11Rik	0	0.00	
1700006E09Rik	0.02594286	0.04	142.3%
1700007B13Rik	0	0.00	
1700007B14Rik	0.01808648	0.02	94.9%
1700007G11Rik	0.05097332	0.06	113.7%
1700007I06Rik	0.10786298	0.06	56.3%
1700007K09Rik	0.0753224	0.03	33.7%
1700007K13Rik	2.546446	0.43	17.0%
1700007N14Rik	0	0.00	
1700008A04Rik	0	0.00	
1700008F21Rik	0.01075238	0.01	137.0%
1700008G05Rik	0	0.00	
1700008I05Rik	0.00380754	0.01	223.6%
1700008P02Rik	0	0.00	
1700008P20Rik	0.16188416	0.08	51.1%
1700009J07Rik	0.00742776	0.02	223.6%
1700009N14Rik	0	0.00	
1700009P17Rik	0.09097164	0.05	50.0%
1700010A17Rik	0.3116392	0.11	35.0%
1700010C24Rik	5.946768	0.62	10.4%
1700010D01Rik	0	0.00	
1700010H22Rik	0	0.00	
1700010I14Rik	0.1888154	0.04	23.0%
1700010M22Rik	0	0.00	
1700011A15Rik	0.00532488	0.01	223.6%
1700011E24Rik	0.02029968	0.03	147.6%
1700011F03Rik	0	0.00	
1700011F14Rik	0	0.00	
1700011H14Rik	0.0047332	0.01	223.6%
1700011I03Rik	0	0.00	
1700011L22Rik	0.0045537	0.01	223.6%
1700012A03Rik	0	0.00	

1700012A16Rik	0.08500926	0.02	17.8%
1700012B07Rik	0	0.00	
1700012B09Rik	0	0.00	
1700012B15Rik	9.703002	0.39	4.1%
1700012G19Rik	24.46534	2.20	9.0%
1700012H17Rik	13.44892	0.42	3.2%
1700012L04Rik	0	0.00	
1700012P22Rik	0.01776576	0.02	139.2%
1700013B16Rik	0.004544204	0.01	158.9%
1700013D24Rik	0	0.00	
1700013E18Rik	0.9264202	0.09	9.8%
1700013F07Rik	0.222369	0.09	42.5%
1700013G24Rik	0	0.00	
1700013H16Rik	0	0.00	
1700013N18Rik	0.02328834	0.04	171.5%
1700014N06Rik	0	0.00	
1700015E13Rik	0.00740286	0.02	223.6%
1700016C15Rik	0	0.00	
1700016D06Rik	0	0.00	
1700016G05Rik	0	0.00	
1700016H13Rik	0.00512942	0.01	223.6%
1700016K19Rik	0.11558846	0.07	58.4%
1700016M24Rik	0.004610418	0.01	144.1%
1700017N19Rik	0	0.00	
1700018B08Rik	0	0.00	
1700018B24Rik	0	0.00	
1700018C11Rik	0.02218894	0.02	91.9%
1700018F24Rik	0	0.00	
1700018L24Rik	0	0.00	
1700019A02Rik	0	0.00	
1700019B03Rik	0.04855604	0.05	97.3%
1700019D03Rik	2.296648	0.55	24.1%
1700019E19Rik	2.635594	0.39	14.8%
1700019G17Rik	0.7556788	0.04	5.0%
1700019H03Rik	0.04853276	0.02	37.3%
1700019L03Rik	0.10672498	0.02	21.2%
1700019M22Rik	0	0.00	
1700019N12Rik	1.460211	0.41	28.4%
1700019N19Rik	0	0.00	
1700019O17Rik	0.00295684	0.01	223.6%
1700019P01Rik	0.04766142	0.02	35.3%
1700020A23Rik	0.0315071	0.03	103.1%
1700020C07Rik	0.00582872	0.01	223.6%
1700020C11Rik	18.02438	0.94	5.2%
1700020D05Rik	0.3861852	0.11	29.0%
1700020L24Rik	0.2880794	0.11	38.4%
1700020N01Rik	0	0.00	

1700020O03Rik	8.762996	0.60	6.8%
1700021C14Rik	1.2264722	0.32	26.2%
1700021F05Rik	14.38508	1.07	7.5%
1700021F07Rik	0	0.00	
1700021K02Rik	0	0.00	
1700021K14Rik	0.03876596	0.04	102.3%
1700021K19Rik	13.87452	0.66	4.7%
1700021P22Rik	2.452546	0.64	26.0%
1700022C21Rik	1.0673998	0.19	17.7%
1700022I11Rik	0.03110418	0.01	44.3%
1700023A16Rik	0.00850266	0.01	140.8%
1700023B02Rik	0.7878604	0.17	21.4%
1700023D19Rik	0	0.00	
1700023E05Rik	0	0.00	
1700023F06Rik	1.0975198	0.18	16.0%
1700023I07Rik	0	0.00	
1700024G10Rik	0.01265578	0.03	223.6%
1700024G13Rik	0	0.00	
1700025D03Rik	0	0.00	
1700025E21Rik	0.08389366	0.04	52.7%
1700025F22Rik	0	0.00	
1700025G04Rik	16.64606	0.36	2.2%
1700025K23Rik	7.076718	0.42	5.9%
1700026D08Rik	0.04952476	0.04	74.9%
1700026J04Rik	0	0.00	
1700026L06Rik	0.15719914	0.06	37.7%
1700027A23Rik	0	0.00	
1700027D21Rik	0.05426204	0.03	59.7%
1700027J05Rik	12.85314	0.97	7.6%
1700027N10Rik	1.47937	0.23	15.7%
1700028J19Rik	0.05815212	0.05	80.3%
1700028K03Rik	0.7143768	0.32	44.3%
1700028P14Rik	0.02152342	0.02	94.5%
1700029F09Rik	1.666298	0.20	12.1%
1700029F12Rik	0.0054322	0.01	223.6%
1700029G01Rik	0.2535394	0.03	11.5%
1700029H14Rik	0	0.00	
1700029I01Rik	0.10932338	0.06	54.6%
1700029I08Rik	0.0111053	0.02	223.6%
1700029I15Rik	0.276439	0.16	57.3%
1700029J07Rik	1.255382	0.20	15.9%
1700029J11Rik	0.01941042	0.04	223.6%
1700029P11Rik	0	0.00	
1700030B21Rik	0	0.00	
1700030E15Rik	0	0.00	
1700030F18Rik	0.00855126	0.02	223.6%
1700030J22Rik	2.13829	0.22	10.4%

1700030K09Rik	3.323538	1.49	44.7%
1700031F05Rik	0	0.00	
1700034E13Rik	0	0.00	
1700034H14Rik	2.47369	0.37	15.1%
1700034I23Rik	0.01405218	0.02	154.6%
1700034J05Rik	0	0.00	
1700034K16Rik	0.00956222	0.02	223.6%
1700034M03Rik	0.80957	0.50	61.8%
1700034O15Rik	0	0.00	
1700036D21Rik	0.003929	0.01	223.6%
1700037C18Rik	1.610922	0.20	12.5%
1700037H04Rik	42.32502	2.52	5.9%
1700039E15Rik	0	0.00	
1700040I03Rik	6.63056	1.08	16.2%
1700040L02Rik	0.07826098	0.05	60.5%
1700041B01Rik	0.05050168	0.01	25.0%
1700041B20Rik	0.6037264	0.08	13.4%
1700041C02Rik	2.716632	0.26	9.4%
1700041E20Rik	0	0.00	
1700042B14Rik	0	0.00	
1700042G07Rik	0	0.00	
1700045I19Rik	0.05574	0.02	33.2%
1700047I17Rik1	0.00273734	0.01	223.6%
1700049E17Rik	0.01555928	0.02	158.8%
1700049G17Rik	1.684096	0.20	11.8%
1700049K14Rik	0.00563442	0.01	223.6%
1700049L16Rik	0.04025624	0.05	127.4%
1700052K11Rik	2.064166	0.38	18.2%
1700052N19Rik	3.745462	0.16	4.2%
1700054N08Rik	1.773292	0.17	9.4%
1700054O13Rik	0	0.00	
1700055M20Rik	0	0.00	
1700055N04Rik	0.00490687	0.01	133.0%
1700057G04Rik	0.00685674	0.02	223.6%
1700057K13Rik	0	0.00	
1700058C13Rik	0	0.00	
1700060E18Rik	0	0.00	
1700060H10Rik	0.7644568	0.14	18.4%
1700061G19Rik	0.03368066	0.02	57.3%
1700061J05Rik	0.00459216	0.01	223.6%
1700063I17Rik	0.0271447	0.04	137.4%
1700065D16Rik	0.12931524	0.05	40.7%
1700065I17Rik	0	0.00	
1700065O13Rik	7.84198	0.26	3.4%
1700066B19Rik	0.0386092	0.03	83.3%
1700067C01Rik	0.02674292	0.02	57.5%
1700067K01Rik	0.03947428	0.03	88.3%

1700067P10Rik	0	0.00	
1700071K01Rik	0.3552984	0.09	25.7%
1700072E05Rik	0.00753014	0.02	223.6%
1700073E17Rik	0.00999354	0.01	137.1%
1700074P13Rik	0	0.00	
1700080E11Rik	0	0.00	
1700080O16Rik	0	0.00	
1700081D17Rik	0	0.00	
1700081L11Rik	17.84384	0.16	0.9%
1700084C01Rik	0.593358	0.15	25.2%
1700088E04Rik	1.1394994	0.20	17.9%
1700090G07Rik	0	0.00	
1700093K21Rik	0.04475124	0.02	45.0%
1700094C09Rik	0	0.00	
1700094D03Rik	1.509422	0.29	19.4%
1700101E01Rik	0.15407126	0.08	53.9%
1700102P08Rik	0.2444784	0.09	35.1%
1700106N22Rik	4.408006	0.34	7.7%
1700108L22Rik	0.12695746	0.06	44.9%
1700108M19Rik	0	0.00	
1700109F18Rik	1.1006314	0.22	19.9%
1700109H08Rik	4.36390922	8.06	184.6%
1700110M21Rik	0.00273176	0.01	223.6%
1700112C13Rik	0	0.00	
1700113H08Rik	0	0.00	
1700113I22Rik	2.30183	0.80	34.8%
1700113O17Rik	0	0.00	
1700120B06Rik	0.04510334	0.02	47.4%
1700120K04Rik	0.0607263	0.05	79.7%
1700123I01Rik	0.05233834	0.03	60.5%
1700123K08Rik	0.13285488	0.06	45.7%
1700123L14Rik	0	0.00	
1700123O20Rik	4.72378	0.24	5.0%
1700124K17Rik	4.20363	0.54	12.8%
1700125D06Rik	0	0.00	
1700125F08Rik	0	0.00	
1700126L10Rik	0.05917618	0.03	54.9%
1700127D06Rik	0	0.00	
1700129C05Rik	0	0.00	
1700129I04Rik	2.021242	0.57	28.0%
1700129I15Rik	0	0.00	
1810006K21Rik	0.8269932	0.37	44.3%
1810007E14Rik	0.052133	0.07	139.6%
1810007P19Rik	8.522686	0.65	7.7%
1810008A18Rik	8.989052	0.81	9.0%
1810008K16Rik	0	0.00	
1810009A15Rik	7.642894	0.66	8.6%

1810009J06Rik	0	0.00	
1810009O10Rik	6.909064	0.65	9.5%
1810010H24Rik	1.63908	0.09	5.7%
1810010M01Rik	0.13706234	0.04	27.0%
1810011O10Rik	0.2862558	0.07	25.5%
1810012P15Rik	8.220818	0.40	4.8%
1810013D10Rik	9.292	0.40	4.3%
1810013L24Rik	17.79084	0.58	3.3%
1810014F10Rik	8.841006	0.90	10.2%
1810015A11Rik	10.933118	1.23	11.3%
1810015C04Rik	27.4093	1.23	4.5%
1810019J16Rik	0.0946196	0.03	34.3%
1810020D17Rik	9.784104	0.95	9.7%
1810020G14Rik	2.912408	1.86	64.0%
1810021J13Rik	7.26497	0.77	10.6%
1810022C23Rik	0.04937958	0.02	48.6%
1810022K09Rik	6.025182	0.85	14.1%
1810023F06Rik	0.0073401	0.02	223.6%
1810024B03Rik	0.2744652	0.09	33.6%
1810026J23Rik	19.26538	1.32	6.9%
1810027O10Rik	35.3478	4.49	12.7%
1810029B16Rik	1.811364	0.12	6.8%
1810030J14Rik	0	0.00	
1810030N24Rik	11.8835	1.26	10.6%
1810030O07Rik	4.935262	0.23	4.6%
1810031K17Rik	11.49636	0.34	2.9%
1810032O08Rik	3.892648	0.42	10.7%
1810033B17Rik	0.03805494	0.04	110.4%
1810033M07Rik	0	0.00	
1810034K20Rik	14.21102	1.32	9.3%
1810035L17Rik	24.79344	3.31	13.4%
1810037C20Rik	5.318714	0.27	5.1%
1810037I17Rik	14.11216	1.17	8.3%
1810041L15Rik	2.132184	0.19	9.0%
1810043G02Rik	9.250634	0.26	2.8%
1810044A24Rik	44.19322	0.65	1.5%
1810046J19Rik	49.89846	5.39	10.8%
1810046K07Rik	0.02108434	0.04	171.3%
1810047C23Rik	16.05588	0.39	2.4%
1810048J11Rik	11.04032	0.38	3.5%
1810049H13Rik	3.331274	0.14	4.1%
1810049H19Rik	0.01586694	0.02	138.1%
1810054D07Rik	6.358472	0.96	15.2%
1810055E12Rik	19.35662	0.77	4.0%
1810055G02Rik	9.60898	0.70	7.2%
1810059G22Rik	4.170754	0.94	22.6%
1810063B05Rik	7.164702	0.31	4.3%

1810063B07Rik	1.58924	0.34	21.4%
1810064F22Rik	0.00401226	0.01	223.6%
1810065E05Rik	0	0.00	
1810073G14Rik	8.86225	0.34	3.9%
1810073H04Rik	0.00249634	0.01	223.6%
1810073N04Rik	23.33026	2.26	9.7%
1810074P20Rik	9.227656	0.20	2.2%
2010001E11Rik	0.0062553	0.01	223.6%
2010001J22Rik	0.03731584	0.02	50.6%
2010001M09Rik	0.02095106	0.02	94.7%
2010002M12Rik	0.1097964	0.04	34.8%
2010002N04Rik	0.4255792	0.13	31.7%
2010003H20Rik	0.1403256	0.02	16.9%
2010003J03Rik	12.11706	0.56	4.7%
2010003K11Rik	0.00884186	0.01	137.8%
2010003O18Rik	12.9122	0.88	6.8%
2010004A03Rik	0.2357138	0.07	29.6%
2010005H15Rik	0	0.00	
2010005J08Rik	12.7469	0.64	5.0%
2010007H12Rik	6.446368	0.31	4.8%
2010011I20Rik	5.261668	0.37	7.1%
2010012C16Rik	1.1506278	0.31	27.2%
2010012O05Rik	13.52666	0.94	6.9%
2010015L04Rik	1.090554	0.10	9.1%
2010100O12Rik	31.255	4.36	14.0%
2010106E10Rik	0.0274055	0.04	139.4%
2010106G01Rik	6.241354	0.50	7.9%
2010107E04Rik	128.37	9.43	7.3%
2010107G12Rik	0.06403728	0.04	60.5%
2010107G23Rik	7.447352	0.83	11.2%
2010107H07Rik	4.855334	0.57	11.8%
2010109I03Rik	0	0.00	
2010109K11Rik	2.00493	0.26	13.2%
2010110K16Rik	11.34106	0.52	4.6%
2010110P09Rik	0.00582618	0.01	223.6%
2010111I01Rik	2.843388	0.88	30.9%
2010200O16Rik	14.75354	0.45	3.1%
2010203O07Rik	2.158038	0.22	10.2%
2010204N08Rik	0.03026394	0.02	52.4%
2010208K18Rik	9.609952	0.52	5.4%
2010209O12Rik	12.5971	0.79	6.3%
2010300C02Rik	0.8450982	0.08	9.4%
2010301N04Rik	2.023488	0.33	16.5%
2010305A19Rik	4.01778	0.44	10.9%
2010305C02Rik	0.06475958	0.04	54.7%
2010309E21Rik	10.03154	0.47	4.7%
2010311D03Rik	23.32072	1.45	6.2%

2010315B03Rik	0	0.00	
2010315L10Rik	0.7208616	0.68	93.9%
2010316F05Rik	0.9874186	0.13	13.5%
2010317E24Rik	0.3458298	0.04	12.5%
2010321M09Rik	4.808374	0.28	5.8%
2200001I15Rik	0	0.00	
2200002D01Rik	0.364074	0.18	50.5%
2200002J24Rik	0	0.00	
2200002K05Rik	1.721994	0.21	12.0%
2210009G21Rik	6.245972	0.78	12.5%
2210010B09Rik	6.308212	0.38	6.0%
2210010C04Rik	0.00377752	0.01	223.6%
2210010C17Rik	0.4472516	0.08	18.2%
2210010L05Rik	22.92178	0.77	3.4%
2210010N04Rik	9.735392	1.07	11.0%
2210012G02Rik	2.854774	0.21	7.3%
2210016L21Rik	55.97806	2.74	4.9%
2210018M11Rik	6.775838	0.33	4.8%
2210020M01Rik	0.03290396	0.02	68.9%
2210021J22Rik	3.409254	0.26	7.6%
2210023G05Rik	0.14353486	0.11	74.0%
2210038L17Rik	7.754588	1.95	25.1%
2210404O07Rik	0.10099006	0.07	74.0%
2210407C18Rik	0.0757988	0.04	57.7%
2210408I21Rik	3.747958	0.37	9.9%
2210411K11Rik	0.3317572	0.10	30.3%
2210412D01Rik	13.52482	1.81	13.4%
2210415F13Rik	0.04690444	0.05	100.6%
2210417D09Rik	0.0431187	0.04	89.5%
2210418G03Rik	0	0.00	
2210418O10Rik	0.12438156	0.04	34.2%
2210421G13Rik	0	0.00	
2300002D11Rik	45.94574	2.23	4.8%
2300002M23Rik	0	0.00	
2300005B03Rik	0	0.00	
2300009A05Rik	0.8100034	0.16	19.1%
2310001A20Rik	21.80196	0.50	2.3%
2310001H12Rik	4.805826	0.16	3.3%
2310002B06Rik	17.27808	0.48	2.8%
2310002B14Rik	0	0.00	
2310002J15Rik	0.11204132	0.07	65.2%
2310002L09Rik	0.0068133	0.02	223.6%
2310002L13Rik	0	0.00	
2310003C23Rik	16.1298	0.39	2.4%
2310003F16Rik	32.3994	2.63	8.1%
2310003H01Rik	10.95044	1.48	13.6%
2310003L22Rik	26.51944	0.95	3.6%

2310004I24Rik	5.441872	0.76	14.0%
2310004L02Rik	14.31124	0.81	5.7%
2310004N24Rik	12.5898	1.45	11.5%
2310005E10Rik	1.808974	0.32	17.6%
2310005G13Rik	0.13459448	0.06	41.3%
2310005N01Rik	29.66938	1.83	6.2%
2310005N03Rik	8.493132	1.36	16.0%
2310005P05Rik	1.844174	0.22	11.9%
2310007A19Rik	0.1075833	0.07	65.9%
2310007B03Rik	0	0.00	
2310007D09Rik	0.0514694	0.02	43.4%
2310007F21Rik	23.57056	0.99	4.2%
2310007H09Rik	5.900794	1.11	18.9%
2310007L24Rik	0	0.00	
2310008H04Rik	0.787943	0.17	21.3%
2310008H09Rik	6.909052	1.22	17.6%
2310008M10Rik	32.85488	2.85	8.7%
2310009B15Rik	3.06447	0.38	12.6%
2310009E04Rik	3.036928	0.47	15.6%
2310010M20Rik	0.08078542	0.05	66.2%
2310010M24Rik	7.241472	0.53	7.4%
2310011G06Rik	0	0.00	
2310011J03Rik	6.278424	0.77	12.3%
2310014G06Rik	11.0526	0.21	1.9%
2310014H01Rik	0.6058396	0.20	33.5%
2310014L17Rik	0.12911264	0.03	22.2%
2310015A05Rik	1.37673	0.15	11.0%
2310015N07Rik	0.5533724	0.30	54.1%
2310016C08Rik	2.815724	0.83	29.4%
2310016C16Rik	5.403606	0.78	14.5%
2310016E02Rik	3.604672	0.68	18.9%
2310016F22Rik	0.01888244	0.02	105.7%
2310016M24Rik	42.46042	2.32	5.5%
2310021H06Rik	0	0.00	
2310021P13Rik	22.00676	0.67	3.1%
2310022A10Rik	9.17343	0.35	3.8%
2310022B05Rik	3.11959	0.29	9.3%
2310022K01Rik	0.5121114	0.27	53.0%
2310022M17Rik	18.91586	0.29	1.5%
2310026E23Rik	5.748338	0.82	14.2%
2310028H24Rik	63.758	1.80	2.8%
2310028N02Rik	3.395548	0.21	6.3%
2310028O11Rik	66.9335	1.21	1.8%
2310030G06Rik	0.16217684	0.07	42.1%
2310030N02Rik	5.27722	0.19	3.6%
2310031A18Rik	0.02469014	0.02	68.4%
2310033E01Rik	0	0.00	

2310033K02Rik	0.07710308	0.02	25.8%
2310033P09Rik	4.324358	0.17	3.9%
2310034C09Rik	0	0.00	
2310035C23Rik	18.9986	0.89	4.7%
2310035K24Rik	9.512936	0.62	6.6%
2310036O22Rik	25.3868	0.94	3.7%
2310037I24Rik	3.868326	0.24	6.1%
2310038E17Rik	0.003871724	0.01	139.3%
2310038H17Rik	5.172552	0.64	12.3%
2310039E09Rik	0.2237972	0.07	33.2%
2310039H08Rik	2.192186	0.32	14.8%
2310040A07Rik	19.78282	1.52	7.7%
2310040C09Rik	0.023144158	0.03	140.7%
2310040M23Rik	0	0.00	
2310042D19Rik	0.6201968	0.05	8.1%
2310042E16Rik	2.958246	0.42	14.1%
2310042E22Rik	0.01918162	0.04	223.6%
2310043J07Rik	0	0.00	
2310043L02Rik	0	0.00	
2310044G17Rik	12.1127	0.60	5.0%
2310044H10Rik	45.76296	1.67	3.7%
2310045A20Rik	5.030598	0.24	4.7%
2310046A06Rik	0.09273822	0.04	43.3%
2310046K01Rik	0.02086814	0.02	78.3%
2310046O06Rik	9.916842	0.64	6.5%
2310047B19Rik	4.707592	0.47	10.0%
2310047D13Rik	2.7727	0.47	16.9%
2310047M10Rik	11.379236	1.06	9.3%
2310047O13Rik	11.68186	0.95	8.1%
2310051M13Rik	0	0.00	
2310056P07Rik	16.5848	2.05	12.4%
2310057J16Rik	16.7141	1.13	6.8%
2310057J18Rik	0.01378774	0.03	223.6%
2310057M21Rik	8.394212	0.85	10.1%
2310057N15Rik	0	0.00	
2310058O09Rik	0.3126374	0.05	15.6%
2310061C15Rik	4.718968	0.38	8.0%
2310061F22Rik	0.621377	0.05	7.9%
2310061I04Rik	31.89682	0.63	2.0%
2310061J03Rik	4.098512	0.38	9.2%
2310065K24Rik	24.53486	1.60	6.5%
2310066E14Rik	18.27118	0.81	4.4%
2310067B10Rik	7.14661	0.81	11.3%
2310073E15Rik	10.837046	2.84	26.2%
2310076L09Rik	0.5848508	0.06	9.9%
2310079F23Rik	1.7665	0.08	4.8%
2310079N02Rik	10.349922	0.65	6.2%

2400001E08Rik	26.85296	2.99	11.2%
2400003C14Rik	30.38488	0.55	1.8%
2410001C21Rik	22.79594	0.92	4.0%
2410002F23Rik	19.32322	1.57	8.1%
2410002I01Rik	5.447688	0.18	3.2%
2410002M20Rik	0.11697874	0.06	52.4%
2410002O22Rik	15.51572	0.60	3.9%
2410003J06Rik	0.04656054	0.02	32.3%
2410004A20Rik	0.00388522	0.01	223.6%
2410004B18Rik	11.7338	0.71	6.1%
2410004F06Rik	0.1394642	0.03	18.8%
2410004L22Rik	6.70188	0.39	5.8%
2410005O16Rik	4.11119	0.25	6.1%
2410008K03Rik	0.4084994	0.10	25.5%
2410012H22Rik	6.579706	0.95	14.5%
2410014A08Rik	29.89028	1.67	5.6%
2410015M20Rik	21.85408	0.81	3.7%
2410015N17Rik	6.968058	0.60	8.7%
2410016O06Rik	2.723386	0.25	9.2%
2410017P07Rik	2.038724	0.22	10.9%
2410018C17Rik	2.802098	0.26	9.3%
2410018C20Rik	2.890088	0.15	5.4%
2410018G20Rik	10.121118	0.78	7.7%
2410018L13Rik	0.7055844	0.16	22.3%
2410018M08Rik	3.751584	0.30	8.0%
2410019A14Rik	6.999116	0.39	5.6%
2410022L05Rik	27.24488	2.05	7.5%
2410022M11Rik	0.8267852	0.05	6.6%
2410025L10Rik	5.320242	0.38	7.1%
2410042D21Rik	4.140822	0.27	6.5%
2410066E13Rik	20.48358	0.54	2.6%
2410075B13Rik	3.975484	0.31	7.9%
2410076I21Rik	1.666622	0.42	25.0%
2410081M15Rik	1.973688	0.39	19.8%
2410089E03Rik	7.0603	0.22	3.1%
2410091C18Rik	12.34312	0.36	2.9%
2410116G06Rik	0	0.00	
2410127E18Rik	50.5846	1.97	3.9%
2410127L17Rik	0.07807456	0.05	66.5%
2410129H14Rik	23.32582	1.22	5.2%
2410131K14Rik	16.30604	0.40	2.4%
2410137M14Rik	0.11470718	0.10	83.2%
2410141K09Rik	0	0.00	
2410146L05Rik	0	0.00	
2410166I05Rik	17.56296	0.68	3.9%
2410187C16Rik	2.531718	0.36	14.3%
2500002L14Rik	1.3556906	0.85	63.0%

2500003M10Rik	16.78492	3.96	23.6%
2510002D24Rik	4.725502	0.40	8.5%
2510003E04Rik	38.97834	1.72	4.4%
2510006C20Rik	1.2325724	0.64	51.5%
2510006D16Rik	32.93182	3.49	10.6%
2510009E07Rik	19.24796	0.46	2.4%
2510012J08Rik	4.553	0.22	4.7%
2510027J23Rik	0	0.00	
2510039O18Rik	7.21227	0.40	5.5%
2510048L02Rik	3.182468	0.58	18.3%
2510049I19Rik	29.77276	1.31	4.4%
2600001A11Rik	0.7097314	0.08	11.0%
2600005O03Rik	0.1811828	0.03	15.5%
2600009E05Rik	18.31	1.15	6.3%
2600010E01Rik	0.3006504	0.06	20.4%
2600011C06Rik	19.33538	0.37	1.9%
2600011E07Rik	3.421516	0.34	10.0%
2610001J05Rik	10.29651	0.96	9.4%
2610002D18Rik	0.10357378	0.04	42.9%
2610002J02Rik	6.317396	0.21	3.4%
2610002M06Rik	17.5362	0.75	4.3%
2610003J06Rik	13.03652	0.68	5.3%
2610008E11Rik	6.45701	0.18	2.7%
2610015P09Rik	0.406982	0.04	9.5%
2610016C23Rik	0.2157082	0.05	22.6%
2610018G03Rik	0.3829466	0.10	25.0%
2610018I03Rik	1.583138	0.18	11.4%
2610019A05Rik	6.975672	1.07	15.3%
2610019F03Rik	2.753764	0.35	12.9%
2610019P18Rik	3.614666	0.28	7.6%
2610020H08Rik	0.572209	0.07	11.9%
2610021K21Rik	0.10109988	0.05	47.1%
2610024E20Rik	25.54548	4.38	17.1%
2610024G14Rik	7.909444	0.27	3.4%
2610027C15Rik	1.590822	0.41	25.6%
2610027L16Rik	3.854502	0.57	14.8%
2610028A01Rik	1.674882	0.37	22.0%
2610028H24Rik	0.02814836	0.01	44.7%
2610029G23Rik	4.178082	0.39	9.3%
2610029I01Rik	7.311352	0.18	2.5%
2610030H06Rik	18.25238	0.95	5.2%
2610034B18Rik	5.400224	0.47	8.6%
2610034M16Rik	0.01484894	0.03	187.6%
2610036D13Rik	5.974488	0.26	4.4%
2610036L11Rik	1.633358	0.52	31.6%
2610039C10Rik	1.455744	0.23	15.9%
2610042L04Rik	0.11147006	0.02	17.3%

2610044O15Rik	10.354252	1.20	11.6%
2610101N10Rik	17.77162	1.47	8.3%
2610109H07Rik	1.000285	0.17	17.0%
2610110G12Rik	32.81068	3.59	10.9%
2610200G18Rik	10.590024	1.78	16.8%
2610203E10Rik	1.29776	0.18	13.9%
2610204K14Rik	2.104916	0.34	16.2%
2610204M08Rik	0.5989204	0.12	20.8%
2610205E22Rik	13.55534	1.59	11.7%
2610206B13Rik	5.711834	0.39	6.8%
2610207I05Rik	6.886458	0.76	11.0%
2610208M17Rik	0	0.00	
2610209A20Rik	1.540222	0.09	5.7%
2610209M04Rik	15.7995	1.12	7.1%
2610301B20Rik	11.19688	0.43	3.8%
2610301F02Rik	0.2763046	0.08	28.1%
2610301G19Rik	20.30156	0.36	1.8%
2610304G08Rik	8.553648	0.33	3.9%
2610305D13Rik	0.540975	0.16	30.2%
2610318N02Rik	0.13209584	0.05	40.2%
2610507B11Rik	40.93136	2.12	5.2%
2610510H03Rik	10.392906	0.54	5.2%
2610524H06Rik	13.8169	1.68	12.1%
2610528E23Rik	3.35778	0.47	13.9%
2610528J11Rik	0.00541438	0.01	223.6%
2610528K11Rik	26.17946	1.41	5.4%
2610529C04Rik	5.29727	0.61	11.5%
2700007P21Rik	11.18796	0.74	6.6%
2700019D07Rik	2.780744	0.26	9.2%
2700029M09Rik	17.43224	2.09	12.0%
2700038C09Rik	11.50934	1.59	13.9%
2700038N03Rik	14.22082	1.63	11.4%
2700045P11Rik	0.2901038	0.14	49.6%
2700049A03Rik	1.39772	0.12	8.5%
2700049P18Rik	0.4310504	0.03	7.2%
2700050L05Rik	6.1	0.28	4.6%
2700059D21Rik	22.48452	1.74	7.8%
2700060E02Rik	7.945092	0.58	7.4%
2700062C07Rik	5.023156	0.74	14.8%
2700078E11Rik	13.44272	0.74	5.5%
2700078K21Rik	7.830544	0.76	9.8%
2700081O15Rik	8.479726	0.45	5.3%
2700094F01Rik	7.745226	0.50	6.5%
2700094K13Rik	19.00616	1.72	9.1%
2700097O09Rik	2.303772	0.23	10.2%
2810002D19Rik	1.2185354	0.25	20.9%
2810002G02Rik	8.07181	0.56	6.9%

2810002I04Rik	0.3417378	0.04	10.7%
2810002N01Rik	14.71984	1.91	12.9%
2810002O09Rik	5.313556	0.26	5.0%
2810003C17Rik	0.7721326	0.12	15.2%
2810004N23Rik	6.189032	0.32	5.2%
2810006K23Rik	2.98921	0.29	9.6%
2810007J24Rik	0	0.00	
2810008M24Rik	15.25514	0.69	4.5%
2810012G03Rik	13.71508	0.55	4.0%
2810021B07Rik	2.533382	0.12	4.6%
2810021J22Rik	2.750398	0.13	4.8%
2810022L02Rik	23.24186	1.97	8.5%
2810025M15Rik	5.719792	1.05	18.4%
2810026P18Rik	7.718404	0.89	11.5%
2810030E01Rik	2.112178	0.14	6.8%
2810032E02Rik	35.64508	2.08	5.8%
2810046L04Rik	3.889072	0.42	10.9%
2810047C21Rik	0.00480488	0.01	223.6%
2810048G17Rik	0.0083837	0.01	143.9%
2810051F02Rik	2.741854	0.53	19.3%
2810055F11Rik	0.9505368	0.10	10.5%
2810403A07Rik	13.43922	0.62	4.6%
2810405J04Rik	15.55364	1.57	10.1%
2810405K02Rik	16.8095	1.02	6.1%
2810406K13Rik	0.03799318	0.02	52.1%
2810407C02Rik	78.41994	3.31	4.2%
2810408A11Rik	0.1856112	0.01	7.1%
2810408M09Rik	2.978732	0.16	5.4%
2810409K11Rik	0.6269006	0.05	7.3%
2810410C14Rik	1.19709	0.13	10.8%
2810410M20Rik	23.00128	2.16	9.4%
2810416G20Rik	0.021828	0.03	118.9%
2810417H13Rik	0.6717414	0.11	16.8%
2810422J05Rik	11.9176	0.67	5.6%
2810422O20Rik	1.696734	0.17	10.1%
2810426N06Rik	12.4441	0.15	1.2%
2810428I15Rik	20.61208	2.30	11.1%
2810432D09Rik	6.847718	0.56	8.1%
2810432L12Rik	42.20322	2.34	5.6%
2810433K01Rik	0.09829742	0.02	25.2%
2810439F02Rik	6.856918	0.37	5.4%
2810441K11Rik	7.25482	0.63	8.6%
2810451A06Rik	0.09528506	0.03	36.3%
2810452K22Rik	13.8671	1.35	9.7%
2810453I06Rik	8.46529	0.63	7.4%
2810457I06Rik	7.919898	0.77	9.8%
2810459M11Rik	4.669004	0.38	8.2%

2810474O19Rik	2.321794	0.28	11.9%
2810485I05Rik	3.723238	0.38	10.3%
2810487A22Rik	0.10320256	0.04	36.9%
2900002H16Rik	6.780316	0.51	7.6%
2900006F19Rik	0.6887922	0.26	37.6%
2900006K08Rik	0.493104	0.14	28.2%
2900009I07Rik	10.024072	1.01	10.0%
2900010J23Rik	62.59508	2.29	3.7%
2900010M23Rik	23.59312	2.04	8.6%
2900011O08Rik	78.87338	1.35	1.7%
2900024C23Rik	33.75976	2.01	5.9%
2900024O10Rik	36.3979	2.90	8.0%
2900026A02Rik	0.8897346	0.09	10.6%
2900042B11Rik	7.022408	1.18	16.9%
2900046G09Rik	12.37066	0.76	6.1%
2900057K09Rik	2.511796	0.23	9.1%
2900064A13Rik	46.30212	1.48	3.2%
2900073G15Rik	13.26884	1.15	8.6%
2900075B16Rik	27.87902	2.14	7.7%
2900083I11Rik	6.653036	0.16	2.5%
2900092C05Rik	0	0.00	
2900092E17Rik	23.67724	2.18	9.2%
2b4	0.005377904	0.00	62.8%
3000004C01Rik	0.4184136	0.10	24.8%
3010026O09Rik	10.99906	0.80	7.3%
3100002J23Rik	0.12665888	0.11	90.4%
3100004P22Rik	18.52936	1.25	6.8%
3110001A13Rik	3.914844	0.41	10.4%
3110001D03Rik	14.06376	0.60	4.3%
3110001I20Rik	10.12092	0.56	5.5%
3110001I22Rik	0.9444018	0.16	17.1%
3110001K24Rik	1.672424	0.20	12.2%
3110002H16Rik	11.4698	0.31	2.7%
3110003A17Rik	6.052354	0.69	11.4%
3110003A22Rik	6.213328	0.33	5.4%
3110004L20Rik	30.1232	2.54	8.4%
3110005G23Rik	15.43662	0.33	2.2%
3110006E14Rik	139.3828	18.43	13.2%
3110007F17Rik	0.04219	0.03	72.7%
3110009E18Rik	1.2558396	0.38	30.5%
3110031B13Rik	3.37942	0.32	9.4%
3110032G18Rik	0.29182334	0.14	48.1%
3110035E14Rik	2.940282	0.36	12.4%
3110037I16Rik	2.67583	0.39	14.7%
3110040N11Rik	9.072414	0.58	6.4%
3110043J09Rik	0.1569848	0.04	25.0%
3110043O21Rik	23.76848	1.12	4.7%

3110045G13Rik	0.884246	0.16	18.2%
3110047P20Rik	0.0423266	0.01	24.0%
3110048E14Rik	10.544086	0.51	4.8%
3110049J23Rik	0.1945366	0.04	19.0%
3110050N22Rik	32.90608	1.31	4.0%
3110052M02Rik	8.401652	0.43	5.1%
3110056O03Rik	9.297998	1.73	18.6%
3110057O12Rik	3.486368	0.44	12.5%
3110062M04Rik	0.7594686	0.11	14.1%
3110070M22Rik	0.2198568	0.05	21.9%
3110079O15Rik	0.0296235	0.04	147.0%
3110082D06Rik	0.62677	0.09	13.6%
3110082I17Rik	1.53767	0.28	18.2%
3200001K10Rik	2.210956	0.44	20.0%
3200002M19Rik	7.256864	4.09	56.3%
3222402P14Rik	12.58908	1.03	8.2%
3230401D17Rik	0.310744	0.08	24.5%
3230401M21Rik	1.726408	0.11	6.2%
3300001A09Rik	0.6374002	0.10	15.5%
3300001G02Rik	7.993382	0.80	10.0%
3300001P08Rik	37.3179	1.48	4.0%
3300002A11Rik	0	0.00	
3300002I08Rik	0.6258234	0.28	44.7%
3321401G04Rik	78.96576	3.62	4.6%
3322402L07Rik	0.00385152	0.01	223.6%
3632451O06Rik	6.534324	0.84	12.8%
3830402I07Rik	3.037862	0.27	8.9%
3830403N18Rik	0.00818668	0.02	223.6%
3830406C13Rik	9.194658	0.55	6.0%
3830408D24Rik	0.03909626	0.03	73.3%
3830417A13Rik	0	0.00	
3930401K13Rik	45.99038	1.50	3.3%
4121402D02Rik	42.1814	1.70	4.0%
4430402I18Rik	1.406535	0.84	59.8%
4432412L15Rik	1.594898	0.31	19.7%
4432416J03Rik	0.05248056	0.02	45.7%
4631405J19Rik	0.00259732	0.01	223.6%
4631416L12Rik	2.799848	0.14	4.9%
4631422C13Rik	0.5925212	0.04	6.5%
4631422O05Rik	0.4604504	0.11	23.3%
4631424J17Rik	5.05421	0.11	2.3%
4631426E05Rik	0	0.00	
4631426J05Rik	7.995332	0.57	7.1%
4631427C17Rik	28.25544	1.30	4.6%
4632404H22Rik	1.185719	0.45	38.1%
4632408A20Rik	0.03178676	0.02	48.8%
4632411B12Rik	20.43498	0.90	4.4%

4632411J06Rik	0.201857	0.02	11.6%
4632412N22Rik	0.03978188	0.01	32.6%
4632415K11Rik	3.165162	0.24	7.6%
4632417K18Rik	1.343512	0.09	6.9%
4632417N05Rik	0.5381512	0.04	6.5%
4632419I22Rik	2.106962	0.24	11.5%
4632419K20Rik	7.318868	0.37	5.0%
4632425D07Rik	5.51997	0.52	9.4%
4632428N05Rik	0.4283556	0.04	10.3%
4632433K11Rik	0.6981306	0.12	17.6%
4632434I11Rik	0.8436022	0.06	6.7%
4732415M23Rik	0.5316276	0.11	20.6%
4732418C07Rik	8.152494	0.81	10.0%
4732429D16Rik	0	0.00	
4732435N03Rik	2.174784	0.12	5.4%
4732454E20Rik	0	0.00	
4732456N10Rik	0.00217902	0.00	223.6%
4732466D17Rik	0.0883998	0.03	33.6%
4732471D19Rik	5.428724	0.18	3.3%
4732473B16Rik	0.07018796	0.04	56.2%
4732474O15Rik	0.01234678	0.01	94.2%
4732479N06Rik	4.156364	0.32	7.7%
4732495G21Rik	0.00281148	0.01	223.6%
4732496O08Rik	4.540102	0.20	4.5%
4733401H18Rik	11.63162	1.01	8.6%
4733401H21Rik	0	0.00	
4831416G18Rik	0.03019856	0.02	51.0%
4831426I19Rik	0.04773958	0.02	48.6%
4832428D23Rik	0.0032966	0.01	223.6%
4833401D15Rik	0.0839098	0.19	223.6%
4833409A17Rik	0.2345262	0.05	21.1%
4833413O15Rik	0	0.00	
4833418A01Rik	3.282708	0.17	5.2%
4833420G17Rik	1.928148	0.21	10.9%
4833422F24Rik	0.00256972	0.01	223.6%
4833423E24Rik	0.0601359	0.04	65.6%
4833424O15Rik	11.136962	1.26	11.3%
4833426J09Rik	3.694658	0.21	5.7%
4833427G06Rik	0.03991308	0.04	100.3%
4833430A08Rik	0.0061492	0.01	137.3%
4833436C18Rik	0.10722258	0.06	57.1%
4833439L19Rik	30.3829	1.20	3.9%
4833442J19Rik	1.71204	0.38	21.9%
4833446K15Rik	2.13385	0.14	6.7%
4921501E09Rik	0.00232644	0.01	223.6%
4921504E06Rik	0	0.00	
4921505C17Rik	10.674314	1.25	11.7%

4921506J03Rik	90.15358	3.40	3.8%
4921506M07Rik	0.00366702	0.01	223.6%
4921507P07Rik	0.7185276	0.08	10.7%
4921509B22Rik	0.00312126	0.01	223.6%
4921509C19Rik	0	0.00	
4921509E05Rik	0.5677716	0.11	18.9%
4921509E07Rik	0.08756128	0.03	33.4%
4921510H08Rik	0	0.00	
4921510J17Rik	0.01478804	0.01	92.3%
4921511C04Rik	0.02104706	0.02	116.3%
4921511C20Rik	0	0.00	
4921511H03Rik	0	0.00	
4921511H13Rik	0.06645078	0.01	20.5%
4921513D11Rik	0.2218846	0.05	22.2%
4921513D23Rik	8.693376	1.19	13.7%
4921515J06Rik	0.03337936	0.02	61.8%
4921517B04Rik	4.501456	0.73	16.2%
4921517D21Rik	0	0.00	
4921517D22Rik	0.00471028	0.01	137.0%
4921517L17Rik	1.447512	0.09	6.0%
4921517N04Rik	4.431944	0.55	12.4%
4921520G13Rik	0.1228554	0.06	46.4%
4921521F21Rik	0.2755072	0.07	25.7%
4921523A10Rik	0.09506318	0.03	28.4%
4921524J06Rik	0.05540302	0.03	49.5%
4921524J17Rik	4.017418	0.24	6.0%
4921524L21Rik	0	0.00	
4921525H12Rik	0.00926286	0.01	71.7%
4921526F01Rik	0.17104152	0.05	27.6%
4921528H16Rik	0.438134	0.13	29.8%
4921528I01Rik	0.01546794	0.01	84.0%
4921528O07Rik	0	0.00	
4921530D09Rik	0.2572178	0.08	29.4%
4921530G04Rik	0.00293236	0.01	223.6%
4921530L21Rik	0.00539258	0.01	223.6%
4921531P07Rik	0.01600704	0.02	98.7%
4921533L14Rik	9.532668	0.48	5.1%
4921536K21Rik	0.1136836	0.04	33.2%
4921537P18Rik	0.00401608	0.01	223.6%
4921539E11Rik	0.00305196	0.01	223.6%
4922501C03Rik	1.0058602	0.17	16.5%
4922501K12Rik	0.01103642	0.01	94.1%
4922502D21Rik	0	0.00	
4922503N01Rik	5.27966	0.44	8.3%
4922505E12Rik	0.00207642	0.00	223.6%
4922505G16Rik	0.1458878	0.03	18.0%
4930401A09Rik	0	0.00	

4930401F20Rik	0	0.00	
4930402E16Rik	7.042696	0.31	4.3%
4930402F06Rik	0.006804	0.02	223.6%
4930402H24Rik	14.242	0.76	5.4%
4930403C10Rik	0.02688208	0.01	36.7%
4930403L05Rik	0	0.00	
4930403N07Rik	0.01703134	0.02	140.5%
4930404A10Rik	0	0.00	
4930404H21Rik	0.01265906	0.03	223.6%
4930404N11Rik	8.155316	0.77	9.4%
4930408G06Rik	0.00597364	0.01	223.6%
4930408O21Rik	2.868834	0.95	32.9%
4930412F15Rik	6.337052	0.45	7.1%
4930415O20Rik	0.05195338	0.04	71.0%
4930417G10Rik	0.2378814	0.09	38.8%
4930417M19Rik	0.2582054	0.08	29.4%
4930418G15Rik	0.01784754	0.03	164.9%
4930420K17Rik	1.485134	0.33	22.4%
4930422G04Rik	0.4318164	0.04	10.4%
4930424G05Rik	0.1646242	0.04	25.5%
4930425N13Rik	0.6294752	0.13	20.2%
4930427A07Rik	0.7521004	0.11	14.6%
4930428D18Rik	0	0.00	
4930429M06Rik	0.3688002	0.12	31.7%
4930430A15Rik	0	0.00	
4930430D24Rik	0.00858516	0.01	137.3%
4930430E16Rik	0.5164522	0.16	31.5%
4930430F08Rik	6.176034	0.56	9.1%
4930431J08Rik	9.509506	1.38	14.5%
4930431L04Rik	3.272136	0.46	14.2%
4930432H15Rik	0	0.00	
4930432K09Rik	0	0.00	
4930432K21Rik	1.154656	0.11	9.3%
4930432O21Rik	4.895134	0.46	9.5%
4930433I11Rik	0	0.00	
4930434E21Rik	0.00650897	0.01	91.8%
4930435E12Rik	0	0.00	
4930443G12Rik	0.00877568	0.02	223.6%
4930444A02Rik	9.409594	0.45	4.8%
4930449A18Rik	0	0.00	
4930449I24Rik	0	0.00	
4930451C15Rik	0.00279814	0.01	223.6%
4930451I11Rik	0	0.00	
4930452B06Rik	1.2076152	0.21	17.6%
4930453N24Rik	6.042366	0.54	8.9%
4930455C21Rik	7.965806	1.18	14.8%
4930455F23Rik	4.992144	0.48	9.6%

4930458L03Rik	0	0.00	
4930465K10Rik	0.0330356	0.05	163.2%
4930471G03Rik	0	0.00	
4930471M23Rik	8.631766	0.42	4.8%
4930473A06Rik	0.188505	0.05	25.2%
4930474M22Rik	0.0079216	0.02	223.6%
4930474N05Rik	0	0.00	
4930479M11Rik	0	0.00	
4930480E11Rik	0	0.00	
4930481F22Rik	1.51126736	0.99	65.8%
4930485B16Rik	0.2840282	0.08	26.7%
4930486G11Rik	0.3057456	0.06	19.6%
4930486L24Rik	0.0060861	0.01	223.6%
4930488E11Rik	1.4318614	0.51	35.5%
4930500O05Rik	11.20268	0.83	7.4%
4930500O09Rik	0.04727574	0.04	75.4%
4930502E18Rik	0.01224048	0.02	141.1%
4930503B20Rik	0.01171828	0.02	141.1%
4930503E14Rik	0.0428928	0.04	104.6%
4930503L19Rik	1.908668	0.33	17.1%
4930504E06Rik	4.420262	0.60	13.6%
4930504H06Rik	0.05966314	0.04	60.0%
4930504O13Rik	0	0.00	
4930505A04Rik	0.16988738	0.09	54.0%
4930505D03Rik	13.8518	0.89	6.4%
4930506M07Rik	22.36414	4.24	19.0%
4930507C10Rik	0	0.00	
4930507D05Rik	0.6631882	0.05	8.0%
4930511H01Rik	0.00425696	0.01	223.6%
4930511H11Rik	0.1106658	0.04	34.3%
4930511I11Rik	0.01037538	0.01	138.0%
4930511J11Rik	2.11528	0.27	12.6%
4930511M11Rik	0	0.00	
4930512M02Rik	0	0.00	
4930515G01Rik	0.0412319	0.03	71.8%
4930517K23Rik	0	0.00	
4930518C23Rik	0	0.00	
4930519B02Rik	0.00460594	0.01	223.6%
4930519F16Rik	0.03157828	0.02	68.0%
4930519G04Rik	0.0031952	0.01	223.6%
4930519N13Rik	0.5189226	0.07	13.5%
4930520O04Rik	0.2301762	0.09	37.5%
4930521A18Rik	0.00692404	0.02	223.6%
4930522H14Rik	0	0.00	
4930523C11Rik	0	0.00	
4930524B15Rik	0.008677	0.01	138.1%
4930524E20Rik	0	0.00	

4930526D03Rik	0.0566923	0.06	97.4%
4930526H21Rik	0.21892628	0.09	42.4%
4930527E24Rik	0	0.00	
4930528F23Rik	0.0306467	0.03	103.8%
4930528G09Rik	0.0153825	0.01	97.3%
4930529M08Rik	0.7859306	0.22	28.3%
4930534B04Rik	1.1603862	0.49	42.3%
4930535B03Rik	12.73998	0.44	3.5%
4930535E21Rik	0.00368824	0.00	109.0%
4930538K18Rik	0.4954046	0.11	21.4%
4930539E08Rik	0.0800137	0.03	36.8%
4930542N07Rik	0	0.00	
4930544G11Rik	0	0.00	
4930544G21Rik	10.455382	0.97	9.3%
4930544O15Rik	0.2488466	0.07	28.7%
4930546H06Rik	1.302032	0.14	10.5%
4930547C10Rik	0	0.00	
4930547N16Rik	0.2517392	0.07	26.9%
4930548H24Rik	0	0.00	
4930549C01Rik	0	0.00	
4930550C14Rik	0.03034868	0.03	100.9%
4930550L24Rik	0.01118998	0.02	144.8%
4930553D19Rik	0.10893776	0.08	72.9%
4930555I21Rik	0.04362222	0.04	81.1%
4930558O21Rik	0.01888316	0.03	148.0%
4930560E09Rik	0.03450284	0.02	69.2%
4930562D19Rik	1.70379	0.19	11.0%
4930562F07Rik	0.00275176	0.01	223.6%
4930563D23Rik	0	0.00	
4930563E19Rik	0.0110458	0.01	95.0%
4930563M21Rik	0.00678376	0.02	223.6%
4930563P21Rik	0.01485548	0.02	141.3%
4930564B18Rik	0	0.00	
4930566A11Rik	9.9751	0.91	9.2%
4930567H17Rik	0	0.00	
4930570C03Rik	31.85818	1.33	4.2%
4930571C24Rik	0.10349394	0.11	105.5%
4930572D21Rik	0	0.00	
4930572J05Rik	18.47724	1.33	7.2%
4930572O03Rik	0	0.00	
4930573I19Rik	4.9687	0.14	2.7%
4930578C19Rik	0	0.00	
4930578G10Rik	0.05515294	0.03	49.5%
4930578I06Rik	0	0.00	
4930578N16Rik	0.3880006	0.10	25.3%
4930579C12Rik	0.00542346	0.01	145.6%
4930579C15Rik	0.03071548	0.03	107.9%

4930579E17Rik	1.269964	0.11	8.9%
4930579G22Rik	0.20395616	0.12	57.5%
4930579G24Rik	0.7910576	0.08	9.7%
4930579J09Rik	0.14324188	0.05	33.8%
4930583H14Rik	1.038506	0.48	46.0%
4930588N13Rik	0.16172896	0.08	51.2%
4930589M24Rik	6.331036	0.26	4.1%
4930590J08Rik	0.04327564	0.01	34.6%
4930595M18Rik	0.0037273	0.01	223.6%
4930596D02Rik	0	0.00	
4931403E03Rik	0.3348544	0.03	8.3%
4931406B18Rik	0	0.00	
4931406C07Rik	4.971348	2.23	44.9%
4931406H21Rik	0.00301308	0.00	137.3%
4931406I20Rik	32.16608	1.90	5.9%
4931406P16Rik	9.945804	0.32	3.2%
4931407G18Rik	0	0.00	
4931408A02Rik	0.1067853	0.04	41.4%
4931408C20Rik	0	0.00	
4931409K22Rik	0	0.00	
4931414P19Rik	1.017215	0.19	19.1%
4931417E11Rik	0.02684204	0.03	102.4%
4931417G12Rik	0.00391688	0.01	223.6%
4931423N10Rik	0.03102212	0.01	40.4%
4931428F04Rik	19.34726	3.46	17.9%
4931428L18Rik	0	0.00	
4931429I11Rik	0.12700752	0.04	28.7%
4931432M23Rik	0.00406084	0.01	223.6%
4931433A01Rik	3.766846	0.50	13.2%
4931440B09Rik	0.03116418	0.02	73.8%
4931440F15Rik	0.238085	0.03	12.3%
4931440L10Rik	0.0123694	0.02	142.7%
4932409I22Rik	1.243354	0.05	4.1%
4932411E22Rik	0.1772622	0.03	16.0%
4932411G14Rik	0.009784852	0.01	122.9%
4932411N23Rik	0.01771748	0.01	56.6%
4932412H11Rik	0.03713022	0.01	14.9%
4932413O14Rik	0.2367212	0.05	20.6%
4932414N04Rik	0.00745982	0.01	96.4%
4932415G16Rik	0	0.00	
4932415M13Rik	0	0.00	
4932416N17Rik	6.17641	0.49	8.0%
4932417H02Rik	10.63856	0.28	2.7%
4932417I16Rik	6.743816	0.22	3.3%
4932418E24Rik	0.003469298	0.00	142.0%
4932425I24Rik	0.12344816	0.05	37.3%
4932429P05Rik	0	0.00	

4932431H17Rik	0.6987756	0.13	19.2%
4932432K03Rik	9.022852	0.64	7.1%
4932438A13Rik	11.4959	0.46	4.0%
4932438H23Rik	0.02919768	0.01	44.2%
4932441B19Rik	0	0.00	
4932441K18Rik	6.06243	0.91	15.0%
4932442K08Rik	18.74594	0.77	4.1%
4932442L08Rik	0.02693836	0.02	66.0%
4932701A20Rik	0	0.00	
4932702K14Rik	0.0238676	0.01	32.4%
4933400A11Rik	0	0.00	
4933400C05Rik	0.2681042	0.10	38.5%
4933400E14Rik	0.0093398	0.01	96.7%
4933401B01Rik	0.04387264	0.05	107.2%
4933401F05Rik	0.00755658	0.02	223.6%
4933402E13Rik	0.0228144	0.02	105.5%
4933402J07Rik	0	0.00	
4933402N03Rik	0	0.00	
4933402N22Rik	0	0.00	
4933402P03Rik	0.01146342	0.01	93.3%
4933403F05Rik	4.920004	0.34	6.8%
4933403G14Rik	1.0590416	0.07	6.2%
4933403O08Rik	0.2274914	0.09	41.5%
4933404M02Rik	0.08379428	0.03	30.9%
4933404M19Rik	0.33812	0.06	18.7%
4933405L10Rik	0.1557239	0.04	28.4%
4933405O20Rik	0	0.00	
4933406E20Rik	18.62592	1.03	5.5%
4933406M09Rik	0	0.00	
4933407C03Rik	76.00356	2.41	3.2%
4933407H18Rik	1.87381	0.23	12.0%
4933407I18Rik	0	0.00	
4933407N01Rik	15.4443	0.93	6.0%
4933407P14Rik	0.00270932	0.01	223.6%
4933408B17Rik	0.08052108	0.05	66.5%
4933409G03Rik	0	0.00	
4933409K07Rik	0	0.00	
4933411G11Rik	0	0.00	
4933411K16Rik	0.01754696	0.03	172.1%
4933411K20Rik	9.37112	0.45	4.8%
4933412E14Rik	0.2380188	0.05	20.6%
4933413G19Rik	0.01425162	0.02	137.9%
4933413N12Rik	0.03740348	0.02	63.6%
4933415F23Rik	0.00376624	0.01	223.6%
4933416C03Rik	0	0.00	
4933417A18Rik	0	0.00	
4933417O08Rik	52.15176	4.46	8.5%

4933421B21Rik	0.3856294	0.09	23.4%
4933421E11Rik	7.635854	0.19	2.5%
4933421I07Rik	0	0.00	
4933422H20Rik	0	0.00	
4933424A20Rik	1.978928	0.63	31.6%
4933424B01Rik	3.77154	0.19	5.0%
4933424C13Rik	0.12039308	0.04	36.5%
4933425B16Rik	0.05005998	0.04	72.5%
4933425K02Rik	0.03195952	0.01	27.8%
4933425L03Rik	9.191384	0.46	5.1%
4933425L06Rik	0.0071209	0.01	138.6%
4933425M15Rik	0	0.00	
4933425O20Rik	0.08991442	0.07	82.8%
4933426I21Rik	1.156104	0.13	11.1%
4933426K21Rik	25.18204	3.34	13.2%
4933426M11Rik	5.919308	0.39	6.6%
4933427D06Rik	0.00852566	0.01	93.5%
4933427D14Rik	3.127044	0.28	8.9%
4933427G17Rik	0.01437562	0.02	155.9%
4933428G09Rik	1.754552	0.30	17.2%
4933428G20Rik	9.241744	0.20	2.1%
4933429F08Rik	0.11292506	0.03	26.7%
4933430H15Rik	0.09700486	0.03	26.4%
4933430I17Rik	0.1471064	0.02	12.4%
4933432B09Rik	0.7751998	0.16	20.2%
4933433C11Rik	0.02804314	0.03	107.4%
4933433K01Rik	0.6282414	0.19	30.1%
4933433P14Rik	6.510596	0.55	8.5%
4933434E20Rik	15.5588	1.04	6.7%
4933434I06Rik	0.148803	0.05	33.4%
4933434I20Rik	0.01266624	0.03	223.6%
4933436I01Rik	0	0.00	
4933437F05Rik	0.512239	0.15	28.6%
4933437K13Rik	4.066656	0.47	11.6%
4933439C20Rik	187.2866	21.22	11.3%
4933439F11Rik	0.1529081	0.06	42.5%
4933439F18Rik	13.00752	0.55	4.2%
4933440H19Rik	0.4717792	0.12	25.1%
4F2_lc	0.259769	0.04	16.4%
5031408O05Rik	0.00206512	0.00	223.6%
5031410I06Rik	0.10404244	0.03	28.4%
5031414D18Rik	0.05258492	0.02	29.5%
5031439G07Rik	20.38874	0.87	4.3%
5033411D12Rik	0.6102254	0.36	59.5%
5033413D22Rik	0.05234174	0.01	22.1%
5033414D02Rik	7.614826	0.90	11.8%
5033414K04Rik	22.66764	2.09	9.2%

5033428A16Rik	8.968984	0.79	8.8%
5133400G04Rik	1.224182	0.22	18.2%
5133401N09Rik	7.60798	0.31	4.1%
5230400G24Rik	95.48286	5.32	5.6%
5330401P04Rik	0.3155614	0.08	25.9%
5330417C22Rik	2.085428	0.25	12.0%
5330421F07Rik	0.4424624	0.06	14.3%
5330429B09Rik	2.00157	0.53	26.7%
5330431N19Rik	27.32726	2.50	9.1%
5330437I02Rik	0.07664282	0.03	40.0%
5330439B14Rik	0.00210314	0.00	177.5%
5330439C02Rik	0.02850014	0.03	94.5%
53BP1	0.5669048	0.29	50.9%
5430401F13Rik	0	0.00	
5430402E10Rik	0	0.00	
5430405G05Rik	3.51372	0.50	14.4%
5430407P10Rik	0.4314702	0.07	16.4%
5430411K18Rik	5.543922	0.38	6.8%
5430413K10Rik	0	0.00	
5430416O09Rik	0	0.00	
5430419D17Rik	0	0.00	
5430420C16Rik	0.01130776	0.03	223.6%
5430421N21Rik	0	0.00	
5430427O19Rik	0.03271764	0.03	78.6%
5430432M24Rik	1.747132	0.06	3.7%
5430432N15Rik	0.2662214	0.05	18.0%
5430433E21Rik	0.01831358	0.01	66.9%
5430435G22Rik	0.02581696	0.02	65.5%
5430437P03Rik	24.62556	1.12	4.6%
5530400B04Rik	0.00430024	0.01	137.9%
5530400C23Rik	0	0.00	
5630401D24Rik	2.1331	0.24	11.1%
5630401J11Rik	0.8496848	0.13	14.9%
5730403B10Rik	57.57106	0.64	1.1%
5730403M16Rik	3.175	0.25	7.7%
5730407K14Rik	8.110286	0.46	5.7%
5730409E04Rik	17.47364	0.49	2.8%
5730409E15Rik	0.04663564	0.01	26.9%
5730409G15Rik	0.8991408	0.20	22.4%
5730410E15Rik	33.2247	2.28	6.9%
5730410I19Rik	10.083652	0.80	8.0%
5730419I09Rik	0.5401416	0.17	31.8%
5730427N09Rik	2.888678	0.16	5.5%
5730437N04Rik	40.74642	3.22	7.9%
5730442P18Rik	0.21493914	0.14	66.8%
5730446C15Rik	36.13834	1.06	2.9%
5730449L18Rik	3.9042	0.55	14.2%

5730453I16Rik	22.82416	0.94	4.1%
5730455O13Rik	7.586228	0.97	12.8%
5730455P16Rik	31.63042	2.04	6.5%
5730469M10Rik	20.98668	1.51	7.2%
5730470L24Rik	10.66392	0.41	3.9%
5730472N09Rik	23.33428	0.67	2.9%
5730494M16Rik	20.30702	2.30	11.3%
5730494N06Rik	7.53235	0.47	6.2%
5730507A09Rik	6.223468	0.16	2.6%
5730507C01Rik	0.00948162	0.02	223.6%
5730508B09Rik	0.3657844	0.21	58.5%
5730509K17Rik	3.363684	0.46	13.6%
5730528L13Rik	6.157604	0.56	9.1%
5730536A07Rik	7.533564	0.45	5.9%
5730557B15Rik	0.2812794	0.05	19.1%
5730559C18Rik	0.2842334	0.05	16.8%
5730577I03Rik	1.228554	0.15	12.5%
5730590G19Rik	0.1380982	0.02	16.6%
5730593F17Rik	0.6016426	0.16	26.0%
5730596K20Rik	5.541086	0.47	8.5%
5730601F06Rik	7.323388	0.70	9.5%
5830403L16Rik	0.1231856	0.02	12.8%
5830404H04Rik	7.212844	0.33	4.6%
5830405F06Rik	0	0.00	
5830405N20Rik	0.00362072	0.01	223.6%
5830406J20Rik	0.02286652	0.02	93.1%
5830411E10Rik	0.15243472	0.04	28.8%
5830411N06Rik	0	0.00	
5830415F09Rik	1.1298754	0.21	19.0%
5830415L20Rik	7.026236	0.45	6.4%
5830416A07Rik	4.854534	0.29	5.9%
5830417C01Rik	6.071134	0.22	3.6%
5830417I10Rik	1.495846	0.21	14.3%
5830418K08Rik	4.739304	1.59	33.6%
5830427D03Rik	0.02818092	0.02	71.6%
5830433M19Rik	2.49379	0.18	7.4%
5830434P21Rik	9.782226	2.24	22.9%
5830443L24Rik	0.03891704	0.01	23.6%
5830445C04Rik	0.2852446	0.04	15.7%
5830457O10Rik	13.08156	0.75	5.7%
5830467P10Rik	0.09860804	0.05	51.6%
5830472H07Rik	0.11826384	0.08	66.7%
5830472M02Rik	11.7694	0.32	2.7%
5830482F20Rik	0.07696776	0.04	57.3%
5830483C08Rik	0.4222656	0.04	9.0%
5830484A20Rik	0	0.00	
5930403L14Rik	0.2008874	0.05	23.7%

5930416I19Rik	5.297188	0.41	7.8%
5930434B04Rik	30.7399	1.34	4.4%
6030405A18Rik	2.74822	0.44	16.2%
6030408B16Rik	0.07319756	0.05	62.2%
6030408C04Rik	1.503068	0.15	9.7%
6030419C18Rik	20.9265	1.78	8.5%
6030422M01Rik	0.09018762	0.04	39.6%
6030422M02Rik	0.00261366	0.01	223.6%
6030429G01Rik	0.11514462	0.04	32.6%
6030436E02Rik	4.006972	0.66	16.6%
6030441H18Rik	1.0413162	0.12	11.8%
6030443O07Rik	13.59284	1.02	7.5%
6030446N20Rik	2.287006	0.24	10.4%
6030458C11Rik	12.0727	0.76	6.3%
6030465E24Rik	3.479902	0.42	12.1%
6030490I01Rik	6.256616	0.40	6.4%
6030498E09Rik	0.01571336	0.02	137.0%
6130401L20Rik	1.334034	0.10	7.6%
6230405M12Rik	3.614916	0.90	25.0%
6230409E13Rik	13.38222	1.61	12.0%
6230410P16Rik	0.3898926	0.03	7.7%
6230416J20Rik	1.514164	0.07	4.9%
6230424H07Rik	0.5926432	0.04	6.9%
6230427J02Rik	1.0449202	0.22	21.3%
6330403A02Rik	7.707802	0.86	11.1%
6330403K07Rik	18.19514	1.74	9.5%
6330406I15Rik	0.2477728	0.09	34.6%
6330407G11Rik	15.20946	1.64	10.8%
6330407J23Rik	20.555	1.47	7.2%
6330408A02Rik	2.493706	0.24	9.8%
6330409N04Rik	20.9526	1.70	8.1%
6330416G13Rik	2.039282	0.22	10.7%
6330416L07Rik	2.81312	0.33	11.7%
6330417G02Rik	3.105242	0.33	10.6%
6330419J24Rik	21.27292	1.01	4.7%
6330439K17Rik	80.03024	5.45	6.8%
6330442E10Rik	31.91406	2.54	7.9%
6330500D04Rik	6.160942	0.31	5.1%
6330503C03Rik	24.37046	0.86	3.5%
6330503K22Rik	10.128314	0.51	5.0%
6330505N24Rik	3.177562	0.23	7.2%
6330512M04Rik	6.718628	0.91	13.6%
6330514A18Rik	1.675626	0.15	9.0%
6330527O06Rik	22.45688	1.43	6.4%
6330534C20Rik	2.83017	0.17	6.1%
6330545A04Rik	0.154736	0.04	26.1%
6330548G22Rik	3.25127	0.26	8.1%

6330569M22Rik	24.87844	2.54	10.2%
6330577E15Rik	50.46518	1.50	3.0%
6330578E17Rik	26.68248	1.12	4.2%
6332401O19Rik	8.614232	0.74	8.6%
6430502M16Rik	10.288438	0.63	6.1%
6430510M02Rik	6.78033	0.96	14.2%
6430514L14Rik	4.309782	0.36	8.4%
6430517E21Rik	17.67592	0.86	4.9%
6430526N21Rik	5.167972	0.24	4.6%
6430527G18Rik	13.05168	0.86	6.6%
6430531B16Rik	0.06734326	0.03	46.6%
6430537H07Rik	0.433477	0.06	13.7%
6430548M08Rik	32.30456	0.93	2.9%
6430550H21Rik	50.3196	2.86	5.7%
6430571L13Rik	2.444242	0.39	15.9%
6430573F11Rik	0.8808318	0.47	53.3%
6430598A04Rik	22.36196	1.10	4.9%
6430604K15Rik	1.125394	0.10	9.0%
6430701C03Rik	0.4512992	0.08	16.9%
6430704M03Rik	15.97652	1.01	6.3%
6430706D22Rik	4.30768	0.55	12.7%
6530401C20Rik	0.2273482	0.07	29.3%
6530401N04Rik	5.603064	0.41	7.3%
6530403A03Rik	7.544628	0.56	7.4%
6530404N21Rik	0.7295136	0.15	19.9%
6530418L21Rik	4.986306	0.36	7.2%
6620401K05Rik	5.151018	0.24	4.8%
6620401M08Rik	7.991258	1.51	18.8%
6720456B07Rik	56.63664	4.56	8.0%
6720456H20Rik	2.007722	0.12	5.8%
6720457D02Rik	2.439196	0.35	14.3%
6720458F09Rik	3.819556	0.58	15.1%
6720460F02Rik	0.7415178	0.12	16.5%
6720463M24Rik	0.5200918	0.06	11.3%
6720467C03Rik	27.08516	0.84	3.1%
6820408C15Rik	0.09032438	0.02	18.2%
6820424L24Rik	4.172708	0.33	7.8%
6820431F20Rik	30.21146	1.28	4.3%
6D	0	0.00	
6M21	0	0.00	
7420416P09Rik	0.007966564	0.01	97.0%
7420426K07Rik	0	0.00	
7530404M11Rik	0.011288798	0.01	71.9%
8030411F24Rik	0	0.00	
8030451F13Rik	0.017950818	0.01	56.6%
8030451K01Rik	2.013934	0.21	10.3%
8030462N17Rik	6.88521	0.51	7.4%

8030466O12Rik	0.10206724	0.03	29.3%
8030491N06Rik	0.6940612	0.06	9.3%
8230402K04Rik	0.01449436	0.01	99.7%
8430406I07Rik	1.2389082	0.16	13.0%
8430408G22Rik	0.21421812	0.12	55.3%
8430410A17Rik	5.823682	0.56	9.6%
8430410K20Rik	21.50202	0.38	1.8%
8430415E04Rik	5.94478	0.36	6.0%
8430416H19Rik	0.04804588	0.03	72.7%
8430419L09Rik	23.88012	1.06	4.4%
8430426H19Rik	0.9468424	0.36	38.4%
8430427H17Rik	6.838954	0.72	10.6%
9030025P20Rik	7.62029	1.59	20.9%
9030205A07Rik	5.01341	0.13	2.7%
9030224M15Rik	0.05305506	0.04	75.9%
9030409G11Rik	14.20394	1.47	10.4%
9030416H16Rik	0.67540734	0.83	122.9%
9030418K01Rik	8.767552	0.81	9.2%
9030421J09Rik	0	0.00	
9030425E11Rik	9.44958	0.26	2.7%
9030605I04Rik	0.026527366	0.03	101.7%
9030607L17Rik	5.136216	0.60	11.7%
9030611O19Rik	0.02674704	0.02	57.3%
9030612M13Rik	12.99498	0.74	5.7%
9030617O03Rik	0.6607558	0.12	17.8%
9030619P08Rik	0	0.00	
9030624G23Rik	0.9292728	0.12	12.6%
9030624J02Rik	20.8764	0.68	3.2%
9030625A04Rik	0.6410742	0.09	13.5%
9130005N14Rik	2.155438	0.47	21.7%
9130008F23Rik	0.04860964	0.01	30.1%
9130011E15Rik	12.425	0.61	4.9%
9130011J15Rik	24.49718	1.41	5.7%
9130014G24Rik	0	0.00	
9130017C17Rik	0.10903286	0.03	23.4%
9130017N09Rik	0.03827756	0.02	48.3%
9130019O22Rik	0.4423836	0.09	20.6%
9130023D20Rik	3.633062	0.38	10.6%
9130023H24Rik	1.20495	0.16	13.0%
9130206N08Rik	12.4352	0.48	3.8%
9130211I03Rik	0.4228398	0.22	52.2%
9130213B05Rik	9.457196	0.23	2.5%
9130218O11Rik	0.00519282	0.01	223.6%
9130221D24Rik	0	0.00	
9130221H12Rik	1.757266	0.22	12.6%
9130227C08Rik	3.198266	0.35	10.9%
9130401M01Rik	11.00728	0.51	4.6%

9130404D08Rik	18.50016	1.11	6.0%
9130404D14Rik	5.414408	0.46	8.5%
9130404H23Rik	0	0.00	
9130409I23Rik	0.0058821	0.01	223.6%
9130416B15	0	0.00	
9230019H11Rik	0	0.00	
9230104L09Rik	0.12537358	0.15	122.6%
9230105E10Rik	0.1561146	0.04	24.1%
9230107M04Rik	0	0.00	
9230109A22Rik	0.00430008	0.01	223.6%
9230110C19Rik	3.362756	0.23	6.7%
9230112D13Rik	0	0.00	
9230116B18Rik	0.9374302	0.27	29.2%
9330101J02Rik	1.334356	0.08	6.0%
9330128J19Rik	2.627956	0.24	9.1%
9330129D05Rik	0.6088828	0.04	6.8%
9330134C04Rik	3.262522	0.20	6.2%
9330155M09Rik	0.01336952	0.01	93.5%
9330158F14Rik	0.006431	0.01	223.6%
9330176C04Rik	0.1993658	0.05	25.6%
9330182L06Rik	14.02092	0.44	3.2%
9430007A20Rik	0	0.00	
9430010O03Rik	21.93472	1.36	6.2%
9430015G10Rik	5.792038	0.40	6.8%
9430016H08Rik	5.538524	0.24	4.4%
9430020K01Rik	2.614628	0.26	9.9%
9430022F06Rik	0.00320218	0.01	223.6%
9430023L20Rik	14.14152	0.48	3.4%
9430028L06Rik	1.506242	0.12	8.2%
9430031J16Rik	2.743794	0.06	2.0%
9430038I01Rik	0.3818206	0.09	22.4%
9430057O19Rik	0.465962	0.18	38.1%
9430067K14Rik	3.647678	0.25	6.8%
9430069I07Rik	0.01179612	0.03	223.6%
9430069J07Rik	8.435932	0.24	2.9%
9430070O13Rik	0.01718982	0.01	63.8%
9430076C15Rik	0.013649282	0.03	194.8%
9530002B09Rik	0	0.00	
9530003J23Rik	0.0076999	0.02	223.6%
9530008L14Rik	0.00404006	0.01	223.6%
9530015I07Rik	0.07133444	0.01	19.5%
9530048O09Rik	1.0229518	0.14	13.6%
9530053A07Rik	0	0.00	
9530058B02Rik	5.235914	0.73	14.0%
9530066K23Rik	0.02500054	0.02	63.0%
9530068E07Rik	47.20682	1.36	2.9%
9530077C05Rik	2.81941	0.16	5.8%

9530091C08Rik	0.00239268	0.01	223.6%
9530096D07Rik	0.7840232	0.20	25.7%
9530098N22Rik	0	0.00	
9630008K15Rik	0.5884682	0.14	24.2%
9630014M24Rik	0.6164676	0.08	12.6%
9630025I21Rik	2.696412	0.22	8.0%
9630028B13Rik	0.0635019	0.03	55.0%
9630031F12Rik	5.295526	0.98	18.5%
9630033F20Rik	5.757086	0.36	6.3%
9630041N07Rik	0.6966144	0.08	11.5%
9630046K23Rik	7.731484	0.93	12.1%
9630050M13Rik	14.59978	0.95	6.5%
9630055N22Rik	1.3015682	0.64	49.2%
9630058J23Rik	9.491292	0.25	2.6%
9830102E05Rik	0.00384512	0.01	223.6%
9830107B12Rik	0	0.00	
9830123M21Rik	0.02898646	0.01	36.2%
9830124H08Rik	3.304752	0.49	14.8%
9830130M13Rik	0.0064496	0.01	137.8%
9830134C10Rik	0.001049026	0.00	223.6%
9830163H01Rik	0.07484558	0.05	61.5%
9830169C18Rik	3.737518	0.15	4.0%
9930012K11Rik	0.6013894	0.14	24.0%
9930013L23Rik	2.886358	0.49	16.9%
9930021D14Rik	1.76120894	1.58	89.9%
9930021J03Rik	10.93878	0.57	5.2%
9930021J17Rik	0.1677872	0.07	39.6%
9930022D16Rik	0.00251174	0.01	223.6%
9930022N03Rik	0.00546622	0.01	223.6%
9930023K05Rik	0.04564856	0.03	57.2%
9930032O22Rik	0	0.00	
9930039A11Rik	0.001877058	0.00	223.6%
9930104L06Rik	3.894052	0.25	6.4%
9930109F21Rik	0.00317352	0.01	223.6%
9930111J21Rik	0	0.00	
a	0.30257518	0.44	143.9%
A030001H23Rik	0	0.00	
A030004J04Rik	0	0.00	
A030007L17Rik	5.797386	0.39	6.7%
A030009A09Rik	0	0.00	
A030009H04Rik	59.21644	0.53	0.9%
A030010K20Rik	0	0.00	
A030013N09Rik	0	0.00	
A130010J15Rik	1.590646	0.10	6.5%
A130022J15Rik	2.62772	0.30	11.5%
A130023I24Rik	0	0.00	
A130042E20Rik	1.20378	0.12	9.7%

A130090K04Rik	0.215635516	0.13	58.3%
A130092J06Rik	0.4615596	0.14	30.3%
A1bg	0	0.00	
A230046K03Rik	10.408966	0.75	7.2%
A230050P20Rik	20.93298	1.00	4.8%
A230051G13Rik	1.907598	0.10	5.3%
A230054D04Rik	11.41852	0.69	6.0%
A230062G08Rik	2.513618	0.12	4.7%
A230067G21Rik	0.638739	0.35	55.6%
A230069A22Rik	0.23095596	0.12	52.2%
A230074B11Rik	0.00925302	0.01	140.3%
A230078I05Rik	8.978374	0.76	8.4%
A230079K17Rik	0	0.00	
A230083G16Rik	0.7993336	0.17	21.7%
A230083H22Rik	28.15664	1.66	5.9%
A230097K15Rik	3.0783	0.29	9.5%
A230106M15Rik	4.425432	0.33	7.5%
A230106M20Rik	43.69866	4.31	9.9%
A230106N23Rik	0.6549926	0.06	9.4%
A230107C01Rik	0.0514619	0.03	66.0%
A2bp1	97.22112	2.62	2.7%
A2m	2.548964	0.17	6.6%
A330008L17Rik	0.9281836	0.24	26.1%
A330019N05Rik	0.05279212	0.02	33.8%
A330021E22Rik	1.396444	0.09	6.3%
A330049M08Rik	0.4806444	0.12	24.3%
A330070K13Rik	0.2592876	0.06	23.3%
A330102H22Rik	2.140052	0.41	19.3%
A330102K23Rik	10.667948	1.04	9.8%
A330104H05Rik	0.01525578	0.01	60.7%
A3galt2	0.09571574	0.05	48.0%
A430005L14Rik	4.048952	0.28	6.8%
A430025D11Rik	0	0.00	
A430033K04Rik	3.221552	0.16	4.9%
A430041B07Rik	10.76842	0.38	3.5%
A430060F13Rik	0.0074476	0.01	93.8%
A430065P19Rik	0.013670792	0.01	78.5%
A430078G23Rik	0	0.00	
A430083B19Rik	0	0.00	
A430084P05Rik	0	0.00	
A430089I19Rik	0	0.00	
A430090E18Rik	0	0.00	
A430090L17Rik	0.003913696	0.01	141.4%
A430093A21Rik	1.269978	0.17	13.7%
A430105I19Rik	0.315335	0.06	18.3%
A430106J12Rik	0.3952724	0.16	39.9%
A430107D22Rik	0.001281094	0.00	223.6%

A430107O13Rik	0.7502212	0.17	22.4%
A430107P09Rik	0	0.00	
A430110N23Rik	0.1829186	0.05	26.4%
A4galt	0.4089764	0.05	12.2%
A4gnt	0	0.00	
A530016L24Rik	0	0.00	
A530016O06Rik	0.08827368	0.04	44.5%
A530023O14Rik	0.00246742	0.01	223.6%
A530032D15Rik	0.008972	0.01	137.2%
A530050D06Rik	0.08169848	0.01	17.6%
A530053G22Rik	0	0.00	
A530054K11Rik	2.345092	0.20	8.4%
A530057A03Rik	0.7267102	0.06	8.9%
A530064D06Rik	0	0.00	
A530082C11Rik	13.92428	0.29	2.1%
A530088E08Rik	0.00729452	0.01	137.0%
A530088H08Rik	0.03940298	0.03	71.1%
A530088I07Rik	0.2144372	0.03	15.2%
A530095I07Rik	0	0.00	
A530098C11Rik	0.0509196	0.03	60.5%
A530099J19Rik	0	0.00	
A630001G21Rik	0.03322532	0.02	45.2%
A630005I04Rik	0.00217254	0.00	223.6%
A630007B06Rik	14.95462	1.89	12.6%
A630010A05Rik	0	0.00	
A630012P03Rik	0	0.00	
A630018P17Rik	0.9323174	0.05	5.9%
A630033E08Rik	3.846922	0.16	4.1%
A630033H20Rik	0.03912512	0.05	116.6%
A630038E17Rik	0.0805434	0.03	42.1%
A630042L21Rik	7.25251	0.24	3.3%
A630047E20Rik	4.911986	0.33	6.7%
A630050E13Rik	0.1404824	0.04	27.3%
A630054L15Rik	9.29374	0.54	5.8%
A630055G03Rik	0.12750104	0.08	61.1%
A630072M18Rik	1.0858606	0.12	11.0%
A630077B13Rik	0.02840666	0.04	140.0%
A630091E08Rik	0	0.00	
A630095E13Rik	0	0.00	
A630098G03Rik	0	0.00	
A730008H23Rik	0.5363292	0.23	43.2%
A730008L03Rik	1.345338	0.27	20.3%
A730011L01Rik	6.1842	0.09	1.4%
A730017C20Rik	16.37716	0.62	3.8%
A730018C14Rik	0	0.00	
A730020M07Rik	0.0222635	0.04	166.6%
A730036I17Rik	0.05346768	0.02	36.0%

A730037C10Rik	0.20994558	0.12	56.7%
A730055C05Rik	0.2347186	0.10	44.3%
A730069N07Rik	0.4884952	0.16	33.4%
A730085E03Rik	0	0.00	
A730098D12Rik	1.5555966	0.73	46.8%
A830006F12Rik	5.966988	0.24	4.1%
A830007P12Rik	0.2085218	0.05	25.6%
A830008O07Rik	3.32923	0.57	17.1%
A830010M20Rik	4.26228	2.48	58.3%
A830018L16Rik	7.36929	0.75	10.1%
A830019P07Rik	0.3452612	0.19	56.3%
A830023I12Rik	3.029192	0.44	14.6%
A830036E02Rik	0	0.00	
A830043J08Rik	0.00908682	0.01	106.0%
A830053O21Rik	0.106086	0.06	52.5%
A830059I20Rik	7.060902	0.69	9.8%
A830073O21Rik	9.58234	0.57	6.0%
A830080D01Rik	1.963774	0.16	8.4%
A830091I15Rik	10.743558	0.99	9.2%
A830093I24Rik	0.5699726	0.20	35.9%
A930001M12Rik	0.204505	0.04	20.6%
A930001N09Rik	4.292688	1.00	23.4%
A930005I04Rik	0.217016	0.02	9.4%
A930006D11Rik	0.03733738	0.02	60.1%
A930008G19Rik	1.65697	0.29	17.3%
A930016P21Rik	2.28914	0.26	11.5%
A930017N06Rik	4.369728	0.26	6.0%
A930018P22Rik	0.01419596	0.02	141.4%
A930021C24Rik	0.1386522	0.04	27.1%
A930021H16Rik	2.275216	0.14	6.3%
A930025D01Rik	2.932604	0.36	12.4%
A930028L21Rik	0.22338536	0.14	61.7%
A930034L06Rik	321.1904	45.80	14.3%
A930037G23Rik	7.894552	0.30	3.8%
A930038C07Rik	4.232806	0.21	5.0%
A930041I02Rik	14.39102	1.46	10.2%
AA388235	1.36535	0.16	11.5%
AA407270	0.0836716	0.03	40.3%
AA407659	0.07985504	0.05	63.5%
AA408296	2.502504	0.29	11.6%
AA409316	1.59048	0.09	5.7%
AA415398	2.723924	0.25	9.4%
AA467197	0.10474734	0.08	79.2%
AA536717	8.773424	0.36	4.1%
AA536749	23.5355	13.17	56.0%
AA673488	8.118034	0.15	1.8%
AA792892	0	0.00	

AA881470	5.17932	0.12	2.4%
AA960436	3.393952	0.34	10.0%
AA986860	0.7041584	0.11	16.1%
AA987161	2.971696	0.11	3.8%
Aaas	10.304776	1.15	11.2%
Aacs	32.5143	4.76	14.6%
Aadac	0	0.00	
Aadacl1	33.27572	2.30	6.9%
Aadacl3	0	0.00	
Aadat	0.2172164	0.08	35.0%
Aak1	33.54156	2.78	8.3%
Aamp	66.39012	2.75	4.1%
Aanat	0.07619144	0.03	42.9%
Aard	3.372188	0.33	9.9%
Aars	84.58456	9.57	11.3%
Aars2	4.59588	0.40	8.8%
Aarsd1	15.20714	1.47	9.7%
Aarsl	0.4410396	0.13	29.6%
Aasdhppt	14.14792	0.42	2.9%
Aass	0.07825752	0.03	40.4%
Aatf	10.182022	0.41	4.1%
Aatk	27.23226	1.09	4.0%
AB013468	0.4152972	0.28	67.5%
AB033515	0	0.00	
AB033524	0	0.00	
AB041550	0.3954328	0.11	27.2%
AB041800	0.5930352	0.16	27.4%
AB056442	0	0.00	
AB080658	0	0.00	
AB112350	2.14167	0.31	14.7%
AB124611	0.00438304	0.01	223.6%
AB182283	12.33106	0.36	2.9%
AB251060	0	0.00	
AB251100	0	0.00	
AB251138	0	0.00	
AB251164	0	0.00	
AB251181	0	0.00	
AB251235	0	0.00	
AB251236	0	0.00	
AB294520	0.5668768	0.93	164.0%
AB294521	4.264222	1.77	41.4%
AB294522	0.648241384	0.41	63.9%
AB294523	0.6467278	0.49	75.3%
AB294524	2.9165428	3.20	109.7%
AB294526	6.41179	2.51	39.2%
AB294528	7.672966	3.78	49.2%
Abat	98.769	5.62	5.7%

Abca1	2.83699	0.19	6.8%
Abca12	0.00629912	0.01	137.0%
Abca13	0.012376838	0.01	54.4%
Abca14	0	0.00	
Abca15	0	0.00	
Abca16	0	0.00	
Abca17	0.4803562	0.03	6.9%
Abca2	21.18438	1.57	7.4%
Abca3	40.83296	1.20	2.9%
Abca4	0.02047109	0.01	72.9%
Abca5	11.93326	1.15	9.6%
abca5	2.319002	0.42	18.0%
Abca6	0.446087	0.29	64.4%
Abca7	5.564732	0.88	15.8%
Abca8a	1.3372362	0.61	45.4%
abca8a	0.3945558	0.21	54.1%
Abca8b	2.570272	0.31	12.1%
Abca9	1.1398234	0.41	36.3%
Abcb10	10.220462	0.58	5.7%
Abcb11	0.003279422	0.00	138.6%
Abcb1a	0.1020986	0.02	19.0%
Abcb1b	0.3702846	0.02	4.3%
Abcb4	0.353608	0.06	16.8%
Abcb5	0	0.00	
Abcb6	12.93716	0.67	5.2%
Abcb7	7.777142	1.00	12.8%
Abcb8	15.59014	0.34	2.2%
Abcb9	17.07774	1.51	8.9%
Abcc1	3.240298	0.13	4.0%
Abcc10	3.803718	0.12	3.1%
Abcc12	0.10062262	0.03	32.4%
Abcc2	0.008969792	0.01	78.3%
Abcc3	0.015177798	0.01	48.6%
Abcc4	1.19708	0.11	8.8%
Abcc5	32.0405	4.27	13.3%
Abcc6	0	0.00	
Abcc8	5.208564	0.34	6.5%
Abcc9	0.1534606	0.03	19.2%
Abcd1	3.06658	0.22	7.3%
Abcd2	4.511872	0.44	9.7%
Abcd3	29.16594	1.40	4.8%
Abcd4	1.520394	0.17	11.1%
Abce1	27.99334	2.24	8.0%
Abcf1	21.52804	1.05	4.9%
Abcf2	18.54924	0.48	2.6%
Abcf3	19.37008	0.94	4.9%
Abcg1	8.411136	1.23	14.7%

Abcg2	2.471366	0.60	24.4%
Abcg3	0.00553912	0.01	138.2%
Abcg4	20.19574	0.30	1.5%
Abcg5	0.00903914	0.01	93.9%
Abcg8	0.00531976	0.01	141.0%
ABCR	0.03139412	0.06	176.8%
Abhd1	3.120484	0.30	9.5%
Abhd10	15.11326	1.00	6.6%
Abhd11	3.299572	0.73	22.0%
Abhd12	55.25248	1.14	2.1%
Abhd13	10.81071	0.74	6.8%
Abhd14a	5.381584	0.45	8.4%
Abhd14b	4.754016	0.73	15.3%
Abhd2	7.763458	0.27	3.5%
Abhd3	5.87975	0.73	12.4%
Abhd4	18.59434	0.60	3.2%
Abhd5	5.373926	0.45	8.4%
Abhd6	6.449272	0.29	4.6%
Abhd7	2.981206	0.24	8.0%
Abhd8	65.7427	2.41	3.7%
Abhd9	0.0307892	0.02	50.8%
Abi1	26.15372	0.82	3.1%
Abi2	46.56122	2.84	6.1%
Abi3	0	0.00	
Abi3bp	0.1497514	0.05	30.8%
Abl1	5.980232	0.24	4.1%
Abl2	5.834154	0.27	4.6%
Ablim1	17.25326	0.80	4.6%
Ablim2	21.00608	1.56	7.4%
ABLIM2	1.123045	0.76	67.3%
Ablim3	18.32332	3.01	16.4%
Abo	0.01073	0.02	147.5%
Abp1	0.01908966	0.01	68.9%
Abpa	0	0.00	
abParts	0	0.00	
Abpd	0	0.00	
Abpe	0	0.00	
Abpg	0	0.00	
Abpz	0	0.00	
Abr	71.39576	0.79	1.1%
Abra	0.00293298	0.01	223.6%
Abt1	2.537332	0.19	7.5%
Abtb1	8.134036	0.67	8.2%
Abtb2	1.48906	0.14	9.7%
Acaa1a	10.3623	0.32	3.1%
Acaa1b	0.7949888	0.13	17.0%
Acaa2	5.590178	0.37	6.6%

Acaca	8.592154	0.79	9.2%
Acacb	0.3185998	0.03	10.4%
Acad10	0.691485	0.09	13.0%
Acad11	4.665898	0.94	20.1%
Acad8	5.579444	0.31	5.5%
Acad9	7.597216	0.19	2.5%
Acadl	8.671446	0.87	10.0%
Acadm	6.951174	0.47	6.8%
Acads	1.9809	0.29	14.7%
Acadsb	14.61492	1.05	7.2%
Acadvl	12.69928	0.28	2.2%
Acan	0.8833888	0.14	16.2%
Acat1	46.53376	2.79	6.0%
Acat2	57.02066	5.34	9.4%
Acat3	7.573334	0.62	8.2%
Acbd3	13.73016	0.70	5.1%
Acbd4	5.2961	0.88	16.6%
Acbd5	21.47516	0.81	3.8%
Acbd6	20.32646	1.84	9.1%
Accn1	20.27848	1.28	6.3%
Accn2	39.48772	2.49	6.3%
Accn3	0.0425642	0.03	68.1%
Accn4	0.1948974	0.04	21.2%
Accn5	0.01030616	0.02	223.6%
Acd	10.69017	0.62	5.8%
Ace	7.159446	0.19	2.6%
Ace2	0.014457308	0.00	28.9%
Ache	99.39146	7.25	7.3%
Acin1	27.4657	2.84	10.3%
Acly	131.8952	11.42	8.7%
Acmsd	0.080258	0.05	67.7%
Acn9	5.844678	0.53	9.1%
Acnat1	0.00998564	0.01	137.0%
Aco1	12.39916	0.54	4.3%
Aco2	137.9162	1.23	0.9%
Acot1	2.73675	0.13	4.6%
Acot10	7.536092	0.24	3.2%
Acot11	1.511578	0.22	14.5%
Acot12	0.02216256	0.03	114.4%
Acot2	1.911334	0.23	12.0%
Acot3	0.3586152	0.06	15.6%
Acot4	0.1094063	0.03	29.1%
Acot5	0.12909314	0.07	53.0%
Acot6	0.6348482	0.10	16.4%
Acot7	185.425	6.94	3.7%
Acot8	6.222982	0.87	14.1%
Acot9	4.325042	0.65	15.1%

Acox1	44.51668	1.24	2.8%
Acox2	0.01434116	0.01	65.4%
Acox3	4.778924	0.17	3.7%
Acoxl	0.02998102	0.01	38.9%
Acp1	1.178688	0.12	10.0%
Acp2	17.4405	0.81	4.7%
Acp5	0.235529	0.04	15.1%
Acp6	2.5588	0.29	11.5%
Acpl2	2.131526	0.13	6.1%
Acpp	0.01229082	0.01	65.5%
Acr	0.5597584	0.11	18.9%
Acrbp	1.475986	0.19	12.7%
ACRP	0.650613	0.12	18.8%
Acrv1	0	0.00	
Acsbg1	11.848176	1.11	9.3%
Acsbg2	0.00218664	0.00	223.6%
Acsl1	7.442342	0.13	1.7%
Acsl3	50.6491	3.13	6.2%
Acsl4	56.46664	3.17	5.6%
Acsl5	4.748568	0.52	10.9%
Acsl6	34.0429	1.72	5.0%
Acsm1	0	0.00	
Acsm2	0.0031381	0.01	223.6%
Acsm3	1.04807342	0.65	61.8%
Acss1	0.9685128	0.12	12.3%
Acss2	9.754494	1.12	11.4%
Acta1	0.4761718	0.10	20.4%
Acta2	3.433016	1.00	29.1%
Actb	367.5268	42.92	11.7%
Actc1	0.1455218	0.04	26.6%
Actg1	30.10976	3.05	10.1%
Actg2	0.02898956	0.03	96.2%
Actl6a	2.64411	0.33	12.6%
Actl6b	19.68226	1.87	9.5%
Actl7a	0.00708448	0.01	137.8%
Actl7b	0	0.00	
Actn1	10.274128	0.71	6.9%
Actn2	0.3693716	0.03	9.3%
Actn3	1.631326	0.19	11.7%
Actn4	16.22394	1.37	8.4%
ActR	0.468354	0.22	47.5%
Actr10	52.8386	1.12	2.1%
Actr1a	123.5422	8.44	6.8%
Actr1b	53.68658	1.28	2.4%
Actr2	62.66932	3.32	5.3%
Actr3	21.46836	1.51	7.0%
Actr3b	10.89892	0.79	7.3%

Actr5	2.129936	0.15	7.1%
Actr6	9.88259	0.39	4.0%
Actr8	14.4552	0.67	4.7%
Actrt1	0	0.00	
Actrt2	0	0.00	
Acvr1	6.864002	0.14	2.0%
Acvr1b	32.02638	1.82	5.7%
Acvr1c	0.1525416	0.04	25.1%
Acvr2a	13.0117	1.22	9.4%
Acvr2b	1.415344	0.27	19.1%
Acvrl1	0.03094252	0.01	38.3%
Acy1	1.88374	0.16	8.2%
Acy1l2	0.5679396	0.16	28.2%
Acy3	0.2429148	0.09	37.5%
Acyp1	9.10444	0.85	9.3%
Acyp2	25.3663	1.30	5.1%
Ada	0.193813	0.06	28.5%
Adad1	0	0.00	
Adal	6.966436	0.38	5.4%
Adam10	13.47262	0.53	3.9%
Adam11	17.27986	1.63	9.4%
Adam12	0.403062	0.07	16.5%
Adam15	20.0013	0.79	4.0%
Adam17	2.473022	0.26	10.4%
Adam18	0	0.00	
Adam19	3.53985	0.54	15.2%
ADAM19	0.63106	0.30	47.4%
Adam1a	0.4525566	0.11	23.7%
Adam1b	0.017766864	0.02	115.7%
Adam2	0	0.00	
Adam21	0.4499714	0.05	10.6%
ADAM22	38.81234	2.47	6.4%
Adam22	16.58018	3.17	19.1%
Adam23	44.98836	1.87	4.2%
Adam24	0	0.00	
Adam25	0	0.00	
Adam26a	0	0.00	
Adam26b	0	0.00	
Adam28	0.01662794	0.02	114.4%
Adam29	0	0.00	
Adam3	0	0.00	
Adam30	0.00406412	0.01	223.6%
Adam32	0.3082072	0.13	40.9%
Adam33	0.00518404	0.01	223.6%
Adam34	0.0029761	0.01	223.6%
Adam39	0.00320868	0.01	223.6%
Adam4	0.0677817	0.03	51.2%

Adam5	0.0221845	0.02	74.4%
Adam6	0	0.00	
Adam7	0.00714924	0.01	98.2%
Adam8	0.516442	0.15	28.8%
Adam9	15.42936	0.69	4.5%
Adamdec1	0.02948676	0.01	30.3%
Adamts1	2.359958	0.35	15.0%
Adamts10	6.739644	1.11	16.5%
Adamts12	2.239278	0.38	17.0%
Adamts13	0.013654638	0.01	45.8%
Adamts14	0.04763532	0.01	20.4%
Adamts15	1.41821	0.19	13.1%
Adamts16	0.2360192	0.05	21.3%
Adamts17	1.298402	0.23	18.1%
Adamts18	0.298162	0.09	29.5%
Adamts19	0.443267	0.04	9.8%
Adamts2	1.573144	0.26	16.4%
Adamts20	1.1356896	0.16	13.9%
Adamts3	1.56458	0.04	2.8%
Adamts4	0.1561068	0.06	35.8%
Adamts5	1.104964	0.05	4.8%
Adamts6	0.8055704	0.10	12.4%
Adamts7	0.9243138	0.08	8.8%
Adamts8	0.4591398	0.04	7.8%
Adamts9	3.413476	0.54	15.8%
Adamtsl1	2.118294	0.13	6.0%
Adamtsl2	1.2508118	0.36	28.4%
Adamtsl3	0.2590278	0.06	23.6%
Adamtsl4	0.07823148	0.01	18.9%
Adamtsl5	0.0626476	0.02	34.7%
Adar	39.39302	1.18	3.0%
Adar3	1.368592	0.09	6.3%
Adarb1	21.33148	0.90	4.2%
Adarb2	1.830922	0.24	13.0%
Adat1	2.120644	0.27	12.6%
Adat3	1.1877484	0.25	20.8%
Adc	10.374204	0.37	3.6%
Adck1	14.79222	0.29	2.0%
Adck2	8.165208	0.10	1.2%
Adck4	6.481974	0.41	6.3%
Adck5	5.089698	0.41	8.1%
Adcy1	35.31638	1.79	5.1%
Adcy2	35.9468	1.29	3.6%
Adcy3	13.91188	0.24	1.7%
Adcy4	0.02258368	0.01	38.1%
Adcy5	13.8776	0.51	3.6%
Adcy6	7.601348	0.76	10.1%

Adcy7	3.624646	0.41	11.2%
Adcy8	17.74162	0.64	3.6%
Adcy9	6.70155	0.22	3.2%
Adcyap1	1.64703	0.35	21.2%
Adcyap1r1	33.67288	2.43	7.2%
Add1	55.42516	2.29	4.1%
ADD1	9.355026	0.50	5.3%
Add2	33.96544	4.08	12.0%
ADD2	0.51067672	0.78	153.0%
Add3	25.28408	2.25	8.9%
Adfp	1.576588	0.17	11.1%
Adh1	0.8033882	0.27	33.7%
Adh4	0	0.00	
Adh5	27.45472	1.07	3.9%
Adh6a	0	0.00	
Adh6b	0	0.00	
Adh6-ps1	0	0.00	
Adh7	0.04689638	0.03	69.1%
Adhfe1	1.73047	0.86	50.0%
Adi1	8.129366	0.21	2.6%
Adipoq	0.02791982	0.02	63.4%
Adipor1	36.8569	1.91	5.2%
Adipor2	17.4415	0.70	4.0%
Adk	12.14834	0.63	5.2%
Adm	1.3475884	0.46	34.3%
Adm2	0.0631864	0.04	60.6%
Admr	0.06638146	0.03	38.7%
Adnp	33.649	1.13	3.4%
Adora1	11.93766	0.90	7.5%
Adora2a	0.3776996	0.05	12.7%
Adora2b	0.594176	0.26	43.5%
Adora3	0	0.00	
Adpgk	9.007076	0.29	3.2%
Adprh	45.66598	1.35	2.9%
Adprhl1	0	0.00	
Adprhl2	7.92016	0.55	6.9%
Adprt3	0.02958004	0.06	187.2%
Adra1a	2.282118	0.13	5.5%
Adra1b	10.276112	1.38	13.5%
Adra1d	0.8868502	0.08	8.5%
Adra2a	17.66096	0.84	4.8%
Adra2b	0.007961938	0.01	100.2%
Adra2c	0.7679106	0.05	6.3%
Adrb1	1.9138	0.20	10.6%
Adrb2	0.1401438	0.06	43.6%
Adrb3	0.4855288	0.09	18.4%
Adrbk1	47.06478	1.13	2.4%

Adrbk2	6.550494	0.42	6.4%
Adrm1	5.994626	0.80	13.4%
Adsl	11.062668	1.33	12.0%
Adss	40.59482	1.78	4.4%
Adssl1	2.445134	0.36	14.8%
Aebp1	0.5873986	0.13	22.7%
Aebp2	11.50766	0.44	3.9%
Aes	154.4864	6.76	4.4%
AF032968	0	0.00	
AF060570	0.11296294	0.04	31.7%
AF067063	0	0.00	
AF080584	0.01177884	0.03	223.6%
AF090403	11.14484	0.93	8.3%
AF106279	0.01347664	0.03	223.6%
AF118847	0.04048876	0.02	48.9%
AF121215	0.0417737	0.01	23.8%
AF217545	248.3488	13.33	5.4%
AF332090	0.07442826	0.02	25.4%
AF357374	0	0.00	
AF357376	0	0.00	
AF357401	0.335758	0.53	157.4%
AF365932	0.10738562	0.17	154.7%
AF365933	0.04555598	0.04	77.3%
AF366264	0	0.00	
AF380425	0.14901312	0.14	95.5%
AF401983	0	0.00	
AF479022	0	0.00	
AF498300	11.047524	2.65	24.0%
AF498301	11.644996	2.48	21.3%
AF498302	21.14846	2.08	9.8%
AF529169	10.765464	1.23	11.4%
Afap1	7.602032	0.85	11.2%
Afap1l1	0.7253292	0.07	9.0%
Afap1l2	0.4554212	0.08	18.2%
Aff1	1.1730164	0.22	19.1%
Aff2	7.185622	0.56	7.8%
Aff3	10.178546	0.56	5.5%
Aff4	23.7827	3.08	13.0%
Afg3l1	9.410154	0.92	9.8%
Afg3l2	27.9851	0.75	2.7%
Afm	0.00295178	0.01	223.6%
Afmid	1.0721906	0.18	16.4%
Afp	0.00365856	0.01	223.6%
Aftph	16.95326	0.70	4.1%
Aga	0.8136048	0.21	25.5%
Agbl1	0.017644078	0.02	135.8%
Agbl2	0.040458586	0.03	81.5%

Agbl3	2.714886	0.67	24.8%
Agbl5	7.943008	0.49	6.2%
Ager	0.3617254	0.13	37.1%
Aggf1	11.64314	0.41	3.5%
Agk	4.924206	0.17	3.5%
Agl	7.497758	0.79	10.6%
Agmat	0.01342822	0.01	93.1%
Agpat1	55.66076	5.61	10.1%
Agpat2	0.7526106	0.28	36.7%
Agpat3	39.70682	1.14	2.9%
Agpat4	14.1195	1.00	7.1%
Agpat5	14.09498	0.63	4.5%
Agpat6	24.4893	1.33	5.4%
Agpat7	24.07204	1.80	7.5%
Agps	14.46286	0.71	4.9%
Agr2	0.29400396	0.26	89.4%
Agr3	0.01051204	0.02	223.6%
Agrin	9.205592	0.74	8.0%
Agrn	19.00096	1.52	8.0%
Agrp	1.2475936	0.41	32.5%
Agt	0.4005092	0.14	35.0%
Agtpbp1	28.72528	1.59	5.5%
Agtr1a	0.254012	0.03	12.4%
Agtr1b	0.02312494	0.03	132.6%
Agtr2	22.6958	4.68	20.6%
Agtrap	2.127842	0.16	7.6%
Agtrl1	0.06927148	0.06	89.6%
Agxt	0	0.00	
Agxt2	0.01132862	0.02	141.4%
Agxt2l1	0.02569792	0.01	39.0%
Agxt2l2	1.739364	0.44	25.2%
Ahctf1	3.710542	0.48	13.0%
Ahcy	0.12571454	0.04	33.7%
Ahcyl1	61.30344	3.65	5.9%
Ahdc1	5.435606	0.49	9.0%
Ahi1	129.4638	3.84	3.0%
Ahi-1	37.9776	2.26	6.0%
Ahnak	2.553644	0.55	21.4%
Ahnak2	1.123406	0.07	6.5%
Ahr	2.984932	0.18	6.0%
Ahrr	0.04538132	0.02	40.5%
Ahsa1	48.47232	2.44	5.0%
Ahsa2	6.067178	0.85	14.0%
Ahsg	0	0.00	
AI115600	10.64768	0.36	3.4%
AI118078	3.627414	0.62	17.2%
AI132487	1.2415	0.12	9.8%

AI182371	0.1430784	0.04	28.3%
AI314180	29.02204	1.57	5.4%
AI314976	3.474592	0.21	6.1%
AI316787	19.31386	0.51	2.6%
AI316807	18.1889	0.63	3.5%
AI317237	7.346176	0.62	8.4%
AI317395	0.09422538	0.02	22.6%
AI324046	0.0031421	0.01	223.6%
AI413582	32.30442	1.37	4.2%
AI413782	13.21092	0.49	3.7%
AI427122	0.5833762	0.08	13.5%
AI427515	59.80826	2.96	5.0%
AI427809	0.03678648	0.02	62.3%
AI428936	0.8351486	0.04	4.3%
AI429214	0.583084	0.14	23.7%
AI449175	5.851524	0.55	9.3%
AI450540	11.64316	0.68	5.8%
AI450948	0.322315	0.13	39.3%
AI451557	0.13838928	0.05	38.2%
AI451617	0.01610434	0.03	161.5%
AI461788	2.434452	0.13	5.3%
AI462493	13.9037	0.75	5.4%
AI464131	1.894272	0.15	7.7%
AI467606	0	0.00	
AI480556	2.287972	0.23	10.1%
AI480653	18.03674	1.32	7.3%
AI481877	0.00408518	0.01	223.6%
AI504432	6.21817	0.38	6.1%
AI593442	88.0024	1.70	1.9%
AI595366	2.147036	0.25	11.4%
AI595406	16.93204	0.97	5.7%
AI597468	14.32192	0.62	4.3%
AI597479	11.505	0.72	6.3%
AI606181	1.1486064	0.29	25.4%
AI607873	0	0.00	
AI646023	8.307466	0.50	6.0%
AI661453	0.05204212	0.05	91.0%
AI662250	3.310718	0.24	7.2%
AI747448	0	0.00	
AI747699	0.2034578	0.05	25.9%
AI790298	0.8573568	0.07	8.0%
AI836003	20.26632	2.53	12.5%
AI837181	32.2472	1.81	5.6%
AI842396	1.452346	0.10	6.7%
AI846148	12.24308	0.21	1.7%
AI847670	5.454972	0.27	4.9%
AI848100	14.11	0.54	3.8%

AI851790	26.9096	1.38	5.1%
AI854703	12.35578	0.85	6.9%
AI894139	5.197914	0.20	3.9%
AI931714	9.486048	0.46	4.8%
AI987662	0.12901082	0.03	23.9%
AI987692	0.05243654	0.04	71.5%
AI987944	4.15134	0.54	12.9%
Aicda	0.0210776	0.02	111.2%
Aif1	0.03963548	0.01	33.1%
Aifl	2.460408	0.88	35.6%
Aifm1	13.16088	0.97	7.4%
Aifm2	5.809318	1.06	18.3%
Aifm3	2.817486	0.43	15.2%
Aig1	31.5787	1.91	6.0%
Aim1	0.0208011	0.02	81.8%
Aim1l	0.16932558	0.06	38.4%
Aim2	0.04368848	0.03	67.1%
Aip	31.52182	1.63	5.2%
Aipl1	0.05734776	0.06	99.1%
Aire	0	0.00	
AJ001378	0	0.00	
AJ311366	0	0.00	
AJ517767	7.808036	1.48	19.0%
Ajap1	9.228412	0.74	8.0%
AK002250	0	0.00	
AK002372	1.6995162	0.62	36.3%
AK002390	0.8859892	0.07	7.6%
AK002417	0	0.00	
AK002541	0.53100708	0.69	130.6%
AK002663	0.0173931	0.02	139.7%
AK002708	0.0127786	0.01	100.0%
AK002723	0.2616564	0.11	40.4%
AK002733	0	0.00	
AK002748	0.04967466	0.05	104.9%
AK002838	0	0.00	
AK002860	1.555658	0.26	16.6%
AK002866	0.0767851	0.02	19.7%
AK002929	0.03421382	0.03	99.8%
AK003073	0.2374108	0.08	33.1%
AK003103	0.04249966	0.03	59.1%
AK003136	0.25001692	0.21	82.3%
AK003315	85.25648	6.62	7.8%
AK003327	0	0.00	
AK003573	11.16208	0.71	6.4%
AK003663	0	0.00	
AK003723	0.2486564	0.08	34.2%
AK003793	1.081541	0.60	55.9%

AK003800	0.9154782	0.27	29.1%
AK003812	0.24538632	0.18	74.0%
AK003841	0.3104328	0.10	31.0%
AK003943	0.00585342	0.01	223.6%
AK004150	7.545708	0.44	5.8%
AK004173	3.5057378	1.54	44.0%
AK004186	0.8781744	0.32	36.2%
AK004221	0.01902628	0.03	137.2%
AK004226	0	0.00	
AK004252	0.635482	0.09	13.4%
AK004338	95.64566	2.22	2.3%
AK004382	25.4129	2.07	8.2%
AK004399	162.9982	8.42	5.2%
AK004434	0.1935828	0.08	39.7%
AK004512	18.66178	0.93	5.0%
AK004513	0.6247668	0.27	43.9%
AK004585	0.7601804	0.17	21.9%
AK004727	37.78382	17.83	47.2%
AK004850	0.2672298	0.08	29.8%
AK005093	2.761178	0.38	13.7%
AK005115	0.52941	0.10	18.8%
AK005116	8.828756	1.09	12.3%
AK005183	5.451364	0.66	12.2%
AK005214	0.3562866	0.09	26.3%
AK005275	0.04933012	0.01	20.3%
AK005305	1.246301	0.23	18.7%
AK005306	1.416686	0.19	13.5%
AK005363	0.2208116	0.16	72.4%
AK005385	0.9079528	0.09	10.0%
AK005414	0	0.00	
AK005418	0	0.00	
AK005477	0.06373964	0.03	43.1%
AK005481	2.0435914	0.71	34.9%
AK005503	4.125268	0.45	11.0%
AK005538	0.4742394	0.22	46.5%
AK005539	0	0.00	
AK005556	2.891642	0.33	11.3%
AK005569	0.1882309	0.18	96.5%
AK005576	0	0.00	
AK005578	0.008351	0.02	223.6%
AK005580	0	0.00	
AK005589	0	0.00	
AK005591	0.19027248	0.15	77.8%
AK005600	0	0.00	
AK005607	0	0.00	
AK005608	0.20860882	0.09	43.2%
AK005616	0.10480626	0.07	64.5%

AK005629	0	0.00	
AK005634	0.003518716	0.01	152.2%
AK005635	0	0.00	
AK005639	0	0.00	
AK005641	1.604364	0.62	38.9%
AK005643	0	0.00	
AK005649	0	0.00	
AK005651	0.15201656	0.07	45.9%
AK005653	0.5307186	0.17	31.1%
AK005657	0	0.00	
AK005663	0.3393674	0.12	33.9%
AK005667	0	0.00	
AK005677	0.03984338	0.04	108.6%
AK005678	0.17938614	0.22	120.7%
AK005679	0.1255996	0.02	18.6%
AK005686	0	0.00	
AK005707	0.05789542	0.04	74.9%
AK005712	0	0.00	
AK005722	0	0.00	
AK005744	0.01517008	0.02	137.0%
AK005746	0.4365072	0.09	20.1%
AK005754	0	0.00	
AK005757	7.578178	0.36	4.7%
AK005780	0.01508388	0.03	223.6%
AK005784	0.5731066	0.13	23.3%
AK005793	0.03917803	0.05	134.0%
AK005807	0	0.00	
AK005811	0.01355466	0.02	141.3%
AK005822	0.03253124	0.02	63.8%
AK005828	0	0.00	
AK005833	0	0.00	
AK005834	0.00601656	0.01	223.6%
AK005838	1.278838	0.31	24.4%
AK005839	0	0.00	
AK005849	0	0.00	
AK005856	0	0.00	
AK005867	0	0.00	
AK005877	0.01461408	0.03	223.6%
AK005894	0.107463	0.11	101.5%
AK005895	0	0.00	
AK005897	0.10208554	0.11	104.2%
AK005898	0.3213372	0.14	43.3%
AK005905	0.09964674	0.11	110.5%
AK005912	0	0.00	
AK005951	0.3457344	0.15	43.7%
AK005956	0.027222	0.04	141.9%
AK005958	0	0.00	

AK005967	1.781402	0.36	20.3%
AK005987	0	0.00	
AK005994	0	0.00	
AK006001	0.1298988	0.09	67.0%
AK006022	0.1674216	0.12	73.1%
AK006024	0	0.00	
AK006029	0	0.00	
AK006031	0	0.00	
AK006037	0.064201	0.14	223.6%
AK006039	0	0.00	
AK006045	0.02104276	0.03	152.4%
AK006051	0.13846056	0.03	23.7%
AK006052	0	0.00	
AK006056	0	0.00	
AK006070	0	0.00	
AK006071	0	0.00	
AK006075	0	0.00	
AK006076	0	0.00	
AK006082	4.785176	0.17	3.5%
AK006087	0.08321332	0.06	68.2%
AK006104	0.00260282	0.01	223.6%
AK006108	0	0.00	
AK006114	0	0.00	
AK006119	0.21284756	0.12	57.5%
AK006121	0	0.00	
AK006131	0	0.00	
AK006138	0.019267698	0.02	121.6%
AK006139	0	0.00	
AK006160	0	0.00	
AK006167	0.01277534	0.03	223.6%
AK006173	0.05236606	0.03	59.0%
AK006186	0	0.00	
AK006189	0	0.00	
AK006191	0.14817926	0.06	42.5%
AK006196	0.1103602	0.05	49.8%
AK006202	0	0.00	
AK006231	0	0.00	
AK006239	0.1162965	0.10	83.3%
AK006240	0	0.00	
AK006245	0.0579038	0.08	140.8%
AK006250	0.1628252	0.02	13.2%
AK006252	0.7147798	0.14	19.0%
AK006255	0.11061238	0.06	57.4%
AK006264	0	0.00	
AK006272	0	0.00	
AK006282	0.01940108	0.02	98.6%
AK006292	0	0.00	

AK006293	0	0.00	
AK006304	0	0.00	
AK006308	0.01019722	0.02	223.6%
AK006310	0.0075329	0.02	223.6%
AK006321	0	0.00	
AK006331	0	0.00	
AK006334	0.01477118	0.02	139.0%
AK006343	0	0.00	
AK006348	0	0.00	
AK006355	0	0.00	
AK006362	0	0.00	
AK006376	0	0.00	
AK006380	0	0.00	
AK006388	0.00392746	0.01	223.6%
AK006389	0	0.00	
AK006397	0.08070672	0.07	81.6%
AK006409	0	0.00	
AK006412	0	0.00	
AK006413	0	0.00	
AK006426	0	0.00	
AK006434	0.1402126	0.03	22.1%
AK006443	0	0.00	
AK006451	0	0.00	
AK006454	0.10137776	0.08	81.8%
AK006455	0	0.00	
AK006457	0	0.00	
AK006461	0.0134464	0.02	139.5%
AK006465	0	0.00	
AK006470	0	0.00	
AK006474	0	0.00	
AK006485	0	0.00	
AK006486	0	0.00	
AK006507	0	0.00	
AK006510	0	0.00	
AK006512	0	0.00	
AK006531	0	0.00	
AK006540	0	0.00	
AK006546	0.00289758	0.01	223.6%
AK006560	0	0.00	
AK006561	0.0757494	0.06	77.6%
AK006563	0	0.00	
AK006568	0	0.00	
AK006570	0	0.00	
AK006572	0	0.00	
AK006576	0.06282626	0.02	37.2%
AK006581	0	0.00	
AK006587	0	0.00	

AK006595	0.17824444	0.12	65.3%
AK006607	0.2012256	0.08	41.3%
AK006610	0.15209618	0.10	69.0%
AK006623	0	0.00	
AK006633	0	0.00	
AK006637	0.0937403	0.03	35.7%
AK006644	0.04575366	0.07	157.5%
AK006651	1.4538286	0.36	24.7%
AK006653	0	0.00	
AK006659	0	0.00	
AK006664	0	0.00	
AK006677	0	0.00	
AK006690	0	0.00	
AK006701	0	0.00	
AK006702	0	0.00	
AK006706	0	0.00	
AK006709	0	0.00	
AK006725	0.0130054	0.02	141.4%
AK006730	0.03471526	0.05	137.5%
AK006734	0.17934958	0.11	62.6%
AK006738	0	0.00	
AK006748	0.2509304	0.12	48.9%
AK006752	0.04888076	0.09	185.7%
AK006756	0	0.00	
AK006766	0	0.00	
AK006768	0	0.00	
AK006782	0	0.00	
AK006812	0.00640402	0.01	223.6%
AK006824	0	0.00	
AK006831	0.05395912	0.02	29.6%
AK006839	0.02945846	0.03	91.7%
AK006844	0	0.00	
AK006852	0	0.00	
AK006859	0	0.00	
AK006870	0	0.00	
AK006884	0.06659024	0.07	111.9%
AK006889	0.11901528	0.06	50.7%
AK006894	0	0.00	
AK006895	0.1560874	0.10	66.6%
AK006896	0	0.00	
AK006899	0	0.00	
AK006906	5.428264	0.28	5.2%
AK006909	0	0.00	
AK006921	0.0022477	0.01	223.6%
AK006923	0	0.00	
AK006930	0	0.00	
AK006933	0.06236384	0.06	103.2%

AK006934	0	0.00	
AK006937	0	0.00	
AK006941	0.3351852	0.27	79.1%
AK006942	0	0.00	
AK006947	0	0.00	
AK006958	0	0.00	
AK006961	0	0.00	
AK006968	0	0.00	
AK006971	0	0.00	
AK006973	0.10709264	0.03	32.5%
AK006978	0	0.00	
AK006980	0.0202271	0.02	97.5%
AK006988	0.5098092	0.28	54.1%
AK006990	0.212649	0.06	28.1%
AK006991	0	0.00	
AK006996	0	0.00	
AK006997	0	0.00	
AK007006	0	0.00	
AK007011	0.688814	0.12	17.7%
AK007020	2.10944	0.29	13.5%
AK007025	0	0.00	
AK007030	0	0.00	
AK007038	0	0.00	
AK007041	0	0.00	
AK007042	0	0.00	
AK007043	0.04182156	0.06	152.5%
AK007073	0	0.00	
AK007082	0.13080512	0.06	43.6%
AK007083	0	0.00	
AK007084	0	0.00	
AK007092	0.04860142	0.03	59.6%
AK007095	0	0.00	
AK007100	0.29716578	0.48	161.0%
AK007102	0	0.00	
AK007110	0	0.00	
AK007112	0	0.00	
AK007116	0	0.00	
AK007118	0.00859428	0.02	223.6%
AK007133	0	0.00	
AK007137	0	0.00	
AK007148	0.07557648	0.03	45.4%
AK007149	0.06568994	0.07	111.8%
AK007151	0	0.00	
AK007154	0.2749464	0.06	22.7%
AK007159	0.00430558	0.01	223.6%
AK007162	0.10056314	0.13	133.7%
AK007165	0.839788	0.26	30.8%

AK007174	0.12732548	0.05	41.7%
AK007175	0.04117902	0.04	99.5%
AK007185	0	0.00	
AK007191	0.8759392	0.93	106.1%
AK007204	0	0.00	
AK007205	0	0.00	
AK007210	0.14945732	0.07	44.1%
AK007214	0	0.00	
AK007216	0	0.00	
AK007218	3.741236	0.46	12.4%
AK007221	0.176609	0.12	66.8%
AK007223	0.4439562	0.10	23.6%
AK007237	0.06925846	0.05	68.0%
AK007238	0	0.00	
AK007249	0.12061702	0.05	40.3%
AK007253	0.01332068	0.03	223.6%
AK007255	0.4090506	0.11	27.4%
AK007260	0	0.00	
AK007274	0	0.00	
AK007280	0.00998208	0.02	223.6%
AK007290	0	0.00	
AK007305	0	0.00	
AK007312	0	0.00	
AK007349	0	0.00	
AK007351	43.76992	4.94	11.3%
AK007373	4.448104	1.00	22.5%
AK007376	0	0.00	
AK007416	0.02410514	0.02	96.0%
AK007434	0	0.00	
AK007436	1.111265	0.37	33.1%
AK007459	5.7933	0.73	12.6%
AK007460	0.16305134	0.15	90.2%
AK007490	2.1272412	1.33	62.6%
AK007531	0	0.00	
AK007545	0.02205462	0.02	102.0%
AK007571	0.3059576	0.11	36.6%
AK007586	0.2375086	0.11	46.3%
AK007597	35.28212	1.77	5.0%
AK007605	0.7058602	0.21	30.4%
AK007719	2.154426	0.23	10.9%
AK007756	5.99921	0.64	10.7%
AK007770	0.8929198	0.20	22.5%
AK007818	0	0.00	
AK007819	0.0091446	0.01	94.9%
AK007836	0.23118008	0.12	50.6%
AK007845	0	0.00	
AK007853	0.06314466	0.05	86.8%

AK007854	0	0.00	
AK007898	1.2346752	0.38	31.0%
AK007899	0.01061848	0.02	223.6%
AK007907	0.11658896	0.09	79.9%
AK007938	0.2337876	0.07	31.9%
AK007947	0	0.00	
AK007971	1.401274	0.17	12.1%
AK007998	1.771208	0.22	12.5%
AK008014	1.342682	0.16	11.9%
AK008044	0.4162626	0.08	18.1%
AK008073	0	0.00	
AK008077	1.050947	0.36	34.1%
AK008166	0.0313503	0.04	142.8%
AK008222	0	0.00	
AK008237	1.713702	0.35	20.5%
AK008288	0.0224748	0.05	223.6%
AK008334	0.02380406	0.02	93.9%
AK008341	1.0598242	0.60	56.8%
AK008384	0.01856478	0.03	141.7%
AK008417	15.731	1.69	10.7%
AK008432	0	0.00	
AK008458	3.054302	0.78	25.5%
AK008482	0	0.00	
AK008488	0	0.00	
AK008496	0	0.00	
AK008545	0.15077034	0.14	90.8%
AK008560	0	0.00	
AK008572	0.0168954	0.02	137.0%
AK008586	16.5436	0.81	4.9%
AK008665	10.037228	1.30	12.9%
AK008705	1.250158	0.14	10.9%
AK008724	47.79814	1.08	2.3%
AK008755	0.00744388	0.01	137.8%
AK008767	0	0.00	
AK008813	6.009458	0.36	5.9%
AK008826	0	0.00	
AK008833	0.2992024	0.13	43.1%
AK008852	1.5867074	0.99	62.3%
AK008856	8.683862	0.87	10.0%
AK008866	5.729022	0.57	10.0%
AK008907	0.146174	0.10	65.2%
AK008921	0	0.00	
AK008969	0.4399318	0.12	27.8%
AK009046	6.380438	1.34	20.9%
AK009064	0.1354295	0.10	71.7%
AK009067	0	0.00	
AK009121	0.14582614	0.04	26.6%

AK009175	12.09162	0.63	5.2%
AK009188	0.00649388	0.01	223.6%
AK009198	1.728074	0.27	15.8%
AK009214	0.0205986	0.05	223.6%
AK009335	0.2603056	0.07	27.4%
AK009348	0.7592794	0.41	53.5%
AK009351	0.05192044	0.05	99.6%
AK009365	65.0618	4.42	6.8%
AK009368	0.15047342	0.08	56.0%
AK009383	0	0.00	
AK009395	4.640192	0.20	4.4%
AK009597	0.05035236	0.04	76.6%
AK009603	3.341024	0.60	17.8%
AK009689	0	0.00	
AK009724	0.11706408	0.08	70.5%
AK009784	0.02105468	0.02	93.9%
AK009785	0.1484154	0.04	29.6%
AK009788	18.17098	0.62	3.4%
AK009813	0.251174	0.14	57.6%
AK009829	1.5030836	0.96	64.1%
AK009869	0.35117402	0.21	60.1%
AK009887	2.971344	0.75	25.3%
AK009897	0	0.00	
AK009900	0.0063127	0.01	223.6%
AK009977	0.11278766	0.02	21.0%
AK010044	0.009366698	0.00	45.6%
AK010122	0	0.00	
AK010129	0.2762536	0.12	44.2%
AK010195	0.0517045	0.04	82.8%
AK010245	0.02867088	0.03	93.8%
AK010366	4.945212	0.41	8.3%
AK010368	0.8830848	0.23	26.5%
AK010390	0.0370195	0.03	68.3%
AK010403	4.029412	0.69	17.2%
AK010406	0.03945194	0.03	80.5%
AK010427	41.14768	2.00	4.8%
AK010542	0.02349716	0.04	156.1%
AK010638	0.04471724	0.01	26.0%
AK010729	0.1972416	0.14	70.0%
AK010767	0	0.00	
AK010770	0.03450018	0.02	62.7%
AK010774	0.04433984	0.05	107.4%
AK010793	1.0673714	0.17	15.5%
AK010824	0.04782124	0.03	61.0%
AK010870	1.72215	0.15	8.9%
AK010894	0.1275062	0.10	77.9%
AK010897	0	0.00	

AK011021	2.182706	0.49	22.5%
AK011120	0.324011	0.06	19.2%
AK011205	15.14762	0.87	5.7%
AK011222	0.2906354	0.04	15.3%
AK011283	3.787042	0.32	8.5%
AK011326	0.3083408	0.11	35.2%
AK011408	0.0504394	0.11	223.6%
AK011444	0.5866234	0.16	27.5%
AK011460	7.547172	0.82	10.9%
AK011479	0.3661176	0.07	18.2%
AK011482	0.0281397	0.03	94.9%
AK011498	3.780422	0.39	10.4%
AK011524	3.195276	0.21	6.5%
AK011549	3.838858	1.65	42.9%
AK011568	0.1332114	0.03	23.1%
AK011682	0.8729026	0.39	44.8%
AK011684	0.4312988	0.25	56.9%
AK011693	0.16286664	0.04	24.9%
AK011865	12.31404	0.98	8.0%
AK011877	0.0053195	0.01	223.6%
AK011879	0.07355466	0.03	42.0%
AK011885	5.638432	0.37	6.6%
AK011897	0.05439756	0.02	40.6%
AK011913	0.327993	0.04	11.3%
AK011997	3.285714	0.62	18.9%
AK012007	0.1432332	0.02	14.7%
AK012035	3.385896	0.31	9.1%
AK012135	0.5410746	0.11	19.7%
AK012142	0.869138	0.18	20.4%
AK012157	0.03186798	0.02	72.3%
AK012214	0.1279829	0.07	54.5%
AK012226	0.028176224	0.03	95.1%
AK012238	0.0775943	0.05	66.7%
AK012278	2.826688	0.41	14.6%
AK012325	1.821456	0.49	27.1%
AK012381	0.01385506	0.01	96.3%
AK012387	0.3891968	0.18	45.3%
AK012418	0.07456938	0.02	25.6%
AK012506	1.495026	0.13	9.0%
AK012546	16.0201	1.22	7.6%
AK012547	0.4792142	0.18	37.0%
AK012572	2.404332	0.91	37.8%
AK012595	7.760766	0.69	8.9%
AK012623	0.32064178	0.26	81.4%
AK012661	0	0.00	
AK012713	7.456488	1.11	14.9%
AK012776	7.02422	0.44	6.2%

AK012841	3.290284	0.29	8.7%
AK012857	11.5646	0.65	5.6%
AK012891	0.3703064	0.18	48.2%
AK012899	0.09183414	0.08	89.3%
AK012900	0.14096512	0.08	55.5%
AK012955	0.095434024	0.07	71.8%
AK012972	0.07581832	0.04	56.3%
AK013046	0.4149068	0.14	34.2%
AK013162	10.9127	2.06	18.9%
AK013187	0.1345872	0.10	72.1%
AK013191	0.09871166	0.04	43.9%
AK013290	0.06320578	0.01	20.3%
AK013293	0.01178472	0.03	223.6%
AK013308	0.29661408	0.17	56.6%
AK013354	3.770206	0.56	14.9%
AK013356	1.550392	0.18	11.4%
AK013372	0	0.00	
AK013380	0.617432	0.15	23.9%
AK013453	2.239138	0.26	11.7%
AK013472	0.3899478	0.15	38.2%
AK013480	0.2551062	0.08	31.6%
AK013506	0.6860892	0.38	56.0%
AK013557	26.28158	0.89	3.4%
AK013564	1.31274	0.15	11.1%
AK013593	2.674796	0.53	19.8%
AK013627	0	0.00	
AK013632	0.1692752	0.04	21.3%
AK013680	0.12964186	0.16	121.2%
AK013687	0.09351608	0.06	65.5%
AK013712	0.09313576	0.02	26.3%
AK013794	2.399744	0.33	13.6%
AK013806	3.64521	0.55	15.0%
AK013818	0.3923076	0.16	40.4%
AK013876	0.8192554	0.18	21.4%
AK013883	9.48196	0.93	9.8%
AK013936	21.19714	1.41	6.7%
AK014028	0	0.00	
AK014032	0.38745398	0.53	136.1%
AK014089	1.324722	0.15	11.0%
AK014098	1.586708	0.16	10.1%
AK014119	0.19519352	0.17	84.8%
AK014147	0.2536644	0.07	29.1%
AK014177	0.38962368	0.53	135.7%
AK014209	0.9538932	0.26	27.2%
AK014224	2.607316	0.67	25.8%
AK014273	0.913195	0.30	33.0%
AK014378	2.79473	0.57	20.5%

AK014409	0.03274566	0.02	63.7%
AK014435	0.427738	0.16	36.9%
AK014460	0.006400318	0.01	152.9%
AK014513	0	0.00	
AK014573	0.01552434	0.02	139.4%
AK014626	0.00272224	0.01	223.6%
AK014646	19.12232	0.48	2.5%
AK014660	0.13756942	0.06	45.2%
AK014677	0	0.00	
AK014683	0.05240358	0.05	94.2%
AK014704	0.0134014	0.02	137.0%
AK014713	0	0.00	
AK014726	0.06269504	0.08	120.5%
AK014752	0.10413536	0.04	40.1%
AK014786	0	0.00	
AK014798	0.15151878	0.04	28.9%
AK014841	0	0.00	
AK014857	0	0.00	
AK014882	0.00416142	0.01	223.6%
AK014951	0.0048448	0.01	223.6%
AK014955	0.4299584	0.13	29.2%
AK014957	0	0.00	
AK014964	0.08950442	0.04	49.7%
AK014969	0	0.00	
AK014974	1.2507262	0.41	32.8%
AK014981	0.02697998	0.02	63.7%
AK014986	0.2636968	0.13	49.2%
AK014992	0	0.00	
AK014999	0	0.00	
AK015004	0.20227476	0.07	35.6%
AK015045	0	0.00	
AK015051	0.2441764	0.03	13.6%
AK015063	0.02184464	0.02	70.9%
AK015072	0	0.00	
AK015079	0	0.00	
AK015092	0.3226354	0.06	19.6%
AK015094	0	0.00	
AK015096	0.4543496	0.17	38.2%
AK015099	0	0.00	
AK015100	1.198368	0.11	9.6%
AK015105	0	0.00	
AK015107	0	0.00	
AK015118	0	0.00	
AK015136	1.772918	0.18	10.1%
AK015174	0.00846962	0.02	223.6%
AK015179	4.95259	0.21	4.2%
AK015183	0.6167518	0.24	39.4%

AK015188	0	0.00	
AK015191	0	0.00	
AK015198	0	0.00	
AK015200	0	0.00	
AK015203	0.00658056	0.01	223.6%
AK015208	0	0.00	
AK015214	0.19543162	0.16	83.8%
AK015219	0	0.00	
AK015225	1.0567354	0.34	31.9%
AK015227	0	0.00	
AK015236	0	0.00	
AK015257	0.02018032	0.03	147.7%
AK015270	1.0085474	0.15	14.5%
AK015277	0	0.00	
AK015279	0.00756734	0.01	137.0%
AK015284	0	0.00	
AK015289	0.0138837	0.01	105.2%
AK015306	0	0.00	
AK015316	0	0.00	
AK015322	0	0.00	
AK015324	0	0.00	
AK015336	0	0.00	
AK015341	0.01168408	0.02	142.4%
AK015347	0	0.00	
AK015360	0.03453652	0.03	75.4%
AK015363	0	0.00	
AK015373	0	0.00	
AK015376	0	0.00	
AK015378	0	0.00	
AK015379	0.01722954	0.02	139.1%
AK015384	0.09640236	0.03	28.5%
AK015392	0.5214254	0.09	17.9%
AK015395	0	0.00	
AK015404	0.01493892	0.02	161.9%
AK015408	0.8671878	0.21	24.6%
AK015409	0	0.00	
AK015413	0	0.00	
AK015421	0.041187414	0.05	112.5%
AK015423	0	0.00	
AK015428	0	0.00	
AK015429	0	0.00	
AK015444	0	0.00	
AK015457	0	0.00	
AK015458	0	0.00	
AK015463	0	0.00	
AK015470	0	0.00	
AK015475	0	0.00	

AK015482	0	0.00	
AK015488	0	0.00	
AK015492	0.15966252	0.06	40.3%
AK015493	0	0.00	
AK015504	0.2086954	0.07	31.7%
AK015518	0	0.00	
AK015520	0	0.00	
AK015526	0	0.00	
AK015528	0.02672624	0.03	110.3%
AK015529	0.0860024	0.11	124.5%
AK015544	21.75404	0.57	2.6%
AK015545	0.08547346	0.08	91.9%
AK015552	0.22555908	0.16	68.8%
AK015560	0.02986984	0.01	16.9%
AK015562	0.09064424	0.05	57.4%
AK015566	0.04282042	0.02	37.5%
AK015581	0	0.00	
AK015607	0	0.00	
AK015608	0.00397358	0.01	223.6%
AK015610	0.01317858	0.02	137.3%
AK015611	1.973522	0.28	14.4%
AK015615	0.01026652	0.01	109.5%
AK015628	0	0.00	
AK015651	0	0.00	
AK015657	0.08446654	0.05	58.1%
AK015664	0	0.00	
AK015665	0.508404	0.23	44.6%
AK015677	0	0.00	
AK015682	0.0549083	0.04	67.5%
AK015703	0.03391004	0.02	60.1%
AK015712	0.00618784	0.01	223.6%
AK015714	0.5015972	0.12	24.9%
AK015741	0.00368128	0.01	223.6%
AK015749	0	0.00	
AK015753	0.00276126	0.01	223.6%
AK015755	0.4107978	0.05	11.9%
AK015760	0	0.00	
AK015762	0	0.00	
AK015772	0	0.00	
AK015773	0.05603616	0.04	65.5%
AK015782	0.022179	0.02	97.4%
AK015792	0.852161	0.17	20.3%
AK015810	0	0.00	
AK015813	0	0.00	
AK015814	0.14730724	0.03	19.2%
AK015818	0	0.00	
AK015826	3.025458	0.25	8.3%

AK015844	0	0.00	
AK015877	0	0.00	
AK015891	0	0.00	
AK015892	0	0.00	
AK015893	0.06049018	0.03	57.7%
AK015900	0	0.00	
AK015904	0	0.00	
AK015907	0.09598566	0.05	50.5%
AK015914	0	0.00	
AK015937	0	0.00	
AK015940	0	0.00	
AK015944	0	0.00	
AK015950	0	0.00	
AK015953	0	0.00	
AK015955	0.080219506	0.10	121.1%
AK015956	0.04547876	0.05	108.8%
AK015958	0	0.00	
AK015967	0.2338259	0.15	63.6%
AK015969	0.3560678	0.23	65.8%
AK015999	0.3923682	0.09	23.7%
AK016002	0	0.00	
AK016007	0.08816764	0.02	17.7%
AK016016	0	0.00	
AK016027	0	0.00	
AK016028	0	0.00	
AK016032	0	0.00	
AK016040	0	0.00	
AK016046	0	0.00	
AK016054	0	0.00	
AK016061	0.12595778	0.05	42.5%
AK016071	0	0.00	
AK016077	0.08285492	0.05	58.4%
AK016085	0.3171546	0.12	36.8%
AK016097	0	0.00	
AK016100	0.00836758	0.02	223.6%
AK016120	0	0.00	
AK016128	0	0.00	
AK016129	0	0.00	
AK016131	0.01055888	0.02	223.6%
AK016150	0.03391016	0.06	174.9%
AK016162	0.03495896	0.01	20.2%
AK016163	0	0.00	
AK016172	0	0.00	
AK016177	0.07561938	0.03	42.9%
AK016178	0.01160156	0.02	137.2%
AK016179	0.01269474	0.02	141.2%
AK016185	16.41716	2.55	15.5%

AK016192	0.00965684	0.02	223.6%
AK016207	0	0.00	
AK016225	0.00532238	0.01	223.6%
AK016243	0	0.00	
AK016244	0	0.00	
AK016251	0.01127208	0.01	64.3%
AK016265	0.12974208	0.08	58.0%
AK016276	0.00632094	0.01	223.6%
AK016280	0	0.00	
AK016281	0	0.00	
AK016283	0	0.00	
AK016284	0.01557158	0.03	223.6%
AK016307	0.02690118	0.03	108.7%
AK016318	0	0.00	
AK016328	0.031586686	0.04	130.6%
AK016332	0	0.00	
AK016335	0.01213834	0.03	223.6%
AK016336	0.4437088	0.15	32.8%
AK016343	0.0211558	0.05	223.6%
AK016345	0	0.00	
AK016349	0	0.00	
AK016361	0	0.00	
AK016372	0	0.00	
AK016377	0.00971836	0.02	223.6%
AK016383	0	0.00	
AK016386	0	0.00	
AK016388	0.02021846	0.03	147.3%
AK016393	0	0.00	
AK016399	0	0.00	
AK016412	0	0.00	
AK016413	0	0.00	
AK016417	0.12940296	0.08	60.5%
AK016427	0.00594406	0.01	223.6%
AK016430	2.057906	0.26	12.6%
AK016444	0.03738322	0.03	67.5%
AK016452	0.1098288	0.12	113.2%
AK016496	0.09283962	0.04	41.1%
AK016511	0.06817442	0.05	67.7%
AK016513	0.062022	0.05	87.2%
AK016521	0.010079118	0.01	119.4%
AK016549	0.11350282	0.07	58.3%
AK016580	0.2450546	0.08	32.8%
AK016584	0.10781052	0.02	22.7%
AK016591	0.20207048	0.08	38.9%
AK016592	0.0025974	0.01	223.6%
AK016594	0	0.00	
AK016605	0	0.00	

AK016630	0.06646982	0.05	77.2%
AK016650	0	0.00	
AK016658	0	0.00	
AK016661	0.0272945	0.02	84.1%
AK016682	0.3425202	0.05	14.7%
AK016686	0	0.00	
AK016693	0.3230984	0.12	36.7%
AK016695	0.02325898	0.02	66.1%
AK016696	0	0.00	
AK016730	0.3709592	0.11	30.4%
AK016731	0	0.00	
AK016734	0	0.00	
AK016788	0.4338872	0.12	28.3%
AK016790	0	0.00	
AK016808	0	0.00	
AK016813	0.0035746	0.01	223.6%
AK016833	0	0.00	
AK016836	0	0.00	
AK016837	0.5731618	0.13	23.0%
AK016841	0.3138222	0.12	37.0%
AK016887	0.03570056	0.01	29.1%
AK016893	0.10145554	0.06	58.0%
AK016898	0	0.00	
AK016914	0	0.00	
AK016926	0	0.00	
AK016931	0	0.00	
AK016948	0	0.00	
AK016961	0	0.00	
AK017006	0	0.00	
AK017010	0.25838758	0.57	220.5%
AK017025	0.00527286	0.01	223.6%
AK017040	0.0337626	0.03	74.3%
AK017048	0	0.00	
AK017068	0	0.00	
AK017076	0.3843566	0.04	10.6%
AK017085	0.118587562	0.14	118.5%
AK017086	0	0.00	
AK017092	0.6579112	0.12	17.7%
AK017104	0	0.00	
AK017106	0	0.00	
AK017111	0	0.00	
AK017125	0.0050791	0.01	223.6%
AK017137	0.001557186	0.00	223.6%
AK017143	1.46763	0.12	8.2%
AK017144	1.123132	0.10	8.7%
AK017148	0	0.00	
AK017177	0.8895144	0.21	23.2%

AK017182	0.04343178	0.05	121.3%
AK017199	0	0.00	
AK017220	0.05574584	0.03	58.3%
AK017239	0.00438428	0.01	223.6%
AK017243	0.1844866	0.03	15.1%
AK017262	0.0572111	0.03	51.1%
AK017296	0.5164422	0.51	99.4%
AK017306	0.02554408	0.03	108.0%
AK017316	4.935332	0.50	10.1%
AK017331	0.04683084	0.01	28.8%
AK017337	0	0.00	
AK017339	0	0.00	
AK017349	0	0.00	
AK017352	0	0.00	
AK017368	0.01650772	0.02	138.6%
AK017409	0.1822412	0.04	19.7%
AK017430	0	0.00	
AK017440	123.004	1.13	0.9%
AK017459	0.5340442	0.17	32.4%
AK017500	1.1025622	0.29	26.4%
AK017534	0.1677026	0.05	28.1%
AK017586	0	0.00	
AK017620	0.00982002	0.02	223.6%
AK017634	0.29101798	0.16	53.6%
AK017645	6.931566	0.55	7.9%
AK017706	0.4776178	0.12	24.1%
AK017756	0.09252932	0.06	65.8%
AK017779	0.1797186	0.05	29.2%
AK017784	0.15225496	0.11	74.6%
AK017883	0.3895956	0.03	8.5%
AK017891	0	0.00	
AK017893	0.04165062	0.03	68.1%
AK017924	1.015489	0.15	14.9%
AK018014	0	0.00	
AK018025	0	0.00	
AK018074	0.3297538	0.11	33.9%
AK018104	34.70066	1.39	4.0%
AK018134	6.361296	0.46	7.2%
AK018156	0.9534742	0.14	14.6%
AK018164	13.09646	0.87	6.7%
AK018183	2.632136	0.45	17.1%
AK018214	0.335896	0.09	26.0%
AK018228	1.7716	0.23	12.8%
AK018302	0.01414362	0.02	142.1%
AK018309	2.322524	0.39	16.9%
AK018352	0	0.00	
AK018376	0.001334898	0.00	223.6%

AK018427	0.04332968	0.04	86.0%
AK018435	0.5464352	0.15	26.8%
AK018450	1.517262	0.26	17.0%
AK018484	0.00935026	0.01	137.4%
AK018490	1.3534888	0.36	26.3%
AK018513	0	0.00	
AK018584	9.158276	0.72	7.9%
AK018624	0.0086205	0.02	223.6%
AK018679	69.594	1.69	2.4%
AK018699	0	0.00	
AK018753	1960.482	249.50	12.7%
AK018772	9.61717	1.78	18.5%
AK018841	0	0.00	
AK018847	0	0.00	
AK018858	0	0.00	
AK018862	0.8494162	0.16	19.3%
AK018866	0	0.00	
AK018879	0	0.00	
AK018880	0	0.00	
AK018891	0	0.00	
AK018902	0	0.00	
AK018905	0	0.00	
AK018908	0	0.00	
AK018911	0.06464738	0.02	35.5%
AK018913	0.6397256	1.25	196.1%
AK018918	0.02975956	0.01	18.9%
AK018924	0.0970356	0.06	56.9%
AK018929	0.09706792	0.06	65.5%
AK018934	0	0.00	
AK018937	0.00927812	0.02	223.6%
AK018940	0	0.00	
AK018946	0	0.00	
AK018948	0	0.00	
AK018959	0	0.00	
AK018965	0	0.00	
AK018967	0.6208894	0.19	29.9%
AK018968	0	0.00	
AK018972	0	0.00	
AK018974	0	0.00	
AK018976	0	0.00	
AK018977	0	0.00	
AK018979	0	0.00	
AK018984	0.01728268	0.02	140.6%
AK018988	0.09769412	0.02	23.3%
AK018990	0.02399384	0.03	105.9%
AK018991	0	0.00	
AK018993	0.1552222	0.04	27.3%

AK018996	0.22557992	0.21	91.4%
AK019003	0.937083	0.22	23.9%
AK019015	0.456104	0.25	55.2%
AK019031	0.14210828	0.12	86.2%
AK019036	0	0.00	
AK019053	0.2200467	0.20	88.7%
AK019057	0.01491666	0.03	223.6%
AK019067	0.6549338	0.27	40.9%
AK019072	0	0.00	
AK019082	0.473133	0.21	44.9%
AK019083	34.11176	4.24	12.4%
AK019114	1.63669	0.41	25.3%
AK019124	0	0.00	
AK019125	0	0.00	
AK019137	0.0459237	0.01	29.3%
AK019154	9.851316	0.74	7.6%
AK019229	1.1955924	0.22	18.7%
AK019237	0.6311368	0.20	31.2%
AK019250	0.0426763	0.03	69.0%
AK019262	8.613756	0.80	9.2%
AK019272	0.07189352	0.06	80.2%
AK019286	2.998118	0.48	16.0%
AK019290	0.2464866	0.23	92.5%
AK019332	0.037885	0.04	94.7%
AK019339	1.07145	0.25	23.3%
AK019348	0.04273664	0.04	92.8%
AK019365	0.9243412	0.43	46.7%
AK019366	0.3123668	0.13	41.1%
AK019375	0.02273968	0.03	138.2%
AK019388	60.0128	1.52	2.5%
AK019420	41.58298	2.61	6.3%
AK019435	0	0.00	
AK019455	0.001432624	0.00	223.6%
AK019514	0	0.00	
AK019536	2.986098	0.28	9.5%
AK019538	0	0.00	
AK019541	0.01495046	0.02	138.1%
AK019561	0	0.00	
AK019563	0	0.00	
AK019573	0	0.00	
AK019585	0	0.00	
AK019592	0	0.00	
AK019593	0	0.00	
AK019596	0	0.00	
AK019598	0	0.00	
AK019601	0	0.00	
AK019604	0	0.00	

AK019614	0.01723884	0.04	223.6%
AK019620	0	0.00	
AK019623	0.01732406	0.04	223.6%
AK019626	0	0.00	
AK019628	0	0.00	
AK019629	0	0.00	
AK019630	0.11965994	0.12	99.9%
AK019631	0.1852524	0.17	90.9%
AK019641	0	0.00	
AK019643	0	0.00	
AK019657	0	0.00	
AK019662	0	0.00	
AK019664	0.0750473	0.04	57.9%
AK019667	0	0.00	
AK019672	0	0.00	
AK019679	0.03312614	0.02	52.0%
AK019680	0.01949702	0.04	223.6%
AK019688	0	0.00	
AK019690	0.8705534	0.27	31.2%
AK019691	0	0.00	
AK019697	0	0.00	
AK019698	0	0.00	
AK019699	0	0.00	
AK019701	0	0.00	
AK019704	0	0.00	
AK019709	0.04651782	0.04	78.7%
AK019710	0	0.00	
AK019711	0	0.00	
AK019719	0.01423042	0.02	139.1%
AK019722	0.0549652	0.03	50.3%
AK019723	0	0.00	
AK019729	0	0.00	
AK019730	0.4439416	0.16	36.2%
AK019731	0	0.00	
AK019734	0	0.00	
AK019737	0	0.00	
AK019738	0.12476574	0.11	88.2%
AK019743	0.779906	0.13	17.2%
AK019745	0	0.00	
AK019751	0.00819354	0.02	223.6%
AK019752	0.04599244	0.02	44.5%
AK019757	0.27437242	0.20	72.0%
AK019772	0.094352	0.09	92.9%
AK019773	0	0.00	
AK019774	0	0.00	
AK019781	0	0.00	
AK019789	0	0.00	

AK019790	0	0.00	
AK019792	0	0.00	
AK019796	0	0.00	
AK019797	0.00321622	0.01	223.6%
AK019798	0	0.00	
AK019801	0	0.00	
AK019804	0	0.00	
AK019810	0	0.00	
AK019812	0	0.00	
AK019818	0	0.00	
AK019824	0.3233492	0.06	18.8%
AK019836	0	0.00	
AK019838	0	0.00	
AK019844	0	0.00	
AK019889	0	0.00	
AK019935	0.0399504	0.04	93.6%
AK019949	0	0.00	
AK019984	0.002858382	0.00	167.2%
AK020025	0.07735218	0.14	182.6%
AK020043	0	0.00	
AK020054	0.07274648	0.06	80.2%
AK020061	0	0.00	
AK020081	0	0.00	
AK020087	0.0997162	0.06	64.1%
AK020089	0.2543246	0.10	37.9%
AK020095	12.08942	0.98	8.1%
AK020100	0.4904054	0.04	9.0%
AK020101	0.0793087	0.02	27.5%
AK020188	0	0.00	
AK020190	0	0.00	
AK020205	0.01979522	0.03	137.5%
AK020213	0.02327403	0.02	72.9%
AK020235	0.01336338	0.02	141.6%
AK020236	0	0.00	
AK020256	0	0.00	
AK020276	0	0.00	
AK020309	0.00873042	0.02	223.6%
AK020320	0.04707872	0.05	97.2%
AK020345	0	0.00	
AK020351	0.01160554	0.03	223.6%
AK020361	0.1540217	0.12	80.4%
AK020388	0.11142922	0.02	17.5%
AK020398	2.524516	0.34	13.3%
AK020406	0.3391354	0.27	80.4%
AK020462	0.8842122	0.23	26.0%
AK020476	0.00291306	0.01	223.6%
AK020485	0	0.00	

AK020496	0.04722828	0.03	70.1%
AK020502	1.1207458	0.16	14.3%
AK020506	0.00427976	0.01	223.6%
AK020517	2.367752	0.26	11.2%
AK020535	0	0.00	
AK020549	0.0196892	0.03	141.5%
AK020556	0	0.00	
AK020565	0.00516502	0.01	223.6%
AK020577	0	0.00	
AK020588	0.0043194	0.01	223.6%
AK020616	6.295096	0.23	3.6%
AK020619	0.4469288	0.08	18.9%
AK020628	0.3557986	0.16	44.3%
AK020645	0.14857076	0.06	37.5%
AK020680	0.00908362	0.02	223.6%
AK020688	10.095802	0.66	6.5%
AK020705	0	0.00	
AK020722	0.9071918	0.20	22.0%
AK020728	1.848086	0.35	18.9%
AK020764	0	0.00	
AK020767	0.2615124	0.11	40.9%
AK020768	0.009952822	0.01	72.0%
AK020793	0.00914204	0.01	137.3%
AK020795	0.002604244	0.00	137.3%
AK020812	5.37196	0.74	13.8%
AK020826	0.02640063	0.02	84.1%
AK020829	0	0.00	
AK020834	0	0.00	
AK020835	38.03246	2.85	7.5%
AK020854	0.00880946	0.02	223.6%
AK020861	0	0.00	
AK020867	0.1673168	0.03	16.1%
AK020885	0	0.00	
AK020898	0.07415488	0.04	48.6%
AK020914	0.00569936	0.01	223.6%
AK020919	0.05941206	0.05	80.0%
AK020930	0.0083282	0.02	223.6%
AK020948	0.0355788	0.03	94.5%
AK020962	0	0.00	
AK021003	0.28188392	0.21	73.6%
AK021004	0.09730806	0.03	26.0%
AK021007	1.1805808	0.39	32.7%
AK021028	0.82625606	0.69	83.4%
AK021029	0.1105496	0.01	7.6%
AK021038	13.65512	1.39	10.2%
AK021048	0.0215986	0.05	223.6%
AK021075	0	0.00	

AK021093	0	0.00	
AK021113	1.561276	0.16	10.6%
AK021127	0.14785112	0.08	56.8%
AK021128	0.01017104	0.02	223.6%
AK021143	4.15399	0.41	9.9%
AK021144	5.586156	0.15	2.7%
AK021178	0	0.00	
AK021191	0	0.00	
AK021192	0	0.00	
AK021197	1.74336	0.22	12.6%
AK021198	0	0.00	
AK021199	0.2995238	0.06	20.0%
AK021214	0.01634824	0.02	137.0%
AK021218	0	0.00	
AK021231	0.2069152	0.12	56.9%
AK021232	0.02634936	0.01	42.5%
AK021262	0.25634866	0.13	51.8%
AK021280	15.26008	1.00	6.6%
AK021297	0.3850846	0.13	34.3%
AK021299	0.179399	0.05	27.5%
AK021325	1.1546714	0.25	21.9%
AK021336	0.07792966	0.06	74.4%
AK021342	0.1713676	0.06	35.7%
AK021368	1.1759444	0.27	23.4%
AK021371	0.127156765	0.15	114.4%
AK021377	0.02998508	0.02	62.8%
AK027905	0	0.00	
AK027928	0.01480256	0.02	137.8%
AK027935	0.09456086	0.18	189.8%
AK027941	45.10856	6.53	14.5%
AK027957	1.1682388	0.21	18.2%
AK027968	0	0.00	
AK027991	0.13621266	0.05	35.0%
AK028005	0.0307926	0.02	59.1%
AK028012	0.00845908	0.02	223.6%
AK028033	0	0.00	
AK028077	0.012313136	0.01	79.2%
AK028088	0	0.00	
AK028089	0	0.00	
AK028094	0.00975676	0.02	223.6%
AK028111	0	0.00	
AK028130	0.02171768	0.03	139.2%
AK028224	1.1271024	0.47	41.9%
AK028243	0.3150584	0.14	44.0%
AK028383	4.885826	0.70	14.3%
AK028394	0.77226028	0.70	90.1%
AK028422	0	0.00	

AK028639	0.0024025	0.01	223.6%
AK028650	0.09828862	0.03	32.3%
AK028711	0	0.00	
AK028767	2.48498	0.44	17.6%
AK028773	0.010416078	0.01	77.1%
AK028782	3.540312	0.78	22.2%
AK028798	0.00224912	0.01	223.6%
AK028852	0.1876148	0.04	20.9%
AK028920	0.4655524	0.06	13.2%
AK029023	0.0147948	0.02	142.8%
AK029086	0.00511142	0.01	223.6%
AK029100	1.48399	0.18	11.9%
AK029153	0.0028624	0.01	223.6%
AK029171	0.3305748	0.06	19.4%
AK029258	0.08072696	0.03	33.1%
AK029312	0	0.00	
AK029359	1.744152	0.39	22.6%
AK029385	0	0.00	
AK029402	0.064216124	0.07	113.4%
AK029404	0	0.00	
AK029407	0.0105272	0.01	137.4%
AK029418	0.04998758	0.05	99.4%
AK029443	0.2515612	0.10	40.1%
AK029473	0.7283686	0.16	22.1%
AK029503	0.00259354	0.01	223.6%
AK029536	0.0595853	0.02	31.9%
AK029560	0	0.00	
AK029576	9.232144	0.41	4.5%
AK029587	0	0.00	
AK029591	0.022915056	0.01	56.3%
AK029596	0	0.00	
AK029598	0	0.00	
AK029599	0	0.00	
AK029605	0	0.00	
AK029614	0	0.00	
AK029620	0	0.00	
AK029624	0	0.00	
AK029630	0.06079498	0.04	61.0%
AK029633	0.11274554	0.03	25.9%
AK029637	0.07832216	0.08	103.4%
AK029638	0.0097819	0.02	223.6%
AK029641	0	0.00	
AK029643	0	0.00	
AK029646	0	0.00	
AK029660	0	0.00	
AK029663	0.0688578	0.10	140.8%
AK029672	0	0.00	

AK029692	0	0.00	
AK029726	0.11058572	0.01	12.9%
AK029746	0.09203118	0.04	40.3%
AK029768	0	0.00	
AK029781	0	0.00	
AK029792	0	0.00	
AK029795	0	0.00	
AK029796	0.11228204	0.08	67.3%
AK029831	4.890656	2.03	41.5%
AK029834	0	0.00	
AK029857	0.331561	0.02	7.1%
AK029860	0.3113868	0.05	15.4%
AK029875	0.323803	0.07	21.2%
AK029878	0.735237	0.11	15.1%
AK029949	0	0.00	
AK029950	0.174372	0.02	10.9%
AK029956	0.18150366	0.11	61.1%
AK029958	0	0.00	
AK029959	0.9008998	0.12	13.0%
AK030052	0	0.00	
AK030117	0.0099589	0.01	108.8%
AK030127	0.014703826	0.01	72.6%
AK030128	0.02864736	0.02	70.9%
AK030154	0	0.00	
AK030183	0	0.00	
AK030206	0.3152494	0.09	29.1%
AK030230	0.01868528	0.01	47.2%
AK030236	0.0292014	0.01	19.7%
AK030238	0	0.00	
AK030251	0.5407236	0.25	46.1%
AK030258	0.004526378	0.01	139.3%
AK030263	0	0.00	
AK030268	0	0.00	
AK030275	51.49292	3.88	7.5%
AK030281	0.03241268	0.03	85.1%
AK030285	0.2610564	0.08	30.0%
AK030357	0.7756914	0.08	10.3%
AK030361	0.6985688	0.23	33.3%
AK030376	0.02722268	0.01	30.1%
AK030448	0.06290512	0.04	66.8%
AK030453	0.273442	0.06	22.5%
AK030468	0.0323408	0.04	137.0%
AK030539	0.9797522	0.23	23.8%
AK030561	0	0.00	
AK030600	4.369986	0.28	6.4%
AK030646	3.752922	0.24	6.4%
AK030763	2.069196	0.32	15.6%

AK030770	0.00973491	0.01	138.5%
AK030777	0.6097054	0.17	27.5%
AK030803	0	0.00	
AK030886	0.209718	0.06	26.6%
AK030906	0.016923	0.01	69.5%
AK030918	0.00685558	0.00	48.8%
AK030920	0.1720366	0.04	25.3%
AK030939	0.2457306	0.08	32.7%
AK030946	2.243776	0.48	21.3%
AK030979	0.6717398	0.11	16.0%
AK031042	0.191803	0.05	24.1%
AK031097	14.86764	0.29	1.9%
AK031134	0.9306466	0.12	12.8%
AK031165	0.4430444	0.19	42.3%
AK031200	1.579106	0.40	25.4%
AK031204	1.921058	0.27	13.8%
AK031221	0.13678574	0.06	45.1%
AK031234	2.347394	0.53	22.5%
AK031278	0.7544806	0.11	14.2%
AK031300	0.02908402	0.03	87.4%
AK031301	0.163849	0.02	14.0%
AK031332	0.07223226	0.01	7.0%
AK031437	0.04388642	0.02	42.3%
AK031460	0.10807606	0.05	47.4%
AK031463	0.06029754	0.03	57.1%
AK031468	0.2368794	0.04	15.7%
AK031485	0.0295024	0.02	54.5%
AK031498	0.4167444	0.06	13.5%
AK031500	0.223298	0.08	35.5%
AK031561	0.06918548	0.02	28.4%
AK031641	0.22625596	0.09	40.3%
AK031668	0.07534578	0.02	28.0%
AK031770	0.03614252	0.02	57.0%
AK031816	6.464984	0.13	2.0%
AK031819	10.526302	0.87	8.3%
AK031828	0	0.00	
AK031861	0.00548326	0.01	223.6%
AK031871	13.05904	0.61	4.7%
AK031919	0.0168995	0.00	22.1%
AK031964	16.62732	1.50	9.0%
AK032024	1.402548	0.16	11.1%
AK032118	0	0.00	
AK032180	0.001018468	0.00	223.6%
AK032194	0.2880702	0.06	21.3%
AK032255	0.671466	0.26	38.3%
AK032343	1.566724	0.30	18.9%
AK032387	0.5569132	0.02	2.8%

AK032401	12.75622	1.26	9.9%
AK032457	0.2257814	0.08	34.0%
AK032537	0.00101083	0.00	223.6%
AK032654	0.001152814	0.00	223.6%
AK032662	0.01774284	0.00	14.3%
AK032746	0.7857636	0.04	5.1%
AK032763	0	0.00	
AK032863	0.0780964	0.03	40.4%
AK032919	0.04386678	0.02	51.9%
AK033000	0	0.00	
AK033041	0	0.00	
AK033049	0.05004966	0.02	30.5%
AK033051	0	0.00	
AK033058	0	0.00	
AK033061	0.00402276	0.01	223.6%
AK033064	0	0.00	
AK033069	0.09454296	0.03	31.6%
AK033134	0.16586386	0.13	79.0%
AK033147	0.22719034	0.35	153.6%
AK033170	0	0.00	
AK033180	0.012102496	0.02	133.1%
AK033206	0.5858726	0.12	19.8%
AK033210	0.03065324	0.02	72.0%
AK033222	0.12836834	0.07	51.5%
AK033245	0.1595914	0.02	13.1%
AK033378	0	0.00	
AK033426	0.08641576	0.02	28.4%
AK033454	0.01749366	0.00	28.5%
AK033508	0.00491688	0.01	140.8%
AK033525	0.2595312	0.19	72.3%
AK033611	0.0939821	0.01	14.8%
AK033649	12.92054	0.41	3.1%
AK033706	0.001969118	0.00	223.6%
AK033717	0	0.00	
AK033743	0	0.00	
AK033745	0	0.00	
AK033794	9.203476	0.86	9.3%
AK033819	0.21948892	0.28	127.9%
AK033827	0.05577212	0.02	33.1%
AK033854	1.280864	0.12	9.5%
AK034016	0.4142438	0.17	40.4%
AK034027	0.001636092	0.00	223.6%
AK034052	4.344366	0.34	7.8%
AK034128	0.2031632	0.08	41.4%
AK034141	17.04552	0.81	4.8%
AK034149	0.1359736	0.04	27.5%
AK034159	0.013566004	0.02	140.7%

AK034200	0.408549	0.09	21.4%
AK034282	0.2994704	0.06	20.5%
AK034302	0.4647644	0.15	32.7%
AK034341	0.09732548	0.04	43.8%
AK034417	0	0.00	
AK034420	0.1153962	0.01	6.8%
AK034421	0.00392364	0.01	223.6%
AK034488	0	0.00	
AK034502	0.421662	0.07	16.2%
AK034519	0.028524442	0.02	53.2%
AK034539	0	0.00	
AK034569	3.209858	0.39	12.2%
AK034698	2.060288	0.22	10.8%
AK034716	4.305456	0.15	3.6%
AK034766	0.04458954	0.03	57.4%
AK034784	0.290419	0.06	22.1%
AK035006	0	0.00	
AK035031	0.0266944	0.05	200.8%
AK035037	20.46208	0.97	4.8%
AK035085	0.01899102	0.02	109.7%
AK035112	0.23905106	0.18	73.6%
AK035124	0	0.00	
AK035211	0	0.00	
AK035229	0.1506848	0.03	18.1%
AK035302	16.80294	0.98	5.8%
AK035331	0.01285564	0.01	94.5%
AK035353	0.0162946	0.00	22.9%
AK035379	0.00227056	0.01	223.6%
AK035387	0.11302092	0.03	29.5%
AK035406	0.01127272	0.02	137.0%
AK035444	0	0.00	
AK035460	0.1917398	0.02	11.5%
AK035466	0.3098126	0.06	19.3%
AK035515	1.5970014	0.54	33.9%
AK035520	0.9073714	0.15	17.0%
AK035525	0.3408584	0.18	54.0%
AK035573	0	0.00	
AK035596	0.00252712	0.01	223.6%
AK035625	0	0.00	
AK035645	27.241	1.63	6.0%
AK035669	0.004772454	0.00	91.3%
AK035687	0.0619089	0.03	45.3%
AK035706	0.0037022	0.01	223.6%
AK035711	0.00664466	0.01	145.2%
AK035732	0.012048852	0.00	33.1%
AK035738	0	0.00	
AK035742	0.2728036	0.12	42.4%

AK035754	0.342639	0.07	20.3%
AK035761	0.05260608	0.04	75.0%
AK035774	0.753724	0.17	23.1%
AK035785	0.209431	0.06	28.7%
AK035829	0.0254179	0.03	111.0%
AK035873	0.02627814	0.01	37.4%
AK035875	0.02095932	0.02	109.3%
AK035901	0.02553058	0.00	13.0%
AK035927	0.02035844	0.01	29.5%
AK035977	0.006824936	0.01	95.7%
AK035979	0.16797934	0.08	45.6%
AK035982	0.9717234	0.06	6.3%
AK035994	0.03148902	0.02	59.0%
AK036020	0.9348988	0.19	20.7%
AK036033	0.001372226	0.00	223.6%
AK036093	0.03921234	0.03	86.4%
AK036194	0.0235446	0.02	103.9%
AK036227	1.7431724	1.08	62.2%
AK036288	0.9544	0.17	17.4%
AK036289	14.5451	1.23	8.4%
AK036307	0.25382984	0.41	162.5%
AK036329	0.1264936	0.03	26.1%
AK036369	3.084468	0.14	4.6%
AK036371	0.07936698	0.03	40.8%
AK036396	0.02337202	0.03	149.7%
AK036422	3.122138	0.36	11.6%
AK036450	0	0.00	
AK036462	0.001668586	0.00	223.6%
AK036466	0.00984322	0.01	138.3%
AK036470	0.01125098	0.01	58.2%
AK036494	0	0.00	
AK036535	0.00398964	0.01	223.6%
AK036547	0.00949844	0.02	223.6%
AK036551	0.008782224	0.01	58.0%
AK036614	1.794404	0.18	10.0%
AK036632	0.04612522	0.06	135.3%
AK036683	0	0.00	
AK036693	0.5072232	0.17	33.8%
AK036733	0.07135634	0.02	30.4%
AK036749	0.00365832	0.01	223.6%
AK036854	0.00748698	0.01	138.1%
AK036897	0.005248044	0.00	94.0%
AK036949	0.05454646	0.05	89.4%
AK036974	0.13332228	0.07	54.3%
AK036982	0.017126	0.02	143.3%
AK037026	0.02097236	0.01	30.0%
AK037056	0.00973904	0.01	93.6%

AK037070	1.279682	0.18	14.2%
AK037090	0.3758316	0.08	21.9%
AK037113	1.53537944	0.94	61.5%
AK037155	0.2218346	0.05	24.6%
AK037159	0.4728098	0.05	11.5%
AK037193	0.1063029	0.06	56.5%
AK037260	0.228466	0.14	59.8%
AK037308	0.001911562	0.00	223.6%
AK037351	0.09526768	0.08	83.6%
AK037419	0	0.00	
AK037512	0	0.00	
AK037556	0.01813688	0.01	29.0%
AK037571	0	0.00	
AK037623	0	0.00	
AK037770	0.05366802	0.04	72.4%
AK037830	0.6430266	0.13	20.7%
AK037841	0.14486834	0.07	46.7%
AK037999	0.2122162	0.09	44.8%
AK038021	0.5063684	0.16	31.3%
AK038079	0.4200132	0.10	23.5%
AK038087	0	0.00	
AK038115	0.05380609	0.06	103.7%
AK038197	0	0.00	
AK038272	0.010324438	0.01	110.9%
AK038306	0.5639144	0.06	10.4%
AK038337	0.00484124	0.01	223.6%
AK038386	0.00458904	0.01	223.6%
AK038391	0	0.00	
AK038423	0.4570726	0.15	32.9%
AK038428	0	0.00	
AK038525	0.5131492	0.11	21.5%
AK038554	0.001821336	0.00	223.6%
AK038589	0	0.00	
AK038598	0.5995014	0.08	13.6%
AK038602	0.7214886	0.30	41.7%
AK038603	0.4929628	0.07	13.8%
AK038605	0.0386336	0.02	64.6%
AK038606	0	0.00	
AK038610	0	0.00	
AK038627	0.0879793	0.08	92.4%
AK038632	0.5246862	0.13	24.9%
AK038649	13.74692	0.81	5.9%
AK038653	0.0267091	0.02	68.2%
AK038665	0.02828278	0.02	73.1%
AK038693	0	0.00	
AK038711	0	0.00	
AK038726	7.652246	3.06	40.0%

AK038730	0	0.00	
AK038731	0.0433158	0.03	65.1%
AK038736	0.14632188	0.06	38.7%
AK038749	0.2597806	0.06	24.8%
AK038767	0.03480666	0.05	136.9%
AK038784	14.50528	0.90	6.2%
AK038791	0.1916332	0.03	15.4%
AK038816	0.06491628	0.04	66.4%
AK038818	0.09822498	0.03	28.5%
AK038827	0.13132804	0.08	61.8%
AK038843	0.001133632	0.00	223.6%
AK038880	0	0.00	
AK038883	1.2105382	0.34	28.2%
AK038885	0.2384498	0.08	31.7%
AK038886	0.21586982	0.12	53.8%
AK038918	25.16766	1.45	5.8%
AK038933	0.10008908	0.04	44.6%
AK038969	0.6187906	0.40	65.4%
AK038985	0.08993624	0.06	62.4%
AK038992	0.4331282	0.08	19.6%
AK038993	0.05087676	0.01	23.1%
AK039009	0.02415896	0.03	112.5%
AK039014	0.0601912	0.06	93.5%
AK039017	0.08601692	0.04	44.3%
AK039020	0.09310808	0.05	56.8%
AK039023	0.11318182	0.05	39.9%
AK039030	0.3186296	0.08	24.5%
AK039036	0	0.00	
AK039053	0.6194474	0.17	28.0%
AK039056	0.6530786	0.21	32.8%
AK039060	0.02208338	0.02	92.5%
AK039063	0.9603432	0.20	20.6%
AK039065	0.09246372	0.06	68.7%
AK039090	0.04239906	0.03	74.0%
AK039091	0.1275136	0.06	44.7%
AK039104	0.1331596	0.02	13.1%
AK039110	0.05321144	0.02	45.3%
AK039113	4.561484	0.67	14.7%
AK039117	11.27808	0.43	3.8%
AK039118	0.0381654	0.03	79.3%
AK039121	0.8745924	0.20	22.7%
AK039125	0.0060443	0.01	223.6%
AK039132	14.19386	1.47	10.4%
AK039140	0.25307106	0.48	188.7%
AK039159	0.6466358	0.15	22.9%
AK039238	0.01054616	0.01	93.9%
AK039254	24.0485	0.65	2.7%

AK039256	0.17925338	0.09	47.6%
AK039268	0.4136692	0.17	40.5%
AK039269	0	0.00	
AK039272	0.08471534	0.06	74.7%
AK039284	0.231998	0.06	27.9%
AK039340	0.09296874	0.02	23.1%
AK039361	0.04312286	0.04	93.0%
AK039362	0	0.00	
AK039372	0.2360394	0.09	39.5%
AK039373	0.00964362	0.01	137.7%
AK039376	0.05436222	0.03	52.6%
AK039382	0.09567324	0.04	44.8%
AK039403	0.943969	0.19	19.9%
AK039412	0	0.00	
AK039417	0.7016102	0.14	19.3%
AK039419	0	0.00	
AK039421	0	0.00	
AK039436	0.07138512	0.03	39.7%
AK039480	0.23631964	0.14	59.0%
AK039487	0	0.00	
AK039494	6.59573	0.49	7.4%
AK039495	0.4104702	0.12	28.3%
AK039498	0.0102596	0.01	91.8%
AK039500	0	0.00	
AK039511	0.05302672	0.05	95.3%
AK039536	1.844664	0.56	30.3%
AK039557	0.0571003	0.06	109.4%
AK039574	1.156608	0.40	34.8%
AK039577	2.00955	0.45	22.4%
AK039589	0	0.00	
AK039595	0	0.00	
AK039596	0.5235206	0.07	12.9%
AK039607	4.398098	0.86	19.6%
AK039608	0.21040476	0.35	167.8%
AK039624	5.228918	1.21	23.2%
AK039634	0	0.00	
AK039635	0.9979348	0.12	12.0%
AK039657	0.01937632	0.03	161.4%
AK039679	0.6196834	0.17	27.6%
AK039693	0.01612816	0.03	155.8%
AK039712	0.13055192	0.03	25.6%
AK039720	0.2532194	0.04	15.8%
AK039734	1.1047302	0.11	10.0%
AK039749	0.00485336	0.01	139.3%
AK039780	0	0.00	
AK039796	1.0369796	0.21	20.6%
AK039809	0.00977994	0.02	223.6%

AK039813	0.0169223	0.03	152.7%
AK039819	0.1969296	0.05	23.7%
AK039820	0.3542438	0.13	36.5%
AK039826	0.07259584	0.02	27.3%
AK039827	0	0.00	
AK039829	0	0.00	
AK039836	0	0.00	
AK039837	0.00750726	0.02	223.6%
AK039862	0.01463594	0.01	94.6%
AK039882	0.4172422	0.18	42.3%
AK039901	6.585184	0.92	13.9%
AK039918	0.07115986	0.02	30.9%
AK039942	0.050018	0.01	22.8%
AK039954	0.15598849	0.31	200.2%
AK039957	0	0.00	
AK039958	0.090478	0.13	139.9%
AK039960	0.17780296	0.08	47.7%
AK039995	0.6442452	0.33	50.8%
AK040009	0.7610782	0.17	21.8%
AK040027	3.58372	0.20	5.6%
AK040028	0	0.00	
AK040035	0	0.00	
AK040057	0.0248292	0.04	142.0%
AK040058	0.04494768	0.04	84.6%
AK040061	0.01750214	0.03	161.8%
AK040062	0	0.00	
AK040090	0.01164172	0.01	94.9%
AK040102	0	0.00	
AK040104	0	0.00	
AK040110	0	0.00	
AK040112	0.08669112	0.02	26.3%
AK040143	0.00575144	0.01	223.6%
AK040159	0.11448064	0.12	106.8%
AK040166	0	0.00	
AK040172	0.04258008	0.02	57.3%
AK040173	0.11271944	0.03	27.1%
AK040177	0.03039298	0.03	97.4%
AK040197	1.6274502	0.65	39.6%
AK040202	0.11608538	0.04	35.4%
AK040222	0	0.00	
AK040224	0.3151796	0.05	15.5%
AK040231	0	0.00	
AK040272	0	0.00	
AK040276	0.4314056	0.12	27.0%
AK040278	0.02572608	0.01	56.4%
AK040297	0.21136706	0.16	76.8%
AK040320	0.5670516	0.06	10.2%

AK040357	0	0.00	
AK040387	0	0.00	
AK040392	0.8919444	0.20	22.7%
AK040398	0.06659076	0.04	66.2%
AK040418	0.0846158	0.05	54.2%
AK040426	0	0.00	
AK040430	0.3711484	0.15	40.7%
AK040452	0.00252554	0.01	223.6%
AK040485	2.81205	0.40	14.4%
AK040553	0	0.00	
AK040557	0.17101914	0.30	174.3%
AK040575	0.13008542	0.03	21.5%
AK040602	0.7157638	0.20	27.3%
AK040640	0	0.00	
AK040659	0.01057738	0.02	223.6%
AK040665	0.01329076	0.03	223.6%
AK040671	0	0.00	
AK040674	0.000108811	0.00	223.6%
AK040684	0	0.00	
AK040707	0	0.00	
AK040715	1.7725502	0.50	27.9%
AK040733	0.2557968	0.06	24.1%
AK040736	0.08835676	0.06	66.4%
AK040737	0.00478674	0.01	223.6%
AK040741	1.0696326	0.23	21.6%
AK040752	0.05587744	0.01	15.6%
AK040794	0.03263568	0.02	57.4%
AK040806	0	0.00	
AK040808	0.03109472	0.01	35.3%
AK040818	3.3045	0.42	12.7%
AK040865	0.01491994	0.02	137.0%
AK040873	0.0073548	0.01	137.3%
AK040904	0.01289936	0.01	97.3%
AK040948	1.910218	0.38	20.1%
AK040960	0.04147714	0.03	68.6%
AK040967	0.0484277	0.03	64.0%
AK040992	0	0.00	
AK041042	0.3180872	0.10	31.8%
AK041061	5.425716	0.46	8.6%
AK041075	0.9442394	0.11	11.8%
AK041081	2.445556	0.38	15.4%
AK041085	0.11228002	0.03	24.5%
AK041096	0.0916097	0.11	124.3%
AK041109	0.08442002	0.04	50.8%
AK041133	0.191198	0.05	24.7%
AK041142	0	0.00	
AK041254	0	0.00	

AK041267	0.00848356	0.02	223.6%
AK041287	0.16877398	0.17	99.5%
AK041310	0	0.00	
AK041328	0	0.00	
AK041330	0.3771998	0.03	8.7%
AK041339	0	0.00	
AK041356	0.01864548	0.01	79.1%
AK041405	29.7449	1.51	5.1%
AK041408	0.12007562	0.04	35.7%
AK041437	0	0.00	
AK041441	0.00859842	0.01	110.6%
AK041461	0.00514076	0.01	223.6%
AK041463	0	0.00	
AK041465	0.07215636	0.03	40.8%
AK041531	0.03681082	0.00	10.3%
AK041537	0.1799958	0.05	27.4%
AK041570	0.3550454	0.10	27.5%
AK041587	0	0.00	
AK041589	0.0055016	0.01	223.6%
AK041600	0	0.00	
AK041609	0.04188534	0.03	61.2%
AK041610	0.2203088	0.05	21.7%
AK041614	0	0.00	
AK041628	0	0.00	
AK041647	0	0.00	
AK041706	0.00253652	0.01	223.6%
AK041716	0	0.00	
AK041746	0	0.00	
AK041755	0	0.00	
AK041760	0.02401314	0.02	85.2%
AK041762	0.01170982	0.03	223.6%
AK041780	0	0.00	
AK041781	0	0.00	
AK041795	0	0.00	
AK041797	0.18267758	0.12	64.7%
AK041893	0.10415336	0.09	87.3%
AK041972	0.1821648	0.05	27.5%
AK041976	0	0.00	
AK041998	0.0324393	0.04	116.1%
AK042010	0.07078672	0.07	102.5%
AK042016	0.00896778	0.01	138.0%
AK042028	0.22399172	0.12	54.0%
AK042041	0.12416402	0.07	54.8%
AK042124	0.03823578	0.05	141.6%
AK042126	0.1647788	0.04	25.2%
AK042136	0	0.00	
AK042188	0.04283956	0.02	39.9%

AK042210	0.02345008	0.02	68.2%
AK042223	0.23290521	0.39	168.4%
AK042229	0	0.00	
AK042235	0.3605112	0.06	18.0%
AK042249	0	0.00	
AK042292	0	0.00	
AK042300	0.6077492	0.11	18.2%
AK042313	0	0.00	
AK042336	0.00641216	0.01	223.6%
AK042353	0	0.00	
AK042354	0.01198784	0.01	59.2%
AK042436	0.8524458	0.17	20.3%
AK042445	0.2006288	0.06	27.9%
AK042514	0.0182966	0.03	137.3%
AK042551	0	0.00	
AK042554	0.06791046	0.04	55.2%
AK042602	0	0.00	
AK042610	0.0538949	0.05	89.1%
AK042619	0.3038354	0.12	40.4%
AK042647	0.005921636	0.01	91.8%
AK042665	0	0.00	
AK042686	0	0.00	
AK042707	12.526	0.60	4.8%
AK042714	0.00457112	0.01	223.6%
AK042718	0.011099564	0.01	78.5%
AK042724	0.0256747	0.03	101.2%
AK042726	0.00531078	0.01	223.6%
AK042746	0.02442698	0.03	105.6%
AK042765	0.10121462	0.02	16.6%
AK042789	0.02133408	0.02	103.7%
AK042791	0.04741764	0.01	28.3%
AK042797	0.3211148	0.13	40.8%
AK042807	0.07867894	0.02	23.0%
AK042838	0.5177334	0.07	13.5%
AK042843	0.01650988	0.02	138.3%
AK042845	0.05031182	0.02	38.8%
AK042904	0.2498126	0.09	34.5%
AK042907	0.4728726	0.19	40.9%
AK042927	0.10463718	0.03	33.3%
AK042933	0	0.00	
AK042944	0.0052107	0.01	223.6%
AK042948	0.03240792	0.01	17.3%
AK042996	14.76148	2.33	15.8%
AK042998	0.8774318	0.07	7.5%
AK043004	0.0943102	0.04	42.2%
AK043032	0.05247844	0.02	39.9%
AK043043	0.1790276	0.11	62.8%

AK043046	1.721282	0.14	7.9%
AK043075	0	0.00	
AK043085	0	0.00	
AK043087	0.07272838	0.05	66.5%
AK043092	0.1903323	0.09	46.8%
AK043101	0.880028	0.18	20.1%
AK043120	0.950547	0.74	77.4%
AK043130	2.002216	0.23	11.4%
AK043153	5.912776	0.59	10.0%
AK043155	0.10130134	0.09	92.4%
AK043163	0	0.00	
AK043166	22.79036	1.62	7.1%
AK043174	1.0585794	0.19	18.0%
AK043182	1.650964	0.16	9.7%
AK043219	0	0.00	
AK043242	4.781864	0.57	12.0%
AK043252	1.59851	0.45	27.9%
AK043267	0.03075494	0.01	41.3%
AK043278	0.1368412	0.03	19.1%
AK043285	0.9139378	0.13	14.7%
AK043286	0.07160058	0.12	171.7%
AK043292	0.1260062	0.03	27.3%
AK043303	0.00505252	0.01	223.6%
AK043305	0.3109756	0.09	27.5%
AK043311	0	0.00	
AK043331	0	0.00	
AK043332	0.02176006	0.01	24.1%
AK043359	16.24124	0.65	4.0%
AK043361	0.5932062	0.05	8.3%
AK043365	0.0054252	0.01	223.6%
AK043393	0.011791155	0.01	92.1%
AK043439	0.3562012	0.15	41.2%
AK043460	0	0.00	
AK043502	0.5810986	0.18	31.4%
AK043505	0	0.00	
AK043509	0.5082668	0.16	32.3%
AK043516	3.51641	0.54	15.4%
AK043531	0.4973512	0.12	23.3%
AK043564	0	0.00	
AK043581	0.01294062	0.02	149.3%
AK043607	0.24473	0.10	40.4%
AK043612	0.16674954	0.07	43.8%
AK043613	0	0.00	
AK043659	0.00239174	0.01	223.6%
AK043662	0	0.00	
AK043679	0.00221976	0.00	223.6%
AK043690	0.01607918	0.02	94.6%

AK043693	0.19648328	0.12	60.8%
AK043723	0.13971804	0.05	38.7%
AK043747	3.329298	0.81	24.2%
AK043766	0.27140714	0.19	71.5%
AK043780	0.3013588	0.09	30.9%
AK043789	0.4858464	0.18	37.2%
AK043790	1.528702	0.34	22.1%
AK043804	0.1102102	0.07	60.1%
AK043814	1.1496096	0.16	14.1%
AK043844	0.07277328	0.04	57.1%
AK043846	0.7642954	0.23	30.3%
AK043847	17.77534	2.23	12.5%
AK043851	2.249838	0.19	8.4%
AK043869	0	0.00	
AK043886	0	0.00	
AK043904	0.08041498	0.02	26.7%
AK043919	0.473032	0.08	16.0%
AK043922	0.00793296	0.01	138.1%
AK043935	0.4308062	0.12	27.5%
AK043947	0.00822132	0.01	141.3%
AK043958	0.3705726	0.18	49.9%
AK043964	0	0.00	
AK043973	0.16749116	0.10	60.1%
AK043982	0.06229836	0.06	94.0%
AK043991	0	0.00	
AK043994	0.13674682	0.05	33.8%
AK043996	0.0374852	0.05	141.5%
AK044020	0.3190412	0.08	23.9%
AK044033	0.20080996	0.09	43.3%
AK044036	2.437562	0.24	9.8%
AK044041	0.00809346	0.01	137.0%
AK044089	0.11343544	0.09	80.0%
AK044106	0.13043812	0.10	76.0%
AK044164	0.09772854	0.05	52.1%
AK044242	0.08683904	0.05	52.9%
AK044313	0.2239252	0.05	20.2%
AK044329	2.043006	0.14	7.1%
AK044354	0.02845813	0.04	151.7%
AK044363	0.02187646	0.01	26.2%
AK044374	0.13475016	0.04	30.8%
AK044376	2.777734	0.31	11.0%
AK044380	0.554951	0.14	25.4%
AK044414	0.3104726	0.07	23.7%
AK044422	15.2321	2.18	14.3%
AK044425	0.7844652	0.23	28.8%
AK044426	0.00273696	0.01	223.6%
AK044430	0.7582502	0.13	16.6%

AK044432	0.75603524	0.65	86.5%
AK044435	0.2135224	0.07	34.1%
AK044447	0.09624434	0.04	41.4%
AK044449	0.00568768	0.01	140.4%
AK044466	0.11001758	0.01	11.9%
AK044480	0.2953796	0.08	26.8%
AK044491	0.00166527	0.00	223.6%
AK044503	0.20017366	0.08	39.4%
AK044505	0.0901335	0.05	50.1%
AK044526	0.2765384	0.06	20.7%
AK044563	3.49356	0.49	13.9%
AK044622	0.4976702	0.09	19.1%
AK044623	0.1313718	0.02	18.5%
AK044644	0.5150202	0.10	18.6%
AK044653	0.05280562	0.02	43.4%
AK044673	0	0.00	
AK044689	2.27851	0.36	15.6%
AK044780	0	0.00	
AK044786	0.009093	0.01	147.9%
AK044789	0.286433	0.02	7.7%
AK044848	0.09311862	0.03	35.9%
AK044901	2.682924	0.30	11.3%
AK045038	7.889482	0.37	4.7%
AK045070	0.11717096	0.02	15.8%
AK045112	0.4344288	0.09	21.7%
AK045137	0.04467702	0.05	105.5%
AK045142	0.09144854	0.04	40.6%
AK045225	1.1041624	0.28	25.8%
AK045242	0	0.00	
AK045244	0.8079946	0.13	16.6%
AK045290	1.227278	0.14	11.3%
AK045371	11.77526	0.41	3.5%
AK045385	0.171895	0.04	25.7%
AK045413	1.922342	0.47	24.5%
AK045483	0.00355372	0.01	223.6%
AK045502	0.5270282	0.08	15.5%
AK045519	0.0888823	0.03	39.2%
AK045535	6.00551	0.59	9.9%
AK045614	0.09136072	0.03	31.5%
AK045624	0.0608596	0.03	41.8%
AK045637	1.730114	0.42	24.5%
AK045643	1.372712	0.22	16.2%
AK045649	9.223206	0.95	10.3%
AK045681	0.2204892	0.06	29.4%
AK045688	0.4982674	0.11	22.6%
AK045700	0.01673278	0.03	155.8%
AK045728	0.01440236	0.02	110.0%

AK045744	0.6474494	0.20	30.5%
AK045755	0.0370608	0.03	91.1%
AK045786	0.0251726	0.06	223.6%
AK045807	0.173881	0.05	26.4%
AK045814	0.7213446	0.09	12.3%
AK045818	0.940274	0.15	16.3%
AK045825	0.07637984	0.06	73.8%
AK045837	0.5123414	0.04	7.9%
AK045842	0.17485924	0.09	51.6%
AK045885	2.433368	0.67	27.7%
AK045913	0.1685628	0.04	22.7%
AK045932	0.2339038	0.04	19.0%
AK045951	0	0.00	
AK045952	1.1666486	0.44	37.5%
AK045970	0.623117	0.13	20.3%
AK046006	0.11967484	0.03	22.1%
AK046023	3.796678	0.22	5.7%
AK046026	0.1105248	0.03	24.6%
AK046042	0.833097	0.29	35.2%
AK046044	1.381976	0.15	10.7%
AK046045	0.07193366	0.04	56.9%
AK046134	1.892962	0.26	14.0%
AK046150	0.3926184	0.09	22.4%
AK046160	0.2507392	0.10	39.1%
AK046168	0	0.00	
AK046172	0.16042046	0.14	86.0%
AK046182	0	0.00	
AK046224	1.771602	0.40	22.5%
AK046225	0.9157168	0.22	23.7%
AK046240	1.0015312	0.51	50.4%
AK046242	0	0.00	
AK046278	7.56932	0.30	4.0%
AK046300	0.04754378	0.02	39.6%
AK046304	6.902172	0.32	4.6%
AK046316	0.90504	0.08	9.2%
AK046349	0.0133405	0.03	223.6%
AK046351	0.919828	0.08	8.2%
AK046385	0.08823232	0.04	45.6%
AK046388	0	0.00	
AK046417	5.921714	0.40	6.7%
AK046418	19.904	3.25	16.3%
AK046440	0.0496196	0.07	141.1%
AK046443	0.6239882	0.13	20.1%
AK046721	0.08866954	0.01	15.4%
AK046727	0.0362584	0.02	52.4%
AK046737	0.0723307	0.03	39.3%
AK046740	22.80574	0.92	4.0%

AK046742	0.1889356	0.27	142.4%
AK046786	0.2257294	0.08	37.1%
AK046846	3.06984	0.29	9.3%
AK046920	3.789188	0.28	7.4%
AK046940	2.354354	0.55	23.5%
AK046967	0.3732284	0.08	20.8%
AK046981	0.0669792	0.05	67.4%
AK047022	7.6404	0.15	1.9%
AK047030	1.235736	0.12	9.6%
AK047141	0.00298464	0.01	223.6%
AK047145	0	0.00	
AK047163	0	0.00	
AK047175	0.0498559	0.02	47.7%
AK047177	0.000949308	0.00	223.6%
AK047199	0.06022294	0.02	37.0%
AK047239	0	0.00	
AK047243	0.030129244	0.03	99.8%
AK047272	1.360568	0.30	21.8%
AK047309	0	0.00	
AK047323	0.11595894	0.06	52.1%
AK047366	0.1945858	0.09	48.7%
AK047370	0.346916	0.11	30.9%
AK047441	0.18104474	0.11	58.4%
AK047520	0.4354862	0.11	25.0%
AK047534	0.02296102	0.03	124.7%
AK047558	1.459252	0.03	2.2%
AK047607	0.09468286	0.03	30.9%
AK047660	0.14342372	0.09	62.0%
AK047676	1.116324	0.10	8.8%
AK047727	0.12752838	0.11	90.0%
AK047741	0.6077558	0.17	27.4%
AK047765	0.02063808	0.02	105.7%
AK047804	0.00897942	0.02	223.6%
AK047857	0.0734481	0.02	22.5%
AK047916	0.01796222	0.01	30.1%
AK047931	2.008966	0.35	17.6%
AK047938	0.010865137	0.01	105.2%
AK047944	0.7118638	0.16	22.6%
AK047946	2.565998	0.49	19.2%
AK047978	0.3070824	0.09	29.8%
AK047984	0.2135548	0.04	20.6%
AK047988	0.2444284	0.04	18.2%
AK048013	1.80296	0.36	20.1%
AK048019	0.00676086	0.02	223.6%
AK048031	0.20942078	0.10	47.9%
AK048032	0	0.00	
AK048037	97.72796	1.78	1.8%

AK048048	0.001918515	0.00	131.3%
AK048050	0.00454664	0.01	223.6%
AK048080	3.2111	0.32	9.9%
AK048089	0.07685546	0.02	24.9%
AK048257	0.13495438	0.13	96.1%
AK048274	0	0.00	
AK048275	0.01706312	0.01	56.2%
AK048280	3.201412	0.18	5.6%
AK048292	0.3794732	0.13	35.3%
AK048368	0.02491914	0.01	51.5%
AK048429	0.2163424	0.12	53.5%
AK048446	2.2300308	1.12	50.1%
AK048484	0.19621524	0.16	82.8%
AK048523	0.05292158	0.02	46.3%
AK048571	1.1136418	0.61	54.7%
AK048592	0.05701642	0.06	108.5%
AK048630	0.13487374	0.06	44.4%
AK048664	0.09727532	0.12	128.5%
AK048672	0.02940858	0.01	39.8%
AK048681	7.68409	1.50	19.6%
AK048710	0.773424	0.14	18.0%
AK048739	0.07126792	0.02	26.2%
AK048775	0.016483242	0.01	69.6%
AK048798	0.07447566	0.05	73.8%
AK048814	0.00349882	0.01	223.6%
AK048828	0.4583648	0.16	34.0%
AK048831	0.04723918	0.01	18.0%
AK048846	1.1935214	0.19	16.0%
AK048878	0.04954188	0.02	40.8%
AK048886	0.6940176	0.13	18.5%
AK048941	0.2101618	0.08	35.9%
AK048946	0	0.00	
AK048993	0.452117	0.05	12.0%
AK048995	0.09671006	0.03	32.0%
AK049017	0.12930738	0.04	30.9%
AK049041	0.350075308	0.71	201.5%
AK049068	0.4226762	0.11	26.0%
AK049102	0.05595932	0.04	68.6%
AK049111	1.565332	0.32	20.2%
AK049123	0.11860756	0.07	58.3%
AK049146	0.006360122	0.01	158.4%
AK049160	0.08115952	0.04	45.2%
AK049168	0.00342902	0.01	223.6%
AK049221	0	0.00	
AK049248	0.03284232	0.04	121.2%
AK049264	0.12937922	0.06	44.0%
AK049282	0	0.00	

AK049289	0.14417632	0.03	23.2%
AK049295	0.01740218	0.02	99.9%
AK049416	0.13253202	0.04	32.4%
AK049508	0.11901442	0.06	50.4%
AK049515	1.7973136	1.40	78.2%
AK049522	0	0.00	
AK049536	0.1518464	0.10	64.0%
AK049578	0.09392686	0.05	52.8%
AK049621	0.20544504	0.12	59.6%
AK049630	0.678946	0.16	24.0%
AK049663	0.1065725	0.06	52.0%
AK049668	2.741722	0.27	10.0%
AK049769	1.6715978	0.54	32.3%
AK049838	0.07965636	0.04	46.4%
AK049847	0.05767324	0.01	12.8%
AK049852	0.802517	0.12	14.5%
AK049860	0.2220554	0.08	35.6%
AK049871	0.0141937	0.02	141.6%
AK049951	9.800738	1.07	10.9%
AK049972	8.06802	0.38	4.7%
AK049985	0.0278545	0.02	64.3%
AK050069	0.0914124	0.09	95.6%
AK050085	0.01072072	0.01	138.1%
AK050091	0.06461226	0.02	35.9%
AK050117	0.12139052	0.20	166.9%
AK050144	0.015747	0.04	223.6%
AK050164	0.04733248	0.02	39.7%
AK050169	6.985346	0.72	10.4%
AK050197	0	0.00	
AK050201	0.1544556	0.04	23.0%
AK050217	0	0.00	
AK050232	0.07616988	0.03	38.1%
AK050253	0.5186694	0.14	26.2%
AK050258	0	0.00	
AK050283	0.00516748	0.01	223.6%
AK050290	0.8430714	0.59	69.5%
AK050336	0	0.00	
AK050349	0	0.00	
AK050351	0	0.00	
AK050360	0	0.00	
AK050464	0.01643008	0.02	108.2%
AK050510	0	0.00	
AK050516	0	0.00	
AK050558	0.05297098	0.02	42.7%
AK050586	1.529378	0.23	15.1%
AK050592	0	0.00	
AK050700	0	0.00	

AK050707	0.7290408	0.12	16.1%
AK050735	0.9191182	0.12	12.6%
AK050745	0.3459774	0.10	29.1%
AK050795	5.487092	0.37	6.7%
AK050809	0.6519176	0.16	24.6%
AK050838	0.02485402	0.01	27.8%
AK050844	0	0.00	
AK050859	1.28937	0.17	13.2%
AK050866	0.01359926	0.01	99.7%
AK050909	0	0.00	
AK050940	0.505056	0.11	22.5%
AK051008	0	0.00	
AK051019	0.07180102	0.03	48.6%
AK051045	1.870368	0.26	13.8%
AK051055	0.00564374	0.01	223.6%
AK051057	0.00553066	0.01	223.6%
AK051160	24.22806	1.63	6.7%
AK051166	0.018413124	0.01	38.7%
AK051202	0.0415765	0.02	41.7%
AK051321	0	0.00	
AK051368	0.10778918	0.05	48.1%
AK051417	3.102044	0.15	4.7%
AK051424	0.03999988	0.03	72.6%
AK051451	0	0.00	
AK051464	0.000670495	0.00	194.8%
AK051482	0.00648868	0.01	141.0%
AK051495	0.016394184	0.01	84.3%
AK051573	201.3888	21.37	10.6%
AK051656	0.5165704	0.08	14.7%
AK051771	0.006835658	0.01	102.3%
AK051804	0.1989616	0.04	22.4%
AK051847	1.998712	0.19	9.6%
AK051864	0.00851092	0.01	91.9%
AK051887	0.2272002	0.05	21.1%
AK051928	0.4871314	0.17	35.5%
AK052076	0.05338576	0.04	82.8%
AK052157	0.21993468	0.25	112.8%
AK052161	0.21275972	0.09	42.8%
AK052226	0.04516878	0.05	108.5%
AK052293	1.2169024	0.39	32.4%
AK052319	4.950596	0.76	15.3%
AK052414	3.227892	0.63	19.5%
AK052495	0.6093578	0.63	103.3%
AK052569	1.1496712	0.24	20.6%
AK052572	0	0.00	
AK052590	0.03020918	0.02	57.2%
AK052594	0.01571528	0.01	68.7%

AK052627	0	0.00	
AK052711	0	0.00	
AK052712	0.011267394	0.01	91.5%
AK052773	0.4248496	0.08	19.7%
AK052788	0	0.00	
AK052792	0.1378928	0.16	117.6%
AK052812	0.431003	0.13	29.8%
AK052874	0.14303016	0.11	73.9%
AK052878	0.0361895	0.03	93.8%
AK052880	0.01382496	0.02	169.6%
AK052881	0.06681586	0.01	13.0%
AK052888	0.00299138	0.01	223.6%
AK052909	0.4441426	0.16	37.0%
AK053027	0.7661718	0.12	15.8%
AK053040	0.0033929	0.01	223.6%
AK053084	0.11274618	0.05	40.3%
AK053109	0.02322056	0.02	83.2%
AK053124	0.001834742	0.00	223.6%
AK053136	0.01139662	0.02	139.0%
AK053148	0.02480294	0.02	99.6%
AK053157	0	0.00	
AK053193	0.0414511	0.02	59.7%
AK053224	0.1819802	0.05	25.5%
AK053225	0	0.00	
AK053246	0.2115045	0.15	71.9%
AK053270	0.3582356	0.10	27.0%
AK053274	0.4931608	0.19	39.1%
AK053295	0.12893324	0.05	36.6%
AK053296	0.0030938	0.01	223.6%
AK053303	0.011274088	0.01	68.6%
AK053334	0.0048573	0.01	138.1%
AK053386	0.12625262	0.08	62.1%
AK053416	0	0.00	
AK053418	0.07796564	0.04	54.6%
AK053469	1.603702	0.31	19.4%
AK053505	0.1540602	0.04	26.8%
AK053540	21.99404	6.97	31.7%
AK053590	5.060982	0.49	9.6%
AK053602	0.03236788	0.03	103.5%
AK053689	3.057464	0.93	30.4%
AK053703	1.276944	0.10	8.1%
AK053708	0.02358634	0.01	41.6%
AK053722	0.011052969	0.01	91.3%
AK053772	0.17951202	0.07	37.4%
AK053779	1.792278	0.32	18.1%
AK053790	0.05653494	0.05	89.2%
AK053957	0.00523206	0.01	137.3%

AK053982	2.685572	0.60	22.2%
AK053993	0.1677176	0.02	13.9%
AK053994	0	0.00	
AK054042	0.3052376	0.10	31.8%
AK054074	0.009885196	0.01	75.0%
AK054076	0.3339804	0.10	31.1%
AK054106	0.0406414	0.04	88.3%
AK054128	0.004963438	0.01	161.4%
AK054201	0.01259318	0.02	138.5%
AK054221	0	0.00	
AK054265	0	0.00	
AK054333	0	0.00	
AK054344	0	0.00	
AK054420	0.04932692	0.01	25.0%
AK054431	0	0.00	
AK054442	0	0.00	
AK054493	0.02704456	0.02	69.1%
AK054504	0	0.00	
AK054515	0.02048802	0.02	100.1%
AK054526	0.00729804	0.02	223.6%
AK057615	7.685312	0.80	10.4%
AK075572	0.5264286	0.15	27.8%
AK075633	0	0.00	
AK075658	0	0.00	
AK075664	4.015184	0.20	5.1%
AK075667	0.03367844	0.03	76.4%
AK075695	0.18972796	0.09	48.4%
AK075724	1.1805188	0.26	22.2%
AK076052	0.17449488	0.08	43.3%
AK076253	0	0.00	
AK076313	0.11754856	0.04	34.8%
AK076318	0.006199462	0.01	152.8%
AK076358	0.07998992	0.05	57.6%
AK076372	0.4626148	0.11	24.0%
AK076389	0.885436	0.18	20.3%
AK076396	0.13385452	0.03	23.6%
AK076440	0.00913498	0.01	94.3%
AK076555	0.10652706	0.03	26.7%
AK076583	2.901664	0.38	13.1%
AK076597	0.0045091	0.01	223.6%
AK076606	2.441276	0.82	33.6%
AK076622	0	0.00	
AK076652	3.78122	0.51	13.6%
AK076655	0	0.00	
AK076657	0	0.00	
AK076658	0	0.00	
AK076660	0	0.00	

AK076661	0.5324336	0.13	23.8%
AK076664	0	0.00	
AK076665	0	0.00	
AK076668	0.6049942	0.39	64.8%
AK076672	0	0.00	
AK076673	0	0.00	
AK076674	0	0.00	
AK076676	0	0.00	
AK076677	0.0165686	0.00	26.4%
AK076683	0.8700664	0.14	16.2%
AK076685	0	0.00	
AK076687	0.00602364	0.01	223.6%
AK076688	0	0.00	
AK076690	0.07746552	0.04	47.5%
AK076692	0	0.00	
AK076700	0	0.00	
AK076701	0.2619692	0.07	25.2%
AK076702	0	0.00	
AK076706	0	0.00	
AK076709	0.01481602	0.02	140.7%
AK076711	0	0.00	
AK076715	0	0.00	
AK076716	0.1907842	0.09	46.6%
AK076722	0.0876587	0.06	72.7%
AK076728	0	0.00	
AK076733	0	0.00	
AK076734	0.0149057	0.03	223.6%
AK076739	0.00398278	0.01	223.6%
AK076752	0.0015926	0.00	223.6%
AK076755	0	0.00	
AK076759	0.12944984	0.10	79.8%
AK076771	0.05369084	0.04	72.4%
AK076774	0	0.00	
AK076779	0.05137424	0.03	57.9%
AK076783	0	0.00	
AK076784	0	0.00	
AK076785	0.00390082	0.01	223.6%
AK076789	0	0.00	
AK076790	0.0259748	0.06	223.6%
AK076794	0	0.00	
AK076796	0	0.00	
AK076800	0.01777384	0.04	223.6%
AK076805	0	0.00	
AK076808	0	0.00	
AK076809	0	0.00	
AK076817	0.00858628	0.02	223.6%
AK076827	0.0088541	0.02	223.6%

AK076831	0.01810854	0.03	144.0%
AK076839	0.3358682	0.10	31.2%
AK076840	0.01112324	0.02	223.6%
AK076842	0	0.00	
AK076845	0	0.00	
AK076846	2.50023	0.09	3.5%
AK076847	0	0.00	
AK076848	0	0.00	
AK076849	0.08226318	0.08	101.5%
AK076851	0	0.00	
AK076852	0	0.00	
AK076853	0	0.00	
AK076859	0.05272428	0.07	126.8%
AK076865	0	0.00	
AK076883	0.0227327	0.02	103.3%
AK076884	0	0.00	
AK076892	0	0.00	
AK076894	0	0.00	
AK076896	0	0.00	
AK076897	0.0124769	0.03	223.6%
AK076902	0	0.00	
AK076905	0	0.00	
AK076906	0	0.00	
AK076909	0	0.00	
AK076911	0	0.00	
AK076914	0	0.00	
AK076916	0.001958556	0.00	223.6%
AK076918	0	0.00	
AK076922	2.312174	0.37	16.0%
AK076925	0.2194246	0.12	55.5%
AK076929	0	0.00	
AK076930	0	0.00	
AK076931	0	0.00	
AK076935	0	0.00	
AK076937	0	0.00	
AK076938	0.00792002	0.02	223.6%
AK076939	0	0.00	
AK076945	0	0.00	
AK076946	0	0.00	
AK076947	0	0.00	
AK076949	0	0.00	
AK076950	0.14706208	0.09	64.1%
AK076952	0	0.00	
AK076954	0	0.00	
AK076959	0	0.00	
AK076961	0	0.00	
AK076963	0	0.00	

AK076965	0.00974642	0.02	223.6%
AK076966	0	0.00	
AK076976	0	0.00	
AK076978	0.998425	0.13	12.5%
AK076979	0.09449886	0.08	82.9%
AK076984	0.02811114	0.03	116.1%
AK076987	0.1276989	0.09	71.8%
AK076988	0	0.00	
AK077008	0	0.00	
AK077029	0.00189406	0.00	223.6%
AK077035	0	0.00	
AK077040	1.9907244	1.38	69.4%
AK077064	0	0.00	
AK077070	0	0.00	
AK077074	0.18013092	0.05	27.6%
AK077115	0	0.00	
AK077134	0.04895436	0.09	176.1%
AK077135	0.0720385	0.03	37.5%
AK077143	0	0.00	
AK077157	0	0.00	
AK077163	0.0443163	0.02	46.1%
AK077166	0	0.00	
AK077175	0.00845382	0.01	94.0%
AK077183	0.00799942	0.02	223.6%
AK077204	0.03844938	0.03	79.7%
AK077272	0.0032599	0.01	223.6%
AK077315	3.14633	0.70	22.3%
AK077350	0	0.00	
AK077353	0.02320118	0.01	30.2%
AK077358	0.01561472	0.01	94.6%
AK077398	0.969002	0.16	16.1%
AK077405	0.04880158	0.04	73.2%
AK077427	0.11907176	0.06	52.5%
AK077474	0.3868198	0.10	26.8%
AK077481	0.136882	0.11	81.4%
AK077492	0.03661364	0.06	152.6%
AK077587	0.4112504	0.10	24.5%
AK077682	0.2771234	0.10	35.5%
AK077773	0.01245662	0.01	62.9%
AK077811	0.06977228	0.04	55.8%
AK077954	0.62373	0.19	31.2%
AK078103	0.05082318	0.03	52.0%
AK078122	0.3731558	0.13	35.1%
AK078191	0.6427382	0.11	16.8%
AK078255	0.12364692	0.10	83.2%
AK078290	13.13658	0.89	6.8%
AK078307	0.22643542	0.11	48.8%

AK078316	0.6633546	0.09	13.3%
AK078321	1.0198728	0.17	17.1%
AK078352	0.15691146	0.10	63.8%
AK078363	0.3184542	0.15	48.0%
AK078367	0	0.00	
AK078446	0.2304132	0.03	12.3%
AK078466	0.1761001	0.11	61.5%
AK078566	0.08634938	0.06	66.2%
AK078606	0.01553984	0.02	103.8%
AK078660	0.3132334	0.07	22.2%
AK078705	0	0.00	
AK078711	4.291988	0.30	7.1%
AK078712	0.10804812	0.05	44.7%
AK078754	0.390478	0.07	18.2%
AK078772	0	0.00	
AK078795	22.58572	0.57	2.5%
AK078801	0.18298898	0.08	44.4%
AK078896	0.01194226	0.03	223.6%
AK078907	0	0.00	
AK078931	0	0.00	
AK078932	0.1477332	0.15	104.2%
AK078937	0.2112696	0.09	40.8%
AK078949	0	0.00	
AK078979	0.357092	0.06	17.2%
AK078991	0	0.00	
AK079003	0	0.00	
AK079012	0	0.00	
AK079017	0.04678344	0.04	79.1%
AK079018	0	0.00	
AK079019	0	0.00	
AK079034	0.12594188	0.05	42.8%
AK079066	0	0.00	
AK079188	0.0690003	0.03	39.0%
AK079194	0.2069564	0.06	27.8%
AK079222	0	0.00	
AK079255	0.195449	0.03	15.6%
AK079263	0.312072	0.05	15.2%
AK079280	0.3541088	0.10	28.4%
AK079285	1.8924172	1.04	54.9%
AK079377	0.06545518	0.05	78.5%
AK079380	1.676976	0.13	7.7%
AK079403	0.18449936	0.07	36.6%
AK079404	0.01007722	0.00	46.0%
AK079438	0	0.00	
AK079527	0.07559054	0.04	54.7%
AK079595	0	0.00	
AK079637	0.13831626	0.08	58.0%

AK079660	0.0113942	0.01	103.1%
AK079662	0	0.00	
AK079670	0	0.00	
AK079675	0	0.00	
AK079699	0.646943	0.16	24.3%
AK079709	0.06303826	0.08	129.9%
AK079713	0	0.00	
AK079714	0	0.00	
AK079730	0.2004168	0.07	33.8%
AK079754	0.6745108	0.09	14.0%
AK079763	0	0.00	
AK079777	0.5928928	0.10	17.0%
AK079798	0	0.00	
AK079802	0	0.00	
AK079804	0.04940884	0.05	92.8%
AK079812	0.001942854	0.00	223.6%
AK079813	9.84758E-05	0.00	223.6%
AK079818	0	0.00	
AK079855	16.5412	1.07	6.5%
AK079857	0	0.00	
AK079861	0.05609228	0.02	35.7%
AK079912	0.0713465	0.06	80.0%
AK079920	0.1745332	0.05	28.0%
AK079921	0	0.00	
AK079929	0.00576662	0.01	223.6%
AK079938	0	0.00	
AK079939	0.5741436	0.16	27.9%
AK079953	0	0.00	
AK079954	0	0.00	
AK079957	0.16537862	0.07	43.7%
AK079958	0.01417092	0.03	223.6%
AK079994	0.02917792	0.03	107.5%
AK080009	0.00268562	0.01	223.6%
AK080010	0	0.00	
AK080030	0.01493764	0.02	141.2%
AK080051	0.1302609	0.06	46.2%
AK080057	0	0.00	
AK080063	0	0.00	
AK080070	0.00733494	0.02	223.6%
AK080084	1.7077726	1.15	67.2%
AK080092	0	0.00	
AK080102	0.0103265	0.02	223.6%
AK080141	0.2654574	0.02	8.4%
AK080151	0	0.00	
AK080160	0.11560286	0.03	29.4%
AK080165	0.10761304	0.09	88.0%
AK080168	0	0.00	

AK080173	0.9172812	0.53	57.7%
AK080176	2.310364	0.08	3.6%
AK080187	0.06390928	0.02	29.2%
AK080222	0.3817864	0.07	17.1%
AK080223	0.04909146	0.05	110.5%
AK080247	0	0.00	
AK080258	0.04119198	0.03	60.8%
AK080278	0.2432502	0.10	42.6%
AK080292	0.3822794	0.07	19.3%
AK080296	0.9626222	0.19	20.2%
AK080311	0	0.00	
AK080343	0	0.00	
AK080350	6.662766	1.39	20.9%
AK080351	0	0.00	
AK080352	0.0229281	0.03	138.6%
AK080367	0.0970927	0.02	24.8%
AK080383	0.8647558	0.40	46.6%
AK080399	0.13632892	0.04	26.0%
AK080411	0.544314	0.11	19.3%
AK080416	0.15459832	0.12	80.7%
AK080422	0	0.00	
AK080425	0.02822252	0.04	144.7%
AK080443	0.05773286	0.05	78.2%
AK080484	0.012718136	0.02	129.0%
AK080597	40.04502	2.40	6.0%
AK080631	0.0270527	0.03	97.5%
AK080648	0.13793726	0.07	49.1%
AK080650	1.0474408	0.44	42.3%
AK080655	0.8242058	0.37	45.2%
AK080656	27.02786	0.62	2.3%
AK080657	0.00387908	0.01	223.6%
AK080663	0.13006658	0.11	83.7%
AK080684	0.4627096	0.23	49.5%
AK080695	0	0.00	
AK080710	0.00227956	0.01	223.6%
AK080719	0.9342606	0.17	17.9%
AK080816	0.05653696	0.03	58.6%
AK080857	0.4045992	0.10	24.3%
AK080902	0	0.00	
AK080946	8.308738	1.48	17.8%
AK080982	0.01047048	0.01	107.4%
AK080999	0.0520051	0.04	70.8%
AK081086	0	0.00	
AK081104	0	0.00	
AK081136	2.433038	0.35	14.2%
AK081140	0.2507442	0.02	6.7%
AK081193	7.598908	0.77	10.2%

AK081204	3.206614	0.32	9.9%
AK081244	4.253534	0.94	22.2%
AK081271	0.00743554	0.02	223.6%
AK081407	2.200912	0.36	16.5%
AK081414	0.7223274	0.09	11.8%
AK081497	1.916318	0.18	9.5%
AK081540	0	0.00	
AK081570	2.054632	0.63	30.6%
AK081581	2.964512	0.74	24.8%
AK081588	0.2592198	0.13	49.0%
AK081763	0.2227566	0.07	29.5%
AK081788	0.006024222	0.00	72.3%
AK081794	0.00963174	0.02	223.6%
AK081796	0.6433392	0.14	21.0%
AK081809	0.00617536	0.01	137.3%
AK081879	6.409558	0.80	12.4%
AK081945	0	0.00	
AK081964	0.0038226	0.01	223.6%
AK081990	3.229344	0.35	11.0%
AK082063	0.09349816	0.06	68.6%
AK082072	1.5497208	0.44	28.5%
AK082086	0.02469598	0.03	104.6%
AK082096	14.77894	0.77	5.2%
AK082104	0	0.00	
AK082110	0.21377438	0.22	105.2%
AK082117	0.05460738	0.03	47.8%
AK082148	0	0.00	
AK082200	0.3143642	0.06	18.9%
AK082250	3.31024732	4.67	141.0%
AK082264	0.4558456	0.10	20.9%
AK082307	0.3019972	0.10	31.6%
AK082314	0	0.00	
AK082325	1.235088	0.16	13.2%
AK082432	0.1050114	0.03	32.1%
AK082440	0	0.00	
AK082462	0.3686258	0.30	81.3%
AK082467	0.55356618	0.54	98.2%
AK082503	0.000878936	0.00	223.6%
AK082514	0	0.00	
AK082553	0.22624242	0.08	36.9%
AK082555	0.49874402	0.66	131.8%
AK082624	18.99766	0.86	4.5%
AK082625	44.64658	2.51	5.6%
AK082685	12.63698	0.66	5.2%
AK082693	0	0.00	
AK082720	0.04787272	0.04	82.4%
AK082731	2.898812	0.43	14.7%

AK082757	14.9442	1.51	10.1%
AK082764	0.01476738	0.02	139.6%
AK082779	0.6710202	0.34	50.7%
AK082801	3.305218	0.77	23.4%
AK082813	0	0.00	
AK082841	1.1541252	0.52	45.0%
AK082845	0.00537694	0.01	223.6%
AK082857	2.285534	0.23	9.9%
AK082862	0	0.00	
AK082869	0.02278492	0.00	14.0%
AK082896	1.32948	0.16	12.0%
AK082921	3.52411	0.20	5.8%
AK082948	0	0.00	
AK082954	0.1672078	0.04	24.6%
AK083021	2.920956	0.86	29.5%
AK083030	0.5509956	0.45	81.5%
AK083054	0.2170212	0.02	11.2%
AK083124	1.777074	0.18	9.9%
AK083149	0.1554118	0.07	43.0%
AK083150	0.01319092	0.02	150.7%
AK083177	0	0.00	
AK083190	1.487104	0.16	10.8%
AK083195	0.5467814	0.21	39.2%
AK083197	13.6952	0.58	4.3%
AK083200	1.919744	0.37	19.4%
AK083203	0.12158132	0.04	32.0%
AK083230	0.341536	0.12	33.7%
AK083252	0.07722616	0.03	34.7%
AK083296	0.03322148	0.01	39.4%
AK083339	0	0.00	
AK083382	4.0149	0.31	7.7%
AK083547	0.7513118	0.04	4.9%
AK083567	4.47803	0.48	10.7%
AK083606	0.00547036	0.01	141.2%
AK083703	0	0.00	
AK083706	0.035897466	0.02	48.5%
AK083757	0.0821843	0.03	41.6%
AK083771	0.03791632	0.04	107.4%
AK083777	0.0596659	0.03	48.0%
AK083927	0.1444248	0.06	43.8%
AK083994	0.02984058	0.03	101.5%
AK084028	0.0395728	0.01	29.2%
AK084059	0.04923392	0.04	71.3%
AK084074	0.01497308	0.00	27.2%
AK084081	0.057082	0.03	52.2%
AK084170	0.2906214	0.05	17.4%
AK084200	0.03054296	0.02	73.8%

AK084265	0.03651762	0.05	142.1%
AK084270	0.00954092	0.01	138.4%
AK084284	0.6237744	0.13	20.6%
AK084356	0	0.00	
AK084362	2.814886	0.34	12.2%
AK084432	0	0.00	
AK084470	0.7665412	0.22	28.2%
AK084486	0.04364634	0.02	43.6%
AK084494	0.19479094	0.12	59.5%
AK084504	0.1857536	0.08	45.3%
AK084517	0.18114446	0.09	49.8%
AK084554	0	0.00	
AK084560	0.11226694	0.06	51.4%
AK084599	0	0.00	
AK084679	0.00172473	0.00	223.6%
AK084741	1.675776	0.23	13.7%
AK084744	0	0.00	
AK084778	2.43984	0.36	14.8%
AK084809	0.5890682	0.10	17.3%
AK084828	0.00790496	0.02	223.6%
AK084954	0.5841186	0.12	20.9%
AK085062	0.09981136	0.05	49.5%
AK085125	0.25244614	0.22	86.4%
AK085136	6.449434	1.39	21.5%
AK085172	0.0045076	0.01	223.6%
AK085201	0.00788628	0.01	161.7%
AK085253	0	0.00	
AK085274	0.1740228	0.04	23.1%
AK085275	0.09382764	0.04	44.2%
AK085279	0	0.00	
AK085287	0.07405012	0.04	52.5%
AK085300	0.00665484	0.01	137.3%
AK085305	0.04077452	0.01	17.0%
AK085316	0	0.00	
AK085322	0	0.00	
AK085325	0.04995404	0.03	67.4%
AK085337	0.3066112	0.05	15.2%
AK085341	0.347882	0.09	25.7%
AK085342	0.010600532	0.01	108.6%
AK085353	0	0.00	
AK085365	2.600972	0.62	23.7%
AK085371	0.1853372	0.05	28.2%
AK085384	0.4288662	0.03	7.3%
AK085396	0.1727688	0.04	23.5%
AK085420	0	0.00	
AK085428	0	0.00	
AK085438	0.1048564	0.09	88.8%

AK085456	0	0.00	
AK085459	0.12458632	0.07	54.3%
AK085477	0.07854204	0.06	75.2%
AK085480	0	0.00	
AK085522	0.06237074	0.07	118.3%
AK085533	0	0.00	
AK085535	0	0.00	
AK085570	0.01385198	0.02	138.5%
AK085609	0.02372034	0.02	93.1%
AK085625	1.389938	0.43	31.1%
AK085632	0	0.00	
AK085652	0	0.00	
AK085758	0	0.00	
AK085760	0.03293278	0.01	37.8%
AK085761	0.0751164	0.05	71.1%
AK085762	0	0.00	
AK085764	0	0.00	
AK085768	0	0.00	
AK085771	0	0.00	
AK085792	0	0.00	
AK085818	0	0.00	
AK085876	0.4466654	0.07	14.9%
AK085881	0	0.00	
AK085889	0.08591892	0.06	70.5%
AK085891	0.177235	0.04	20.2%
AK085912	0	0.00	
AK085942	0.2224758	0.09	38.4%
AK085943	0	0.00	
AK085944	0.6550552	0.11	16.4%
AK085963	0.93821444	1.25	132.7%
AK085969	0.4473704	0.05	10.2%
AK085972	0	0.00	
AK085986	0	0.00	
AK085995	0.07194672	0.03	39.1%
AK086005	0.01837862	0.04	223.6%
AK086006	0.0103468	0.02	223.6%
AK086010	0.1294564	0.03	24.2%
AK086013	0	0.00	
AK086039	0	0.00	
AK086050	10.141132	0.65	6.4%
AK086087	1.1343784	0.24	21.2%
AK086149	0.7969188	0.17	20.7%
AK086164	0.11836746	0.04	37.8%
AK086168	0.06498518	0.02	29.8%
AK086245	0.00723994	0.02	223.6%
AK086273	0.229978	0.06	26.8%
AK086288	0.00871582	0.02	223.6%

AK086290	1.34298	0.22	16.7%
AK086315	1.68595	0.25	14.7%
AK086341	0.4199454	0.14	34.3%
AK086355	37.58588	1.98	5.3%
AK086375	0.4618434	0.06	13.3%
AK086420	0.01445766	0.02	137.6%
AK086439	0	0.00	
AK086455	0.0903297	0.01	7.7%
AK086460	0.2105634	0.04	19.7%
AK086477	9.158076	1.31	14.3%
AK086522	0	0.00	
AK086534	43.92226	7.15	16.3%
AK086556	0.08357652	0.02	21.3%
AK086602	0.59948306	0.43	71.8%
AK086654	0.2572904	0.14	55.6%
AK086660	0	0.00	
AK086712	0.779973	0.07	8.9%
AK086736	0.7116214	0.14	19.7%
AK086741	0.3658908	0.09	24.6%
AK086749	1.798484	0.30	16.7%
AK086758	3.681058	0.63	17.0%
AK086793	0.5747056	0.12	20.9%
AK086826	0.3828636	0.04	11.4%
AK086852	0.557539	0.14	24.4%
AK086861	2.164616	0.39	18.1%
AK086881	6.755986	0.64	9.5%
AK086911	0.01324964	0.01	62.8%
AK086914	1.814946	0.49	26.7%
AK086943	0	0.00	
AK086952	0.5618728	0.11	18.9%
AK086959	0	0.00	
AK086971	0	0.00	
AK087024	0.0414831	0.03	72.8%
AK087045	0	0.00	
AK087094	0.04896236	0.05	100.0%
AK087115	0.00315616	0.01	223.6%
AK087124	2.781572	0.15	5.5%
AK087178	0	0.00	
AK087181	0	0.00	
AK087201	0	0.00	
AK087209	0	0.00	
AK087218	1.295636	0.34	26.5%
AK087231	0.01579398	0.00	13.4%
AK087237	0.06818996	0.02	22.8%
AK087248	2.654352	0.33	12.5%
AK087264	0.0247047	0.01	51.5%
AK087278	0	0.00	

AK087290	0.23015922	0.20	87.8%
AK087292	0	0.00	
AK087295	0	0.00	
AK087313	0	0.00	
AK087338	0.01057362	0.02	147.6%
AK087340	0.03860916	0.02	59.9%
AK087351	0.03464306	0.05	144.2%
AK087376	0.5561528	0.16	28.3%
AK087382	0.02327942	0.02	94.4%
AK087392	0	0.00	
AK087393	0.3225472	0.15	45.0%
AK087460	0.00911406	0.01	144.7%
AK087464	0.6411252	0.23	35.3%
AK087483	4.501022	0.31	6.9%
AK087504	19.18276	0.66	3.4%
AK087529	0.8078542	0.10	12.1%
AK087569	0.0471807	0.02	44.4%
AK087581	0	0.00	
AK087604	1.80881	0.31	17.2%
AK087624	0.04499164	0.04	90.7%
AK087644	0.0945798	0.10	108.5%
AK087650	0.0053767	0.01	223.6%
AK087691	0.00724364	0.02	223.6%
AK087699	0	0.00	
AK087718	0.12409572	0.05	41.7%
AK087719	0	0.00	
AK087725	0.2487436	0.05	20.9%
AK087735	0.17341484	0.06	37.4%
AK087736	0	0.00	
AK087740	0.02496442	0.00	19.5%
AK087743	1.0429174	0.23	22.3%
AK087744	0	0.00	
AK087755	0.09753234	0.04	38.1%
AK087762	0.019436416	0.02	90.6%
AK087784	0.004157866	0.01	138.9%
AK087806	0.09702764	0.07	67.1%
AK087850	0.524502	0.18	34.3%
AK087886	0.0145466	0.02	137.1%
AK087905	5.812916	1.56	26.8%
AK088092	0.05983242	0.03	55.6%
AK088204	3.481848	0.29	8.2%
AK088368	0.0307587	0.02	77.6%
AK088579	0.0076361	0.02	223.6%
AK088734	0.06005364	0.03	52.7%
AK088743	0.08739926	0.04	40.4%
AK088822	0.02935776	0.01	34.6%
AK088877	0	0.00	

AK088880	0	0.00	
AK088897	2.963436	0.43	14.6%
AK088911	0.0025766	0.01	223.6%
AK088914	1.74349	0.18	10.0%
AK088920	5.337892	0.59	11.1%
AK088937	0	0.00	
AK088959	0.2106372	0.10	45.4%
AK088966	0.1731084	0.39	223.6%
AK089020	0	0.00	
AK089049	0	0.00	
AK089118	0.4184086	0.16	38.8%
AK089123	0.00894106	0.01	137.8%
AK089155	0.06496956	0.05	76.9%
AK089217	0.1001014	0.08	76.6%
AK089365	0.03909384	0.01	31.5%
AK089373	0.4065372	0.13	32.7%
AK089375	0.00492052	0.01	223.6%
AK089379	0.05094096	0.02	40.8%
AK089406	0.0031934	0.01	223.6%
AK089468	0	0.00	
AK089512	0.00825176	0.01	93.3%
AK089514	1.436886	0.19	13.2%
AK089519	0	0.00	
AK089615	0.0039491	0.01	223.6%
AK089801	0	0.00	
AK089914	0.0043421	0.01	223.6%
AK089981	0	0.00	
AK090016	1.3157754	1.30	98.5%
AK090048	0.04659164	0.02	47.2%
AK090061	0	0.00	
AK090072	0.4339692	0.18	41.7%
AK090150	0.0140792	0.02	140.0%
AK090163	0.162758	0.05	31.4%
AK090172	0.027159892	0.02	66.8%
AK090187	0.011989504	0.01	109.8%
AK090209	0	0.00	
AK090249	3.256132	0.44	13.5%
AK090251	0.00609836	0.01	223.6%
AK090261	2.750024	0.31	11.3%
AK090268	0.02388792	0.02	102.6%
AK090336	17.21426	0.71	4.1%
AK090337	0	0.00	
AK090359	0	0.00	
AK090372	0.02489468	0.02	94.4%
Ak1	52.29768	2.53	4.8%
AK122209	3.345404	0.25	7.6%
AK122507	7.136774	0.58	8.2%

AK122525	0.9762154	0.03	3.2%
AK129128	4.81156	0.13	2.7%
AK129160	1.1307564	0.31	27.2%
AK129302	11.78114	0.44	3.8%
AK129341	2.04608	0.22	10.9%
AK129421	3.982668	0.38	9.5%
AK131678	0.0578655	0.03	48.6%
AK131696	0	0.00	
AK131712	0.01387218	0.01	94.5%
AK131719	0.0311996	0.04	118.5%
AK131739	0.03978744	0.04	105.7%
AK131740	0	0.00	
AK131742	0	0.00	
AK131746	0.0251201	0.02	83.1%
AK131759	0	0.00	
AK131776	0	0.00	
AK131781	0	0.00	
AK131804	0	0.00	
AK131819	0.09052024	0.05	59.4%
AK131821	0.01706122	0.02	141.5%
AK131825	0.05356732	0.04	77.7%
AK131826	0	0.00	
AK131831	0.10139496	0.06	57.5%
AK131834	0.4646584	0.16	33.7%
AK131839	0.03375474	0.04	107.6%
AK131860	0.00714872	0.02	223.6%
AK131873	0.00867774	0.02	223.6%
AK131876	0	0.00	
AK131895	0.06722818	0.06	86.9%
AK131900	0.14244534	0.03	24.1%
AK131952	0.0059292	0.01	223.6%
AK132074	0	0.00	
AK132076	0.00839208	0.02	223.6%
AK132077	0.6686532	0.14	20.7%
AK132084	0	0.00	
AK132123	0.15886924	0.07	43.1%
AK132134	0	0.00	
AK132154	0.04533612	0.03	69.7%
AK132189	0.0273206	0.01	50.0%
AK132190	0	0.00	
AK132299	0.9331046	0.10	10.5%
AK132321	8.633654	0.76	8.8%
AK132380	0.009904478	0.01	68.0%
AK132440	0.3013116	0.07	23.0%
AK132460	2.487798	0.40	16.2%
AK132462	0	0.00	
AK132490	0.08504598	0.02	19.7%

AK132555	0.016214918	0.01	55.8%
AK132605	0	0.00	
AK132606	5.494612	0.29	5.3%
AK132630	0.951589	0.06	6.1%
AK132658	0	0.00	
AK132681	0.4209322	0.09	22.5%
AK132687	0	0.00	
AK132712	0.02514204	0.02	96.5%
AK132719	0	0.00	
AK132720	0.0605921	0.01	13.9%
AK132723	0.11091516	0.03	27.1%
AK132724	0.914972	0.47	51.5%
AK132729	0.032336	0.03	98.4%
AK132743	0	0.00	
AK132765	1.482596	0.47	31.9%
AK132798	0.1979628	0.02	9.9%
AK132828	0.0657842	0.05	72.9%
AK132829	0.3305596	0.21	64.6%
AK132836	0	0.00	
AK132847	0	0.00	
AK132865	0.01566732	0.04	223.6%
AK132872	0.00511034	0.01	223.6%
AK132885	1.1105376	0.18	15.9%
AK132898	0	0.00	
AK132902	0	0.00	
AK132929	0	0.00	
AK132930	0.2578356	0.10	39.3%
AK132940	0.00187102	0.00	223.6%
AK132955	1.121484	0.06	5.7%
AK132957	0	0.00	
AK132958	0	0.00	
AK132982	0.06173718	0.02	40.3%
AK133007	0	0.00	
AK133010	0.00417078	0.01	223.6%
AK133014	0	0.00	
AK133017	0.04456118	0.08	174.1%
AK133024	0.0054685	0.01	223.6%
AK133032	0	0.00	
AK133035	0.04156372	0.02	41.2%
AK133038	0.00291596	0.01	223.6%
AK133046	0.11240858	0.07	63.6%
AK133078	0	0.00	
AK133093	0	0.00	
AK133134	0.00476916	0.01	223.6%
AK133139	0.1779996	0.05	27.9%
AK133142	0.1122057	0.05	40.1%
AK133153	0.0755507	0.02	32.2%

AK133155	0.00254916	0.01	223.6%
AK133166	0.022412554	0.02	76.2%
AK133167	0.00608237	0.01	93.9%
AK133186	0	0.00	
AK133227	0.00912588	0.01	157.4%
AK133228	0.12462608	0.14	112.4%
AK133246	0.00291902	0.01	223.6%
AK133250	0.00224588	0.01	223.6%
AK133285	0.02040778	0.03	139.5%
AK133303	0	0.00	
AK133324	10.81998	0.44	4.0%
AK133329	1.780384	0.13	7.4%
AK133333	0.0145861	0.00	24.8%
AK133337	0	0.00	
AK133365	0	0.00	
AK133372	0	0.00	
AK133391	0.5000512	0.13	25.5%
AK133438	0.06829718	0.04	61.3%
AK133446	0	0.00	
AK133627	0.09056662	0.02	18.7%
AK133643	0.00849924	0.01	137.0%
AK133650	0.9213534	0.17	18.2%
AK133727	0	0.00	
AK133750	13.62624	1.08	7.9%
AK133793	0.00679648	0.01	137.0%
AK133807	0.10595812	0.04	33.8%
AK133808	2.157094	0.48	22.3%
AK133925	0.16391	0.05	29.4%
AK133929	0.02859728	0.01	30.8%
AK133987	0.137883	0.04	26.9%
AK134047	0.01070776	0.01	94.8%
AK134119	0	0.00	
AK134158	0	0.00	
AK134203	0.7859226	0.11	14.2%
AK134249	0.060141032	0.05	78.1%
AK134270	0.06575776	0.04	54.8%
AK134369	0.2140554	0.06	26.7%
AK134400	0.02155398	0.03	119.9%
AK134426	0.00497394	0.01	223.6%
AK134490	0.04437858	0.04	81.1%
AK134493	0.11961228	0.10	83.2%
AK134523	0.02183604	0.01	56.8%
AK134610	1.0531846	0.31	29.2%
AK134636	8.624988	0.35	4.0%
AK134637	1.401362	0.17	11.9%
AK134652	0	0.00	
AK134673	5.087982	0.55	10.9%

AK134703	0.09598214	0.03	33.1%
AK134715	0.2544856	0.09	35.6%
AK134717	0.3203012	0.09	28.3%
AK134749	0.14029316	0.03	24.1%
AK134755	0.12465696	0.03	24.3%
AK134769	5.442438	0.27	4.9%
AK134789	0.2212072	0.04	17.9%
AK134888	0.3426741	0.28	81.4%
AK134928	2.517512	0.21	8.2%
AK134933	0.3720078	0.05	13.6%
AK134962	1.40018	0.19	13.8%
AK135004	0.09820034	0.05	49.9%
AK135068	0.00471928	0.01	223.6%
AK135132	2.11998	0.30	14.3%
AK135254	0.293352	0.05	15.6%
AK135370	0.2315733	0.38	163.6%
AK135377	0	0.00	
AK135410	0.309642	0.13	40.4%
AK135429	0.00512304	0.01	138.9%
AK135471	0	0.00	
AK135487	2.798134	0.37	13.1%
AK135548	14.49264	1.25	8.6%
AK135576	0.3838798	0.17	45.2%
AK135599	0.35392824	0.69	193.7%
AK135643	0.1760834	0.05	26.7%
AK135671	0.00626266	0.01	140.3%
AK135675	0	0.00	
AK135688	0	0.00	
AK135691	0.01854202	0.02	94.1%
AK135703	0	0.00	
AK135708	0.0049137	0.01	137.7%
AK135715	0.194320143	0.31	157.6%
AK135721	1.339828	0.19	14.2%
AK135726	0	0.00	
AK135736	0.0051496	0.01	223.6%
AK135737	0	0.00	
AK135744	0	0.00	
AK135764	0	0.00	
AK135766	0	0.00	
AK135774	0	0.00	
AK135800	0	0.00	
AK135804	0	0.00	
AK135813	0.00869048	0.01	166.5%
AK135830	0	0.00	
AK135845	0.005537376	0.01	94.2%
AK135852	0.06686338	0.03	43.0%
AK135878	0	0.00	

AK135894	0.074861	0.02	22.9%
AK135904	0	0.00	
AK135932	0.1307825	0.06	44.9%
AK135939	0	0.00	
AK135960	0.00498592	0.01	223.6%
AK135963	0.02385728	0.02	89.6%
AK135973	0.00645054	0.01	138.3%
AK135975	0	0.00	
AK135986	0	0.00	
AK135992	0	0.00	
AK135997	8.075628	0.29	3.6%
AK136006	0	0.00	
AK136017	0	0.00	
AK136021	0	0.00	
AK136028	0	0.00	
AK136034	0	0.00	
AK136039	0.00856778	0.01	140.4%
AK136047	0	0.00	
AK136065	0	0.00	
AK136089	0.00640572	0.01	223.6%
AK136114	0.00476372	0.01	223.6%
AK136121	0	0.00	
AK136126	0	0.00	
AK136128	0.0127452	0.03	223.6%
AK136146	2.610654	0.29	10.9%
AK136154	0.00607122	0.01	223.6%
AK136164	0	0.00	
AK136178	0	0.00	
AK136239	0.00245846	0.01	223.6%
AK136254	0.156458	0.05	31.8%
AK136276	0.02543224	0.02	66.8%
AK136355	0.7117154	0.17	24.1%
AK136391	0.033378096	0.01	42.3%
AK136409	0	0.00	
AK136601	0.00651428	0.01	223.6%
AK136613	0.05258036	0.04	79.7%
AK136619	0.3913406	0.08	21.4%
AK136630	0	0.00	
AK136639	0	0.00	
AK136642	0	0.00	
AK136654	0.11596622	0.11	91.1%
AK136683	0	0.00	
AK136722	0.275822	0.07	26.2%
AK136730	0	0.00	
AK136742	2.103648	0.39	18.6%
AK136780	0.00808642	0.01	91.8%
AK136817	0.181323	0.03	19.3%

AK136844	22.88094	2.12	9.3%
AK136846	0	0.00	
AK136863	0.013845	0.02	168.1%
AK136877	0.00396698	0.01	223.6%
AK136882	0.14198696	0.03	21.8%
AK136919	0.6656414	0.10	15.2%
AK136927	0.2588548	0.05	21.1%
AK137010	0.1374317	0.06	41.1%
AK137033	0.6668704	0.14	21.3%
AK137099	6.669034	1.35	20.3%
AK137133	0.03915772	0.01	34.0%
AK137157	1.1992222	0.28	23.2%
AK137190	0	0.00	
AK137192	0.04363518	0.03	62.2%
AK137243	0	0.00	
AK137251	3.165758	0.40	12.6%
AK137304	4.674076	0.58	12.5%
AK137341	0	0.00	
AK137370	0.3150924	0.07	22.7%
AK137397	0.5248194	0.15	28.9%
AK137428	0.359758	0.04	10.9%
AK137448	0	0.00	
AK137473	0.4206172	0.15	34.8%
AK137492	0.12451624	0.03	20.9%
AK137525	0	0.00	
AK137536	0.01672732	0.02	129.7%
AK137548	2.79701	0.22	8.0%
AK137552	0.00350814	0.01	223.6%
AK137583	1.394152	0.19	13.7%
AK137696	0	0.00	
AK137735	0	0.00	
AK137923	0.355992	0.20	56.3%
AK138088	0.02898416	0.00	15.2%
AK138089	0	0.00	
AK138135	0.077051304	0.06	77.4%
AK138161	0.42435	0.09	21.9%
AK138201	0.02692226	0.02	57.0%
AK138212	0	0.00	
AK138215	0.0240795	0.03	139.2%
AK138232	0.0165007	0.01	31.2%
AK138248	0.2518528	0.08	33.7%
AK138253	3.284052	0.25	7.7%
AK138287	0.383601	0.07	17.4%
AK138291	0.06663726	0.04	64.7%
AK138296	1.331916	0.22	16.9%
AK138305	0	0.00	
AK138307	0.04671514	0.02	42.3%

AK138323	0.03197682	0.04	126.5%
AK138328	0.6045982	0.19	31.5%
AK138343	0	0.00	
AK138346	0.2155256	0.07	30.4%
AK138348	0.0049667	0.01	223.6%
AK138371	0.01197418	0.02	137.0%
AK138376	0	0.00	
AK138383	1.1075846	0.15	13.3%
AK138393	0.08749168	0.02	26.1%
AK138412	29.00324	0.80	2.8%
AK138421	0.01912832	0.02	114.0%
AK138450	0.01117658	0.02	223.6%
AK138451	0.04314306	0.03	59.5%
AK138462	0.0075179	0.02	223.6%
AK138476	0.2281562	0.06	26.5%
AK138505	0.1579118	0.06	40.0%
AK138521	0.0311506	0.02	75.5%
AK138522	0.63588036	0.36	56.2%
AK138532	3.693338	0.70	18.9%
AK138554	0.0885303	0.09	106.3%
AK138592	0.10134774	0.04	34.5%
AK138624	0	0.00	
AK138658	0.08346698	0.02	27.5%
AK138660	0	0.00	
AK138667	0.0175497	0.01	58.0%
AK138691	0	0.00	
AK138755	0.132549	0.07	55.7%
AK138756	0.4249996	0.16	38.3%
AK138781	0.4399898	0.16	35.7%
AK138792	0	0.00	
AK138811	0	0.00	
AK138842	0	0.00	
AK138849	0.003648602	0.01	140.7%
AK138853	0	0.00	
AK138907	0.16077148	0.09	59.1%
AK138915	0.4845368	0.04	7.8%
AK138921	0.00216408	0.00	223.6%
AK138941	0	0.00	
AK138949	47.26384	2.17	4.6%
AK139027	0.0191969	0.01	58.0%
AK139053	0.285548	0.17	59.3%
AK139063	0.0149747	0.02	138.9%
AK139070	0.416628	0.06	14.6%
AK139076	0.7902728	0.20	25.1%
AK139092	0.010760104	0.01	134.3%
AK139109	0.9341194	0.23	25.0%
AK139149	0	0.00	

AK139182	24.35908	1.71	7.0%
AK139229	0.08170548	0.04	43.9%
AK139280	0.44072974	0.57	129.2%
AK139295	1.433116	0.12	8.6%
AK139319	3.673398	0.57	15.6%
AK139359	0.03995826	0.03	75.5%
AK139424	0.13760824	0.06	45.9%
AK139461	0.3415238	0.10	28.3%
AK139481	2.940252	0.25	8.6%
AK139483	1.0166586	0.05	4.4%
AK139487	0	0.00	
AK139502	0	0.00	
AK139503	0.00622964	0.01	223.6%
AK139522	0	0.00	
AK139523	0.8514796	0.14	15.9%
AK139528	0	0.00	
AK139544	0	0.00	
AK139551	0	0.00	
AK139560	0	0.00	
AK139587	0.09478792	0.07	72.7%
AK139591	0	0.00	
AK139607	0	0.00	
AK139611	0.0712794	0.03	46.7%
AK139614	0.007497	0.01	138.1%
AK139634	0.2196914	0.04	19.4%
AK139678	0.008233	0.02	223.6%
AK139687	0.00841454	0.01	137.0%
AK139698	0.00967696	0.01	153.1%
AK139701	0.009879992	0.01	105.1%
AK139730	0.003365284	0.00	121.8%
AK139752	0	0.00	
AK139772	0	0.00	
AK139774	0.00680342	0.02	223.6%
AK139799	0.08284182	0.02	23.1%
AK139806	0	0.00	
AK139810	0.00745244	0.01	92.9%
AK139822	0.009493832	0.01	114.1%
AK139842	0.0145757	0.02	137.7%
AK139864	0	0.00	
AK139882	0	0.00	
AK139889	0.01623084	0.04	223.6%
AK139890	0.00483004	0.01	223.6%
AK139997	0.4725456	0.09	19.5%
AK139999	1.3812516	0.66	47.8%
AK140017	0	0.00	
AK140049	0.02496538	0.02	92.4%
AK140072	0.5543398	0.09	17.0%

AK140089	0.1154485	0.03	27.1%
AK140090	0.2126274	0.04	16.7%
AK140147	0.04831806	0.02	46.8%
AK140161	0.00866534	0.01	140.4%
AK140178	0.4558954	0.06	12.5%
AK140186	36.35238	1.57	4.3%
AK140192	0.2328094	0.05	22.2%
AK140218	2.029774	0.37	18.2%
AK140221	1.56488	0.21	13.7%
AK140224	7.835494	0.49	6.3%
AK140234	0.1499198	0.03	20.5%
AK140238	0.0685016	0.03	38.5%
AK140265	1064.1324	164.35	15.4%
AK140288	1.863114	0.11	6.0%
AK140292	0.032854454	0.02	73.3%
AK140336	0.5072128	0.05	9.3%
AK140363	0.007954	0.01	138.9%
AK140370	2.102494	0.69	32.9%
AK140384	0.433269	0.10	23.6%
AK140390	0.7602158	0.11	14.8%
AK140437	0.20106254	0.07	33.1%
AK140446	2.238282	0.31	14.0%
AK140479	0.03958446	0.03	66.5%
AK140520	0.07187218	0.03	47.9%
AK140543	0.17707464	0.09	49.9%
AK140547	12.51586	1.13	9.1%
AK140580	0.02607344	0.02	80.6%
AK140583	0.00271224	0.01	223.6%
AK140632	1.961768	0.13	6.6%
AK140725	5.232246	0.77	14.7%
AK140772	0.2521668	0.05	19.9%
AK140896	0.01714204	0.02	104.6%
AK140919	0.06269588	0.03	48.7%
AK141015	0	0.00	
AK141151	0.00179353	0.00	223.6%
AK141187	0.013160016	0.01	80.8%
AK141205	0.10810154	0.04	38.6%
AK141227	1.0943606	0.25	23.2%
AK141238	1.719264	0.25	14.3%
AK141303	0.12187406	0.07	59.0%
AK141396	0	0.00	
AK141402	17.8335	1.74	9.8%
AK141411	1.520994	0.26	17.3%
AK141527	0.2166072	0.06	28.2%
AK141540	0.013964	0.01	68.0%
AK141565	0.09336402	0.04	46.4%
AK141570	8.896188	0.51	5.7%

AK141588	3.629022	0.49	13.6%
AK141638	1.44179	0.15	10.6%
AK141659	0.2377488	0.06	23.1%
AK141725	0.1560602	0.01	9.0%
AK141748	0	0.00	
AK141842	11.91354	0.98	8.2%
AK141900	0.07562304	0.05	71.5%
AK141935	0.3476802	0.13	38.6%
AK142001	0.051302	0.02	43.1%
AK142028	0.5034444	0.21	41.0%
AK142058	0.05425074	0.04	72.2%
AK142074	0	0.00	
AK142089	0.18613186	0.09	50.8%
AK142161	0.1919824	0.11	55.0%
AK142198	0.809443	0.15	18.6%
AK142214	0.344965	0.10	28.5%
AK142244	1.1089322	0.20	17.6%
AK142254	0.08657142	0.05	61.4%
AK142359	0.15369614	0.04	25.3%
AK142386	1.0383062	0.18	17.6%
AK142406	0.00473336	0.01	138.2%
AK142431	1.82661	4.08	223.6%
AK142440	2.764528	0.26	9.3%
AK142467	0.0398532	0.02	62.0%
AK142524	0.00303728	0.01	223.6%
AK142593	1.0673668	0.20	18.6%
AK142602	0	0.00	
AK142614	0.02642772	0.02	81.0%
AK142627	0	0.00	
AK142664	0	0.00	
AK142678	0	0.00	
AK142681	0.9296148	0.07	7.2%
AK142847	0.0162049	0.04	223.6%
AK142879	0.2363736	0.03	14.3%
AK142892	0.10517748	0.05	44.0%
AK142949	0	0.00	
AK142977	0.00583532	0.01	137.8%
AK142978	0.13699954	0.09	63.1%
AK142999	0.1690424	0.20	118.6%
AK143097	0.3535722	0.04	12.5%
AK143102	0	0.00	
AK143160	0	0.00	
AK143195	0	0.00	
AK143234	3.50042	0.41	11.7%
AK143260	0.0914017	0.04	44.5%
AK143305	0.00453668	0.01	223.6%
AK143407	15.21126	0.76	5.0%

AK143417	0.559435	0.51	90.7%
AK143610	4.09012	0.62	15.3%
AK143758	0	0.00	
AK143760	0	0.00	
AK143784	0.03723966	0.01	19.8%
AK143789	0	0.00	
AK143813	0	0.00	
AK143833	0	0.00	
AK143856	0.00449108	0.01	137.0%
AK143902	0.01819046	0.00	25.6%
AK143924	0	0.00	
AK143926	0.3302612	0.18	54.7%
AK143933	0	0.00	
AK143934	0	0.00	
AK143952	0	0.00	
AK143957	0.0605165	0.05	85.0%
AK143961	0	0.00	
AK143968	0.07071028	0.02	23.1%
AK143982	0.34099646	0.26	75.6%
AK144055	0	0.00	
AK144087	0	0.00	
AK144104	0	0.00	
AK144120	0.00821508	0.02	223.6%
AK144143	0.01782498	0.02	103.9%
AK144172	0.00963206	0.02	223.6%
AK144265	31.81212	9.13	28.7%
AK144266	0.02814174	0.03	103.7%
AK144267	0.19644548	0.10	52.2%
AK144274	0.00591886	0.01	223.6%
AK144295	0.0778465	0.04	57.0%
AK144297	0.03699032	0.05	138.7%
AK144299	0.07657285	0.05	61.6%
AK144317	0.8516628	0.06	7.1%
AK144329	0	0.00	
AK144330	0.0336784	0.03	102.0%
AK144366	0	0.00	
AK144367	0.6774776	0.16	23.8%
AK144388	0	0.00	
AK144462	0	0.00	
AK144477	0.01197246	0.03	223.6%
AK144485	0.5491064	0.11	20.2%
AK144536	13.50954	1.13	8.4%
AK144545	0.193104	0.05	28.0%
AK144563	0.004544142	0.01	140.5%
AK144571	0	0.00	
AK144589	0.3550638	0.05	14.3%
AK144610	0.0354212	0.03	97.3%

AK144617	0.00489754	0.01	223.6%
AK144626	0	0.00	
AK144632	0.01635298	0.02	93.0%
AK144662	0.3500366	0.16	45.4%
AK144664	0.4482002	0.14	30.8%
AK144695	0.07831892	0.04	45.5%
AK144724	0.00447832	0.01	223.6%
AK144747	0.00309056	0.01	223.6%
AK144771	0.189731	0.05	27.6%
AK144776	0	0.00	
AK144798	0	0.00	
AK144811	0.08791628	0.05	53.4%
AK144823	0.00234904	0.01	223.6%
AK144829	0.01330294	0.02	114.0%
AK144834	0	0.00	
AK144843	0	0.00	
AK144852	0.0075017	0.01	138.9%
AK144859	0.4213264	0.23	55.0%
AK144862	0	0.00	
AK144867	3.68875	0.69	18.6%
AK144870	0.1929012	0.09	46.4%
AK144882	0	0.00	
AK144886	0.00767532	0.02	223.6%
AK144897	0.09069354	0.06	69.9%
AK144909	0.5179844	0.19	37.3%
AK144910	0	0.00	
AK144912	0	0.00	
AK144923	0.04951848	0.01	29.2%
AK144927	0.003323448	0.00	142.7%
AK144984	0	0.00	
AK145079	0.8903326	0.07	7.4%
AK145081	0.00475954	0.01	223.6%
AK145095	1.0227894	0.39	38.1%
AK145113	0.02005358	0.04	179.8%
AK145129	0	0.00	
AK145139	0	0.00	
AK145144	0.0061821	0.01	223.6%
AK145161	0.07568382	0.04	53.4%
AK145172	0	0.00	
AK145180	0.236718	0.11	48.1%
AK145215	0	0.00	
AK145218	0	0.00	
AK145231	0.0991894	0.03	26.4%
AK145253	0.00467124	0.01	223.6%
AK145276	0.034382	0.02	47.5%
AK145301	0.11473762	0.09	81.7%
AK145303	0.00808254	0.02	223.6%

AK145307	0	0.00	
AK145310	0.02606284	0.04	137.1%
AK145334	0.00280774	0.01	223.6%
AK145340	0	0.00	
AK145347	0	0.00	
AK145360	0	0.00	
AK145370	0	0.00	
AK145393	2.341498	0.33	14.1%
AK145395	0.04054432	0.03	78.3%
AK145396	0.4605618	0.21	45.7%
AK145430	0.3186818	0.16	50.9%
AK145433	0	0.00	
AK145464	0.0196454	0.01	50.0%
AK145481	0	0.00	
AK145489	0.4985758	0.11	23.0%
AK145533	0.01959044	0.02	92.4%
AK145544	0	0.00	
AK145597	0	0.00	
AK145614	0.0375663	0.02	45.6%
AK145828	0.00945838	0.02	223.6%
AK146012	2.94744	0.82	27.8%
AK146025	0	0.00	
AK146139	0	0.00	
AK146146	0.01701978	0.04	223.6%
AK146150	0	0.00	
AK146156	0.07794604	0.05	58.7%
AK146160	0.01691766	0.02	139.3%
AK146183	0	0.00	
AK146213	0	0.00	
AK146230	0.12338314	0.04	31.4%
AK146255	0	0.00	
AK146381	0.0056161	0.01	138.2%
AK146386	0	0.00	
AK146407	0.10428582	0.07	70.0%
AK146690	0.12679602	0.04	32.9%
AK146694	0.00799646	0.01	161.4%
AK146748	0	0.00	
AK146861	0	0.00	
AK146888	0	0.00	
AK146937	0.00912154	0.01	138.2%
AK146979	0	0.00	
AK147021	0.03078422	0.03	93.8%
AK147070	0	0.00	
AK147071	0	0.00	
AK147079	0	0.00	
AK147121	1.959038	0.10	5.3%
AK147193	0.02460046	0.04	172.7%

AK147443	0.06286062	0.02	34.0%
AK147532	4.847846	0.08	1.6%
AK147796	1.612256	0.15	9.5%
AK147860	0.771305	0.07	9.7%
AK147911	6.947314	0.26	3.8%
AK147993	2.049536	0.13	6.5%
AK148045	0.1030984	0.23	223.6%
AK148054	5.2969	0.84	15.8%
AK148126	1.402502	0.27	19.1%
AK148130	0	0.00	
AK148198	0.00386798	0.01	223.6%
AK148282	0.594789	0.13	21.0%
AK148294	2.363528	0.53	22.6%
AK148297	0.03568158	0.01	16.1%
AK148313	0.13735554	0.05	34.0%
AK148321	0.02719186	0.02	83.0%
AK148373	0.1886637	0.09	49.6%
AK148391	0.1499251	0.12	79.8%
AK148393	0	0.00	
AK148461	3.971266	0.39	9.9%
AK148766	0.342007	0.13	38.7%
AK148800	0.06718016	0.04	60.5%
AK149000	0.0459672	0.01	32.3%
AK149094	9.12799	1.52	16.7%
AK149188	0.1959322	0.04	21.4%
AK149205	0.2604842	0.09	35.0%
AK149240	0.06870706	0.03	49.6%
AK149260	0.00254644	0.01	223.6%
AK149295	0.3495134	0.08	22.0%
AK149307	0.6271056	0.09	14.9%
AK149321	0.01504008	0.01	68.8%
AK149394	0	0.00	
AK149453	0.03905352	0.03	67.8%
AK149454	0	0.00	
AK149509	0	0.00	
AK149537	0.00288162	0.01	223.6%
AK149842	0.0488658	0.02	50.0%
AK149987	0.7414604	0.21	28.5%
AK150110	0.2687686	0.06	23.2%
AK150172	0.14176662	0.08	54.5%
AK150199	0.051673794	0.03	58.6%
AK150559	7.37185	0.73	9.8%
AK151508	0.5859458	0.06	11.0%
AK151523	0	0.00	
AK151815	0.005269951	0.01	193.1%
AK152002	10.023136	1.08	10.8%
AK152097	11.30854	0.53	4.7%

AK152437	0.2215848	0.08	34.6%
AK153014	0.07562428	0.03	41.6%
AK153180	0	0.00	
AK153212	0	0.00	
AK153351	0	0.00	
AK153427	0.03042238	0.01	35.5%
AK153491	0.02233296	0.03	137.0%
AK153503	1.87915	0.31	16.8%
AK153534	0.01851716	0.01	50.5%
AK153575	0.14269934	0.10	71.5%
AK153584	0	0.00	
AK153590	0.0390698	0.01	33.3%
AK153639	0.00419124	0.01	223.6%
AK153786	0	0.00	
AK153821	0.12635582	0.05	38.1%
AK153828	0	0.00	
AK153849	5.497806	0.35	6.3%
AK153988	0.1274023	0.04	30.8%
AK154034	0.2070848	0.16	75.9%
AK154077	0.4826628	0.19	39.7%
AK154113	0.5647518	0.11	19.5%
AK154177	0.10062932	0.01	12.8%
AK154184	0	0.00	
AK154190	0.336446	0.10	30.8%
AK154211	0.1116478	0.09	84.8%
AK154225	1.541062	0.08	4.9%
AK154275	0.3070704	0.03	10.1%
AK154380	3.008374	0.24	8.1%
AK154406	0.03996038	0.04	91.0%
AK154427	0.51398	0.12	23.0%
AK154515	0.004000716	0.01	143.5%
AK154552	0.001323588	0.00	223.6%
AK154587	0.00807118	0.02	223.6%
AK154631	0	0.00	
AK154635	0	0.00	
AK154735	0	0.00	
AK154977	0.009665494	0.01	83.2%
AK154990	0.009904616	0.01	74.7%
AK155068	0	0.00	
AK155141	33.9895	1.18	3.5%
AK155149	0.0305367	0.02	75.8%
AK155169	0	0.00	
AK155239	2.856016	0.15	5.4%
AK155472	0.5178754	0.27	52.3%
AK155489	0.00964542	0.02	223.6%
AK155516	0	0.00	
AK155560	0.0036118	0.01	223.6%

AK155592	0.2571632	0.06	23.8%
AK155734	0.001964928	0.00	223.6%
AK155839	0.01247904	0.02	145.7%
AK155877	0	0.00	
AK156148	0	0.00	
AK156292	0	0.00	
AK156308	0.00231356	0.01	223.6%
AK156427	0.02065578	0.01	64.9%
AK156452	0.00317256	0.01	223.6%
AK156477	1.0428424	0.14	13.7%
AK156636	0.5979292	0.12	20.4%
AK156678	0	0.00	
AK156750	0.3329254	0.11	33.8%
AK156771	0.04434958	0.01	24.4%
AK156842	1.767142	0.17	9.4%
AK156859	0	0.00	
AK156902	0	0.00	
AK156967	0	0.00	
AK156980	0	0.00	
AK157178	46.22424	10.19	22.0%
AK157216	0	0.00	
AK157218	0.007191696	0.01	121.5%
AK157237	0.17404608	0.16	89.9%
AK157435	0.06674662	0.03	47.2%
AK157456	0.00924204	0.01	137.0%
AK157499	5.393056	0.80	14.8%
AK157519	0.6269654	0.10	15.8%
AK157531	0	0.00	
AK157632	0	0.00	
AK157659	0.2311694	0.07	31.5%
AK157788	0.1706584	0.04	21.7%
AK157795	0.6400372	0.12	18.7%
AK157878	0.200532	0.05	23.4%
AK157887	1.1655316	0.14	12.2%
AK157916	0.02038404	0.02	80.9%
AK157941	0.009399542	0.01	97.8%
AK157947	0.09411882	0.04	40.3%
AK157963	20.4631	2.15	10.5%
AK157980	0.00533268	0.01	223.6%
AK158018	0.003798	0.01	223.6%
AK158030	17.59938	0.93	5.3%
AK158034	0.009223326	0.01	68.2%
AK158055	0.23847	0.14	58.3%
AK158109	0.170837648	0.25	146.5%
AK158152	0.0215439	0.00	18.6%
AK158157	0.00348014	0.01	223.6%
AK158189	0.3963292	0.12	31.4%

AK158196	0.00787781	0.01	112.4%
AK158233	0	0.00	
AK158239	0	0.00	
AK158244	0.194549635	0.20	100.6%
AK158291	0.01799452	0.02	117.9%
AK158295	0.0075474	0.02	223.6%
AK158300	21.6709	3.35	15.5%
AK158379	0.4354582	0.09	21.7%
AK158414	0.866335	0.17	19.1%
AK158434	0.07116026	0.04	58.7%
AK158473	0.00677372	0.00	59.3%
AK158482	0.019908598	0.01	72.6%
AK158612	0.00504096	0.01	137.0%
AK158619	1.63803	0.27	16.3%
AK158658	0.00583862	0.01	223.6%
AK158669	0.9537566	0.42	43.9%
AK158810	0	0.00	
AK158854	0.288044	0.05	16.6%
AK158926	0.1265236	0.03	22.4%
AK158929	0.14855546	0.06	41.9%
AK158974	0	0.00	
AK159003	0.04753326	0.01	25.9%
AK159010	0	0.00	
AK159017	0.04614294	0.04	79.5%
AK159035	0.01321462	0.02	148.0%
AK160141	0.2535274	0.09	36.5%
AK160158	0	0.00	
AK160260	0	0.00	
AK160359	0.12064126	0.06	48.9%
AK160408	0.3740706	0.16	44.0%
AK160416	0	0.00	
AK160425	0	0.00	
AK160437	0	0.00	
AK160502	0.3197036	0.08	23.7%
AK160548	2.601732	0.55	21.2%
AK160668	2.782768	0.35	12.6%
AK160711	1.314262	0.08	6.4%
AK160850	0.3762666	0.09	23.1%
AK160921	0.1986468	0.04	19.1%
AK160964	0.00855969	0.01	90.2%
AK161046	0.06209914	0.02	25.2%
AK161073	0.1803654	0.06	34.0%
AK161079	0.0238208	0.01	51.0%
AK161191	0	0.00	
AK161337	0.077941	0.12	150.7%
AK161354	0.00821234	0.02	223.6%
AK161361	0.28800228	0.13	44.8%

AK161362	0.00546956	0.01	138.3%
AK161363	20.94428	1.15	5.5%
AK161373	0	0.00	
AK161402	0	0.00	
AK161406	0.03573934	0.01	35.6%
AK161423	5.617354	0.38	6.7%
AK161424	2.699486	0.25	9.4%
AK161431	0.001589104	0.00	223.6%
AK161459	0.15781816	0.05	33.9%
AK161463	0.03800584	0.02	60.2%
AK161480	0.00373598	0.01	223.6%
AK161481	0.105243098	0.14	132.8%
AK161589	0	0.00	
AK161590	0.2396572	0.19	78.4%
AK161599	0.8704206	0.53	60.7%
AK161639	0.03992308	0.05	115.5%
AK161717	0.017304866	0.01	32.1%
AK161769	1.0742732	0.12	11.0%
AK161843	7.302128	0.63	8.7%
AK161845	0.014032496	0.02	115.5%
AK161900	1.0425752	0.18	16.9%
AK161948	0.02585694	0.01	43.8%
AK162010	0.1509546	0.02	13.7%
AK162043	1.584374	0.39	24.6%
AK162063	0.05172588	0.03	50.2%
AK162128	4.225332	0.42	10.0%
AK162138	0.6897716	0.11	15.8%
AK162140	0	0.00	
AK162351	0.258675	0.03	12.3%
AK162363	0.1710208	0.06	32.8%
AK162386	0.3253838	0.05	14.6%
AK162388	0	0.00	
AK162519	0.11347208	0.03	27.8%
AK162599	0.0068384	0.02	223.6%
AK162636	0.3130469	0.26	83.5%
AK162666	0	0.00	
AK162670	4.321318	0.29	6.7%
AK162774	0.00283996	0.01	223.6%
AK162806	0.0648187	0.04	57.9%
AK162833	0	0.00	
AK162870	0.380984	0.03	7.0%
AK162906	0.501005204	0.38	76.3%
AK162911	0.07700832	0.02	21.2%
AK162916	0.02841702	0.01	30.1%
AK162930	0.4095164	0.15	36.5%
AK162942	0.2765052	0.08	29.5%
AK162963	0.0153835	0.03	223.6%

AK162965	0.2078448	0.05	25.4%
AK163002	0	0.00	
AK163016	0.17863	0.03	14.4%
AK163020	0.08399108	0.02	23.0%
AK163054	0.005186894	0.01	221.7%
AK163067	0.208481	0.05	22.1%
AK163075	0.05682784	0.03	58.0%
AK163095	11.19368	0.61	5.4%
AK163097	0.1695572	0.01	7.6%
AK163099	0.8754556	0.23	26.3%
AK163103	0.00934678	0.02	223.6%
AK163121	9.85859	0.82	8.3%
AK163132	0.2974662	0.10	34.6%
AK163149	2.859846	0.31	10.8%
AK163151	0.00594814	0.01	223.6%
AK163160	0.21300194	0.07	32.1%
AK163166	0.16491218	0.08	48.3%
AK163212	0	0.00	
AK163252	0.2104158	0.10	48.2%
AK163289	0.001783478	0.00	223.6%
AK163292	0.0134429	0.03	223.6%
AK163309	0	0.00	
AK163331	0	0.00	
AK163333	0	0.00	
AK163421	0.607183	0.11	18.3%
AK163429	3.839576	0.49	12.7%
AK163433	6.130286	0.54	8.8%
AK163440	2988.508	381.52	12.8%
AK163447	0.13160114	0.05	41.0%
AK163460	0.2512694	0.11	42.7%
AK163475	5.26098	0.58	11.1%
AK163488	4.089752	0.74	18.0%
AK163556	1.958908	0.07	3.7%
AK163572	0.3100624	0.05	17.1%
AK163592	2.347642	0.37	15.7%
AK163667	0.05132756	0.07	134.8%
AK163681	0	0.00	
AK163710	1.778952	0.44	24.6%
AK163711	0.1158643	0.05	44.9%
AK163747	2.96814	0.66	22.3%
AK163775	0.1314902	0.08	57.8%
AK163827	0.183043587	0.17	92.7%
AK163997	0.01290722	0.01	59.5%
AK164069	0.1513696	0.04	27.8%
AK164083	2.716072	0.39	14.3%
AK164098	0.2260272	0.08	33.7%
AK164124	0.5233842	0.04	8.0%

AK164126	7.124296	0.50	7.1%
AK164180	0.06120654	0.03	51.4%
AK164218	0.02377548	0.03	140.3%
AK164256	1.16554	0.13	11.3%
AK164315	0.259877	0.12	44.5%
AK164331	1.8968	0.42	22.0%
AK164362	0.0118547	0.01	94.8%
AK164420	0.016661252	0.01	83.9%
AK164583	0.1823609	0.11	62.5%
AK164611	0.2897762	0.06	20.7%
AK164650	0.39130464	0.28	71.3%
AK164741	0.010513346	0.01	73.5%
AK164742	0.05321476	0.02	37.2%
AK164779	0.1776542	0.05	26.3%
AK164829	0.01252998	0.01	64.5%
AK164836	0.2228802	0.05	22.8%
AK164875	20.12512	0.77	3.8%
AK164928	2.266	0.54	23.6%
AK164976	0.4499468	0.15	32.8%
AK165089	0.00525106	0.01	137.0%
AK165120	0.12370678	0.05	44.3%
AK165129	25.46728	1.30	5.1%
AK165297	0	0.00	
AK165329	0.04780576	0.06	118.0%
AK165338	0.0060344	0.01	223.6%
AK165366	0.0076521	0.01	138.6%
AK165443	0	0.00	
AK165489	0.02640986	0.02	92.2%
AK165532	0.1668508	0.17	100.4%
AK165607	0.0057427	0.01	223.6%
AK165697	0.0040802	0.01	137.0%
AK165766	0.04046764	0.02	46.5%
AK165776	0.08654172	0.07	77.0%
AK165802	0.4124278	0.13	32.0%
AK165804	0	0.00	
AK165817	13.6723	2.17	15.9%
AK165835	0.00527032	0.01	223.6%
AK165854	0	0.00	
AK165876	0	0.00	
AK165889	5.347472	0.30	5.7%
AK166041	0	0.00	
AK166044	0.03288202	0.02	52.3%
AK166060	0.0022217	0.00	223.6%
AK166079	0.3024004	0.12	40.6%
AK166159	0.00686454	0.01	137.0%
AK166274	0	0.00	
AK166336	0.00512152	0.01	162.9%

AK166404	0.12230736	0.09	73.2%
AK166420	0.07489844	0.04	59.0%
AK166453	36.47514	3.47	9.5%
AK166469	1.1599914	0.23	20.2%
AK166513	2.068248	0.11	5.3%
AK166554	0	0.00	
AK166556	0	0.00	
AK166708	0.0298803	0.02	65.1%
AK166773	1.0184272	0.08	8.1%
AK166824	0.00566534	0.01	223.6%
AK166895	0.01927926	0.01	50.4%
AK167137	0.02683392	0.01	22.3%
AK167556	0.0095943	0.01	92.7%
AK167606	0.4960056	0.08	16.6%
AK167957	0	0.00	
AK168184	0.04518182	0.01	30.7%
AK168746	0.04223162	0.02	52.6%
AK168860	0.0394266	0.02	59.0%
AK169220	0.00668834	0.01	137.6%
AK169479	0	0.00	
AK169482	0.15505478	0.07	43.0%
AK169506	0	0.00	
AK169507	0.00943099	0.00	11.2%
AK169509	0	0.00	
AK169510	0.06608104	0.03	51.6%
AK169574	0.185713	0.04	19.8%
AK169739	0.3565112	0.10	28.7%
AK169777	14.91664	0.70	4.7%
AK169807	0.006877876	0.01	159.6%
AK169831	9.811202	0.61	6.3%
AK169894	3.994368	0.75	18.7%
AK169954	1.3180238	0.34	25.8%
AK169958	0.0592527	0.01	25.1%
AK170078	1.6224364	0.44	26.8%
AK170106	0.01770222	0.01	33.6%
AK170112	24.3375	1.60	6.6%
AK170194	0.03574362	0.01	32.8%
AK170206	0.003156	0.01	223.6%
AK170301	0.00580918	0.01	139.0%
AK170353	0.001945766	0.00	223.6%
AK170409	0.00473802	0.01	223.6%
AK170464	0	0.00	
AK170612	0.010537	0.02	223.6%
AK170805	42.86576	2.51	5.9%
AK170837	0.21187196	0.16	75.8%
AK170868	0.00233608	0.01	223.6%
AK170928	0.1723826	0.02	12.7%

AK171116	0.002847686	0.00	111.3%
AK171117	0.0457026	0.03	65.4%
AK171153	0	0.00	
AK171159	3.361336	0.49	14.7%
AK171183	0.1691	0.05	29.1%
AK171382	0.14553764	0.05	35.7%
AK171408	0	0.00	
AK171764	0.01816748	0.02	100.3%
AK171817	1.422096	0.11	7.5%
AK171836	0.1718932	0.03	16.9%
AK171902	1.1255988	0.34	30.5%
AK171952	9.788048	1.08	11.1%
AK171953	0.00357826	0.01	223.6%
AK171960	11.77422	0.58	5.0%
AK171985	1.313499	0.41	31.3%
AK172090	0.3007058	0.04	13.7%
AK172139	1.551538	0.20	12.7%
AK172190	0.168319	0.03	19.2%
AK172315	0.10815026	0.07	61.2%
AK172386	0	0.00	
AK172428	1.226766	0.13	10.6%
AK172452	0	0.00	
AK172459	0.01908647	0.01	71.2%
AK172630	0	0.00	
AK172672	0.3008256	0.06	19.5%
AK172678	0.12501506	0.19	152.3%
AK172683	0	0.00	
AK172704	0.20499612	0.27	130.2%
AK172713	0.02748774	0.01	51.7%
AK173072	8.749388	1.07	12.2%
AK177174	2.251522	0.57	25.2%
AK177718	0.5766524	0.22	38.9%
AK177731	6.876734	0.43	6.3%
AK177749	0.4603424	0.26	56.2%
AK177772	3.330528	0.47	14.1%
AK178082	0	0.00	
AK178359	2.7654132	2.16	78.2%
AK178429	8.580744	1.65	19.3%
AK178562	0.7040864	0.34	47.8%
AK178706	6.079762	0.24	4.0%
AK179413	0.8445722	0.23	27.7%
AK179731	0	0.00	
AK179786	0.1948852	0.06	33.0%
AK180240	31.9103	1.86	5.8%
AK180382	0.3400166	0.16	47.8%
AK180697	5.721148	0.75	13.1%
AK180712	1.61178	0.40	25.0%

AK180825	0	0.00	
AK180911	0.5419074	0.27	49.4%
AK180939	0.06459586	0.06	92.8%
AK181631	0	0.00	
AK181830	1.471843	0.61	41.6%
AK181896	3.0459166	6.60	216.5%
AK181982	1.0138518	0.38	37.4%
AK182361	0.627501	0.29	47.0%
AK182695	3.028416	1.00	32.9%
AK182874	4.493668	0.63	14.0%
AK182887	5.124464	0.74	14.5%
AK183134	0.6114618	0.14	23.0%
AK183258	0	0.00	
AK183279	0	0.00	
AK183458	4.891632	1.08	22.1%
AK183986	0	0.00	
AK184121	0.005628374	0.00	71.6%
AK184225	3.398994	0.69	20.3%
AK184603	1.4090362	0.49	34.8%
AK184713	0.11789622	0.11	90.2%
AK184873	0.0206876	0.05	223.6%
AK185421	0.4677646	0.13	28.3%
AK185530	2.481758	0.77	31.0%
AK185549	0.0433016	0.10	223.6%
AK185681	77.08938	5.46	7.1%
AK185715	0.5401396	0.34	62.2%
AK185737	18.45072	2.82	15.3%
AK185785	0.37685252	0.27	72.6%
AK185798	0.1176362	0.16	137.5%
AK185910	0	0.00	
AK186650	0	0.00	
AK187054	139.691	6.23	4.5%
AK187371	1.3126716	0.26	19.6%
AK187594	0.52455	0.33	63.3%
AK188320	1.537116	0.37	24.1%
AK188430	5.364138	1.39	26.0%
AK188772	0.8638582	0.30	35.1%
AK188812	0.4560132	0.22	48.4%
AK188991	0.274344	0.38	138.7%
AK189432	2.346076	0.52	22.2%
AK189470	9.336638	1.34	14.3%
AK189601	0	0.00	
AK189633	6.700042	1.07	16.0%
AK189748	10.430934	2.19	21.0%
AK190196	3.89572	0.17	4.5%
AK190465	0.462671	0.12	26.1%
AK190497	1.676541	2.40	143.2%

AK190704	0.208051	0.14	67.9%
AK191080	2.737652	0.83	30.4%
AK191411	0.300622	0.24	80.8%
AK191492	0	0.00	
AK191498	1.19927	0.51	42.6%
AK191723	0	0.00	
AK191823	0.4554206	0.31	68.2%
AK192194	1.6163196	0.59	36.6%
AK192218	12.07132	0.95	7.9%
AK192544	48.21896	7.89	16.4%
AK192563	8.469178	0.47	5.6%
AK192647	2.186144	0.34	15.3%
AK192827	3.058228	0.56	18.2%
AK192985	0.13109628	0.03	26.0%
AK193394	0	0.00	
AK193439	0	0.00	
AK193714	14.3858	0.67	4.7%
AK194005	0.7564374	0.16	20.9%
AK194036	0	0.00	
AK194371	5.025018	0.52	10.4%
AK194920	1.423334	0.19	13.1%
AK195221	0.06771182	0.01	16.2%
AK195420	0	0.00	
AK195428	0	0.00	
AK195755	0.09698664	0.03	28.6%
AK195788	0	0.00	
AK195902	0.0525182	0.12	223.6%
AK195979	0.0552592	0.12	223.6%
AK196015	0.13062794	0.09	66.8%
AK196141	0.921862	0.33	36.1%
AK196214	0.292644	0.14	47.3%
AK196542	3.54514	1.34	37.8%
AK196832	0	0.00	
AK196959	0.1614836	0.05	28.4%
AK197085	6.098656	0.59	9.7%
AK197526	9.487962	1.65	17.4%
AK197603	0.109851	0.11	95.9%
AK197782	22.7088	0.54	2.4%
AK197973	0.4227218	0.22	51.9%
AK198271	3.724308	1.03	27.6%
AK198904	12.053398	1.90	15.8%
AK198984	0.7406712	0.22	29.1%
AK199184	0	0.00	
AK199258	24.19912	3.33	13.7%
AK199358	2.066158	0.30	14.5%
AK199495	0.2683538	0.08	28.0%
AK199694	0.7887996	0.24	30.8%

AK199756	0.1470466	0.21	145.6%
Ak2	8.554982	0.83	9.7%
AK200207	0.4473218	0.30	67.6%
AK200600	0.9269204	0.19	20.3%
AK200837	1.177838	0.46	38.8%
AK201601	11.75448	1.36	11.5%
AK202078	1.788454	0.59	33.1%
AK202488	0	0.00	
AK202494	1.865552	0.45	24.2%
AK202543	5.786278	1.72	29.7%
AK202580	0.9746928	0.37	37.6%
AK202663	0.0297132	0.06	195.1%
AK202794	22.14436	1.46	6.6%
AK203140	106.0336	3.78	3.6%
AK203176	23.7071	3.61	15.2%
AK203396	3.744372	0.97	26.0%
AK203628	0.3674538	0.22	59.2%
AK203706	1.893986	0.42	22.3%
AK203938	0.22875348	0.19	81.2%
AK204095	0	0.00	
AK204212	8.499652	2.53	29.7%
AK204365	1.1792268	0.53	44.6%
AK204442	3.879198	0.38	9.7%
AK204443	18.26638	1.83	10.0%
AK204799	0.5881916	0.16	27.7%
AK204824	5.64979	0.66	11.7%
AK204918	0	0.00	
AK205032	0.434666	0.28	63.8%
AK205147	0.4972064	0.09	18.6%
AK205190	0.1811142	0.18	100.2%
AK205332	0.0360502	0.08	223.6%
AK205379	0.05526546	0.08	149.0%
AK205396	0.0550448	0.08	136.9%
AK205555	26.91446	1.52	5.7%
AK205829	1.77149	0.50	28.4%
AK205936	4.774548	1.95	40.9%
AK206103	27.39176	3.76	13.7%
AK206637	20.5855	1.29	6.3%
AK206655	0.9578094	0.41	42.8%
AK206687	1.60834	0.32	19.6%
AK206780	0.1890526	0.12	61.0%
AK206957	8.425232	0.81	9.6%
AK207024	0.37565176	0.25	66.8%
AK207408	2.691778	0.91	33.6%
AK207593	0.5636636	0.16	28.3%
AK207688	0.01125414	0.03	223.6%
AK207899	0.0759064	0.10	138.0%

AK208354	0	0.00	
AK208404	26.89766	5.25	19.5%
AK208460	9.978502	1.14	11.4%
AK208554	16.99548	0.40	2.4%
AK208597	8.626362	1.19	13.8%
AK209396	1.6728074	1.13	67.8%
AK209590	3.758098	0.34	9.1%
AK209600	5.227634	0.76	14.5%
AK209647	38.48194	1.43	3.7%
AK209715	9.537928	2.24	23.4%
AK209885	5.544928	0.84	15.1%
AK210533	0.0242318	0.05	223.6%
AK210662	0.7105528	0.23	31.7%
AK211096	2.027652	0.26	12.9%
AK211120	0.0859318	0.09	104.7%
AK211336	1.0098244	0.54	53.1%
AK211473	2.794726	0.71	25.3%
AK211659	0	0.00	
AK212208	6.590664	0.92	13.9%
AK212603	3.36329	0.64	19.1%
AK212710	0.0873782	0.20	223.6%
AK212965	10.508454	1.06	10.1%
AK213166	0.2737484	0.27	100.4%
AK213200	0.4037918	0.21	51.6%
AK213404	0.2613294	0.03	11.5%
AK213543	1.884832	0.24	12.7%
AK213563	0.6933576	0.14	20.3%
AK213609	35.2583	3.20	9.1%
AK213636	0.0439746	0.10	223.6%
AK213652	0.4403874	0.27	61.2%
AK213749	0.1945446	0.03	16.8%
AK213775	1.0955128	0.56	50.9%
AK214072	0.2809846	0.28	97.9%
AK214167	0.5386236	0.06	11.3%
AK214332	0	0.00	
AK214371	0.6737044	0.37	54.5%
AK214451	3.657396	2.23	60.9%
AK214512	2.642428	0.66	25.0%
AK214583	4.611798	0.47	10.2%
AK214641	1.76691	0.13	7.6%
AK214683	0.2475174	0.14	57.2%
AK215081	0.05691388	0.03	58.3%
AK215237	0.5440924	0.29	53.3%
AK215297	0.37212	0.35	95.4%
AK215506	0	0.00	
AK215526	2.037182	0.55	26.8%
AK215842	2.311842	0.55	23.6%

AK216043	2.240264	0.49	21.9%
AK216440	0.5534516	0.18	32.9%
AK216443	0.1487042	0.20	137.0%
AK216459	0	0.00	
AK216578	0.0634534	0.14	223.6%
AK216819	0	0.00	
AK216824	0.07376536	0.12	160.3%
AK216857	0	0.00	
AK216920	5.491554	1.06	19.3%
AK217025	14.2216	1.27	9.0%
AK217047	1.0068436	0.60	59.5%
AK217058	0.5554112	0.09	15.3%
AK217269	0.02858112	0.04	147.4%
AK217604	4.857452	0.50	10.4%
AK217757	0	0.00	
AK217807	0.3704386	0.35	93.5%
AK217854	7.608952	1.07	14.0%
AK217856	0.24710098	0.11	44.4%
AK217863	1.66782	0.43	25.9%
AK217931	0.7307152	0.21	28.9%
AK217941	0.0997042	0.22	223.6%
AK217959	0.4541894	0.28	61.8%
AK217986	0.0582136	0.08	137.5%
AK218226	0.6525636	0.32	49.2%
AK218261	1.1730226	0.67	57.1%
AK218282	0	0.00	
AK218396	0	0.00	
AK218399	0	0.00	
AK218564	0	0.00	
AK219036	0.4370698	0.14	31.5%
AK219068	6.699902	0.48	7.2%
AK219219	0.4201726	0.40	94.4%
AK219239	9.039322	0.53	5.8%
AK219417	0.14123114	0.11	75.6%
AK219629	1.2713582	0.23	18.2%
AK219638	0.17502094	0.09	54.1%
AK219843	0.1183754	0.17	140.2%
AK220016	358.7628	9.39	2.6%
AK220028	0.028273	0.06	223.6%
AK220210	0.06812392	0.01	19.0%
AK220484	2.137586	0.04	2.0%
AK224942	0.212402672	0.47	223.6%
Ak3	20.8128	0.71	3.4%
Ak3l1	3.61829	0.83	22.9%
Ak5	7.234678	1.02	14.1%
Ak7	0.0542557	0.06	106.4%
Akap1	7.168334	0.24	3.4%

Akap10	7.457242	1.05	14.0%
Akap11	33.9317	1.48	4.4%
Akap12	29.38948	1.67	5.7%
Akap13	2.143338	0.40	18.7%
Akap14	0.01380922	0.02	141.3%
Akap2	17.54752	0.64	3.7%
Akap3	0.1367286	0.03	20.9%
Akap4	0.0027299	0.01	223.6%
Akap6	17.32826	1.00	5.8%
Akap7	7.449466	0.25	3.4%
Akap8	8.016926	1.56	19.4%
Akap8l	18.53994	2.21	11.9%
Akap9	5.627072	0.36	6.4%
AKAP95	7.216186	0.85	11.8%
Akd2	2.57843	0.42	16.5%
Akna	0.4089436	0.04	9.8%
Akp2	0.8210788	0.09	10.8%
Akp3	0.0187953	0.03	155.5%
Akp5	0	0.00	
Akr1a4	155.2596	11.06	7.1%
Akr1b3	1.973044	0.41	20.8%
Akr1b7	0.02517612	0.03	133.3%
Akr1b8	0.155426	0.11	71.3%
Akr1c12	0.3540152	0.08	21.6%
Akr1c13	0.805666	0.25	31.5%
Akr1c14	0.2825318	0.06	21.4%
Akr1c18	0	0.00	
Akr1c19	0.04320098	0.04	86.1%
Akr1c20	0	0.00	
Akr1c21	0.00669882	0.01	223.6%
Akr1c6	0.00496076	0.01	223.6%
Akr1d1	0	0.00	
Akr1e1	5.88376	0.54	9.3%
Akr7a5	7.316156	0.59	8.0%
Akt1	47.14404	2.33	4.9%
Akt1s1	11.68802	0.44	3.8%
Akt2	4.894596	0.26	5.4%
Akt3	26.5928	1.01	3.8%
Aktip	31.31308	2.07	6.6%
Alad	8.16739	1.04	12.8%
Alas1	11.69158	0.33	2.9%
Alas2	0.05726094	0.01	21.3%
Alb	0.01090006	0.01	99.8%
Alcam	31.60768	1.93	6.1%
Aldh16a1	2.730574	1.05	38.5%
Aldh18a1	12.37798	0.39	3.1%
Aldh1a1	1.688304	0.25	15.0%

Aldh1a2	0.2254504	0.04	18.0%
Aldh1a3	0.692447	0.06	8.6%
Aldh1a7	1.877378	0.20	10.9%
Aldh1b1	4.041104	0.46	11.3%
Aldh1l1	12.84906	1.49	11.6%
Aldh1l2	3.940512	0.59	15.0%
Aldh2	14.01956	0.50	3.6%
Aldh3a1	0.01110584	0.02	223.6%
Aldh3a2	12.59774	0.83	6.6%
Aldh3b1	0.160962	0.06	40.1%
Aldh3b2	0.01409358	0.01	68.6%
Aldh4a1	5.04953	0.32	6.3%
Aldh5a1	21.68462	1.16	5.4%
Aldh6	0.03619984	0.01	19.4%
Aldh6a1	10.519254	0.64	6.1%
Aldh7a1	7.301166	0.33	4.5%
Aldh8a1	0.07688476	0.02	30.4%
Aldh9a1	12.655	0.93	7.3%
Aldoa	417.5116	23.88	5.7%
Aldoart1	29.61456	1.90	6.4%
Aldoart2	28.3713	2.05	7.2%
Aldob	1.45943	0.27	18.6%
Aldoc	251.3778	16.39	6.5%
Alf4	11.53794	0.66	5.8%
Alg1	10.18455	0.45	4.4%
Alg10b	15.84342	1.04	6.5%
Alg11	13.30912	1.09	8.2%
Alg12	6.647436	0.23	3.4%
Alg13	4.750416	0.82	17.3%
Alg14	8.013654	0.44	5.5%
Alg2	93.69948	3.79	4.0%
Alg3	8.58252	0.76	8.8%
Alg5	10.066274	0.68	6.7%
Alg6	3.88132	0.26	6.8%
Alg8	3.14098	0.35	11.0%
Alg9	12.30412	0.39	3.2%
alk	2.8260382	1.80	63.8%
Alk	2.784578	0.61	21.9%
Alkbh1	7.690624	2.54	33.0%
Alkbh2	1.320416	0.18	13.6%
Alkbh3	4.074526	0.37	9.0%
Alkbh4	2.374378	0.15	6.5%
Alkbh5	8.344082	0.39	4.7%
Alkbh6	10.328836	0.78	7.5%
Alkbh7	9.357522	1.09	11.7%
Alkbh8	12.33324	0.83	6.7%
Allc	0	0.00	

Alms1	0.01251102	0.02	145.6%
Alox12	0.011550446	0.02	135.2%
Alox12b	0	0.00	
Alox12e	0.00208524	0.00	223.6%
Alox15	0.01950178	0.01	63.6%
Alox5	0.04991294	0.05	92.1%
Alox5ap	0.19135088	0.08	44.4%
Alox8	0.001527962	0.00	223.6%
Aloxe3	1.0749296	0.19	17.8%
alpha1B	7.528746	0.46	6.1%
Alpi	0	0.00	
Alpk2	0.01537028	0.00	27.8%
Alpk3	0.05680708	0.02	40.6%
Als2	11.85186	0.75	6.3%
Als2cl	0.7490402	0.04	5.8%
Als2cr11	0.0043156	0.01	223.6%
Als2cr12	0	0.00	
Als2cr13	19.56434	0.33	1.7%
Als2cr2	14.31722	1.24	8.6%
Als2cr4	7.913008	0.35	4.4%
Als2cr7	0.08540938	0.05	59.7%
Alx1	0.09822938	0.06	58.3%
Alx3	0.0155453	0.02	105.6%
Alx4	0.1511334	0.03	20.4%
AM295304	0.019166	0.01	39.2%
Amac1	0.1069502	0.04	41.9%
Amacr	3.562556	0.31	8.6%
Ambn	0	0.00	
Ambp	0.00916038	0.01	138.8%
Amd2	3.120666	0.18	5.8%
Amdhd1	0	0.00	
Amdhd2	7.49439	0.96	12.9%
Amelx	0.00665654	0.01	223.6%
Amfr	43.82658	1.67	3.8%
Amh	0.36747	0.14	38.7%
Amhr2	0.0853261	0.03	35.0%
Amica1	0.0191405	0.01	61.0%
Amigo1	13.29902	0.89	6.7%
Amigo2	4.11945	0.24	5.8%
Amigo3	1.0828198	0.09	8.7%
Ammecr1	1.923578	0.17	8.7%
Ammecr1l	9.300904	0.97	10.5%
Amn	0.5092014	0.08	16.4%
Amot	3.720904	0.11	2.8%
Amotl1	5.842764	0.54	9.2%
Amotl2	5.851344	0.34	5.8%
Ampd1	0.00325098	0.01	223.6%

Ampd2	19.92656	0.19	1.0%
Ampd3	2.873214	0.17	5.8%
Amph	70.34288	1.23	1.8%
Amt	6.45133	0.89	13.8%
Amy1	21.37636	4.65	21.8%
amy-1	0.1653098	0.05	28.3%
Amy2-1	0.0451621	0.03	60.6%
Anapc1	14.8266	0.81	5.4%
Anapc10	3.337196	0.42	12.5%
Anapc11	12.10718	0.61	5.0%
Anapc13	15.68044	1.16	7.4%
Anapc2	25.31548	0.93	3.7%
Anapc4	28.04634	1.42	5.1%
Anapc5	65.54418	1.47	2.2%
Anapc7	13.05314	0.77	5.9%
Ang	1.49889822	1.50	99.8%
Ang2	0	0.00	
Ang4	0	0.00	
Ang6	0	0.00	
Angel1	3.100528	0.28	9.0%
Angel2	9.484262	0.65	6.9%
Angpt1	2.207076	0.25	11.2%
Angpt2	0.03362544	0.02	55.1%
Angpt4	0.03304978	0.02	73.6%
Angptl1	0.15925502	0.05	31.8%
Angptl2	0.3434	0.09	26.4%
Angptl3	0.00340804	0.01	223.6%
Angptl4	0.07629118	0.05	64.0%
Angptl6	2.926336	0.15	5.0%
Angptl7	0.00323334	0.01	223.6%
Ank	50.05826	1.66	3.3%
Ank1	13.34578	1.06	7.9%
Ank2	103.73712	4.81	4.6%
Ank3	15.96948	2.13	13.3%
Ankar	0.01611278	0.02	98.8%
Ankfn1	0.1599666	0.02	13.7%
Ankfy1	7.524544	0.66	8.8%
Ankhd1	12.98402	0.71	5.5%
Ankhzn	0.14325438	0.11	77.7%
Ankib1	7.597938	0.29	3.8%
Ankk1	0.01805092	0.01	59.7%
Ankmy1	0.12234126	0.03	25.9%
Ankmy2	28.35184	1.67	5.9%
Ankra2	13.70198	0.59	4.3%
Ankrd1	0.01857186	0.02	100.7%
Ankrd10	15.05196	1.01	6.7%
Ankrd11	17.54032	1.37	7.8%

Ankrd12	7.635716	0.47	6.1%
Ankrd13a	10.9828	0.43	3.9%
Ankrd13b	23.7041	1.45	6.1%
Ankrd13c	29.68278	0.77	2.6%
Ankrd13d	42.1417	0.94	2.2%
Ankrd15	1.671298	0.15	8.8%
Ankrd16	3.512446	0.12	3.5%
Ankrd17	27.162	1.21	4.5%
Ankrd2	0.0076757	0.02	223.6%
Ankrd22	0.0037878	0.01	223.6%
Ankrd23	3.3472	0.40	11.8%
Ankrd24	8.205606	0.75	9.1%
Ankrd25	1.298948	0.16	12.3%
Ankrd26	2.722324	0.35	13.0%
Ankrd27	4.95361	0.26	5.3%
Ankrd28	6.800212	0.31	4.6%
Ankrd32	5.028036	0.22	4.4%
Ankrd33	0.09572374	0.06	57.5%
Ankrd34	5.130116	0.24	4.7%
Ankrd35	0.6310588	0.09	14.3%
Ankrd36	0	0.00	
Ankrd37	1.8572658	1.03	55.4%
Ankrd38	4.68356	0.50	10.7%
Ankrd39	2.676326	0.19	7.3%
Ankrd40	19.30544	0.72	3.8%
Ankrd41	0.12628148	0.06	47.2%
Ankrd42	4.920626	0.27	5.5%
Ankrd43	1.945768	0.20	10.2%
Ankrd44	3.464344	0.26	7.5%
Ankrd45	1.0798216	0.11	10.6%
Ankrd46	100.90406	3.47	3.4%
Ankrd47	0.7252206	0.13	17.6%
Ankrd48	0	0.00	
Ankrd49	3.789594	0.27	7.0%
Ankrd5	0.13180538	0.04	29.3%
Ankrd50	21.3237	1.25	5.9%
Ankrd52	19.65906	0.92	4.7%
Ankrd53	0.16340224	0.09	53.7%
Ankrd54	4.623802	0.51	11.0%
Ankrd55	0.4001458	0.08	20.4%
Ankrd56	0.7225734	0.15	20.6%
Ankrd58	0	0.00	
Ankrd6	1.184358	0.06	5.4%
Ankrd7	0	0.00	
Ankrd9	3.1306	0.37	11.7%
Anks1	3.852752	0.19	4.9%
Anks1b	21.42746	0.66	3.1%

Anks3	8.643034	0.99	11.4%
Anks4b	0	0.00	
Anks6	2.033938	0.18	8.8%
Ankzf1	6.795324	0.66	9.8%
Anln	0.7577428	0.18	23.4%
AnMKRN1	0.4581144	0.19	41.0%
Anp32a	39.69906	1.96	4.9%
Anp32b	18.1845	1.23	6.8%
Anp32e	21.14498	2.53	12.0%
Anpep	1.0157404	0.19	18.8%
Antxr1	2.486724	0.29	11.9%
Antxr2	1.525348	0.38	24.7%
Antxrl	0	0.00	
Anubl1	1.613194	0.19	12.0%
Anx3	0.09102494	0.02	24.4%
Anxa1	0.976365	0.08	8.2%
Anxa10	0.03781298	0.02	41.9%
Anxa11	0.335689	0.09	27.1%
Anxa13	0.01003006	0.01	138.8%
Anxa2	8.645658	2.10	24.3%
Anxa3	0.5774076	0.11	19.6%
Anxa4	2.43035	0.71	29.3%
Anxa5	20.56542	1.59	7.7%
Anxa6	39.89338	5.15	12.9%
Anxa7	11.9583	0.78	6.5%
Anxa8	0.06963212	0.02	29.7%
Anxa9	2.655452	0.66	24.7%
Aoah	0.05773048	0.03	44.3%
Aoc2	0.8778298	0.12	13.7%
Aoc3	0.02436714	0.01	42.8%
Aof1	2.50601	0.50	20.1%
Aof2	21.77774	0.71	3.3%
AOP2	1.0248496	0.58	56.4%
Aox1	0.12963018	0.07	57.5%
Aox3	0.04600006	0.02	42.8%
Aox3l1	0.005094034	0.00	58.5%
Aox4	0.3664106	0.04	11.3%
Ap1b1	63.08414	2.00	3.2%
Ap1g1	26.4976	1.62	6.1%
Ap1g2	3.225852	0.73	22.5%
Ap1gbp1	10.204262	0.65	6.4%
Ap1m1	40.47606	2.63	6.5%
Ap1m2	0.19859284	0.12	62.1%
Ap1s1	36.03122	3.06	8.5%
Ap1s2	71.95616	1.58	2.2%
Ap1s3	0.7918322	0.18	22.2%
Ap2a1	89.73118	2.89	3.2%

Ap2a2	54.65592	2.36	4.3%
Ap2b1	80.57466	4.01	5.0%
Ap2m1	16.8416	0.71	4.2%
Ap2s1	63.71916	2.78	4.4%
Ap3b1	11.25322	0.13	1.2%
Ap3b2	59.16524	2.44	4.1%
Ap3d1	48.46222	1.85	3.8%
Ap3m1	7.13748	0.71	9.9%
Ap3m2	31.85406	0.33	1.0%
Ap3s1	14.4006	0.57	3.9%
Ap3s2	22.57362	0.49	2.2%
Ap4b1	7.691262	0.39	5.1%
Ap4e1	3.680306	0.17	4.5%
Ap4m1	4.607132	0.17	3.7%
Ap4s1	18.71978	1.05	5.6%
Apa1	0.4626408	0.20	42.2%
Apaf1	4.764464	0.41	8.6%
Apba1	64.16326	4.77	7.4%
Apba2	31.23422	2.07	6.6%
Apba2bp	5.383736	0.76	14.1%
Apba3	2.9054	0.33	11.3%
Apbb1	82.67004	3.42	4.1%
Apbb1ip	0.11312048	0.03	27.6%
Apbb2	12.15488	0.64	5.3%
Apbb3	14.49216	0.90	6.2%
Apbh	0	0.00	
Apc	28.78142	2.45	8.5%
Apc2	26.02342	1.25	4.8%
Apcdd1	2.075104	0.15	7.2%
Apcs	0	0.00	
Apeh	14.5246	0.40	2.7%
Apex1	7.478692	1.71	22.9%
Apex2	2.141978	0.11	5.2%
APG16L	1.1053336	0.22	19.6%
apg-1b	0.0452663	0.01	23.2%
Aph1a	2.57082	0.17	6.5%
Aph1b	5.347372	0.30	5.7%
Aph1c	2.501948	0.34	13.4%
Api5	12.45598	0.42	3.4%
Apip	8.445846	0.79	9.4%
Apitd1	1.672682	0.20	12.0%
Apln	1.0637754	0.22	20.9%
Aplp1	196.151	10.61	5.4%
Aplp2	211.371	4.38	2.1%
APMAP	0.9101318	0.43	47.3%
apo	1.09912	0.43	39.0%
Apoa1	0.4279248	0.16	38.3%

Apoa1bp	29.2388	1.64	5.6%
Apoa2	0.01320876	0.03	223.6%
Apoa4	0	0.00	
Apoa5	0	0.00	
Apob	0.04136984	0.01	32.3%
Apob48r	0.0982491	0.03	27.1%
Apobec1	0.506543	0.05	10.4%
Apobec2	0.00729016	0.02	223.6%
Apobec3	0.5554248	0.16	28.8%
Apobec4	0	0.00	
Apoc1	0	0.00	
Apoc2	0.00717636	0.02	223.6%
Apoc3	0	0.00	
Apoc4	0	0.00	
Apod	4.29975	1.07	25.0%
Apoe	39.8434	2.19	5.5%
Apof	0	0.00	
Apoh	0.1937508	0.04	21.2%
Apol2	0.1848908	0.06	30.8%
Apol3	0.0228065	0.02	108.0%
Apol6	0.00638312	0.01	138.0%
Apom	0.03239902	0.04	110.9%
Apon	0.00756656	0.02	223.6%
Apool	2.091114	0.13	6.3%
App	426.2766	14.94	3.5%
Appbp1	18.93776	0.80	4.2%
Appbp2	32.73848	0.93	2.9%
Appl1	16.24572	1.18	7.3%
Appl2	6.31332	0.75	12.0%
Aprt	14.84988	1.16	7.8%
aprt	0.588052	0.19	32.0%
Aptx	5.690404	0.24	4.2%
Aqp1	0.4809338	0.27	56.3%
Aqp11	7.112948	0.56	7.8%
Aqp12	0.00536448	0.01	223.6%
Aqp2	0	0.00	
Aqp3	0.0031651	0.01	223.6%
Aqp4	7.122618	1.03	14.5%
Aqp5	0.6813324	0.21	31.3%
Aqp6	0	0.00	
Aqp7	0.00295454	0.01	223.6%
Aqp8	2.804956	0.69	24.6%
Aqp9	0.02362486	0.00	21.1%
Aqr	8.142426	0.43	5.3%
aquaporin_9	0.02122024	0.01	28.3%
Ar	0.6212294	0.10	16.7%
Araf	114.5742	2.32	2.0%

Arbp	32.3405	4.13	12.8%
Arc	0.9706446	0.25	25.7%
Arcn1	34.23554	2.04	6.0%
Ard1	11.428342	1.37	12.0%
Ard1b	0.007685578	0.01	161.6%
Areg	0.02228092	0.02	100.0%
Arf1	102.70718	4.21	4.1%
Arf2	32.56746	2.62	8.1%
Arf3	243.5626	11.74	4.8%
Arf4	69.76438	6.73	9.6%
Arf5	56.03014	4.66	8.3%
Arf6	14.1577	0.78	5.5%
Arfgap1	23.78898	0.82	3.4%
Arfgap3	8.503964	0.44	5.2%
Arfgef1	26.99612	1.59	5.9%
Arfgef2	10.747958	0.58	5.4%
Arfip1	4.193236	0.18	4.3%
Arfip2	24.39902	0.67	2.7%
Arfrp1	14.64034	1.04	7.1%
arg1	0.04230926	0.01	18.8%
Arg1	0.03309022	0.02	54.0%
Arg2	0.9162908	0.18	19.1%
Arhgap1	14.53688	0.86	5.9%
Arhgap10	1.648948	0.17	10.4%
Arhgap11a	1.446536	0.14	9.5%
Arhgap12	18.90696	0.82	4.3%
Arhgap15	0.0085141	0.02	223.6%
Arhgap17	1.331622	0.15	11.4%
Arhgap18	1.562522	0.16	10.0%
Arhgap19	0.792828	0.05	6.9%
Arhgap20	13.65192	0.67	4.9%
Arhgap21	14.53338	0.81	5.6%
Arhgap22	0.4015746	0.03	7.6%
Arhgap23	3.304568	0.41	12.3%
Arhgap24	9.619554	1.42	14.8%
Arhgap25	0.04906796	0.03	58.3%
Arhgap26	10.33663	0.39	3.8%
Arhgap27	0.8355604	0.20	24.4%
Arhgap28	0.400701	0.02	4.3%
Arhgap29	1.877762	0.23	12.1%
Arhgap30	0.010722838	0.00	44.1%
Arhgap4	0.1396625	0.17	124.8%
Arhgap5	22.99312	1.59	6.9%
Arhgap6	0.6671728	0.18	26.4%
Arhgap8	9.29298	0.77	8.3%
Arhgap9	0.01108492	0.01	93.6%
Arhgdia	140.2968	6.97	5.0%

Arhgdib	1.438836	0.16	10.8%
Arhgdig	42.54332	4.42	10.4%
Arhgef1	10.406622	1.09	10.5%
Arhgef10	1.328416	0.07	5.4%
Arhgef10l	9.69771	0.37	3.8%
Arhgef11	20.03218	0.62	3.1%
Arhgef12	15.5019	0.50	3.2%
Arhgef15	1.179412	0.12	10.3%
Arhgef16	0.265376	0.02	6.2%
Arhgef17	15.77548	1.66	10.5%
Arhgef18	7.674228	0.39	5.1%
Arhgef19	0.13730648	0.03	19.4%
Arhgef2	9.113656	0.31	3.5%
Arhgef3	2.93075	0.17	5.6%
Arhgef4	25.83266	1.66	6.4%
Arhgef5	0.2195312	0.04	17.5%
Arhgef6	2.596228	0.55	21.4%
Arhgef7	30.16002	1.86	6.2%
Arhgef9	33.05596	2.12	6.4%
Ari2	1.981538	1.03	52.1%
Arid1a	13.1906	0.25	1.9%
Arid1b	8.435728	0.38	4.5%
Arid2	8.697696	0.19	2.2%
Arid3a	1.284912	0.12	9.2%
Arid3b	0.3873694	0.05	14.0%
Arid3c	0.05302694	0.02	34.2%
Arid4a	5.915612	0.44	7.4%
Arid4b	11.31942	0.54	4.8%
Arid5a	1.0376924	0.11	10.7%
Arid5b	5.676432	0.48	8.4%
Arih1	18.31482	0.81	4.4%
Arih2	5.616278	0.79	14.1%
Arip2	16.04462	2.62	16.3%
Arkadia	1.973906	0.28	14.4%
Arl1	18.23526	0.77	4.2%
Arl10	11.67696	0.76	6.5%
Arl11	0.0368249	0.04	100.6%
Arl13a	0	0.00	
Arl13b	1.689766	0.27	16.0%
Arl15	7.732168	0.65	8.5%
Arl16	8.518244	0.67	7.9%
Arl2	57.15882	2.64	4.6%
Arl2bp	39.93102	1.85	4.6%
Arl3	48.98676	1.75	3.6%
Arl4a	11.15362	0.63	5.6%
Arl4c	36.43482	1.07	2.9%
Arl4d	0.9431258	0.20	21.3%

Arl5a	20.01738	0.59	3.0%
Arl5b	8.543316	0.79	9.2%
Arl5c	0.3090828	0.04	14.1%
Arl6	14.4241	0.41	2.8%
Arl6ip1	128.797	2.83	2.2%
Arl6ip2	19.04298	0.76	4.0%
Arl6ip4	23.65532	0.99	4.2%
Arl6ip5	43.9786	0.99	2.3%
Arl6ip6	4.184832	0.27	6.4%
Arl8a	105.0335	10.04	9.6%
Arl8b	102.6716	2.05	2.0%
Arl9	0.01793844	0.04	223.6%
Armc1	25.2944	1.27	5.0%
Armc10	4.883464	0.32	6.6%
Armc2	0.4227722	0.05	11.2%
Armc3	0.011786628	0.01	70.8%
Armc4	0.07544954	0.04	55.6%
Armc5	3.802796	0.22	5.9%
Armc6	17.00466	1.90	11.2%
Armc7	2.729918	0.34	12.6%
Armc8	19.47678	1.23	6.3%
Armc9	8.313068	1.16	13.9%
Armcx1	44.49056	1.01	2.3%
Armcx2	63.15018	2.47	3.9%
Armcx3	9.693554	0.23	2.4%
Armcx5	8.619164	0.69	8.0%
Armcx6	10.478974	0.86	8.2%
Armet	18.977	1.16	6.1%
Armetl1	0.5756406	0.07	12.7%
Arnt	2.965848	0.28	9.3%
Arnt2	24.8528	1.31	5.3%
Arntl	2.75741	0.17	6.1%
Arntl2	0.3268192	0.08	24.3%
Arpc1a	48.3995	3.53	7.3%
Arpc1b	1.687582	0.40	23.4%
Arpc2	60.8235	5.73	9.4%
Arpc3	39.74844	2.27	5.7%
Arpc4	50.47494	3.99	7.9%
Arpc5	21.35396	3.64	17.0%
Arpc5l	36.03056	0.98	2.7%
Arpm1	0.7485168	0.09	12.1%
Arpp19	32.20018	1.88	5.8%
Arpp21	30.82852	1.41	4.6%
Arr3	0	0.00	
Arrb1	33.27126	0.55	1.6%
Arrb2	17.6744	1.05	6.0%
Arrdc1	6.641632	0.42	6.3%

Arrdc2	3.033004	0.38	12.7%
Arrdc3	13.24684	0.57	4.3%
Arrdc4	4.77443	0.67	14.1%
Arrdc5	0	0.00	
Ars2	21.627	1.99	9.2%
Arsa	10.860798	0.83	7.6%
Arsb	6.579012	0.42	6.4%
Arsg	4.504726	2.11	46.8%
Arsi	0.1793418	0.08	44.0%
Arsj	0.00941314	0.01	69.1%
Arsk	1.187176	0.06	4.8%
Art1	0.00568306	0.01	223.6%
Art2a	0	0.00	
Art2b	0	0.00	
art3	0.05487048	0.09	156.3%
Art3	0.05213524	0.02	36.1%
Art4	0.01796446	0.01	46.3%
Art5	0	0.00	
Artn	0.2280286	0.05	23.2%
Arts1	1.891042	0.17	9.2%
Arv1	5.049192	0.41	8.2%
Arvcf	10.005984	0.72	7.2%
Arx	0.11669682	0.03	23.9%
As3mt	0.2207976	0.08	36.0%
Asah1	30.7752	1.61	5.2%
Asah2	6.301134	1.25	19.8%
Asah3	0.00289234	0.01	223.6%
Asah3l	0.10295624	0.04	42.9%
Asahl	2.35755	0.24	10.0%
Asb1	9.687738	1.03	10.7%
Asb10	0.00405546	0.01	223.6%
Asb11	0.00468444	0.01	223.6%
Asb12	0.0119817	0.02	139.1%
Asb13	24.90614	1.65	6.6%
Asb14	0.04554134	0.03	73.1%
Asb15	0.02047354	0.03	128.9%
Asb16	0.38632	0.07	18.9%
Asb17	0.00567282	0.01	223.6%
Asb18	0.2001744	0.07	36.1%
Asb2	0.027414988	0.03	98.6%
Asb3	5.43136	0.41	7.6%
Asb4	0.3884746	0.08	20.3%
Asb5	0.6882816	0.07	10.6%
Asb6	8.911228	0.76	8.5%
Asb7	3.128564	0.41	13.1%
Asb8	23.95632	1.50	6.3%
Asb9	0.00477266	0.01	223.6%

Ascc1	14.56412	1.02	7.0%
Ascc2	4.324684	0.19	4.3%
Ascc3	4.891338	0.43	8.8%
Ascc3l1	15.99318	0.77	4.8%
Ascl1	1.567278	0.20	12.8%
Ascl2	0.13490596	0.06	41.0%
Ascl3	0.0448244	0.01	22.7%
Asf1a	5.48503	0.34	6.3%
Asf1b	1.226982	0.16	12.8%
Asgr1	0.03731914	0.03	70.0%
Asgr2	0	0.00	
Ash1l	7.823546	0.54	6.9%
Ash2l	14.49248	0.93	6.4%
ASK2	0.0276251	0.02	62.5%
Asl	5.457048	0.14	2.5%
Asna1	39.82918	1.85	4.7%
Asns	72.15838	8.41	11.7%
Asnsd1	15.00458	1.74	11.6%
Asp	0.02441154	0.01	36.8%
asp3	0.3129554	0.28	87.9%
asp4	0.34418666	0.15	44.8%
Aspa	0.5646882	0.08	13.6%
Asph	39.99618	0.78	1.9%
Asphd1	18.9807	1.58	8.3%
Asphd2	16.26986	0.43	2.6%
Aspm	0.3405322	0.08	24.8%
Aspn	7.780642	1.62	20.8%
Asprv1	0.22692818	0.09	39.0%
Aspscr1	21.48688	1.10	5.1%
Asrgl1	15.3415	1.23	8.0%
Ass1	1.221238	0.11	8.8%
Aste1	1.475526	0.08	5.5%
Astl	0.00224498	0.01	223.6%
Astn1	43.7472	1.52	3.5%
Astn2	14.4323	0.57	3.9%
Asxl1	6.751888	0.30	4.4%
Asxl2	3.516718	0.21	5.9%
Asxl3	1.958876	0.27	13.6%
Asz1	0.00430862	0.01	223.6%
Atad1	69.6805	2.09	3.0%
Atad2	1.226206	0.15	11.8%
Atad2b	4.087044	0.17	4.1%
Atad3a	7.07936	0.35	5.0%
Atad4	0.09748348	0.02	18.5%
Atad5	1.1352	0.07	6.2%
Atcay	53.46124	1.64	3.1%
Ate1	22.08056	1.25	5.7%

Atf1	2.090956	0.18	8.7%
Atf2	35.2545	1.69	4.8%
Atf3	3.463118	0.62	18.0%
Atf4	90.61412	8.96	9.9%
Atf5	14.8562	2.42	16.3%
Atf6	6.824776	0.39	5.7%
Atf7	2.515774	0.19	7.7%
Atf7ip	7.0343	0.27	3.8%
Atf7ip2	0.00946814	0.01	138.9%
Atg10	4.719384	0.26	5.6%
Atg12	57.68224	2.81	4.9%
Atg16l1	13.73864	0.48	3.5%
Atg16l2	2.813708	0.27	9.5%
Atg2a	9.792428	0.56	5.7%
Atg2b	9.024872	0.59	6.6%
Atg3	15.66488	0.76	4.8%
Atg4a	0.4835018	0.07	15.2%
Atg4b	19.84442	0.48	2.4%
Atg4c	11.39294	0.45	3.9%
Atg4d	9.47974	0.55	5.8%
Atg5	9.20027	0.29	3.1%
Atg7	5.354632	0.30	5.6%
Atg9	2.0353406	0.84	41.1%
Atg9a	42.65876	0.95	2.2%
Atg9b	1.551178	0.18	11.3%
Athl1	0.8318564	0.11	13.1%
Atic	9.179184	0.80	8.8%
Atm	1.934276	0.24	12.3%
Atn1	41.66128	1.92	4.6%
Atoh1	0.11006766	0.05	46.0%
Atoh7	0.1950468	0.08	38.5%
Atoh8	0.1153112	0.05	41.8%
Atox1	67.71006	6.23	9.2%
Atp10a	0.8362734	0.08	9.3%
Atp10b	0.011440726	0.01	69.1%
Atp10d	0.934691	0.25	26.5%
Atp11a	10.35644	0.30	2.9%
Atp11b	42.97018	4.83	11.2%
Atp11c	7.091306	0.25	3.6%
Atp12a	0.02358914	0.01	41.5%
Atp13a1	13.33288	0.16	1.2%
Atp13a2	76.48256	3.23	4.2%
Atp13a4	0.704639	0.19	26.8%
Atp13a5	0.017804402	0.01	39.3%
Atp1a1	62.87256	3.25	5.2%
Atp1a2	28.27176	2.60	9.2%
Atp1a3	974.394	57.57	5.9%

Atp1b1	681.843	24.37	3.6%
Atp1b2	69.53864	5.82	8.4%
Atp1b3	27.13962	2.32	8.5%
Atp1b4	0.00186074	0.00	223.6%
Atp2a1	0.02216362	0.02	105.0%
Atp2a2	147.0924	1.81	1.2%
Atp2a3	0.08184304	0.01	10.1%
Atp2b1	47.73796	3.43	7.2%
Atp2b2	30.23348	1.72	5.7%
Atp2b3	22.62638	1.35	6.0%
Atp2b4	15.6017	1.27	8.1%
Atp2c1	20.7149	0.96	4.6%
Atp4a	0.335591	0.08	23.8%
Atp4b	0	0.00	
Atp5a1	416.0366	10.11	2.4%
Atp5b	569.8456	11.79	2.1%
Atp5c1	91.00316	3.74	4.1%
Atp5d	114.0988	3.45	3.0%
Atp5e	91.5677	5.97	6.5%
Atp5f1	88.05356	3.27	3.7%
Atp5g1	14.27316	1.05	7.4%
Atp5g2	0.1527994	0.04	28.2%
Atp5g3	323.2932	15.99	4.9%
Atp5h	10.082016	0.79	7.9%
Atp5j	75.73316	8.18	10.8%
Atp5j2	83.8025	4.80	5.7%
Atp5k	28.2595	1.70	6.0%
Atp5l	4.550444	1.00	22.0%
Atp5o	77.87972	6.99	9.0%
Atp5s	14.79748	0.64	4.3%
Atp6ap1	169.327	3.98	2.3%
Atp6ap2	151.6746	6.84	4.5%
Atp6c2	0.01009554	0.01	115.8%
Atp6v0a1	166.7228	5.71	3.4%
Atp6v0a2	8.73174	0.64	7.3%
Atp6v0a4	0.8067518	0.49	61.2%
Atp6v0b	141.3212	6.56	4.6%
Atp6v0c	220.0258	8.48	3.9%
Atp6v0d1	135.9836	9.23	6.8%
Atp6v0d2	0	0.00	
Atp6v0e	9.046116	0.65	7.2%
Atp6v0e2	216.902	13.19	6.1%
Atp6v1a	137.218	2.39	1.7%
Atp6v1b1	0.0240088	0.03	112.3%
Atp6v1b2	288.8432	5.05	1.7%
Atp6v1c1	128.708	2.09	1.6%
Atp6v1c2	0.031367718	0.02	69.3%

Atp6v1d	78.92774	1.35	1.7%
Atp6v1e1	172.3334	4.30	2.5%
Atp6v1e2	0.01195852	0.02	138.6%
Atp6v1f	52.61584	2.32	4.4%
Atp6v1g1	26.31142	2.21	8.4%
Atp6v1g2	152.1878	7.97	5.2%
Atp6v1g3	0	0.00	
Atp6v1h	71.99206	1.95	2.7%
Atp7a	1.2206184	0.15	12.3%
Atp7b	0.6450472	0.16	25.0%
Atp8a1	32.23668	0.95	3.0%
Atp8a2	14.42202	0.84	5.8%
Atp8b1	0.2067232	0.05	26.3%
Atp8b2	17.99464	0.80	4.5%
Atp8b3	0.10534668	0.03	25.2%
Atp8b4	0.002675786	0.00	137.7%
Atp9a	139.5974	7.60	5.4%
Atp9b	7.53515	0.66	8.8%
Atpaf1	2.971966	0.44	14.7%
Atpaf2	10.04709	0.18	1.7%
Atpase6	0.10293648	0.08	79.5%
Atpbd1b	2.846114	0.17	5.9%
Atpbd1c	8.178638	0.65	8.0%
Atpbd3	3.22808	0.45	14.0%
Atpbd4	8.565176	0.28	3.3%
Atpif1	118.334	11.20	9.5%
Atr	1.272152	0.28	22.1%
Atrn	21.4618	0.80	3.7%
Atrnl1	14.5466	0.38	2.6%
Atrx	17.53042	1.08	6.2%
Atxn1	3.172756	0.40	12.7%
Atxn10	177.1926	3.67	2.1%
Atxn2	30.57914	1.23	4.0%
Atxn2l	42.03764	1.55	3.7%
Atxn3	5.693942	0.52	9.2%
Atxn7	1.963544	0.29	14.6%
Atxn7l1	2.020766	0.06	2.9%
Atxn7l2	2.581756	0.22	8.4%
Atxn7l3	52.9535	2.11	4.0%
Atxn7l4	7.826558	0.43	5.5%
AU014645	18.857	1.04	5.5%
AU015228	0.04544096	0.03	55.7%
AU017455	0.09971522	0.03	30.2%
AU018091	0.00286926	0.01	223.6%
AU018778	0.0042826	0.01	223.6%
AU018829	0	0.00	
AU019823	4.703872	0.32	6.7%

AU020772	2.555396	0.15	5.9%
AU021034	0.0032178	0.01	223.6%
AU021092	0	0.00	
AU021838	14.55236	1.24	8.5%
AU022252	6.39508	0.38	5.9%
AU022751	0.01188734	0.01	103.0%
AU022870	12.59306	0.65	5.1%
AU023871	0	0.00	
AU040320	13.21638	0.29	2.2%
AU040829	30.9756	1.28	4.1%
AU042651	0.01122404	0.02	139.6%
AU042671	21.09724	1.34	6.3%
Auh	30.3143	1.73	5.7%
Aup1	18.98036	1.26	6.6%
Aurka	0.8372954	0.13	16.1%
Aurkaip1	36.52844	2.48	6.8%
Aurkb	0.6833688	0.19	28.3%
Aurkc	0.00943036	0.01	138.8%
Auts2	23.176	1.21	5.2%
av	0.31271954	0.17	53.8%
AV249152	1.37592	0.18	12.9%
AV312086	7.634396	0.23	3.0%
AV340375	0.08016294	0.03	37.8%
Av381126	0.00813066	0.02	223.6%
Aven	2.94689	0.53	18.1%
Avil	0.5950566	0.08	13.4%
Avp	0	0.00	
Avpi1	7.413454	1.45	19.5%
Avpr1a	1.1150792	0.26	23.2%
Avpr1b	0	0.00	
Avpr2	0.942098	0.34	36.2%
AW049604	64.89492	4.96	7.6%
AW061290	1.628162	0.18	11.3%
AW124722	1.359224	0.13	9.3%
AW125753	14.6965	1.21	8.2%
AW146020	0.9297274	0.28	29.9%
AW146154	1.567038	0.18	11.8%
AW146242	20.20354	1.32	6.5%
AW146299	0	0.00	
AW209491	6.1101	0.37	6.0%
AW456874	0.1583144	0.05	30.9%
AW491445	0.03635984	0.02	46.8%
AW544981	0.001652216	0.00	223.6%
AW548124	4.210066	0.39	9.2%
AW549877	39.6891	2.37	6.0%
AW551984	57.33956	4.68	8.2%
AW554918	6.517416	0.52	8.0%

AW555464	34.37562	1.07	3.1%
Awp1	0.9509364	0.24	25.4%
Axin1	4.850448	0.15	3.1%
Axin2	3.586916	0.50	13.9%
Axl	1.302704	0.19	14.3%
Axud1	0.3669824	0.07	20.3%
AY026312	0	0.00	
AY053573	0.03510436	0.03	75.2%
AY074887	0.241392	0.09	36.4%
AY078069	0.4494554	0.09	19.7%
AY096003	0.03089996	0.03	110.6%
AY139115	0	0.00	
AY227025	0.4894704	0.23	46.1%
AY227026	0.794394	0.23	28.4%
AY227735	0	0.00	
AY255546	9.43594	1.53	16.2%
AY255578	0.052958	0.07	139.1%
AY324401	0	0.00	
AY358078	0.4009148	0.12	30.0%
AY512908	0	0.00	
AY512909	0	0.00	
AY512910	0.01250258	0.01	102.6%
AY512912	0.03166338	0.02	63.7%
AY512915	0	0.00	
AY512917	0	0.00	
AY512931	0.0037354	0.01	223.6%
AY512935	0	0.00	
AY512941	0.00269618	0.01	223.6%
AY512942	0.2232764	0.09	39.8%
AY512943	0.00244722	0.01	223.6%
AY589788	0.2022964	0.07	33.8%
AY589789	0.004119864	0.01	181.4%
AY589791	0	0.00	
AY590888	0	0.00	
AY616753	0.0121481	0.02	141.4%
AY672066	0.839334	0.58	69.3%
AY761184	0.0254368	0.06	223.6%
AY761185	0	0.00	
AY940615	0	0.00	
AY940618	0	0.00	
Aym1	0	0.00	
Aytl1a	0.351097	0.09	25.9%
Aytl1b	0.00258464	0.01	223.6%
Aytl2	18.39784	2.29	12.5%
AZ2	1.479248	0.27	18.1%
Azgp1	0.004187	0.01	223.6%
Azi1	5.93219	0.13	2.2%

Azi2	19.0863	0.32	1.7%
Azin1	67.88366	6.61	9.7%
B020004J07Rik	0	0.00	
B020006M18Rik	0.0027981	0.01	223.6%
B020018G12Rik	8.697638	0.87	10.1%
B020031M17Rik	0	0.00	
B130050I23Rik	37.72496	0.87	2.3%
B144	0.05608196	0.05	97.2%
B230112C05Rik	16.17136	0.86	5.3%
B230118G17Rik	5.735818	0.54	9.4%
B230118H07Rik	6.00182	0.17	2.9%
B230120H23Rik	1.515234	0.16	10.5%
B230206H07Rik	0.0127005	0.02	179.3%
B230208H17Rik	19.04458	1.49	7.8%
B230209C24Rik	94.50008	5.26	5.6%
B230217C12Rik	19.41836	1.08	5.6%
B230218L05Rik	0.13185596	0.05	34.3%
B230219D22Rik	35.99062	2.19	6.1%
B230312A22Rik	8.728962	0.58	6.7%
B230312I18Rik	3.187836	0.23	7.3%
B230315F11Rik	2.528162	0.81	32.0%
B230315N10Rik	8.610742	0.47	5.4%
B230317C12Rik	30.43686	1.98	6.5%
B230339M05Rik	16.21024	0.83	5.1%
B230340J04Rik	1.0249278	0.12	11.2%
B230342M21Rik	12.98098	1.14	8.8%
B230354K17Rik	1.699524	0.34	20.1%
B230358A15Rik	0	0.00	
B230363K08Rik	0.12743118	0.06	47.1%
B230380D07Rik	11.60076	0.91	7.9%
B230396O12Rik	1.0100842	0.09	8.9%
B230399E16Rik	0.8980514	0.11	12.2%
B230399H06Rik	0	0.00	
B2m	31.9204	2.58	8.1%
B3bp	2.780194	0.22	7.9%
B3galnt1	30.30052	1.66	5.5%
B3galnt2	2.987056	0.20	6.6%
B3galt1	14.69316	0.66	4.5%
B3galt2	8.601032	0.46	5.4%
B3galt4	2.588914	0.18	6.8%
B3galt5	8.499696	1.23	14.4%
B3galt6	4.609944	0.30	6.4%
B3galtl	4.298848	0.34	7.8%
B3gat1	39.03182	1.90	4.9%
B3gat2	8.221596	0.60	7.3%
B3gat3	70.78196	2.84	4.0%
B3gnt1	67.4302	4.40	6.5%

B3gnt2	11.22266	1.36	12.1%
B3gnt3	0.10265958	0.01	14.0%
B3gnt4	0.2309208	0.08	33.3%
B3gnt5	1.0102558	0.25	24.3%
B3gnt6	0.2200998	0.03	13.7%
B3gnt7	1.159567	0.22	19.3%
B3gnt8	0.09700508	0.03	28.9%
B3gntl1	3.308096	1.91	57.6%
B430006D22Rik	2.060748	0.25	11.9%
B430119L13Rik	0.2297518	0.08	33.9%
B430203M17Rik	0.204303	0.11	52.5%
B430211C08Rik	0.5075222	0.06	11.7%
B430306N03Rik	0.001029658	0.00	223.6%
B4galnt1	37.41106	3.53	9.4%
B4galnt2	0.5938136	0.17	29.0%
B4galnt3	0.1580594	0.03	22.1%
B4galnt4	19.81662	1.20	6.0%
B4galt1	2.207546	0.32	14.4%
B4galt2	15.63578	1.63	10.4%
B4galt3	9.446806	0.69	7.3%
B4galt4	5.017726	0.16	3.3%
B4galt5	20.48636	2.49	12.2%
B4galt6	40.43598	1.06	2.6%
B4galt7	12.8345	0.67	5.2%
B630005N14Rik	10.343122	0.35	3.4%
B630019A10Rik	0.05716046	0.03	55.1%
B630019K06Rik	36.2145	2.26	6.2%
B830028B13Rik	1.552492	0.21	13.6%
B830045N13Rik	6.264708	0.51	8.2%
B930006L02Rik	54.52166	1.93	3.5%
B930007L02Rik	3.31079	0.37	11.2%
B930007M17Rik	0.0187266	0.02	91.4%
B930011P16Rik	0.05281582	0.03	65.3%
B930037P14Rik	46.5865	0.48	1.0%
B930041F14Rik	14.10502	0.45	3.2%
B930095G15Rik	3.58979	0.37	10.4%
B9d1	6.22017	1.11	17.8%
B9d2	8.580848	0.78	9.1%
Baalc	12.02044	1.02	8.5%
Baat	0.03864764	0.01	34.2%
Bace	4.643382	1.46	31.4%
Bace1	19.27728	1.66	8.6%
Bace2	1.477048	0.28	19.0%
Bach1	5.463014	0.33	6.1%
Bach2	1.513246	0.13	8.8%
Bad	23.48794	2.81	12.0%
Bag1	42.9768	2.21	5.2%

Bag2	5.801986	0.42	7.3%
Bag3	1.365878	0.21	15.6%
Bag4	12.0288	0.67	5.6%
Bag5	11.94798	0.46	3.9%
Bahcc1	2.730382	0.20	7.4%
Bahd1	5.140342	0.27	5.2%
Bai1	41.28892	0.92	2.2%
Bai2	34.20248	0.77	2.3%
Bai3	19.49944	0.97	5.0%
Baiap2	6.09118	0.44	7.3%
Baiap2l1	0.241909	0.05	20.9%
Baiap2l2	0.6322026	0.06	9.9%
Baiap3	0.9676702	0.21	21.6%
Bak1	10.50284	1.40	13.3%
Bambi	0.7683048	0.07	9.3%
Banf1	42.37466	4.92	11.6%
Bank1	0.00476846	0.01	142.7%
Banp	3.671096	0.25	6.8%
Bap1	31.34902	1.01	3.2%
Bara	0.6031336	0.06	10.7%
Bard1	0.7957842	0.12	14.7%
Barhl1	5.285682	1.48	28.0%
Barhl2	1.981704	0.40	20.3%
Barx1	1.371278	0.18	13.4%
Barx2	0.05249284	0.03	63.8%
Basp1	296.3142	23.45	7.9%
Bat1a	70.28548	3.40	4.8%
Bat2	42.44946	1.29	3.0%
Bat2d	29.22	1.40	4.8%
Bat3	64.16718	2.89	4.5%
Bat4	2.961668	0.35	11.8%
Bat5	19.51866	0.67	3.5%
Batf	0.00437136	0.01	223.6%
Batf2	0.00807978	0.01	140.8%
Bax	26.1362	2.27	8.7%
Baz1a	0.214527	0.04	20.8%
Baz1b	12.73832	0.57	4.5%
Baz2a	9.309664	1.22	13.1%
Baz2b	6.476676	0.62	9.6%
BB014433	0	0.00	
BB146404	0.01380132	0.03	223.6%
BB220380	0.00665461	0.01	98.8%
Bbc3	6.88596	0.31	4.6%
Bbox1	0.05485542	0.05	83.2%
Bbs1	8.92165	0.20	2.3%
Bbs12	0.9594258	0.23	23.7%
Bbs2	7.496396	0.82	10.9%

Bbs4	15.04206	0.49	3.3%
Bbs5	5.963606	0.62	10.4%
Bbs7	7.876944	0.45	5.7%
Bbs9	4.479118	0.14	3.1%
Bbx	2.823064	0.24	8.5%
BC002059	1.472694	0.12	8.4%
BC002163	82.62706	4.84	5.9%
BC002199	25.1911	1.66	6.6%
BC002216	13.36638	0.36	2.7%
BC002230	2.124108	0.12	5.7%
BC002288	0.06647494	0.03	45.2%
BC002310	4.138362	0.30	7.2%
BC002320	0.668716	0.59	88.8%
BC003266	17.76688	1.65	9.3%
BC003267	5.859348	0.49	8.3%
BC003324	3.139044	0.40	12.7%
BC003331	12.2301	0.72	5.9%
BC003348	29.19028	1.09	3.7%
BC003885	10.87955	0.93	8.6%
BC003940	43.00226	3.64	8.5%
BC003993	2.349676	0.24	10.3%
BC004004	15.41924	1.27	8.2%
BC004022	8.202394	0.56	6.9%
BC004044	11.28238	0.67	6.0%
BC004728	0.298251	0.07	25.0%
BC005537	46.31426	3.38	7.3%
BC005561	1.3062352	0.35	26.8%
BC005624	11.03006	0.65	5.9%
BC005764	56.05518	3.04	5.4%
BC005803	11.69976	0.73	6.3%
BC006662	9.308492	0.83	8.9%
BC006779	0.4089074	0.09	22.9%
BC006885	11.18668	0.29	2.6%
BC006965	0.0708591	0.04	60.2%
BC008155	22.21362	1.86	8.4%
BC008163	3.772288	0.30	8.1%
BC009120	2.127676	0.29	13.5%
BC010304	16.39594	0.89	5.5%
BC010787	12.32118	1.93	15.7%
BC011248	7.185536	0.68	9.5%
BC011383	0.7839638	0.23	28.7%
BC011426	2.251752	0.17	7.5%
BC011467	10.92786	0.94	8.6%
BC011487	0.5591076	0.02	4.0%
BC012278	2.4031	0.39	16.4%
BC012717	0.5631096	0.19	33.5%
BC013481	0.2103174	0.05	24.5%

BC013491	0.06580014	0.05	72.1%
BC013529	6.106832	0.38	6.2%
BC013672	0.021937428	0.02	94.8%
BC013712	0	0.00	
BC014805	0	0.00	
BC015286	0.00265734	0.01	223.6%
BC016201	0	0.00	
BC016423	4.540426	0.24	5.3%
BC016495	1.66014	0.25	14.9%
BC016548	0.00602282	0.01	223.6%
BC016579	0.0034949	0.01	223.6%
BC017158	7.851328	0.24	3.0%
BC017612	0.736501	0.23	31.9%
BC017643	9.003342	0.46	5.2%
BC017647	8.918438	0.72	8.0%
BC018101	0.3032854	0.04	12.0%
BC018200	15.9004	1.58	10.0%
BC018242	62.26446	4.93	7.9%
BC018310	1.791052	0.18	10.3%
BC018371	0.949485	0.10	10.1%
BC018465	0.00323038	0.01	223.6%
BC018507	17.30964	0.66	3.8%
BC019639	7.038798	0.61	8.7%
BC019681	0.09451888	0.04	42.4%
BC019699	0.1708626	0.07	41.3%
BC019819	0.0035333	0.01	223.6%
BC019943	5.813728	0.16	2.7%
BC020002	7.253418	0.43	6.0%
BC020077	0.3331008	0.05	13.7%
BC020182	1.6566032	0.79	47.5%
BC020326	3.285382	0.32	9.6%
BC020394	0.0314333	0.00	10.9%
BC020449	1.83254	0.37	20.4%
BC020535	0.00925798	0.01	110.5%
BC021381	8.594214	0.68	8.0%
BC021395	10.877426	0.83	7.6%
BC021523	6.265878	0.13	2.0%
BC021608	0.10209068	0.05	53.8%
BC021611	7.990414	0.45	5.6%
BC021614	0	0.00	
BC021767	0.7674586	0.08	10.2%
BC021774	0.003603996	0.00	137.3%
BC021785	0.05092384	0.04	84.6%
BC021891	0.8483008	0.14	15.9%
BC021919	0	0.00	
BC021953	0.431735	0.19	43.0%
BC022224	2.111722	0.22	10.2%

BC022590	10.77677	0.97	9.0%
BC022623	4.216274	0.22	5.2%
BC022651	0.11130392	0.06	50.5%
BC022687	3.36963	0.39	11.6%
BC023055	36.23802	2.79	7.7%
BC023070	0.00855942	0.02	223.6%
BC023105	0.00413386	0.01	223.6%
BC023179	1.137642	0.11	9.7%
BC023202	4.264616	0.11	2.6%
BC023483	7.688634	1.44	18.7%
BC023719	0.08318064	0.02	27.2%
BC023744	0.5462494	0.13	24.7%
BC023814	4.902426	0.28	5.6%
BC023818	12.56928	1.85	14.8%
BC023829	12.80928	0.51	4.0%
BC023882	2.107152	0.57	27.0%
BC023947	3.204928	0.69	21.5%
BC024139	0.003402238	0.00	137.8%
BC024386	0	0.00	
BC024479	4.880528	0.37	7.6%
BC024561	0.1379676	0.09	62.5%
BC024571	3.124058	0.69	22.0%
BC024582	0.04422336	0.02	49.6%
BC024672	0.10689654	0.06	55.2%
BC024760	0.2065624	0.06	28.1%
BC024814	9.684442	1.28	13.2%
BC024868	23.9472	0.40	1.7%
BC024997	0.00261094	0.01	223.6%
BC025054	0.2483114	0.03	13.0%
BC025067	2.182532	0.70	32.3%
BC025076	2.390752	0.22	9.1%
BC025190	4.640736	0.44	9.4%
BC025446	0.06432896	0.04	63.0%
BC025462	0.19304588	0.17	89.2%
BC025546	1.0933812	0.11	10.4%
BC025575	18.25622	1.71	9.4%
BC025816	3.155776	0.20	6.3%
BC025885	29.6798	0.65	2.2%
BC025945	0	0.00	
BC026374	0	0.00	
BC026434	8.616608	0.50	5.8%
BC026439	0	0.00	
BC026478	0.2920652	0.06	21.0%
BC026585	0.2511438	0.06	23.2%
BC026590	4.213206	0.30	7.1%
BC026600	0.16324282	0.07	44.6%
BC026601	2.638516	0.06	2.1%

BC026682	0.0222501	0.00	8.9%
BC026782	0	0.00	
BC026913	7.85917	1.51	19.2%
BC027057	0	0.00	
BC027072	0.011791674	0.01	90.6%
BC027231	2.51152	0.17	6.6%
BC027344	4.678514	0.19	4.0%
BC027359	1.608848	0.36	22.4%
BC027389	0.33953734	0.15	42.8%
BC027541	0.280081	0.14	49.8%
BC027544	0	0.00	
BC027576	0	0.00	
BC027664	0	0.00	
BC027670	0.0243438	0.05	223.6%
BC027685	0.05049118	0.05	92.0%
BC027696	0.10552048	0.02	15.5%
BC027703	1.4891074	0.47	31.6%
BC027814	0.7653358	0.09	11.1%
BC027835	29.90722	2.32	7.8%
BC028252	0.20829824	0.07	32.9%
BC028528	0.2325866	0.04	17.7%
BC028532	0.00949368	0.02	223.6%
BC028660	0	0.00	
BC028663	8.831238	0.73	8.3%
BC028777	0.53633794	0.51	95.8%
BC028801	15.8561	1.96	12.3%
BC028804	0.0370374	0.02	53.7%
BC028808	0.07501234	0.04	55.3%
BC028950	0	0.00	
BC029165	9.519584	0.45	4.7%
BC029169	1.684724	0.21	12.2%
BC029214	9.765794	1.06	10.9%
BC029331	5.602504	0.25	4.5%
BC029684	0.0111539	0.02	143.6%
BC029710	0	0.00	
BC029726	1.789434	0.16	8.7%
BC030045	18.22504	1.01	5.6%
BC030046	11.08452	0.31	2.8%
BC030050	0.3952376	0.09	23.5%
BC030183	0.00741334	0.02	223.6%
BC030307	0.4485266	0.03	6.5%
BC030336	26.34772	1.29	4.9%
BC030392	0	0.00	
BC030396	0	0.00	
BC030398	0.04672904	0.04	81.9%
BC030440	0.2417146	0.07	30.9%
BC030469	0	0.00	

BC030470	0.6461848	0.20	31.2%
BC030476	0.14681598	0.07	44.9%
BC030477	0.3000626	0.09	29.8%
BC030499	0.08875728	0.09	101.1%
BC030500	2.509198	0.46	18.3%
BC030867	0.160166	0.02	13.1%
BC030884	11.2137	0.50	4.5%
BC031181	63.05832	4.75	7.5%
BC031353	8.768002	0.47	5.4%
BC031434	1.0512328	0.31	29.2%
BC031441	1.658534	0.24	14.2%
BC031503	3.042064	0.20	6.6%
BC031748	3.668992	0.27	7.4%
BC031781	6.995414	0.56	8.0%
BC031853	8.633592	0.40	4.6%
BC031901	0.00366814	0.01	223.6%
BC032181	0.00215888	0.00	223.6%
BC032204	1.276185	0.48	37.4%
BC032265	7.85171	0.40	5.1%
BC032967	1.333952	0.06	4.3%
BC033430	1.991266	0.29	14.5%
BC033443	0.05520428	0.02	32.5%
BC033467	0.08992622	0.03	29.1%
BC033915	24.3908	2.08	8.5%
BC033916	0.2398508	0.06	26.8%
BC033932	0.03677294	0.03	74.5%
BC034076	0.08338178	0.03	30.2%
BC034090	0.4368682	0.07	15.1%
BC034093	0.6199322	0.14	22.0%
BC034252	5.582418	1.01	18.2%
BC034735	9.406642	1.08	11.4%
BC034902	0.026660025	0.03	99.8%
BC035044	0.0050535	0.01	137.6%
BC035255	7.548674	0.36	4.8%
BC035295	10.706036	1.01	9.5%
BC035532	0.577768	0.14	23.5%
BC035537	3.546786	0.29	8.2%
BC035947	0.07372214	0.03	37.3%
BC035954	0.4690482	0.12	26.5%
BC036313	17.3296	0.77	4.4%
BC036641	0.001634418	0.00	223.6%
BC037032	1.1369848	0.10	9.2%
BC037034	27.31372	0.97	3.6%
BC037112	13.43244	0.62	4.6%
BC037156	0	0.00	
BC037178	0.4021368	0.25	63.3%
BC037369	7.147756	0.48	6.7%

BC037393	3.969058	0.39	9.7%
BC037520	12.68864	1.14	8.9%
BC037703	0.04878432	0.02	42.5%
BC038073	0.00126686	0.00	223.6%
BC038155	0.0057694	0.01	223.6%
BC038156	3.737108	0.29	7.8%
BC038167	0	0.00	
BC038171	0.5453914	0.10	19.1%
BC038278	0.00819744	0.01	162.7%
BC038286	87.01396	2.43	2.8%
BC038328	0.08327424	0.06	69.5%
BC038330	0.07580272	0.04	54.3%
BC038349	5.430844	0.18	3.3%
BC038474	0.396436	0.14	36.2%
BC038479	2.119554	0.26	12.4%
BC038520	0.03277326	0.02	68.6%
BC038648	0	0.00	
BC038822	2.940146	0.09	3.2%
BC038925	1.658462	0.08	4.7%
BC038927	0.0062604	0.01	223.6%
BC038929	0.13803314	0.04	27.0%
BC039093	9.19481	0.65	7.1%
BC039210	0.3351382	0.08	24.9%
BC039632	0.5257072	0.11	20.7%
BC039766	0	0.00	
BC039771	0.0300855	0.01	22.6%
BC039791	0	0.00	
BC039928	3.602292	0.29	8.0%
BC039940	0.0348791	0.05	142.4%
BC039975	5.194412	0.33	6.4%
BC039997	0.14262098	0.09	64.8%
BC040216	0.03243079	0.02	61.8%
BC040255	0.0978875	0.04	36.8%
BC040266	1.1832806	0.19	16.4%
BC040690	1.536856	0.14	9.2%
BC040754	0	0.00	
BC040758	0.008248018	0.01	142.7%
BC040803	5.65567	1.26	22.2%
BC040804	5.622146	0.27	4.7%
BC042407	0	0.00	
BC042696	1.995008	0.30	15.1%
BC042720	12.129386	1.67	13.8%
BC042727	0.02109124	0.01	57.8%
BC042782	0.03177626	0.03	105.2%
BC042783	0.14756976	0.10	65.7%
BC042789	0	0.00	
BC043098	106.4016	1.16	1.1%

BC043118	0.3038404	0.05	15.3%
BC043301	2.663688	0.22	8.4%
BC043476	2.80979	0.49	17.4%
BC043932	1.1098376	0.17	15.3%
BC043934	0.01266688	0.01	108.1%
BC043943	0.3873238	0.05	14.0%
BC044745	4.618548	0.43	9.2%
BC044868	0	0.00	
BC044870	0.3301778	0.08	24.0%
BC044880	0.01722416	0.01	83.9%
BC046251	0.09375358	0.05	53.9%
BC046331	5.618634	0.31	5.6%
BC046404	0.7823814	0.09	12.0%
BC046436	0	0.00	
BC046487	0.03925192	0.04	106.0%
BC046508	0	0.00	
BC047062	0	0.00	
BC047208	9.000084	0.82	9.1%
BC047216	0	0.00	
BC048080	0.7170696	0.10	13.3%
BC048354	14.3883	0.58	4.0%
BC048355	1.381622	0.10	7.5%
BC048390	0	0.00	
BC048393	0.2135472	0.02	10.5%
BC048403	3.728582	0.31	8.4%
BC048502	0	0.00	
BC048507	2.027172	0.56	27.4%
BC048519	0	0.00	
BC048546	0.14183732	0.11	77.3%
BC048555	0	0.00	
BC048556	11.23154	1.26	11.2%
BC048559	0	0.00	
BC048562	0	0.00	
BC048576	0	0.00	
BC048579	0	0.00	
BC048584	0.01161974	0.03	223.6%
BC048585	0	0.00	
BC048594	0.3298934	0.14	42.8%
BC048599	0	0.00	
BC048600	0	0.00	
BC048601	0	0.00	
BC048602	0	0.00	
BC048609	0.000001852	0.00	223.6%
BC048612	1.4061268	0.36	25.5%
BC048615	0	0.00	
BC048628	0	0.00	
BC048643	0	0.00	

BC048644	0.02997412	0.02	61.1%
BC048649	0	0.00	
BC048651	0.00427326	0.01	223.6%
BC048654	0	0.00	
BC048655	0	0.00	
BC048656	0	0.00	
BC048662	0	0.00	
BC048663	0.14872316	0.07	46.0%
BC048666	0.03321898	0.05	154.5%
BC048671	0	0.00	
BC048672	0	0.00	
BC048678	0	0.00	
BC048679	0	0.00	
BC048715	0.04045928	0.03	71.5%
BC048720	0.12709708	0.08	61.5%
BC048731	2.536912	0.32	12.7%
BC048749	0.7998716	0.13	16.5%
BC048761	0.06821528	0.02	24.9%
BC048764	0	0.00	
BC048765	0	0.00	
BC048766	0.13239702	0.06	43.7%
BC048859	0.033821258	0.02	61.7%
BC048943	0.00238186	0.01	223.6%
BC048947	1.111792013	0.70	63.2%
BC048951	7.915252	0.33	4.2%
BC049144	1.317896	0.15	11.2%
BC049259	13.67926	2.08	15.2%
BC049265	1.1621172	1.46	125.9%
BC049349	3.569114	0.34	9.7%
BC049352	0.0763961	0.03	38.1%
BC049560	0.00530558	0.01	139.1%
BC049635	1.686888	0.40	23.7%
BC049642	0	0.00	
BC049653	0.1205545	0.06	47.8%
BC049655	0.00762834	0.02	223.6%
BC049671	1.922098	0.58	30.2%
BC049685	0.03017882	0.03	83.4%
BC049692	0	0.00	
BC049702	0	0.00	
BC049706	0	0.00	
BC049715	1.679334	0.21	12.5%
BC049716	0.01505534	0.02	140.6%
BC049717	0	0.00	
BC049730	0.0055072	0.01	223.6%
BC049735	0.1442392	0.21	146.1%
BC049749	0	0.00	
BC049760	0.00720656	0.02	223.6%

BC049762	0.04623248	0.03	62.5%
BC049805	4.538122	0.38	8.5%
BC049806	14.40664	2.30	16.0%
BC049807	4.84221	0.31	6.3%
BC049966	0.02673558	0.01	30.1%
BC050071	0.07944194	0.03	37.7%
BC050078	3.413876	0.24	7.1%
BC050092	0.6648486	0.08	11.3%
BC050099	0.0098051	0.01	91.8%
BC050122	1.0019276	0.11	11.0%
BC050135	0.01353454	0.03	223.6%
BC050140	0.03967986	0.03	70.7%
BC050196	0.09207466	0.03	36.4%
BC050210	0.00354722	0.01	223.6%
BC050213	0.07196376	0.01	19.3%
BC050254	3.39007	0.23	6.9%
BC050777	0.04772386	0.03	54.3%
BC050789	0.009971	0.01	137.6%
BC050868	21.17036	0.73	3.5%
BC050972	0	0.00	
BC050974	0.10039278	0.04	37.4%
BC050980	0.04452992	0.03	63.6%
BC050982	0	0.00	
BC050987	0.1112685	0.03	23.9%
BC051019	0.36760708	0.50	135.1%
BC051062	0.5310694	0.15	28.2%
BC051070	0	0.00	
BC051142	0.8793726	0.13	14.5%
BC051212	0.03304162	0.03	87.3%
BC051227	11.130518	1.07	9.6%
BC051232	1.3285052	0.26	19.2%
BC051402	0	0.00	
BC051407	0.4304332	0.15	34.8%
BC051515	9.064086	0.72	8.0%
BC051516	0.07667148	0.03	37.3%
BC051525	0	0.00	
BC051537	0.00527668	0.01	223.6%
BC051589	6.012858	0.87	14.4%
BC051628	0.00302198	0.01	223.6%
BC051665	0	0.00	
BC052040	6.046092	0.43	7.1%
BC052055	12.87576	1.40	10.9%
BC052328	3.457006	0.38	11.1%
BC052364	0.03783692	0.03	79.6%
BC052484	0.0171647	0.02	99.6%
BC052524	0	0.00	
BC052551	0.7091048	0.21	29.0%

BC052688	0.145729	0.03	20.7%
BC053393	0	0.00	
BC053440	4.159358	0.33	7.8%
BC053725	0.611409	0.12	20.3%
BC053749	1.559778	0.13	8.5%
BC053974	0.4547708	0.12	26.1%
BC053977	0	0.00	
BC053978	0	0.00	
BC053994	1.327638	0.20	15.2%
BC054059	0	0.00	
BC054075	3.0172	0.65	21.6%
BC054394	3.924612	0.26	6.5%
BC054406	0.11777318	0.04	35.5%
BC054438	0.5864088	0.19	31.8%
BC055004	0	0.00	
BC055065	0	0.00	
BC055107	0.11235888	0.02	20.1%
BC055111	0.08107786	0.05	60.6%
BC055324	1.8042368	1.04	57.4%
BC055402	0.0050503	0.01	138.6%
BC055403	10.98958	0.76	6.9%
BC055463	0.4280258	0.39	90.1%
BC055806	2.572288	0.34	13.1%
BC055823	0.05821094	0.04	76.0%
BC055918	2.09512	0.58	27.7%
BC055944	0.5029299	0.51	100.8%
BC056163	0.0562594	0.13	223.6%
BC056174	1.212604	0.22	18.2%
BC056189	0.3176802	0.12	36.8%
BC056356	0.06752006	0.02	22.5%
BC056393	62.25208	4.51	7.2%
BC056474	5.200462	0.81	15.6%
BC056923	0.02680646	0.01	40.5%
BC057022	1.888036	0.30	15.6%
BC057079	6.53648	0.44	6.8%
BC057135	0.009598398	0.01	72.7%
BC057170	0.2193622	0.06	25.8%
BC057371	23.30814	0.98	4.2%
BC057381	7.622316	1.00	13.1%
BC057552	10.143906	1.32	13.0%
BC057593	0.6140336	0.25	41.2%
BC057611	0.13282192	0.06	41.9%
BC057627	7.110072	0.66	9.3%
BC057893	2.784886	0.23	8.2%
BC058096	0.6021838	0.13	21.5%
BC058417	0.205819	0.03	12.9%
BC058420	1.174154	0.13	11.0%

BC058428	2.459522	0.39	15.7%
BC058545	2.131022	0.26	12.3%
BC058760	12.6956	0.77	6.0%
BC058988	0.11393822	0.09	78.7%
BC059025	6.801172	1.63	23.9%
BC059842	3.99384	0.47	11.7%
BC059853	3.921252	0.21	5.5%
BC059867	3.593622	0.30	8.4%
BC059891	3.47501	0.39	11.2%
BC059892	3.955174	0.93	23.5%
BC060111	7.18143	1.61	22.5%
BC060136	5.307142	0.73	13.7%
BC060146	0.145182546	0.13	91.0%
BC060267	0.1620038	0.04	22.6%
BC060302	0	0.00	
BC060616	3.765616	0.38	10.0%
BC060631	22.22048	0.71	3.2%
BC060632	35.24702	1.09	3.1%
BC060988	2.311334	0.38	16.6%
BC061039	0.08447466	0.04	44.5%
BC061047	0	0.00	
BC061156	0	0.00	
BC061194	0.4118276	0.20	47.6%
BC061212	0	0.00	
BC061237	0	0.00	
BC061253	11.6826	0.82	7.0%
BC061511	0.6119618	0.20	32.4%
BC062109	11.34516	0.97	8.5%
BC062115	1.83156	0.09	4.8%
BC062127	0.1265034	0.01	10.9%
BC062185	0.3829504	0.09	23.5%
BC062254	0.7748502	0.28	36.0%
BC062650	2.474208	0.16	6.5%
BC062820	0.19011342	0.29	154.5%
BC062821	0	0.00	
BC062895	3.368948	0.57	16.8%
BC062913	0.09639944	0.06	63.3%
BC063263	1.99531	0.18	9.1%
BC063749	0.8798172	0.13	15.0%
BC064033	0.0476319	0.03	54.3%
BC064072	0.00661499	0.01	158.7%
BC064078	2.767576	0.35	12.5%
BC064103	0.02752312	0.02	69.1%
BC064105	0.02994602	0.04	142.9%
BC064108	0.374172	0.16	42.0%
BC064116	0	0.00	
BC064118	0.5288176	0.16	30.9%

BC064125	0	0.00	
BC064451	0	0.00	
BC064456	4.412458	0.59	13.4%
BC064714	0.8315078	0.14	17.3%
BC064812	0.3341898	0.02	6.1%
BC065048	0.10926608	0.02	19.5%
BC065085	1.196366	0.14	11.5%
BC065087	0.0070646	0.01	138.2%
BC065127	0.6558634	0.10	15.2%
BC065149	0.7612628	0.11	15.0%
BC065393	0.0501634	0.08	152.7%
BC065397	0.9446438	0.17	17.8%
BC065398	0	0.00	
BC065403	0	0.00	
BC065408	1.801176	0.47	26.1%
BC065777	2.376272	0.51	21.6%
BC065800	0.4094352	0.12	29.8%
BC066028	11.61586	0.38	3.3%
BC066100	8.022692	0.75	9.4%
BC066107	2.745546	0.23	8.3%
BC066135	0	0.00	
BC066226	0.08835318	0.03	38.3%
BC067010	6.171578	0.32	5.2%
BC067033	0.016725724	0.01	64.3%
BC067047	3.508728	0.40	11.3%
BC067068	2.392486	0.20	8.2%
BC067074	0.1506312	0.02	13.5%
BC067207	20.85062	0.85	4.1%
BC067249	0.01866328	0.04	223.6%
BC068110	12.07518	0.59	4.9%
BC068148	1.764306	0.29	16.5%
BC068157	6.682842	0.32	4.8%
BC068229	0.05421484	0.02	40.9%
BC068251	1.71785	0.14	8.0%
BC068281	2.098326	0.26	12.6%
BC068298	6.378464	0.49	7.7%
BC070417	5.14963	1.57	30.5%
BC070425	0.07116124	0.03	47.8%
BC070450	11.350228	1.16	10.2%
BC071190	0.3209672	0.08	25.4%
BC071263	0.01074588	0.02	140.1%
BC072557	0.2487648	0.06	23.2%
BC072601	0.0108238	0.01	68.9%
BC072620	6.234112	0.38	6.1%
BC072643	0.001655638	0.00	223.6%
BC072654	4.967724	0.39	7.9%
BC075635	0.3729694	0.05	14.1%

BC075654	0.15190714	0.11	69.2%
BC075659	0.3733996	0.08	20.7%
BC076616	0.7650668	0.31	40.4%
BC078436	12.53794	1.14	9.1%
BC079612	0.9064284	0.12	12.7%
BC079836	7.153166	0.41	5.7%
BC079845	4.049816	1.71	42.3%
BC079901	0	0.00	
BC079904	0	0.00	
BC080687	2.188172	0.35	15.8%
BC080695	0	0.00	
BC080696	0	0.00	
BC080769	0.010379374	0.00	34.6%
BC082544	0	0.00	
BC082591	0.02175676	0.01	30.8%
BC082597	0.5601844	0.09	16.8%
BC083001	0	0.00	
BC083116	0.6130008	0.31	49.8%
BC083167	3.059394	0.34	11.0%
BC083502	0.7978238	0.06	7.8%
BC084590	0	0.00	
BC085144	0.4613744	0.14	30.5%
BC085231	0.9848902	0.27	27.3%
BC085271	0	0.00	
BC085284	0	0.00	
BC085508	1.59677	0.14	8.6%
BC086315	1.775826	0.41	23.2%
BC086318	0.5170382	0.12	24.2%
BC086805	14.61244	0.68	4.6%
BC086906	0	0.00	
BC087938	0.06636972	0.04	63.2%
BC087945	2.489386	0.58	23.2%
BC087948	0	0.00	
BC088983	2.15049	0.24	11.3%
BC089377	0.5557706	0.16	29.4%
BC089491	0.5502704	0.07	13.6%
BC089513	0	0.00	
BC089597	0.0185955	0.03	137.0%
BC091771	1.3111138	0.20	14.9%
BC094257	0.21864158	0.11	51.4%
BC094334	11.1795	0.40	3.6%
BC094356	3.943264	0.73	18.5%
BC094408	0.1726946	0.12	71.7%
BC094509	0.08872832	0.03	34.1%
BC094555	8.1404	0.47	5.8%
BC094615	0.01593314	0.01	91.8%
BC094633	0.2654638	0.15	55.4%

BC094642	0	0.00	
BC094916	0	0.00	
BC094935	2.952866	0.31	10.4%
BC096037	2.569426	0.32	12.6%
BC096391	16.75056	2.69	16.1%
BC096410	14.672	1.72	11.7%
BC096411	0.026948582	0.02	90.5%
BC096416	0.06116382	0.02	35.3%
BC096478	0	0.00	
BC098222	0	0.00	
BC098228	0.189315	0.21	113.1%
BC099381	0	0.00	
BC099422	0.7339018	0.25	34.1%
BC099512	2.087954	0.35	17.0%
BC099513	0	0.00	
BC099522	0.31247126	0.38	122.8%
BC099523	0.08961442	0.11	120.0%
BC099526	0.01109792	0.02	223.6%
BC099528	1.997068	0.22	10.9%
BC099544	0.00910002	0.02	223.6%
BC099561	0.3589756	0.11	31.2%
BC099562	0.06481582	0.07	106.8%
BC099567	0.3466474	0.12	35.4%
BC099568	2.620624	0.43	16.4%
BC099571	1.165553	0.26	22.1%
BC099572	0	0.00	
BC099577	0.06519896	0.03	44.6%
BC099582	0	0.00	
BC099591	2.727712	0.27	9.7%
BC099610	0.04505936	0.04	89.2%
BC099611	1.2026966	0.47	39.1%
BC099953	0.0025797	0.01	223.6%
BC100303	0	0.00	
BC100417	0.0881706	0.06	71.4%
BC100427	0.179092	0.09	49.5%
BC100480	0	0.00	
BC100490	0.00874996	0.02	223.6%
BC100491	0.05912082	0.05	80.4%
BC100492	0.08578972	0.05	60.2%
BC100494	0	0.00	
BC100495	0	0.00	
BC100504	0	0.00	
BC100510	0	0.00	
BC100523	0.2076386	0.07	34.7%
BC100524	0.00985234	0.02	223.6%
BC100530	0	0.00	
BC100534	0.1657549	0.14	84.4%

BC100589	0	0.00	
BC106127	0.9432384	0.10	10.6%
BC107230	0	0.00	
BC107309	0	0.00	
BC107310	0.011631946	0.01	113.9%
BC107364	0	0.00	
BC108272	0	0.00	
BC108341	0.09797026	0.03	26.5%
BC109180	0.0028497	0.01	223.6%
BC112410	1.335538	0.16	11.6%
BC113126	0.00337312	0.01	223.6%
BC114417	0.453286	0.19	42.8%
BC114580	0	0.00	
BC115444	0	0.00	
BC115953	1.858054	0.30	15.9%
BC115961	0.011344558	0.01	112.8%
BC116234	3.680772	0.30	8.2%
BC116340	0	0.00	
BC117090	0	0.00	
BC119421	0	0.00	
BC119519	0.0022126	0.00	223.6%
BC120798	0	0.00	
BC125002	0.03379968	0.02	69.2%
BC126883	0	0.00	
BC126955	0.0147665	0.03	223.6%
BC127131	0.3945246	0.14	35.1%
BC128282	0.08861622	0.02	23.3%
BC128327	0.596028	0.17	28.3%
BC134356	187.024	29.46	15.8%
BC141885	0	0.00	
BC145649	0.06395078	0.03	52.0%
BC145724	0.2758424	0.04	14.4%
BC145794	0.01617452	0.02	137.9%
BC145836	0	0.00	
BC152372	0.0654182	0.09	139.7%
Bcam	12.00746	1.49	12.4%
Bcan	19.32954	2.80	14.5%
Bcap29	18.90674	1.54	8.1%
Bcap31	42.2837	1.77	4.2%
Bcap37	0.770515	0.18	23.2%
Bcar1	11.27284	0.28	2.5%
Bcar3	0.4956438	0.11	21.7%
Bcas1	0.479	0.17	34.9%
Bcas2	30.29728	1.27	4.2%
Bcas3	14.41952	1.21	8.4%
Bcat1	10.03622	0.29	2.8%
Bcat2	4.350646	0.26	6.0%

Bccip	12.65584	0.66	5.2%
Bcdin3d	1.849486	0.28	15.3%
Bcdo2	0.0663642	0.05	78.7%
Bche	1.557486	0.08	5.3%
Bckdha	12.66172	0.60	4.7%
Bckdhb	18.9571	1.14	6.0%
Bckdk	6.18231	0.33	5.3%
Bcl10	4.519352	0.28	6.1%
Bcl11a	24.83088	0.81	3.3%
Bcl11b	1.957344	0.22	11.5%
Bcl2	2.614974	0.13	4.9%
Bcl2a1a	0	0.00	
Bcl2a1b	0	0.00	
Bcl2a1c	0	0.00	
Bcl2a1d	0	0.00	
Bcl2l1	21.01832	0.81	3.8%
Bcl2l10	0	0.00	
Bcl2l11	3.840196	0.70	18.3%
Bcl2l12	0.5008484	0.06	13.0%
Bcl2l13	8.1617	0.19	2.4%
Bcl2l14	0.00946	0.01	154.4%
Bcl2l2	31.9122	2.09	6.6%
Bcl3	0.1696492	0.05	30.7%
Bcl6	4.480082	0.36	8.1%
Bcl6b	0.06711192	0.04	60.2%
Bcl7a	13.76156	0.69	5.0%
Bcl7b	11.40716	0.38	3.3%
BCL7B	1.0182292	0.23	22.7%
Bcl7c	5.324116	0.62	11.6%
Bcl9	8.665352	0.49	5.7%
Bcl9l	5.738828	0.59	10.4%
Bclaf1	27.76764	1.66	6.0%
Bclp2	0.00692992	0.02	223.6%
Bcmo1	0.00250276	0.01	223.6%
Bcng-1	0.0137845	0.00	18.9%
Bcor	4.348068	0.33	7.5%
Bcorl1	3.113042	0.16	5.0%
Bcr	10.89412	0.31	2.8%
Bcs1l	6.078186	0.29	4.7%
Bdh1	37.68262	5.04	13.4%
Bdh2	0.5360672	0.11	20.0%
Bdkrb1	0	0.00	
Bdkrb2	0.09404568	0.02	19.6%
Bdnf	8.01955	0.79	9.8%
Bdp1	4.526686	0.27	6.1%
Bean	4.81645	0.34	7.1%
Becn1	26.92972	1.26	4.7%

Begain	44.4126	3.10	7.0%
Best1	0.0028322	0.01	223.6%
Best2	0.01402318	0.02	115.7%
Best3	0.01469822	0.01	72.7%
Bet1	13.35944	0.52	3.9%
Bet1l	11.40162	0.69	6.0%
Bex1	30.86212	2.80	9.1%
Bex2	198.3306	8.40	4.2%
Bex4	4.62126	0.54	11.8%
Bex6	0	0.00	
Bfar	14.29624	0.41	2.9%
Bfsp1	0.04238102	0.03	59.6%
Bfsp2	0.04167794	0.04	104.3%
Bglap1	0.01484374	0.03	223.6%
Bglap2	0	0.00	
Bglap-rs1	0	0.00	
Bgn	12.700682	2.70	21.2%
Bhlhb2	9.473894	1.94	20.5%
Bhlhb3	5.13154	1.04	20.3%
Bhlhb4	0.03980528	0.05	123.2%
Bhlhb5	2.802566	0.18	6.3%
Bhlhb8	0.03739448	0.01	24.5%
Bhlhb9	37.86532	1.66	4.4%
Bhmt	0	0.00	
BHMT	0	0.00	
Bhmt2	0.0189905	0.01	50.7%
Bicc1	1.973134	0.22	11.1%
Bicd1	13.76792	1.07	7.8%
Bicd2	11.00014	0.50	4.6%
Bid	8.66568	0.47	5.4%
Bik	0.02262282	0.03	147.8%
Bin1	42.03722	1.42	3.4%
Bin3	3.210428	0.19	5.8%
Birc1f	0.022349672	0.01	50.1%
Birc2	10.063892	0.37	3.6%
Birc3	0.357463	0.05	13.0%
Birc4	13.62656	0.79	5.8%
Birc5	1.337503	0.26	19.5%
Birc6	12.82112	1.15	9.0%
Birc7	0	0.00	
Bivm	13.01274	0.39	3.0%
Blcap	58.04648	2.16	3.7%
Blk	0.01095946	0.02	223.6%
Blm	2.998356	0.13	4.3%
Blmh	42.91476	2.14	5.0%
Blnk	0	0.00	
Bloc1s1	7.515364	0.78	10.3%

Bloc1s2	10.542442	0.70	6.7%
Bloc1s3	6.029612	0.47	7.8%
Blr1	0.00425351	0.01	137.4%
Blvra	5.480458	0.59	10.8%
Blvrb	4.858056	0.66	13.6%
Blzf1	9.339542	4.29	46.0%
bmal1b	0.1610088	0.03	19.1%
Bmf	0.5489562	0.12	21.3%
Bmi1	7.876676	0.85	10.8%
Bmp1	3.198306	0.46	14.4%
Bmp10	0	0.00	
Bmp15	0.0263332	0.01	45.4%
Bmp2	1.213922	0.21	17.4%
Bmp2k	2.158576	0.15	7.2%
Bmp3	0.9621096	0.14	14.3%
Bmp4	1.356014	0.23	16.9%
Bmp5	0.0690232	0.04	51.6%
Bmp6	0.5084228	0.09	17.7%
Bmp7	2.713472	0.24	9.0%
Bmp8a	0.02670888	0.02	87.4%
Bmp8b	0.05704254	0.01	16.1%
Bmper	6.687552	0.52	7.8%
Bmpr1a	10.62828	0.44	4.1%
Bmpr1b	1.011219	0.08	8.3%
Bmpr2	19.32776	1.45	7.5%
bMRP64	0.3829838	0.16	40.6%
Bms1	5.532596	0.42	7.5%
Bmx	0.01032094	0.02	154.3%
Bmyc	30.3465	2.46	8.1%
Bnc1	0.022190726	0.01	60.1%
Bnc2	0.3528698	0.11	31.8%
Bnip1	8.256082	1.01	12.3%
Bnip2	7.554266	0.65	8.6%
Bnip3	35.72546	5.51	15.4%
Bnip3l	15.37456	0.75	4.9%
Bnipl	0.05026162	0.04	72.2%
Boc	2.136966	0.32	14.8%
Bok	5.05673	0.82	16.1%
Bola1	6.678256	0.50	7.4%
Bola2	24.39468	2.13	8.7%
Bola3	7.01987	0.40	5.7%
Boll	0.00906274	0.02	223.6%
Bop1	11.93328	1.97	16.5%
Bpag1	17.75536	1.01	5.7%
Bpag1-n	0.09555828	0.01	7.6%
Bpgm	11.13582	1.87	16.8%
Bphl	9.84384	0.60	6.0%

Bpi	0	0.00	
Bpil1	0	0.00	
Bpil2	0.16393684	0.07	45.2%
Bpil3	0	0.00	
Bpnt1	22.94506	1.48	6.4%
BPOZ	1.2886246	0.39	30.1%
Bptf	11.89404	0.81	6.8%
Braf	17.15614	1.11	6.5%
Brap	9.72354	0.36	3.7%
Brca1	0.5439472	0.06	11.3%
Brca2	0.8505276	0.15	17.7%
Brcc3	13.46522	1.10	8.2%
Brctd1	3.33293	0.38	11.5%
Brd1	0.3065352	0.06	21.0%
Brd2	48.6478	1.77	3.6%
Brd3	19.10898	3.21	16.8%
Brd4	18.76646	0.99	5.3%
Brd7	12.2736	0.75	6.1%
Brd8	13.72402	0.38	2.8%
Brd9	14.74728	0.43	2.9%
Brdt	0.975277	0.04	3.9%
Bre	32.05068	2.97	9.3%
Brf1	6.573318	0.23	3.4%
Brf2	6.566392	0.70	10.6%
Bri3	8.110148	0.89	10.9%
Bri3bp	31.56276	1.08	3.4%
Brip1	0.09536624	0.02	17.4%
Brms1	4.075936	0.79	19.3%
Brms1l	12.36778	0.54	4.4%
Brp16	4.557886	0.18	3.9%
Brp44	44.72836	1.33	3.0%
Brp44l	7.872688	0.56	7.2%
Brpf1	4.919188	0.42	8.4%
Brpf3	4.218814	0.20	4.7%
Brs3	0.00584498	0.01	137.6%
Brsk1	60.04292	3.25	5.4%
Brsk2	28.06386	1.18	4.2%
Brul4	13.341458	3.96	29.7%
Brunol4	62.5624	0.90	1.4%
Brunol5	0.351501	0.08	23.1%
Brunol6	9.354828	0.86	9.2%
Brwd1	30.26658	2.04	6.7%
Brwd2	12.45238	0.43	3.5%
Brwd3	1.533458	0.09	5.9%
BSAC2A	0.001718039	0.00	119.9%
Bscl2	67.26912	2.38	3.5%
Bsdc1	12.95126	0.42	3.3%

Bsg	225.0836	7.37	3.3%
Bsn	7.846538	0.52	6.7%
Bsnd	0	0.00	
Bspry	0.5152168	0.09	17.8%
Bst1	0.1835852	0.05	28.6%
Bst2	0.9624126	0.23	24.2%
Bsx	0	0.00	
Btaf1	3.99821	0.18	4.6%
Btbd1	12.78678	0.54	4.2%
Btbd10	13.26388	1.25	9.4%
Btbd11	16.98874	0.33	2.0%
Btbd12	4.872848	0.47	9.6%
Btbd14a	27.81262	2.24	8.1%
Btbd14b	18.65368	0.56	3.0%
Btbd16	0	0.00	
Btbd2	44.60142	3.56	8.0%
Btbd3	15.92266	0.99	6.2%
Btbd6	9.054256	0.40	4.4%
Btbd7	3.19198	0.27	8.5%
Btbd9	16.26892	1.58	9.7%
Btc	0.0227965	0.02	67.7%
Btd	3.499716	0.54	15.4%
Btf3	7.447216	4.71	63.3%
Btf3l4	6.274022	0.27	4.3%
Btg1	13.72162	0.93	6.8%
Btg2	3.485962	0.26	7.4%
Btg3	0.09657092	0.08	87.4%
Btg4	0.01458292	0.03	223.6%
Btk	0	0.00	
Btla	0.0704732	0.02	34.0%
Btn1a1	0.2188776	0.04	17.6%
Btn2a2	0.08115012	0.05	64.5%
Btnl1	0	0.00	
Btnl2	0	0.00	
Btnl7	0.03716022	0.03	72.8%
Btnl9	0.00238808	0.01	223.6%
Btrc	32.77616	0.41	1.2%
Bub1	0.4952956	0.06	11.4%
Bub1b	0.5632502	0.10	18.0%
Bub3	20.51318	0.98	4.8%
Bud13	2.97145	0.28	9.3%
Bud31	27.7697	1.66	6.0%
Butr1	0.001503464	0.00	223.6%
Bves	1.066439	0.17	16.2%
Bxdc1	3.291222	0.44	13.5%
Bxdc2	11.45826	0.41	3.5%
Bxdc5	5.862224	0.52	9.0%

BY080835	0.33971	0.07	20.9%
Bysl	6.710812	1.07	15.9%
Bzrap1	15.5237	0.85	5.5%
Bzrpl1	0	0.00	
Bzw1	65.95318	7.79	11.8%
Bzw2	25.53142	3.72	14.6%
C030003D03Rik	2.547504	0.25	9.8%
C030006K11Rik	5.595954	0.30	5.4%
C030011J08Rik	0.01643358	0.00	22.7%
C030011O14Rik	35.95356	3.44	9.6%
C030014K22Rik	6.606192	0.32	4.8%
C030014M07Rik	19.49736	2.20	11.3%
C030017K20Rik	0.0464712	0.02	32.5%
C030018G13Rik	5.535938	0.90	16.2%
C030019F02Rik	3.46513	0.33	9.6%
C030019I05Rik	0.07926796	0.04	45.4%
C030027K23Rik	0.4480594	0.08	17.5%
C030030A07Rik	1.390944	0.19	13.7%
C030039L03Rik	3.932366	0.18	4.6%
C030045D06Rik	0.331251	0.12	35.6%
C030046E11Rik	11.52054	0.70	6.1%
C030046I01Rik	54.72034	5.81	10.6%
C030048B08Rik	2.25716	0.29	12.8%
C130022K22Rik	6.22431	0.31	5.0%
C130023O10Rik	0	0.00	
C130026I21Rik	0	0.00	
C130026L21Rik	0.5098534	0.08	15.2%
C130032J12Rik	9.32613	0.41	4.4%
C130034I18Rik	0.08803288	0.04	50.5%
C130038G02Rik	4.877416	0.29	5.9%
C130039O16Rik	2.307236	0.28	12.2%
C130050O18Rik	0.02140524	0.03	134.9%
C130057D23Rik	0.00723124	0.02	223.6%
C130060K24Rik	0.2633624	0.06	20.9%
C130069I09Rik	0.3275302	0.10	31.3%
C130073F10Rik	0.01478168	0.02	141.3%
C130074G19Rik	0.255865	0.04	14.2%
C130076O07Rik	16.85994	0.77	4.6%
C130079G13Rik	0	0.00	
C130090K23Rik	0.2506862	0.07	26.6%
C184	0.3339976	0.13	38.0%
C1d	19.24502	1.36	7.1%
C1galt1	5.13195	0.32	6.3%
C1galt1c1	10.08002	0.68	6.8%
C1qa	0	0.00	
C1qb	0	0.00	
C1qbp	31.4963	0.95	3.0%

C1qc	0	0.00	
C1qdc2	0.4206002	0.13	31.0%
C1ql1	2.776306	0.56	20.1%
C1ql2	2.24886	0.46	20.4%
C1ql3	7.29172	0.41	5.6%
C1ql4	1.3943776	0.45	32.1%
C1qtnf1	0.0633709	0.02	33.4%
C1qtnf2	0.12584138	0.09	69.6%
C1qtnf3	0.577784	0.24	41.0%
C1qtnf4	14.97476	0.74	4.9%
C1qtnf5	0.09186592	0.05	49.7%
C1qtnf6	4.181216	0.61	14.5%
C1qtnf7	0.2775418	0.04	14.9%
C1qtnf9	0.01501986	0.01	63.3%
C1r	0.3802546	0.16	43.3%
C1rb	0.08494608	0.03	36.2%
C1rl	0.08496144	0.05	59.5%
C1s	0.9450538	0.36	38.1%
C2	0.13894316	0.04	30.5%
C21orf63	0.02367924	0.01	36.0%
C21orf66	12.72452	0.85	6.7%
C21orf80	0.3940676	0.09	21.8%
C21orf83	0.3371534	0.19	55.6%
C230004F18Rik	4.026816	0.48	11.8%
C230021P08Rik	10.159224	0.68	6.7%
C230029F24Rik	0	0.00	
C230052I12Rik	1.281054	0.15	11.9%
C230055K05Rik	0.07541916	0.04	52.4%
C230071H18Rik	0.033291	0.03	96.4%
C230078M08Rik	1.0566232	0.22	20.5%
C230078M14Rik	5.0711	0.51	10.1%
C230081A13Rik	0.08397728	0.06	69.9%
C230093N12Rik	9.973796	0.51	5.1%
C230094A16Rik	6.901112	0.41	5.9%
C230095G01Rik	0.00410153	0.01	138.7%
C230096C10Rik	19.30626	4.15	21.5%
C230097I24Rik	0.3767212	0.12	31.2%
C2ta	0.002310305	0.00	150.8%
C3	0.07107484	0.05	67.2%
C330001K17Rik	0.7217074	0.06	8.8%
C330002I19Rik	62.71848	4.02	6.4%
C330005M16Rik	0.14494826	0.05	34.1%
C330006K01Rik	4.910926	0.34	7.0%
C330007P06Rik	6.30148	0.22	3.5%
C330011K17Rik	1.971636	0.18	9.0%
C330016O10Rik	2.638126	0.41	15.5%
C330018D20Rik	2.623598	0.20	7.8%

C330019G07Rik	6.489874	0.12	1.9%
C330019L16Rik	0.698011	0.06	9.0%
C330021F23Rik	2.440234	0.57	23.3%
C330023M02Rik	14.89506	1.00	6.7%
C330024D12Rik	0.0101141	0.02	223.6%
C330027C09Rik	1.303184	0.08	6.3%
C330043M08Rik	0.7949242	0.07	9.4%
C330046G03Rik	0.07057164	0.03	41.8%
C3ar1	0.0110841	0.01	118.3%
C430003P19Rik	11.7556	0.91	7.8%
C430004E15Rik	3.096932	0.07	2.2%
C430014M02Rik	1.978348	0.18	9.0%
C430048L16Rik	2.688198	0.32	12.1%
C4a	0.1750324	0.06	34.5%
C4b	0.324224	0.03	9.1%
C4bp	0.03533294	0.03	71.6%
C530008M07Rik	0.06123586	0.02	27.3%
C530008M17Rik	4.9181	0.55	11.2%
C530028I08Rik	0.6580488	0.12	17.9%
C530028O21Rik	29.40886	1.24	4.2%
C530043G21Rik	28.793	0.39	1.3%
C530044N13Rik	5.711564	0.15	2.7%
C5ar1	0.11162548	0.05	44.7%
C6	0	0.00	
C630004H02Rik	30.2157	0.80	2.6%
C630007B19Rik	13.06058	0.59	4.5%
C630028N24Rik	0.8511444	0.11	13.5%
C630035N08Rik	9.68775	0.74	7.6%
C630041L24Rik	0.01635964	0.02	112.7%
C730007P19Rik	0	0.00	
C730025P13Rik	5.79893	0.70	12.1%
C730027E14Rik	0.0175563	0.02	93.3%
C730027J19Rik	0.02498136	0.03	105.3%
C730027P07Rik	0.05283004	0.03	49.5%
C730034F03Rik	0.2587812	0.07	26.1%
C730036D15Rik	0.0047715	0.01	223.6%
C730048C13Rik	0.003710655	0.01	215.3%
C76566	1.456854	0.20	13.8%
C77080	13.14444	1.84	14.0%
C77370	1.0914454	0.25	23.2%
C78339	15.30622	1.62	10.6%
C78409	1.985604	0.39	19.4%
C79127	0.1745834	0.04	22.4%
C79267	3.9802	0.29	7.3%
C79407	0.5122206	0.07	13.7%
C80913	17.59106	0.86	4.9%
C85492	14.26076	0.94	6.6%

C85627	0.00475806	0.01	223.6%
C86695	0	0.00	
C87414	0	0.00	
C87436	4.357026	0.44	10.0%
C87499	0	0.00	
C8a	0.001666806	0.00	223.6%
C8b	0.04166204	0.03	65.5%
C8g	1.0319614	0.25	24.0%
C9	0.00519218	0.01	223.6%
C920005C14Rik	0.1452776	0.02	13.3%
C920006C10Rik	25.9769	3.39	13.0%
C920016K16Rik	6.792734	0.45	6.6%
Cab39	52.0194	1.35	2.6%
Cab39l	5.246716	0.23	4.4%
Cabc1	8.355944	1.18	14.1%
Cabin1	12.301834	1.88	15.3%
Cables1	2.107686	0.15	7.1%
Cables2	8.446534	0.57	6.8%
Cabp1	21.2691	1.97	9.3%
Cabp2	0	0.00	
Cabp4	0.1785132	0.04	23.5%
Cabp5	0.02724404	0.01	26.5%
Cabp7	3.856968	1.01	26.1%
Cabyr	1.0241816	0.21	20.7%
Cachd1	11.85576	0.13	1.1%
Cacna1a	18.84142	1.06	5.6%
Cacna1b	10.296098	0.40	3.9%
Cacna1c	3.283196	0.28	8.4%
Cacna1d	2.668108	0.06	2.2%
cacna1d	0.6395856	0.23	35.3%
Cacna1e	2.00191	0.19	9.6%
Cacna1f	0.023997436	0.02	71.1%
Cacna1g	4.091266	0.40	9.9%
Cacna1h	2.33977	0.41	17.5%
Cacna1i	1.760804	0.24	13.7%
Cacna1s	0.00217104	0.00	138.6%
Cacna2d1	16.37378	0.97	5.9%
Cacna2d2	18.70052	0.88	4.7%
Cacna2d3	8.87771	0.49	5.5%
Cacna2d4	0.181876	0.03	17.8%
Cacnb1	15.89976	0.75	4.7%
Cacnb2	2.56098	0.35	13.8%
Cacnb3	15.69302	1.82	11.6%
Cacnb4	15.19204	1.50	9.9%
Cacng1	0	0.00	
Cacng2	25.98072	1.35	5.2%
Cacng3	18.64872	1.63	8.8%

Cacng4	16.44202	3.91	23.8%
Cacng5	6.464152	0.45	7.0%
Cacng6	0.02726172	0.04	152.4%
Cacng7	55.51874	3.53	6.4%
Cacng8	5.993492	0.67	11.1%
Cacybp	52.9613	3.76	7.1%
Cad	4.431868	0.23	5.3%
Cadm1	26.18688	0.74	2.8%
Cadm2	43.88658	1.91	4.3%
Cadm3	92.23622	1.40	1.5%
Cadm4	82.267	5.92	7.2%
Cadps	82.74876	1.39	1.7%
Cadps2	3.285718	0.26	8.0%
Cage1	0.4065778	0.05	12.3%
Calb1	10.27411	0.82	8.0%
Calb2	15.84312	1.45	9.2%
Calca	19.40314	4.11	21.2%
Calcb	0.1627122	0.10	60.5%
calcium_channel	0.2785842	0.11	40.6%
Calcoco1	11.441922	1.47	12.9%
Calcoco2	0	0.00	
Calcr	0.7212938	0.12	16.4%
Calcrl	1.89887	0.18	9.6%
Cald1	15.64762	1.66	10.6%
Calm1	556.463	10.31	1.9%
Calm2	315.9716	19.13	6.1%
Calm3	603.6144	28.56	4.7%
Calm4	0	0.00	
Calm5	0	0.00	
Calml3	0.02358706	0.02	101.3%
Calml4	0.1786238	0.07	41.5%
Caln1	16.60036	1.34	8.1%
calp80	0.321138	0.09	28.4%
Calr	175.1958	7.38	4.2%
Calr3	0.914887	0.09	9.7%
Calr4	0.1165866	0.04	35.2%
Calu	42.7225	1.42	3.3%
Camk1	7.284892	0.51	6.9%
Camk1d	14.04388	0.71	5.0%
Camk1g	13.76628	0.80	5.8%
Camk2a	4.03458	0.64	16.0%
Camk2b	107.09824	5.21	4.9%
Camk2d	81.81252	1.57	1.9%
Camk2g	73.6673	3.63	4.9%
Camk2n1	54.82786	3.62	6.6%
Camk2n2	117.5138	7.88	6.7%
Camk4	12.93938	0.83	6.4%

Camkk1	18.4143	1.62	8.8%
Camkk2	8.062674	0.25	3.1%
Camkv	2.690296	0.17	6.2%
Caml	17.20996	1.91	11.1%
Camp	0	0.00	
cAMP-GEFII	0.2131389	0.08	36.9%
Camsap1	18.9024	1.28	6.8%
Camsap1l1	26.74484	0.91	3.4%
Camta1	53.85298	2.63	4.9%
Camta2	26.94628	1.78	6.6%
Cand1	41.80242	1.07	2.5%
Cand2	1.78736	0.04	2.2%
Cant1	12.53976	0.34	2.7%
Canx	184.747	5.14	2.8%
Cap	0.6340066	0.19	29.4%
Cap1	56.61758	3.22	5.7%
Cap2	1.461058	0.12	8.1%
Caper	8.813488	2.06	23.3%
Capg	0.13471344	0.06	43.7%
Capn1	5.111698	0.76	14.9%
Capn10	8.388956	0.35	4.2%
Capn11	0.03997266	0.02	42.4%
capn12	0.17777562	0.18	98.7%
Capn12	0.15185408	0.15	99.2%
Capn13	0.03174914	0.04	119.6%
Capn2	24.36238	1.02	4.2%
Capn3	0.9207456	0.39	42.0%
Capn5	17.02078	1.02	6.0%
Capn6	5.35171	1.20	22.5%
Capn7	15.64092	1.22	7.8%
Capn8	0.01772672	0.01	82.8%
Capn9	0.01685318	0.02	112.5%
Capns1	96.24558	5.02	5.2%
Caprin1	84.93396	1.37	1.6%
Caprin2	3.282736	0.21	6.4%
Caps2	0.1654918	0.05	28.0%
Capsl	0.06785294	0.05	75.8%
Capza1	3.227928	0.31	9.7%
Capza2	86.37798	2.21	2.6%
Capza3	0.01155452	0.02	141.2%
Capzb	79.06262	3.60	4.6%
Car1	0.0407297	0.03	63.6%
Car10	63.12042	4.37	6.9%
Car11	14.04582	0.54	3.8%
Car12	0.4462672	0.18	39.5%
Car13	0.212628	0.05	22.4%
Car14	0.08130798	0.08	97.5%

Car15	0.5084738	0.13	25.9%
Car2	6.362234	0.74	11.6%
Car3	0.3193774	0.10	31.2%
Car4	0.01344318	0.02	147.2%
Car5a	0.00274004	0.01	223.6%
Car5b	0.2967014	0.07	22.3%
Car6	0.03116976	0.01	45.0%
Car7	1.267744	0.25	20.0%
Car8	2.105834	0.21	10.1%
Car9	0.00792988	0.01	145.4%
Card10	0.5389438	0.04	6.7%
Card11	0.002815638	0.00	138.0%
Card14	0.014045442	0.01	61.2%
Card15	0.01267914	0.01	65.4%
Card6	0.1558922	0.02	11.2%
Card9	0.08242102	0.02	27.4%
Carf	4.388694	0.51	11.7%
Carhsp1	9.849788	0.89	9.0%
Carkl	0.8985578	0.04	4.3%
Carm1	14.5015	0.75	5.2%
Cars	20.02046	1.68	8.4%
Cars2	5.002384	0.06	1.1%
Cartpt	1.25719	0.32	25.7%
Casc1	0.043660568	0.06	142.7%
Casc3	5.966664	1.04	17.5%
Casc4	54.57522	1.31	2.4%
Casc5	0.2075128	0.05	22.7%
Casd1	21.90102	0.72	3.3%
Cask	52.78174	3.35	6.3%
Caskin1	41.54494	1.60	3.8%
Caskin2	0.4878658	0.07	14.3%
Casp1	0.04988442	0.02	48.9%
Casp12	0.4956734	0.13	25.5%
Casp14	0.00361214	0.01	223.6%
Casp2	4.763886	0.21	4.4%
Casp3	22.8795	3.21	14.0%
Casp4	0.04047712	0.02	50.3%
Casp6	1.12132	0.18	16.2%
Casp7	4.79302	0.83	17.3%
Casp8	0.2465848	0.05	19.5%
Casp8ap2	2.5725	0.14	5.6%
Casp9	6.442472	0.43	6.7%
Casq1	0.03275416	0.03	105.7%
Casq2	0.3876186	0.15	39.3%
Casr	0.003795246	0.01	147.3%
Cast	1.2021696	0.23	19.5%
Casz1	0.502263	0.08	16.3%

Cat	23.51676	1.44	6.1%
CAT56	0.4533518	0.10	21.5%
CAT60	2.522316	0.38	15.2%
Catsper1	0.00267912	0.01	223.6%
Catsper2	1.2289886	0.18	14.4%
Catsper3	0.1649311	0.04	23.9%
Catsper4	0.00357234	0.01	223.6%
Cav1	0.7816208	0.14	18.1%
CaV1.2-a	0.2022768	0.06	27.9%
Cav2	1.1536046	0.15	13.2%
Cav3	0	0.00	
Cbara1	18.95014	0.98	5.2%
Cbfa2t1h	0.2994338	0.03	9.0%
Cbfa2t2	9.579586	0.30	3.1%
Cbfa2t3h	1.511612	0.06	4.1%
Cbfb	6.464482	0.50	7.8%
Cbl	2.853084	0.22	7.6%
Cblb	3.57657	0.15	4.1%
Cblc	0.03039282	0.01	45.2%
Cbll1	6.536362	0.46	7.0%
Cbln1	36.39742	3.50	9.6%
Cbln2	19.23818	0.81	4.2%
Cbln3	0.766959	0.19	25.4%
Cbln4	17.55406	1.80	10.3%
cbp146	0.227074	0.09	37.8%
cbp82	13.53488	0.41	3.0%
Cbr1	35.04954	1.81	5.2%
Cbr2	0.00509934	0.01	223.6%
Cbr3	1.395844	0.19	13.9%
Cbr4	5.420788	0.32	5.9%
Cbs	1.59267	0.23	14.1%
Cbwd1	6.5871	0.51	7.8%
Cbx1	10.012454	0.92	9.2%
Cbx2	0.865209	0.20	23.2%
Cbx3	4.597494	0.48	10.5%
Cbx4	22.26894	0.57	2.6%
Cbx5	37.4231	2.22	5.9%
Cbx6	73.3801	9.80	13.4%
Cbx7	13.2756	0.99	7.4%
Cbx8	4.205886	0.35	8.2%
Cby1	16.91082	0.82	4.9%
CC1	3.96519	0.56	14.1%
Cc2d1a	17.6293	0.80	4.6%
Cc2d1b	4.252668	0.20	4.6%
Ccar1	15.0368	0.72	4.8%
Ccbe1	0.2000746	0.03	14.6%
Ccbl1	4.540332	0.31	6.9%

Ccbl2	1.633162	0.11	6.8%
Ccbp2	0.047528144	0.03	68.4%
Ccdc100	15.30672	0.33	2.2%
Ccdc101	3.355974	0.54	16.2%
Ccdc102a	0.6397536	0.10	15.2%
Ccdc103	0.2887008	0.05	16.6%
Ccdc104	37.81118	0.92	2.4%
Ccdc106	13.62758	0.77	5.7%
Ccdc108	0.07220626	0.03	35.2%
Ccdc109a	11.25762	0.41	3.6%
Ccdc109b	1.375602	0.19	13.8%
Ccdc11	0.0229999	0.02	94.6%
Ccdc110	0.026600222	0.02	67.3%
Ccdc111	0.8165534	0.32	38.8%
Ccdc112	1.590497	0.55	34.4%
Ccdc113	0.04030634	0.03	71.8%
Ccdc114	0.0648809	0.03	52.7%
Ccdc115	3.472028	0.12	3.4%
Ccdc116	3.615586	1.17	32.4%
Ccdc117	4.24546	0.31	7.4%
Ccdc12	2.613062	0.29	11.1%
Ccdc120	2.955328	0.26	8.7%
Ccdc122	0.353849	0.10	29.5%
Ccdc123	1.800962	0.21	11.8%
Ccdc124	25.98726	3.14	12.1%
Ccdc125	0.11339438	0.03	30.7%
Ccdc126	4.941604	0.47	9.6%
Ccdc127	15.15852	0.61	4.0%
Ccdc128	13.93806	0.76	5.4%
Ccdc129	0.025062346	0.01	40.8%
Ccdc13	0.06014394	0.04	72.8%
Ccdc130	3.03706	0.40	13.1%
Ccdc131	9.618224	0.78	8.1%
Ccdc132	17.35556	0.85	4.9%
Ccdc134	2.716136	0.19	6.8%
Ccdc135	0.06572276	0.06	94.2%
Ccdc136	12.5738	0.53	4.2%
Ccdc137	3.644652	0.18	4.9%
Ccdc138	0.13744212	0.06	45.2%
Ccdc139	8.797974	1.20	13.6%
Ccdc14	0.0501172	0.02	43.2%
Ccdc142	2.32864	0.57	24.6%
Ccdc144b	0.01879722	0.03	155.5%
Ccdc15	0.8409536	0.21	24.8%
Ccdc16	3.35516	0.20	5.9%
Ccdc18	0.2155816	0.13	60.1%
Ccdc19	0.06636154	0.01	21.0%

Ccdc21	1.58517	0.13	8.4%
Ccdc22	5.249712	0.50	9.5%
Ccdc23	9.656282	0.60	6.2%
Ccdc24	0.4728698	0.18	38.1%
Ccdc25	15.79766	0.92	5.8%
Ccdc27	0.0056231	0.01	223.6%
Ccdc28a	3.43764	0.91	26.4%
Ccdc28b	13.83298	2.23	16.1%
Ccdc3	1.0297584	0.27	26.4%
Ccdc32	12.0769	0.95	7.9%
Ccdc33	0.00704864	0.01	106.4%
Ccdc34	3.743092	0.37	10.0%
Ccdc36	0.028840738	0.02	75.6%
Ccdc37	0.2341184	0.03	12.5%
Ccdc38	0.5137686	0.08	15.8%
Ccdc39	1.315496	0.15	11.5%
Ccdc40	0.326478	0.12	35.3%
Ccdc41	7.57333	0.44	5.8%
Ccdc42	0	0.00	
Ccdc43	5.706554	0.57	9.9%
Ccdc44	7.070136	0.24	3.4%
Ccdc45	3.798676	0.92	24.3%
Ccdc46	0.3249192	0.05	15.1%
Ccdc47	24.13494	0.77	3.2%
Ccdc49	5.169276	0.27	5.3%
Ccdc5	3.11337	0.37	11.9%
Ccdc50	20.61924	1.76	8.5%
Ccdc51	1.742272	0.08	4.7%
Ccdc52	1.820578	0.26	14.2%
Ccdc53	6.341146	0.65	10.2%
Ccdc54	0	0.00	
Ccdc55	4.290918	0.46	10.8%
Ccdc56	47.09734	2.43	5.2%
Ccdc57	1.278172	0.15	12.1%
Ccdc58	2.113272	0.13	6.1%
Ccdc59	6.622968	0.57	8.5%
Ccdc60	0.02589832	0.01	38.8%
Ccdc63	0.01499784	0.02	142.5%
Ccdc64	10.92262	0.45	4.1%
Ccdc64b	0.01091981	0.01	119.0%
Ccdc65	3.83013	0.31	8.0%
Ccdc66	2.097022	0.25	11.9%
Ccdc67	0.013794802	0.01	70.5%
Ccdc68	0.04742954	0.04	88.4%
Ccdc69	0.0603295	0.03	47.8%
Ccdc7	0	0.00	
Ccdc70	0	0.00	

Ccdc71	10.83671	0.81	7.5%
Ccdc72	11.56456	1.54	13.3%
Ccdc73	1.84136	0.14	7.6%
Ccdc76	1.835264	0.17	9.2%
Ccdc77	1.345184	0.15	11.3%
Ccdc79	0.11260462	0.03	30.4%
Ccdc80	1.51742	0.19	12.6%
Ccdc81	0.1592601	0.05	34.2%
Ccdc82	0	0.00	
Ccdc83	0	0.00	
Ccdc84	2.567238	0.36	13.9%
Ccdc85a	14.71876	0.39	2.7%
Ccdc85b	6.705474	0.25	3.8%
Ccdc86	2.736016	0.22	8.2%
Ccdc87	0.5325064	0.13	24.8%
Ccdc88a	12.74428	1.39	10.9%
Ccdc88b	0.02713246	0.01	31.9%
Ccdc88c	1.631778	0.21	12.9%
Ccdc9	5.131612	0.64	12.5%
Ccdc90a	0.8116638	0.21	26.1%
Ccdc90b	4.838628	0.26	5.3%
Ccdc91	13.61754	0.90	6.6%
Ccdc92	26.9205	2.05	7.6%
Ccdc93	3.92367	0.36	9.1%
Ccdc94	1.860062	0.12	6.4%
Ccdc95	12.5876	0.91	7.2%
Ccdc96	0.3288716	0.12	37.1%
Ccdc97	12.49528	0.42	3.3%
Ccdc98	1.462434	0.29	19.8%
Ccdc99	0.3829168	0.07	19.3%
Cchcr1	0.5055776	0.07	14.6%
Ccin	0.05022238	0.02	45.9%
Cck	0.2775696	0.10	34.7%
Cckar	0.12405574	0.05	41.7%
Cckbr	2.429696	0.40	16.4%
Ccl1	0	0.00	
Ccl11	0.0822279	0.10	123.5%
Ccl12	0	0.00	
Ccl17	1.0771414	0.45	41.8%
Ccl19	0.01539298	0.02	140.8%
Ccl2	0.0602388	0.09	143.3%
Ccl20	0.12728164	0.17	136.7%
Ccl21a	0	0.00	
Ccl21b	0	0.00	
Ccl21c	0	0.00	
Ccl22	0.00716792	0.01	137.6%
Ccl24	0.006918	0.02	223.6%

Ccl25	4.321308	0.34	7.9%
Ccl26l	0	0.00	
Ccl27	4.561266	4.27	93.6%
Ccl28	0	0.00	
Ccl3	0	0.00	
Ccl4	0	0.00	
Ccl5	0	0.00	
Ccl6	0	0.00	
Ccl7	0.03204518	0.03	108.3%
Ccl8	0	0.00	
Ccl9	0.00269052	0.01	223.6%
Ccm2	12.3806	0.66	5.4%
Ccn1	1.843566	0.68	37.0%
Ccna1	0.12072718	0.03	26.7%
Ccna2	2.511498	0.22	8.6%
Ccnb1	0.516554	0.10	19.2%
Ccnb2	0.8230556	0.16	18.9%
Ccnb3	0	0.00	
Ccnc	14.80932	1.22	8.2%
Ccnd1	3.74144	0.35	9.5%
Ccnd2	19.7834	1.28	6.5%
Ccnd3	4.269346	0.63	14.9%
Ccndbp1	25.33312	0.86	3.4%
Ccne1	5.843288	0.56	9.5%
Ccne2	6.815744	1.87	27.4%
Ccnf	0.945896	0.17	18.5%
Ccng1	27.76356	2.00	7.2%
Ccng2	10.51794	1.66	15.8%
Ccnh	23.83198	1.43	6.0%
Ccni	66.8044	3.36	5.0%
Ccnj	3.04891	0.16	5.2%
Ccnjl	0.2580798	0.11	43.4%
Ccnk	7.896178	0.49	6.2%
Ccnl1	6.409514	1.05	16.3%
Ccnl2	19.00186	1.51	7.9%
Ccno	0.3054108	0.09	28.4%
Ccnt1	7.421892	0.78	10.5%
Ccnt2	12.13536	0.80	6.6%
Ccny	22.90928	0.85	3.7%
Ccnyl1	1.459764	0.24	16.4%
Ccpg1	17.96834	1.51	8.4%
Ccr1	0	0.00	
Ccr10	0.3548086	0.09	26.2%
Ccr1l1	0	0.00	
Ccr2	0	0.00	
Ccr3	0.00363876	0.01	223.6%
Ccr4	0	0.00	

Ccr5	0	0.00	
Ccr6	0	0.00	
Ccr7	0.02356304	0.01	44.7%
Ccr8	0	0.00	
Ccr9	0.0061912	0.01	136.9%
Ccrk	5.34717	0.26	4.8%
Ccrl1	5.070032	1.18	23.3%
Ccrl2	0.01530454	0.02	102.0%
Ccrn4l	13.99892	1.55	11.1%
Ccs	10.89202	1.09	10.0%
Cct2	85.52106	4.49	5.3%
Cct3	49.2472	3.73	7.6%
Cct4	63.00412	4.84	7.7%
Cct5	111.6672	5.95	5.3%
Cct6a	106.2918	4.75	4.5%
Cct6b	0.06884868	0.01	19.7%
Cct7	80.09318	4.33	5.4%
Cct8	93.91558	3.89	4.1%
Cd109	0.1780066	0.03	14.9%
Cd14	0.17117488	0.06	36.7%
Cd151	8.790732	0.66	7.6%
Cd160	0.01350116	0.01	93.7%
Cd163	0.001855642	0.00	223.6%
Cd163l1	0	0.00	
Cd164	24.15332	2.67	11.0%
Cd164l2	1.806828	0.21	11.5%
Cd177	0.01122072	0.01	113.9%
Cd180	0.0053322	0.01	137.6%
Cd19	0.0021787	0.00	223.6%
Cd1d1	3.444622	0.43	12.6%
Cd1d2	0.32336	0.06	18.5%
Cd2	0	0.00	
Cd200	66.0854	3.77	5.7%
Cd200r1	0.00281514	0.01	223.6%
Cd200r2	0.188926	0.06	33.4%
Cd200r3	0.00281106	0.01	223.6%
Cd200r4	0	0.00	
Cd207	0.01787964	0.02	110.4%
Cd209a	0	0.00	
Cd209b	0	0.00	
Cd209c	0	0.00	
Cd209d	0	0.00	
Cd209e	0	0.00	
Cd209f	0	0.00	
Cd209g	0	0.00	
Cd22	0	0.00	
Cd226	0.01913082	0.01	74.1%

Cd244	0.006613966	0.01	77.6%
Cd247	0.9965038	0.49	49.1%
Cd248	0.629966	0.20	32.2%
Cd24a	62.45246	6.95	11.1%
Cd27	0.01388586	0.02	137.5%
Cd274	0.5108194	0.08	16.0%
Cd276	3.292508	0.34	10.4%
Cd28	0.008963926	0.01	102.5%
Cd2ap	4.64426	0.19	4.0%
Cd2bp2	13.4584	0.61	4.5%
CD3_[zeta]/[eta]	0.08809564	0.07	75.8%
Cd300a	0.00150255	0.00	223.6%
Cd300c	0	0.00	
Cd300d	0	0.00	
Cd300e	0.0038974	0.01	223.6%
Cd300lb	0	0.00	
Cd300lf	0	0.00	
Cd300lg	0.00449546	0.01	223.6%
Cd302	2.547152	0.29	11.4%
Cd320	2.665148	0.36	13.5%
Cd33	0	0.00	
Cd34	0.6481808	0.18	28.1%
Cd36	0.03043334	0.03	106.8%
Cd37	0.00976832	0.01	95.4%
Cd38	0.2192928	0.03	13.7%
Cd3d	0.00933162	0.02	223.6%
Cd3e	0	0.00	
Cd3eap	4.98379	0.32	6.4%
Cd3g	0	0.00	
Cd4	0.012904506	0.02	127.7%
Cd40	0.2380124	0.01	5.2%
Cd40lg	0	0.00	
Cd44	1.508366	0.26	17.3%
Cd46	0.1983947	0.12	59.9%
Cd47	52.97534	3.44	6.5%
Cd48	0	0.00	
Cd5	0.00448894	0.01	137.3%
Cd52	0.01145228	0.03	223.6%
Cd53	0.04129314	0.05	130.4%
Cd55	2.29441	0.28	12.0%
Cd59a	0.8406972	0.11	12.8%
Cd59b	0.0667289	0.06	85.5%
Cd5l	0.00265788	0.01	223.6%
Cd6	0	0.00	
Cd63	6.794134	0.53	7.8%
Cd68	1.1289684	0.22	19.8%
Cd69	0	0.00	

Cd7	0.01999798	0.02	92.4%
Cd70	0.0053212	0.01	223.6%
Cd72	2.500411894	2.74	109.5%
Cd74	0.4622786	0.10	21.4%
Cd79a	0.0179429	0.01	59.1%
Cd79b	0.00358796	0.01	223.6%
Cd80	0.15971566	0.09	57.2%
Cd81	170.64	9.98	5.8%
Cd82	0.4492008	0.10	22.4%
Cd83	11.036438	1.73	15.7%
CD83	1.1287624	0.12	10.4%
Cd84	0.00473208	0.01	223.6%
Cd86	0	0.00	
Cd8a	0	0.00	
Cd8b1	0	0.00	
Cd9	3.088808	0.52	16.8%
Cd93	0.015641896	0.01	57.4%
Cd94	0	0.00	
Cd96	0.00574766	0.01	223.6%
Cd97	0.7178168	0.21	28.7%
Cd99l2	45.64644	0.88	1.9%
Cda	0.15511144	0.09	57.7%
Cdadc1	13.36846	0.64	4.8%
Cdan1	3.011528	0.18	6.0%
Cdc123	27.3865	1.53	5.6%
Cdc14a	0.522206	0.08	15.0%
Cdc14b	1.635652	0.40	24.2%
Cdc16	23.54312	1.27	5.4%
Cdc20	0.759847	0.25	33.0%
Cdc23	14.09372	0.48	3.4%
Cdc25a	3.32225	0.11	3.3%
Cdc25b	2.163758	0.24	11.0%
Cdc25c	0.2796024	0.08	28.7%
Cdc26	6.737764	1.31	19.4%
Cdc27	17.8317	0.55	3.1%
Cdc2a	1.1308984	0.31	27.3%
Cdc2l1	14.9884	0.43	2.8%
Cdc2l5	8.22884	0.22	2.6%
Cdc2l6	1.34306	0.18	13.1%
Cdc34	8.425234	0.83	9.8%
Cdc37	37.78104	1.41	3.7%
Cdc37l1	39.96838	2.67	6.7%
Cdc40	5.261052	0.35	6.7%
Cdc42	152.7202	8.27	5.4%
Cdc42bpa	21.17406	1.29	6.1%
Cdc42bpb	18.86418	0.59	3.1%
Cdc42bpg	1.24248	0.06	4.7%

Cdc42ep1	1.462202	0.22	14.9%
Cdc42ep2	13.68394	1.35	9.9%
Cdc42ep3	4.053826	0.33	8.2%
Cdc42ep4	5.30572	0.45	8.4%
Cdc42ep5	0.7498402	0.36	47.9%
Cdc42se1	10.216856	0.43	4.2%
Cdc42se2	50.09008	5.14	10.3%
Cdc45l	0.8368972	0.07	8.3%
Cdc5l	6.704454	0.40	5.9%
Cdc6	0.15714826	0.05	32.4%
cdc6	0.04197468	0.04	95.2%
Cdc7	4.77205	0.59	12.3%
Cdc73	4.154692	0.37	8.8%
Cdca2	0.424442	0.07	16.8%
Cdca3	1.403448	0.36	25.4%
Cdca4	2.299556	0.33	14.2%
Cdca5	0.5275136	0.10	19.0%
Cdca7	0.574185	0.09	15.5%
Cdca7l	0.3588648	0.13	36.6%
Cdca8	1.0978404	0.16	14.2%
Cdcp1	0.06701666	0.02	35.1%
Cdcp2	0.00422712	0.01	137.8%
Cdgap	1.19874	0.15	12.5%
Cdh1	0.344669	0.03	8.7%
Cdh10	17.77826	0.68	3.8%
Cdh11	18.00414	1.38	7.6%
Cdh12	1.845484	0.19	10.4%
Cdh13	75.55284	2.25	3.0%
Cdh15	0.0029839	0.01	223.6%
Cdh16	0	0.00	
Cdh17	0.010030982	0.02	173.9%
Cdh18	22.79866	0.60	2.6%
Cdh19	0.04621976	0.02	37.5%
Cdh2	24.54784	0.47	1.9%
Cdh20	1.792204	0.11	6.2%
Cdh22	5.440262	0.97	17.8%
Cdh23	0.09603998	0.03	29.4%
Cdh24	11.86374	1.51	12.7%
Cdh26	0.03554232	0.02	58.2%
Cdh3	0.035998006	0.03	71.9%
Cdh4	7.104596	0.46	6.5%
Cdh5	0.8602956	0.23	27.3%
Cdh6	6.58416	0.37	5.7%
Cdh7	7.003076	0.20	2.9%
Cdh8	11.7094	1.00	8.5%
Cdipt	54.68102	1.86	3.4%
Cdk10	9.356328	0.69	7.4%

Cdk2	1.355682	0.30	22.4%
Cdk2ap2	11.62362	0.51	4.4%
Cdk3	0.4224378	0.15	34.4%
Cdk4	22.00158	1.73	7.8%
Cdk5	44.81148	1.27	2.8%
Cdk5r1	37.16284	1.78	4.8%
Cdk5r2	71.18472	8.67	12.2%
Cdk5rap1	3.595342	0.22	6.0%
Cdk5rap2	1.414828	0.13	9.0%
Cdk5rap3	10.87102	0.77	7.1%
Cdk6	0.1769154	0.07	38.5%
Cdk7	7.602736	0.61	8.1%
Cdk8	7.48564	0.27	3.6%
Cdk9	15.13948	0.66	4.4%
Cdkal1	3.74448	0.18	4.8%
Cdkl1	0.7407778	0.15	20.7%
Cdkl2	22.74612	0.67	3.0%
Cdkl3	7.961198	0.53	6.6%
Cdkl4	0.5370826	0.10	18.0%
Cdkl5	5.212904	0.42	8.0%
Cdkn1a	22.69896	3.96	17.5%
Cdkn1b	13.90044	0.31	2.2%
Cdkn1c	9.584512	2.57	26.8%
Cdkn2a	5.225492	0.81	15.6%
Cdkn2aip	4.063534	0.16	3.9%
Cdkn2aipnl	19.62964	1.51	7.7%
Cdkn2b	2.394226	0.22	9.0%
Cdkn2c	1.0542838	0.27	25.9%
Cdkn2d	0.02063572	0.01	59.1%
Cdkn3	0.5059208	0.15	29.2%
Cdo1	12.42346	1.61	13.0%
Cdon	1.686698	0.16	9.3%
Cdr2	6.69411	0.54	8.0%
Cdr2l	9.898644	0.56	5.7%
Cdrt4	0.07990346	0.04	51.7%
Cds1	15.74884	0.97	6.1%
Cds2	69.86156	4.02	5.8%
Cdsn	0.06801544	0.02	32.7%
Cdt1	2.297088	0.30	13.2%
Cdv1R	3.488658	0.92	26.3%
Cdv3	17.94236	1.35	7.5%
Cdx1	0.0033657	0.01	223.6%
Cdx2	0.00348992	0.01	223.6%
Cdx4	0.05179206	0.02	29.5%
Cdyl	1.628376	0.16	9.9%
Cdyl2	1.872922	0.18	9.4%
Ceacam1	0.00821218	0.01	102.3%

Ceacam10	0.00700198	0.02	223.6%
Ceacam11	0	0.00	
Ceacam12	0	0.00	
Ceacam13	0.0062946	0.01	223.6%
Ceacam14	0	0.00	
Ceacam15	0.00570982	0.01	223.6%
Ceacam16	0.2306074	0.06	27.2%
Ceacam18	0.00586398	0.01	223.6%
Ceacam19	0	0.00	
Ceacam2	0.00256724	0.01	223.6%
Ceacam20	0.3937254	0.10	24.5%
Ceacam9	0.03047452	0.01	44.9%
Cebpa	0.898689	0.11	12.7%
Cebpb	1.773516	0.25	14.3%
Cebpd	1.14446	0.31	26.8%
Cebpe	0.01191298	0.02	141.9%
Cebpg	7.489744	0.26	3.5%
Cebpz	6.060364	0.50	8.2%
Cecr2	0.255053	0.06	24.2%
Cecr5	3.22918	0.28	8.6%
Cecr6	2.372342	0.13	5.4%
Ceecam1	3.178182	0.28	8.9%
Cel	0.00244292	0.01	223.6%
Celf3	1.846326	0.54	29.1%
Celsr1	0.2028722	0.02	11.1%
Celsr2	15.39932	0.42	2.7%
Celsr3	10.016466	0.31	3.1%
Cend1	152.261	7.36	4.8%
Cenpa	0.369398	0.11	29.6%
Cenp-a	0.5864814	0.20	34.4%
Cenpb	18.20808	0.79	4.3%
Cenpc1	7.424266	1.02	13.8%
Cenpe	0.4150622	0.03	7.6%
Cenpf	0.4438786	0.13	28.7%
Cenph	0.864267	0.20	23.3%
Cenpi	0.8101572	0.13	16.7%
Cenpj	0.7115416	0.06	8.0%
Cenpk	0.4032356	0.18	44.9%
Cenpl	0.9607034	0.07	6.8%
Cenpm	1.1331508	0.15	13.7%
Cenpn	1.118474	0.20	17.7%
Cenpo	3.39793	0.29	8.4%
Cenpp	0.67135	0.17	25.1%
Cenpq	2.380286	0.35	14.8%
Cenpt	6.312588	1.12	17.7%
Centa1	62.50966	3.77	6.0%
Centa2	0.05404038	0.02	28.3%

Centb1	0.00502874	0.01	138.8%
Centb2	7.166128	0.48	6.8%
Centb5	18.35924	1.16	6.3%
Centd1	0.454192	0.21	45.8%
Centd2	4.074966	0.95	23.2%
Centd3	0.03308294	0.01	23.0%
Centg1	29.76658	1.94	6.5%
Centg2	29.85246	1.20	4.0%
Centg3	80.83812	4.51	5.6%
Cep110	1.39701	0.52	37.6%
Cep135	1.253216	0.19	14.9%
Cep152	0.2282068	0.03	11.2%
Cep164	3.000978	0.31	10.3%
Cep170	26.06862	1.51	5.8%
Cep192	1.434318	0.24	16.5%
Cep2	0.855692	0.40	46.4%
Cep250	2.814516	0.33	11.7%
Cep27	6.250302	0.56	9.0%
Cep290	0.2143466	0.13	60.3%
Cep350	2.33841	0.19	7.9%
Cep55	0.4095106	0.07	16.3%
Cep57	5.030194	0.34	6.9%
Cep63	4.428996	0.19	4.4%
Cep68	8.837642	1.01	11.5%
Cep70	6.22422	0.50	8.0%
Cep72	0.7329038	0.17	23.1%
Cep76	1.429124	0.10	6.9%
Cep78	4.231094	0.19	4.4%
Cept1	15.09614	0.79	5.3%
Cer1	0	0.00	
Cerk	32.40574	1.00	3.1%
Cerkl	0.028523924	0.03	107.2%
Ces1	0.00483636	0.01	223.6%
Ces2	0	0.00	
Ces3	0.02805584	0.01	39.1%
Ces5	0.02838284	0.03	98.1%
Ces6	0	0.00	
Ces7	0.04473654	0.03	72.2%
Cetn1	0.00456584	0.01	223.6%
Cetn2	10.720736	0.69	6.4%
Cetn3	25.03352	1.64	6.6%
Cetn4	0.7651896	0.20	26.2%
Cfb	0.14886896	0.04	29.6%
Cfc1	0.00671654	0.02	223.6%
Cfd	0	0.00	
Cfdp1	46.9331	1.55	3.3%
Cfh	0.3645456	0.08	21.0%

Cfhr1	0	0.00	
Cfhrb_4/2	0	0.00	
Cfi	0.0098542	0.01	142.3%
Cfl1	227.7232	16.81	7.4%
Cfl2	75.59394	4.88	6.5%
Cflar	1.617092	0.19	12.0%
Cfp	1.803762	0.42	23.1%
Cftr	0.2021452	0.03	16.1%
CFTR	0.07966938	0.07	89.0%
Cga	0.026697	0.06	223.6%
Cggbp1	25.70124	1.17	4.5%
Cgn	4.392212	0.11	2.5%
Cgnl1	2.887852	0.36	12.5%
Cgref1	14.23446	0.82	5.8%
Cgrrf1	6.917126	0.55	8.0%
Ch25h	0.2722148	0.06	22.9%
CHAC	2.147266	0.25	11.6%
Chac1	14.73296	1.83	12.4%
Chac2	4.529574	1.24	27.3%
Chad	0.01474228	0.02	138.0%
Chaf1a	1.698376	0.08	5.0%
Chaf1b	1.411974	0.15	10.8%
ChaK	0.3052362	0.10	33.4%
chat	2.5321	0.41	16.1%
Chat	0.07472374	0.08	110.1%
Chchd1	20.81338	1.89	9.1%
Chchd2	36.61874	1.34	3.7%
Chchd3	24.85364	0.97	3.9%
Chchd4	17.66446	1.34	7.6%
Chchd5	5.099466	0.51	10.1%
Chchd6	33.50736	2.25	6.7%
Chchd7	11.21794	1.01	9.0%
Chchd8	3.937716	0.62	15.8%
Chd1	2.826914	0.20	6.9%
Chd1l	0.9358482	0.09	9.2%
Chd2	6.497872	0.55	8.5%
Chd3	63.15762	1.38	2.2%
Chd4	14.54968	1.21	8.3%
Chd5	21.29618	0.61	2.9%
Chd6	11.9155	0.46	3.9%
Chd7	1.549746	0.10	6.3%
Chd8	15.67326	0.57	3.6%
Chd9	5.063534	0.54	10.6%
Chdh	0.020705254	0.02	74.0%
Chek1	0.7328234	0.08	11.4%
Chek2	0.7637924	0.12	16.2%
chemokine_LARC	0.04277508	0.03	73.6%

Cherp	12.06252	1.48	12.3%
Ches1	0.12381574	0.07	56.8%
Chfr	10.72906	0.59	5.5%
Chga	118.116	5.74	4.9%
Chgb	79.72786	1.23	1.5%
Chi3l1	0.282842	0.08	27.1%
Chi3l3	0.03275856	0.02	68.4%
Chi3l4	0	0.00	
Chia	0.01549034	0.02	159.0%
Chic1	5.604378	0.85	15.2%
Chic2	5.908046	0.23	3.9%
Chid1	11.24074	1.32	11.8%
Chit1	0.12470624	0.05	38.8%
Chk2	0.1614094	0.02	14.0%
Chka	23.29506	2.75	11.8%
Chkb	12.44238	0.36	2.9%
Chl1	59.81726	3.80	6.4%
Chm	11.90622	0.42	3.5%
Chml	6.242602	0.58	9.3%
Chmp1a	29.1372	0.90	3.1%
Chmp1b	4.891616	0.33	6.7%
Chmp2a	21.57082	1.46	6.8%
Chmp2b	44.04904	1.65	3.7%
Chmp4b	59.4758	3.10	5.2%
Chmp4c	0.03249224	0.02	50.8%
Chmp5	38.40422	1.84	4.8%
Chmp6	11.60542	1.28	11.1%
Chmp7	25.69862	0.69	2.7%
Chn1	32.48596	2.19	6.7%
Chn2	3.74497	0.12	3.3%
Chodl	2.796456	0.43	15.4%
Chordc1	25.40038	1.22	4.8%
Chpt1	12.5161	1.47	11.7%
Chrac1	6.960602	0.95	13.6%
Chrd	1.1297382	0.57	50.2%
Chrdl1	0.7514518	0.12	15.8%
Chrdl2	0.04723996	0.01	30.2%
Chrm1	0.0064537	0.01	118.3%
Chrm2	18.50262	1.15	6.2%
Chrm3	8.73957	0.39	4.5%
Chrm4	2.234862	0.40	17.8%
Chrm5	0.06815074	0.03	39.0%
Chrna1	0.0070755	0.01	103.1%
Chrna10	0.000107139	0.00	223.6%
Chrna2	0.02744146	0.01	20.9%
Chrna3	0.275886	0.14	51.8%
Chrna4	20.67094	1.41	6.8%

Chrna5	0.3836258	0.14	36.5%
Chrna6	0.01037758	0.01	59.4%
Chrna7	8.656464	0.76	8.8%
Chrna9	0	0.00	
Chrnb1	0.4118318	0.12	28.8%
Chrnb2	20.07258	0.54	2.7%
Chrnb3	0.005663962	0.01	92.9%
Chrnb4	0.161446	0.09	54.1%
Chrnd	0	0.00	
Chrne	0	0.00	
Chrng	0	0.00	
Chrp	13.00974	0.49	3.8%
Chst1	11.9196	0.79	6.6%
Chst10	14.3983	0.70	4.9%
Chst11	7.65821	0.63	8.3%
Chst12	8.02446	0.69	8.6%
Chst2	23.88628	1.11	4.6%
Chst3	0.06665986	0.02	24.1%
Chst4	0.03991026	0.04	111.3%
Chst5	0.02314432	0.02	106.5%
Chst7	0.8792562	0.14	16.0%
Chst8	1.72503	0.36	20.6%
Chst9	0.7752564	0.13	16.8%
Chsy1	2.909172	0.09	3.1%
Chtf18	0.3295832	0.01	2.2%
Chuk	8.358946	0.31	3.7%
Churc1	23.22642	3.31	14.3%
Ci1	0.09346976	0.09	94.7%
Ciao1	13.10442	0.35	2.7%
Ciapin1	23.02316	2.04	8.9%
Cib1	3.674544	0.22	6.0%
Cib2	12.58816	0.34	2.7%
Cib3	0	0.00	
Cic	29.44922	1.33	4.5%
Cidea	0.3377974	0.11	33.7%
Cideb	0.3270414	0.13	41.1%
Cidec	0	0.00	
Ciita	0.01595106	0.01	59.6%
Cilp	1.0066924	0.15	14.9%
Cilp2	0.4038354	0.04	8.7%
cip7	1.982308	0.62	31.0%
Cip85	2.111652	0.56	26.4%
Cirbp	67.60346	10.27	15.2%
Cirh1a	7.72575	0.69	8.9%
Cis4	0.1055549	0.05	50.2%
Cisd1	64.98554	3.15	4.9%
Cisd2	9.43567	0.35	3.7%

Cish	1.33919	0.17	12.5%
Cit	3.510408	0.59	16.9%
Cited1	6.681028	1.46	21.9%
Cited2	22.41512	0.91	4.0%
Cited4	0.3825054	0.12	32.6%
Ciz1	8.289928	0.60	7.2%
Ckap2	2.328148	0.46	19.8%
Ckap2l	0.7707998	0.15	19.9%
Ckap4	19.68894	1.65	8.4%
Ckap5	26.96104	1.57	5.8%
Ckb	147.6438	12.55	8.5%
Cklf	1.34053	0.18	13.7%
CKLF6	0.2373278	0.10	44.2%
Cklfsf2a	0.00703632	0.01	141.9%
Ckm	0.02913538	0.02	71.1%
Ckmt1	123.5294	5.13	4.1%
Ckmt2	0	0.00	
Cks1b	2.286306	0.58	25.3%
Cks2	0.4172642	0.17	40.6%
Ckt2	0.01012076	0.01	137.3%
Clasp1	30.88804	1.38	4.5%
Clasp2	94.54906	1.53	1.6%
ClaspL	11.58466	0.56	4.8%
Clast2	0.5329986	0.31	58.1%
Clast4	0.1397694	0.03	22.4%
Clca1	0.03612208	0.01	37.7%
Clca2	0.0089061	0.01	102.2%
Clca3	0.013342424	0.01	107.4%
Clca4	0.00723628	0.01	93.3%
Clca5	0.00544966	0.01	138.1%
Clca6	0.00555476	0.01	223.6%
Clcc1	5.491268	0.49	8.9%
Clcf1	1.6717036	0.67	40.1%
Clcn1	0.1915332	0.04	22.1%
Clcn2	7.257858	0.18	2.5%
Clcn3	52.63078	3.10	5.9%
CLCN3	0.638683	0.17	26.4%
Clcn4-2	48.52664	1.22	2.5%
Clcn5	17.83006	1.24	6.9%
Clcn6	31.50824	1.43	4.5%
Clcn7	14.04322	0.54	3.8%
Clcnka	0	0.00	
Clcnkb	0.01282394	0.01	112.5%
Cldn1	5.206062	1.08	20.7%
Cldn10	1.0726112	0.12	10.8%
Cldn11	0.287254	0.17	58.7%
Cldn12	7.79274	0.38	4.8%

Cldn13	0	0.00	
Cldn14	0.00358604	0.01	223.6%
Cldn15	0.06675274	0.03	41.3%
Cldn16	0	0.00	
Cldn17	0	0.00	
Cldn18	0.09998548	0.07	71.5%
Cldn19	0.03885744	0.02	55.1%
Cldn2	0.06855704	0.02	30.2%
Cldn23	0.1243958	0.03	27.0%
Cldn3	0.04096852	0.03	64.3%
Cldn4	0.01938234	0.02	107.0%
Cldn5	0.00419804	0.01	223.6%
Cldn6	0.1830028	0.09	47.1%
Cldn7	0.07171826	0.05	66.1%
Cldn8	0	0.00	
Cldn9	0	0.00	
Cldnd1	29.35864	2.37	8.1%
Cldnd2	0	0.00	
Clec11a	1.3060558	0.36	27.9%
Clec12a	0.01015292	0.02	152.5%
Clec14a	0.007815872	0.01	104.5%
Clec16a	12.1917	0.88	7.2%
Clec1a	0.2071432	0.07	33.5%
Clec1b	0.0138455	0.02	137.0%
Clec2d	0.4758546	0.09	17.9%
Clec2e	0	0.00	
Clec2g	0.00361528	0.01	223.6%
Clec2h	0.0063362	0.01	137.0%
Clec2i	0	0.00	
Clec3a	0.0207646	0.05	223.6%
Clec3b	4.959812	1.22	24.6%
Clec4a1	0.00401236	0.01	223.6%
Clec4a2	0.00857848	0.01	139.0%
Clec4a3	0	0.00	
Clec4a4	0	0.00	
Clec4b1	0	0.00	
Clec4b2	0	0.00	
Clec4d	0	0.00	
Clec4e	0	0.00	
Clec4f	0	0.00	
Clec4g	0	0.00	
Clec4n	0	0.00	
Clec5a	0.00527919	0.00	94.7%
Clec6a	0	0.00	
Clec7a	0.02329748	0.02	94.4%
Clec9a	0.0133189	0.02	137.0%
Clg	0.12784186	0.09	67.1%

Clgn	5.476106	0.42	7.7%
Clic1	4.805534	0.85	17.6%
Clic3	0.17773224	0.12	68.3%
Clic4	8.902578	0.49	5.5%
Clic5	0.06678194	0.03	47.7%
Clic6	0.5749522	0.14	24.7%
Clim1	0.02269122	0.00	21.7%
Clint1	17.61558	0.43	2.5%
Clip1	13.3648	0.88	6.6%
Clip2	19.3075	0.42	2.2%
Clip3	136.5646	5.59	4.1%
Clip4	14.20042	0.73	5.1%
Clip50	0.7633334	0.40	51.9%
Clk1	23.90372	3.40	14.2%
clk-1	0.1305943	0.03	21.8%
Clk2	8.25528	0.54	6.6%
Clk3	15.18474	0.52	3.4%
Clk4	14.35612	1.44	10.0%
CLM1-A	1.7505946	0.90	51.5%
Clm3	0	0.00	
Clmn	0.9893308	0.17	17.0%
Cln3	3.40284	0.36	10.6%
Cln5	4.47662	0.49	10.9%
Cln6	6.691046	0.22	3.3%
Cln8	4.056642	0.17	4.3%
Clnk	0.05929704	0.02	28.4%
Clns1a	0.0331876	0.03	89.0%
Clock	9.920948	0.68	6.9%
Clp1	2.249342	0.21	9.5%
Clpb	12.9521	0.69	5.3%
Clpp	22.10456	2.30	10.4%
Clps	0.2122768	0.07	32.3%
Clptm1	51.43216	2.10	4.1%
Clptm1l	41.41798	2.24	5.4%
Clpx	9.984208	0.57	5.7%
Clrn1	0.01107918	0.02	136.9%
Clrn3	0	0.00	
Clspn	0.3756372	0.09	23.1%
Clstn1	170.7234	3.52	2.1%
Clstn2	13.59144	1.18	8.7%
Clstn3	98.33866	4.18	4.2%
Clta	110.5168	6.11	5.5%
Cltb	82.91766	4.43	5.3%
Cltc	159.5734	4.54	2.8%
Clu	21.71322	3.30	15.2%
Cluap1	8.564788	0.61	7.2%
Clybl	7.103908	0.46	6.5%

Cma1	0	0.00	
Cma2	0	0.00	
Cmah	0.00582102	0.01	138.4%
CMAH	0.000509782	0.00	223.6%
Cmas	54.73246	2.80	5.1%
Cmbl	0.05724262	0.04	70.5%
Cmklr1	0.0629865	0.01	18.9%
Cml1	2.337634	0.22	9.4%
Cml2	0.040791696	0.03	85.2%
cml3	0.00331018	0.01	223.6%
Cml3	0	0.00	
Cml4	0	0.00	
Cml5	0	0.00	
Cmpk	63.3237	3.08	4.9%
Cmtm2a	0	0.00	
Cmtm2b	0	0.00	
Cmtm3	4.457048	0.87	19.5%
Cmtm4	7.681374	0.36	4.7%
Cmtm5	3.27525	0.34	10.3%
Cmtm6	3.704748	0.38	10.3%
Cmtm7	1.1421596	0.20	17.7%
Cmtm8	0.3751302	0.08	20.9%
Cmya5	0.014116156	0.01	42.8%
CN716893	0	0.00	
Cnbp	88.84482	1.15	1.3%
Cnbp2	0	0.00	
Cndp1	0.0021729	0.00	223.6%
Cndp2	22.21724	1.78	8.0%
Cnfn	0	0.00	
Cnga1	0.00537594	0.01	140.5%
Cnga2	0.0284033	0.01	48.9%
Cnga3	0.00744504	0.01	154.0%
Cnga4	0.16701272	0.08	50.0%
Cngb1b	0.035605846	0.02	64.9%
Cngb3	0.00168418	0.00	223.6%
Cnih	44.12112	3.54	8.0%
Cnih2	57.83998	7.39	12.8%
Cnih3	5.369654	0.39	7.3%
Cnih4	6.778868	0.74	10.9%
Cnksr1	0.03446036	0.02	57.2%
Cnksr2	3.6892	0.52	14.0%
Cnksr3	0.4922144	0.06	11.6%
Cnn1	0.3407874	0.08	22.6%
Cnn2	6.061286	0.57	9.4%
Cnn3	15.47474	1.45	9.3%
Cnnm1	18.1269	2.44	13.4%
Cnnm2	17.24904	0.72	4.2%

Cnnm3	5.244934	0.31	5.9%
Cnnm4	2.5737	0.15	5.7%
Cno	4.49684	0.35	7.9%
Cnot1	28.27718	2.13	7.5%
Cnot10	15.05176	0.33	2.2%
Cnot2	13.94802	0.46	3.3%
Cnot3	5.03778	0.81	16.0%
Cnot4	11.37236	0.44	3.9%
Cnot6	8.969312	0.92	10.3%
Cnot6l	9.675232	0.54	5.6%
Cnot7	39.18824	1.26	3.2%
Cnot8	11.36184	0.63	5.6%
Cnp	6.264134	0.66	10.5%
Cnpy1	0.03778632	0.02	56.9%
Cnr1	32.06016	2.65	8.3%
Cnr2	0.04427086	0.03	67.7%
CNR3	0.19819998	0.09	47.1%
Cnt3	0	0.00	
Cntd1	0.346718	0.11	30.9%
Cntf	0.690691	0.22	32.0%
Cntfr	22.61404	2.00	8.8%
Cntn1	138.7748	3.87	2.8%
Cntn2	9.052066	0.37	4.1%
Cntn3	2.760082	0.09	3.2%
Cntn4	12.34568	0.90	7.3%
Cntn5	4.523126	0.37	8.1%
Cntn6	7.510142	0.45	6.0%
Cntnap1	73.47142	6.38	8.7%
Cntnap2	25.50548	1.47	5.8%
Cntnap3	1.719974	0.13	7.6%
Cntnap4	4.91125	0.23	4.7%
Cntnap5a	6.67592	0.83	12.4%
Cntnap5c	0.8507338	0.13	15.1%
Cntrob	2.463154	0.15	6.3%
Coasy	11.4076	0.19	1.7%
Cobl	7.709274	0.55	7.1%
Cobll1	0.8868428	0.15	17.3%
Coch	4.3139	0.44	10.1%
Cog1	9.155604	0.92	10.1%
Cog2	6.43757	0.21	3.3%
Cog3	7.296006	0.31	4.2%
Cog4	19.52576	1.09	5.6%
Cog5	8.432596	0.51	6.1%
Cog6	9.372712	0.26	2.8%
Cog7	13.51616	0.33	2.5%
Cog8	11.55262	0.57	5.0%
Coil	7.553248	0.48	6.4%

Col10a1	0.1988652	0.05	23.2%
Col11a1	2.791804	0.35	12.4%
Col11a2	1.221366	0.14	11.1%
Col12a1	1.4133462	0.30	21.2%
Col13a1	0.04296872	0.03	75.6%
Col14a1	4.265196	0.55	12.9%
Col15a1	9.27285	2.21	23.8%
Col16a1	1.3717762	0.28	20.7%
Col17a1	0.02236975	0.01	45.8%
Col18a1	1.80943	0.45	24.8%
Col19a1	1.348852	0.12	9.2%
Col1a1	19.762952	10.68	54.1%
Col1a2	31.49716	8.19	26.0%
Col20a1	0.2755104	0.09	31.2%
Col22a1	0.6712188	0.10	15.6%
Col23a1	1.888486	0.27	14.5%
Col24a1	0.2222976	0.06	25.5%
Col25a1	6.252476	0.81	13.0%
Col27a1	0.5579612	0.05	9.1%
Col28a1	0.2315208	0.05	22.9%
Col2a1	0.3056368	0.04	11.6%
Col3a1	106.78096	26.55	24.9%
Col4a1	46.76358	7.45	15.9%
Col4a2	33.19546	4.52	13.6%
Col4a3	0.012754232	0.01	49.0%
Col4a3bp	16.79062	1.11	6.6%
Col4a4	0.011012142	0.00	37.3%
Col4a5	2.61228	0.35	13.3%
Col4a6	0.331978	0.06	18.9%
Col5a1	8.628138	2.97	34.4%
Col5a2	5.694028	1.13	19.8%
Col5a3	0.187837	0.03	16.7%
Col6a1	24.76046	6.92	28.0%
Col6a2	26.70632	7.70	28.8%
Col6a3	14.443432	3.34	23.1%
Col6a5	0.0940566	0.03	30.1%
Col7a1	0.3836506	0.09	22.5%
Col8a1	0.2996182	0.10	34.8%
Col8a2	0.6382344	0.18	28.2%
Col9a1	0.05177236	0.03	54.2%
Col9a2	0.12374438	0.04	35.8%
Col9a3	0.7443022	0.11	14.5%
Colec10	0.005398464	0.01	150.1%
Colec11	0.01638762	0.01	58.7%
Colec12	3.581022	0.74	20.7%
Colq	0.02165934	0.02	86.8%
Commd1	13.30082	0.87	6.5%

Commd10	9.185006	1.45	15.8%
Commd2	3.730992	0.27	7.3%
Commd3	35.84852	1.35	3.8%
Commd4	20.69864	1.31	6.3%
Commd5	10.614574	0.74	7.0%
Commd6	12.74224	0.62	4.9%
Commd7	12.39688	0.48	3.9%
Commd8	10.62196	0.38	3.6%
Commd9	12.34458	0.41	3.4%
Comp	0.04981946	0.03	51.7%
Comt	12.59038	0.41	3.3%
Comtd1	3.716102	0.34	9.1%
Copa	60.74572	2.61	4.3%
Copb1	29.96946	1.40	4.7%
Copb2	40.88728	2.00	4.9%
Cope	36.85588	1.71	4.7%
Cope1	2.31793	0.47	20.4%
Copg	107.002	3.09	2.9%
Copg2	30.41188	1.48	4.9%
Cops2	44.71372	1.03	2.3%
COPS2	0.1807118	0.03	19.3%
Cops3	35.869	1.59	4.4%
Cops4	40.53594	1.29	3.2%
Cops5	55.37506	4.22	7.6%
Cops6	51.70292	2.38	4.6%
COPS6	4.525734	0.77	17.1%
Cops7a	35.0981	0.95	2.7%
Cops7b	9.814176	0.89	9.1%
COPS7b	0.8630686	0.43	50.4%
Cops8	31.11754	2.34	7.5%
Copz1	55.2752	4.05	7.3%
Copz2	2.20822	0.43	19.3%
Coq10a	12.46452	0.53	4.3%
Coq10b	9.364774	0.73	7.8%
Coq2	12.73474	0.92	7.2%
Coq3	8.445722	0.40	4.8%
Coq4	6.95637	1.53	22.0%
Coq5	16.57496	0.56	3.4%
Coq6	9.64344	0.45	4.7%
Coq7	12.91704	2.12	16.4%
Coq9	18.64914	0.68	3.7%
Corin	0.04350093	0.03	59.2%
Corl2	0.3223586	0.08	25.2%
Coro1a	15.31504	0.63	4.1%
Coro1b	26.36758	1.11	4.2%
Coro1c	31.99726	3.52	11.0%
Coro2a	6.978574	3.91	56.0%

Coro2b	16.2045	0.87	5.4%
Coro6	33.28736	7.01	21.1%
Coro7	14.59534	0.67	4.6%
Cort	0.12787212	0.08	60.3%
cot	0.02631974	0.02	60.8%
Cotl1	23.76748	2.72	11.4%
Cox10	9.570064	0.57	5.9%
Cox11	3.335708	0.41	12.4%
Cox15	19.89188	1.19	6.0%
Cox17	24.8286	1.65	6.7%
Cox18	14.00694	0.98	7.0%
Cox19	16.59616	1.69	10.2%
Cox2	215.3946	27.58	12.8%
COX4AL	0.742803	0.19	25.4%
Cox4i1	317.967	13.54	4.3%
Cox4i2	0.0725905	0.04	55.3%
Cox4nb	13.33064	0.20	1.5%
Cox5a	129.3154	8.19	6.3%
Cox6a1	224.6466	20.82	9.3%
Cox6a2	0.7784622	0.30	38.9%
Cox6b1	195.0996	9.93	5.1%
Cox6b2	10.639924	0.92	8.6%
Cox6c	70.52384	4.44	6.3%
Cox7a1	1.2223692	0.41	33.9%
Cox7a2	98.87094	3.72	3.8%
Cox7a2l	49.96876	4.12	8.2%
Cox7b	57.76354	2.97	5.1%
Cox7b2	0.12544388	0.05	42.7%
Cox7c	12.78804	0.85	6.6%
Cox8a	335.6526	17.74	5.3%
Cox8b	0.11544976	0.05	39.6%
Cox8c	0	0.00	
Cp	2.063568	0.47	22.6%
Cpa1	0.01080164	0.02	142.2%
Cpa2	0.09415136	0.03	36.0%
Cpa3	0	0.00	
Cpa4	0.11335756	0.03	30.5%
Cpa5	0.01215586	0.01	97.3%
Cpa6	0.05111172	0.04	75.0%
Cpb1	0.03229358	0.04	135.1%
Cpb2	0.01160552	0.02	145.6%
Cpd	6.240364	0.50	8.0%
Cpe	348.2086	7.87	2.3%
Cpeb1	12.5995	1.71	13.6%
Cpeb2	23.64516	0.38	1.6%
cpeb2	0.11448298	0.06	55.1%
Cpeb3	21.78288	1.33	6.1%

Cpeb4	27.25066	1.69	6.2%
Cphx	0	0.00	
Cplx1	327.2848	12.50	3.8%
Cplx2	62.60988	1.84	2.9%
Cplx3	0.00979023	0.01	59.9%
Cplx4	0.00385254	0.01	223.6%
Cpm	0.4262444	0.08	19.2%
Cpn1	0.03590686	0.04	105.8%
Cpn2	0	0.00	
Cpne1	18.98218	0.46	2.4%
Cpne2	1.320144	0.28	21.1%
Cpne3	3.3102	0.36	11.0%
Cpne4	6.219148	0.21	3.4%
Cpne5	3.120414	0.71	22.7%
Cpne6	0.5877376	0.06	9.4%
Cpne7	0.6697442	0.11	16.3%
Cpne8	3.476492	0.30	8.6%
Cpne9	0.18161556	0.16	86.8%
Cpox	6.354324	0.35	5.5%
Cps1	0	0.00	
Cpsf1	14.71578	0.58	3.9%
Cpsf2	13.06826	0.92	7.0%
Cpsf3	13.8377	0.54	3.9%
Cpsf3l	9.010594	0.74	8.2%
Cpsf4	7.974102	0.17	2.2%
Cpsf6	24.88422	0.84	3.4%
Cpt1a	3.117906	0.47	15.1%
Cpt1b	0.6536732	0.17	26.0%
Cpt1c	22.56048	0.48	2.1%
Cpt2	2.583206	0.17	6.7%
Cpvl	0.01264516	0.02	147.5%
Cpxcr1	0	0.00	
Cpxm1	4.091794	0.16	4.0%
Cpxm2	0.12736372	0.03	25.6%
Cpz	0.2983542	0.16	53.0%
Cr2	0.04317214	0.03	58.6%
Crabp1	42.77618	12.58	29.4%
Crabp2	0.08683202	0.04	45.2%
Cradd	2.06105	0.11	5.1%
Cramp1l	3.619112	0.22	6.2%
Crat	5.663862	0.20	3.5%
Crb1	1.855034	0.13	7.1%
Crb2	0.1761882	0.03	15.5%
Crb3	0.3419024	0.15	44.7%
Crbn	30.8026	2.72	8.8%
Crcp	15.04204	0.53	3.5%
Crct1	0.02319768	0.02	91.9%

Creb1	6.064502	0.22	3.6%
Creb3	18.15598	1.33	7.3%
Creb3l1	4.710048	0.67	14.1%
Creb3l2	5.352088	0.29	5.3%
Creb3l3	0.0229266	0.02	82.0%
Creb3l4	0.10764916	0.08	69.7%
Creb5	0.12959504	0.06	47.1%
Crebbp	6.27993	0.15	2.4%
Crebl1	19.85678	1.81	9.1%
Crebl2	11.007906	2.51	22.8%
Crebzf	7.15508	0.54	7.5%
Creg1	15.61212	0.77	4.9%
Creg2	16.76286	1.51	9.0%
Creld1	39.36088	1.46	3.7%
Creld2	9.639628	0.83	8.7%
Crem	2.649762	0.30	11.3%
Crh	0.2173016	0.07	30.1%
Crhbp	0.206752	0.07	34.7%
Crhr1	4.086966	0.93	22.7%
Crhr2	0.09470882	0.03	27.0%
Crik	0.22964636	0.12	54.0%
Crim1	13.33938	0.08	0.6%
Crim2	0.2850822	0.10	33.9%
Crip1	0.5363656	0.09	17.6%
Crip2	3.988066	0.31	7.9%
Crip3	0.7403246	0.18	24.9%
Cript	25.68494	0.57	2.2%
Crisp1	0.01432064	0.02	138.6%
Crisp2	0	0.00	
Crisp3	0.00638306	0.01	223.6%
Crisp4	0	0.00	
Crispld1	1.443064	0.26	18.0%
Crispld2	0.12353164	0.05	41.5%
Crk	41.58486	1.88	4.5%
Crkl	15.57148	0.29	1.9%
Crkr	0.00108255	0.00	223.6%
Crkrs	0.5075234	0.13	25.5%
Crlf1	2.648894	0.42	15.9%
Crlf3	2.46364	0.07	2.9%
CRLM3	0.8899456	0.14	16.0%
Crls1	14.18102	0.41	2.9%
Crmp1	124.8858	17.08	13.7%
Crmp5	0.419309	0.12	29.1%
Crnkl1	3.646038	0.44	12.0%
Crnn	0	0.00	
Crocc	2.040782	0.34	16.6%
Crot	7.277368	0.51	7.0%

Crp	0.01222678	0.02	137.0%
Crry	2.965964	0.22	7.3%
CRT	1.1556872	0.24	20.4%
Crtac1	17.63998	1.23	7.0%
Crtam	0.00607234	0.01	137.3%
Crtap	4.919446	0.88	18.0%
Crtc1	14.91836	0.39	2.6%
Crtc2	7.39682	0.23	3.1%
Crtc3	2.256852	0.08	3.5%
Crtr-1	0.04441264	0.06	141.9%
Crx	0	0.00	
Crxos1	0.01258174	0.01	106.1%
Cry1	3.826564	0.14	3.6%
Cry2	16.11884	0.47	2.9%
Cryaa	0	0.00	
Cryab	0.51808662	0.32	61.1%
Cryba1	0	0.00	
Cryba2	0	0.00	
Cryba4	0.01297112	0.02	137.3%
Crybb1	0.05390092	0.04	70.3%
Crybb2	0.0075101	0.02	223.6%
Crybb3	0.07417408	0.05	63.7%
Cryga	0.09796764	0.06	58.6%
Crygb	0	0.00	
Crygc	0	0.00	
Crygd	0	0.00	
Cryge	0	0.00	
Crygf	0	0.00	
Crygn	0.0484638	0.05	109.4%
Crygs	0	0.00	
Cryl1	3.76321	0.41	10.8%
Crym	0.10563558	0.10	92.7%
Cryz	5.769002	0.31	5.4%
Cryzl1	22.59336	1.00	4.4%
Cs	116.7676	6.95	6.0%
Cs1	0	0.00	
CS444420	0	0.00	
CS444436	0	0.00	
CS444445	0	0.00	
CS444455	0	0.00	
CS444458	0	0.00	
CS444466	0	0.00	
CS444476	0	0.00	
CS444477	0	0.00	
CS444488	0	0.00	
CS444494	0	0.00	
CS444505	0	0.00	

CS444510	0	0.00	
CS447912	0	0.00	
CSA	1.4150918	0.65	45.8%
Csad	5.610012	0.64	11.5%
Csda	12.28488	1.68	13.7%
Csdc2	78.58376	4.37	5.6%
Csde1	48.91208	0.98	2.0%
Cse1l	29.43802	0.90	3.1%
Csf1	2.920214	0.34	11.6%
Csf1r	0.018342068	0.02	84.5%
Csf2	0	0.00	
Csf2ra	6.3091	0.54	8.5%
Csf2rb	0.00151488	0.00	223.6%
Csf2rb2	0.00174104	0.00	223.6%
Csf3	0.0074573	0.02	223.6%
Csf3r	0.001602304	0.00	223.6%
Csk	30.26784	2.48	8.2%
Cskn2a2	1.1549022	0.81	69.7%
Csl	29.67418	2.04	6.9%
Csmd1	3.118134	0.21	6.8%
Csmd2	1.42889	0.16	11.5%
Csmd3	3.746904	0.67	17.9%
Csn1s1	0.00671068	0.02	223.6%
Csn1s2a	0	0.00	
Csn1s2b	0	0.00	
Csn2	0	0.00	
Csn3	0.03891512	0.05	126.7%
Csnk1a1	77.80852	2.60	3.3%
Csnk1d	35.3179	1.02	2.9%
Csnk1e	37.88566	1.29	3.4%
Csnk1g1	4.59092	0.18	3.9%
Csnk1g2	33.71878	1.85	5.5%
Csnk1g3	15.05546	0.27	1.8%
Csnk2a1	20.159	1.21	6.0%
Csnk2a2	15.88008	0.63	4.0%
Csnk2b	53.36892	4.03	7.5%
Cspg2	2.74177	0.39	14.1%
Cspg4	0.3632382	0.11	30.2%
Cspg5	71.0625	6.16	8.7%
Cspp1	4.510972	0.23	5.1%
Csprs	0	0.00	
Csrp1	6.713444	0.45	6.7%
Csrp2	3.378632	0.69	20.4%
Csrp2bp	6.75419	0.47	7.0%
Csrp3	0.02087422	0.02	93.1%
Cst10	0	0.00	
Cst11	0	0.00	

Cst12	0	0.00	
Cst13	0	0.00	
Cst3	177.3008	19.36	10.9%
Cst6	0.10373178	0.01	14.2%
Cst7	0	0.00	
Cst8	0	0.00	
Cst9	0	0.00	
Csta	0	0.00	
Cstad	0.2578136	0.10	37.4%
Cstb	17.6747	1.34	7.6%
Cstf1	9.398498	0.23	2.4%
Cstf2	34.181	1.92	5.6%
Cstf2t	26.52858	1.06	4.0%
Cstf3	15.55024	1.31	8.4%
Cstl1	0.1234223	0.07	54.5%
Ctage5	9.471304	0.36	3.8%
Ctbp1	63.41494	5.03	7.9%
CtBP1	0.540617	0.12	21.6%
Ctbp2	9.589376	0.29	3.1%
CtBP2	0.0245536	0.02	62.3%
Ctbs	1.987992	0.17	8.4%
Ctcf	12.79564	0.53	4.2%
Ctcfl	0.005403744	0.01	94.0%
Ctdp1	3.424716	0.22	6.3%
Ctdsp1	4.56531	0.28	6.0%
Ctdsp2	7.704736	1.12	14.6%
Ctdspl	2.265476	0.12	5.3%
Ctdspl2	6.215296	0.37	5.9%
Ctf1	0.4518636	0.05	10.0%
Ctf2	0.02319434	0.03	150.6%
Ctgf	5.405292	0.60	11.1%
Cth	1.864904	0.33	17.7%
Cthrc1	0.3489174	0.12	33.6%
Ctla2a	0.17035148	0.09	53.8%
Ctla2b	0.00846636	0.02	223.6%
Ctla4	0.01000564	0.01	137.7%
ctla-4	0.0049727	0.01	223.6%
Ctnna1	7.859492	0.62	7.9%
Ctnna2	44.4156	1.69	3.8%
Ctnna3	0.4012408	0.10	25.9%
Ctnnal1	2.247678	0.06	2.7%
Ctnnb1	140.04	4.75	3.4%
Ctnnbip1	25.09274	2.22	8.8%
Ctnnbl1	6.553096	0.23	3.5%
Ctnnd1	9.501446	0.59	6.3%
Ctnnd2	47.8624	2.05	4.3%
Ctns	3.04724	0.78	25.6%

Ctps	18.12462	1.90	10.5%
Ctps2	11.8423	0.32	2.7%
Ctr9	12.83256	0.40	3.1%
Ctrb1	0.02259876	0.04	158.0%
Ctrc	0	0.00	
Ctrl	0.3965812	0.15	37.3%
Cts3	0	0.00	
Cts6	0	0.00	
Cts7	0	0.00	
Cts8	0	0.00	
Ctsa	13.8914	0.96	6.9%
Ctsb	77.09634	9.13	11.8%
Ctsc	1.57789	0.24	15.3%
Ctsd	150.5078	2.87	1.9%
Ctse	0.08071494	0.05	65.1%
Ctsf	30.8774	3.11	10.1%
Ctsg	0	0.00	
Ctsh	1.3751308	0.40	29.3%
Ctsj	0	0.00	
Ctsk	0.9952134	0.21	21.0%
Ctsl	64.62364	4.05	6.3%
Ctsm	0	0.00	
Ctso	3.429736	0.36	10.4%
Ctsq	0	0.00	
Ctsr	0	0.00	
Ctss	0	0.00	
Ctsw	0.03659774	0.03	83.7%
Ctsz	3.785202	0.97	25.6%
Cttn	39.91704	2.50	6.3%
Cttnbp2	6.480312	0.57	8.8%
Cttnbp2nl	6.27578	0.76	12.2%
Ctxn1	17.2933	2.82	16.3%
Cubn	0.1829754	0.04	22.8%
Cuedc1	7.536758	0.36	4.8%
Cuedc2	67.50836	3.88	5.7%
Cugbp1	15.32024	1.70	11.1%
Cugbp2	17.68154	0.55	3.1%
Cul1	33.86916	0.84	2.5%
Cul2	26.4431	1.53	5.8%
Cul3	49.00888	1.60	3.3%
Cul4a	22.85694	0.99	4.3%
Cul4b	21.18616	0.32	1.5%
Cul5	15.2673	1.28	8.4%
Cul7	22.6276	0.79	3.5%
Cuta	26.91866	1.35	5.0%
Cutc	2.02789	0.32	15.7%
Cutl1	21.66546	0.76	3.5%

Cutl2	6.778326	0.29	4.3%
Cux/CDP(1B1)	0.090995484	0.12	136.3%
Cuzd1	0.00787022	0.01	144.3%
Cwc15	24.22692	0.82	3.4%
Cwf19l1	7.113632	0.42	5.9%
Cwf19l2	3.48145	0.36	10.2%
Cx3cl1	50.16282	4.44	8.9%
Cx3cr1	0	0.00	
Cxadr	12.84098	3.05	23.7%
Cxcl1	0.09770804	0.06	61.2%
Cxcl10	0.01039328	0.02	164.4%
Cxcl11	0	0.00	
Cxcl12	3.44691	0.46	13.4%
Cxcl13	0.02250806	0.02	108.2%
Cxcl14	6.219894	0.70	11.2%
Cxcl15	0.00379468	0.01	223.6%
Cxcl16	0.04053114	0.02	52.3%
Cxcl2	0	0.00	
Cxcl3	0	0.00	
Cxcl4	0	0.00	
Cxcl5	0.2695874	0.11	39.1%
Cxcl9	0.00277124	0.01	223.6%
Cxcr3	0	0.00	
Cxcr4	1.630282	0.21	13.1%
Cxcr6	0.01177092	0.02	148.3%
Cxcr7	2.353864	0.25	10.6%
Cxx1a	6.831684	0.51	7.5%
Cxx1b	12.09442	0.75	6.2%
Cxx1c	2.031366	0.20	10.0%
Cxxc1	7.7314	0.40	5.2%
Cxxc4	3.733652	0.34	9.0%
Cxxc5	12.07926	1.10	9.1%
Cxxc6	2.724552	0.29	10.8%
Cyb5	20.35752	2.11	10.4%
Cyb561	25.42974	0.98	3.9%
Cyb561d1	1.85406	0.28	15.2%
Cyb561d2	6.108972	0.50	8.2%
Cyb5b	40.36224	2.81	7.0%
Cyb5d1	2.075376	0.35	16.9%
Cyb5d2	0.2766906	0.10	34.8%
Cyb5r1	8.440986	1.04	12.3%
Cyb5r2	0.05602042	0.07	126.5%
Cyb5r3	35.8651	3.51	9.8%
Cyb5r4	2.751152	0.33	12.0%
Cyba	0.4242192	0.15	36.1%
Cybasc3	8.55072	0.78	9.1%
Cybb	0.0020607	0.00	223.6%

Cybrd1	0.7334448	0.17	23.6%
Cyc1	139.6254	4.04	2.9%
Cycs	2.980762	0.08	2.7%
Cyct	0.01073516	0.02	223.6%
Cyfip1	7.574058	0.21	2.7%
Cyfip2	69.33814	1.32	1.9%
Cygb	24.40232	2.19	9.0%
Cyhr1	40.40112	2.30	5.7%
Cylc1	0	0.00	
Cyld	8.724706	1.08	12.3%
Cyp11a1	0.1999064	0.04	19.5%
Cyp11b1	0.00509004	0.01	137.3%
Cyp11b2	0	0.00	
Cyp17a1	0.0107049	0.01	94.7%
Cyp19a1	0.02331928	0.01	46.2%
Cyp1a1	0.024759722	0.01	53.9%
Cyp1a2	0	0.00	
Cyp1b1	3.001718	0.63	21.1%
Cyp20a1	3.981744	0.25	6.3%
Cyp21a1	0	0.00	
Cyp24a1	0.1254188	0.02	16.9%
Cyp26a1	2.735866	0.68	24.8%
Cyp26b1	2.21885	0.26	11.5%
Cyp27a1	0.0637672	0.03	51.7%
Cyp27b1	0.05950166	0.02	36.7%
Cyp2a12	0	0.00	
Cyp2a4	0	0.00	
Cyp2a5	0	0.00	
Cyp2ab1	0.00702384	0.01	144.1%
Cyp2b10	0.00420982	0.01	223.6%
Cyp2b13	0	0.00	
Cyp2b19	0	0.00	
Cyp2b23	0.00306252	0.01	223.6%
Cyp2b9	0	0.00	
Cyp2c29	0	0.00	
Cyp2c37	0	0.00	
Cyp2c38	0	0.00	
Cyp2c39	0	0.00	
Cyp2c40	0	0.00	
Cyp2c44	0.0076554	0.01	138.6%
Cyp2c50	0	0.00	
Cyp2c54	0	0.00	
Cyp2c55	0.02853134	0.04	127.4%
Cyp2c65	0.0706661	0.03	37.7%
Cyp2c66	0.01341054	0.02	142.5%
Cyp2c67	0	0.00	
Cyp2c68	0	0.00	

Cyp2c69	0	0.00	
Cyp2c70	0	0.00	
Cyp2d10	0	0.00	
Cyp2d11	0	0.00	
Cyp2d12	0	0.00	
Cyp2d13	0.00340486	0.01	223.6%
Cyp2d22	0.16866694	0.06	35.9%
Cyp2d26	0.01524116	0.02	116.8%
Cyp2d34	0	0.00	
Cyp2d40	0.02223884	0.02	102.3%
Cyp2d9	0	0.00	
Cyp2e1	0.00364998	0.01	223.6%
Cyp2f2	0.02497228	0.01	49.3%
Cyp2g1	0	0.00	
Cyp2j11	0.00300078	0.01	223.6%
Cyp2j12	0.00340714	0.01	223.6%
Cyp2j13	0.007653088	0.01	118.2%
Cyp2j5	0.0020371	0.00	223.6%
Cyp2j6	2.15362	0.38	17.7%
Cyp2j7	0	0.00	
Cyp2j8	0.03278746	0.06	186.9%
Cyp2j9	0.3168754	0.04	13.1%
Cyp2r1	0.0575993	0.04	72.1%
Cyp2s1	0.5846358	0.17	28.7%
Cyp2t4	0.2453756	0.02	7.9%
Cyp2u1	3.780408	0.29	7.8%
Cyp39a1	0.4496436	0.02	5.3%
Cyp3a11	0	0.00	
Cyp3a13	0.01942766	0.01	26.7%
Cyp3a16	0	0.00	
Cyp3a25	0	0.00	
Cyp3a41a	0	0.00	
Cyp3a44	0	0.00	
Cyp3a57	0.00573752	0.01	223.6%
Cyp46a1	31.28188	1.84	5.9%
Cyp4a10	0.00265732	0.01	223.6%
Cyp4a12a	0	0.00	
Cyp4a12b	0.00719626	0.01	137.0%
Cyp4a14	0	0.00	
Cyp4a29	0	0.00	
Cyp4a30b	0	0.00	
Cyp4a31	0	0.00	
Cyp4a32	0	0.00	
Cyp4b1	0.10510832	0.03	24.6%
Cyp4f13	1.960256	0.12	6.1%
Cyp4f14	0.1696104	0.06	36.7%
Cyp4f15	0.3144598	0.07	21.1%

Cyp4f16	1.493396	0.19	13.0%
Cyp4f18	0.0074702	0.01	141.6%
Cyp4f37	0.14292602	0.04	28.5%
Cyp4f39	0.01831014	0.01	47.5%
Cyp4v3	0.601071	0.10	16.2%
Cyp4x1	1.782246	0.22	12.1%
Cyp51	41.68494	5.59	13.4%
Cyp7a1	0	0.00	
Cyp7b1	2.95528	0.47	15.8%
Cyp8b1	0	0.00	
Cypt10	0	0.00	
Cypt2	0	0.00	
Cypt3	0.0106215	0.02	223.6%
Cypt7	0	0.00	
Cypt8	0	0.00	
Cyr61	1.2015462	0.25	20.8%
Cys1	0.6835166	0.15	21.8%
Cysltr1	0.00318546	0.01	223.6%
Cysltr2	0	0.00	
Cyt1	0.00320466	0.01	223.6%
Cyt19	0.2892648	0.18	61.6%
Cytl1	0.0028466	0.01	223.6%
Cytor4	0.03237216	0.01	17.7%
Cyyr1	0.0108818	0.01	94.8%
D030011O10Rik	10.65568	0.60	5.6%
D030016E14Rik	16.4927	0.53	3.2%
D030022P06Rik	4.477596	0.47	10.6%
D030041N04Rik	4.142268	0.27	6.6%
D030051N19Rik	13.46362	0.44	3.3%
D030056L22Rik	8.415248	0.52	6.2%
D030070L09Rik	12.41494	0.99	8.0%
D030074E01Rik	17.31054	0.50	2.9%
D0H4S114	107.12808	16.87	15.7%
D0HXS9928E	12.87938	0.88	6.8%
D10627	3.428786	0.15	4.5%
D10920	3.806848	0.32	8.5%
D10Bwg1364e	47.56086	4.14	8.7%
D10Bwg1379e	7.05364	1.06	15.0%
D10Ertd322e	20.04462	1.67	8.3%
D10Ertd610e	26.60604	1.72	6.4%
D10Ertd641e	16.58732	0.58	3.5%
D10Jhu81e	28.93738	1.00	3.5%
D10Wsu102e	9.32489	0.60	6.4%
D10Wsu52e	49.47838	1.09	2.2%
D11Bwg0434e	65.82446	5.17	7.8%
D11Bwg0517e	26.0409	2.02	7.8%
D11Ertd636e	0.04356472	0.01	33.2%

D11lgp2	0.05066164	0.05	103.9%
D11Wsu47e	2.474948	0.21	8.7%
D11Wsu99e	6.119018	0.11	1.8%
D12Ertd551e	8.994258	0.77	8.6%
D12Ertd553e	16.04862	1.51	9.4%
D12Ertd647e	3.723568	0.74	19.9%
D130029J02Rik	12.5793	1.01	8.1%
D130040H23Rik	0.7702612	0.13	17.5%
D130043K22Rik	7.168588	0.56	7.8%
D130054N24Rik	3.114836	0.20	6.4%
D130059P03Rik	3.260588	0.38	11.5%
D13Wsu177e	9.395914	0.68	7.3%
D14Abb1e	2.964268	0.26	8.7%
D14Ertd171e	20.87596	0.93	4.5%
D14Ertd436e	4.305942	0.39	9.1%
D14Ertd449e	0.2271227	0.10	44.6%
D14Ertd500e	1.518434	0.13	8.6%
D14Ertd581e	2.961156	0.68	22.9%
D14Ertd668e	0.0356744	0.03	80.2%
D15Ertd621e	20.80756	0.93	4.5%
D15Mgi27	20.26904	1.20	5.9%
D15Wsu169e	16.70606	0.81	4.9%
D15Wsu75e	28.72132	2.39	8.3%
D16605	0	0.00	
D16Ertd472e	0.4775858	0.07	13.9%
D16Ertd642e	0.03789124	0.02	52.9%
D16H22S680E	48.89466	1.84	3.8%
D17H6S53E	3.63249	0.29	8.1%
D17H6S56E-3	0.03659266	0.01	22.4%
D17H6S56E-5	1.320164	0.21	15.9%
D17Wsu104e	23.4118	2.16	9.2%
D17Wsu92e	27.06298	1.31	4.8%
D18Ertd653e	3.656438	0.36	9.9%
D19Bwg1357e	9.114298	0.93	10.2%
D19Ertd386e	2.683276	0.15	5.5%
D19Ertd652e	0.14341628	0.28	193.9%
D19Ertd721e	35.78674	3.18	8.9%
D19Ertd737e	11.42414	1.17	10.3%
D19Wsu162e	13.30292	0.83	6.2%
D1Bwg0212e	7.209778	0.13	1.8%
D1Bwg1363e	29.30378	2.30	7.9%
D1Ertd622e	13.73164	0.57	4.2%
D1Pas1	1.440016	0.03	2.2%
D230004J03Rik	17.03494	0.63	3.7%
D230007K08Rik	0.0396668	0.02	59.6%
D230010M03Rik	2.993758	0.25	8.5%
D230012E17Rik	12.81716	1.85	14.5%

D230014K01Rik	2.910698	0.24	8.2%
D230025D16Rik	8.75111	0.39	4.5%
D230037D09Rik	7.278058	0.74	10.2%
D230039L06Rik	2.55074	0.74	29.0%
D230044M03Rik	0.01402224	0.02	138.2%
D2Bwg1335e	5.854692	0.19	3.2%
D2Ertd391e	18.73392	1.60	8.5%
D2Ertd750e	0.8781286	0.08	8.8%
D2hgdh	2.03922	0.14	6.9%
D2Wsu81e	10.435168	0.57	5.5%
D330001F17Rik	1.75146	0.34	19.5%
D330012F22Rik	2.34854	0.18	7.5%
D330017J20Rik	6.663846	0.38	5.6%
D330022A01Rik	0.17239	0.10	55.8%
D330028D13Rik	1.044213	0.20	19.6%
D330037H05Rik	0.14000648	0.06	41.0%
D330038O06Rik	6.659394	0.34	5.1%
D330045A20Rik	0.02840092	0.02	84.2%
D3Bwg0562e	20.38828	2.16	10.6%
D3Ertd300e	10.072384	0.81	8.0%
D3Ertd751e	3.151834	0.34	10.8%
D3Ucla1	23.77064	0.66	2.8%
D430015B01Rik	4.156592	0.29	6.9%
D430028G21Rik	1.71257	0.03	1.9%
D430033N04Rik	0.768171	0.18	23.4%
D430039N05Rik	27.87208	1.54	5.5%
D430041B17Rik	8.734584	0.50	5.7%
D430041D05Rik	11.8303	0.27	2.3%
D430042O09Rik	2.54752	0.17	6.9%
D4Bwg0951e	6.835584	0.70	10.3%
D4Cole1e	1.296076	0.16	12.7%
D4Ertd196e	19.56222	1.38	7.1%
D4Ertd22e	6.89662	0.70	10.2%
D4Ertd429e	16.25388	1.08	6.6%
D4Wsu114e	1.437264	0.18	12.3%
D4Wsu132e	7.491404	0.43	5.7%
D4Wsu53e	76.30684	9.15	12.0%
D530005L17Rik	2.0535	0.20	10.0%
D530033C11Rik	2.048464	0.20	9.9%
D5Ertd135e	0.339765	0.11	32.1%
D5Ertd40e	2.814222	0.22	7.7%
D5Ertd577e	0	0.00	
D5Ertd579e	19.103	0.81	4.2%
D5Ertd585e	5.746198	0.22	3.8%
D5Ertd593e	0.15530702	0.05	34.6%
D5Wsu178e	18.74788	0.81	4.3%
D630002G06Rik	0.01996854	0.02	104.6%

D630002J15Rik	0	0.00	
D630003M21Rik	0.8841546	0.13	15.0%
D630004A14Rik	1.83774	0.17	9.3%
D630023B12Rik	4.012606	0.19	4.9%
D630023F18Rik	1.763458	0.24	13.9%
D630037F22Rik	2.286568	0.27	11.8%
D630039A03Rik	0.219093	0.04	18.0%
D630040G17Rik	7.06094	0.55	7.8%
D630042F21Rik	0.002382	0.01	223.6%
D630042P16Rik	0.00394748	0.01	223.6%
D630044L22Rik	0.1959448	0.02	8.7%
D630045J12Rik	3.946782	0.29	7.4%
D6Mm5e	0.0250755	0.03	100.4%
D6Wsu116e	22.09182	0.95	4.3%
D6Wsu163e	9.361612	0.31	3.3%
D6Wsu176e	41.13254	3.84	9.3%
D730001G18Rik	0.00415158	0.01	223.6%
D730039F16Rik	0.04627094	0.03	71.2%
D730040F13Rik	4.101162	0.40	9.7%
D730048I06Rik	0	0.00	
D830007B15Rik	1.248896	0.09	7.6%
D830007F02Rik	0.02772038	0.01	38.3%
D830014E11Rik	0.0353138	0.03	85.2%
D830029A09Rik	0.09273354	0.02	17.2%
D830030K20Rik	0.15710382	0.04	28.6%
D8Ertd457e	14.37812	0.82	5.7%
D8Ertd587e	4.239998	0.42	9.9%
D8Ertd738e	39.2966	4.50	11.5%
D8Ertd82e	5.903312	0.30	5.1%
D930001I22Rik	8.268888	0.53	6.5%
D930005D10Rik	3.964952	0.34	8.5%
D930014E17Rik	1.4648748	1.19	80.9%
D930015E06Rik	4.195096	0.21	5.0%
D930017K21Rik	0.01776776	0.04	223.6%
D930020B18Rik	0.036694982	0.03	90.4%
D930020E02Rik	0.0142016	0.02	106.0%
D930028F11Rik	0.7372198	0.12	16.5%
D930036F22Rik	0.222417	0.06	26.3%
D930048N14Rik	2.590354	0.22	8.6%
D9Ertd280e	0.3001706	0.02	6.4%
D9Ertd402e	5.264798	0.43	8.2%
D9Mgc48e	0.4642594	0.12	26.6%
Daam1	20.83948	1.96	9.4%
Daam2	1.0198422	0.19	18.9%
Dab1	24.32814	1.67	6.9%
Dab2	3.95076	0.65	16.5%
Dab2ip	11.5272	0.24	2.1%

Dach1	5.045966	0.32	6.4%
Dach2	2.691564	0.49	18.3%
Dact1	3.566042	0.31	8.6%
Dact2	5.36939	0.32	6.0%
Dact3	24.38906	1.46	6.0%
Dad1	62.735	3.92	6.2%
Daf2	0.0371648	0.03	68.5%
Dag1	21.28002	0.62	2.9%
Dagla	13.2739	0.78	5.8%
Daglb	6.074006	0.63	10.4%
Dak	4.266622	0.52	12.2%
Dalrd3	20.03832	0.77	3.8%
Dand5	0.089878	0.03	32.6%
Dao1	0.01377582	0.01	96.5%
Dap	6.050654	0.87	14.4%
Dap3	28.54438	0.61	2.1%
Dapk1	13.188	0.50	3.8%
Dapk2	0.16293742	0.06	39.6%
DAPK2	0.02055904	0.01	61.5%
Dapk3	14.71268	0.76	5.2%
Dapl1	0.0111318	0.02	223.6%
Dapp1	0.07298204	0.03	46.2%
Darc	22.27738	1.13	5.1%
Dars	28.69178	2.38	8.3%
Dars2	5.758544	0.31	5.4%
Daxx	6.20844	0.36	5.8%
Dazap1	16.56126	1.24	7.5%
Dazap2	31.83392	1.23	3.9%
Dazl	0.257398	0.05	20.3%
Dbc1	43.06386	2.58	6.0%
Dbf4	0.9384288	0.05	5.7%
Dbh	0.00860838	0.01	92.4%
Dbi	72.4258	2.79	3.8%
Dbil5	0.12354218	0.07	59.9%
Dbn1	43.41808	3.15	7.3%
Dbndd1	10.67902	0.39	3.6%
Dbndd2	11.33972	0.52	4.6%
Dbnl	31.97512	2.40	7.5%
Dbp	6.655494	1.64	24.6%
Dbpht2	1.706898	0.17	9.9%
Dbr1	3.3016	0.15	4.5%
Dbt	5.995916	0.10	1.7%
Dbx1	0	0.00	
Dbx2	0.4566704	0.07	15.8%
dby	0.0798447	0.01	14.0%
Dcakd	45.51004	5.91	13.0%
Dcamkl1	13.01094	1.34	10.3%

Dcamkl2	7.450502	1.82	24.5%
Dcbld1	5.555938	0.32	5.8%
Dcbld2	12.48156	1.21	9.7%
Dcc	7.876028	0.77	9.8%
Dcdc2	0.2196586	0.08	37.5%
Dcdc2a	0.02282768	0.01	23.0%
Dchs1	5.698976	0.43	7.6%
Dci	2.741644	0.25	9.2%
Dck	11.4726	0.63	5.5%
Dclk1	53.44396	7.72	14.5%
Dclk2	19.91318	1.44	7.2%
Dclk3	0.6060646	0.10	16.9%
Dclre1a	2.424946	0.10	4.1%
Dclre1b	1.389164	0.35	25.3%
Dclre1c	1.816728	0.20	11.0%
Dcn	24.7473	5.55	22.4%
Dcp1a	3.214836	0.08	2.6%
Dcp1b	2.711508	0.27	10.0%
Dcp2	6.890794	0.62	9.0%
Dcpp1	0.05131084	0.04	69.3%
Dcpp2	0.00917606	0.02	223.6%
Dcpp3	0	0.00	
Dcps	6.526948	0.80	12.3%
Dcst1	0.14803236	0.04	28.1%
Dct	0.00245736	0.01	223.6%
Dctd	0.5173682	0.08	15.2%
Dctn1	73.17372	2.41	3.3%
Dctn2	108.514	3.61	3.3%
Dctn3	49.49366	4.47	9.0%
Dctn4	45.73288	0.51	1.1%
Dctn5	28.51828	2.07	7.3%
Dctn6	32.79602	1.80	5.5%
Dcun1d1	8.198588	0.31	3.8%
Dcun1d2	10.709076	1.23	11.5%
Dcun1d3	3.040368	0.34	11.3%
Dcun1d4	65.0248	0.95	1.5%
Dcun1d5	18.35106	1.57	8.5%
Dcx	6.850248	1.45	21.2%
Dcxr	4.085712	0.27	6.7%
DD413430	0	0.00	
DD57	0.142210558	0.16	114.6%
Ddah1	18.58254	1.09	5.9%
Ddah2	8.679004	1.42	16.4%
Ddb1	102.64066	2.62	2.6%
Ddb2	3.909956	0.53	13.5%
Ddc	0.06187126	0.04	59.9%
Ddc8	0.0047385	0.01	223.6%

Ddef1	38.289	1.21	3.2%
Ddef2	12.29164	1.89	15.4%
Ddefl1	0.698565	0.11	15.9%
Ddhd1	12.22514	0.51	4.2%
Ddhd2	14.1982	0.79	5.5%
Ddi1	0	0.00	
Ddi2	7.255414	0.48	6.6%
Ddip	0.09427232	0.04	40.6%
Ddit3	32.64068	3.46	10.6%
Ddit4	23.22402	1.88	8.1%
Ddit4l	0.7721408	0.09	12.1%
DDM36	0.2383888	0.13	53.1%
Ddn	3.67071	0.25	6.7%
Ddo	0.1906094	0.04	22.4%
DDO	0.01674432	0.00	8.2%
Ddost	41.07624	3.71	9.0%
Ddp2	0.1711312	0.07	41.4%
Ddr1	8.125668	0.57	7.0%
Ddr2	2.602924	0.48	18.5%
Ddt	19.5134	1.33	6.8%
Ddx1	59.45346	2.19	3.7%
Ddx10	6.510022	0.43	6.6%
Ddx11	0.4706402	0.06	12.4%
Ddx17	139.7746	14.54	10.4%
Ddx18	0.7996656	0.10	11.9%
Ddx19a	5.61588	0.28	5.0%
Ddx19b	10.184632	0.35	3.4%
Ddx20	3.448622	0.19	5.5%
Ddx21	5.05719	0.20	3.9%
Ddx23	21.97344	0.65	2.9%
Ddx24	61.52582	1.04	1.7%
Ddx25	26.80082	1.28	4.8%
Ddx26b	9.715364	1.06	10.9%
Ddx27	10.07149	0.78	7.8%
Ddx28	5.814866	0.56	9.6%
Ddx31	1.787116	0.10	5.8%
Ddx39	7.176652	0.51	7.1%
Ddx3x	59.30226	1.14	1.9%
Ddx3y	0.06827734	0.01	19.3%
Ddx4	0.6034426	0.10	16.3%
Ddx41	8.954372	0.48	5.4%
Ddx42	21.46934	1.15	5.4%
Ddx46	10.13296	0.39	3.9%
Ddx47	18.14854	0.74	4.1%
Ddx49	7.27311	0.64	8.9%
Ddx5	157.5008	5.56	3.5%
Ddx50	9.637696	0.43	4.5%

Ddx51	2.70561	0.17	6.2%
Ddx52	5.068704	0.39	7.7%
Ddx54	9.85791	0.90	9.1%
Ddx55	4.698132	0.26	5.6%
Ddx56	11.8785	0.73	6.2%
Ddx58	0.8337222	0.06	7.7%
Ddx59	3.275038	0.17	5.2%
Ddx6	29.501	2.02	6.8%
Deadc1	3.297896	0.28	8.5%
Deaf1	42.62854	3.83	9.0%
Dear1	0.14028618	0.11	81.2%
Deb1	27.21196	0.76	2.8%
Decr1	1.464746	0.14	9.4%
Decr2	7.750026	0.26	3.4%
Dectin-1	0	0.00	
Dedd	12.02328	0.32	2.7%
Dedd2	3.47141	0.36	10.2%
Def6	0.5820946	0.08	13.7%
Def8	27.10716	0.96	3.6%
Defb1	0.045358	0.03	71.6%
Defb10	0	0.00	
Defb11	0	0.00	
Defb12	0	0.00	
Defb13	0	0.00	
Defb14	0	0.00	
Defb15	0	0.00	
Defb18	0	0.00	
Defb19	0	0.00	
Defb2	0.06581098	0.06	93.2%
Defb20	0	0.00	
Defb21	0	0.00	
Defb22	0	0.00	
Defb23	0	0.00	
Defb25	0	0.00	
Defb26	0	0.00	
Defb28	0	0.00	
Defb29	0	0.00	
Defb3	0	0.00	
Defb30	0	0.00	
Defb34	0	0.00	
Defb35	0	0.00	
Defb36	0	0.00	
Defb37	0	0.00	
Defb38	0	0.00	
Defb39	0	0.00	
Defb4	0	0.00	
Defb40	0	0.00	

Defb41	0	0.00	
Defb42	0	0.00	
Defb43	0	0.00	
Defb46-ps	0	0.00	
Defb48-ps	0	0.00	
Defb49	0.0345848	0.08	223.6%
Defb5	0	0.00	
Defb50	0	0.00	
Defb54-ps	0	0.00	
Defb6	0	0.00	
Defb7	0	0.00	
Defb8	0	0.00	
Defb9	0	0.00	
Defcr20	0	0.00	
Defcr21	0	0.00	
Defcr22	0	0.00	
Defcr23	0.01738672	0.04	223.6%
Defcr26	0	0.00	
Defcr3	0	0.00	
Defcr4	0	0.00	
Defcr5	0	0.00	
Defcr6	0.03788842	0.05	137.3%
Defcr-rs1	0.05599626	0.08	138.6%
Degs1	48.05922	1.59	3.3%
Degs2	0.12705772	0.10	79.0%
Dek	11.42636	0.50	4.4%
Del1	0.12698224	0.09	69.9%
Deltex3	4.583644	0.46	9.9%
Dennd1a	9.053816	0.29	3.2%
Dennd1b	2.17451	0.40	18.3%
Dennd1c	0.02641896	0.02	79.4%
Dennd2a	3.451766	0.23	6.8%
Dennd2c	0.4656774	0.05	11.1%
Dennd2d	0.0843154	0.06	70.0%
Dennd3	0.6937048	0.09	12.9%
Dennd4b	8.65737	0.40	4.6%
Dennd4c	1.3424	0.18	13.5%
Denr	26.38898	2.15	8.1%
Depdc1a	0.0577479	0.04	65.0%
Depdc1b	0.2054702	0.06	29.4%
Depdc2	0.9768836	0.16	16.3%
Depdc5	8.531148	0.59	6.9%
Depdc6	1.82411	0.28	15.5%
Depdc7	0.3841596	0.10	26.3%
Dera	2.712826	0.44	16.3%
Derl1	24.62456	1.34	5.5%
Derl2	12.41686	0.77	6.2%

Derl3	4.760386	0.93	19.6%
Des	0.00530678	0.01	138.1%
Det1	1.628004	0.15	9.1%
Dexi	7.72275	1.01	13.0%
Dffa	10.716306	1.05	9.8%
Dffb	2.102286	0.26	12.5%
Dfna5h	4.06545	0.20	4.9%
Dfnb59	0.20007584	0.07	36.8%
Dgat	5.053456	0.40	7.9%
Dgat1	11.5549	1.54	13.3%
Dgat2	9.328528	0.47	5.0%
Dgat2l3	0	0.00	
Dgat2l4	0.03360854	0.03	85.2%
Dgcr2	45.14858	1.26	2.8%
Dgcr6	20.7155	2.81	13.6%
Dgcr8	6.755958	0.38	5.6%
Dgka	0.9686358	0.06	5.9%
Dgkb	10.85808	0.63	5.8%
Dgkd	14.3964	2.62	18.2%
Dgke	10.026336	0.33	3.3%
Dgkg	2.77646	0.44	16.0%
Dgkh	1.376976	0.22	15.7%
Dgki	2.059896	0.70	34.1%
Dgkk	26.71832	1.04	3.9%
Dgkq	15.79598	2.39	15.1%
Dgkz	22.89576	1.00	4.4%
Dguok	6.291222	0.50	8.0%
Dhcr24	59.70556	8.87	14.9%
Dhcr7	28.62282	3.18	11.1%
Dhdds	21.73908	0.60	2.8%
Dhdh	0.4336736	0.08	19.1%
Dhfr	0.6295634	0.07	10.7%
Dhh	0.01770708	0.01	51.4%
Dhodh	4.445818	0.22	4.8%
Dhps	19.37052	1.47	7.6%
Dhrs1	17.99342	0.89	4.9%
Dhrs13	12.30264	1.11	9.0%
Dhrs2	0.0506506	0.03	50.9%
Dhrs3	1.364784	0.20	14.6%
Dhrs4	1.727082	0.13	7.5%
Dhrs7	6.514988	1.01	15.6%
Dhrs7b	10.28915	1.19	11.6%
Dhrs7c	0	0.00	
Dhrs9	0.00463358	0.01	137.3%
Dhrsx	0	0.00	
Dhtkd1	1.417104	0.23	16.3%
Dhx15	27.20844	0.82	3.0%

Dhx16	5.113486	0.25	5.0%
Dhx29	6.022178	0.73	12.2%
Dhx30	25.78256	0.58	2.2%
Dhx32	7.7941	0.68	8.8%
Dhx33	3.847822	0.27	7.0%
Dhx34	2.62505	0.12	4.5%
Dhx35	4.850418	0.19	3.8%
Dhx36	30.05	1.00	3.3%
Dhx37	3.48865	0.38	10.8%
Dhx38	6.99445	0.41	5.9%
Dhx40	6.494176	0.28	4.3%
Dhx57	20.84428	1.42	6.8%
Dhx58	0.1963776	0.07	33.4%
Dhx8	8.607676	0.21	2.5%
Dhx9	48.1314	2.06	4.3%
Dia2	0.3027146	0.15	49.8%
Diablo	7.850582	0.51	6.4%
Diap1	6.162974	0.29	4.7%
Diap2	1.61694	0.34	21.3%
Diap3	0.3935488	0.06	14.5%
Dicer1	7.348612	0.30	4.1%
Dido1	10.01055	0.51	5.1%
DIF-3	0.532667	0.13	25.1%
Dimt1	0.7631392	0.16	21.2%
Dinb1	0.5701452	0.13	22.6%
Dio1	0	0.00	
Dio-1	0.03191818	0.04	114.3%
Dio2	0.05096306	0.01	25.4%
Dio3	0.2965282	0.06	20.6%
Dip2a	10.96324	0.21	1.9%
Dip2b	2.63559	0.78	29.7%
Dip2c	20.41928	0.59	2.9%
Diras1	56.29508	1.71	3.0%
Diras2	19.566	1.72	8.8%
Dirc2	13.53898	0.23	1.7%
Dis3	5.669058	0.58	10.2%
Dis3l	7.626114	0.36	4.7%
Dis3l2	8.257144	0.30	3.6%
Disc1	0	0.00	
Disp1	0	0.00	
Disp2	91.18888	7.90	8.7%
Diws1t	0.57213718	0.51	88.5%
Dixdc1	9.843694	0.63	6.4%
Dkc1	5.59409	0.33	5.8%
Dkk1	0.1910644	0.06	32.6%
Dkk2	0.2680466	0.09	34.5%
Dkk3	1.440308	0.22	15.4%

Dkk-3	0.2342656	0.21	89.7%
Dkk4	0.00437516	0.01	223.6%
Dkkl1	0.01291876	0.02	138.8%
Dlat	48.13406	1.33	2.8%
Dlc1	4.690926	0.28	6.0%
Dld	70.50652	1.43	2.0%
Dlec1	0.13538376	0.05	35.0%
Dleu7	1.711182	0.24	14.1%
Dlg1	8.042396	0.41	5.1%
Dlg2	34.96596	0.89	2.5%
Dlg3	19.06188	0.97	5.1%
Dlg4	80.0291	3.83	4.8%
Dlg5	6.157606	0.16	2.6%
Dlg7	0.3777596	0.10	26.1%
Dlgap1	18.3263	1.08	5.9%
Dlgap2	2.684692	0.16	6.1%
Dlgap3	20.97352	1.84	8.8%
Dlgap4	24.38998	1.79	7.3%
Dlgh3	0.209464	0.06	27.8%
Dlk1	149.48562	48.72	32.6%
Dlk2	7.955772	0.64	8.0%
Dll1	2.053648	0.16	7.7%
Dll3	0.816112	0.07	8.9%
Dll4	0.089043058	0.06	72.0%
Dlst	64.12228	2.70	4.2%
Dlx1	0.030271342	0.02	50.3%
Dlx2	0.01597484	0.01	73.4%
Dlx3	0.028187106	0.02	79.9%
Dlx4	0.1743054	0.01	8.2%
Dlx5	0.0545219	0.05	98.5%
Dlx6	0.02233954	0.02	102.6%
Dmap1	10.68928	0.54	5.1%
Dmbt1	0.3566128	0.23	63.7%
Dmbx1	0.08452124	0.02	23.7%
Dmc1	0.10911844	0.01	11.0%
Dmd	6.8603	0.36	5.3%
Dmgdh	0.02983434	0.02	55.3%
Dmkn	0.04114818	0.03	78.1%
Dmn	3.534092	0.28	8.0%
Dmp1	0.1473795	0.04	30.4%
Dmpk	1.914822	0.39	20.1%
Dmrt1	0.00440284	0.01	223.6%
Dmrt2	0.469259	0.07	15.1%
Dmrt3	0.2477082	0.05	20.1%
Dmrta1	0.049471	0.01	21.3%
Dmrta2	0.3084936	0.06	19.4%
Dmrtb1	0.6570782	0.11	16.8%

Dmrtc1a	0.7593258	0.23	29.8%
Dmrtc1b	0	0.00	
Dmrtc1c	0	0.00	
Dmrtc2	0.01848308	0.02	105.1%
Dmtf1	14.856	1.09	7.3%
Dmwd	36.91992	1.46	4.0%
Dmxl1	6.133188	0.52	8.4%
Dmxl2	69.04356	2.72	3.9%
Dna2l	0.371487	0.10	27.4%
Dnahc1	0.03810544	0.06	144.5%
Dnahc11	0.02119274	0.01	23.7%
Dnahc12	0.00582974	0.01	223.6%
Dnahc17	0	0.00	
Dnahc2	0.1164588	0.02	15.3%
Dnahc3	0.038109122	0.02	48.6%
Dnahc5	0.02374198	0.00	20.0%
Dnahc6	0.0311942	0.04	143.4%
Dnahc8	0.038135286	0.03	81.9%
Dnahc9	0.4260614	0.15	35.8%
Dnaic1	0.13217378	0.04	30.5%
Dnaic2	0.1434874	0.03	23.3%
Dnaja1	24.2479	1.29	5.3%
Dnaja2	51.56366	2.13	4.1%
Dnaja3	28.4837	0.96	3.4%
Dnaja4	3.130512	0.23	7.3%
Dnajb1	20.68634	1.79	8.6%
Dnajb10	40.8265	1.18	2.9%
Dnajb11	25.12542	2.38	9.5%
Dnajb12	9.775952	0.86	8.8%
Dnajb13	0.0048707	0.01	223.6%
Dnajb14	9.721786	1.20	12.3%
Dnajb3	0.778383	0.29	37.9%
Dnajb4	23.6754	0.57	2.4%
Dnajb5	12.66502	0.96	7.6%
Dnajb6	7.457614	0.65	8.7%
Dnajb7	0.00585034	0.01	223.6%
Dnajb8	0	0.00	
Dnajb9	27.97914	1.60	5.7%
Dnajc1	2.495384	0.45	17.9%
Dnajc10	26.46646	1.07	4.1%
Dnajc11	23.51158	1.02	4.3%
Dnajc12	8.385974	0.54	6.5%
Dnajc13	10.143266	0.35	3.5%
Dnajc14	11.69016	0.20	1.7%
Dnajc15	21.68466	0.65	3.0%
Dnajc16	7.451506	0.41	5.5%
Dnajc17	1.274668	0.06	4.6%

Dnajc18	22.09326	1.34	6.1%
Dnajc19	1.515224	0.18	11.9%
Dnajc2	8.689336	0.86	9.9%
Dnajc3a	32.6304	2.56	7.8%
Dnajc4	10.430958	0.89	8.5%
Dnajc5	126.6454	2.83	2.2%
Dnajc5b	0	0.00	
Dnajc5g	0	0.00	
Dnajc6	65.62606	2.59	3.9%
Dnajc7	51.34082	2.38	4.6%
Dnajc8	25.77514	1.87	7.2%
Dnajc9	11.480578	1.20	10.5%
Dnalc1	5.695548	0.51	9.0%
Dnalc4	7.147322	0.30	4.1%
Dnali1	0.06988472	0.03	41.2%
dnam-1	0.013228226	0.01	76.2%
DNA-PKcs(Prkds)	1.2380186	0.41	33.4%
Dnase1	1.528986	0.41	26.9%
Dnase1l1	0.4037108	0.10	25.0%
Dnase1l2	1.548448	0.30	19.5%
Dnase1l3	0.00340089	0.00	137.7%
Dnase2a	2.238754	0.34	15.3%
Dnase2b	0	0.00	
Dnchc2	0.3459022	0.17	48.2%
Dncic2	0.10732116	0.05	47.1%
Dnd1	1.5604138	1.26	81.0%
Dner	84.36296	3.30	3.9%
Dnm1	210.0424	10.67	5.1%
Dnm1l	77.55308	2.41	3.1%
Dnm2	7.56679	0.37	4.9%
Dnm3	35.52348	3.41	9.6%
Dnm3os	1.0405074	0.17	16.4%
Dnmbp	1.98089	0.05	2.3%
Dnmt1	12.11744	0.66	5.4%
Dnmt3a	39.63626	1.08	2.7%
Dnmt3b	0.1573259	0.07	44.6%
Dnmt3l	0.13153156	0.07	56.7%
Dnpep	13.69632	1.13	8.3%
Dnt2	0.1931016	0.05	27.6%
Dntt	0	0.00	
Dnttip1	7.908406	0.69	8.7%
Dnttip2	7.667014	0.41	5.4%
Doc2a	4.198888	0.45	10.7%
Doc2b	1.300732	0.09	6.8%
Doc2g	2.425442	0.41	16.8%
Dock1	2.024178	0.18	9.0%
Dock10	0.8096472	0.48	58.7%

Dock11	3.972818	0.20	5.1%
Dock2	0.012132352	0.01	79.5%
Dock3	15.8101	0.64	4.1%
Dock4	7.538368	0.65	8.6%
Dock5	0.3384208	0.04	11.0%
Dock6	2.211794	0.41	18.6%
Dock7	7.701744	0.39	5.1%
Dock8	0.0841992	0.04	50.2%
Dock9	7.158796	0.28	3.9%
Dohh	27.73548	1.76	6.3%
Dok1	0.2283468	0.04	17.3%
Dok2	0.237538	0.04	15.2%
Dok3	1.216331	0.18	15.1%
Dok4	6.498988	0.35	5.5%
Dok5	13.80264	1.30	9.4%
Dok6	12.044582	1.79	14.8%
Dok7	2.233508	0.22	9.6%
DOKist4	3.632624	0.66	18.1%
Dolk	15.72696	1.36	8.6%
Dolpp1	10.432732	0.47	4.5%
Dom3z	3.30778	0.17	5.1%
Donson	6.262498	0.28	4.4%
Dopey1	9.408524	0.54	5.8%
Dopey2	13.62892	1.23	9.0%
Dorz1	0.6805062	0.27	40.2%
Dos	122.8334	6.60	5.4%
Dot1	1.788722	0.23	12.9%
Dot1l	4.71339	0.24	5.1%
Doxl2	0	0.00	
Dpagt1	9.57625	0.24	2.5%
Dpcr1	0.6171242	0.26	41.8%
Dpep1	0.7133238	0.37	51.4%
Dpep2	0.01529536	0.02	111.2%
Dpep3	0	0.00	
Dpf2	15.30322	0.76	5.0%
Dpf3	0.2293998	0.05	22.8%
Dph1	3.187564	0.16	4.9%
Dph2	2.649308	0.29	11.0%
Dph3	8.990424	0.80	8.9%
Dph4	2.425294	0.35	14.5%
Dph5	4.53685	0.33	7.2%
Dpm1	8.71867	0.61	7.0%
Dpm2	25.60264	1.86	7.3%
Dpm3	12.61538	1.35	10.7%
Dpp10	30.96404	1.00	3.2%
Dpp3	31.31462	1.48	4.7%
Dpp4	0.5682906	0.19	32.8%

Dpp6	75.9521	4.24	5.6%
Dpp7	4.658198	0.26	5.6%
Dpp8	34.38692	2.25	6.5%
Dpp9	17.60576	0.98	5.6%
Dppa1	0	0.00	
Dppa2	0.00333276	0.01	223.6%
Dppa3	0	0.00	
Dppa4	0	0.00	
Dppa5	0.0452832	0.06	137.3%
Dpt	0.2730634	0.13	48.1%
Dpy19l1	14.42088	0.36	2.5%
Dpy19l3	4.427018	0.46	10.4%
Dpy19l4	5.580338	0.64	11.4%
Dpyd	0.9976558	0.18	17.7%
Dpys	0.01699006	0.02	93.4%
Dpysl2	118.387	14.45	12.2%
Dpysl3	108.47486	14.72	13.6%
Dpysl4	28.91092	2.55	8.8%
Dpysl5	67.52734	8.89	13.2%
DQ059756	0	0.00	
DQ072380	0.11594006	0.03	28.5%
DQ072382	0.1276174	0.04	32.4%
DQ072386	56.62878	5.95	10.5%
DQ072390	5.895552	0.53	9.0%
DQ266428	1.3757616	0.39	28.2%
DQ279480	0	0.00	
DQ284430	0	0.00	
DQ354407	0.0267022	0.02	68.8%
DQ539905	0	0.00	
DQ539915	7272.806	1117.58	15.4%
DQ539956	0	0.00	
DQ539985	0	0.00	
DQ539992	0	0.00	
DQ539999	0	0.00	
DQ540009	0	0.00	
DQ540032	0	0.00	
DQ540050	0	0.00	
DQ540060	0	0.00	
DQ540096	0	0.00	
DQ540135	0	0.00	
DQ540160	0	0.00	
DQ540165	0	0.00	
DQ540208	0	0.00	
DQ540256	0	0.00	
DQ540266	0	0.00	
DQ540318	0	0.00	
DQ540346	0	0.00	

DQ540358	0	0.00	
DQ540360	0	0.00	
DQ540388	0	0.00	
DQ540432	0	0.00	
DQ540451	0	0.00	
DQ540490	0	0.00	
DQ540523	0	0.00	
DQ540525	0	0.00	
DQ540543	0	0.00	
DQ540574	0	0.00	
DQ540576	0	0.00	
DQ540649	0	0.00	
DQ540688	0	0.00	
DQ540690	0	0.00	
DQ540716	0	0.00	
DQ540739	0	0.00	
DQ540775	0	0.00	
DQ540809	0	0.00	
DQ540885	0	0.00	
DQ540907	0	0.00	
DQ540913	0	0.00	
DQ540925	0	0.00	
DQ540943	0	0.00	
DQ540995	0	0.00	
DQ541037	0	0.00	
DQ541157	0	0.00	
DQ541158	0	0.00	
DQ541184	0	0.00	
DQ541188	0	0.00	
DQ541191	0	0.00	
DQ541234	0	0.00	
DQ541263	0	0.00	
DQ541300	0	0.00	
DQ541423	0	0.00	
DQ541485	0	0.00	
DQ541502	0	0.00	
DQ541645	0	0.00	
DQ541658	0	0.00	
DQ541662	0	0.00	
DQ541699	0	0.00	
DQ541735	0	0.00	
DQ541751	0	0.00	
DQ541757	0	0.00	
DQ541758	0	0.00	
DQ541762	0	0.00	
DQ541918	0	0.00	
DQ541956	0	0.00	

DQ541958	0	0.00	
DQ541975	0	0.00	
DQ542007	0	0.00	
DQ542029	0	0.00	
DQ542042	0	0.00	
DQ542114	0	0.00	
DQ542172	0	0.00	
DQ542181	0	0.00	
DQ542236	0	0.00	
DQ542264	0	0.00	
DQ542302	0	0.00	
DQ542332	0	0.00	
DQ542353	0	0.00	
DQ542380	0	0.00	
DQ542393	0	0.00	
DQ542397	0	0.00	
DQ542547	0	0.00	
DQ542586	0	0.00	
DQ542656	0	0.00	
DQ542709	0	0.00	
DQ542726	0	0.00	
DQ542751	0	0.00	
DQ542758	0	0.00	
DQ542762	0	0.00	
DQ542813	0	0.00	
DQ542896	0.171312	0.38	223.6%
DQ542917	0	0.00	
DQ542949	0	0.00	
DQ542954	0	0.00	
DQ542957	0	0.00	
DQ542960	0	0.00	
DQ543008	0	0.00	
DQ543012	0	0.00	
DQ543022	0	0.00	
DQ543031	0	0.00	
DQ543127	0	0.00	
DQ543173	0	0.00	
DQ543186	0	0.00	
DQ543219	0	0.00	
DQ543221	0	0.00	
DQ543265	0	0.00	
DQ543350	0	0.00	
DQ543356	0	0.00	
DQ543366	0	0.00	
DQ543375	0	0.00	
DQ543391	0	0.00	
DQ543393	0	0.00	

DQ543396	0	0.00	
DQ543397	0	0.00	
DQ543428	0	0.00	
DQ543550	0	0.00	
DQ543635	0	0.00	
DQ543648	0	0.00	
DQ543661	0	0.00	
DQ543687	0	0.00	
DQ543725	0	0.00	
DQ543728	0	0.00	
DQ543747	0	0.00	
DQ543754	0	0.00	
DQ543758	0	0.00	
DQ543801	0	0.00	
DQ543820	0	0.00	
DQ543863	0	0.00	
DQ543883	0	0.00	
DQ543890	0	0.00	
DQ544002	0	0.00	
DQ544060	0	0.00	
DQ544102	0	0.00	
DQ544141	0	0.00	
DQ544170	0	0.00	
DQ544183	0	0.00	
DQ544189	0	0.00	
DQ544201	0	0.00	
DQ544250	0	0.00	
DQ544261	0	0.00	
DQ544285	0	0.00	
DQ544332	0	0.00	
DQ544467	0	0.00	
DQ544492	0	0.00	
DQ544519	0	0.00	
DQ544534	0	0.00	
DQ544591	0	0.00	
DQ544595	0	0.00	
DQ544597	0	0.00	
DQ544617	0	0.00	
DQ544646	0	0.00	
DQ544660	0	0.00	
DQ544692	0	0.00	
DQ544708	0	0.00	
DQ544744	0	0.00	
DQ544756	0	0.00	
DQ544783	0	0.00	
DQ544804	0	0.00	
DQ544812	0	0.00	

DQ544838	0	0.00	
DQ544871	0	0.00	
DQ544893	0	0.00	
DQ544949	0	0.00	
DQ545110	0	0.00	
DQ545168	0	0.00	
DQ545188	0	0.00	
DQ545191	0	0.00	
DQ545246	0	0.00	
DQ545254	0	0.00	
DQ545285	0	0.00	
DQ545291	0	0.00	
DQ545332	0	0.00	
DQ545414	0	0.00	
DQ545416	0	0.00	
DQ545447	0	0.00	
DQ545475	0	0.00	
DQ545486	0	0.00	
DQ545495	0	0.00	
DQ545576	0	0.00	
DQ545582	0	0.00	
DQ545600	0	0.00	
DQ545635	0	0.00	
DQ545645	0	0.00	
DQ545740	0	0.00	
DQ545755	0	0.00	
DQ545773	0	0.00	
DQ545796	0	0.00	
DQ545816	0	0.00	
DQ545830	0	0.00	
DQ545836	0	0.00	
DQ545837	0	0.00	
DQ545846	0	0.00	
DQ545882	0	0.00	
DQ545902	0	0.00	
DQ545935	0	0.00	
DQ545990	0	0.00	
DQ546002	0	0.00	
DQ546008	0	0.00	
DQ546014	0	0.00	
DQ546033	0	0.00	
DQ546036	0	0.00	
DQ546052	0	0.00	
DQ546121	0	0.00	
DQ546201	0	0.00	
DQ546209	0	0.00	
DQ546218	0	0.00	

DQ546236	0	0.00	
DQ546271	0	0.00	
DQ546316	0	0.00	
DQ546340	0	0.00	
DQ546366	0	0.00	
DQ546372	0	0.00	
DQ546400	0	0.00	
DQ546448	0	0.00	
DQ546462	0	0.00	
DQ546464	0	0.00	
DQ546489	0	0.00	
DQ546527	0	0.00	
DQ546551	0	0.00	
DQ546569	0	0.00	
DQ546580	0	0.00	
DQ546601	0	0.00	
DQ546614	0	0.00	
DQ546624	0	0.00	
DQ546625	0	0.00	
DQ546629	0	0.00	
DQ546809	0	0.00	
DQ546822	0	0.00	
DQ546911	0	0.00	
DQ546924	0	0.00	
DQ547015	0	0.00	
DQ547048	0	0.00	
DQ547059	0	0.00	
DQ547091	0	0.00	
DQ547106	0	0.00	
DQ547177	0	0.00	
DQ547185	0	0.00	
DQ547205	0	0.00	
DQ547239	0	0.00	
DQ547277	0	0.00	
DQ547388	0	0.00	
DQ547412	0	0.00	
DQ547422	0	0.00	
DQ547437	0	0.00	
DQ547438	0	0.00	
DQ547453	0	0.00	
DQ547458	0	0.00	
DQ547490	0	0.00	
DQ547491	0	0.00	
DQ547508	0	0.00	
DQ547562	0	0.00	
DQ547595	0	0.00	
DQ547645	0	0.00	

DQ547689	0	0.00	
DQ547696	0	0.00	
DQ547698	0	0.00	
DQ547719	0	0.00	
DQ547751	0	0.00	
DQ547778	0	0.00	
DQ547822	0	0.00	
DQ547823	0	0.00	
DQ547894	0	0.00	
DQ547909	0	0.00	
DQ547910	0	0.00	
DQ547919	0	0.00	
DQ547934	0	0.00	
DQ547941	0	0.00	
DQ547972	0	0.00	
DQ547976	0	0.00	
DQ547994	0	0.00	
DQ548009	0	0.00	
DQ548015	0	0.00	
DQ548051	0	0.00	
DQ548068	0	0.00	
DQ548087	0	0.00	
DQ548090	0	0.00	
DQ548100	0	0.00	
DQ548101	0	0.00	
DQ548140	0	0.00	
DQ548180	0	0.00	
DQ548190	0	0.00	
DQ548214	0	0.00	
DQ548268	0	0.00	
DQ548336	0	0.00	
DQ548344	0	0.00	
DQ548345	0	0.00	
DQ548355	0	0.00	
DQ548393	0	0.00	
DQ548591	0	0.00	
DQ548624	0	0.00	
DQ548697	0	0.00	
DQ548704	0	0.00	
DQ548855	0	0.00	
DQ548859	0	0.00	
DQ548964	0	0.00	
DQ548994	6.979926	0.55	7.9%
DQ549025	0	0.00	
DQ549026	0	0.00	
DQ549082	0	0.00	
DQ549109	0	0.00	

DQ549139	0	0.00	
DQ549194	0	0.00	
DQ549198	0	0.00	
DQ549212	0	0.00	
DQ549220	0	0.00	
DQ549221	0	0.00	
DQ549223	0	0.00	
DQ549229	0	0.00	
DQ549248	0	0.00	
DQ549253	0	0.00	
DQ549285	0	0.00	
DQ549294	0	0.00	
DQ549305	0	0.00	
DQ549323	0	0.00	
DQ549365	0	0.00	
DQ549375	0	0.00	
DQ549406	0	0.00	
DQ549471	0	0.00	
DQ549481	0	0.00	
DQ549511	0	0.00	
DQ549545	0	0.00	
DQ549561	0	0.00	
DQ549573	0	0.00	
DQ549577	0	0.00	
DQ549578	0	0.00	
DQ549582	0	0.00	
DQ549597	0	0.00	
DQ549608	0	0.00	
DQ549625	0	0.00	
DQ549643	0	0.00	
DQ549647	0	0.00	
DQ549692	0	0.00	
DQ549709	0	0.00	
DQ549711	0	0.00	
DQ549720	0	0.00	
DQ549732	0	0.00	
DQ549737	0	0.00	
DQ549743	0	0.00	
DQ549744	0	0.00	
DQ549792	0	0.00	
DQ549875	0	0.00	
DQ549901	0	0.00	
DQ549908	0	0.00	
DQ549935	0	0.00	
DQ549940	0	0.00	
DQ549963	0	0.00	
DQ549971	0	0.00	

DQ550004	0	0.00	
DQ550042	0	0.00	
DQ550048	0	0.00	
DQ550093	0	0.00	
DQ550104	0	0.00	
DQ550209	0	0.00	
DQ550222	0	0.00	
DQ550225	0	0.00	
DQ550247	0	0.00	
DQ550253	0	0.00	
DQ550256	0	0.00	
DQ550274	0	0.00	
DQ550326	0	0.00	
DQ550364	0	0.00	
DQ550368	0	0.00	
DQ550371	0	0.00	
DQ550390	0	0.00	
DQ550407	0	0.00	
DQ550425	0	0.00	
DQ550471	0	0.00	
DQ550484	0	0.00	
DQ550513	0	0.00	
DQ550549	0	0.00	
DQ550564	0	0.00	
DQ550594	0	0.00	
DQ550606	0	0.00	
DQ550639	0	0.00	
DQ550653	0	0.00	
DQ550686	0	0.00	
DQ550701	0	0.00	
DQ550788	0	0.00	
DQ550824	0	0.00	
DQ550843	0	0.00	
DQ550871	0	0.00	
DQ550879	0	0.00	
DQ550935	0	0.00	
DQ550973	0	0.00	
DQ551000	0	0.00	
DQ551003	0	0.00	
DQ551006	0	0.00	
DQ551012	0	0.00	
DQ551037	0	0.00	
DQ551069	0	0.00	
DQ551097	0	0.00	
DQ551102	0	0.00	
DQ551105	0	0.00	
DQ551112	0	0.00	

DQ551195	0	0.00	
DQ551281	0	0.00	
DQ551325	0	0.00	
DQ551326	0	0.00	
DQ551367	0	0.00	
DQ551402	37.33	1.01	2.7%
DQ551428	0	0.00	
DQ551495	0	0.00	
DQ551521	0	0.00	
DQ551543	0	0.00	
DQ551615	0	0.00	
DQ551641	0	0.00	
DQ551692	0	0.00	
DQ551725	0	0.00	
DQ551786	0	0.00	
DQ551812	0	0.00	
DQ551828	0	0.00	
DQ551845	0	0.00	
DQ551892	0	0.00	
DQ552053	0	0.00	
DQ552069	0	0.00	
DQ552147	0	0.00	
DQ552243	0	0.00	
DQ552245	0	0.00	
DQ552264	0	0.00	
DQ552305	0	0.00	
DQ552381	0	0.00	
DQ552388	0	0.00	
DQ552415	0	0.00	
DQ552423	0	0.00	
DQ552427	0	0.00	
DQ552475	0	0.00	
DQ552494	0	0.00	
DQ552539	0	0.00	
DQ552563	0	0.00	
DQ552568	0	0.00	
DQ552585	0	0.00	
DQ552594	0	0.00	
DQ552655	0	0.00	
DQ552759	0	0.00	
DQ552812	0	0.00	
DQ552872	0	0.00	
DQ552897	0	0.00	
DQ552899	0	0.00	
DQ552940	0	0.00	
DQ552986	0	0.00	
DQ552992	0	0.00	

DQ553019	0	0.00	
DQ553039	0	0.00	
DQ553098	0	0.00	
DQ553099	0	0.00	
DQ553108	0	0.00	
DQ553114	0	0.00	
DQ553243	0	0.00	
DQ553245	4.801712	0.41	8.6%
DQ553248	0	0.00	
DQ553291	0	0.00	
DQ553300	0	0.00	
DQ553325	0	0.00	
DQ553341	0	0.00	
DQ553350	0	0.00	
DQ553376	0	0.00	
DQ553419	0	0.00	
DQ553449	0	0.00	
DQ553469	0	0.00	
DQ553502	0	0.00	
DQ553514	0	0.00	
DQ553546	0	0.00	
DQ553547	0	0.00	
DQ553550	0	0.00	
DQ553589	0	0.00	
DQ553595	0	0.00	
DQ553618	0	0.00	
DQ553663	0	0.00	
DQ553711	0	0.00	
DQ553778	0	0.00	
DQ553816	0	0.00	
DQ553827	0	0.00	
DQ553839	0	0.00	
DQ553872	0	0.00	
DQ553881	0	0.00	
DQ553941	0	0.00	
DQ553947	0	0.00	
DQ553967	0	0.00	
DQ553972	0	0.00	
DQ553995	0	0.00	
DQ554009	0	0.00	
DQ554043	0	0.00	
DQ554056	0	0.00	
DQ554077	0	0.00	
DQ554087	0	0.00	
DQ554143	0	0.00	
DQ554183	0	0.00	
DQ554213	0	0.00	

DQ554228	0	0.00	
DQ554281	0	0.00	
DQ554286	0	0.00	
DQ554303	0	0.00	
DQ554362	0	0.00	
DQ554395	0	0.00	
DQ554412	0	0.00	
DQ554414	0	0.00	
DQ554417	0	0.00	
DQ554434	0	0.00	
DQ554544	0	0.00	
DQ554622	0	0.00	
DQ554713	0	0.00	
DQ554718	0	0.00	
DQ554735	0	0.00	
DQ554737	0	0.00	
DQ554761	0	0.00	
DQ554804	0	0.00	
DQ554814	0	0.00	
DQ554951	0	0.00	
DQ555000	0	0.00	
DQ555008	0	0.00	
DQ555031	0	0.00	
DQ555142	0	0.00	
DQ555188	0	0.00	
DQ555193	0	0.00	
DQ555209	0	0.00	
DQ555237	0	0.00	
DQ555272	0	0.00	
DQ555274	0	0.00	
DQ555312	0	0.00	
DQ555342	0	0.00	
DQ555423	0	0.00	
DQ555424	0	0.00	
DQ555432	0	0.00	
DQ555438	0	0.00	
DQ555442	0	0.00	
DQ555443	0	0.00	
DQ555454	0	0.00	
DQ555467	0	0.00	
DQ555475	0	0.00	
DQ555500	0	0.00	
DQ555528	0	0.00	
DQ555542	0	0.00	
DQ555555	0	0.00	
DQ555568	0	0.00	
DQ555617	0	0.00	

DQ555620	0	0.00	
DQ555636	0	0.00	
DQ555679	0	0.00	
DQ555728	0	0.00	
DQ555770	0	0.00	
DQ555790	0	0.00	
DQ555931	0	0.00	
DQ555935	0	0.00	
DQ555983	0	0.00	
DQ556009	0	0.00	
DQ556017	0	0.00	
DQ556020	0	0.00	
DQ556070	0	0.00	
DQ556074	0	0.00	
DQ556080	0	0.00	
DQ556087	0	0.00	
DQ556096	0	0.00	
DQ556130	0	0.00	
DQ556132	0	0.00	
DQ556136	0	0.00	
DQ556154	0	0.00	
DQ556157	0	0.00	
DQ556167	0	0.00	
DQ556184	0	0.00	
DQ556191	0	0.00	
DQ556195	0	0.00	
DQ556208	0	0.00	
DQ556210	0	0.00	
DQ556220	0	0.00	
DQ556266	0	0.00	
DQ556290	0	0.00	
DQ556292	0	0.00	
DQ556349	0	0.00	
DQ556350	0	0.00	
DQ556351	0	0.00	
DQ556384	0	0.00	
DQ556396	0	0.00	
DQ556400	0	0.00	
DQ556462	0	0.00	
DQ556485	0	0.00	
DQ556509	0	0.00	
DQ556518	0	0.00	
DQ556540	0	0.00	
DQ556660	0	0.00	
DQ556670	0	0.00	
DQ556681	0	0.00	
DQ556759	0	0.00	

DQ556775	0	0.00	
DQ556784	0	0.00	
DQ556786	0	0.00	
DQ556830	0	0.00	
DQ556844	0	0.00	
DQ556852	0	0.00	
DQ556909	0	0.00	
DQ556983	0	0.00	
DQ557000	0	0.00	
DQ557036	0	0.00	
DQ557075	0	0.00	
DQ557199	0	0.00	
DQ557218	0	0.00	
DQ557254	0	0.00	
DQ557261	0	0.00	
DQ557269	0	0.00	
DQ557287	0	0.00	
DQ557309	0	0.00	
DQ557314	0	0.00	
DQ557323	0	0.00	
DQ557327	0	0.00	
DQ557359	0	0.00	
DQ557413	0	0.00	
DQ557426	0	0.00	
DQ557433	0	0.00	
DQ557440	0	0.00	
DQ557517	0	0.00	
DQ557551	0	0.00	
DQ557648	0	0.00	
DQ557665	0	0.00	
DQ557693	0	0.00	
DQ557702	0	0.00	
DQ557715	0	0.00	
DQ557814	0	0.00	
DQ557880	0	0.00	
DQ557896	0	0.00	
DQ557907	0	0.00	
DQ557910	0	0.00	
DQ557960	0	0.00	
DQ558014	0	0.00	
DQ558020	0	0.00	
DQ558043	0	0.00	
DQ558065	0	0.00	
DQ558068	0	0.00	
DQ558095	0	0.00	
DQ558102	0	0.00	
DQ558110	0	0.00	

DQ558121	0	0.00	
DQ558230	0	0.00	
DQ558423	0	0.00	
DQ558508	0	0.00	
DQ558583	0	0.00	
DQ558605	0	0.00	
DQ558686	0	0.00	
DQ558780	0	0.00	
DQ558791	0	0.00	
DQ558885	0	0.00	
DQ558919	0	0.00	
DQ558960	0	0.00	
DQ558976	0	0.00	
DQ559130	0	0.00	
DQ559163	0	0.00	
DQ559474	0	0.00	
DQ559597	0	0.00	
DQ559606	0	0.00	
DQ559648	0	0.00	
DQ559678	0	0.00	
DQ559926	0	0.00	
DQ559937	0	0.00	
DQ560024	0	0.00	
DQ560072	0	0.00	
DQ560122	0	0.00	
DQ560132	0	0.00	
DQ560189	0	0.00	
DQ560204	0	0.00	
DQ560267	0	0.00	
DQ560276	0	0.00	
DQ560318	0	0.00	
DQ560399	0	0.00	
DQ560408	0	0.00	
DQ560421	0	0.00	
DQ560459	0	0.00	
DQ560463	0	0.00	
DQ560483	0	0.00	
DQ560594	0	0.00	
DQ560613	0	0.00	
DQ560618	0	0.00	
DQ560624	0	0.00	
DQ560645	0	0.00	
DQ560654	0	0.00	
DQ560656	0	0.00	
DQ560661	0	0.00	
DQ560729	0	0.00	
DQ560731	0	0.00	

DQ560765	0	0.00	
DQ560772	3.245092	0.87	26.8%
DQ560842	0	0.00	
DQ560888	0	0.00	
DQ560976	0	0.00	
DQ560983	0	0.00	
DQ561039	0	0.00	
DQ561056	0	0.00	
DQ561066	0	0.00	
DQ561150	0	0.00	
DQ561154	0	0.00	
DQ561165	0	0.00	
DQ561175	0	0.00	
DQ561259	0	0.00	
DQ561275	0	0.00	
DQ561281	0	0.00	
DQ561286	0	0.00	
DQ561289	0	0.00	
DQ561333	0	0.00	
DQ561355	0	0.00	
DQ561358	0	0.00	
DQ561359	0	0.00	
DQ561364	0	0.00	
DQ561366	0	0.00	
DQ561371	0	0.00	
DQ561373	0	0.00	
DQ561384	0	0.00	
DQ561391	0	0.00	
DQ561419	0	0.00	
DQ561426	0	0.00	
DQ561518	0	0.00	
DQ561527	0	0.00	
DQ561547	0	0.00	
DQ561579	0	0.00	
DQ561616	0.7618328	0.58	76.0%
DQ561682	0	0.00	
DQ561744	0	0.00	
DQ561781	0	0.00	
DQ561798	0	0.00	
DQ561838	0	0.00	
DQ561892	0	0.00	
DQ561908	0	0.00	
DQ561922	0	0.00	
DQ561948	0	0.00	
DQ562011	0	0.00	
DQ562020	0	0.00	
DQ562057	0	0.00	

DQ562103	0	0.00	
DQ562131	0	0.00	
DQ562178	0	0.00	
DQ562194	0	0.00	
DQ562205	0	0.00	
DQ562220	0	0.00	
DQ562229	0	0.00	
DQ562254	0	0.00	
DQ562286	0	0.00	
DQ562297	0	0.00	
DQ562327	0	0.00	
DQ562345	0	0.00	
DQ562358	0	0.00	
DQ562384	0	0.00	
DQ562395	0	0.00	
DQ562459	0	0.00	
DQ562467	0	0.00	
DQ562510	0	0.00	
DQ562530	0	0.00	
DQ562556	0	0.00	
DQ562562	0	0.00	
DQ562580	0	0.00	
DQ562653	0	0.00	
DQ562682	0	0.00	
DQ562719	0	0.00	
DQ562746	0	0.00	
DQ562758	0	0.00	
DQ562776	0	0.00	
DQ562815	0	0.00	
DQ562822	0	0.00	
DQ562857	0	0.00	
DQ562875	0	0.00	
DQ562876	0	0.00	
DQ562883	0	0.00	
DQ562927	0	0.00	
DQ562964	0	0.00	
DQ562991	0	0.00	
DQ562993	0	0.00	
DQ562995	0	0.00	
DQ563063	0	0.00	
DQ563137	0	0.00	
DQ563224	0	0.00	
DQ563230	0	0.00	
DQ563315	0	0.00	
DQ563349	0	0.00	
DQ563356	0	0.00	
DQ563368	0	0.00	

DQ563380	0	0.00	
DQ563452	0	0.00	
DQ563495	0	0.00	
DQ563500	0	0.00	
DQ563503	0	0.00	
DQ563505	0	0.00	
DQ563521	0	0.00	
DQ563597	0	0.00	
DQ563604	0	0.00	
DQ563616	0	0.00	
DQ563634	0	0.00	
DQ563658	0	0.00	
DQ563704	0	0.00	
DQ563733	0	0.00	
DQ563835	0	0.00	
DQ563867	0	0.00	
DQ563884	0	0.00	
DQ563906	0	0.00	
DQ563910	0	0.00	
DQ564011	0	0.00	
DQ564048	0	0.00	
DQ564056	0	0.00	
DQ564081	0	0.00	
DQ564178	0	0.00	
DQ564199	0	0.00	
DQ564238	0	0.00	
DQ564276	0	0.00	
DQ564299	0	0.00	
DQ564327	0	0.00	
DQ564349	0	0.00	
DQ564413	0	0.00	
DQ564424	0	0.00	
DQ564426	0	0.00	
DQ564444	0	0.00	
DQ564498	0	0.00	
DQ564514	0	0.00	
DQ564518	0	0.00	
DQ564566	0	0.00	
DQ564575	0	0.00	
DQ564592	0	0.00	
DQ564602	0	0.00	
DQ564617	0	0.00	
DQ564628	0	0.00	
DQ564716	0	0.00	
DQ564750	0	0.00	
DQ564778	0	0.00	
DQ564813	0	0.00	

DQ564818	0	0.00	
DQ564835	0	0.00	
DQ564838	0	0.00	
DQ564894	0	0.00	
DQ564907	0	0.00	
DQ564912	0	0.00	
DQ564920	0	0.00	
DQ564937	0	0.00	
DQ564948	0	0.00	
DQ564951	0	0.00	
DQ565026	0	0.00	
DQ565064	0	0.00	
DQ565074	0	0.00	
DQ565149	0	0.00	
DQ565203	0	0.00	
DQ565213	0	0.00	
DQ565253	0	0.00	
DQ565270	0	0.00	
DQ565313	0	0.00	
DQ565376	0	0.00	
DQ565479	0	0.00	
DQ565491	0	0.00	
DQ565497	0	0.00	
DQ565516	0	0.00	
DQ565595	0	0.00	
DQ565607	0	0.00	
DQ565635	0	0.00	
DQ565646	0	0.00	
DQ565670	0	0.00	
DQ565709	0	0.00	
DQ565717	0	0.00	
DQ565738	0	0.00	
DQ565789	0	0.00	
DQ565798	0	0.00	
DQ565828	0	0.00	
DQ565854	0	0.00	
DQ565859	0	0.00	
DQ565975	0	0.00	
DQ565977	0	0.00	
DQ565978	0	0.00	
DQ566004	0	0.00	
DQ566013	0	0.00	
DQ566016	0	0.00	
DQ566045	0	0.00	
DQ566107	0	0.00	
DQ566114	0	0.00	
DQ566132	0	0.00	

DQ566296	0	0.00	
DQ566326	0	0.00	
DQ566337	0	0.00	
DQ566408	0	0.00	
DQ566446	0	0.00	
DQ566455	0	0.00	
DQ566462	0	0.00	
DQ566510	0	0.00	
DQ566529	0	0.00	
DQ566590	0	0.00	
DQ566622	0	0.00	
DQ566656	0	0.00	
DQ566704	0	0.00	
DQ566736	0	0.00	
DQ566763	0	0.00	
DQ566768	0	0.00	
DQ566781	0	0.00	
DQ566885	0	0.00	
DQ566912	0	0.00	
DQ566918	0	0.00	
DQ566922	0	0.00	
DQ567021	0	0.00	
DQ567042	0	0.00	
DQ567094	0	0.00	
DQ567125	0	0.00	
DQ567154	0	0.00	
DQ567160	0	0.00	
DQ567356	0	0.00	
DQ567367	0	0.00	
DQ567429	0	0.00	
DQ567446	0	0.00	
DQ567479	0	0.00	
DQ567480	0	0.00	
DQ567485	0	0.00	
DQ567513	0	0.00	
DQ567533	0	0.00	
DQ567545	0	0.00	
DQ567628	0	0.00	
DQ567655	0	0.00	
DQ567693	0	0.00	
DQ567724	0	0.00	
DQ567784	0	0.00	
DQ567786	0	0.00	
DQ567796	0	0.00	
DQ567835	0	0.00	
DQ567862	0	0.00	
DQ567872	0	0.00	

DQ567887	0	0.00	
DQ567921	0	0.00	
DQ567966	0	0.00	
DQ567985	0	0.00	
DQ567995	0	0.00	
DQ568005	0	0.00	
DQ568071	0	0.00	
DQ568095	0	0.00	
DQ568116	0	0.00	
DQ568121	0	0.00	
DQ568127	0	0.00	
DQ568153	0	0.00	
DQ568193	0	0.00	
DQ568245	0	0.00	
DQ568346	0	0.00	
DQ568352	0	0.00	
DQ568359	0	0.00	
DQ568384	0	0.00	
DQ568433	0	0.00	
DQ568457	0	0.00	
DQ568458	0	0.00	
DQ568508	0	0.00	
DQ568510	0	0.00	
DQ568525	0	0.00	
DQ568561	0	0.00	
DQ568566	0	0.00	
DQ568592	0	0.00	
DQ568607	0	0.00	
DQ568633	0	0.00	
DQ568657	0	0.00	
DQ568672	0	0.00	
DQ568728	0	0.00	
DQ568730	0	0.00	
DQ568775	0	0.00	
DQ568792	0	0.00	
DQ568855	0	0.00	
DQ568862	0	0.00	
DQ568925	0	0.00	
DQ568963	0	0.00	
DQ568996	0	0.00	
DQ568998	0	0.00	
DQ569210	0	0.00	
DQ569229	0	0.00	
DQ569232	0	0.00	
DQ569248	0	0.00	
DQ569264	0	0.00	
DQ569270	0	0.00	

DQ569294	0	0.00	
DQ569369	0	0.00	
DQ569402	0	0.00	
DQ569434	0	0.00	
DQ569435	0	0.00	
DQ569443	0	0.00	
DQ569461	0	0.00	
DQ569478	0	0.00	
DQ569489	0	0.00	
DQ569537	0	0.00	
DQ569544	0	0.00	
DQ569553	0	0.00	
DQ569558	0	0.00	
DQ569600	0	0.00	
DQ569627	0	0.00	
DQ569637	0	0.00	
DQ569657	0	0.00	
DQ569686	0	0.00	
DQ569721	0	0.00	
DQ569722	0	0.00	
DQ569723	0	0.00	
DQ569753	0	0.00	
DQ569787	0	0.00	
DQ569827	0	0.00	
DQ569880	0	0.00	
DQ569883	0	0.00	
DQ569891	0	0.00	
DQ684691	0	0.00	
DQ684694	0	0.00	
DQ684698	0	0.00	
DQ684713	0	0.00	
DQ684763	0	0.00	
DQ684769	0	0.00	
DQ684799	0	0.00	
DQ684830	0	0.00	
DQ684877	0	0.00	
DQ684881	0	0.00	
DQ684909	0	0.00	
DQ684910	0	0.00	
DQ684917	0	0.00	
DQ684929	0	0.00	
DQ685022	0	0.00	
DQ685054	0	0.00	
DQ685077	0	0.00	
DQ685082	0	0.00	
DQ685100	0	0.00	
DQ685101	0	0.00	

DQ685106	0	0.00	
DQ685109	0	0.00	
DQ685120	0	0.00	
DQ685128	0	0.00	
DQ685134	0	0.00	
DQ685173	0	0.00	
DQ685176	0	0.00	
DQ685187	0	0.00	
DQ685202	0	0.00	
DQ685226	0	0.00	
DQ685229	0	0.00	
DQ685280	0	0.00	
DQ685289	0	0.00	
DQ685298	0	0.00	
DQ685301	0	0.00	
DQ685312	0	0.00	
DQ685325	0	0.00	
DQ685350	0	0.00	
DQ685369	0	0.00	
DQ685458	0	0.00	
DQ685460	0	0.00	
DQ685524	0	0.00	
DQ685562	0	0.00	
DQ685565	0	0.00	
DQ685593	0	0.00	
DQ685610	0	0.00	
DQ685618	0	0.00	
DQ685621	0	0.00	
DQ685631	0	0.00	
DQ685645	0	0.00	
DQ685657	0	0.00	
DQ685694	0	0.00	
DQ685795	0	0.00	
DQ685806	0	0.00	
DQ685809	0	0.00	
DQ685811	0	0.00	
DQ685891	0	0.00	
DQ685895	0	0.00	
DQ685907	0	0.00	
DQ685919	0	0.00	
DQ685946	0	0.00	
DQ685947	0	0.00	
DQ685974	0	0.00	
DQ685977	0	0.00	
DQ686003	0	0.00	
DQ686008	0	0.00	
DQ686014	0	0.00	

DQ686029	0	0.00	
DQ686040	0	0.00	
DQ686042	0	0.00	
DQ686061	0	0.00	
DQ686091	0	0.00	
DQ686092	0	0.00	
DQ686107	0	0.00	
DQ686125	0	0.00	
DQ686126	0	0.00	
DQ686130	0	0.00	
DQ686138	0	0.00	
DQ686143	0	0.00	
DQ686167	0	0.00	
DQ686181	0	0.00	
DQ686182	0	0.00	
DQ686199	0	0.00	
DQ686205	0	0.00	
DQ686227	0	0.00	
DQ686266	0	0.00	
DQ686274	0	0.00	
DQ686289	0	0.00	
DQ686310	0	0.00	
DQ686332	0	0.00	
DQ686359	0	0.00	
DQ686370	0	0.00	
DQ686384	0	0.00	
DQ686386	0	0.00	
DQ686389	0	0.00	
DQ686395	0	0.00	
DQ686411	0	0.00	
DQ686414	0	0.00	
DQ686437	0	0.00	
DQ686454	0	0.00	
DQ686472	0	0.00	
DQ686506	0	0.00	
DQ686518	0	0.00	
DQ686547	0	0.00	
DQ686550	0	0.00	
DQ686571	0	0.00	
DQ686574	0	0.00	
DQ686600	0	0.00	
DQ686635	0	0.00	
DQ686644	0	0.00	
DQ686655	0	0.00	
DQ686667	0	0.00	
DQ686674	0	0.00	
DQ686676	0	0.00	

DQ686689	0	0.00	
DQ686693	0	0.00	
DQ686707	0	0.00	
DQ686731	0	0.00	
DQ686742	0	0.00	
DQ686744	0	0.00	
DQ686750	0	0.00	
DQ686802	0	0.00	
DQ686814	0	0.00	
DQ686818	0	0.00	
DQ686862	0	0.00	
DQ686870	0	0.00	
DQ686953	0	0.00	
DQ686960	0	0.00	
DQ686988	0	0.00	
DQ687013	0	0.00	
DQ687046	0	0.00	
DQ687084	0	0.00	
DQ687099	0	0.00	
DQ687106	0	0.00	
DQ687127	0	0.00	
DQ687132	0	0.00	
DQ687150	0	0.00	
DQ687153	0	0.00	
DQ687156	0	0.00	
DQ687171	0	0.00	
DQ687176	0	0.00	
DQ687198	0	0.00	
DQ687208	0	0.00	
DQ687226	0	0.00	
DQ687248	0	0.00	
DQ687290	0	0.00	
DQ687305	0	0.00	
DQ687328	0	0.00	
DQ687329	0	0.00	
DQ687334	0	0.00	
DQ687337	0	0.00	
DQ687339	0	0.00	
DQ687357	0	0.00	
DQ687367	0	0.00	
DQ687370	0	0.00	
DQ687373	0	0.00	
DQ687398	0	0.00	
DQ687481	0	0.00	
DQ687492	0	0.00	
DQ687495	0	0.00	
DQ687503	0	0.00	

DQ687525	0	0.00	
DQ687551	0	0.00	
DQ687563	0	0.00	
DQ687584	0	0.00	
DQ687587	0	0.00	
DQ687612	0	0.00	
DQ687613	0	0.00	
DQ687636	0	0.00	
DQ687643	0	0.00	
DQ687657	0	0.00	
DQ687658	0	0.00	
DQ687660	0	0.00	
DQ687665	0	0.00	
DQ687709	0	0.00	
DQ687711	0	0.00	
DQ687771	0	0.00	
DQ687774	0	0.00	
DQ687790	0	0.00	
DQ687803	0	0.00	
DQ687821	0	0.00	
DQ687830	0	0.00	
DQ687895	0	0.00	
DQ687902	0	0.00	
DQ687955	0	0.00	
DQ688019	0	0.00	
DQ688048	0	0.00	
DQ688065	0	0.00	
DQ688096	0	0.00	
DQ688098	0	0.00	
DQ688102	0	0.00	
DQ688114	0	0.00	
DQ688115	0	0.00	
DQ688124	0	0.00	
DQ688126	0	0.00	
DQ688142	0.3174	0.71	223.6%
DQ688151	0	0.00	
DQ688189	0	0.00	
DQ688193	0	0.00	
DQ688225	0	0.00	
DQ688231	0	0.00	
DQ688238	0	0.00	
DQ688254	0	0.00	
DQ688323	0	0.00	
DQ688327	0	0.00	
DQ688332	0	0.00	
DQ688341	0	0.00	
DQ688370	0	0.00	

DQ688388	0	0.00	
DQ688394	0	0.00	
DQ688408	0	0.00	
DQ688429	0	0.00	
DQ688435	0	0.00	
DQ688440	0	0.00	
DQ688473	0	0.00	
DQ688496	0	0.00	
DQ688503	0	0.00	
DQ688517	0	0.00	
DQ688556	0	0.00	
DQ688565	0	0.00	
DQ688608	0	0.00	
DQ688639	0	0.00	
DQ688640	0	0.00	
DQ688647	0	0.00	
DQ688664	0	0.00	
DQ688666	0	0.00	
DQ688679	0	0.00	
DQ688687	0	0.00	
DQ688694	0	0.00	
DQ688695	0	0.00	
DQ688703	0	0.00	
DQ688740	0	0.00	
DQ688757	0	0.00	
DQ688766	0	0.00	
DQ688826	0	0.00	
DQ688833	0	0.00	
DQ688834	0	0.00	
DQ688854	0	0.00	
DQ688862	0	0.00	
DQ688864	0	0.00	
DQ688882	0	0.00	
DQ688885	0	0.00	
DQ688913	0	0.00	
DQ688916	0	0.00	
DQ688935	0	0.00	
DQ688995	0	0.00	
DQ689040	0	0.00	
DQ689085	0	0.00	
DQ689096	0	0.00	
DQ689104	0	0.00	
DQ689110	0	0.00	
DQ689115	0	0.00	
DQ689126	0	0.00	
DQ689150	0	0.00	
DQ689242	0	0.00	

DQ689250	0	0.00	
DQ689332	0	0.00	
DQ689358	0	0.00	
DQ689436	0	0.00	
DQ689443	0	0.00	
DQ689451	0	0.00	
DQ689476	0	0.00	
DQ689480	0	0.00	
DQ689483	0	0.00	
DQ689488	0	0.00	
DQ689581	0	0.00	
DQ689593	0	0.00	
DQ689603	0	0.00	
DQ689607	0	0.00	
DQ689650	0	0.00	
DQ689656	0	0.00	
DQ689676	0	0.00	
DQ689677	0	0.00	
DQ689702	0	0.00	
DQ689725	0	0.00	
DQ689729	0	0.00	
DQ689754	0	0.00	
DQ689765	0	0.00	
DQ689766	0	0.00	
DQ689782	0	0.00	
DQ689802	0	0.00	
DQ689835	0	0.00	
DQ689856	0	0.00	
DQ689871	0	0.00	
DQ689873	0	0.00	
DQ689881	0	0.00	
DQ689904	0	0.00	
DQ689945	0	0.00	
DQ689956	0	0.00	
DQ689966	0	0.00	
DQ689974	0	0.00	
DQ689989	0	0.00	
DQ690034	0	0.00	
DQ690080	0	0.00	
DQ690155	0	0.00	
DQ690161	0	0.00	
DQ690190	0	0.00	
DQ690205	0	0.00	
DQ690206	0	0.00	
DQ690216	0	0.00	
DQ690220	0	0.00	
DQ690224	0	0.00	

DQ690284	0	0.00	
DQ690299	0	0.00	
DQ690311	0	0.00	
DQ690349	0	0.00	
DQ690373	0	0.00	
DQ690391	0	0.00	
DQ690425	0	0.00	
DQ690455	0	0.00	
DQ690487	0	0.00	
DQ690510	0	0.00	
DQ690514	0	0.00	
DQ690520	0	0.00	
DQ690536	0	0.00	
DQ690554	0	0.00	
DQ690559	0	0.00	
DQ690563	0	0.00	
DQ690574	0	0.00	
DQ690575	0	0.00	
DQ690615	0	0.00	
DQ690617	0	0.00	
DQ690622	0	0.00	
DQ690639	0	0.00	
DQ690641	0	0.00	
DQ690655	0	0.00	
DQ690676	0	0.00	
DQ690706	0	0.00	
DQ690714	0	0.00	
DQ690752	0	0.00	
DQ690769	0	0.00	
DQ690772	0	0.00	
DQ690809	0	0.00	
DQ690858	0	0.00	
DQ690904	0	0.00	
DQ690906	0	0.00	
DQ690931	0	0.00	
DQ690939	0	0.00	
DQ690982	0	0.00	
DQ691027	0	0.00	
DQ691051	0	0.00	
DQ691069	0	0.00	
DQ691074	0	0.00	
DQ691086	0	0.00	
DQ691089	0	0.00	
DQ691118	0	0.00	
DQ691150	0	0.00	
DQ691191	0	0.00	
DQ691236	0	0.00	

DQ691244	0	0.00	
DQ691258	0	0.00	
DQ691263	0	0.00	
DQ691281	0	0.00	
DQ691284	0	0.00	
DQ691295	0	0.00	
DQ691313	0	0.00	
DQ691347	0	0.00	
DQ691372	0	0.00	
DQ691381	0	0.00	
DQ691393	0	0.00	
DQ691398	0	0.00	
DQ691417	0	0.00	
DQ691421	0	0.00	
DQ691427	0	0.00	
DQ691435	0	0.00	
DQ691477	0	0.00	
DQ691498	0	0.00	
DQ691512	0	0.00	
DQ691522	0	0.00	
DQ691537	0	0.00	
DQ691565	0	0.00	
DQ691575	0	0.00	
DQ691590	0	0.00	
DQ691591	0	0.00	
DQ691614	0	0.00	
DQ691628	0	0.00	
DQ691629	0	0.00	
DQ691637	0	0.00	
DQ691656	0	0.00	
DQ691659	0	0.00	
DQ691666	0	0.00	
DQ691809	0	0.00	
DQ691824	0	0.00	
DQ691826	0	0.00	
DQ691839	0	0.00	
DQ691873	0	0.00	
DQ691876	0	0.00	
DQ691885	0	0.00	
DQ691918	0.292798	0.65	223.6%
DQ691942	0	0.00	
DQ691946	0	0.00	
DQ691962	0	0.00	
DQ692003	0	0.00	
DQ692005	0	0.00	
DQ692011	0	0.00	
DQ692040	0	0.00	

DQ692044	0	0.00	
DQ692052	0	0.00	
DQ692066	0	0.00	
DQ692070	0	0.00	
DQ692085	0	0.00	
DQ692087	0	0.00	
DQ692099	0	0.00	
DQ692123	0	0.00	
DQ692154	0	0.00	
DQ692163	0	0.00	
DQ692167	0	0.00	
DQ692176	0	0.00	
DQ692178	0	0.00	
DQ692221	0	0.00	
DQ692255	0	0.00	
DQ692282	0	0.00	
DQ692312	0	0.00	
DQ692328	0	0.00	
DQ692337	0	0.00	
DQ692342	0	0.00	
DQ692353	0	0.00	
DQ692361	0	0.00	
DQ692365	0	0.00	
DQ692371	0	0.00	
DQ692397	0	0.00	
DQ692402	0	0.00	
DQ692405	0	0.00	
DQ692414	0	0.00	
DQ692419	0	0.00	
DQ692422	0	0.00	
DQ692466	0	0.00	
DQ692484	0	0.00	
DQ692485	0	0.00	
DQ692500	0	0.00	
DQ692517	0	0.00	
DQ692519	0	0.00	
DQ692526	0	0.00	
DQ692542	0	0.00	
DQ692550	0	0.00	
DQ692567	0	0.00	
DQ692580	0	0.00	
DQ692601	0	0.00	
DQ692605	0	0.00	
DQ692606	0	0.00	
DQ692629	0	0.00	
DQ692632	0	0.00	
DQ692637	0	0.00	

DQ692648	0	0.00	
DQ692655	0	0.00	
DQ692658	0	0.00	
DQ692659	0	0.00	
DQ692674	0	0.00	
DQ692680	0	0.00	
DQ692714	0	0.00	
DQ692748	0	0.00	
DQ692750	0	0.00	
DQ692751	0	0.00	
DQ692767	0	0.00	
DQ692774	0	0.00	
DQ692785	0	0.00	
DQ692803	0	0.00	
DQ692804	0	0.00	
DQ692822	0	0.00	
DQ692840	0	0.00	
DQ692874	0	0.00	
DQ692889	0	0.00	
DQ692898	0	0.00	
DQ692926	0	0.00	
DQ692955	0	0.00	
DQ692983	0	0.00	
DQ693004	0	0.00	
DQ693016	0	0.00	
DQ693081	0	0.00	
DQ693128	0	0.00	
DQ693130	0	0.00	
DQ693132	0	0.00	
DQ693159	0	0.00	
DQ693219	0	0.00	
DQ693224	0	0.00	
DQ693233	0	0.00	
DQ693239	0	0.00	
DQ693245	0	0.00	
DQ693247	0	0.00	
DQ693283	0	0.00	
DQ693303	0	0.00	
DQ693330	0	0.00	
DQ693348	0	0.00	
DQ693362	0	0.00	
DQ693371	0	0.00	
DQ693385	0	0.00	
DQ693426	0	0.00	
DQ693432	0	0.00	
DQ693437	0	0.00	
DQ693451	0	0.00	

DQ693458	0	0.00	
DQ693466	0	0.00	
DQ693481	0	0.00	
DQ693536	0	0.00	
DQ693547	0	0.00	
DQ693555	0	0.00	
DQ693563	0	0.00	
DQ693585	0	0.00	
DQ693587	0	0.00	
DQ693599	0	0.00	
DQ693608	0	0.00	
DQ693615	0	0.00	
DQ693626	0	0.00	
DQ693681	0	0.00	
DQ693684	0	0.00	
DQ693697	0	0.00	
DQ693763	0	0.00	
DQ693766	0	0.00	
DQ693841	0	0.00	
DQ693890	0	0.00	
DQ693943	0	0.00	
DQ694023	0	0.00	
DQ694039	0	0.00	
DQ694045	0	0.00	
DQ694056	0	0.00	
DQ694073	0	0.00	
DQ694086	0	0.00	
DQ694120	0	0.00	
DQ694131	0	0.00	
DQ694138	0	0.00	
DQ694154	0	0.00	
DQ694167	0	0.00	
DQ694195	0	0.00	
DQ694222	0	0.00	
DQ694250	0	0.00	
DQ694251	0	0.00	
DQ694273	0	0.00	
DQ694302	0	0.00	
DQ694348	0	0.00	
DQ694442	0	0.00	
DQ694455	0	0.00	
DQ694462	0	0.00	
DQ694471	0	0.00	
DQ694503	0	0.00	
DQ694548	0	0.00	
DQ694553	0	0.00	
DQ694559	0	0.00	

DQ694571	0	0.00	
DQ694598	0	0.00	
DQ694599	0	0.00	
DQ694605	0	0.00	
DQ694625	0	0.00	
DQ694636	0	0.00	
DQ694656	0	0.00	
DQ694702	0	0.00	
DQ694705	0	0.00	
DQ694711	0	0.00	
DQ694726	0	0.00	
DQ694728	0	0.00	
DQ694760	0	0.00	
DQ694768	0	0.00	
DQ694780	0	0.00	
DQ694781	0	0.00	
DQ694814	0	0.00	
DQ694834	0	0.00	
DQ694838	0	0.00	
DQ694846	0	0.00	
DQ694852	0	0.00	
DQ694857	0	0.00	
DQ694887	0	0.00	
DQ694892	0	0.00	
DQ694903	0	0.00	
DQ694912	0	0.00	
DQ694932	0	0.00	
DQ694934	0	0.00	
DQ694944	0	0.00	
DQ694960	0	0.00	
DQ694964	0	0.00	
DQ695012	0	0.00	
DQ695013	0	0.00	
DQ695014	0	0.00	
DQ695017	0	0.00	
DQ695049	0	0.00	
DQ695053	0	0.00	
DQ695059	0	0.00	
DQ695082	0	0.00	
DQ695085	0	0.00	
DQ695092	0	0.00	
DQ695109	0	0.00	
DQ695125	0	0.00	
DQ695127	0	0.00	
DQ695156	0	0.00	
DQ695187	0	0.00	
DQ695197	0	0.00	

DQ695213	0	0.00	
DQ695223	0	0.00	
DQ695238	0	0.00	
DQ695242	0	0.00	
DQ695250	0	0.00	
DQ695285	0	0.00	
DQ695289	0	0.00	
DQ695326	0	0.00	
DQ695332	0	0.00	
DQ695344	0	0.00	
DQ695354	0	0.00	
DQ695356	0	0.00	
DQ695412	0	0.00	
DQ695450	0	0.00	
DQ695469	0	0.00	
DQ695529	0	0.00	
DQ695538	0	0.00	
DQ695567	0	0.00	
DQ695570	0	0.00	
DQ695612	0	0.00	
DQ695624	0	0.00	
DQ695667	0	0.00	
DQ695691	0	0.00	
DQ695717	0	0.00	
DQ695744	0	0.00	
DQ695761	0	0.00	
DQ695786	0	0.00	
DQ695801	0	0.00	
DQ695813	0	0.00	
DQ695827	0	0.00	
DQ695859	0	0.00	
DQ695881	0	0.00	
DQ695884	0	0.00	
DQ695909	0	0.00	
DQ695911	0	0.00	
DQ695947	0	0.00	
DQ695949	0	0.00	
DQ695960	0	0.00	
DQ695964	0	0.00	
DQ695991	0	0.00	
DQ696009	0	0.00	
DQ696026	0	0.00	
DQ696040	0	0.00	
DQ696046	0	0.00	
DQ696055	0	0.00	
DQ696073	0	0.00	
DQ696095	0	0.00	

DQ696105	0	0.00	
DQ696182	0	0.00	
DQ696186	0	0.00	
DQ696189	0	0.00	
DQ696198	0	0.00	
DQ696209	0	0.00	
DQ696253	0	0.00	
DQ696267	0	0.00	
DQ696282	0	0.00	
DQ696303	0	0.00	
DQ696325	0	0.00	
DQ696335	0	0.00	
DQ696348	0	0.00	
DQ696405	0	0.00	
DQ696417	0	0.00	
DQ696435	0	0.00	
DQ696446	0	0.00	
DQ696487	0	0.00	
DQ696544	0	0.00	
DQ696597	0	0.00	
DQ696615	0	0.00	
DQ696619	0	0.00	
DQ696623	0	0.00	
DQ696633	0	0.00	
DQ696666	0	0.00	
DQ696703	0	0.00	
DQ696725	0	0.00	
DQ696739	0	0.00	
DQ696806	0	0.00	
DQ696813	0	0.00	
DQ696815	0	0.00	
DQ696825	0	0.00	
DQ696832	0	0.00	
DQ696836	0	0.00	
DQ696837	0	0.00	
DQ696873	0	0.00	
DQ696923	0	0.00	
DQ696926	0	0.00	
DQ696941	0	0.00	
DQ696944	0	0.00	
DQ696976	0	0.00	
DQ697004	0	0.00	
DQ697005	0	0.00	
DQ697027	0	0.00	
DQ697053	0	0.00	
DQ697067	0	0.00	
DQ697070	0	0.00	

DQ697096	0.1561312	0.35	223.6%
DQ697120	0	0.00	
DQ697124	0	0.00	
DQ697151	0	0.00	
DQ697183	0	0.00	
DQ697222	0	0.00	
DQ697233	0	0.00	
DQ697253	0	0.00	
DQ697298	0	0.00	
DQ697303	0	0.00	
DQ697314	0	0.00	
DQ697332	0	0.00	
DQ697348	0	0.00	
DQ697364	0	0.00	
DQ697366	0	0.00	
DQ697370	0	0.00	
DQ697373	0	0.00	
DQ697403	0	0.00	
DQ697422	0	0.00	
DQ697472	0	0.00	
DQ697475	0	0.00	
DQ697489	0	0.00	
DQ697495	0	0.00	
DQ697509	0	0.00	
DQ697538	0	0.00	
DQ697567	0	0.00	
DQ697578	0	0.00	
DQ697620	0	0.00	
DQ697642	0	0.00	
DQ697673	0	0.00	
DQ697701	0	0.00	
DQ697726	0	0.00	
DQ697752	0	0.00	
DQ697774	0	0.00	
DQ697836	0	0.00	
DQ697848	0	0.00	
DQ697893	0	0.00	
DQ697912	0	0.00	
DQ697944	0	0.00	
DQ697946	0	0.00	
DQ697988	0	0.00	
DQ697992	0	0.00	
DQ698005	0	0.00	
DQ698039	0	0.00	
DQ698049	0	0.00	
DQ698060	0	0.00	
DQ698080	0	0.00	

DQ698081	0	0.00	
DQ698136	0	0.00	
DQ698138	0	0.00	
DQ698173	0	0.00	
DQ698179	0	0.00	
DQ698182	0	0.00	
DQ698221	0	0.00	
DQ698253	0	0.00	
DQ698272	0	0.00	
DQ698276	0	0.00	
DQ698328	0	0.00	
DQ698341	0	0.00	
DQ698355	0	0.00	
DQ698370	0	0.00	
DQ698417	0	0.00	
DQ698427	0	0.00	
DQ698435	0	0.00	
DQ698459	0	0.00	
DQ698460	0	0.00	
DQ698463	0	0.00	
DQ698470	0	0.00	
DQ698474	0	0.00	
DQ698476	0	0.00	
DQ698478	0	0.00	
DQ698532	0	0.00	
DQ698553	0	0.00	
DQ698559	0	0.00	
DQ698563	0	0.00	
DQ698574	0	0.00	
DQ698583	0	0.00	
DQ698589	0	0.00	
DQ698598	0	0.00	
DQ698602	0	0.00	
DQ698625	0	0.00	
DQ698688	0	0.00	
DQ698692	0	0.00	
DQ698722	0	0.00	
DQ698748	0	0.00	
DQ698749	0	0.00	
DQ698755	0	0.00	
DQ698761	0	0.00	
DQ698767	0	0.00	
DQ698772	0	0.00	
DQ698791	0	0.00	
DQ698807	0	0.00	
DQ698829	0	0.00	
DQ698838	0	0.00	

DQ698848	0	0.00	
DQ698861	0	0.00	
DQ698879	0	0.00	
DQ698885	0	0.00	
DQ698894	0	0.00	
DQ698901	0	0.00	
DQ698911	0	0.00	
DQ698920	0	0.00	
DQ698970	0	0.00	
DQ699063	0	0.00	
DQ699068	0	0.00	
DQ699081	0	0.00	
DQ699108	0	0.00	
DQ699135	0	0.00	
DQ699139	0	0.00	
DQ699161	0	0.00	
DQ699237	0	0.00	
DQ699248	0	0.00	
DQ699275	0	0.00	
DQ699288	0	0.00	
DQ699365	0	0.00	
DQ699366	0	0.00	
DQ699373	0	0.00	
DQ699395	0	0.00	
DQ699411	0	0.00	
DQ699481	0	0.00	
DQ699523	1.0616182	0.25	23.3%
DQ699575	0	0.00	
DQ699636	0	0.00	
DQ699669	0	0.00	
DQ699675	0	0.00	
DQ699682	0	0.00	
DQ699684	0	0.00	
DQ699688	0	0.00	
DQ699700	0	0.00	
DQ699808	0	0.00	
DQ699817	0	0.00	
DQ699830	0	0.00	
DQ699833	0	0.00	
DQ699844	0	0.00	
DQ699883	0	0.00	
DQ699907	0	0.00	
DQ699918	0	0.00	
DQ699921	0	0.00	
DQ699928	0	0.00	
DQ699940	0	0.00	
DQ699947	0	0.00	

DQ699981	0	0.00	
DQ699990	0	0.00	
DQ700012	0	0.00	
DQ700023	0	0.00	
DQ700040	0	0.00	
DQ700075	0	0.00	
DQ700079	0	0.00	
DQ700103	0	0.00	
DQ700114	0	0.00	
DQ700154	0	0.00	
DQ700184	0	0.00	
DQ700189	0	0.00	
DQ700265	0	0.00	
DQ700318	0	0.00	
DQ700325	0	0.00	
DQ700364	0	0.00	
DQ700365	0	0.00	
DQ700366	0	0.00	
DQ700372	0	0.00	
DQ700379	0	0.00	
DQ700388	0	0.00	
DQ700390	0	0.00	
DQ700395	0	0.00	
DQ700417	0	0.00	
DQ700421	0	0.00	
DQ700448	0	0.00	
DQ700505	0	0.00	
DQ700514	0	0.00	
DQ700517	0	0.00	
DQ700539	0	0.00	
DQ700541	0	0.00	
DQ700559	0	0.00	
DQ700565	0	0.00	
DQ700577	0	0.00	
DQ700581	0	0.00	
DQ700641	0	0.00	
DQ700643	0	0.00	
DQ700655	0	0.00	
DQ700691	0	0.00	
DQ700738	0	0.00	
DQ700748	0	0.00	
DQ700818	0	0.00	
DQ700826	0	0.00	
DQ700852	0	0.00	
DQ700866	0	0.00	
DQ700870	0	0.00	
DQ700891	0	0.00	

DQ700900	0	0.00	
DQ700905	0	0.00	
DQ700909	0	0.00	
DQ700921	0	0.00	
DQ700977	0	0.00	
DQ700980	0	0.00	
DQ701001	0	0.00	
DQ701015	0	0.00	
DQ701047	0	0.00	
DQ701068	0	0.00	
DQ701088	0	0.00	
DQ701108	0	0.00	
DQ701143	0	0.00	
DQ701172	0	0.00	
DQ701178	0	0.00	
DQ701201	0	0.00	
DQ701251	0	0.00	
DQ701279	0	0.00	
DQ701288	0	0.00	
DQ701289	0	0.00	
DQ701296	0	0.00	
DQ701325	0	0.00	
DQ701380	0	0.00	
DQ701393	0	0.00	
DQ701397	0	0.00	
DQ701432	0	0.00	
DQ701434	0	0.00	
DQ701447	0	0.00	
DQ701448	0	0.00	
DQ701498	0	0.00	
DQ701507	0	0.00	
DQ701531	0	0.00	
DQ701542	0	0.00	
DQ701554	0	0.00	
DQ701556	0	0.00	
DQ701600	0	0.00	
DQ701617	0	0.00	
DQ701642	0	0.00	
DQ701654	0	0.00	
DQ701692	0	0.00	
DQ701698	0	0.00	
DQ701744	0	0.00	
DQ701777	0	0.00	
DQ701795	0	0.00	
DQ701826	0	0.00	
DQ701829	0	0.00	
DQ701864	0	0.00	

DQ701874	0	0.00	
DQ701885	0	0.00	
DQ701901	0	0.00	
DQ701953	0	0.00	
DQ701960	0	0.00	
DQ701973	0	0.00	
DQ701975	0	0.00	
DQ701977	0	0.00	
DQ702018	0	0.00	
DQ702089	0	0.00	
DQ702092	0	0.00	
DQ702100	0	0.00	
DQ702112	0	0.00	
DQ702163	0	0.00	
DQ702193	0	0.00	
DQ702206	0	0.00	
DQ702273	0	0.00	
DQ702305	0	0.00	
DQ702357	0	0.00	
DQ702367	0	0.00	
DQ702372	0	0.00	
DQ702374	0	0.00	
DQ702375	0	0.00	
DQ702386	0	0.00	
DQ702426	0	0.00	
DQ702453	0	0.00	
DQ702458	0	0.00	
DQ702468	0	0.00	
DQ702483	0	0.00	
DQ702512	0	0.00	
DQ702516	0	0.00	
DQ702540	0	0.00	
DQ702558	0	0.00	
DQ702571	0	0.00	
DQ702572	0	0.00	
DQ702575	0	0.00	
DQ702585	0	0.00	
DQ702589	0	0.00	
DQ702609	0	0.00	
DQ702610	0	0.00	
DQ702619	0	0.00	
DQ702678	0	0.00	
DQ702689	0	0.00	
DQ702697	0	0.00	
DQ702754	0	0.00	
DQ702757	0	0.00	
DQ702783	0	0.00	

DQ702854	0	0.00	
DQ702868	0	0.00	
DQ702910	0	0.00	
DQ702916	0	0.00	
DQ702922	0	0.00	
DQ702961	0	0.00	
DQ702968	0	0.00	
DQ702976	0	0.00	
DQ702978	0	0.00	
DQ703000	0	0.00	
DQ703005	0	0.00	
DQ703017	0	0.00	
DQ703018	0	0.00	
DQ703023	0	0.00	
DQ703049	0	0.00	
DQ703057	0	0.00	
DQ703064	0	0.00	
DQ703065	0	0.00	
DQ703082	0	0.00	
DQ703105	0	0.00	
DQ703168	0	0.00	
DQ703267	0	0.00	
DQ703299	0	0.00	
DQ703302	0	0.00	
DQ703331	0	0.00	
DQ703338	0	0.00	
DQ703340	0	0.00	
DQ703376	0	0.00	
DQ703386	0	0.00	
DQ703407	0	0.00	
DQ703419	0	0.00	
DQ703520	0	0.00	
DQ703566	11.97146	0.61	5.1%
DQ703578	0	0.00	
DQ703591	0	0.00	
DQ703595	0	0.00	
DQ703598	0	0.00	
DQ703605	0	0.00	
DQ703657	0	0.00	
DQ703690	0	0.00	
DQ703701	0	0.00	
DQ703766	0	0.00	
DQ703862	0	0.00	
DQ703878	0	0.00	
DQ703907	0	0.00	
DQ703913	0	0.00	
DQ703915	0	0.00	

DQ703942	0	0.00	
DQ703943	0	0.00	
DQ703967	0	0.00	
DQ703976	0	0.00	
DQ703994	0	0.00	
DQ704010	0	0.00	
DQ704011	0	0.00	
DQ704037	0	0.00	
DQ704048	0	0.00	
DQ704059	0	0.00	
DQ704065	0	0.00	
DQ704074	0	0.00	
DQ704082	0	0.00	
DQ704103	0	0.00	
DQ704143	0	0.00	
DQ704149	0	0.00	
DQ704153	0	0.00	
DQ704177	0	0.00	
DQ704230	0	0.00	
DQ704256	0	0.00	
DQ704262	0	0.00	
DQ704275	0	0.00	
DQ704289	0	0.00	
DQ704356	0	0.00	
DQ704374	0	0.00	
DQ704415	0	0.00	
DQ704437	0	0.00	
DQ704442	0	0.00	
DQ704447	0	0.00	
DQ704454	0	0.00	
DQ704470	0	0.00	
DQ704514	0	0.00	
DQ704526	0	0.00	
DQ704530	0	0.00	
DQ704550	0	0.00	
DQ704553	0	0.00	
DQ704574	0	0.00	
DQ704580	0	0.00	
DQ704607	0	0.00	
DQ704627	0	0.00	
DQ704629	0	0.00	
DQ704650	0	0.00	
DQ704652	0	0.00	
DQ704661	0	0.00	
DQ704682	0	0.00	
DQ704690	0	0.00	
DQ704723	0	0.00	

DQ704729	0	0.00	
DQ704761	0	0.00	
DQ704763	0	0.00	
DQ704796	0	0.00	
DQ704807	0	0.00	
DQ704809	0	0.00	
DQ704816	0	0.00	
DQ704842	0	0.00	
DQ704849	0	0.00	
DQ704887	0	0.00	
DQ704895	0	0.00	
DQ704903	0	0.00	
DQ704925	0	0.00	
DQ704975	0	0.00	
DQ705008	0	0.00	
DQ705017	0	0.00	
DQ705021	0	0.00	
DQ705033	0	0.00	
DQ705035	0	0.00	
DQ705092	0	0.00	
DQ705113	0	0.00	
DQ705124	0	0.00	
DQ705130	0	0.00	
DQ705171	0	0.00	
DQ705175	0	0.00	
DQ705200	0	0.00	
DQ705208	0	0.00	
DQ705218	0	0.00	
DQ705222	0	0.00	
DQ705271	0	0.00	
DQ705335	0	0.00	
DQ705359	0	0.00	
DQ705419	0	0.00	
DQ705435	0	0.00	
DQ705523	0	0.00	
DQ705537	0	0.00	
DQ705542	0	0.00	
DQ705564	0	0.00	
DQ705566	0	0.00	
DQ705597	0	0.00	
DQ705620	0	0.00	
DQ705640	0	0.00	
DQ705642	0	0.00	
DQ705652	0	0.00	
DQ705702	0	0.00	
DQ705726	0	0.00	
DQ705727	0	0.00	

DQ705769	0	0.00	
DQ705824	0	0.00	
DQ705847	0	0.00	
DQ705849	0	0.00	
DQ705860	0	0.00	
DQ705864	0	0.00	
DQ705865	0	0.00	
DQ705881	0	0.00	
DQ705886	0	0.00	
DQ705896	0	0.00	
DQ705898	0	0.00	
DQ705907	0	0.00	
DQ705922	0	0.00	
DQ705927	0	0.00	
DQ705933	0	0.00	
DQ706009	0	0.00	
DQ706020	0	0.00	
DQ706036	0	0.00	
DQ706049	0	0.00	
DQ706062	0	0.00	
DQ706069	0	0.00	
DQ706101	0	0.00	
DQ706115	0	0.00	
DQ706116	0	0.00	
DQ706140	0	0.00	
DQ706153	0	0.00	
DQ706172	0	0.00	
DQ706175	0	0.00	
DQ706184	0	0.00	
DQ706185	0	0.00	
DQ706219	0	0.00	
DQ706243	0	0.00	
DQ706266	0	0.00	
DQ706333	0	0.00	
DQ706335	0	0.00	
DQ706336	0	0.00	
DQ706369	0	0.00	
DQ706373	0	0.00	
DQ706374	0	0.00	
DQ706406	0	0.00	
DQ706458	0	0.00	
DQ706459	0	0.00	
DQ706488	0	0.00	
DQ706494	0	0.00	
DQ706499	0	0.00	
DQ706509	0	0.00	
DQ706510	0	0.00	

DQ706519	0	0.00	
DQ706549	0	0.00	
DQ706580	0	0.00	
DQ706582	0	0.00	
DQ706644	0	0.00	
DQ706648	0	0.00	
DQ706703	0	0.00	
DQ706720	0	0.00	
DQ706722	0	0.00	
DQ706843	0	0.00	
DQ706864	0	0.00	
DQ706890	0	0.00	
DQ706910	0	0.00	
DQ706931	0	0.00	
DQ706969	0	0.00	
DQ706974	0	0.00	
DQ706995	0	0.00	
DQ707012	0	0.00	
DQ707019	0	0.00	
DQ707024	0	0.00	
DQ707045	0	0.00	
DQ707051	0	0.00	
DQ707078	0	0.00	
DQ707179	0	0.00	
DQ707199	0	0.00	
DQ707201	0	0.00	
DQ707213	0	0.00	
DQ707224	0	0.00	
DQ707240	0	0.00	
DQ707248	0	0.00	
DQ707250	0	0.00	
DQ707267	0	0.00	
DQ707271	0	0.00	
DQ707295	0	0.00	
DQ707303	0	0.00	
DQ707315	0	0.00	
DQ707320	0	0.00	
DQ707326	0	0.00	
DQ707327	0	0.00	
DQ707353	0	0.00	
DQ707380	0	0.00	
DQ707400	0	0.00	
DQ707403	0	0.00	
DQ707409	0	0.00	
DQ707432	0	0.00	
DQ707450	0	0.00	
DQ707477	0	0.00	

DQ707526	0	0.00	
DQ707556	0	0.00	
DQ707564	0	0.00	
DQ707569	0	0.00	
DQ707575	0	0.00	
DQ707587	0	0.00	
DQ707596	0	0.00	
DQ707628	0	0.00	
DQ707640	0	0.00	
DQ707670	0	0.00	
DQ707675	0	0.00	
DQ707678	0	0.00	
DQ707708	0	0.00	
DQ707709	0	0.00	
DQ707711	0	0.00	
DQ707714	0	0.00	
DQ707749	0	0.00	
DQ707763	0	0.00	
DQ707789	0	0.00	
DQ707872	0	0.00	
DQ707882	0	0.00	
DQ707901	0	0.00	
DQ707909	0	0.00	
DQ707913	0	0.00	
DQ707915	0	0.00	
DQ707920	0	0.00	
DQ707969	0	0.00	
DQ707980	0	0.00	
DQ708001	0	0.00	
DQ708007	0	0.00	
DQ708019	0	0.00	
DQ708036	0	0.00	
DQ708063	0	0.00	
DQ708067	0	0.00	
DQ708075	0	0.00	
DQ708091	0	0.00	
DQ708189	0	0.00	
DQ708201	0	0.00	
DQ708205	0	0.00	
DQ708234	0	0.00	
DQ708250	0	0.00	
DQ708288	0	0.00	
DQ708292	0	0.00	
DQ708300	0	0.00	
DQ708318	0	0.00	
DQ708335	0	0.00	
DQ708345	0	0.00	

DQ708362	0	0.00	
DQ708409	0	0.00	
DQ708450	0	0.00	
DQ708476	0	0.00	
DQ708492	0	0.00	
DQ708497	0	0.00	
DQ708505	0	0.00	
DQ708512	0	0.00	
DQ708570	0	0.00	
DQ708574	0	0.00	
DQ708589	0	0.00	
DQ708637	0	0.00	
DQ708654	0	0.00	
DQ708683	0	0.00	
DQ708686	0	0.00	
DQ708697	0	0.00	
DQ708698	0	0.00	
DQ708713	0	0.00	
DQ708714	0	0.00	
DQ708719	0	0.00	
DQ708724	0	0.00	
DQ708735	0	0.00	
DQ708760	0	0.00	
DQ708778	0	0.00	
DQ708782	0	0.00	
DQ708858	0	0.00	
DQ708880	0	0.00	
DQ708892	0	0.00	
DQ708916	0	0.00	
DQ708924	38.09332	2.10	5.5%
DQ708946	0	0.00	
DQ708963	0	0.00	
DQ708973	0	0.00	
DQ708976	0	0.00	
DQ708984	0	0.00	
DQ708993	0	0.00	
DQ709019	0	0.00	
DQ709035	0	0.00	
DQ709048	0	0.00	
DQ709050	0	0.00	
DQ709054	0	0.00	
DQ709101	0	0.00	
DQ709102	0	0.00	
DQ709133	0	0.00	
DQ709135	0	0.00	
DQ709154	0	0.00	
DQ709165	0	0.00	

DQ709175	0	0.00	
DQ709177	0	0.00	
DQ709199	0	0.00	
DQ709229	0	0.00	
DQ709247	0	0.00	
DQ709308	0	0.00	
DQ709329	0	0.00	
DQ709334	0	0.00	
DQ709345	0	0.00	
DQ709401	0	0.00	
DQ709404	0	0.00	
DQ709433	0	0.00	
DQ709488	0	0.00	
DQ709491	0	0.00	
DQ709495	0	0.00	
DQ709498	0	0.00	
DQ709520	0	0.00	
DQ709549	0	0.00	
DQ709581	0	0.00	
DQ709607	0	0.00	
DQ709610	0	0.00	
DQ709611	0	0.00	
DQ709618	0	0.00	
DQ709620	0	0.00	
DQ709682	0	0.00	
DQ709686	0	0.00	
DQ709694	0	0.00	
DQ709708	0	0.00	
DQ709712	0	0.00	
DQ709738	0	0.00	
DQ709752	0	0.00	
DQ709755	0	0.00	
DQ709773	0	0.00	
DQ709778	0	0.00	
DQ709965	0	0.00	
DQ709984	0	0.00	
DQ709996	0	0.00	
DQ710007	0	0.00	
DQ710034	0	0.00	
DQ710056	0	0.00	
DQ710076	0	0.00	
DQ710118	0	0.00	
DQ710126	0	0.00	
DQ710144	0	0.00	
DQ710167	0	0.00	
DQ710175	0	0.00	
DQ710195	0	0.00	

DQ710218	0	0.00	
DQ710249	0	0.00	
DQ710257	0	0.00	
DQ710318	0	0.00	
DQ710326	0	0.00	
DQ710343	0	0.00	
DQ710344	0	0.00	
DQ710350	0	0.00	
DQ710363	0	0.00	
DQ710385	0	0.00	
DQ710427	0	0.00	
DQ710443	0	0.00	
DQ710451	0	0.00	
DQ710533	0	0.00	
DQ710605	0	0.00	
DQ710611	0	0.00	
DQ710622	0	0.00	
DQ710663	0	0.00	
DQ710664	0	0.00	
DQ710678	0	0.00	
DQ710713	0	0.00	
DQ710722	0	0.00	
DQ710745	0	0.00	
DQ710769	0	0.00	
DQ710791	0	0.00	
DQ710812	0	0.00	
DQ710814	0	0.00	
DQ710816	0	0.00	
DQ710826	0	0.00	
DQ710884	0	0.00	
DQ710894	0	0.00	
DQ710901	0	0.00	
DQ710937	0	0.00	
DQ710940	0	0.00	
DQ710955	0	0.00	
DQ711019	0.8608106	0.32	37.2%
DQ711072	0	0.00	
DQ711089	0	0.00	
DQ711111	0	0.00	
DQ711149	0	0.00	
DQ711159	0	0.00	
DQ711162	0	0.00	
DQ711171	0	0.00	
DQ711172	0	0.00	
DQ711183	0	0.00	
DQ711199	0	0.00	
DQ711212	0	0.00	

DQ711233	0	0.00	
DQ711256	0	0.00	
DQ711269	0	0.00	
DQ711271	0	0.00	
DQ711282	0	0.00	
DQ711310	0	0.00	
DQ711315	0	0.00	
DQ711333	0	0.00	
DQ711353	0	0.00	
DQ711354	0	0.00	
DQ711387	0	0.00	
DQ711393	0	0.00	
DQ711460	0	0.00	
DQ711473	0	0.00	
DQ711486	0	0.00	
DQ711501	0	0.00	
DQ711508	0	0.00	
DQ711509	0	0.00	
DQ711515	0	0.00	
DQ711524	0	0.00	
DQ711529	0	0.00	
DQ711544	0	0.00	
DQ711552	0	0.00	
DQ711560	0	0.00	
DQ711584	0	0.00	
DQ711604	0	0.00	
DQ711633	0	0.00	
DQ711646	0	0.00	
DQ711659	0	0.00	
DQ711691	0	0.00	
DQ711745	0	0.00	
DQ711818	0	0.00	
DQ711851	0	0.00	
DQ711897	0	0.00	
DQ711916	0	0.00	
DQ711917	0	0.00	
DQ711921	0	0.00	
DQ711929	0	0.00	
DQ711953	2.358482	0.16	6.6%
DQ711975	0	0.00	
DQ712012	0	0.00	
DQ712046	0	0.00	
DQ712055	0	0.00	
DQ712071	0	0.00	
DQ712092	0	0.00	
DQ712111	0	0.00	
DQ712122	0	0.00	

DQ712160	0	0.00	
DQ712179	0	0.00	
DQ712184	0	0.00	
DQ712213	0	0.00	
DQ712214	0	0.00	
DQ712273	0	0.00	
DQ712296	0	0.00	
DQ712343	0	0.00	
DQ712360	0	0.00	
DQ712368	0	0.00	
DQ712376	0	0.00	
DQ712402	0	0.00	
DQ712420	0	0.00	
DQ712497	0	0.00	
DQ712503	0	0.00	
DQ712520	0	0.00	
DQ712548	0	0.00	
DQ712556	3.600364	0.33	9.1%
DQ712560	0	0.00	
DQ712603	0	0.00	
DQ712628	0	0.00	
DQ712774	0	0.00	
DQ712777	0	0.00	
DQ712790	0	0.00	
DQ712794	0	0.00	
DQ712803	0	0.00	
DQ712809	0	0.00	
DQ712810	0	0.00	
DQ712878	0	0.00	
DQ712887	0	0.00	
DQ712890	0	0.00	
DQ712916	0	0.00	
DQ712917	0	0.00	
DQ712936	0	0.00	
DQ712948	0	0.00	
DQ713008	0	0.00	
DQ713015	0	0.00	
DQ713034	0	0.00	
DQ713064	0	0.00	
DQ713076	0	0.00	
DQ713079	0	0.00	
DQ713163	0	0.00	
DQ713197	0	0.00	
DQ713227	0	0.00	
DQ713276	0	0.00	
DQ713287	0	0.00	
DQ713310	0	0.00	

DQ713313	0	0.00	
DQ713375	0	0.00	
DQ713407	0	0.00	
DQ713414	0	0.00	
DQ713442	0	0.00	
DQ713456	0	0.00	
DQ713459	0	0.00	
DQ713469	0	0.00	
DQ713505	0	0.00	
DQ713519	0	0.00	
DQ713526	0	0.00	
DQ713539	0	0.00	
DQ713546	0	0.00	
DQ713554	0	0.00	
DQ713582	0	0.00	
DQ713604	0	0.00	
DQ713653	0	0.00	
DQ713654	0	0.00	
DQ713678	0	0.00	
DQ713701	0	0.00	
DQ713721	0	0.00	
DQ713732	0	0.00	
DQ713814	0	0.00	
DQ713846	0	0.00	
DQ713888	0	0.00	
DQ713954	0	0.00	
DQ713958	0	0.00	
DQ713959	0	0.00	
DQ713967	0	0.00	
DQ713989	0	0.00	
DQ714076	0	0.00	
DQ714108	0	0.00	
DQ714171	0	0.00	
DQ714183	0	0.00	
DQ714193	0	0.00	
DQ714220	0	0.00	
DQ714222	0	0.00	
DQ714285	0	0.00	
DQ714298	0	0.00	
DQ714309	0	0.00	
DQ714346	0	0.00	
DQ714356	0	0.00	
DQ714381	0	0.00	
DQ714398	0	0.00	
DQ714413	0	0.00	
DQ714420	0	0.00	
DQ714446	0	0.00	

DQ714447	0	0.00	
DQ714455	0	0.00	
DQ714499	0	0.00	
DQ714525	0	0.00	
DQ714579	0	0.00	
DQ714600	0	0.00	
DQ714612	0	0.00	
DQ714635	0	0.00	
DQ714645	0	0.00	
DQ714646	0	0.00	
DQ714658	0	0.00	
DQ714716	0	0.00	
DQ714762	0	0.00	
DQ714775	0	0.00	
DQ714790	0	0.00	
DQ714799	0	0.00	
DQ714803	0	0.00	
DQ714811	0	0.00	
DQ714826	0	0.00	
DQ714852	0	0.00	
DQ714866	0	0.00	
DQ714868	0	0.00	
DQ714893	0	0.00	
DQ714940	0	0.00	
DQ714985	0	0.00	
DQ715046	0	0.00	
DQ715063	0	0.00	
DQ715077	0	0.00	
DQ715099	0	0.00	
DQ715157	0	0.00	
DQ715174	0	0.00	
DQ715195	0	0.00	
DQ715231	0	0.00	
DQ715236	0	0.00	
DQ715243	0	0.00	
DQ715268	0	0.00	
DQ715271	0	0.00	
DQ715292	0	0.00	
DQ715309	0	0.00	
DQ715325	0	0.00	
DQ715333	0	0.00	
DQ715352	0	0.00	
DQ715402	0	0.00	
DQ715404	0	0.00	
DQ715410	0	0.00	
DQ715413	0	0.00	
DQ715421	0	0.00	

DQ715424	0	0.00	
DQ715437	0	0.00	
DQ715484	0	0.00	
DQ715509	0	0.00	
DQ715510	0	0.00	
DQ715521	0	0.00	
DQ715522	0	0.00	
DQ715551	0	0.00	
DQ715562	0	0.00	
DQ715600	0	0.00	
DQ715649	0	0.00	
DQ715667	30.58754	2.60	8.5%
DQ715668	0	0.00	
DQ715729	0.12547992	0.05	38.7%
DQ715731	0	0.00	
DQ715735	0	0.00	
DQ715767	0	0.00	
DQ715779	0	0.00	
DQ715789	0	0.00	
DQ715816	0	0.00	
DQ715827	0	0.00	
DQ715831	0	0.00	
DQ715854	0	0.00	
DQ715856	0	0.00	
DQ715862	0	0.00	
DQ715869	0	0.00	
DQ715910	0	0.00	
DQ715911	0	0.00	
DQ715921	0	0.00	
DQ715968	0	0.00	
DQ715973	17.10406	0.60	3.5%
DQ716012	0	0.00	
DQ716014	0	0.00	
DQ716022	0	0.00	
DQ716029	0	0.00	
DQ716032	0	0.00	
DQ716085	0	0.00	
DQ716099	0	0.00	
DQ716102	0	0.00	
DQ716115	0	0.00	
DQ716137	0	0.00	
DQ716191	0	0.00	
DQ716236	0	0.00	
DQ716259	0	0.00	
DQ716264	0	0.00	
DQ716288	0	0.00	
DQ716308	0	0.00	

DQ716338	0	0.00	
DQ716352	0	0.00	
DQ716374	0	0.00	
DQ716375	0	0.00	
DQ716404	0	0.00	
DQ716425	0	0.00	
DQ716436	12.6241	0.78	6.1%
DQ716443	0	0.00	
DQ716500	0	0.00	
DQ716522	0	0.00	
DQ716540	0	0.00	
DQ716549	0	0.00	
DQ716579	0	0.00	
DQ716625	0	0.00	
DQ716653	0	0.00	
DQ716672	0	0.00	
DQ716682	0	0.00	
DQ716692	0	0.00	
DQ716699	0	0.00	
DQ716700	0	0.00	
DQ716701	0	0.00	
DQ716714	0	0.00	
DQ716732	0	0.00	
DQ716740	0	0.00	
DQ716783	0	0.00	
DQ716785	0	0.00	
DQ716800	0	0.00	
DQ716835	0	0.00	
DQ716898	0	0.00	
DQ716920	0	0.00	
DQ716963	0	0.00	
DQ716966	0	0.00	
DQ716981	0	0.00	
DQ717001	0	0.00	
DQ717002	0	0.00	
DQ717005	0	0.00	
DQ717033	0	0.00	
DQ717037	0	0.00	
DQ717047	0	0.00	
DQ717065	0	0.00	
DQ717083	0	0.00	
DQ717093	0	0.00	
DQ717113	0	0.00	
DQ717116	0	0.00	
DQ717142	0	0.00	
DQ717161	0	0.00	
DQ717291	0	0.00	

DQ717301	0	0.00	
DQ717333	0	0.00	
DQ717340	0	0.00	
DQ717357	0	0.00	
DQ717383	0	0.00	
DQ717414	0	0.00	
DQ717522	0	0.00	
DQ717549	0	0.00	
DQ717559	0	0.00	
DQ717560	0	0.00	
DQ717569	0	0.00	
DQ717580	0	0.00	
DQ717620	0	0.00	
DQ717630	0	0.00	
DQ717633	0	0.00	
DQ717665	0	0.00	
DQ717692	0	0.00	
DQ717693	0	0.00	
DQ717704	0	0.00	
DQ717768	0	0.00	
DQ717824	0	0.00	
DQ717831	0	0.00	
DQ717863	0	0.00	
DQ717868	0	0.00	
DQ717871	0	0.00	
DQ717888	0.901372	0.08	8.6%
DQ717895	0	0.00	
DQ717907	0	0.00	
DQ717915	0	0.00	
DQ717919	0	0.00	
DQ717944	0	0.00	
DQ717961	0	0.00	
DQ717970	0	0.00	
DQ717978	0	0.00	
DQ717983	0	0.00	
DQ717984	0	0.00	
DQ717988	0	0.00	
DQ718008	0	0.00	
DQ718012	0	0.00	
DQ718033	0	0.00	
DQ718113	0	0.00	
DQ718121	0	0.00	
DQ718144	0	0.00	
DQ718145	0	0.00	
DQ718173	0	0.00	
DQ718202	0	0.00	
DQ718207	0	0.00	

DQ718216	0	0.00	
DQ718221	0	0.00	
DQ718229	0	0.00	
DQ718260	0	0.00	
DQ718281	0	0.00	
DQ718285	0	0.00	
DQ718296	0	0.00	
DQ718309	0	0.00	
DQ718368	0	0.00	
DQ718380	0	0.00	
DQ718403	0	0.00	
DQ718421	0	0.00	
DQ718451	0	0.00	
DQ718500	0	0.00	
DQ718505	0	0.00	
DQ718522	0	0.00	
DQ718537	0.306996	0.69	223.6%
DQ718556	0	0.00	
DQ718565	0	0.00	
DQ718591	0	0.00	
DQ718612	0	0.00	
DQ718635	0	0.00	
DQ718672	0	0.00	
DQ718673	0	0.00	
DQ718704	0	0.00	
DQ718749	0	0.00	
DQ718759	0	0.00	
DQ718766	1.1589004	0.78	67.6%
DQ718767	0	0.00	
DQ718776	0	0.00	
DQ718785	0	0.00	
DQ718809	0	0.00	
DQ718811	0	0.00	
DQ718858	0	0.00	
DQ718921	0	0.00	
DQ718970	0	0.00	
DQ718974	0	0.00	
DQ719003	0	0.00	
DQ719008	0	0.00	
DQ719134	0	0.00	
DQ719139	0	0.00	
DQ719232	0	0.00	
DQ719254	0	0.00	
DQ719264	0	0.00	
DQ719269	0	0.00	
DQ719293	0	0.00	
DQ719298	0	0.00	

DQ719336	0	0.00	
DQ719367	0	0.00	
DQ719396	0	0.00	
DQ719401	0	0.00	
DQ719409	0	0.00	
DQ719413	0	0.00	
DQ719430	0	0.00	
DQ719437	0	0.00	
DQ719456	0	0.00	
DQ719459	0	0.00	
DQ719499	0	0.00	
DQ719504	0	0.00	
DQ719517	0	0.00	
DQ719525	0	0.00	
DQ719554	0	0.00	
DQ719555	0	0.00	
DQ719561	0	0.00	
DQ719573	0	0.00	
DQ719581	0	0.00	
DQ719582	0	0.00	
DQ719585	0	0.00	
DQ719602	0	0.00	
DQ719622	0	0.00	
DQ719678	0	0.00	
DQ719681	0	0.00	
DQ719700	0	0.00	
DQ719729	0	0.00	
DQ719737	0	0.00	
DQ719770	0	0.00	
DQ719819	0	0.00	
DQ719824	0	0.00	
DQ719838	0	0.00	
DQ719872	0	0.00	
DQ719888	0	0.00	
DQ719893	0	0.00	
DQ719911	0	0.00	
DQ719961	0	0.00	
DQ719983	0	0.00	
DQ720021	0	0.00	
DQ720065	0	0.00	
DQ720100	0	0.00	
DQ720133	0	0.00	
DQ720136	0	0.00	
DQ720158	0	0.00	
DQ720182	0	0.00	
DQ720185	0	0.00	
DQ720245	0	0.00	

DQ720250	0	0.00	
DQ720385	0	0.00	
DQ720410	0	0.00	
DQ720412	0	0.00	
DQ720427	0	0.00	
DQ720431	0	0.00	
DQ720439	0	0.00	
DQ720445	0	0.00	
DQ720468	0	0.00	
DQ720479	0	0.00	
DQ720502	0	0.00	
DQ720535	0	0.00	
DQ720544	0	0.00	
DQ720552	0	0.00	
DQ720562	0	0.00	
DQ720605	0	0.00	
DQ720611	0	0.00	
DQ720628	0	0.00	
DQ720635	0	0.00	
DQ720673	0	0.00	
DQ720689	0	0.00	
DQ720696	0	0.00	
DQ720699	0	0.00	
DQ720715	0	0.00	
DQ720744	0	0.00	
DQ720745	0	0.00	
DQ720750	0	0.00	
DQ720754	0	0.00	
DQ720759	0	0.00	
DQ720772	0	0.00	
DQ720778	0	0.00	
DQ720805	0	0.00	
DQ720829	0	0.00	
DQ720870	0	0.00	
DQ720875	0	0.00	
DQ720901	0	0.00	
DQ720906	0	0.00	
DQ720927	0	0.00	
DQ720952	0	0.00	
DQ720968	0	0.00	
DQ720972	0	0.00	
DQ720997	0	0.00	
DQ721008	0	0.00	
DQ721012	0	0.00	
DQ721024	0	0.00	
DQ721038	0	0.00	
DQ721063	0	0.00	

DQ721065	0	0.00	
DQ721078	0	0.00	
DQ721090	0	0.00	
DQ721116	0	0.00	
DQ721120	0	0.00	
DQ721121	0	0.00	
DQ721203	0	0.00	
DQ721216	0	0.00	
DQ721222	0	0.00	
DQ721229	0	0.00	
DQ721239	0	0.00	
DQ721308	0	0.00	
DQ721311	0	0.00	
DQ721319	0	0.00	
DQ721325	0	0.00	
DQ721327	0	0.00	
DQ721346	0	0.00	
DQ721347	0	0.00	
DQ721352	0	0.00	
DQ721373	0	0.00	
DQ721377	0	0.00	
DQ721385	0	0.00	
DQ721394	0	0.00	
DQ721397	0	0.00	
DQ721405	0	0.00	
DQ721411	0	0.00	
DQ721446	0	0.00	
DQ721504	0	0.00	
DQ721562	0	0.00	
DQ721581	0	0.00	
DQ721601	0	0.00	
DQ721604	0	0.00	
DQ721631	0	0.00	
DQ721672	0	0.00	
DQ721680	0	0.00	
DQ721685	0	0.00	
DQ721726	0	0.00	
DQ721765	0	0.00	
DQ721786	0	0.00	
DQ721815	0	0.00	
DQ721839	0	0.00	
DQ721858	0	0.00	
DQ721911	0	0.00	
DQ721917	0	0.00	
DQ721924	0	0.00	
DQ721944	0	0.00	
DQ721958	0	0.00	

DQ721963	0	0.00	
DQ721965	0	0.00	
DQ721971	0	0.00	
DQ721975	0	0.00	
DQ721976	0	0.00	
DQ722071	0	0.00	
DQ722090	0	0.00	
DQ722103	0	0.00	
DQ722152	0	0.00	
DQ722155	1.0239532	0.06	6.3%
DQ722161	0	0.00	
DQ722162	0	0.00	
DQ722174	0	0.00	
DQ722182	0	0.00	
DQ722193	0	0.00	
DQ722203	0	0.00	
DQ722227	0	0.00	
DQ722278	0	0.00	
DQ722287	0	0.00	
DQ722332	0	0.00	
DQ722338	0	0.00	
DQ722351	0	0.00	
DQ722357	0	0.00	
DQ722402	0	0.00	
DQ722415	0	0.00	
DQ722435	0	0.00	
DQ722438	0	0.00	
DQ722444	0	0.00	
DQ722445	0	0.00	
DQ722446	0	0.00	
DQ722450	0	0.00	
DQ722464	0	0.00	
DQ722489	0	0.00	
DQ722532	0	0.00	
DQ722594	0	0.00	
DQ722649	0	0.00	
DQ722660	0	0.00	
DQ722682	0	0.00	
DQ722700	0	0.00	
DQ722747	0	0.00	
DQ722800	0	0.00	
DQ722824	0	0.00	
DQ722831	0	0.00	
DQ722851	0.798953	0.12	15.3%
DQ722852	0	0.00	
DQ722884	0	0.00	
DQ722902	0	0.00	

DQ722916	0	0.00	
DQ722917	0	0.00	
DQ722920	0	0.00	
DQ723005	0	0.00	
DQ723006	0	0.00	
DQ723029	0	0.00	
DQ723061	0	0.00	
DQ723085	0	0.00	
DQ723102	0	0.00	
DQ723134	0	0.00	
DQ723160	0	0.00	
DQ723184	0	0.00	
DQ723201	0	0.00	
DQ723223	0	0.00	
DQ723226	0	0.00	
DQ723231	0	0.00	
DQ723239	0	0.00	
DQ723262	0	0.00	
DQ723275	0	0.00	
DQ723305	0	0.00	
DQ723308	0	0.00	
DQ723383	0	0.00	
DQ723397	0	0.00	
DQ723401	0	0.00	
DQ723404	0	0.00	
DQ723429	0	0.00	
DQ723438	0	0.00	
DQ723452	0	0.00	
DQ723458	0	0.00	
DQ723500	0	0.00	
DQ723510	0	0.00	
DQ723566	0	0.00	
DQ723570	0	0.00	
DQ723578	0	0.00	
DQ723589	0	0.00	
DQ723592	0	0.00	
DQ723595	0	0.00	
DQ723598	0	0.00	
DQ723607	0	0.00	
DQ723631	0	0.00	
DQ723633	0	0.00	
DQ723643	0	0.00	
DQ723648	0	0.00	
DQ723665	0	0.00	
DQ723677	0	0.00	
DQ723704	0	0.00	
DQ723763	0	0.00	

DQ723771	0	0.00	
DQ723777	0	0.00	
DQ723845	0	0.00	
DQ723850	0	0.00	
DQ723871	0	0.00	
DQ723876	0	0.00	
DQ723890	0	0.00	
DQ723909	0	0.00	
DQ723948	0	0.00	
DQ723949	0	0.00	
DQ723956	0	0.00	
DQ723978	0	0.00	
DQ723991	0	0.00	
DQ723996	0	0.00	
DQ724019	0	0.00	
DQ724027	0	0.00	
DQ724032	0	0.00	
DQ724092	0	0.00	
DQ724107	0	0.00	
DQ724109	0	0.00	
DQ724143	0	0.00	
DQ724198	0	0.00	
DQ724203	0	0.00	
DQ724237	0	0.00	
DQ724247	0	0.00	
DQ724252	0	0.00	
DQ724256	0	0.00	
DQ724299	0	0.00	
DQ724322	0	0.00	
DQ724356	0	0.00	
DQ724363	0	0.00	
DQ724380	0	0.00	
DQ724387	0	0.00	
DQ724475	0	0.00	
DQ724494	0	0.00	
DQ724508	0	0.00	
DQ724511	0	0.00	
DQ724512	0	0.00	
DQ724516	0	0.00	
DQ724531	0	0.00	
DQ724532	0	0.00	
DQ724541	0	0.00	
DQ724542	0	0.00	
DQ724543	0	0.00	
DQ724562	0	0.00	
DQ724574	0	0.00	
DQ724579	0	0.00	

DQ724588	0	0.00	
DQ724590	0	0.00	
DQ724625	0	0.00	
DQ724629	0	0.00	
DQ724646	0	0.00	
DQ724649	0	0.00	
DQ724664	0	0.00	
DQ724678	0	0.00	
DQ724684	0	0.00	
DQ724707	0	0.00	
DQ724729	0	0.00	
DQ724813	0	0.00	
DQ724814	0	0.00	
DQ724863	0	0.00	
DQ724865	0	0.00	
DQ724884	0	0.00	
DQ724896	0	0.00	
DQ724898	0	0.00	
DQ724952	0	0.00	
DQ725003	0	0.00	
DQ725027	0	0.00	
DQ725048	0	0.00	
DQ725059	0	0.00	
DQ725072	0	0.00	
DQ725081	0	0.00	
DQ725097	0	0.00	
DQ725111	0	0.00	
DQ725126	0	0.00	
DQ725128	0	0.00	
DQ725163	0	0.00	
DQ725177	0	0.00	
DQ725182	0	0.00	
DQ725209	0	0.00	
DQ725213	0	0.00	
DQ725256	0	0.00	
DQ725291	0	0.00	
DQ725296	0	0.00	
DQ725319	0	0.00	
DQ725325	0	0.00	
DQ725337	0	0.00	
DQ725339	0	0.00	
DQ725355	0	0.00	
DQ725364	0	0.00	
DQ725413	0	0.00	
DQ725435	0	0.00	
DQ725449	0	0.00	
DQ725458	0	0.00	

DQ725525	0	0.00	
DQ725560	0	0.00	
DQ725586	0	0.00	
DQ725616	0	0.00	
DQ725630	0	0.00	
DQ725656	0	0.00	
DQ725679	0	0.00	
DQ725680	0	0.00	
DQ725681	0	0.00	
DQ725733	0	0.00	
DQ725761	0	0.00	
DQ725778	0	0.00	
DQ725779	0	0.00	
DQ725784	0	0.00	
DQ725791	0	0.00	
DQ725848	0	0.00	
DQ725849	0	0.00	
DQ725860	0	0.00	
DQ725871	0	0.00	
DQ725875	0	0.00	
DQ725877	0	0.00	
DQ725898	0	0.00	
DQ725943	0	0.00	
DQ725962	0	0.00	
DQ726010	0	0.00	
DQ726025	0	0.00	
DQ726035	0	0.00	
DQ726065	0	0.00	
DQ726078	0	0.00	
DQ726121	0	0.00	
DQ726127	0	0.00	
DQ726146	0	0.00	
DQ726151	0	0.00	
DQ726154	0	0.00	
DQ726158	0	0.00	
DQ726174	0	0.00	
DQ726177	0	0.00	
DQ726245	0	0.00	
DQ726247	0	0.00	
DQ726248	0	0.00	
DQ726275	0	0.00	
DQ726285	0	0.00	
DQ726341	0	0.00	
DQ726346	0	0.00	
DQ726353	0	0.00	
DQ726379	0	0.00	
DQ726408	0	0.00	

DQ726412	0	0.00	
DQ726464	0	0.00	
DQ726470	0	0.00	
DQ726480	0	0.00	
DQ726481	0	0.00	
DQ726489	0	0.00	
DQ726495	0	0.00	
DQ726545	0	0.00	
DQ726565	0	0.00	
DQ726586	0	0.00	
DQ726589	0	0.00	
DQ726604	0	0.00	
DQ726614	0	0.00	
DQ726630	0	0.00	
DQ726631	0	0.00	
DQ726638	0	0.00	
DQ726650	0	0.00	
DQ726662	0	0.00	
DQ726766	0	0.00	
DQ726768	0	0.00	
DQ726785	0	0.00	
DQ726829	0	0.00	
DQ726843	0	0.00	
DQ726846	0	0.00	
DQ726861	0	0.00	
DQ726874	0	0.00	
DQ726875	0	0.00	
DQ726893	0	0.00	
DQ726911	0	0.00	
DQ726916	0	0.00	
DQ726954	0	0.00	
DQ727011	0	0.00	
DQ727024	0	0.00	
DQ727054	0	0.00	
DQ727056	0	0.00	
DQ727057	0	0.00	
DQ727065	0	0.00	
DQ727080	0	0.00	
DQ727096	0	0.00	
DQ727109	0	0.00	
DQ727184	0	0.00	
DQ727197	0	0.00	
DQ727213	0	0.00	
DQ727224	0	0.00	
DQ727227	0	0.00	
DQ727241	0	0.00	
DQ727320	0	0.00	

DQ727336	0	0.00	
DQ727343	0	0.00	
DQ727348	0	0.00	
DQ727384	0	0.00	
DQ727392	0	0.00	
DQ851563	0	0.00	
DQ874391	0	0.00	
Dqx1	0.169232	0.04	20.8%
Dr1	18.36384	1.07	5.8%
Dra	0	0.00	
Drap1	65.2014	4.33	6.6%
Drctnnb1a	4.30481	0.64	14.9%
Drd1a	0.07373384	0.02	33.6%
Drd1ip	88.08132	3.85	4.4%
Drd2	7.23483	0.30	4.2%
Drd3	0.00864052	0.01	140.8%
Drd4	0.07192894	0.01	18.4%
Drd5	0.496315	0.14	27.5%
DREAM/calsenilin/KChIP3	0.2234282	0.07	32.3%
Drg1	38.85294	1.78	4.6%
Drg2	15.74706	0.92	5.8%
DRIM	2.585688	0.14	5.6%
Drp1	5.13001	0.67	13.0%
Drp2	4.288802	0.67	15.6%
Dsc1	0.0286306	0.02	65.1%
Dsc2	0.6297574	0.09	14.2%
Dsc3	0.001429774	0.00	223.6%
DSC3	0	0.00	
Dscam	17.5214	0.71	4.0%
Dscaml1	3.223164	0.13	3.9%
Dscr2	8.901934	0.92	10.4%
Dscr3	20.93748	0.82	3.9%
Dse	1.895424	0.15	8.0%
Dsel	3.554972	0.12	3.4%
Dsg1b	0.004056206	0.01	140.5%
Dsg1c	0	0.00	
Dsg2	0.2119194	0.07	32.5%
Dsg3	0.011437932	0.01	72.5%
Dsg4	0	0.00	
Dsn1	2.414792	0.12	4.8%
Dsp	0.05308878	0.02	44.1%
Dspp	0.001330124	0.00	223.6%
Dst	12.23692	1.26	10.3%
Dstn	72.96252	5.91	8.1%
Dtd1	30.10212	0.77	2.6%
Dtl	0.595982	0.05	8.0%
Dtna	26.04974	2.00	7.7%

dtna	0.9046962	0.70	77.1%
Dtnb	10.09393	1.03	10.2%
dtn-b	0.2126198	0.07	33.8%
Dtnbp1	17.01814	1.65	9.7%
Dtwd1	2.331336	0.23	9.7%
Dtwd2	1.434618	0.11	7.7%
Dtx1	21.13314	0.52	2.4%
Dtx2	2.52505	0.25	9.9%
Dtx3	63.5939	2.30	3.6%
Dtx3l	0.3323706	0.05	14.7%
Dtx4	15.66276	1.57	10.1%
Dtymk	20.48522	1.38	6.7%
Dub1	0	0.00	
Dub1a	0	0.00	
Dub2	0	0.00	
Dub2a	0	0.00	
Dullard	14.83046	1.05	7.1%
Duox1	0.009363498	0.01	83.6%
Duox2	0.2009128	0.06	28.2%
Duoxa1	0.01679148	0.02	118.4%
Duoxa2	0.1144319	0.02	20.0%
Dupd1	0	0.00	
Dus1l	7.39689	0.38	5.1%
Dus2l	5.887908	0.55	9.3%
Dus3l	32.06912	2.14	6.7%
Dus4l	1.373646	0.03	2.3%
Dusp1	2.64007	0.41	15.4%
Dusp10	2.497346	0.20	8.1%
Dusp11	10.2655	0.17	1.7%
Dusp12	9.324302	0.90	9.7%
Dusp13	0.0328058	0.03	84.4%
Dusp14	0.6371192	0.12	18.6%
Dusp15	5.30398	0.46	8.7%
Dusp16	1.41109	0.33	23.5%
Dusp18	0.7779302	0.11	14.2%
Dusp19	3.342126	0.49	14.5%
Dusp2	0.9299394	0.13	13.5%
Dusp21	0	0.00	
Dusp22	15.50908	1.01	6.5%
Dusp23	1.526406	0.11	7.5%
Dusp26	23.17332	1.22	5.3%
Dusp27	0.013862388	0.01	47.0%
Dusp28	6.329628	0.24	3.8%
Dusp3	12.54342	0.68	5.4%
Dusp4	5.263074	1.48	28.2%
Dusp5	1.646166	0.34	20.4%
Dusp6	7.120518	0.72	10.1%

Dusp7	6.280318	0.29	4.7%
Dusp8	25.01758	1.19	4.8%
Dusp9	0.8623464	0.25	29.2%
Dut	8.412336	0.24	2.8%
Dutp	0.05895942	0.01	19.4%
Dux	0	0.00	
Dvl1	16.44918	1.16	7.0%
Dvl2	5.387514	0.59	10.9%
Dvl3	20.05948	0.37	1.8%
DXBay18	0.00339938	0.01	223.6%
Dxml1	0.12834022	0.05	37.5%
Dydc1	0.00510628	0.01	223.6%
Dydc2	0.08181352	0.06	74.8%
Dym	18.51862	0.39	2.1%
Dync1h1	45.29256	2.54	5.6%
Dync1i1	38.33088	1.09	2.8%
Dync1i2	48.74378	1.42	2.9%
Dync1li1	46.08504	1.74	3.8%
Dync1li2	49.49486	1.52	3.1%
Dync2h1	3.540218	0.32	9.1%
Dync2li1	7.169532	0.44	6.2%
Dynll1	63.09012	7.98	12.6%
Dynll2	281.1604	1.00	0.4%
Dynlrb1	133.3422	9.82	7.4%
Dynlrb2	0.11839048	0.03	22.5%
Dynlt1	0.17048316	0.08	45.1%
Dynlt3	78.21034	2.62	3.4%
Dyrk1a	19.94652	0.54	2.7%
Dyrk1b	4.919758	0.42	8.5%
Dyrk2	5.599786	0.29	5.2%
Dyrk3	1.281096	0.20	16.0%
Dyrk4	0	0.00	
Dysf	0.6614744	0.12	18.7%
Dysfip1	0.0067778	0.02	223.6%
dystrophin	3.600476	0.59	16.5%
Dytn	0.00456998	0.01	137.0%
Dyx1c1	0.4638118	0.12	26.2%
Dzip1	56.99888	1.41	2.5%
Dzip1l	2.6997	0.31	11.4%
Dzip3	38.17534	1.25	3.3%
E030002O03Rik	0	0.00	
E030010A14Rik	0.14364124	0.07	46.8%
E030011K20Rik	0	0.00	
E030018N11Rik	0.9569266	0.14	14.4%
E030019B06Rik	0.451994	0.14	30.6%
E030025P04Rik	0	0.00	
E030041M21Rik	14.77254	0.68	4.6%

E030049G20Rik	0.8689212	0.08	9.4%
E130009J12Rik	0.05172354	0.04	82.8%
E130012A19Rik	6.061358	0.52	8.6%
E130014J05Rik	3.447296	0.12	3.5%
E130016E03Rik	0.2380864	0.02	6.9%
E130018O15Rik	0	0.00	
E130112L23Rik	5.68271	0.54	9.5%
E130114P18Rik	0.5042472	0.34	68.0%
E130120F12Rik	0.1732106	0.07	38.7%
E130203B14Rik	2.074786	0.31	15.2%
E130303B06Rik	2.60217	0.15	5.9%
E130304F04Rik	0	0.00	
E130306D19Rik	0.1683758	0.02	10.3%
E130308A19Rik	2.727224	0.43	15.7%
E130309D02Rik	5.701948	0.35	6.2%
E130309D14Rik	6.65998	0.46	6.8%
E130309F12Rik	7.181926	1.51	21.0%
E130310K16Rik	1.4292326	0.30	20.8%
E130311K13Rik	2.709614	0.21	7.8%
E130319B15Rik	1.84661	0.16	8.7%
E230008N13Rik	0	0.00	
E230019M04Rik	0	0.00	
E230022H04Rik	4.215268	0.61	14.4%
E230025N22Rik	0.0146779	0.02	136.8%
E2f1	3.723008	0.36	9.6%
E2f2	0.5900158	0.12	20.8%
E2f3	2.469958	0.31	12.6%
E2F3	0.5941856	0.40	67.0%
E2f4	7.292492	0.53	7.3%
E2f5	2.11122	0.22	10.5%
E2f6	9.591628	0.88	9.1%
E2f7	0.217937	0.02	10.7%
E2f8	0.2565778	0.06	24.0%
E330009J07Rik	5.152156	0.32	6.3%
E330009P21Rik	0	0.00	
E330016A19Rik	0.2696046	0.06	23.1%
E330017A01Rik	0	0.00	
E330018D03Rik	2.69384	0.30	11.1%
E330021D16Rik	0	0.00	
E330026B02Rik	0.1256976	0.05	42.4%
E330034G19Rik	0	0.00	
E430002G05Rik	0.49022	0.13	26.5%
E430004N04Rik	0.01216404	0.01	106.6%
E430018J23Rik	1.234588	0.12	9.9%
E430025E21Rik	13.42574	0.20	1.5%
E430028B21Rik	5.774602	0.37	6.4%
E430029J22Rik	0	0.00	

E4f1	5.852106	0.15	2.5%
E530011F12Rik	0	0.00	
Eaf1	4.68017	0.24	5.1%
Eaf2	0.12628584	0.06	45.5%
Ean57	0.005050418	0.01	163.8%
Eapa2	0.012412314	0.00	16.8%
Eapp	13.6502	0.73	5.3%
Ear1	0	0.00	
Ear10	0	0.00	
Ear11	0	0.00	
Ear14	0	0.00	
Ear2	0	0.00	
Ear5	0	0.00	
Ear6	0	0.00	
Ears2	2.595826	0.22	8.4%
Ebag9	14.3889	0.55	3.8%
EBF	0.31280168	0.17	54.2%
Ebf1	5.8734	0.23	3.9%
Ebf2	0.8120416	0.25	30.8%
Ebf3	16.72286	1.09	6.5%
Ebf4	7.999898	0.35	4.4%
Ebi2	0	0.00	
Ebi3	0.7793602	0.16	19.9%
Ebna1bp2	14.45232	0.52	3.6%
Ebp	11.79472	1.77	15.0%
Ebpl	5.641228	0.41	7.3%
Ecd	11.1824	0.45	4.1%
Ece1	5.714858	0.41	7.2%
Ece2	11.55218	0.68	5.9%
Ecel1	12.89108	0.44	3.4%
Ecgf1	6.436416	0.26	4.0%
Ech1	8.564708	0.42	4.9%
Echdc1	10.64028	0.53	4.9%
Echdc2	1.30642	0.17	13.4%
Echdc3	0.01636708	0.02	114.0%
Echs1	23.43838	1.62	6.9%
Ecm1	0.6432206	0.11	17.1%
Ecm2	0.7426428	0.13	17.0%
Ecsit	12.8298	0.52	4.1%
Ect2	0.765642	0.12	15.6%
Ed2	4.414862	0.77	17.4%
Eda	2.02142	0.17	8.4%
Eda2r	2.532186	0.47	18.5%
Edar	0.00556492	0.01	143.4%
Edaradd	0.03597796	0.02	56.0%
Edc3	9.159068	0.61	6.7%
Edc4	13.92936	0.30	2.2%

Edem1	3.805622	0.20	5.2%
Edem2	7.305468	0.58	7.9%
Edem3	11.1369	0.57	5.1%
EDEM3	0.1092063	0.02	21.5%
Edf1	26.62948	2.62	9.9%
Edg1	2.906154	0.27	9.2%
Edg2	3.50284	0.38	10.7%
Edg3	4.192166	0.44	10.4%
Edg4	0.6914902	0.08	11.0%
Edg5	1.288712	0.12	9.3%
Edg6	0.00818396	0.01	139.0%
Edg7	0.1159384	0.05	45.9%
Edg8	0.4793524	0.11	22.3%
Edil3	88.36518	2.87	3.3%
Edn1	0.0834448	0.03	31.2%
Edn2	0.00410738	0.01	223.6%
Edn3	0.12690836	0.04	28.4%
Ednra	2.4759492	1.22	49.4%
Ednrb	21.13548	2.13	10.1%
edpk	0.635912	0.16	24.5%
Edr2_p36	0.989169	0.29	29.3%
Eea1	9.134066	0.43	4.7%
Eed	3.262554	0.30	9.3%
Eef1a1	368.7156	29.98	8.1%
Eef1a2	184.9072	9.39	5.1%
Eef1b2	115.3708	7.56	6.6%
Eef1d	22.0865	2.40	10.9%
Eef1e1	26.8425	3.94	14.7%
Eef1g	35.5068	3.03	8.5%
Eef2	354.381	21.81	6.2%
Eef2k	7.090918	0.55	7.7%
Eefsec	4.458374	0.54	12.2%
EF651840	0	0.00	
EF660528	0.0506185	0.05	104.7%
Efcab1	0.9324294	0.14	15.1%
Efcab2	7.33601	0.34	4.7%
Efcab3	0.08195502	0.03	41.8%
Efcab4a	0.632037	0.13	20.5%
Efcab4b	0.13177612	0.08	64.2%
Efcab5	0.4488044	0.11	23.4%
Efcbp1	7.695632	0.34	4.4%
Efcbp2	13.50422	1.24	9.2%
Efemp1	0.3829264	0.16	43.0%
Efemp2	10.180976	0.99	9.7%
Efha1	12.9694	0.99	7.6%
Efhb	0.018857022	0.02	80.0%
Efhc1	0.3288622	0.10	31.6%

Efhc2	0.11467738	0.04	36.7%
Efhd1	1.0330324	0.14	13.3%
Efhd2	17.29152	1.68	9.7%
Efna1	0.1801144	0.06	33.6%
Efna2	2.583892	0.48	18.7%
Efna3	23.33204	1.79	7.7%
Efna4	0.5291508	0.10	18.8%
Efna5	12.35022	0.50	4.1%
Efnb1	2.828482	0.33	11.8%
Efnb2	8.713572	0.62	7.1%
Efnb3	11.099472	1.85	16.7%
Efs	1.826344	0.34	18.4%
Eftud1	3.234458	0.13	4.0%
Eftud2	14.54384	0.26	1.8%
EG114600	0	0.00	
EG13909	0	0.00	
EG194588	0	0.00	
EG208426	0.919522	0.13	13.7%
EG209380	0	0.00	
EG209786	0.00976018	0.02	223.6%
EG210155	0	0.00	
EG210583	0.10999266	0.01	13.6%
EG210853	0.7206362	0.15	20.7%
EG210876	0.01023886	0.01	140.3%
EG211223	0	0.00	
EG212225	0.001911792	0.00	223.6%
EG214321	0.06066608	0.03	51.3%
EG214403	0.00946708	0.01	92.4%
EG215472	0	0.00	
EG215714	0.2078672	0.07	35.2%
EG218444	0	0.00	
EG224552	0.00339436	0.01	223.6%
EG224572	0	0.00	
EG224576	0	0.00	
EG224582	0	0.00	
EG224916	0	0.00	
EG225594	0.01551608	0.01	66.3%
EG229571	0.00319422	0.01	223.6%
EG229862	0	0.00	
EG231591	0	0.00	
EG232358	0	0.00	
EG232599	0.10992612	0.03	29.8%
EG232801	0	0.00	
EG233164	0.00386048	0.01	223.6%
EG235327	0.00405834	0.01	223.6%
EG236749	0.12935826	0.03	20.7%
EG237300	0.00577936	0.01	143.7%

EG238829	0.00756282	0.02	223.6%
EG240055	0.4840454	0.09	19.6%
EG240327	0.01051434	0.01	92.9%
EG240549	0.14263808	0.07	45.6%
EG241041	0.0129428	0.02	138.8%
EG243628	0	0.00	
EG244595	0.00389368	0.01	223.6%
EG245263	0.0052681	0.01	137.3%
EG245297	5.643488	0.49	8.7%
EG245376	0.01992996	0.03	164.7%
EG245405	5.561228	1.12	20.1%
EG266459	0.0105954	0.01	137.4%
EG269902	0.11589406	0.01	12.2%
EG278087	0	0.00	
EG317677	0.21198292	0.09	40.9%
EG328231	0.00645018	0.01	223.6%
EG328264	0.01306416	0.01	99.8%
EG328354	0.00504602	0.01	223.6%
EG328479	0.147476	0.02	16.6%
EG328644	13.05414	1.46	11.2%
EG328839	0.00359006	0.01	223.6%
EG329070	6.11546	0.85	13.9%
EG329541	0.03165082	0.02	69.6%
EG329763	0.00650856	0.01	223.6%
EG330157	0.00245644	0.01	223.6%
EG330305	0	0.00	
EG330496	0	0.00	
EG330503	6.321352	0.25	4.0%
EG330513	0.001812506	0.00	223.6%
EG330602	0.01651566	0.02	147.8%
EG331392	5.705788	0.56	9.9%
EG331493	0	0.00	
EG331529	0	0.00	
EG333452	0	0.00	
EG333669	0	0.00	
EG368203	0.15495484	0.12	76.3%
EG380907	0	0.00	
EG381438	12.75992	1.28	10.1%
EG381483	0	0.00	
EG381818	0	0.00	
EG381852	0	0.00	
EG381936	0	0.00	
EG382106	0.00988234	0.01	140.5%
EG382109	0.00776438	0.02	223.6%
EG382156	0	0.00	
EG382275	0	0.00	
EG382639	0.15763868	0.05	32.1%

EG384219	0	0.00	
EG384220	0.00330688	0.01	223.6%
EG384221	0	0.00	
EG384813	0	0.00	
EG384814	0	0.00	
EG385328	0	0.00	
EG386551	0	0.00	
EG406223	0	0.00	
EG408196	0	0.00	
EG432436	0	0.00	
EG432555	0.00225728	0.01	223.6%
EG432637	0.05210938	0.02	43.2%
EG432723	0	0.00	
EG432743	0.01087374	0.02	147.6%
EG432825	0	0.00	
EG432838	0	0.00	
EG432867	0	0.00	
EG432870	0.05843316	0.03	45.8%
EG432879	0.2854338	0.07	25.0%
EG432939	0.6084276	0.14	23.5%
EG432982	0.04699338	0.02	46.8%
EG432987	0	0.00	
EG433016	0	0.00	
EG433070	0	0.00	
EG433178	0	0.00	
EG433180	0	0.00	
EG433182	15.1289	1.58	10.5%
EG433365	0.0068599	0.01	140.4%
EG433632	0.0916299	0.04	44.4%
EG433634	0	0.00	
EG433923	19.73678	1.57	8.0%
EG434008	23.16506	2.62	11.3%
EG434128	61.46932	3.50	5.7%
EG434171	0.001669114	0.00	223.6%
EG434172	0	0.00	
EG434179	1.0248306	0.12	12.2%
EG434197	0.006571692	0.01	91.8%
EG434280	0.2221172	0.05	20.5%
EG434396	0	0.00	
EG434402	3.162982	0.49	15.6%
EG434459	0.0240297	0.02	77.6%
EG434510	0	0.00	
EG434674	0	0.00	
EG434701	0	0.00	
EG434726	0	0.00	
EG434727	0	0.00	
EG434729	0	0.00	

EG434760	0	0.00	
EG434764	0	0.00	
EG434797	0.004148278	0.01	138.8%
EG434881	0	0.00	
EG435337	0	0.00	
EG435366	0.13414092	0.04	26.6%
EG435864	0	0.00	
EG435916	0	0.00	
EG435970	0.06716176	0.02	35.6%
EG436240	0.6716062	0.11	16.9%
EG436523	0.00900034	0.02	223.6%
EG544710	0	0.00	
EG545013	0.02501328	0.04	153.6%
EG545047	0	0.00	
EG545136	0.389208	0.09	22.3%
EG545253	0.6770054	0.15	22.6%
EG545391	1.843182	0.19	10.1%
EG545477	0.0047551	0.01	223.6%
EG545645	0	0.00	
EG545728	0	0.00	
EG545758	0.01812616	0.03	155.9%
EG545861	0.002851552	0.00	137.7%
EG545874	0	0.00	
EG545893	0.02989878	0.04	137.7%
EG545925	0	0.00	
EG545929	0	0.00	
EG545936	0	0.00	
EG545947	0	0.00	
EG545952	0	0.00	
EG545963	0.8425842	0.17	20.5%
EG546038	0	0.00	
EG546150	0.00402938	0.01	223.6%
EG546166	0.02612676	0.02	82.0%
EG546282	0	0.00	
EG546325	0.01023606	0.01	79.5%
EG546335	0	0.00	
EG546347	0	0.00	
EG546729	0	0.00	
EG546896	0.00666434	0.01	223.6%
EG546912	0	0.00	
EG546913	0	0.00	
EG546981	0	0.00	
EG547109	0.2004206	0.06	28.4%
EG547347	0.04257952	0.02	46.5%
EG56544	0.3049076	0.07	21.6%
EG574081	0	0.00	
EG574083	0	0.00	

EG574403	3.112722	0.16	5.2%
EG574429	0.00280988	0.01	223.6%
EG619517	0	0.00	
EG619597	0.003135492	0.00	140.7%
EG619697	0	0.00	
EG619788	0	0.00	
EG620592	2.877144	0.32	11.2%
EG620672	0	0.00	
EG622139	0	0.00	
EG622339	0.02230264	0.03	118.0%
EG622408	0.0717535	0.03	36.4%
EG622976	0.02454168	0.02	76.9%
EG623131	0.489545	0.11	21.9%
EG623734	0.04515	0.05	118.2%
EG623898	0	0.00	
EG624219	0.02838718	0.03	108.4%
EG624765	0	0.00	
EG624845	0	0.00	
EG624855	0.041022	0.01	35.5%
EG624866	0.9458052	0.19	20.2%
EG625029	0.00717664	0.01	140.3%
EG625068	0.0376995	0.01	30.4%
EG625109	0.014289	0.01	104.1%
EG625131	0.14031826	0.08	57.6%
EG625321	0.01015484	0.01	140.2%
EG625424	0.00737308	0.01	142.3%
EG625699	0	0.00	
EG625716	0.03095476	0.03	97.0%
EG626359	0.05838818	0.02	38.2%
EG626615	0	0.00	
EG626942	0	0.00	
EG627132	0	0.00	
EG627367	0	0.00	
EG627479	0	0.00	
EG627537	0	0.00	
EG627576	0	0.00	
EG627636	0	0.00	
EG627743	0	0.00	
EG627782	1.426412	0.17	11.6%
EG627805	0	0.00	
EG627814	0	0.00	
EG627927	0.00903296	0.02	223.6%
EG628185	0	0.00	
EG628416	0.00603326	0.01	137.7%
EG628456	0	0.00	
EG628518	0	0.00	
EG628586	0.012268302	0.02	184.2%

EG629079	0	0.00	
EG629219	0	0.00	
EG629678	0.06439098	0.02	36.5%
EG629734	0.00601096	0.01	223.6%
EG630499	0.23560574	0.12	52.2%
EG630579	1.211438	0.13	11.1%
EG632671	0	0.00	
EG632778	0.02795146	0.02	61.4%
EG633640	10.556918	0.78	7.4%
EG634650	0.056698	0.03	58.4%
EG635895	0	0.00	
EG636104	0.0026619	0.01	223.6%
EG637004	0.00733976	0.01	138.4%
EG637053	0	0.00	
EG637908	0	0.00	
EG638102	0	0.00	
EG638695	0	0.00	
EG639396	0.04404152	0.05	103.1%
EG639653	0.8227644	0.12	14.8%
EG653016	0	0.00	
EG654453	0	0.00	
EG654465	0	0.00	
EG665186	0	0.00	
EG665203	0	0.00	
EG665210	0	0.00	
EG665227	0	0.00	
EG665255	0	0.00	
EG665376	0	0.00	
EG665378	0.00292778	0.01	223.6%
EG666002	0	0.00	
EG666085	0	0.00	
EG666105	0.12616294	0.05	37.3%
EG667060	0.00312914	0.01	223.6%
EG667069	0	0.00	
EG667180	0	0.00	
EG667283	0	0.00	
EG667736	0.00756684	0.02	223.6%
EG667977	1.345214	0.08	6.0%
EG668525	0.1866156	0.08	42.9%
EG668668	8.119382	0.84	10.3%
Egf	0.15351186	0.05	33.8%
Egfbp2	0	0.00	
Egfl6	0.05358214	0.02	34.7%
Egfl7	5.525094	0.65	11.8%
Egfl8	0.41536854	0.46	109.8%
Egflam	1.869712	0.31	16.3%
Egfr	2.43575	0.27	11.0%

Egln1	21.7726	1.33	6.1%
Egln2	23.09018	0.85	3.7%
Egln3	3.78182	0.82	21.6%
Egr1	8.08625	1.07	13.3%
Egr2	0.1923304	0.05	26.2%
Egr3	0.6423558	0.08	12.6%
Egr4	0.7547634	0.14	19.0%
Ehbp1	10.6908	0.48	4.5%
Ehbp1l1	1.1885036	0.23	19.3%
Ehd1	17.7601	2.22	12.5%
Ehd2	1.2809824	0.25	19.5%
Ehd3	22.51668	0.75	3.3%
Ehd4	4.98133	0.24	4.7%
Ehf	0.05618484	0.03	48.4%
Ehhadh	0.1918736	0.04	23.3%
Ehmt1	7.942198	0.49	6.1%
Ehmt2	28.61986	0.80	2.8%
Ei24	45.3275	2.76	6.1%
Eid1	61.33034	4.35	7.1%
Eid2	20.04886	0.82	4.1%
Eid3	0.02360436	0.01	61.4%
Eif1	51.90262	4.93	9.5%
Eif1a	26.27196	1.81	6.9%
Eif1ay	40.10922	1.11	2.8%
Eif1b	61.18594	3.48	5.7%
Eif2a	15.2093	0.51	3.4%
Eif2ak1	15.14478	1.10	7.3%
Eif2ak2	2.408502	0.25	10.2%
Eif2ak3	7.048756	0.31	4.4%
Eif2ak4	4.100316	0.17	4.2%
Eif2b1	9.86451	0.44	4.5%
Eif2b2	11.02176	0.99	9.0%
Eif2b3	9.90362	1.27	12.8%
Eif2b4	13.61408	0.61	4.5%
Eif2b5	24.4206	2.20	9.0%
Eif2c1	10.361218	0.37	3.5%
Eif2c2	16.99222	2.74	16.1%
Eif2c3	0.8740222	0.16	18.6%
Eif2c4	1.0856608	0.18	17.0%
Eif2s1	9.396228	0.64	6.8%
Eif2s2	19.22954	1.91	9.9%
Eif2s3x	5.648674	0.46	8.2%
Eif2s3y	0.7551886	0.11	14.7%
Eif3b	33.58834	2.34	7.0%
Eif3c	57.86146	4.55	7.9%
Eif3d	37.97342	4.00	10.5%
Eif3e	15.36546	1.22	7.9%

Eif3eip	53.88522	4.37	8.1%
Eif3f	61.38556	4.46	7.3%
Eif3g	30.1156	2.08	6.9%
Eif3h	53.23096	4.34	8.2%
Eif3i	25.43888	2.09	8.2%
Eif3j	1.247462	0.10	7.7%
Eif3k	46.50816	4.08	8.8%
Eif3m	12.774	0.96	7.5%
Eif3s10	56.90678	2.50	4.4%
Eif3s12	1.058503	0.42	39.6%
Eif3s2	0.09810208	0.03	29.8%
Eif4a1	15.12294	1.59	10.5%
Eif4a2	148.2048	4.30	2.9%
Eif4a3	8.539842	0.51	6.0%
Eif4b	55.8167	1.12	2.0%
Eif4e	20.3365	1.26	6.2%
Eif4e1b	0	0.00	
Eif4e2	32.16002	1.13	3.5%
Eif4e3	3.28587	0.25	7.6%
Eif4ebp1	2.481344	0.61	24.4%
Eif4ebp2	10.624532	1.15	10.8%
Eif4ebp3	3.11296	0.23	7.5%
Eif4enif1	9.812012	0.63	6.4%
Eif4g1	75.30158	2.85	3.8%
Eif4g2	141.966	5.30	3.7%
Eif4g3	38.19908	0.31	0.8%
Eif4h	95.8919	1.95	2.0%
Eif5	61.41338	1.45	2.4%
Eif5a	82.91286	6.24	7.5%
Eif5a2	18.69902	1.11	5.9%
Eif5b	8.893386	0.36	4.1%
Eif6	22.0729	2.07	9.4%
Ela1	0.09565484	0.04	44.4%
Ela2	0.00751372	0.02	223.6%
Ela2a	0.19023806	0.41	217.5%
Ela3	0.0061661	0.01	223.6%
Elac1	7.241264	0.53	7.3%
Elac2	6.766858	0.22	3.2%
Elavl1	13.9055	0.46	3.3%
Elavl2	53.2177	2.73	5.1%
Elavl3	59.60518	2.53	4.2%
Elavl4	43.26416	2.38	5.5%
Elf1	3.439566	0.50	14.6%
Elf2	6.142406	0.40	6.5%
ELF2	0.22381078	0.10	45.9%
Elf3	0.6534576	0.21	32.3%
Elf4	0.1988152	0.07	32.8%

Elf5	0.0122767	0.01	93.8%
Elk1	13.9836	2.96	21.2%
Elk3	1.459848	0.16	10.8%
Elk4	2.561834	0.32	12.6%
Ell	3.795856	0.24	6.4%
Ell2	3.79899	0.34	9.1%
Ell3	0.2128488	0.05	23.4%
Elm1	0.5426806	0.24	43.7%
Elmo1	49.36488	2.90	5.9%
Elmo2	13.3596	0.50	3.8%
Elmo3	0.4741638	0.03	6.9%
Elmod1	49.54168	2.13	4.3%
Elmod2	12.75842	0.64	5.0%
Eln	2.303532	0.88	38.2%
Elof1	20.92366	2.54	12.1%
Elovl1	2.619776	0.41	15.8%
Elovl2	5.731724	1.01	17.7%
Elovl3	0.00347938	0.01	223.6%
Elovl4	78.4401	3.34	4.3%
Elovl5	29.02216	2.36	8.1%
Elovl6	28.32036	3.71	13.1%
Elovl7	1.2494486	0.23	18.1%
ELP	0.0064363	0.01	223.6%
Elp2	34.64892	1.63	4.7%
Elp3	36.08646	2.80	7.8%
Elp4	5.365342	0.27	4.9%
Eltd1	0.01331845	0.00	35.5%
Emb	9.054696	0.64	7.1%
Emcn	0.05198596	0.05	87.0%
Emd	31.7215	2.94	9.3%
Eme1	0.1328862	0.02	14.5%
Eme2	6.861636	1.15	16.8%
Emg1	11.50532	0.63	5.5%
Emid1	1.88952	0.34	17.9%
Emid2	6.58917	1.33	20.2%
Emilin1	1.624906	0.29	17.8%
Emilin2	1.115164	0.05	4.5%
Emilin3	0.2331876	0.03	12.8%
Eml1	7.566408	0.53	6.9%
Eml2	41.54076	2.78	6.7%
Eml3	3.366624	0.69	20.4%
Eml4	8.777388	0.20	2.2%
Eml5	4.214722	0.45	10.8%
Emp1	14.51076	2.61	18.0%
Emp2	1.721648	0.19	11.2%
Emp3	1.0930684	0.47	43.4%
Emr1	0.03508104	0.03	84.3%

Emr4	0.01997116	0.01	44.7%
Emx1	0.5868536	0.15	25.6%
Emx2	0.8423228	0.15	17.2%
Emzf1	0.14300214	0.07	51.8%
En1	4.449082	0.23	5.1%
En2	0.820613	0.16	20.0%
Enah	20.48424	0.84	4.1%
Enam	0	0.00	
Enc1	36.24316	3.08	8.5%
Endod1	45.2907	1.26	2.8%
Endog	7.879156	0.61	7.7%
Endogl1	8.75655	0.71	8.1%
Eng	0.054278368	0.03	61.0%
Eno1	2.078816	0.61	29.1%
Eno2	348.5066	21.95	6.3%
Eno3	3.63719	0.30	8.3%
ENO3	0.4662322	0.44	93.6%
Enoph1	13.43292	0.70	5.2%
Enox1	8.581036	0.23	2.7%
Enox2	4.221594	0.28	6.7%
Enpep	0.20020088	0.07	34.4%
Enpp1	0.910251	0.11	12.0%
Enpp2	7.95412	0.45	5.7%
Enpp3	0.1801524	0.06	31.3%
Enpp4	2.6969	0.10	3.5%
Enpp5	101.63956	4.27	4.2%
Enpp6	0.1149162	0.09	75.4%
Enpp7	0	0.00	
Ensa	78.64626	2.37	3.0%
ENSMUSG00000033219	0	0.00	
ENSMUSG00000043661	0.0967146	0.11	115.2%
ENSMUSG00000048936	5.510494	0.61	11.1%
ENSMUSG00000052469	0.005192248	0.01	148.5%
ENSMUSG00000053178	0.4006514	0.09	23.4%
ENSMUSG00000058057	0.01982688	0.02	103.1%
ENSMUSG00000058934	0.00954074	0.02	223.6%
ENSMUSG00000068790	0	0.00	
ENSMUSG00000072602	0	0.00	
ENSMUSG00000073257	0	0.00	
Ent1	0.4779198	0.10	21.3%
Entpd1	0.2497062	0.07	26.9%
Entpd2	0.6888796	0.29	42.8%
Entpd3	1.2552538	0.24	19.1%
Entpd4	24.88428	2.25	9.0%
Entpd5	5.920552	0.24	4.0%
Entpd6	26.18208	1.00	3.8%
Entpd7	4.177466	0.18	4.4%

Entpd8	0.00574458	0.01	138.4%
env	0	0.00	
Eny2	18.92854	1.13	6.0%
Eomes	0.03611542	0.02	67.5%
ep	0.6990302	0.26	37.6%
Ep300	9.069894	0.34	3.8%
Ep400	7.098702	0.63	8.9%
Epas1	1.801066	0.32	17.7%
Epb4.1	13.69122	1.10	8.0%
Epb4.1l1	75.21048	1.52	2.0%
Epb4.1l2	12.098652	2.40	19.9%
Epb4.1l3	105.5484	3.53	3.3%
Epb4.1l4a	2.077752	0.40	19.1%
Epb4.1l4b	2.138938	0.24	11.2%
Epb4.1l5	2.299114	0.29	12.7%
Epb4.2	0.00285128	0.01	223.6%
Epb4.9	71.16162	3.40	4.8%
Epc1	11.36796	0.82	7.2%
Epc2	10.297254	0.39	3.8%
Epdr1	15.4602	1.70	11.0%
Epdr2	0.6040046	0.49	81.3%
Epgn	0.02100664	0.02	118.4%
Epha1	0.161001	0.04	27.1%
Epha10	1.0355368	0.14	13.8%
Epha2	0.13915316	0.05	38.8%
Epha3	1.820182	0.23	12.7%
Epha4	19.68948	0.50	2.5%
Epha5	22.46038	1.31	5.8%
Epha6	1.988094	0.43	21.8%
Epha7	5.587076	0.37	6.6%
Epha8	2.004284	0.12	6.0%
Ephb1	13.71008	1.47	10.7%
Ephb2	7.090094	0.69	9.8%
Ephb3	1.700612	0.18	10.5%
Ephb4	1.27069	0.12	9.5%
Ephb6	9.982844	0.30	3.0%
Ephx1	15.33754	1.54	10.0%
Ephx2	0.4384448	0.03	6.5%
Epm2a	7.245046	0.76	10.5%
Epm2aip1	77.97824	2.01	2.6%
Epn1	78.7515	4.12	5.2%
Epn2	11.45534	0.45	3.9%
Epn3	0.06681174	0.02	26.1%
Epo	0	0.00	
Epor	0.223977	0.03	12.0%
Eppk1	0.000970284	0.00	138.8%
Eprs	34.02178	2.20	6.5%

Eps15	60.83744	2.89	4.8%
Eps15l1	18.36774	0.45	2.4%
Eps8	3.470762	0.29	8.2%
Eps8l1	0.10453552	0.03	28.6%
Eps8l2	0.07258334	0.03	42.0%
Eps8l3	0.0152734	0.00	24.3%
Eps8R1	0.032121445	0.02	58.9%
Epsti1	0.00455372	0.01	223.6%
Epx	0.02603478	0.02	88.1%
Epyc	0.00987292	0.01	138.6%
Eral1	9.293994	0.54	5.9%
Eras	0.0262193	0.02	61.4%
Erbb2	0.6428446	0.09	14.6%
Erbb2ip	8.15017	0.38	4.7%
Erbb3	0.05494122	0.01	20.9%
Erbb4	1.4778106	0.32	21.3%
erbB4	0.2461624	0.13	53.4%
Erc1	16.55554	2.35	14.2%
Erc2	16.87308	1.03	6.1%
Ercc1	5.294606	0.40	7.6%
Ercc2	4.127352	0.57	13.9%
Ercc3	9.959114	0.61	6.2%
Ercc4	6.466704	0.31	4.8%
Ercc5	4.472124	0.23	5.1%
Ercc6	1.863158	0.07	3.8%
Ercc6l	0.2643006	0.05	18.6%
Ercc8	3.621964	0.84	23.3%
Erd2.1	0.378664	0.20	52.8%
Ereg	0.00228474	0.01	223.6%
Erf	0.89803	0.18	19.9%
Erg	0.01998142	0.02	89.2%
erg3	0.037232916	0.04	114.4%
Ergic1	38.17256	1.95	5.1%
Ergic2	12.39612	1.09	8.8%
Ergic3	59.9194	4.56	7.6%
Erh	7.842806	0.99	12.7%
Erich1	3.161414	0.15	4.8%
Erlin1	5.82882	0.25	4.3%
Erlin2	16.27096	0.73	4.5%
Ermap	0.03422742	0.02	50.7%
Ermp1	21.41148	0.50	2.4%
Ern1	1.390334	0.14	10.0%
Ern2	0.0439398	0.03	69.6%
Ero1l	10.378946	1.27	12.3%
Ero1lb	3.46472	0.29	8.4%
Erp27	0.00602122	0.01	223.6%
Erp29	47.8752	3.08	6.4%

Errfi1	10.248638	0.71	6.9%
ErT-1	0.00319026	0.01	223.6%
Es1	0	0.00	
Es113	0.4267442	0.17	38.8%
Es22	0.00298028	0.01	223.6%
Es2el	5.637244	0.14	2.5%
Es31	0	0.00	
Esam	0.09565498	0.06	61.0%
Esam1	0.10544642	0.04	38.6%
Esco1	4.89338	0.26	5.3%
Esco2	0.420283	0.16	38.5%
Esd	9.261666	0.29	3.1%
Esf1	7.865046	0.17	2.2%
Esm1	0.0083295	0.01	138.2%
Espl1	0.3379176	0.06	16.5%
Espn	0.2532432	0.06	23.5%
Espnl	0.01557333	0.01	58.1%
Esr1	0.1306096	0.01	11.2%
Esr2	0.02757668	0.01	37.2%
Esrra	12.53852	0.82	6.6%
Esrrb	1.190596	0.10	8.0%
Esrrg	13.36036	0.72	5.4%
Esx1	0.07818914	0.04	52.7%
ET	2.798896	0.89	31.7%
Etaa1	2.940724	0.32	10.8%
Etd	0	0.00	
Etf1	28.89432	1.54	5.3%
Etfa	9.555836	0.46	4.8%
Etfb	14.15958	0.79	5.6%
Etfdh	9.799948	0.20	2.0%
Ethe1	3.566028	0.24	6.8%
Etl4	9.212386	0.84	9.2%
Etnk1	69.55944	5.03	7.2%
Etnk2	0.683925	0.19	27.6%
Etohi1	0.3098522	0.06	17.8%
etoile	7.190358	1.11	15.4%
Ets1	1.68488	0.27	16.3%
Ets2	8.615736	1.00	11.7%
Etv1	1.282226	0.12	9.7%
Etv2	0.00468668	0.01	223.6%
Etv3	2.774004	0.19	6.9%
Etv4	2.73602	0.24	8.6%
Etv5	7.26867	0.56	7.7%
Etv6	2.247088	0.12	5.4%
Evc	1.430968	0.31	21.4%
Evc2	1.0959604	0.16	14.8%
Evi1	1.191208	0.20	16.8%

evi-1	0.07177486	0.05	67.7%
Evi27	0.021926952	0.01	57.2%
Evi2a	0.01061052	0.01	94.9%
Evi2b	0.00209858	0.00	223.6%
Evi5	13.76538	1.30	9.4%
Evl	67.44078	3.10	4.6%
Evl1	0.9707336	0.43	43.9%
Evpl	0.03237212	0.02	59.2%
Evx1	0.8982492	0.18	20.0%
Evx2	0.296704	0.03	11.7%
Ewsr1	24.4424	1.48	6.1%
Exdl1	0.599738	0.03	5.7%
Exdl2	10.512038	1.21	11.5%
Exo1	0.3035726	0.08	25.0%
Exoc1	15.68772	1.00	6.4%
Exoc2	12.60994	0.61	4.9%
Exoc3	26.03862	0.48	1.8%
Exoc3l	0.5116226	0.11	21.9%
Exoc3l2	0.788146	0.18	22.5%
Exoc4	22.63748	0.92	4.1%
Exoc5	14.31966	1.23	8.6%
Exoc6	10.539818	1.08	10.2%
Exoc7	26.64806	1.29	4.8%
Exoc8	5.1856	0.19	3.6%
Exod1	1.242202	0.52	41.5%
Exosc1	9.450436	0.66	7.0%
Exosc10	13.4108	0.57	4.2%
Exosc2	8.228292	0.77	9.4%
Exosc3	4.128538	0.10	2.4%
Exosc4	7.431284	0.49	6.6%
Exosc5	4.938586	0.44	8.9%
Exosc6	3.149022	0.35	11.0%
Exosc7	9.16934	0.99	10.8%
Exosc8	5.387242	0.44	8.1%
Exosc9	9.511742	0.95	10.0%
Exph5	0.02859642	0.01	35.8%
Expi	0	0.00	
Ext1	10.469162	1.19	11.3%
Ext2	16.38042	0.95	5.8%
Extl1	0.5167236	0.06	11.8%
Extl2	34.6804	1.18	3.4%
Extl3	23.83848	0.48	2.0%
Eya1	3.089622	0.26	8.3%
Eya2	1.763358	0.23	13.0%
Eya3	4.47911	0.10	2.2%
Eya4	0.1378738	0.04	25.5%
Ezh1	11.39252	0.52	4.6%

Ezh2	1.590546	0.33	20.8%
F10	0.00340312	0.01	223.6%
F11	0	0.00	
F11r	0.996254	0.17	17.4%
F12	0.00282232	0.01	223.6%
F13a1	0.07239382	0.06	76.7%
F13b	0	0.00	
F2	0.07136964	0.07	93.2%
F2r	10.024806	0.66	6.6%
F2rl1	0.09559132	0.05	51.3%
F2rl2	0.0975285	0.05	52.0%
F2rl3	0.2417661	0.10	42.7%
F3	2.669986	0.42	15.7%
F430201B04Rik	0.0030885	0.01	223.6%
F5	0.00000252	0.00	223.6%
F630043A04Rik	0.6985048	0.14	19.6%
F630110N24Rik	13.8391	1.05	7.6%
F7	0.00404912	0.01	223.6%
F730014I05Rik	8.118682	0.54	6.7%
F730015K02Rik	7.090608	0.85	12.0%
F730031O20Rik	0.153942	0.04	28.6%
F730047E07Rik	0.4056202	0.09	22.9%
F8	0.5532824	0.03	6.1%
F830021D11Rik	7.292422	0.13	1.8%
F830045P16Rik	0	0.00	
F830104D24Rik	0	0.00	
F830116E18Rik	0	0.00	
F8a	7.398652	0.41	5.6%
F9	0	0.00	
F930017I19Rik	3.775344	3.28	86.9%
F930104E18Rik	0.014512	0.02	104.8%
Fa2h	0.11144188	0.05	45.6%
Faah	4.8979	0.29	5.9%
Fabp1	0	0.00	
Fabp2	0.00851618	0.02	223.6%
Fabp3	5.948526	1.24	20.8%
Fabp4	0.10468336	0.11	104.4%
Fabp5	7.974686	0.61	7.6%
Fabp6	0	0.00	
Fabp7	168.401	16.78	10.0%
Fabp9	0	0.00	
Face-2	2.69973	0.87	32.1%
fad123	0.10000932	0.04	39.5%
Fadd	0.8905784	0.08	9.2%
Fads1	76.06134	6.23	8.2%
Fads2	78.95802	9.69	12.3%
Fads3	17.82188	0.55	3.1%

Fads6	1.443518	0.25	17.3%
FAE	3.444974	1.09	31.7%
Faf1	18.02808	0.97	5.4%
Fah	1.539692	0.29	18.7%
Fahd1	6.400562	0.34	5.3%
Fahd2a	6.592596	0.42	6.3%
Faim	17.61352	0.84	4.8%
Faim2	34.8838	7.51	21.5%
Faim3	0.02182132	0.03	127.6%
FAK	1.451908	0.29	20.1%
Fam100a	27.568	2.77	10.0%
Fam101b	1.231847	0.15	12.4%
Fam102b	9.201266	0.83	9.0%
Fam105b	2.496448	0.50	20.0%
Fam112b	0.01765642	0.02	139.2%
Fam113a	3.449212	0.31	8.9%
Fam122b	1.053096	0.49	46.3%
Fam18b	14.59096	0.67	4.6%
Fam46c	0.2943908	0.09	30.8%
Fam4a2	0.4693204	0.29	61.6%
Fam50b	0.0066401	0.01	138.0%
Fam55d	0.365836	0.03	9.2%
Fam57b	62.3476	4.18	6.7%
Fam76b	0.5832766	0.08	13.9%
Fam77D	4.412456	0.53	11.9%
Fam83d	0.4544494	0.04	8.2%
Fanca	2.879666	0.76	26.2%
Fancb	0.5241506	0.06	11.8%
Fancc	1.917154	0.44	22.9%
Fancd2	0.2047424	0.05	25.5%
Fance	4.17093	0.36	8.7%
Fancg	3.830696	0.29	7.5%
Fanci	0.522136	0.26	49.1%
Fancl	2.4143	0.57	23.6%
Fancm	2.220474	0.13	5.7%
Fank1	0.8510952	0.07	8.5%
Fap	2.015254	0.25	12.6%
Farp1	9.345538	0.81	8.7%
Farp2	2.222852	0.19	8.5%
Fars2	5.049536	0.17	3.3%
Farsa	1.613036	0.52	32.1%
Farsb	32.44114	3.10	9.6%
Farsla	18.03642	1.40	7.7%
Farslb	4.036032	1.08	26.8%
Fas	0.6434612	0.09	14.0%
Fasl	0.6362474	0.11	16.7%
Fasn	53.88096	5.64	10.5%

Fastk	25.3183	1.08	4.3%
Fastkd1	7.805544	0.28	3.6%
Fastkd2	6.280166	0.46	7.3%
Fastkd3	3.711526	0.19	5.2%
Fastkd5	2.784476	0.18	6.5%
Fat1	3.637354	0.49	13.4%
Fat2	0	0.00	
Fat3	2.855718	0.25	8.6%
Fat4	1.32065	0.15	11.4%
Fate1	0.01402084	0.02	137.6%
Fath	7.335214	0.79	10.8%
Fau	2.352224	0.58	24.6%
Fba	10.440318	1.16	11.1%
Fbf1	9.631266	0.34	3.5%
Fbl	1.0179848	0.17	17.2%
Fblim1	3.231538	0.27	8.4%
Fbln1	6.416948	1.46	22.7%
Fbln2	0.9554626	0.39	40.4%
Fbln5	1.425138	0.25	17.6%
Fbn1	4.454418	0.99	22.3%
Fbn2	3.43498	0.80	23.2%
Fbp1	0.159899	0.05	33.8%
FBP11	0.04637644	0.03	56.4%
Fbp2	0.0285457	0.04	130.4%
Fbs1	9.462046	0.59	6.3%
Fbxl10	9.32996	1.27	13.6%
Fbxl11	18.87146	2.97	15.7%
Fbxl12	6.714448	1.27	18.9%
Fbxl13	0.30233232	0.66	219.3%
Fbxl14	5.473444	0.36	6.6%
Fbxl15	11.1694	0.75	6.8%
Fbxl16	61.97994	1.54	2.5%
Fbxl17	25.46118	1.59	6.2%
Fbxl18	7.09938	0.18	2.5%
Fbxl19	24.9489	2.20	8.8%
Fbxl2	11.83694	0.42	3.6%
Fbxl20	8.462376	0.80	9.4%
Fbxl21	1.5908	0.10	6.6%
Fbxl22	0.04582206	0.01	22.7%
Fbxl3	9.201872	0.29	3.2%
Fbxl4	10.73744	0.68	6.3%
Fbxl5	14.63	0.63	4.3%
Fbxl6	8.80081	0.44	5.0%
Fbxl7	1.414924	0.07	5.3%
Fbxl8	0.8165724	0.11	13.4%
Fbxo10	9.606834	0.91	9.5%
Fbxo11	40.30566	2.17	5.4%

Fbxo15	0.1161999	0.03	27.7%
Fbxo16	4.64191	0.51	11.1%
Fbxo17	0.09673478	0.05	56.0%
Fbxo18	25.74014	0.88	3.4%
Fbxo2	32.01648	2.37	7.4%
Fbxo21	36.82516	0.52	1.4%
Fbxo22	15.0008	1.24	8.3%
Fbxo24	0.06561014	0.02	31.8%
Fbxo25	27.08148	0.77	2.8%
Fbxo27	1.883512	0.23	12.1%
Fbxo28	9.94188	0.13	1.3%
Fbxo3	37.3325	0.96	2.6%
Fbxo30	3.31365	0.29	8.7%
Fbxo31	28.07696	1.24	4.4%
Fbxo32	0.6078096	0.18	29.4%
Fbxo33	4.981574	0.66	13.3%
Fbxo34	10.49696	0.23	2.1%
Fbxo36	1.491506	0.43	28.7%
Fbxo38	11.74292	0.45	3.9%
Fbxo39	0.12554594	0.06	45.7%
Fbxo4	2.058724	0.20	9.9%
Fbxo40	0.01348282	0.01	66.3%
Fbxo41	21.26838	0.63	3.0%
Fbxo42	10.2335	0.26	2.5%
Fbxo43	0.007313984	0.01	100.0%
Fbxo44	34.9633	1.35	3.9%
Fbxo45	11.57876	0.48	4.1%
Fbxo46	2.600574	0.11	4.1%
Fbxo47	0.6978392	0.14	20.2%
Fbxo5	1.519458	0.21	14.0%
Fbxo6	7.670748	0.30	4.0%
Fbxo7	6.56008	0.36	5.6%
Fbxo8	3.979796	0.38	9.5%
Fbxo9	26.74048	3.73	13.9%
Fbxw10	0.0745052	0.10	140.2%
Fbxw11	43.96308	0.84	1.9%
Fbxw12	0	0.00	
Fbxw13	0	0.00	
Fbxw14	0	0.00	
Fbxw15	0.00449176	0.01	223.6%
Fbxw16	0	0.00	
Fbxw17	2.143468	0.08	3.7%
Fbxw19	0	0.00	
Fbxw2	29.16616	2.43	8.3%
Fbxw4	11.10612	0.86	7.8%
Fbxw5	27.47994	1.25	4.6%
Fbxw6	6.08524	0.48	7.8%

Fbxw7	13.33314	0.45	3.4%
Fbxw8	9.143674	0.42	4.6%
Fbxw9	14.13488	1.58	11.1%
Fcamr	0.0220162	0.01	65.2%
Fcer1a	0	0.00	
Fcer1g	0	0.00	
Fcer2a	0.327917	0.04	12.3%
Fcf1	1.0034228	0.22	22.0%
Fcgbp	0.09594638	0.05	54.5%
Fcgr1	0.0574246	0.03	45.7%
Fcgr2b	0.01270798	0.02	159.0%
Fcgr3	0.0593855	0.07	109.5%
Fcgr4	0	0.00	
Fcgrt	1.3159936	0.28	21.4%
Fcho1	22.3065	1.19	5.3%
Fcho2	4.642192	0.50	10.7%
Fchsd1	3.885514	0.16	4.2%
Fchsd2	18.575	0.61	3.3%
Fcmd	10.29182	0.37	3.5%
Fcna	0.0061111	0.01	223.6%
Fcnb	0.01666018	0.02	94.0%
Fcrl1	0	0.00	
Fcrl5	0	0.00	
Fcrl6	0.00759384	0.02	223.6%
Fcrla	0	0.00	
Fcrlb	0.09293576	0.05	57.0%
Fdft1	53.24392	7.69	14.5%
Fdps	11.9845	2.40	20.0%
Fdx1	5.0268	0.35	6.9%
Fdxr	1.712892	0.19	11.3%
Fech	12.47826	0.28	2.2%
Fem1a	8.988054	0.53	5.9%
Fem1b	52.17462	1.87	3.6%
Fem1c	10.95806	0.71	6.4%
Fen1	5.98333	0.46	7.6%
Fer1l3	0.3357344	0.06	19.2%
Ferd3l	0.02600984	0.02	60.2%
Fert2	6.48974	0.80	12.3%
Fes	0.07140766	0.04	59.9%
Fetub	0.00296854	0.01	223.6%
Fev	0.15196466	0.08	55.0%
Fez1	84.15584	2.66	3.2%
Fez2	15.03854	0.72	4.8%
Fezf1	0.00633034	0.01	138.0%
Fezf2	0.03855508	0.03	79.8%
Ffar1	0	0.00	
Ffar2	0.00888982	0.01	137.7%

Ffar3	0.00471148	0.01	223.6%
Fga	0	0.00	
Fgb	0	0.00	
Fgd1	8.537072	0.49	5.7%
Fgd2	0.009970712	0.01	118.9%
Fgd3	0.14074402	0.05	35.6%
Fgd4	3.828816	0.46	12.0%
Fgd5	4.76469	0.62	12.9%
Fgd6	0.6783324	0.06	8.3%
Fgf1	5.241652	1.03	19.7%
Fgf10	1.275968	0.20	15.9%
Fgf11	9.774814	1.23	12.5%
Fgf12	75.90488	2.09	2.8%
Fgf13	64.52772	4.61	7.1%
Fgf14	5.2873	0.32	6.0%
Fgf15	1.340124	0.27	20.5%
Fgf16	0.06309304	0.03	42.5%
Fgf17	0.14312472	0.06	41.8%
Fgf18	2.46671	0.18	7.5%
Fgf2	0.27270294	0.18	65.1%
Fgf20	0.00720368	0.02	223.6%
Fgf21	0.0343476	0.03	101.5%
Fgf22	1.56372	0.17	10.9%
Fgf23	0.25308	0.07	28.9%
Fgf3	1.4074	0.17	12.4%
Fgf4	0	0.00	
Fgf5	0.459936	0.07	15.3%
Fgf6	0	0.00	
Fgf7	0.5336244	0.12	22.9%
Fgf8	0.04018386	0.04	107.3%
Fgf9	12.21292	1.01	8.3%
Fgfbp1	0.01189624	0.01	91.9%
Fgfbp3	0.7687976	0.20	25.9%
Fgfr1	19.06654	1.07	5.6%
Fgfr1op	8.969908	0.28	3.1%
Fgfr1op2	12.24346	0.89	7.2%
Fgfr2	3.28517	0.22	6.8%
Fgfr3	1.1044572	0.21	19.4%
Fgfr4	0.1212275	0.05	39.3%
Fgfrl1	4.388716	0.53	12.2%
Fgg	0.01221256	0.01	93.8%
Fgl1	0.00604104	0.01	223.6%
Fgl2	0.5055834	0.04	8.4%
Fgr	0.01305534	0.01	59.4%
Fh1	58.17256	2.53	4.3%
Fhad1	0.137809	0.03	23.4%
Fhit	2.650626	0.40	15.0%

Fhl1	73.64128	2.33	3.2%
Fhl2	4.983896	0.47	9.5%
Fhl3	0.328305	0.07	21.1%
Fhl4	0.320155	0.05	16.3%
Fhl5	0.02673622	0.03	102.6%
Fhod1	1.399276	0.10	6.9%
Fhod3	11.63278	0.89	7.6%
FHOS2	0.03592546	0.01	25.9%
fi58gm	11.59318	1.09	9.4%
Fibcd1	3.260214	0.32	9.7%
Fibp	25.51384	0.62	2.4%
Fig4	12.45722	0.68	5.4%
Figf	0.4141098	0.12	29.7%
Figla	0.00666858	0.01	223.6%
Fign	0.5362922	0.05	9.5%
Fignl1	0.5137316	0.08	15.9%
Filip1	0.04170878	0.01	27.6%
Fip1l1	20.8618	0.61	2.9%
Fis1	47.11522	3.57	7.6%
Fiz1	6.488324	0.27	4.1%
Fjx1	8.950094	0.99	11.1%
Fkbp10	3.470354	0.72	20.8%
Fkbp11	3.031012	0.35	11.5%
FKBP12-T1	4.666434	1.13	24.3%
Fkbp14	5.82469	0.54	9.2%
Fkbp15	0.11888554	0.05	37.9%
Fkbp1a	92.87622	5.32	5.7%
Fkbp1b	31.23262	2.80	9.0%
FKBP1b-B	0.945939	0.43	45.8%
Fkbp2	70.04778	4.27	6.1%
FKBP23	0.2476286	0.16	66.2%
Fkbp3	93.68238	3.82	4.1%
Fkbp4	63.84206	3.83	6.0%
Fkbp5	4.801752	0.59	12.3%
Fkbp6	0	0.00	
Fkbp7	2.305702	0.25	11.0%
Fkbp8	79.16632	3.98	5.0%
Fkbp9	11.369258	1.26	11.0%
Fkbpl	2.963608	0.22	7.4%
Fkhl18	0.2495008	0.07	29.0%
Fkhr2	0.04892158	0.01	29.3%
Fkrp	13.5498	0.76	5.6%
Flad1	6.51724	0.20	3.1%
Flame-3	1.359624	0.42	30.8%
Flcn	12.73248	0.37	2.9%
Flg	0	0.00	
Flg2	0	0.00	

Fli1	0.13208588	0.06	44.7%
Flii	9.392226	0.64	6.9%
Flna	17.36002	1.29	7.5%
Flnb	3.870786	0.57	14.8%
Flnc	1.40416	0.18	13.1%
Flot1	56.86772	2.68	4.7%
Flot2	45.64536	1.80	3.9%
FLRG	0.17420376	0.05	29.0%
Flrt1	1.894628	0.26	13.9%
Flrt2	4.789986	0.39	8.2%
Flrt3	11.42404	0.81	7.1%
Flt1	0.6947968	0.20	29.3%
Flt3	3.014596	0.41	13.6%
Flt3l	0.498861	0.04	8.0%
Flt4	0.1949772	0.03	14.8%
Flvcr1	6.320982	1.11	17.6%
Flvcr2	0.12293372	0.03	21.6%
Flywch1	64.32094	5.36	8.3%
Flywch2	16.5885	2.70	16.3%
Fmn1	0.3051164	0.11	35.9%
Fmn2	9.570806	0.35	3.7%
Fmnl1	6.010128	0.27	4.4%
Fmnl2	7.346212	0.36	4.9%
Fmnl3	1.376458	0.08	5.6%
Fmo1	0.8215154	0.45	55.0%
Fmo2	0.1312135	0.08	63.5%
Fmo3	0	0.00	
Fmo4	0	0.00	
Fmo5	0.07575506	0.03	35.4%
Fmo9	0.00294876	0.01	223.6%
Fmod	0.261338	0.06	22.9%
Fmr1	16.25836	0.94	5.8%
Fmr1nb	0.08467838	0.06	76.3%
fmr2	0.01852394	0.00	25.1%
Fn1	7.571182	1.87	24.7%
Fn3k	13.240392	2.33	17.6%
Fn5	0.8985876	0.59	66.1%
Fnbp1	41.2052	2.00	4.9%
Fnbp1l	20.94804	2.94	14.0%
Fnbp4	9.785422	0.41	4.2%
Fndc1	0	0.00	
Fndc3a	16.73602	0.71	4.3%
Fndc3b	5.349582	0.41	7.6%
Fndc4	27.9648	2.10	7.5%
Fndc5	16.8173	1.34	8.0%
Fndc7	0.00396425	0.01	140.8%
Fndc8	0.6777212	0.13	18.7%

Fnip1	8.006474	0.87	10.8%
Fnta	35.14738	1.34	3.8%
Fntb	7.95761	0.31	3.8%
Fog-2	0.02843348	0.01	26.1%
Folh1	0.436867	0.13	30.3%
Folr1	0.04488774	0.07	163.6%
Folr2	0.02494778	0.02	84.3%
Folr4	0.02482918	0.02	83.2%
Fos	3.05726	0.70	23.0%
Fosb	0.2878402	0.06	22.0%
Fosl1	0.05542632	0.03	54.7%
Fosl2	2.179088	0.17	7.7%
Foxa1	0.1316416	0.03	21.9%
Foxa2	0.6237616	0.23	36.6%
Foxa3	0.00980818	0.01	139.0%
Foxb1	0.3827616	0.06	14.5%
Foxb2	0.0246183	0.01	38.9%
Foxc1	0.4619584	0.03	7.4%
Foxc2	0.04815396	0.02	49.3%
Foxd1	0.1995652	0.08	42.6%
Foxd2	0.11164474	0.04	36.2%
Foxd3	1.33637	0.24	18.1%
Foxd4	0	0.00	
Foxe1	0.2121362	0.06	27.6%
Foxe3	0.0093062	0.02	223.6%
Foxf1a	0.4422028	0.12	27.4%
Foxf2	0.310305	0.09	27.9%
Foxg1	0.11075094	0.02	21.7%
Foxh1	0.02893936	0.01	50.7%
Foxi1	0	0.00	
Foxi2	0.00563284	0.01	138.7%
Foxj1	2.879712	0.74	25.9%
Foxj2	5.449428	0.24	4.4%
Foxj3	18.07072	0.71	3.9%
Foxk1	4.570586	0.18	3.9%
Foxk2	22.6778	2.34	10.3%
Foxl1	0.00484094	0.01	139.7%
Foxl2	1.1356926	0.30	26.7%
Foxm1	1.2396858	0.21	16.9%
Foxn1	0	0.00	
Foxn2	1.0579618	0.15	14.2%
Foxn3	23.92762	6.56	27.4%
Foxn4	0	0.00	
Foxo1	0.835595	0.05	6.6%
Foxo3a	10.198706	1.24	12.2%
Foxo4	3.018138	0.61	20.3%
Foxo6	0.9499664	0.14	15.1%

Foxp1	3.53172	0.24	6.9%
Foxp1a	0.03521732	0.01	41.3%
Foxp2	3.300938	0.31	9.3%
Foxp3	0.37046316	0.52	139.4%
Foxp4	1.1506808	0.23	20.0%
Foxq1	0.3818242	0.05	14.1%
Foxr1	0.0035359	0.01	223.6%
Foxred1	13.1387	0.78	6.0%
Foxred2	10.514058	0.73	6.9%
Fpgs	1.923426	0.11	5.7%
Fpgt	6.178598	0.28	4.5%
Fpr1	0.02880586	0.03	103.9%
Fprl1	0	0.00	
Fpr-rs2	0.04356496	0.04	96.1%
Fpr-rs6	0	0.00	
Fr1	0.3374722	0.22	64.4%
Frag1	7.147384	0.67	9.4%
Frap1	16.06924	0.60	3.7%
Fras1	1.11608	0.09	7.8%
Frat1	3.885286	0.31	7.9%
Frat2	1.738078	0.20	11.4%
Frem1	0.3622832	0.02	6.5%
Frem2	0.13190212	0.06	42.6%
Freq	74.82578	2.06	2.8%
Frg1	12.50462	0.50	4.0%
Frk	0.2562058	0.04	16.8%
Frmd3	5.221062	0.54	10.3%
Frmd4a	13.35364	1.45	10.9%
Frmd4b	1.761442	0.25	14.0%
Frmd5	11.13712	0.77	6.9%
Frmd6	1.353562	0.13	9.9%
Frmpd1	1.839098	0.11	5.8%
Frmpd3	3.759016	0.58	15.5%
Frmpd4	4.079106	0.61	15.0%
Frrs1	0.3145468	0.10	31.3%
Frs2	7.533568	0.50	6.6%
Frs3	13.54136	1.16	8.6%
Fry	12.3978	1.08	8.7%
Fryl	4.999422	0.24	4.9%
Frzb	1.315432	0.15	11.1%
Fsbp	0.1518128	0.04	23.8%
Fscn1	131.3712	14.92	11.4%
Fscn2	0.1544116	0.08	53.9%
Fscn3	0.02327026	0.02	64.6%
Fsd1	31.26636	1.51	4.8%
Fsd1l	2.467912	0.31	12.4%
Fsd2	0.008803034	0.01	59.9%

Fshb	0	0.00	
Fshr	0	0.00	
Fsip1	0.1918562	0.04	21.6%
Fsrg3	2.621868	0.19	7.1%
Fst	1.0403152	0.23	21.8%
Fstl1	39.04274	4.99	12.8%
Fstl3	0.10304996	0.03	31.1%
Fstl4	2.857626	0.11	3.8%
Fstl5	26.76062	1.33	5.0%
Ftcd	0.00553244	0.01	223.6%
Fth1	434.6834	13.46	3.1%
Fthl17	0	0.00	
Ftl1	7.353584	0.52	7.0%
Ftl2	24.31184	3.49	14.3%
Ftmt	0	0.00	
Fto	64.49394	1.14	1.8%
Ftsj1	5.416612	0.54	10.0%
Ftsj2	3.16647	0.24	7.7%
Ftsj3	8.716836	0.34	4.0%
Fubp1	25.145	1.40	5.6%
Fubp3	7.34733	0.12	1.6%
Fuca1	12.63276	1.30	10.3%
Fuca2	19.23724	1.07	5.6%
Fuk	1.1053426	0.19	16.7%
FuK	0.6760458	0.10	14.1%
Fundc1	22.13362	1.86	8.4%
Fundc2	28.1599	1.42	5.0%
Furin	6.19809	0.41	6.7%
Fus	53.62798	1.34	2.5%
Fusip1	9.234562	1.40	15.2%
Fut1	0.03701214	0.05	122.2%
Fut10	0.4416098	0.06	14.6%
Fut11	4.817704	0.29	6.0%
Fut2	0.05511126	0.02	28.3%
Fut4	0.09006994	0.02	27.2%
Fut7	0.903695	0.11	11.9%
Fut8	15.81024	1.06	6.7%
Fut9	13.08522	1.04	7.9%
Fuz	2.926694	0.17	5.9%
Fv1	2.005224	0.37	18.6%
Fvt1	5.538656	0.47	8.4%
Fxc1	9.978752	1.31	13.1%
Fxn	2.239036	0.20	9.1%
Fxr1	5.952188	0.40	6.7%
Fxr1h	27.9373	1.57	5.6%
Fxr2	26.13348	0.60	2.3%
Fxy	5.500482	4.10	74.5%

Fxyd1	0.6041596	0.19	30.8%
Fxyd2	0.8882618	0.34	38.3%
Fxyd3	1.670664	0.40	23.9%
Fxyd4	0.02199702	0.02	91.9%
Fxyd5	0.519056	0.18	34.8%
Fxyd6	70.12866	10.08	14.4%
Fxyd7	68.65306	10.35	15.1%
Fyb	1.0698666	0.61	57.3%
Fyco1	2.128974	0.31	14.4%
Fyn	65.03364	4.86	7.5%
Fyttd1	31.38138	0.80	2.6%
Fzd1	5.055748	0.42	8.4%
Fzd10	2.539886	0.43	16.8%
Fzd3	5.17922	0.58	11.1%
Fzd4	1.295582	0.16	12.0%
Fzd5	0.4748444	0.04	8.1%
Fzd6	0.2981372	0.08	25.2%
Fzd7	1.483862	0.30	20.4%
Fzd8	2.610014	0.10	3.8%
Fzd9	1.712992	0.11	6.2%
Fzr1	33.16946	2.57	7.8%
G0s2	1.775932	0.61	34.6%
G2ad	1.8252932	0.96	52.4%
G3BP	0.12482298	0.03	24.1%
G3bp1	19.52826	1.33	6.8%
G3bp2	75.9277	1.81	2.4%
G430022H21Rik	5.388592	0.28	5.1%
G5pr	0.4453482	0.10	23.2%
G630014P10Rik	3.714	0.16	4.2%
G630016D24Rik	0.08713394	0.06	70.7%
G630039H03Rik	0.755701	0.11	15.1%
G6pc	0	0.00	
G6pc2	0.01978676	0.02	119.0%
G6pc3	36.12208	1.13	3.1%
G6pd2	3.018874	0.14	4.6%
G6pdx	7.946242	0.38	4.8%
G9a	2.573852	0.33	12.8%
Gaa	93.81834	3.21	3.4%
Gab1	3.085614	0.36	11.7%
Gab2	6.915042	0.51	7.4%
Gab3	0.950887	0.09	9.7%
GABA-B-R	0.4848576	0.18	37.5%
Gabarap	105.69172	9.71	9.2%
Gabarapl1	243.666	5.48	2.2%
Gabarapl2	13.32014	0.92	6.9%
Gabbr1	169.7202	6.64	3.9%
Gabbr2	26.20778	0.39	1.5%

Gabpa	8.5826	1.10	12.8%
Gabpb1	8.571086	1.13	13.2%
Gabpb2	4.119154	0.53	12.8%
Gabra1	9.01074	0.80	8.9%
Gabra2	28.22418	1.85	6.6%
Gabra3	26.0508	0.83	3.2%
Gabra4	5.83007	0.17	2.9%
Gabra5	28.9894	3.17	10.9%
Gabra6	0	0.00	
Gabrb1	3.906874	0.48	12.3%
Gabrb2	13.46166	1.18	8.8%
Gabrb3	56.24872	2.58	4.6%
Gabrd	0.21333736	0.08	38.4%
Gabre	1.0810398	0.24	22.4%
Gabrg1	3.84602	0.26	6.9%
Gabrg2	5.16443	0.24	4.7%
Gabrg3	6.216938	0.67	10.8%
Gabrp	0.00442304	0.01	137.7%
Gabrq	3.408924	0.29	8.5%
Gabrr1	0.00224802	0.01	223.6%
Gabrr2	0.04062042	0.02	40.6%
Gabrr3	0	0.00	
Gad1	10.03442	1.02	3.4%
Gad2	13.8832	0.51	2.1%
Gadd45a	4.590574	0.34	7.4%
Gadd45b	1.485746	0.26	17.4%
Gadd45g	4.75608	0.45	9.5%
Gadd45gip1	8.006658	1.34	16.7%
Gadl1	5.53716	0.42	7.6%
Gak	26.53206	0.71	2.7%
Gal	0.186828	0.14	76.3%
Gal3st1	3.281578	0.36	10.9%
Gal3st2	0	0.00	
Gal3st3	11.259	0.31	2.8%
Gal3st4	1.0932684	0.19	17.0%
Galc	4.847698	0.44	9.1%
Gale	9.746038	0.60	6.1%
Galk1	3.472008	0.77	22.1%
Galk2	10.094926	0.86	8.5%
Galm	0.8642788	0.18	20.5%
Galnact2	3.353404	0.28	8.4%
Galns	3.90878	0.42	10.8%
Galnt1	14.86006	0.70	4.7%
Galnt10	2.523986	0.17	6.8%
Galnt11	23.311	1.39	6.0%
Galnt12	0.212529	0.06	26.5%
Galnt13	16.3242	0.85	5.2%

Galnt14	9.251702	0.30	3.3%
Galnt2	22.56834	1.58	7.0%
Galnt3	0.13954124	0.07	50.4%
Galnt4	0.6742018	0.12	17.1%
Galnt5	0	0.00	
Galnt6	0.15812254	0.09	59.3%
Galnt7	4.72448	0.29	6.2%
Galnt9	9.468436	0.38	4.0%
Galntl1	32.2283	0.68	2.1%
Galntl2	0.0053373	0.01	223.6%
Galntl4	4.323084	0.40	9.3%
Galntl5	0	0.00	
Galp	0	0.00	
Galr1	0.5353272	0.16	30.6%
Galr2	0.4362224	0.10	23.6%
Galr3	2.3909507	5.21	217.9%
Galt	7.62355	0.52	6.9%
Gamt	2.180472	0.37	16.8%
Gan	0.8398926	0.19	22.9%
Ganab	62.07152	1.21	2.0%
Ganc	1.977514	0.48	24.3%
gap1m	0.2064228	0.08	37.6%
Gap43	299.2398	29.71	9.9%
Gapdh	0.475571	0.06	12.8%
Gapdhs	0.67850104	1.43	210.9%
Gapvd1	18.56324	0.72	3.9%
Garnl1	21.6351	1.05	4.9%
Garnl3	15.91916	1.40	8.8%
Garnl4	10.094832	0.98	9.7%
Gars	77.15952	4.79	6.2%
Gart	12.41118	0.79	6.3%
Gas1	3.496686	0.76	21.8%
Gas2	2.083158	0.25	12.1%
Gas2l1	9.407812	0.59	6.3%
Gas2l3	0.5531388	0.12	21.1%
Gas5	40.51308	1.12	2.8%
Gas6	35.3592	1.95	5.5%
Gas7	8.398166	0.59	7.0%
Gas8	8.604858	0.95	11.0%
Gast	0.05295208	0.06	106.7%
Gata1	0	0.00	
Gata2	0.276714	0.05	19.1%
Gata3	0.1969484	0.07	34.0%
Gata4	1.479318	0.39	26.6%
Gata5	0.04031812	0.02	39.7%
Gata6	0.5956948	0.28	47.2%
Gatad1	22.759	1.56	6.8%

Gatad2a	8.420778	0.22	2.7%
Gatad2b	11.150096	1.02	9.2%
Gatm	4.98988	0.53	10.6%
Gats	17.7647	2.18	12.3%
Gba	11.92032	0.42	3.5%
Gba2	22.4109	1.27	5.7%
Gbas	28.82762	1.05	3.6%
Gbe1	6.661472	0.54	8.2%
Gbf1	35.85954	1.63	4.5%
Gbgt1	0.02995258	0.02	63.2%
Gbl	6.504472	0.37	5.7%
Gbp1	0.040396124	0.03	75.2%
Gbp2	0.09777392	0.03	33.8%
Gbp3	0.2479804	0.05	21.3%
Gbp4	0.026933318	0.01	41.3%
Gbp5	0.03040478	0.02	59.6%
Gbp6	0.3481972	0.05	12.9%
Gbx1	0.13169388	0.03	25.5%
Gbx2	4.034756	0.79	19.6%
Gc	0.04077304	0.03	71.6%
Gca	3.045298	0.15	4.9%
Gcap14	24.81396	1.35	5.4%
Gcat	3.7845452	1.99	52.5%
Gcc1	3.504652	0.14	4.1%
Gcc2	17.5832	0.80	4.6%
Gcdh	7.100846	0.31	4.3%
Gcet2	0.00231998	0.01	223.6%
Gcg	0	0.00	
Gcgr	0	0.00	
Gch1	0.4739992	0.10	21.9%
Gchfr	0.2516752	0.11	45.4%
Gck	0.0845152	0.04	43.1%
Gckr	0.02398144	0.02	69.1%
Gclc	14.46984	0.57	3.9%
Gclm	12.90426	0.47	3.7%
Gcm1	0	0.00	
Gcm2	0	0.00	
Gcn1l1	13.43698	0.33	2.5%
Gcn5l2	23.8708	1.50	6.3%
Gcnt1	0.3366714	0.06	17.4%
Gcnt2	1.439504	1.30	90.5%
Gcnt3	0.2246788	0.13	57.2%
Gcnt7	0.01309074	0.02	120.9%
Gcs1	9.485652	0.36	3.8%
Gcsh	13.9832	0.76	5.5%
Gda	3.003396	0.33	11.1%
Gdap1	51.75572	2.12	4.1%

Gdap1l1	40.5648	4.00	9.9%
Gdap2	8.448356	0.47	5.5%
Gde5	7.003876	1.29	18.4%
Gdf1	3.557334	1.40	39.3%
Gdf10	1.462646	0.19	13.0%
Gdf11	5.32109	0.40	7.6%
Gdf15	0.03391404	0.03	83.7%
Gdf2	0	0.00	
Gdf3	0	0.00	
Gdf5	0.01585462	0.01	60.1%
Gdf6	0.2303206	0.04	17.3%
Gdf7	0.224026	0.07	31.7%
Gdf9	0.2752424	0.07	25.6%
Gdi1	248.0038	7.57	3.1%
Gdi2	79.0935	4.61	5.8%
Gdnf	0.2153264	0.09	43.2%
GDNFR-alpha	0.1364784	0.02	15.2%
Gdpd1	31.9178	3.64	11.4%
Gdpd2	1.032976	0.14	13.1%
Gdpd3	0.2517484	0.08	33.2%
Gdpd4	0.00478676	0.01	137.6%
Gdpd5	3.49822	0.19	5.4%
Gem	0.3536542	0.12	33.0%
Gemin4	5.360504	1.61	30.0%
Gemin5	4.82942	0.25	5.2%
Gemin6	1.44099	0.19	13.3%
Gemin7	16.60822	0.94	5.6%
Gemin8	0.754589	0.21	27.3%
gene_154	0	0.00	
gene_17	0	0.00	
Gfap	78.63166	10.13	12.9%
Gfer	7.520268	0.27	3.5%
Gfi1	0.07226602	0.05	64.3%
Gfi1b	0.00293706	0.01	223.6%
Gfm1	17.49754	0.42	2.4%
Gfm2	8.024592	0.40	5.0%
Gfod1	5.46621	0.28	5.2%
Gfod2	11.18182	1.09	9.8%
Gfpt1	26.60422	1.17	4.4%
Gfpt2	1.376926	0.30	22.0%
Gfra1	34.71168	1.79	5.1%
Gfra2	14.21716	0.70	4.9%
Gfra3	0	0.00	
Gfra4	6.006388	0.47	7.8%
Gfral	0.01326234	0.01	92.6%
GFRalpha2	0.4547944	0.06	13.8%
Gga1	15.87692	0.79	5.0%

Gga2	8.436556	0.68	8.1%
Gga3	12.8129	0.52	4.1%
Ggcx	4.185456	0.07	1.8%
Ggh	5.50319	0.55	9.9%
Ggn	0.306816	0.09	30.0%
Ggnbp1	0.4165282	0.19	45.3%
Ggnbp2	29.58852	0.40	1.3%
Ggps1	27.45316	0.52	1.9%
Ggt1	0.252551	0.03	10.9%
Ggt6	0	0.00	
Ggta1	3.366074	0.47	13.9%
Ggtl3	27.02836	1.67	6.2%
Ggtla1	0.13953088	0.07	48.0%
Gh	0	0.00	
Ghdc	4.71372	0.40	8.4%
Ghitm	163.3514	3.28	2.0%
Ghr	7.52083	1.28	17.0%
Ghrh	0.01550454	0.03	223.6%
Ghrhr	0	0.00	
Ghrl	0.15323896	0.09	58.5%
Ghsr	0.6460762	0.15	23.9%
Gif	0.00506028	0.01	223.6%
Gig18	15.979418	5.75	36.0%
Gimap1	0.00751708	0.02	223.6%
Gimap3	0	0.00	
Gimap4	0	0.00	
Gimap5	0	0.00	
Gimap6	0.01474628	0.01	99.1%
Gimap7	0.01126882	0.02	138.7%
Gimap8	0.04571716	0.02	47.7%
Gimap9	0.03085864	0.03	98.2%
Gins1	1.1074664	0.13	11.9%
Gins2	1.439574	0.19	13.5%
Gins3	0.8601972	0.11	12.4%
Gins4	8.127518	0.52	6.4%
Gip	0	0.00	
Gipc1	18.44262	1.67	9.1%
Gipc2	0.07635074	0.04	48.3%
Gipc3	0.07780066	0.04	54.3%
Gipr	0.18140002	0.08	42.1%
Git1	33.18836	1.71	5.2%
Git2	6.037264	0.21	3.5%
Giyd2	5.432574	0.39	7.1%
Gja1	11.98724	0.88	7.3%
Gja10	0.01499248	0.01	96.6%
Gja12	0.7213248	0.13	18.0%
Gja3	0.04247952	0.03	68.5%

Gja4	1.0305702	0.14	13.5%
Gja5	0.04157338	0.03	74.3%
Gja6	0.01542794	0.01	92.5%
Gja7	1.92242	0.19	9.7%
Gja8	0.05629246	0.04	65.1%
Gja9	20.29452	2.42	11.9%
Gjb1	0.04696454	0.03	59.2%
Gjb2	0.09835264	0.02	19.9%
Gjb3	0.06063952	0.02	27.2%
Gjb4	0.00854692	0.01	141.7%
Gjb5	0.04817226	0.05	101.6%
Gjb6	0.16211074	0.05	31.6%
Gjc1	0.3719434	0.13	33.9%
Gje1	0.05643412	0.03	55.6%
Gk2	0.0723252	0.05	65.3%
Gk5	1.069749	0.14	13.0%
Gkap1	6.048156	0.20	3.3%
Gkn1	0.940883	0.35	37.6%
Gkn2	3.073994	0.89	29.1%
gl	0.15780138	0.04	24.5%
Gla	5.814124	0.47	8.0%
Glb1	12.59704	0.47	3.7%
Glb1l	1.805812	0.15	8.2%
Glb1l3	0.00426962	0.01	223.6%
Glcci1	4.36221	2.50	57.2%
Glce	4.960248	0.32	6.4%
Gldc	1.732948	0.09	4.9%
Gldn	0.04189436	0.03	72.5%
Gle1l	11.47666	0.62	5.4%
Glepp1	0.0402088	0.01	15.9%
Glg1	33.88738	2.90	8.5%
Gli1	1.1740368	0.40	34.4%
Gli2	1.2203872	0.32	26.1%
Gli3	0.427153	0.05	12.5%
Glipr1	0.8162362	0.27	32.9%
Glipr1l1	0	0.00	
Glipr1l2	0	0.00	
Glipr2	3.743964	0.52	14.0%
Glis1	0.3815612	0.11	28.4%
Glis2	3.98923	0.34	8.4%
Glis3	0.7132616	0.15	21.6%
Glmn	4.518108	1.47	32.6%
Glo1	32.63008	1.26	3.9%
Glod4	26.34868	2.15	8.2%
Glod5	0.5425574	0.14	25.8%
glp	1.762262	0.54	30.4%
Glp1	0.12969638	0.05	40.7%

Glp1r	1.936256	0.22	11.3%
Glp2r	0.08461172	0.03	32.3%
Glra1	12.848712	2.58	20.0%
Glra2	8.761032	1.08	12.3%
Glra3	5.173716	0.66	12.8%
Glra4	0.00837082	0.01	140.3%
Glrb	108.062	3.47	3.2%
Glrp1	0.0037756	0.01	223.6%
Glrx	33.67446	1.34	4.0%
Glrx2	12.9789	0.71	5.5%
Glrx3	9.667338	1.15	11.9%
Glrx5	36.71134	3.12	8.5%
Gls	51.04698	4.93	9.7%
Gls2	2.6258	0.49	18.8%
Glt1d1	7.688002	0.31	4.1%
Glt25d1	13.8785	0.67	4.8%
Glt25d2	0.6259238	0.04	5.9%
Glt28d2	0.1167906	0.05	40.2%
Glt6d1	0	0.00	
Glt8d1	13.249	0.51	3.9%
Glt8d2	0.033987199	0.02	63.9%
Glt8d3	4.348984	0.03	0.6%
Glt8d4	0.8822926	0.23	25.8%
Gltp	3.739802	0.27	7.2%
Gltscr1	1.91535	0.16	8.1%
Gltscr2	10.495514	1.04	9.9%
Glud1	41.0643	1.46	3.6%
Glul	55.55446	3.59	6.5%
Gluld1	0	0.00	
GluR_alpha3	4.949412	0.61	12.4%
GLUT4	0.09087702	0.01	9.1%
GLUTX1	1.5261056	0.96	62.8%
Glyat	0.03916006	0.04	106.4%
Glycam1	6.331066	0.88	13.9%
Gm1006	0	0.00	
Gm1008	0.01490812	0.01	61.5%
Gm1019	0	0.00	
Gm1040	5.778994	0.37	6.4%
Gm106	0.2135966	0.08	35.9%
Gm1060	1.36987	0.04	2.9%
Gm1070	0.05580724	0.04	70.8%
Gm1082	0.005560396	0.01	155.2%
Gm1123	0.00909952	0.01	137.3%
Gm114	6.37128	0.49	7.7%
Gm1141	0.07778598	0.05	69.5%
Gm1157	0.02157308	0.01	62.9%
Gm128	0.00728712	0.01	137.9%

Gm129	3.542376	0.26	7.4%
Gm1322	0.00369808	0.01	223.6%
Gm1337	0.012195258	0.01	77.5%
Gm136	0.00358484	0.01	223.6%
Gm1381	0	0.00	
Gm1527	0	0.00	
Gm156	0	0.00	
Gm1564	0.2587912	0.03	10.0%
Gm1568	2.472374	0.17	7.0%
Gm1574	0.00938068	0.01	138.1%
Gm1587	0	0.00	
Gm1604	0	0.00	
Gm1631	0	0.00	
Gm166	0.598028	0.14	22.6%
Gm1661	0.22388092	0.11	50.2%
Gm1673	30.27252	2.44	8.1%
Gm1679	0	0.00	
Gm1698	0	0.00	
Gm1961	0.00266916	0.01	223.6%
Gm1964	0.3206782	0.05	15.7%
Gm1965	0	0.00	
Gm1966	0	0.00	
Gm1967	0.10253548	0.05	49.4%
Gm1968	0.00518064	0.01	223.6%
Gm216	0.12358684	0.07	55.0%
Gm237	11.7288	0.79	6.7%
Gm239	0.010070376	0.01	63.8%
Gm249	0.0175243	0.03	162.6%
Gm266	0.07140422	0.04	51.6%
Gm273	0	0.00	
Gm2a	24.0632	2.00	8.3%
Gm317	0	0.00	
Gm323	17.44698	0.49	2.8%
Gm347	6.055234	0.14	2.3%
Gm382	0	0.00	
Gm397	0	0.00	
Gm410	0.02333402	0.03	127.0%
Gm414	0.00249374	0.01	223.6%
Gm428	0	0.00	
Gm436	0	0.00	
Gm443	0.0279407	0.02	69.9%
Gm444	1.380642	0.06	4.1%
Gm467	0	0.00	
Gm484	0	0.00	
Gm50	19.18424	0.54	2.8%
Gm52	1.305356	0.06	4.8%
Gm525	0.01999776	0.03	137.0%

Gm527	1.434182	0.15	10.5%
Gm561	14.3846	1.51	10.5%
Gm566	0.3431172	0.11	30.7%
Gm568	0.00896234	0.02	223.6%
Gm572	0.00418954	0.01	223.6%
Gm587	0.00219654	0.00	223.6%
Gm595	0.01071258	0.02	156.7%
Gm597	0	0.00	
Gm603	0.5254912	0.06	11.8%
Gm606	0.00587098	0.01	147.4%
Gm608	1.485472	0.14	9.3%
Gm609	0	0.00	
Gm614	0	0.00	
Gm623	0	0.00	
Gm628	0.03116618	0.01	21.7%
Gm632	18.527	0.84	4.6%
Gm648	0.02098572	0.02	99.7%
Gm672	19.46716	0.67	3.5%
Gm687	0.1507252	0.07	45.1%
Gm693	14.2006	1.59	11.2%
Gm694	0.0390171	0.03	87.2%
Gm71	9.446994	0.57	6.0%
Gm711	0.05404854	0.01	24.9%
Gm719	0.9478228	0.42	44.2%
Gm732	0	0.00	
Gm749	0	0.00	
Gm75	0	0.00	
Gm757	0	0.00	
Gm766	0.08281544	0.04	52.0%
Gm767	0	0.00	
Gm77	0	0.00	
Gm773	0	0.00	
Gm784	0.05939808	0.02	26.1%
Gm806	0.00230616	0.01	223.6%
Gm813	0	0.00	
Gm815	0	0.00	
Gm826	0.00622356	0.01	223.6%
Gm829	0	0.00	
Gm833	0	0.00	
Gm839	0	0.00	
Gm846	0.14345496	0.06	43.1%
Gm851	0	0.00	
Gm853	0	0.00	
Gm854	0.01192976	0.02	158.8%
Gm867	0.07044818	0.04	61.5%
Gm879	4.548258	0.34	7.5%
Gm884	0	0.00	

Gm885	0.33022362	0.70	213.0%
Gm889	0.03384512	0.03	103.1%
Gm9	0	0.00	
Gm904	0	0.00	
Gm906	0	0.00	
Gm920	0.00244974	0.01	223.6%
Gm93	0	0.00	
Gm94	0	0.00	
Gm941	0.6940748	0.19	27.6%
Gm944	4.66305	0.14	2.9%
Gm949	0	0.00	
Gm96	3.96536	0.28	7.1%
Gm960	1.410102	0.15	11.0%
Gm962	2.141016	0.15	6.9%
Gm967	2.981146	0.33	11.0%
Gm97	0	0.00	
Gm973	0.0649363	0.03	47.1%
Gm98	0.4059902	0.07	16.7%
Gm996	12.24424	1.14	9.3%
Gmcl1	9.416384	0.56	6.0%
Gmcl1l	0	0.00	
Gmds	7.490942	0.37	4.9%
Gmeb1	3.787716	0.15	4.1%
Gmeb2	3.257214	0.19	5.8%
Gmfb	74.96572	1.87	2.5%
Gmfg	0.00720234	0.02	223.6%
Gmip	2.815162	0.13	4.6%
Gmnn	1.573194	0.12	7.4%
Gmppa	13.26324	0.46	3.5%
Gmppb	5.232734	0.57	10.8%
Gmpr	12.78744	0.87	6.8%
Gmpr2	7.904978	0.37	4.7%
Gmps	20.51098	2.46	12.0%
Gna11	24.06466	1.57	6.5%
Gna12	9.191346	0.93	10.1%
Gna13	8.063152	0.51	6.4%
Gna14	0.2641544	0.04	16.7%
Gna15	0.01922236	0.01	63.5%
Gnai1	52.08852	1.12	2.2%
Gnai2	39.02984	2.66	6.8%
Gnai3	15.81876	1.53	9.7%
Gnal	48.4989	0.40	0.8%
Gnao1	156.5906	7.99	5.1%
Gnaq	67.24938	3.73	5.5%
Gna-rs1	32.71184	2.25	6.9%
Gnas	968.9778	44.77	4.6%
Gnat1	0.0290099	0.01	19.9%

Gnat2	0.06689278	0.03	43.6%
Gnat3	0.00523168	0.01	223.6%
Gnaz	24.62966	1.39	5.7%
Gnb1	304.996	12.59	4.1%
Gnb1l	1.0590704	0.13	12.3%
Gnb2	92.28672	7.97	8.6%
Gnb2l1	126.9248	11.56	9.1%
Gnb3	0.1664736	0.08	47.5%
Gnb4	9.337442	1.36	14.6%
Gnb5	35.49338	1.07	3.0%
Gne	9.274368	0.50	5.4%
Gng10	17.2869	2.70	15.6%
Gng11	0.592626	0.11	18.9%
Gng12	19.80012	0.92	4.6%
Gng13	4.819378	0.51	10.7%
Gng2	94.72332	4.47	4.7%
Gng3	183.1896	24.67	13.5%
Gng3lg	3.534468	1.31	37.2%
Gng4	32.15412	6.84	21.3%
Gng5	3.042404	0.56	18.4%
Gng7	6.345678	0.44	7.0%
Gng8	0.10808732	0.06	56.2%
Gngt1	0	0.00	
Gngt2	0	0.00	
Gnl2	15.99884	0.45	2.8%
Gnl3	12.06326	0.31	2.6%
Gnl3l	87.59038	2.64	3.0%
Gnmt	0.2176336	0.07	32.8%
Gnpat	17.42832	0.22	1.3%
Gnpda1	3.382642	0.33	9.9%
Gnpda2	8.301638	0.95	11.5%
Gnpnat1	2.90004	0.40	13.8%
Gnptab	21.69884	1.17	5.4%
Gnptg	14.09552	0.61	4.3%
Gnrh1	0.0681387	0.07	97.3%
Gnrhr	0	0.00	
Gns	23.99082	0.53	2.2%
GOBLIN	0.01963906	0.01	39.8%
GODZ	4.24894	0.78	18.5%
Golga1	5.78055	0.26	4.5%
Golga2	21.39542	0.42	2.0%
Golga3	9.84013	0.42	4.2%
Golga4	10.650162	0.90	8.5%
Golga5	7.075518	0.36	5.1%
Golga7	24.3626	1.93	7.9%
Golgb1	7.989198	0.44	5.5%
Golim4	4.364186	0.29	6.7%

Golm1	4.329096	0.82	18.8%
Golph3	40.92216	1.09	2.7%
Golph3l	6.806734	0.50	7.4%
Golt1a	0.00796592	0.01	98.3%
Golt1b	13.72294	0.56	4.0%
Gon4l	6.539042	0.16	2.4%
Gopc	25.26458	0.72	2.9%
Gorasp1	7.128856	0.25	3.6%
Gorasp2	39.69946	3.97	10.0%
Gosr1	18.21416	0.25	1.4%
Gosr2	30.5531	0.89	2.9%
Got1	172.5404	7.56	4.4%
Got1l1	0.3813028	0.08	20.5%
Got2	51.91826	1.62	3.1%
Gp1ba	0.5491644	0.10	18.4%
Gp1bb	4.762426	0.54	11.4%
Gp2	0	0.00	
Gp49a	0	0.00	
Gp5	0.09922854	0.03	28.6%
Gp6	0	0.00	
Gp9	0	0.00	
Gpa33	0.02938986	0.02	67.0%
Gpaa1	18.00454	1.20	6.7%
GPalpha_IIb	0.3056758	0.01	3.6%
Gpam	10.95208	0.51	4.6%
Gpatc1	0.20978128	0.09	44.2%
Gpatch1	3.447354	0.19	5.6%
Gpatch2	4.763186	0.25	5.2%
Gpatch3	1.544482	0.14	9.4%
Gpatch4	6.545776	0.94	14.3%
Gpbar1	0	0.00	
GPBP	1.0166234	0.42	41.1%
Gpbp1	25.8034	1.74	6.7%
Gpbp1l1	4.1927	0.43	10.2%
Gpc1	24.43506	1.51	6.2%
Gpc2	6.325904	0.59	9.3%
Gpc3	29.2244	5.90	20.2%
Gpc4	5.749848	0.58	10.1%
Gpc5	14.1286	0.70	4.9%
Gpc6	4.066	0.31	7.7%
Gpd1	0.7315756	0.13	17.4%
Gpd1l	15.02542	1.01	6.7%
Gpd2	9.019766	0.59	6.6%
Gper	0.01697862	0.02	112.5%
Gpha2	0	0.00	
Gphb5	0	0.00	
Gphn	42.36262	4.14	9.8%

Gpi1	123.594	8.29	6.7%
Gpihbp1	0	0.00	
Gpkow	9.61498	0.35	3.7%
Gpld1	1.507902	0.11	7.1%
Gpm6a	305.3164	5.48	1.8%
Gpm6b	82.98012	3.02	3.6%
Gpnmb	0.03255722	0.01	33.2%
Gpr1	0.10324344	0.03	31.0%
Gpr101	4.671002	0.70	15.0%
Gpr103	0.5372384	0.06	12.1%
Gpr107	16.66598	0.66	4.0%
Gpr108	5.1813	0.26	5.1%
Gpr109a	0	0.00	
Gpr110	0.00641876	0.01	92.5%
Gpr111	0.09342172	0.03	36.0%
Gpr112	0.000840496	0.00	223.6%
Gpr113	0.001849832	0.00	223.6%
Gpr114	0	0.00	
Gpr115	0.0916542	0.04	41.0%
Gpr116	0.015013432	0.01	49.8%
Gpr119	0.01643378	0.01	63.7%
Gpr12	1.564756	0.15	9.7%
Gpr120	0.03659332	0.03	93.3%
Gpr123	5.56674	0.35	6.4%
Gpr124	1.165454	0.10	8.8%
Gpr125	5.153548	0.26	5.1%
Gpr126	1.585254	0.07	4.6%
Gpr128	0	0.00	
Gpr132	0.06532192	0.04	60.9%
Gpr133	0.018065216	0.01	71.1%
Gpr135	1.87899	0.17	8.9%
Gpr137	52.86588	1.64	3.1%
Gpr137b	8.173586	0.37	4.5%
Gpr139	3.78927	0.45	11.9%
Gpr141	0.01077056	0.01	97.7%
Gpr142	0.00597348	0.01	223.6%
Gpr143	0.00397862	0.01	223.6%
Gpr146	0.6443384	0.09	14.3%
Gpr149	2.253526	0.20	8.9%
Gpr15	0.7610866	0.18	23.9%
Gpr150	0.3372416	0.11	31.4%
Gpr151	0.11532524	0.03	28.3%
Gpr152	0.02950182	0.01	36.6%
Gpr153	14.40144	1.06	7.4%
Gpr155	12.6707	0.53	4.2%
Gpr156	0.7823202	0.04	4.9%
Gpr157	0.9273026	0.09	9.6%

Gpr158	17.31018	1.05	6.1%
Gpr160	0.03681262	0.02	46.1%
Gpr161	3.43759	0.54	15.8%
Gpr162	36.2366	1.27	3.5%
Gpr165	1.2678992	0.38	29.9%
Gpr17	0.6258188	0.26	41.8%
Gpr171	0	0.00	
Gpr172b	6.212176	0.72	11.7%
Gpr173	4.915658	0.33	6.6%
Gpr174	0.00892298	0.01	92.8%
Gpr175	10.745206	1.33	12.4%
Gpr176	2.572182	0.21	8.2%
Gpr177	15.17904	1.08	7.1%
Gpr179	0.02934328	0.01	31.4%
Gpr18	0	0.00	
Gpr180	10.83974	0.98	9.0%
Gpr19	6.49814	0.50	7.7%
Gpr20	0.04151922	0.02	51.1%
Gpr21	0.274941	0.10	37.5%
Gpr22	12.79866	1.25	9.8%
Gpr23	1.2272238	0.25	20.0%
Gpr26	1.19272	0.15	12.2%
Gpr27	1.978794	0.37	18.8%
Gpr3	3.349588	0.34	10.1%
Gpr31c	0	0.00	
Gpr33	0	0.00	
Gpr34	0.01383414	0.02	140.2%
Gpr35	0.0543959	0.03	50.3%
Gpr37	0.7055882	0.11	15.6%
Gpr37l1	0.1052937	0.07	65.3%
Gpr39	0.06733698	0.04	52.2%
Gpr4	0.409118	0.09	22.7%
Gpr44	0.17073	0.02	12.5%
Gpr45	3.262892	0.10	3.1%
Gpr50	1.1996132	0.18	14.9%
Gpr55	0.02062642	0.03	127.0%
Gpr56	6.719562	0.50	7.5%
Gpr6	0.08532972	0.06	76.1%
Gpr61	5.47916	0.27	4.9%
Gpr63	1.218714	0.15	12.3%
Gpr64	0.9832718	0.05	4.8%
Gpr65	0.00478544	0.01	223.6%
Gpr68	2.234236	0.18	8.0%
Gpr75	3.188132	0.18	5.5%
Gpr77	0.02979864	0.03	92.3%
Gpr81	0	0.00	
Gpr82	0	0.00	

Gpr83	0.4906692	0.14	28.3%
Gpr84	0.04277424	0.02	54.9%
Gpr85	12.15224	1.16	9.5%
Gpr87	0	0.00	
Gpr88	3.61206	0.26	7.2%
Gpr89	11.94404	0.97	8.1%
Gpr92	0.02062274	0.03	155.1%
Gpr97	0.00783722	0.01	93.9%
Gpr98	0.1648266	0.01	7.0%
Gprasp1	191.2342	6.72	3.5%
Gprasp2	118.1822	4.34	3.7%
Gprc2a-rs5	0.0637298	0.03	43.4%
Gprc5a	0.12186512	0.05	44.2%
Gprc5b	18.57954	2.29	12.3%
Gprc5c	0.2125718	0.07	31.3%
Gprc5d	0.01536652	0.01	92.7%
Gprc6a	0.00524418	0.01	137.7%
Gprin1	20.1613	3.05	15.1%
Gprin3	1.955336	0.30	15.2%
Gps1	4.024082	0.18	4.5%
Gps2	5.482224	0.34	6.2%
Gpsm1	27.69176	2.08	7.5%
Gpsm2	1.620266	0.24	14.6%
Gpsm3	0.092657	0.05	56.5%
Gpsn2	275.9816	25.42	9.2%
Gpt1	1.639736	0.19	11.7%
Gpt2	13.20042	0.37	2.8%
Gpx1	31.3554	2.30	7.3%
Gpx2	0.2694988	0.08	29.8%
Gpx3	6.16591	0.71	11.5%
Gpx4	18.94876	1.90	10.0%
GPx4	0.2366172	0.09	38.9%
Gpx5	0.00398858	0.01	223.6%
Gpx6	0.03215128	0.04	129.2%
Gpx7	0.5122906	0.12	22.7%
Gramd1a	30.35	0.54	1.8%
Gramd1b	6.52752	1.94	29.7%
Gramd1c	0.15045566	0.06	39.8%
Gramd2	0.8144764	0.11	13.3%
Gramd3	1.73117	0.15	8.4%
Gramp3	0.02708682	0.01	49.6%
Grap	0.01114442	0.02	142.2%
Grap2	0	0.00	
Grasp	1.306694	0.16	12.4%
Grb10	42.8893	2.85	6.6%
Grb14	4.420896	0.42	9.5%
Grb2	43.48748	2.45	5.6%

Grb7	0.1586716	0.04	26.5%
GRC	0.335481	0.07	22.3%
Grcc10	69.01974	5.72	8.3%
Great	0	0.00	
Greb1	0.05466824	0.01	21.9%
Grem1	0.4512694	0.05	11.7%
Grem2	14.21266	0.48	3.4%
Grg4	0.03112128	0.01	18.5%
Grhl1	0.3251626	0.05	15.6%
Grhl2	0.026992794	0.02	82.8%
Grhl3	0.4272306	0.09	21.4%
Grhpr	9.746666	1.62	16.6%
Gria1	30.35608	0.80	2.6%
Gria2	60.77608	4.62	7.6%
Gria3	19.70934	1.96	9.9%
Gria4	67.54714	3.46	5.1%
Grid1	21.62912	0.95	4.4%
Grid2	11.0825	0.62	5.6%
Grid2ip	0.1561936	0.03	21.8%
Grifin	0.01960266	0.03	139.4%
Grik1	5.235484	0.54	10.4%
Grik2	9.172482	0.74	8.1%
Grik3	9.686086	0.97	10.0%
Grik4	6.51067	0.17	2.6%
Grik5	33.36404	1.33	4.0%
Grin1	122.381	6.18	5.0%
Grin2a	2.127506	0.28	13.2%
Grin2b	2.3164	0.66	28.6%
Grin2c	0.04316324	0.01	32.8%
Grin2d	7.605112	0.41	5.4%
Grin3b	0.6538906	0.10	15.1%
Grina	267.7604	11.70	4.4%
Grinl1a	102.43636	2.48	2.4%
Grip1	6.533838	0.86	13.1%
Grip2	7.741148	0.56	7.2%
Gripap1	21.03256	1.15	5.4%
Grit	8.406346	0.71	8.4%
Grk1	0.00396513	0.01	137.0%
Grk4	0.51786	0.08	14.8%
Grk5	2.120722	0.17	8.2%
Grk6	17.71076	1.17	6.6%
GRK6	2.9203294	1.95	66.6%
Grlf1	40.72074	1.76	4.3%
Grm1	2.443274	0.26	10.8%
Grm2	0.3390048	0.13	37.6%
Grm3	4.035222	0.67	16.6%
Grm4	9.94198	1.08	10.9%

Grm5	16.30998	1.72	10.5%
Grm6	0	0.00	
Grm7	18.03178	0.34	1.9%
Grm8	3.685954	0.15	4.1%
Grm8A	0.07063578	0.03	45.0%
Grn	22.46354	1.21	5.4%
Grp	1.588414	0.50	31.4%
Grpel1	12.17208	0.78	6.4%
Grpel2	5.243292	0.62	11.8%
Grpr	0.809517	0.18	22.0%
Grrp1	0.7027654	0.13	18.6%
Grsf1	46.50674	1.34	2.9%
Grtp1	1.532514	0.24	15.8%
Grwd1	5.92121	0.94	15.8%
Gsbs	0.12055546	0.04	35.6%
Gsc	0.10823006	0.04	37.8%
Gscl	0.02013542	0.02	95.6%
Gsdm1	0	0.00	
Gsdm2	0.03779352	0.04	105.5%
Gsdm3	0.00350166	0.01	223.6%
Gsdmdc1	0.07574196	0.04	57.1%
Gsdmdc2	0.0060025	0.01	223.6%
Gse1	7.868918	0.31	3.9%
Gsg1	0.0392721	0.03	76.8%
Gsg2	0.1498112	0.07	45.7%
Gsk3a	53.63812	3.21	6.0%
Gsk3b	59.93602	1.58	2.6%
Gsn	5.030608	0.49	9.7%
Gspt1	28.7742	1.22	4.3%
Gspt2	14.7455	0.79	5.3%
Gsr	14.20678	0.92	6.5%
Gss	4.764438	1.16	24.3%
Gsta1	0.1205304	0.09	74.5%
Gsta2	0.04398174	0.03	72.8%
Gsta3	0.2696346	0.12	44.1%
Gsta4	23.42582	1.03	4.4%
Gstcd	2.566818	0.31	11.9%
Gstk1	1.1255202	0.35	31.0%
Gstm1	16.2988	1.04	6.4%
Gstm2	0.7185984	0.31	42.6%
Gstm3	0.0412993	0.06	151.7%
Gstm4	3.103326	0.53	17.2%
Gstm5	17.25072	0.72	4.2%
Gstm6	0.1552204	0.06	36.0%
Gstm7	0.8067144	0.17	21.0%
Gsto1	27.5366	1.92	7.0%
Gsto2	0.20066782	0.09	44.3%

Gstp1	24.15676	1.66	6.9%
Gstp2	5.825104	0.73	12.5%
Gstt1	0.8476806	0.23	27.0%
Gstt2	0.3945734	0.06	16.4%
Gstt3	1.4415354	0.40	27.9%
Gstt4	0.00685378	0.02	223.6%
Gstz1	15.53824	1.13	7.3%
Gsx1	0.2391706	0.09	39.0%
Gsx2	0.05538532	0.03	60.5%
Gt4-1	1.4173506	0.50	35.5%
Gtdc1	9.329096	0.48	5.1%
Gtf2a1	6.875158	0.67	9.7%
Gtf2a1l	0.00782138	0.01	141.7%
Gtf2a2	17.71884	1.39	7.8%
Gtf2b	10.14522	0.37	3.6%
Gtf2e1	7.268688	0.48	6.6%
Gtf2e2	7.498716	0.30	4.0%
Gtf2f1	19.1732	0.55	2.8%
Gtf2f2	5.975696	0.73	12.2%
Gtf2h1	10.98682	0.48	4.4%
Gtf2h2	8.527836	0.63	7.4%
Gtf2h3	5.238332	0.36	6.8%
Gtf2h4	3.12294	0.31	10.0%
Gtf2h5	36.67018	1.84	5.0%
Gtf2i	41.00384	1.06	2.6%
Gtf2ird1	6.748048	0.39	5.7%
Gtf2ird2	3.641552	0.41	11.3%
Gtf3a	4.288346	0.99	23.2%
Gtf3c1	13.37816	0.59	4.4%
Gtf3c2	10.107466	0.32	3.2%
Gtf3c3	9.395312	0.36	3.8%
Gtf3c4	11.283136	0.82	7.2%
Gtf3c5	6.712426	0.61	9.1%
Gtf3c6	7.010768	1.08	15.5%
Gtl-13	0.0952143	0.02	22.2%
Gtl2	193.5778	33.03	17.1%
Gtl3	17.49138	1.10	6.3%
Gtlf3a	0.8658356	0.10	11.1%
Gtlf3b	3.176418	0.13	4.0%
Gtpbp1	11.94956	1.23	10.3%
Gtpbp10	1.402982	0.13	9.5%
Gtpbp2	13.0046	1.08	8.3%
Gtpbp3	6.349328	0.23	3.7%
Gtpbp4	12.7436	0.58	4.6%
Gtpbp5	4.975924	0.35	7.1%
Gtpbp6	17.49586	1.73	9.9%
Gtpbp8	9.476314	0.82	8.6%

Gtrgeo22	19.06198	1.87	9.8%
Gtse1	1.308078	0.08	6.2%
Guca1a	0.03613048	0.04	104.7%
Guca1b	0	0.00	
Guca2a	0	0.00	
Guca2b	0	0.00	
Gucy1a2	7.424394	1.33	17.9%
Gucy1a3	0.345682	0.04	10.9%
Gucy1b2	0.01011104	0.01	144.3%
Gucy1b3	27.377	0.80	2.9%
Gucy2c	0.06266834	0.02	35.0%
Gucy2d	0.00782348	0.02	223.6%
Gucy2e	0.02732746	0.02	63.7%
Gucy2f	0.004952586	0.00	94.2%
Gucy2g	0.0025493	0.01	223.6%
Guf1	10.674112	1.62	15.1%
Guk1	51.65906	3.23	6.2%
Gulo	0.01229692	0.01	109.1%
Gulp1	1.989888	0.33	16.6%
Gusb	3.749422	0.18	4.9%
Gvin1	0	0.00	
Gyg	16.73412	1.65	9.9%
Gyk	7.662742	0.34	4.5%
Gykl1	0.00316736	0.01	223.6%
Gyltl1b	0.15630688	0.09	54.8%
Gypa	0.03148058	0.04	115.9%
Gypc	0.2886548	0.07	25.1%
Gys1	6.651216	0.64	9.6%
Gys2	0.01635452	0.01	83.7%
Gzf1	9.310016	0.48	5.2%
Gzma	0	0.00	
Gzmb	0	0.00	
Gzmc	0.07629104	0.06	74.3%
Gzmd	0	0.00	
Gzme	0	0.00	
Gzmf	0.00409728	0.01	223.6%
Gzmg	0	0.00	
Gzmk	0.02135098	0.03	145.6%
Gzmm	0.3994456	0.13	32.5%
Gzmn	0	0.00	
H13	21.56134	1.70	7.9%
H19	0.12232386	0.05	39.6%
H1f0	0.02876812	0.01	42.4%
H1fnt	0.00334316	0.01	223.6%
H1foo	0	0.00	
H1fx	12.24604	1.18	9.6%
H28	0.10364044	0.04	42.0%

H2-Aa	0.01408562	0.02	142.2%
H2-Ab1	1.4655496	0.60	40.8%
H2afj	5.420242	0.50	9.3%
H2afv	0.709884	0.40	56.4%
H2afx	15.50824	0.77	5.0%
H2afy	27.00014	0.65	2.4%
H2afy2	7.866186	1.33	16.9%
H2afz	3.029172	0.31	10.1%
H2-B2	2.67893	1.12	41.9%
H2-Bl	5.383458	0.65	12.0%
H2-D1	28.94692	2.69	9.3%
H2-DMa	3.691872	0.37	9.9%
H2-DMb1	0.093708574	0.08	90.5%
H2-DMb2	0.07241612	0.08	105.3%
H2-Ea	0	0.00	
H2-Eb1	0.01793286	0.01	60.5%
H2-Eb2	0.01701868	0.01	64.0%
H2-GS14	0.05063308	0.01	24.3%
H2-K1	9.297162	0.75	8.1%
H2-Ke2	17.4693	1.81	10.4%
H2-Ke6	5.654	0.70	12.4%
H2-M1	0.01375974	0.02	138.6%
H2-M10.1	0	0.00	
H2-M10.2	0.03103296	0.02	68.2%
H2-M10.3	0	0.00	
H2-M10.4	0.01231648	0.02	138.6%
H2-M10.5	0.00374818	0.01	223.6%
H2-M10.6	0.00349616	0.01	223.6%
H2-M11	0	0.00	
H2-M2	0.0657702	0.07	99.8%
H2-M3	0.5389386	0.09	16.0%
H2-M5	0.3311066	0.10	29.8%
H2-M9	0	0.00	
H2-Oa	0.001076478	0.00	223.6%
H2-Ob	0	0.00	
H2-Q1	1.0746968	0.18	16.6%
H2-Q10	1.0630002	0.12	11.3%
H2-Q2	0.8635584	0.19	22.0%
H2-Q6	1.2499864	0.39	31.0%
H2-Q7	1.4453288	0.33	23.1%
H2-Q8	0.06880494	0.04	60.2%
H2-T10	0.827257	0.39	47.4%
H2-T22	1.1354034	0.17	14.8%
H2-T23	2.11885	0.68	32.1%
H2-T24	0.01204968	0.02	152.8%
H2-T3	0.04454878	0.01	22.3%
H2-T3-like	0.03168976	0.01	25.4%

H2-T9	0.06586762	0.03	40.1%
H2-Tw3	0.0568637	0.01	25.4%
H3f3a	5.628404	0.50	8.9%
H3f3b	53.2399	2.68	5.0%
H4	0	0.00	
H47	17.65712	1.06	6.0%
H6pd	3.060832	0.21	6.8%
Haao	0.06904732	0.03	47.0%
Habp2	0.04389274	0.03	68.7%
Habp4	37.99218	1.90	5.0%
hac1	1.2078262	0.34	28.4%
Hace1	7.550988	0.54	7.1%
Hacl1	1.0468574	0.17	15.8%
Hadh	6.425788	0.42	6.5%
Hadha	13.7054	0.52	3.8%
Hadhb	1.447222	0.14	9.9%
Hagh	26.50024	0.53	2.0%
Haghl	51.71802	2.03	3.9%
Hai2	1.43214	0.19	13.2%
Hal	0.02114014	0.03	132.4%
Hamp1	0	0.00	
Hamp2	0	0.00	
Hand1	0	0.00	
Hand2	0.5618852	0.07	12.0%
Hao1	0.01483416	0.01	96.7%
Hao3	0	0.00	
Hap1	172.6194	9.30	5.4%
Hapln1	9.252402	1.17	12.6%
Hapln2	0.2217164	0.08	38.1%
Hapln3	0.04536108	0.04	86.6%
Hapln4	11.684766	2.29	19.6%
Hars	49.8122	2.70	5.4%
Hars2	5.46439	0.19	3.6%
Has1	0.08212534	0.05	56.7%
Has2	0.2072608	0.05	26.1%
Has3	0.4695554	0.07	15.4%
Hat1	8.973958	0.60	6.7%
Hausp	3.090238	0.46	14.9%
Havcr1	0	0.00	
Havcr2	0.01091022	0.01	116.0%
Hax1	4.935802	0.62	12.6%
Hba-a2	0.0085597	0.02	223.6%
Hba-x	0	0.00	
Hbb-b1	0	0.00	
Hbb-bh1	0	0.00	
Hbb-y	0	0.00	
Hbegf	2.48635	0.17	7.0%

Hbld1	18.04894	1.29	7.1%
Hbp1	10.67884	0.82	7.7%
Hbp2	0.0509713	0.02	44.7%
Hbq1	0	0.00	
Hbs1l	18.25714	0.38	2.1%
Hbxip	14.20052	0.64	4.5%
Hc	0.01143728	0.02	138.9%
Hccs	9.96035	0.42	4.2%
Hcfc1	17.34184	0.65	3.8%
Hcfc1r1	41.54094	5.46	13.1%
Hcfc2	6.461322	0.49	7.5%
Hck	0.1868748	0.04	22.2%
Hcls1	0.02990758	0.02	83.5%
Hcn1	8.17776	0.53	6.5%
Hcn2	40.51356	1.12	2.8%
Hcn3	19.37066	2.40	12.4%
Hcn4	6.506896	0.62	9.5%
Hcrt	0.03372916	0.04	105.9%
Hcrtr1	1.569594	0.37	23.7%
Hcrtr2	3.302664	0.39	11.7%
Hcst	0.04795882	0.04	93.0%
Hdac1	0.6779666	0.09	13.9%
Hdac10	2.862462	0.20	7.0%
Hdac11	105.74856	3.98	3.8%
Hdac2	45.32918	2.42	5.3%
Hdac3	49.10018	1.30	2.6%
Hdac4	8.104434	1.34	16.6%
Hdac5	24.49906	0.78	3.2%
HDAC5	3.009836	0.28	9.3%
Hdac6	18.27982	0.46	2.5%
Hdac7a	10.44013	1.00	9.6%
Hdac8	3.067872	0.47	15.2%
Hdac9	2.630782	0.34	12.8%
Hdc	0.14013146	0.04	32.0%
Hddc2	9.305092	0.92	9.9%
Hddc3	19.21792	1.13	5.9%
Hdgf	37.62668	1.10	2.9%
Hdgfl1	0	0.00	
Hdgfrp2	16.62212	0.53	3.2%
Hdgfrp3	30.66196	2.35	7.7%
Hdh	9.475454	0.85	8.9%
Hdhd1a	0.0456256	0.04	91.3%
Hdhd2	20.17438	0.85	4.2%
Hdhd3	2.825242	0.30	10.5%
Hdlbp	50.5296	1.45	2.9%
Hdrpa	1.1831138	0.24	19.9%
Hdx	0.6535258	0.10	15.5%

Heatr1	2.98974	0.13	4.2%
Heatr2	1.461454	0.16	10.9%
Heatr3	12.66194	0.62	4.9%
Heatr5a	1.65272	0.05	2.9%
Heatr5b	20.7964	1.04	5.0%
Heatr6	4.297354	0.10	2.3%
Hebp1	4.289542	0.63	14.6%
Hebp2	1.406776	0.17	12.2%
Heca	7.293886	0.68	9.3%
Hectd1	15.9591	0.71	4.4%
Hectd2	4.963482	0.19	3.9%
Hectd3	10.57188	0.33	3.1%
Hecw1	17.86864	0.59	3.3%
Hecw2	6.770238	0.45	6.6%
Heg1	0.7306684	0.12	16.3%
Hel308	2.052754	0.22	10.8%
Helb	0.6543766	0.04	5.7%
helB	0.20455622	0.09	44.8%
HELG	4.683008	1.05	22.4%
Hells	2.420692	0.16	6.8%
Helt	0.00709836	0.01	139.0%
Helz	2.4678	0.32	13.0%
Hemgn	0	0.00	
Hemk1	5.656272	0.25	4.4%
Hemk2	2.923266	0.60	20.4%
HemT	0	0.00	
Hemt1	0.003713	0.01	223.6%
Hepacam	2.057656	0.36	17.5%
Heph	0.3683908	0.05	12.8%
Hephl1	0.00751616	0.01	94.3%
Herc1	20.56652	0.95	4.6%
Herc2	9.24921	0.68	7.3%
Herc3	36.32114	1.15	3.2%
Herc4	4.861124	0.33	6.7%
Herc5	0.36629494	0.16	43.0%
Herpud1	26.7214	0.87	3.2%
Herpud2	14.37078	0.77	5.3%
Hes1	2.403316	0.51	21.3%
Hes2	0	0.00	
Hes3	0.00677194	0.01	139.7%
Hes5	0.5587346	0.11	20.2%
Hes6	13.13354	1.21	9.2%
Hes7	0.436461	0.16	36.3%
Hesx1	0	0.00	
Hexa	15.23596	0.84	5.5%
Hexb	15.56314	1.15	7.4%
Hexdc	9.371924	1.39	14.8%

Hexim1	10.365228	0.52	5.0%
Hexim2	8.434602	0.68	8.1%
Hey1	8.695812	0.74	8.5%
Hey2	0.933355	0.11	11.4%
Heyl	0.09270078	0.02	25.2%
Hfe	0.1647244	0.06	36.8%
Hfe2	0.00311822	0.01	223.6%
Hfm1	0.4038204	0.08	19.3%
Hgd	0.0255104	0.02	75.8%
Hgf	0.0505552	0.04	71.7%
Hgfac	0.02637474	0.02	76.8%
Hgfl	0.3691226	0.09	25.5%
Hgs	31.94546	1.18	3.7%
Hgsnat	17.93868	0.62	3.4%
Hhat	0.8595588	0.10	11.3%
Hhatl	0.03942234	0.04	99.5%
Hhex	0.186004	0.03	18.3%
Hhip	1.224054	0.06	5.2%
Hiat1	21.8783	1.40	6.4%
Hiatl1	12.04868	0.78	6.4%
Hibadh	22.31676	1.00	4.5%
Hibch	9.786824	0.40	4.1%
Hic1	2.131194	0.16	7.6%
Hic2	1.425556	0.11	7.5%
hide1	0.00213792	0.00	223.6%
Hif1a	34.86454	1.73	5.0%
Hif1an	16.92642	0.36	2.1%
Hif3a	0.3933952	0.11	27.7%
Higd1a	47.77784	3.05	6.4%
Higd1b	0	0.00	
Higd1c	0.02728141	0.02	78.3%
Higd2a	57.5104	4.93	8.6%
Hils1	0.00624292	0.01	223.6%
Hint1	166.527	9.68	5.8%
Hint2	11.7754	0.69	5.9%
Hint3	11.69512	0.73	6.2%
Hip1	7.149156	0.13	1.9%
Hip1r	10.4392	0.34	3.3%
Hip2	36.41992	1.32	3.6%
Hipk1	10.247954	0.98	9.6%
Hipk2	4.528392	0.72	16.0%
Hipk3	8.843986	0.66	7.4%
Hipk4	10.86425	0.71	6.6%
Hira	11.9438	0.61	5.1%
Hirip3	3.696026	0.29	8.0%
Hisppd1	10.001972	0.76	7.6%
Hisppd2a	40.26098	2.78	6.9%

Hist1h1a	0.02330722	0.04	159.7%
Hist1h1b	0.02350248	0.02	95.4%
Hist1h1c	4.780472	0.85	17.8%
Hist1h1d	0.05482844	0.04	72.0%
Hist1h1e	0.08234214	0.01	11.4%
Hist1h1t	0	0.00	
Hist1h2aa	0	0.00	
Hist1h2ab	0.02852	0.06	223.6%
Hist1h2ac	0.07436566	0.04	50.5%
Hist1h2ae	0.00628448	0.01	223.6%
Hist1h2af	0.01206122	0.03	223.6%
Hist1h2ag	0	0.00	
Hist1h2ah	0	0.00	
Hist1h2ai	0.0155702	0.03	223.6%
Hist1h2ak	0.01642526	0.04	223.6%
Hist1h2an	0	0.00	
Hist1h2ao	0	0.00	
Hist1h2ba	0.10004338	0.07	68.0%
Hist1h2bb	0.1048297	0.08	78.2%
Hist1h2bc	4.473932	0.72	16.2%
Hist1h2be	0.14494258	0.03	23.4%
Hist1h2bf	0.05550062	0.03	58.7%
Hist1h2bg	0.1057994	0.10	93.6%
Hist1h2bh	0	0.00	
Hist1h2bj	0	0.00	
Hist1h2bk	0	0.00	
Hist1h2bl	0.01323658	0.03	223.6%
Hist1h2bm	0	0.00	
Hist1h2bn	0	0.00	
Hist1h2bp	0.0358181	0.05	146.9%
Hist1h3a	0	0.00	
Hist1h3b	0	0.00	
Hist1h3c	0	0.00	
Hist1h3d	0.07436272	0.02	29.0%
Hist1h3e	0.04433102	0.07	160.6%
Hist1h3f	0	0.00	
Hist1h3g	0	0.00	
Hist1h3h	0.02168126	0.03	142.5%
Hist1h3i	0	0.00	
Hist1h4a	0.04687276	0.07	154.5%
Hist1h4b	0.0035836	0.01	223.6%
Hist1h4c	0.04065538	0.06	138.4%
Hist1h4d	0.06898664	0.07	99.6%
Hist1h4f	0	0.00	
Hist1h4h	0.15094916	0.09	58.9%
Hist1h4i	0.4980544	0.21	42.1%
Hist1h4j	0	0.00	

Hist1h4k	0.01579198	0.04	223.6%
Hist1h4m	0	0.00	
Hist2h2aa2	0.07395832	0.06	85.1%
Hist2h2ab	0.01657762	0.04	223.6%
Hist2h2ac	0.01675052	0.04	223.6%
Hist2h2bb	1.1726106	0.32	27.6%
Hist2h2be	1.0201432	0.20	19.4%
Hist2h3b	0	0.00	
Hist2h3c1	0.2843508	0.05	16.3%
Hist2h4	0.21226198	0.16	73.3%
Hist3h2a	19.42678	1.46	7.5%
Hist3h2ba	20.1809	2.86	14.2%
Hist3h2bb	0.1934278	0.10	51.9%
Hist4h4	0.05952744	0.06	99.9%
Hivep1	5.691692	0.79	13.9%
Hivep2	10.54007	0.55	5.3%
Hivep3	1.286032	0.11	8.4%
Hk1	97.7112	2.34	2.4%
Hk2	2.04391	0.67	32.6%
Hk3	0.00833094	0.01	139.4%
Hkdc1	0.2539476	0.08	30.6%
Hlcs	5.192824	0.33	6.3%
Hlf	3.681512	0.71	19.2%
HLS2	0.000156898	0.00	223.6%
Hltf	3.876428	0.37	9.6%
Hlx	0.4131782	0.05	12.9%
Hm13	4.349688	0.35	8.0%
Hmbox1	13.09812	1.16	8.9%
Hmbs	1.766016	0.10	5.4%
Hmcn1	0.08819154	0.02	22.4%
Hmcn2	0.01451768	0.00	26.0%
Hmg20a	19.63724	0.41	2.1%
Hmg20b	2.485512	0.23	9.3%
Hmga1	3.08845	0.19	6.0%
Hmga2	0.3541814	0.11	30.5%
Hmgb1	2.98551	0.33	11.0%
Hmgb2	0.5262468	0.12	22.8%
Hmgb2l1	4.644372	0.37	8.1%
Hmgb3	15.23108	1.94	12.7%
Hmgb4	0.00636722	0.01	223.6%
Hmgcl	6.728952	0.85	12.6%
Hmgcll1	2.845176	0.24	8.3%
Hmgcr	58.64318	5.25	9.0%
Hmgcs1	0	0.00	
Hmgcs2	0.12678102	0.07	58.9%
Hmgn1	18.11048	0.83	4.6%
Hmgn2	0.849792	0.11	12.6%

Hmgn3	16.84552	0.77	4.5%
Hmgx2	0.4633678	0.14	30.2%
Hmha1	0.031933634	0.02	74.5%
Hmmr	0.5357728	0.13	23.7%
Hmox1	7.217252	0.12	1.6%
Hmox2	26.847	1.32	4.9%
Hmx1	0.9202798	0.16	17.0%
Hmx2	0.2078472	0.06	30.9%
Hmx3	0.7382584	0.09	11.9%
Hn1	32.0519	5.40	16.9%
Hn1l	4.99993	0.91	18.2%
Hnf1a	0.4792426	0.06	12.9%
Hnf1b	0.254015	0.04	17.3%
Hnf4a	0.13133222	0.04	28.3%
Hnf4g	0.00480284	0.01	223.6%
Hnmt	1.75385	0.32	18.1%
hnRNP_A2/B1	9.925156	1.66	16.8%
hnRNP_X	2.02679	0.55	27.0%
Hnrnpa1	12.20122	1.01	8.3%
Hnrnpa2b1	84.08552	4.14	4.9%
Hnrnpc	31.51676	1.07	3.4%
Hnrnpl	46.12148	3.69	8.0%
Hnrnpr	17.02794	1.45	8.5%
Hnrnpu	76.53156	3.08	4.0%
Hnrpa3	7.320804	0.59	8.0%
Hnrpab	33.8264	2.98	8.8%
Hnrpd	29.79296	1.53	5.1%
Hnrpdl	32.29402	2.65	8.2%
Hnrpf	4.402642	0.35	7.9%
Hnrph1	68.30942	2.19	3.2%
Hnrph2	20.9855	0.48	2.3%
Hnrph3	14.63768	1.31	8.9%
Hnrpk	45.91198	3.01	6.6%
Hnrpll	14.22962	0.69	4.8%
Hnrpm	32.90312	0.39	1.2%
Hnrpr	0.772134	0.27	35.1%
Hnrpul1	20.63464	0.29	1.4%
Hnrpul2	45.04146	1.71	3.8%
Hnt	44.87764	1.51	3.4%
Homer1	8.60914	0.76	8.8%
Homer2	1.754774	0.23	13.1%
Homer3	4.001352	0.78	19.4%
Homez	2.993132	0.08	2.8%
Hook1	10.681398	1.23	11.5%
Hook2	6.034626	0.37	6.2%
Hook3	7.4885	0.80	10.7%
Hopx	0.5815266	0.26	45.0%

Hormad1	0	0.00	
Hormad2	0.00429356	0.01	223.6%
Hoxa1	0.9296568	0.23	25.1%
Hoxa10	0.1376375	0.06	42.9%
Hoxa11	0.002244661	0.00	199.7%
Hoxa13	0.2539634	0.10	38.9%
Hoxa2	3.636712	0.56	15.5%
Hoxa3	6.012596	0.48	8.0%
Hoxa4	6.697456	0.79	11.7%
Hoxa5	46.24906	2.90	6.3%
Hoxa6	13.2245	1.19	9.0%
Hoxa7	9.998272	0.50	5.0%
Hoxa9	1.111874	0.10	8.6%
Hoxb1	0.00456784	0.01	223.6%
Hoxb13	1.646034	0.30	18.5%
Hoxb2	5.374946	0.41	7.6%
Hoxb3	10.248434	1.05	10.3%
Hoxb4	3.506876	0.27	7.8%
Hoxb5	17.98204	0.91	5.0%
Hoxb6	15.2389	1.22	8.0%
Hoxb7	22.15044	2.48	11.2%
Hoxb8	77.03724	7.62	9.9%
Hoxb9	24.97388	3.21	12.9%
Hoxc10	1.08551	0.08	7.4%
Hoxc11	0	0.00	
Hoxc12	0	0.00	
Hoxc13	0.428542	0.05	11.6%
Hoxc4	33.72062	5.47	16.2%
Hoxc5	27.4799	4.27	15.5%
Hoxc6	45.83498	6.12	13.3%
Hoxc8	8.45258	1.20	14.2%
Hoxc9	2.181338	0.24	10.8%
Hoxd1	0.05303438	0.03	64.9%
Hoxd10	0.05069648	0.03	57.1%
Hoxd11	0.0159875	0.01	92.5%
Hoxd12	0	0.00	
Hoxd13	0.19673	0.02	9.2%
Hoxd3	9.370684	0.43	4.6%
Hoxd4	3.185102	0.31	9.6%
Hoxd8	9.10656	0.70	7.7%
Hoxd9	4.622364	0.40	8.7%
Hp	0.03349798	0.03	78.7%
Hp1bp3	40.45398	0.95	2.3%
Hpca	25.12444	4.15	16.5%
Hpcal1	70.1555	3.58	5.1%
Hpcal4	13.62906	0.96	7.0%
Hpd	0	0.00	

Hpdl	0.0996352	0.05	48.6%
Hpgd	0.15506724	0.04	25.6%
Hpn	0.5258202	0.16	29.6%
Hprt1	120.2308	1.74	1.4%
Hps1	2.025684	0.19	9.4%
Hps3	6.336126	0.34	5.4%
Hps4	5.730638	0.42	7.4%
Hps5	2.05463	0.17	8.2%
Hps6	2.66836	0.40	15.0%
Hpse	0.831225	0.24	28.3%
Hpx	0.14652584	0.08	51.6%
Hr	0.419268	0.09	21.4%
H-ras	1.0179286	0.27	26.4%
Hras1	55.23716	3.64	6.6%
Hrasls	0.3322404	0.09	26.7%
Hrasls3	1.450176	0.19	12.9%
Hrasls5	0.00558028	0.01	223.6%
Hrb	28.45262	1.36	4.8%
Hrbl	13.09018	1.57	12.0%
Hrc	0.0815347	0.03	38.2%
Hrg	0	0.00	
Hrh1	0.3033654	0.06	20.6%
Hrh2	0.11153496	0.04	32.4%
Hrh3	0.7619894	0.26	33.5%
Hrh4	0.00460664	0.01	223.6%
Hrk	4.200932	0.77	18.4%
Hrnr	0	0.00	
Hrsp12	19.26548	1.01	5.2%
Hs1bp3	1.675494	0.17	10.1%
Hs2st	1.1060096	0.58	52.1%
Hs2st1	17.1182	0.43	2.5%
Hs3st1	5.489628	1.26	22.9%
Hs3st2	9.027446	0.61	6.8%
Hs3st3a1	0.4499118	0.07	14.7%
Hs3st3b1	2.100494	0.12	5.7%
Hs3st5	12.76558	0.83	6.5%
Hs3st6	0.05390504	0.04	74.4%
Hs6st1	21.2624	1.80	8.5%
Hs6st2	35.45526	0.90	2.5%
Hs6st3	5.904166	0.61	10.3%
Hsbp1	176.6034	12.96	7.3%
Hscb	4.419292	0.42	9.6%
Hsd11b1	0.6091134	0.39	63.7%
Hsd11b2	0.06435242	0.04	54.8%
Hsd17b1	0.12131818	0.03	25.1%
Hsd17b10	13.66666	1.26	9.2%
Hsd17b11	4.199758	0.45	10.6%

Hsd17b12	46.31684	4.39	9.5%
Hsd17b13	0.00446862	0.01	223.6%
Hsd17b14	0.08902608	0.03	33.3%
Hsd17b2	0.0237639	0.02	99.2%
Hsd17b3	0.00510516	0.01	223.6%
Hsd17b4	15.08344	0.71	4.7%
Hsd17b6	0	0.00	
Hsd17b7	11.220786	1.80	16.1%
Hsd3b1	0	0.00	
Hsd3b2	1.274324	1.14	89.6%
Hsd3b3	0.43141392	0.53	122.4%
Hsd3b4	0	0.00	
Hsd3b5	0	0.00	
Hsd3b6	0	0.00	
Hsd3b7	5.556128	0.73	13.1%
Hsdl1	22.05466	0.43	2.0%
Hsdl2	10.80032	0.48	4.5%
Hsf1	11.246192	0.84	7.5%
Hsf2	12.47122	0.85	6.9%
Hsf2bp	0.3513796	0.08	21.8%
Hsf4	1.928682	0.33	16.9%
Hsf5	0.05983726	0.03	43.1%
Hsfy2	0	0.00	
Hsh2d	0.04627368	0.04	84.2%
Hsn2	3.529042	0.67	19.0%
Hsp110	39.92606	1.73	4.3%
Hsp25	0.09514456	0.05	54.3%
Hsp90aa1	88.14658	3.11	3.5%
Hsp90ab1	459.2732	23.42	5.1%
Hsp90b1	140.2132	6.93	4.9%
Hspa12a	82.38212	3.59	4.4%
Hspa12b	0.8250588	0.10	11.9%
Hspa14	12.53856	1.64	13.1%
Hspa1a	1.1942808	0.30	24.8%
Hspa1b	0.6004632	0.08	12.9%
Hspa1l	1.321942	0.10	7.6%
Hspa2	2.987694	0.46	15.3%
Hspa4	69.7078	2.29	3.3%
Hspa4l	36.81004	2.24	6.1%
Hspa5	142.484	6.39	4.5%
Hspa8	48.27552	2.91	6.0%
Hspa9	81.5536	4.05	5.0%
Hspb1	0.05619648	0.04	70.5%
Hspb2	0.1374536	0.31	223.6%
Hspb3	0	0.00	
Hspb6	4.41867	0.49	11.2%
Hspb7	0.001728326	0.00	223.6%

Hspb8	0.7586732	0.19	25.3%
Hspbap1	0.7870126	0.10	13.1%
Hspc171	19.76346	2.59	13.1%
Hspd1	43.14724	1.86	4.3%
Hspe	0.09296122	0.06	66.8%
Hspe1	24.28418	1.39	5.7%
Hspg2	3.435848	1.09	31.6%
Htatip	22.30184	0.67	3.0%
Htatip2	0.4011556	0.18	44.2%
Htatsf1	21.42972	2.94	13.7%
Htf9c	8.134992	0.17	2.1%
Htr1a	4.405996	0.27	6.2%
Htr1b	3.4554418	1.84	53.3%
Htr1d	0.2505624	0.06	23.0%
Htr1f	0.03416864	0.03	77.2%
Htr2a	6.485528	0.82	12.6%
Htr2b	0.01619778	0.02	139.2%
Htr2c	46.51112	5.15	11.1%
Htr3a	0.07764886	0.03	43.3%
Htr3b	0.02939726	0.03	86.5%
Htr4	1.772444	0.32	17.8%
Htr5a	0.7344406	0.03	3.7%
Htr5b	0.05585322	0.05	82.7%
Htr6	0.7168226	0.13	18.5%
Htr7	13.4136	0.80	6.0%
Htra1	3.876146	0.46	11.8%
Htra2	12.1696	0.69	5.7%
HtrA2	3.647658	0.24	6.6%
Htra3	0.1830822	0.08	42.9%
Htra4	0.04526718	0.01	16.1%
Hunk	1.160938	0.07	5.7%
Hus1	3.57905	0.17	4.8%
Hus1b	0.0085326	0.02	223.6%
Huwe1	26.03748	3.98	15.3%
Hvcn1	2.886178	0.21	7.1%
Hyal1	0.1582466	0.06	37.6%
Hyal2	2.831494	0.29	10.4%
Hyal3	0.12895	0.06	46.6%
Hyal4	0	0.00	
Hyal5	0	0.00	
Hydin	0.007980134	0.01	69.0%
Hyi	5.264928	1.28	24.3%
Hyls1	2.018148	0.21	10.4%
Hyou1	39.55788	2.22	5.6%
Hypc	3.392594	0.53	15.7%
I830077J02Rik	0.00485004	0.01	223.6%
Iah1	7.581676	0.95	12.5%

IAN5	0	0.00	
IAP	0.0119791	0.03	223.6%
Iapp	0.02349532	0.03	137.0%
Iars	17.15012	0.41	2.4%
Iars2	12.29682	0.69	5.6%
Ibrdc3	22.87626	3.72	16.3%
Ibsp	0	0.00	
Ibtk	3.383454	0.57	16.7%
Ica1	16.8565	1.09	6.5%
Ica1l	11.8227	1.19	10.1%
Icam1	0.6457282	0.18	27.5%
Icam2	0.0200618	0.03	141.7%
Icam4	0.11850572	0.08	64.6%
Icam5	3.147846	0.43	13.7%
Ick	4.755232	0.24	5.0%
Icmt	13.85012	0.52	3.7%
Icos	0.00488564	0.01	223.6%
Icosl	0.624345	0.10	16.5%
Ict1	16.10844	1.80	11.2%
Id1	1.705846	0.23	13.7%
Id2	10.862992	1.34	12.3%
Id3	4.17069	1.09	26.1%
Id4	31.5009	2.41	7.6%
Ide	27.54334	2.27	8.2%
Idh1	59.40266	10.50	17.7%
Idh2	12.6444	0.86	6.8%
Idh3a	111.8572	4.38	3.9%
Idh3b	96.88924	2.03	2.1%
Idh3g	64.9573	1.16	1.8%
Idi1	8.059934	1.50	18.6%
Idi2	0.00841578	0.01	154.2%
Ids	101.4859	1.78	1.8%
Idua	0.3430624	0.10	28.0%
Ier2	1.249484	0.14	11.3%
Ier3	5.908066	1.02	17.2%
Ier3ip1	32.18686	2.23	6.9%
Ier5	1.644636	0.14	8.4%
Ier5l	1.982818	0.20	10.3%
if3	0	0.00	
if5	0.000802998	0.00	223.6%
Ifi202b	0.20186712	0.12	58.7%
Ifi203	0.07806748	0.03	37.5%
Ifi204	0.0873205	0.08	88.0%
Ifi205	0.00216546	0.00	223.6%
Ifi27	0.23086192	0.17	71.7%
Ifi30	3.304346	0.30	9.0%
Ifi35	0.202891	0.05	26.8%

Ifi44	0.01744944	0.02	137.6%
Ifi47	0.01209818	0.01	94.5%
Ifih1	0.1769684	0.07	39.2%
Ifit1	0.2220392	0.10	45.7%
Ifit2	2.874002	0.28	9.7%
Ifit3	0.454101	0.14	31.8%
Ifitm1	0.2874498	0.19	67.4%
Ifitm2	10.533136	2.03	19.3%
Ifitm3	4.26954	1.32	31.0%
Ifitm5	0.04711848	0.03	67.5%
Ifitm6	0.08975096	0.07	72.8%
Ifitm7	0.1519114	0.02	12.4%
Ifltd1	0.11337054	0.03	30.8%
Ifna1	0	0.00	
Ifna11	0	0.00	
Ifna12	0	0.00	
Ifna13	0	0.00	
Ifna14	0	0.00	
Ifna2	0	0.00	
Ifna4	0	0.00	
Ifna5	0	0.00	
Ifna6	0	0.00	
Ifna7	0	0.00	
Ifna9	0.00766738	0.02	223.6%
Ifnab	0.00636132	0.01	223.6%
Ifnar1	13.80418	0.73	5.3%
Ifnar2	4.016648	0.31	7.7%
Ifnb1	0	0.00	
Ifne1	0	0.00	
Ifng	0	0.00	
Ifngr1	5.217078	0.68	12.9%
Ifngr2	31.02626	4.01	12.9%
Ifnk	0.04664774	0.06	135.8%
Ifnz	0	0.00	
Ifrd1	12.45022	1.69	13.6%
Ifrd2	1.042672	0.17	16.0%
Ifrg15	17.83452	1.28	7.2%
Ift122	4.292978	0.12	2.8%
Ift140	2.848476	0.11	3.7%
Ift172	9.948214	0.53	5.3%
Ift20	28.58164	1.51	5.3%
Ift52	13.21432	0.71	5.4%
Ift57	6.2653	0.44	7.0%
Ift74	5.51452	0.23	4.1%
Ift80	3.711212	0.25	6.8%
Ift81	8.44729	0.51	6.0%
Ift88	5.987948	0.32	5.3%

Ig[gamma]1/CH[gamma]1	0	0.00	
Igbp1	13.28932	0.36	2.7%
Igbp1b	0.01092806	0.01	94.2%
Igf1	1.317372	0.08	5.9%
Igf1r	3.025032	0.16	5.2%
Igf2	342.0172	77.70	22.7%
Igf2bp1	0.10669928	0.04	35.1%
Igf2bp2	1.098686	0.15	13.2%
Igf2bp3	1.397268	0.18	13.0%
Igf2r	7.953026	1.20	15.0%
Igfals	0.00229714	0.01	223.6%
Igfbp1	0.12286616	0.04	31.9%
Igfbp2	38.41952	6.56	17.1%
Igfbp3	20.97016	3.00	14.3%
Igfbp4	50.64636	8.71	17.2%
Igfbp5	67.26982	5.36	8.0%
Igfbp6	0.3911728	0.13	33.8%
Igfbp7	6.684174	1.51	22.6%
Igfbpl1	0.4904776	0.06	12.5%
Igfl3	0	0.00	
Igh	0.00273242	0.01	223.6%
Igha	0	0.00	
Igh-A_(1g2)	0.00861676	0.02	223.6%
Ighg	0.00271094	0.01	223.6%
Ighmbp2	3.720238	0.47	12.6%
Igj	0.08395646	0.10	116.3%
Igll1	0	0.00	
Igsf1	3.743024	0.37	9.8%
Igsf10	2.01863	0.39	19.3%
Igsf11	2.373692	0.17	7.0%
Igsf2	0.0024035	0.01	223.6%
Igsf21	10.882218	1.83	16.8%
Igsf3	7.548704	0.36	4.8%
Igsf5	0.40655122	0.85	208.1%
Igsf6	0.0232878	0.02	82.3%
Igsf8	37.60114	2.29	6.1%
Igsf9	0.9375634	0.10	10.3%
Igsf9b	8.255832	1.09	13.2%
Igtp	0.1989434	0.07	34.1%
IHABP	0.175436	0.02	10.2%
Ihh	0.5252312	0.13	24.9%
Ihpk1	43.68234	2.98	6.8%
Ihpk2	40.6205	2.56	6.3%
Ihpk3	0.01031054	0.01	141.8%
IIGP	0	0.00	
Iigp1	0.07510052	0.02	30.8%
Iigp2	0.2010206	0.06	31.4%

Ik	32.38702	0.66	2.0%
IkappaB-zeta	0.05055552	0.05	104.1%
Ikbkap	22.4041	1.61	7.2%
Ikbkb	6.104686	0.34	5.6%
Ikbke	0.1395396	0.03	18.3%
Ikbkg	9.528134	0.67	7.0%
Ikkb	0.013480718	0.01	74.5%
IKKbeta	1.5303254	0.47	30.7%
Ikzf1	0.02307183	0.02	66.0%
Ikzf2	0.2757252	0.09	31.0%
Ikzf3	0	0.00	
Ikzf4	2.446238	0.10	4.0%
Ikzf5	3.861918	0.07	1.8%
Il10	0	0.00	
Il10r2	0.2479184	0.07	28.6%
Il10ra	0.12079772	0.03	22.6%
Il10rb	0.499386	0.10	19.8%
Il11	0.13325494	0.06	42.4%
Il11ra1	7.519738	0.85	11.3%
Il11ra2	0.1337274	0.04	27.1%
Il12a	0.03270598	0.03	89.5%
Il12b	0	0.00	
Il12rb1	0.032595222	0.02	57.4%
Il12rb2	0.5163612	0.12	22.9%
Il13	0	0.00	
Il13ra1	0.9500358	0.10	10.3%
Il13ra2	0.02375804	0.02	80.7%
Il15	0.00550546	0.01	223.6%
Il15ra	0.4012508	0.10	24.3%
IL15ra	0.16129408	0.08	47.0%
Il16	0.07893838	0.02	19.1%
Il17a	0	0.00	
Il17b	0.0046658	0.01	223.6%
Il17c	0.0203612	0.05	223.6%
Il17d	2.528808	0.23	9.1%
Il17f	0.0050526	0.01	223.6%
Il17ra	1.171982	0.10	8.7%
Il17rb	0.020161048	0.02	82.1%
Il17rc	0.5488968	0.12	21.2%
Il17rd	1.429366	0.13	9.0%
Il17re	0.00681342	0.01	143.6%
Il18	1.896624	0.17	8.9%
Il18bp	1.505426	0.17	11.1%
Il18r1	0.00212438	0.00	223.6%
Il18rap	0	0.00	
Il19	0.01500362	0.02	137.0%
Il1a	0.05040272	0.03	64.1%

Il1b	0.00267354	0.01	223.6%
Il1e	0	0.00	
Il1f10	0	0.00	
Il1f5	0	0.00	
Il1f6	0	0.00	
Il1f8	0	0.00	
Il1f9	0	0.00	
Il1r1	0.18186228	0.07	38.0%
Il1r2	0.04433872	0.03	61.3%
Il1rap	1.931916	0.24	12.5%
Il1rapl1	1.1418992	0.20	17.1%
Il1rapl2	1.179124	0.09	7.9%
Il1rl1	0.003223318	0.00	141.3%
Il1rl2	0.05184	0.03	48.8%
Il1rn	0.0033323	0.01	223.6%
Il2	0	0.00	
Il20	0	0.00	
Il20ra	0.02238526	0.01	64.4%
Il20rb	0.1840006	0.04	20.9%
Il21	0.07033418	0.03	39.2%
Il21r	0.001737168	0.00	223.6%
Il22	0	0.00	
Il22ra1	0	0.00	
Il22ra2	0	0.00	
Il23a	0.02788478	0.03	95.0%
Il23r	0.0058673	0.01	137.0%
Il24	0	0.00	
Il25	0.13656004	0.08	60.9%
Il27	0	0.00	
Il27ra	0.03788462	0.02	51.6%
Il28	0	0.00	
Il28ra	0.10007138	0.00	4.1%
Il2ra	0.016420608	0.01	58.6%
Il2rb	0.0292946	0.02	74.2%
Il2rg	0.05772876	0.04	65.8%
Il3	0.00645536	0.01	223.6%
Il31	0	0.00	
Il31ra	0.03802042	0.03	66.3%
Il33	2.904852	0.32	11.2%
Il3ra	1.477606	0.24	16.3%
Il4	0.01807012	0.02	137.6%
IL-4	0.01126678	0.03	223.6%
Il4i1	1.9725312	1.15	58.2%
Il4ra	0.2378768	0.04	18.2%
Il5	0.07121488	0.03	43.9%
Il5ra	0	0.00	
Il6	0	0.00	

Il6ra	0.09349398	0.04	41.7%
Il6st	15.59752	0.85	5.4%
Il7	0.07312748	0.01	14.8%
Il7r	0	0.00	
Il8ra	0.0052464	0.01	223.6%
Il8rb	0	0.00	
Il9	0	0.00	
Il9r	0.004409012	0.01	139.4%
Ildr1	0.05549712	0.04	63.9%
Ilf2	35.67386	2.16	6.1%
Ilf3	25.41112	0.64	2.5%
Ilk	12.87344	0.67	5.2%
Ilkap	15.79872	1.28	8.1%
Iltifb	0	0.00	
IL-TIFb	0	0.00	
Ilvbl	13.50622	0.64	4.7%
Immp1l	1.226986	0.23	18.8%
Immp2l	1.97975	0.30	15.1%
Immt	56.0122	2.24	4.0%
Imp3	9.927804	0.97	9.8%
Imp4	5.182924	0.38	7.4%
Impa1	16.32212	0.75	4.6%
IMPA1	0.2125628	0.05	23.6%
Impa2	2.164	0.34	15.7%
Impact	59.48332	3.16	5.3%
Impad1	43.8916	2.89	6.6%
Impdh1	27.54612	1.18	4.3%
Impdh2	2.435838	0.13	5.5%
Impg1	0	0.00	
Impg2	0.11139454	0.04	32.5%
Importin9	8.618276	1.10	12.8%
Ina	129.8978	6.86	5.3%
Inadl	3.477332	0.34	9.7%
iNAT	0	0.00	
Incenp	3.872744	0.22	5.6%
Indo	0.04515364	0.03	71.1%
Indol1	0.4049256	0.13	32.8%
Ing1	9.93821	0.72	7.3%
Ing2	3.104576	0.24	7.7%
Ing3	3.84464	0.26	6.7%
Ing4	13.63246	0.46	3.3%
Ing5	3.083712	0.12	3.8%
Inha	14.48132	1.24	8.6%
Inhba	0.3830086	0.07	18.1%
Inhbb	3.042338	0.25	8.1%
Inhbc	0.0097195	0.01	139.1%
Inhbe	0.00235254	0.01	223.6%

Inmt	0.04028212	0.02	44.8%
Inoc1	3.547032	0.19	5.5%
Inpp1	10.031336	0.51	5.1%
Inpp4a	23.30794	1.76	7.6%
Inpp4b	6.298358	0.35	5.5%
Inpp5a	12.08288	0.26	2.2%
Inpp5b	5.979084	0.27	4.5%
Inpp5d	0.02338968	0.01	50.8%
Inpp5e	7.65925	0.48	6.2%
Inpp5f	283.6812	13.17	4.6%
Inppl1	3.777892	0.27	7.2%
Ins1	0	0.00	
Ins2	0	0.00	
Insc	0.05623382	0.02	38.6%
Insig1	48.59596	2.21	4.5%
Insig2	21.56258	0.97	4.5%
Insl3	0.8288294	0.25	30.2%
Insl5	0.17882558	0.10	56.2%
Insl6	0.3500988	0.12	34.7%
Insm1	0.6678162	0.09	13.6%
Insm2	0.11460398	0.02	21.2%
Insr	4.647012	0.30	6.4%
Insrr	0.001996418	0.00	137.4%
Ints1	17.2032	0.77	4.5%
Ints10	15.16468	0.48	3.2%
Ints12	3.408282	0.16	4.8%
Ints2	4.240458	0.13	3.2%
Ints3	17.16564	0.35	2.0%
Ints4	9.134466	0.55	6.1%
Ints5	9.56064	0.36	3.7%
Ints6	4.897584	0.34	6.9%
Ints7	4.36396	0.11	2.6%
Ints8	7.325058	1.42	19.4%
Ints9	3.209808	0.15	4.8%
Intu	0.9734474	0.08	8.3%
inv	0.04363742	0.01	31.6%
Invs	2.325128	0.22	9.6%
Ip6k1	25.95338	1.55	6.0%
Ipmk	6.338958	0.47	7.5%
Ipo11	21.91738	1.89	8.6%
Ipo13	11.68212	0.91	7.8%
Ipo4	30.51188	0.14	0.5%
Ipo7	45.30282	1.46	3.2%
Ipo8	6.399346	0.42	6.5%
Ipo9	40.1393	0.85	2.1%
Ipp	4.044348	0.47	11.6%
Ippk	3.98533	0.23	5.9%

IQArfGEF	0.6821106	0.14	21.1%
Iqca	0.01953498	0.01	59.6%
Iqcb1	9.592426	0.79	8.2%
Iqcc	1.980452	0.18	9.2%
Iqcd	0.375934	0.06	16.1%
Iqce	3.485268	0.15	4.2%
Iqcf1	0.03278064	0.05	141.7%
Iqcf3	0	0.00	
Iqcf4	0	0.00	
Iqcf5	0.01266544	0.03	223.6%
Iqcg	1.0848102	0.11	10.5%
Iqch	0.04053066	0.04	90.2%
Iqck	3.065304	1.35	44.1%
Iqgap1	2.541792	0.24	9.4%
Iqgap2	0.9910432	0.16	15.8%
Iqgap3	0.2450434	0.08	32.6%
Iqsec2	17.01102	1.22	7.2%
Iqsec3	10.062654	1.12	11.1%
Iqub	0.1892544	0.05	26.8%
Iqwd1	9.906278	0.43	4.4%
Irak1	3.64065	0.26	7.1%
Irak1bp1	7.10984	0.36	5.0%
IRAK1-S	0.7358252	0.25	34.4%
Irak2	0.3372552	0.06	16.9%
Irak3	0.2260224	0.05	22.0%
Irak4	0.7994016	0.09	11.5%
ire1_alpha	0.16844362	0.06	33.0%
Ireb2	25.19384	1.33	5.3%
Irf1	0.5274482	0.12	23.0%
Irf2	6.192036	0.25	4.1%
Irf2bp1	18.32206	1.33	7.3%
Irf3	1.648208	0.26	15.5%
Irf4	0.0647794	0.01	19.7%
Irf5	1.90928	0.31	16.2%
Irf6	0.0512171	0.05	94.0%
Irf7	0.05285626	0.04	73.2%
Irf8	0.16181	0.05	29.0%
Irg1	0.04299916	0.02	50.9%
Irgc1	0.00320656	0.01	223.6%
Irgm	1.1640124	0.26	22.0%
Irgq	20.0752	0.66	3.3%
Irs1	5.788804	0.23	4.0%
Irs2	8.482756	0.63	7.5%
Irs3	0.0082575	0.02	223.6%
Irs4	1.656216	0.07	4.5%
Irx1	2.590404	0.20	7.6%
Irx2	2.908426	0.17	5.9%

Irx3	1.695456	0.08	4.6%
Irx4	0.1927746	0.03	16.4%
Irx5	1.0686656	0.15	14.3%
Irx6	0.04234056	0.03	72.8%
Isca1	28.3722	1.34	4.7%
Iscu	14.6301	1.40	9.5%
Isg12	0.02872778	0.03	92.1%
ISG12a	0.2359064	0.11	44.7%
Isg15	0.08635878	0.03	35.0%
Isg20	0.13732768	0.09	65.7%
Isg20l1	12.51134	1.56	12.5%
Isg20l2	3.197268	0.14	4.2%
Isgf3g	2.934484	0.26	8.7%
Isl1	0.3544452	0.12	32.9%
isl1-beta	0.04345392	0.03	77.0%
Isl2	0.7230194	0.13	18.4%
Islr	9.24777	1.83	19.8%
Islr2	0.9259116	0.09	9.2%
Isoc1	32.1505	2.22	6.9%
Isoc2b	1.4465	0.26	18.3%
Isx	0.17212622	0.07	39.2%
Isy1	8.089688	0.60	7.4%
Isyna1	1.87925	0.37	19.6%
IsynA1	0.4317216	0.16	37.3%
Itch	10.67393	0.84	7.8%
Itfg1	83.99078	2.03	2.4%
Itfg2	5.519288	0.44	8.0%
Itfg3	5.49711	0.36	6.6%
Itga1	0.9542178	0.36	37.2%
Itga10	0.3149314	0.06	17.8%
Itga11	0.2195578	0.03	11.9%
Itga2	0.04077366	0.01	36.3%
Itga2b	0.2641954	0.10	36.1%
Itga3	10.850646	0.65	6.0%
Itga4	0.4259728	0.18	42.1%
Itga5	1.327192	0.15	11.1%
Itga6	2.486978	0.16	6.5%
Itga7	1.0272628	0.10	9.5%
Itga8	0.5242746	0.14	26.2%
Itga9	0.7392398	0.11	14.4%
Itgad	0.03820642	0.01	31.7%
Itgae	0.04035804	0.01	36.6%
Itgal	0.01593859	0.01	56.9%
Itgam	0.02372866	0.01	44.9%
Itgav	3.829466	0.36	9.3%
Itgax	0	0.00	
Itgb1	30.35722	1.67	5.5%

Itgb1bp1	10.005866	0.92	9.2%
Itgb1bp2	0.05115822	0.04	84.8%
Itgb1bp3	0.16645444	0.07	41.6%
Itgb2	0.020943696	0.01	43.0%
Itgb2l	0	0.00	
Itgb3	0.345552	0.09	26.2%
Itgb3bp	0.8309498	0.11	13.1%
Itgb4	0.874432	0.29	32.6%
Itgb5	3.621062	0.27	7.5%
Itgb6	0.13851674	0.08	55.0%
Itgb7	0.1175656	0.04	35.6%
Itgb8	3.414712	0.20	5.8%
Itgbl1	0.4910754	0.11	22.9%
Itih1	0	0.00	
Itih2	0.0409581	0.02	49.0%
Itih3	0.10028402	0.02	20.4%
Itih4	0.009962254	0.01	60.6%
Itih5	0.6094396	0.16	26.9%
Itk	0.282493	0.06	21.3%
Itln1	0.00595998	0.01	223.6%
Itm2a	33.36792	5.87	17.6%
Itm2b	154.2624	4.53	2.9%
Itm2c	86.25926	4.79	5.6%
Itpa	10.638484	0.81	7.6%
Itpk1	10.76682	0.51	4.8%
Itpka	1.1548142	0.17	14.6%
Itpkb	3.119272	0.47	15.2%
Itpkc	2.560042	0.20	7.7%
Itpr1	1.809106	0.32	17.9%
Itpr2	0.3443196	0.06	18.8%
Itpr3	0.313276	0.04	12.0%
Itsn	1.8846556	1.90	100.6%
Itsn1	14.3344	0.97	6.8%
Itsn2	9.035954	0.71	7.9%
Ivd	12.66026	0.85	6.7%
Ivl	0.00612951	0.01	94.2%
Ivns1abp	32.71346	2.39	7.3%
Iws1	5.209778	0.45	8.6%
Iyd	0.0780406	0.05	60.6%
Izumo1	0.00836568	0.01	137.0%
J00476	0	0.00	
Jag1	1.507818	0.08	5.2%
Jag2	6.67403	0.30	4.5%
Jagn1	28.02816	1.31	4.7%
Jak1	34.04226	1.40	4.1%
Jak2	8.41986	0.32	3.9%
Jak3	2.451762	0.53	21.5%

Jakmip1	15.30174	1.33	8.7%
Jam2	5.34416	0.62	11.5%
Jam3	4.029138	0.30	7.5%
Jarid1a	8.254486	0.85	10.3%
Jarid1b	13.21702	0.18	1.4%
Jarid1c	8.51319	0.47	5.6%
Jarid1d	0.001501706	0.00	223.6%
Jarid2	9.752652	0.44	4.5%
Jazf1	25.45996	3.32	13.0%
Jbp1	0.5326082	0.27	51.0%
Jemm2	1.3477084	0.47	35.2%
Jhdm1d	1.716302	0.39	22.5%
Jmjd1a	6.625554	0.90	13.5%
Jmjd1b	6.631612	0.24	3.6%
Jmjd1c	5.496976	0.31	5.6%
Jmjd2a	12.06982	0.33	2.7%
Jmjd2b	15.13968	0.62	4.1%
Jmjd2c	4.271086	0.31	7.2%
Jmjd2d	0.7762688	0.08	9.7%
Jmjd3	4.925884	0.34	6.9%
Jmjd4	9.584726	0.64	6.7%
Jmjd5	0.8728996	0.13	15.4%
Jmjd6	6.000408	0.51	8.5%
Jmy	7.815668	0.77	9.8%
Jmy-pending	0.8277894	0.46	56.1%
jnk1	1.899742	0.60	31.4%
Jnk2	1.341798	0.23	17.4%
Josd1	17.57358	0.41	2.3%
Josd2	4.00509	0.37	9.3%
Josd3	5.909332	2.91	49.3%
Jph1	0.384514	0.03	6.6%
Jph2	0.02444291	0.01	46.4%
Jph3	29.9432	2.32	7.7%
Jph4	23.5493	0.93	3.9%
JRAB	0.40269568	0.23	56.3%
Jrk	4.279026	0.24	5.7%
Jsap1	0.16867838	0.12	73.6%
JSAP2	0.0490314	0.04	78.0%
Jsrp1	0.03482744	0.04	106.0%
Jtb	12.12944	1.41	11.6%
Jtv1	14.6183	1.67	11.5%
Jub	0.758565	0.14	18.6%
Jun	17.68928	1.39	7.9%
Junb	1.517068	0.24	15.8%
Jund1	33.28984	1.88	5.7%
Jundm2	8.3553	0.32	3.8%
Jup	7.234868	0.61	8.5%

K230010J24Rik	0.262064	0.07	28.3%
Kalrn	2.067892	0.32	15.4%
Kap	0	0.00	
Kars	33.56846	3.13	9.3%
Katna1	2.305644	0.23	9.9%
Katnal1	13.36616	0.96	7.2%
Katnal2	0.969343	0.16	16.2%
Katnb1	9.87266	0.43	4.3%
Kazald1	3.425138	0.26	7.7%
Kbtbd10	0.02849234	0.02	75.7%
Kbtbd11	6.539234	0.28	4.3%
Kbtbd2	17.19744	1.10	6.4%
Kbtbd3	5.027936	0.26	5.2%
Kbtbd4	9.561844	0.52	5.4%
Kbtbd5	0.07917878	0.06	75.9%
Kbtbd7	10.036488	0.18	1.8%
Kbtbd8	0.8596908	0.08	9.1%
Kcc4	0.05104884	0.02	30.1%
Kcmf1	21.61346	1.03	4.8%
Kcna1	12.545514	1.64	13.1%
Kcna10	0.0070923	0.01	140.8%
Kcna2	11.830216	2.12	17.9%
Kcna3	3.742328	0.27	7.2%
Kcna4	3.978652	0.36	9.1%
Kcna5	0.1798156	0.05	25.6%
Kcna6	17.83032	1.09	6.1%
Kcna7	0.06758354	0.02	36.0%
Kcnab1	3.572846	0.50	14.1%
Kcnab2	45.5189	6.16	13.5%
Kcnab3	1.746928	0.32	18.5%
Kcnb1	4.940092	0.33	6.7%
Kcnb2	1.984998	0.21	10.7%
Kcnc1	27.47524	1.97	7.2%
Kcnc2	7.451352	0.92	12.3%
Kcnc3	16.22718	1.57	9.7%
Kcnc4	14.66868	0.60	4.1%
Kcnd1	4.461486	0.24	5.3%
Kcnd2	24.68834	0.48	1.9%
Kcnd3	7.408916	0.40	5.4%
Kcne1	0	0.00	
Kcne1l	0.08149988	0.02	24.8%
Kcne2	0.12873106	0.05	40.9%
Kcne3	0.08300642	0.05	55.6%
Kcne4	0.3513024	0.12	33.2%
Kcnf1	0.6274388	0.06	9.2%
Kcng1	0.1602316	0.05	30.2%
Kcng3	0.280286	0.05	19.5%

Kcng4	0.12132412	0.04	34.8%
Kcnh1	2.722196	0.12	4.3%
Kcnh2	20.17306	0.54	2.7%
Kcnh3	0.1767532	0.03	15.9%
Kcnh5	3.861928	0.82	21.1%
Kcnh6	1.616294	0.10	6.1%
Kcnh7	2.808316	0.41	14.7%
Kcnh8	1.0600552	0.23	21.6%
Kcnip1	36.00776	3.96	11.0%
Kcnip2	0.9029922	0.25	27.2%
Kcnip3	1.1927296	0.27	22.7%
Kcnip4	11.9422	0.89	7.4%
Kcnj1	0.009047	0.01	161.5%
Kcnj10	3.616434	0.31	8.7%
Kcnj11	2.991248	0.18	6.0%
Kcnj12	1.911308	0.19	10.1%
Kcnj14	0.1749056	0.02	11.7%
Kcnj15	0.025545572	0.02	60.5%
Kcnj16	0.152501	0.01	6.7%
Kcnj2	1.0451322	0.17	16.2%
Kcnj3	9.080082	0.08	0.9%
Kcnj4	0.1825862	0.01	2.7%
Kcnj5	0.012944338	0.02	125.8%
Kcnj6	1.814066	0.31	17.1%
Kcnj8	0.5837396	0.08	14.1%
Kcnj9	1.081008	0.04	3.6%
Kcnk1	17.281	0.41	2.3%
Kcnk10	3.595186	0.45	12.4%
Kcnk12	2.099354	0.25	11.8%
Kcnk13	0.10924222	0.03	24.2%
Kcnk15	0.006418	0.01	223.6%
Kcnk18	0	0.00	
Kcnk2	9.865842	0.31	3.1%
Kcnk3	10.190226	0.65	6.3%
Kcnk4	3.51152	0.19	5.5%
Kcnk5	0.1704828	0.03	20.1%
Kcnk6	0.2294656	0.08	34.7%
Kcnk7	0.00987944	0.02	223.6%
Kcnk9	226.8928	15.66	6.9%
Kcnma1	10.95116	1.17	10.7%
Kcnmb1	0	0.00	
Kcnmb2	2.018262	0.28	14.0%
Kcnmb4	5.790204	1.18	20.3%
Kcnn1	6.648372	1.43	21.5%
Kcnn2	7.401362	0.62	8.4%
Kcnn3	1.433814	0.17	11.8%
Kcnn4	0.00528758	0.01	137.1%

Kcnq1	0.08214246	0.03	35.4%
Kcnq2	36.70036	4.08	11.1%
Kcnq3	12.20046	0.72	5.9%
Kcnq4	0.4210534	0.02	5.6%
Kcnq5	4.864382	0.30	6.2%
Kcnrg	0.387775	0.10	27.0%
Kcns1	0.218773	0.06	29.0%
Kcns2	4.31547	0.25	5.8%
Kcns3	4.956708	0.22	4.4%
Kcnt1	5.095954	0.41	8.1%
Kcnt2	2.822154	0.30	10.6%
Kcnu1	0.06056174	0.04	62.8%
Kcnv1	1.0414226	0.15	14.8%
Kcnv2	0.02695174	0.01	26.2%
Kcp	0.06195212	0.02	33.5%
Kctd1	11.3031	1.02	9.0%
Kctd10	6.980386	0.43	6.2%
Kctd11	0.2272856	0.08	36.0%
Kctd12	18.21504	1.05	5.8%
Kctd12b	0.3315888	0.07	22.0%
Kctd13	27.57154	1.70	6.2%
Kctd14	0.03244672	0.02	67.1%
Kctd15	6.21877	0.62	9.9%
Kctd16	2.011994	0.26	12.7%
Kctd17	39.95754	2.50	6.3%
Kctd18	7.942172	0.47	6.0%
Kctd19	0.372594052	0.34	91.4%
Kctd2	15.76766	0.34	2.1%
Kctd20	18.73472	0.73	3.9%
Kctd21	4.283554	0.39	9.0%
Kctd3	17.78632	0.77	4.3%
Kctd4	0.3172642	0.02	7.3%
Kctd5	7.766682	0.76	9.8%
Kctd6	12.13702	0.62	5.1%
Kctd7	7.142344	0.26	3.6%
Kctd8	8.61007	0.55	6.3%
Kctd9	6.151976	0.79	12.8%
Kdelc1	4.68413	0.32	6.9%
Kdelc2	4.246206	0.50	11.8%
Kdelr1	31.18268	2.60	8.3%
Kdelr2	24.70266	2.08	8.4%
Kdelr3	3.440498	0.88	25.6%
Kdr	0.1808346	0.03	15.5%
Keap1	15.97076	0.63	3.9%
Keg1	0	0.00	
Kel	0	0.00	
Kepi	5.069998	0.52	10.3%

Kera	0.02501768	0.02	77.7%
Khdrbs1	34.9804	0.77	2.2%
Khdrbs2	4.673964	0.24	5.2%
Khdrbs3	24.57548	1.25	5.1%
Khk	4.643884	0.64	13.7%
Khsrp	15.71106	0.76	4.9%
Ki-67	0.5939992	0.10	16.1%
Kiaa0028	0.05123798	0.02	45.9%
KIAA0143	1.7410036	1.15	66.1%
Kiaa0146	0.018013476	0.01	28.5%
KIAA0150	0.2209396	0.10	45.0%
Kiaa0196	1.2077554	0.60	49.6%
Kiaa0240	8.742606	0.46	5.3%
Kiaa0241	15.03434	1.07	7.1%
Kiaa0286	0.4426768	0.09	20.9%
Kiaa0528	11.7251	0.39	3.3%
Kiaa0575	0.05162312	0.02	33.6%
Kiaa0819	4.73022	0.23	4.9%
Kiaa0841	0.5934064	0.20	34.2%
Kiaa0859	0.2729078	0.04	13.6%
Kiaa1152	2.271492	0.57	25.0%
KIAA1199_homolog	1.0086402	0.14	13.4%
Kiaa1279	0.17713646	0.15	86.5%
kiaa1322	0.1991207	0.25	127.0%
KIAA1440	3.650382	0.43	11.7%
KIAA1447	0.481556	0.24	50.6%
KIAA1768	6.654432	0.34	5.0%
Kiaa1815	0.03095024	0.01	19.8%
Kiaa2022	3.535542	0.41	11.5%
KIAA312	17.1679	2.71	15.8%
Kif11	1.028333	0.22	21.3%
Kif12	0.00212848	0.00	223.6%
Kif13a	7.420732	0.99	13.4%
Kif13b	1.2117638	0.21	17.2%
Kif14	0.11387142	0.02	17.4%
Kif15	0.3934174	0.11	27.1%
Kif16b	1.93316	0.14	7.3%
Kif17	1.396758	0.20	14.3%
Kif18a	0.5106644	0.08	16.6%
Kif19a	0.03117534	0.03	87.7%
Kif1a	174.5324	8.91	5.1%
Kif1b	92.71732	2.98	3.2%
KIF1B	0.12209564	0.10	85.3%
Kif1c	5.189464	0.51	9.8%
Kif20a	1.0052684	0.17	16.7%
Kif21a	64.26992	5.03	7.8%
Kif21b	18.49774	1.79	9.7%

Kif22	1.0894346	0.20	18.2%
Kif23	0.8411866	0.24	28.4%
Kif24	0.5757348	0.10	17.8%
Kif26a	0.2799016	0.03	10.9%
Kif26b	4.558762	0.66	14.6%
Kif27	0.2133864	0.02	11.1%
Kif2a	37.93378	2.28	6.0%
Kif2b	0	0.00	
Kif2c	0.4847796	0.10	20.8%
Kif3a	60.60896	2.79	4.6%
Kif3b	21.1099	0.89	4.2%
Kif3c	51.02318	1.90	3.7%
Kif4	0.4921322	0.09	19.1%
Kif5a	183.1146	6.30	3.4%
Kif5b	34.92734	3.30	9.5%
Kif5c	139.303	2.13	1.5%
Kif6	0.3715408	0.08	22.4%
Kif7	0.3264878	0.19	58.0%
Kif9	1.17662	0.08	6.8%
Kifap3	89.57536	2.03	2.3%
Kifc1	1.034661	0.11	10.5%
Kifc2	85.54218	3.47	4.1%
Kifc3	9.113986	0.18	1.9%
Kin	4.04207	0.13	3.2%
Kir3dl1	0	0.00	
Kir3dl2	0	0.00	
Kirrel	2.29883	0.29	12.7%
Kirrel2	0.02779162	0.01	28.7%
Kirrel3	7.9968	0.58	7.3%
Kiss1	0.013717012	0.02	157.3%
Kiss1r	3.205934	0.27	8.3%
Kit	7.293434	0.63	8.6%
Kitl	7.595762	0.25	3.3%
Kl	0.2987484	0.04	12.3%
Klb	0.2098514	0.05	24.7%
Klc1	192.1526	4.21	2.2%
Klc2	91.82156	3.97	4.3%
Klc3	0.9189312	0.79	85.8%
Klc4	5.53865	0.47	8.5%
Klf1	0.0322416	0.01	44.4%
Klf10	3.740078	0.38	10.1%
Klf11	1.686364	0.12	7.2%
Klf12	8.753742	0.85	9.7%
Klf13	3.253346	0.61	18.7%
Klf15	4.744664	0.46	9.6%
Klf16	5.75846	0.54	9.5%
Klf17	0.00223384	0.00	223.6%

Klf2	0.3730396	0.07	18.5%
Klf3	2.40656	0.36	15.1%
Klf4	1.1875724	0.28	23.3%
Klf5	1.481256	0.30	20.5%
Klf6	13.67214	1.06	7.8%
Klf7	4.778962	0.50	10.5%
Klf8	2.403168	0.38	15.9%
Klf9	7.813132	0.95	12.1%
Klhdc1	3.496246	0.26	7.4%
Klhdc2	68.08024	1.29	1.9%
Klhdc3	53.0642	1.97	3.7%
Klhdc4	5.758312	0.66	11.5%
Klhdc5	4.404336	0.17	3.9%
Klhdc6	0.03848932	0.02	55.4%
Klhdc7a	0.10135066	0.04	36.7%
Klhdc8a	2.345008	0.21	9.1%
Klhdc8b	3.215522	0.25	7.9%
Klhdc9	2.789928	0.39	14.1%
Klhl1	2.87199	0.18	6.1%
Klhl10	0.2000646	0.05	27.1%
Klhl11	17.50612	2.64	15.1%
Klhl12	12.54748	0.52	4.2%
Klhl13	43.68132	0.51	1.2%
Klhl14	2.025304	0.13	6.6%
Klhl15	3.722334	0.31	8.3%
Klhl17	8.55488	0.88	10.3%
Klhl18	6.41386	0.46	7.2%
Klhl2	16.34062	0.98	6.0%
Klhl20	8.785146	0.28	3.2%
Klhl21	2.55121	0.27	10.6%
Klhl22	31.59	0.78	2.5%
Klhl23	15.48746	1.18	7.6%
Klhl24	12.25588	1.22	10.0%
Klhl25	4.116776	0.16	4.0%
Klhl26	14.78654	1.01	6.8%
Klhl28	5.939856	0.67	11.3%
Klhl30	0.00442927	0.01	138.9%
Klhl31	0.0032858	0.01	223.6%
Klhl32	7.513978	0.43	5.7%
Klhl34	2.55015	0.33	13.0%
Klhl4	0.8850156	0.09	10.4%
Klhl5	7.511552	0.80	10.6%
Klhl6	0.0695557	0.03	46.7%
Klhl7	41.29336	1.31	3.2%
Klhl8	7.426984	0.31	4.2%
Klhl9	27.33622	0.86	3.2%
Klk1	0.00623552	0.01	223.6%

Klk10	0	0.00	
Klk11	0	0.00	
Klk12	0	0.00	
Klk13	0	0.00	
Klk14	0	0.00	
Klk15	0	0.00	
Klk1b1	0	0.00	
Klk1b11	0	0.00	
Klk1b16	0	0.00	
Klk1b21	0	0.00	
Klk1b22	0	0.00	
Klk1b24	0.02822674	0.03	102.7%
Klk1b27	0	0.00	
Klk1b3	0	0.00	
Klk1b4	0.00640892	0.01	223.6%
Klk1b5	0	0.00	
Klk1b8	0	0.00	
Klk1b9	0	0.00	
Klk4	0	0.00	
Klk5	0	0.00	
Klk6	0	0.00	
Klk7	0.016412	0.02	111.4%
Klk8	0.10811292	0.03	27.9%
Klk9	0.0193306	0.02	103.1%
Klkb1	0.01417624	0.00	19.5%
Klra1	0	0.00	
Klra10	0.00906926	0.02	223.6%
Klra12	0	0.00	
Klra13	0	0.00	
Klra15	0	0.00	
Klra17	0	0.00	
Klra18	0	0.00	
Klra19	0	0.00	
Klra2	0.00863908	0.01	137.3%
Klra21	0.00110657	0.00	223.6%
Klra22	0	0.00	
Klra23	0	0.00	
Klra29	0	0.00	
Klra3	0	0.00	
Klra4	0	0.00	
Klra5	0	0.00	
Klra6	0	0.00	
Klra7	0.00640822	0.01	223.6%
Klra8	0.00644722	0.01	223.6%
Klra9	0	0.00	
Klrb1a	0.01397518	0.02	137.0%
Klrb1b	0.002102882	0.00	91.8%

Klrb1c	0.006302356	0.01	91.7%
Klrb1d	0.00424336	0.01	223.6%
Klrb1f	0	0.00	
Klrb6	0.02652342	0.03	94.8%
Klrc1	0	0.00	
Klrc2	0	0.00	
Klrc3	0	0.00	
Klrd1	0	0.00	
Klre1	0	0.00	
Klrg1	0.12564022	0.09	71.6%
Klrg2	0.0606913	0.03	42.2%
Klri1	0.0462371	0.03	68.6%
Klri2	0	0.00	
Klrk1	0	0.00	
Kmo	0.00924876	0.01	137.0%
Kncn	0.03602154	0.01	25.5%
Kndc1	8.927734	0.77	8.6%
Kng1	0.0128574	0.01	58.4%
Kng2	0.05935106	0.03	47.6%
KNT	0.8912616	0.45	50.7%
Kntc1	0.1857108	0.06	30.7%
Kor3	16.60092	2.11	12.7%
Kox-1	0.08894858	0.02	19.0%
Kpc1	7.734512	1.13	14.7%
Kpna1	29.69014	2.55	8.6%
Kpna2	0.07173378	0.04	49.2%
Kpna3	25.42888	1.41	5.5%
Kpna4	24.44464	1.75	7.1%
Kpna6	20.24722	0.42	2.1%
Kpnb1	34.95314	1.07	3.0%
Kprp	0	0.00	
Kptn	7.42575	0.75	10.1%
Kras	24.3289	0.60	2.4%
KRAZ1	0.828784	0.16	19.8%
Krba1	7.11029	0.36	5.0%
Krc	3.019082	0.77	25.5%
Krcc1	3.953246	0.35	8.9%
Kremen1	1.091241	0.10	8.9%
Kremen2	0.09612176	0.04	41.6%
Kri1	6.4223	0.73	11.4%
Krit1	7.208686	0.35	4.9%
Krr1	8.22269	0.32	3.9%
Krt1	2.06888	0.20	9.6%
Krt10	1.726064	0.24	13.6%
Krt13	0.00331638	0.01	223.6%
Krt14	0.00829518	0.01	137.0%
Krt15	0	0.00	

Krt16	0	0.00	
Krt17	0	0.00	
Krt18	1.694172	0.40	23.4%
Krt19	0.7227396	0.19	25.7%
Krt2	0.00259062	0.01	223.6%
Krt20	0.02054728	0.02	94.9%
Krt222	7.17776	0.49	6.9%
Krt23	0.02372096	0.03	109.7%
Krt24	0.00306812	0.01	223.6%
Krt25	0.00352858	0.01	223.6%
Krt26	0.001945428	0.00	223.6%
Krt27	0	0.00	
Krt28	0.00527294	0.01	137.4%
Krt31	0	0.00	
Krt32	0.00274536	0.01	223.6%
Krt33a	0	0.00	
Krt33b	0.00459664	0.01	223.6%
Krt34	0	0.00	
Krt35	0	0.00	
Krt36	0.00218316	0.00	223.6%
Krt39	0	0.00	
Krt4	0	0.00	
Krt40	0.0080006	0.01	138.8%
Krt42	0.00841922	0.01	138.1%
Krt5	0.00221286	0.00	223.6%
Krt6a	0	0.00	
Krt6b	0	0.00	
Krt7	0.13236324	0.06	47.4%
Krt71	0.0022035	0.00	223.6%
Krt72	0	0.00	
Krt73	0.0143008	0.01	98.4%
Krt74	0	0.00	
Krt75	0.001701642	0.00	223.6%
Krt76	0.0027689	0.01	223.6%
Krt77	0.00641226	0.01	141.6%
Krt78	0.014788416	0.01	74.9%
Krt79	0	0.00	
Krt8	0.9978964	0.29	28.9%
Krt80	0.00701367	0.01	161.9%
Krt81	0	0.00	
Krt82	0	0.00	
Krt83	0	0.00	
Krt84	0	0.00	
Krt86	0	0.00	
Krt9	0.003686832	0.01	137.7%
Krtap12-1	0	0.00	
Krtap13	0	0.00	

Krtap13-1	0	0.00	
Krtap14	0	0.00	
Krtap15	0	0.00	
Krtap16.6	0	0.00	
Krtap16-1	0	0.00	
Krtap16-4	0	0.00	
Krtap16-5	0	0.00	
Krtap16-7	0	0.00	
Krtap16-9	0	0.00	
Krtap17-1	0	0.00	
Krtap3-1	0	0.00	
Krtap3-2	0	0.00	
Krtap3-3	0	0.00	
Krtap4-7	0	0.00	
Krtap5-1	0.00847208	0.02	223.6%
Krtap5-2	0.00747558	0.02	223.6%
Krtap5-4	0	0.00	
Krtap5-5	0.00492062	0.01	223.6%
Krtap6-1	0	0.00	
Krtap6-2	0	0.00	
Krtap8-2	0	0.00	
Krtap9-1	0	0.00	
Krtcap2	39.77806	3.07	7.7%
Krtcap3	0.4026766	0.12	30.0%
Krtdap	0	0.00	
Ksr1	3.886468	0.41	10.5%
Ksr2	7.630696	1.11	14.5%
Ktelc1	13.06116	0.89	6.9%
Kti12	4.453474	0.46	10.3%
Ktn1	11.776482	3.66	31.1%
Ky	0.4330818	0.05	11.2%
Kynu	0.05526426	0.04	71.9%
L1cam	58.55146	1.17	2.0%
L1td1	0.013795034	0.01	69.4%
L2hgdh	2.841162	0.19	6.8%
L3mbtl	4.909182	0.15	3.0%
L3mbtl2	10.93948	0.77	7.0%
L3mbtl3	7.131216	0.31	4.4%
L3mbtl4	0.3956998	0.09	22.6%
l7Rn6	16.36404	0.53	3.2%
Lace1	5.645796	0.29	5.1%
Lactb	2.712764	0.26	9.6%
Lactb2	3.519182	0.18	5.0%
Lad1	0.07877798	0.01	15.5%
Lag3	0.2276426	0.03	12.9%
Lage3	8.42018	0.92	10.9%
Lair1	0.2548362	0.07	27.2%

Lalba	0.0241698	0.05	223.6%
Lama1	6.583726	1.32	20.0%
Lama2	4.914096	0.90	18.2%
lama3	0.05572538	0.01	25.1%
Lama3	0.02098946	0.01	28.0%
Lama3B	0.08408036	0.02	19.8%
Lama4	1.59186	0.28	17.5%
Lama5	0.2534352	0.05	18.2%
Lamb1-1	15.55136	3.32	21.3%
Lamb2	3.520346	0.50	14.3%
Lamb3	0.09381784	0.04	40.4%
Lamc1	14.84874	2.49	16.7%
Lamc2	0.5909492	0.24	40.1%
Lamc3	0.0171682	0.01	35.0%
Lamp1	109.3304	5.62	5.1%
Lamp2	43.1186	1.60	3.7%
LAMP2	0.1223319	0.04	31.6%
Lamp3	1.324628	0.11	8.3%
Lancl1	24.84732	0.18	0.7%
Lancl2	29.44184	2.22	7.5%
Lancl3	2.258742	0.47	20.9%
Lao1	0	0.00	
Lap1B	0.33832454	0.33	96.1%
Lap3	7.431442	0.44	5.9%
Laptm4a	72.95982	4.64	6.4%
Laptm4b	43.90196	2.38	5.4%
Laptm5	0.04069142	0.02	56.9%
Large	32.31168	1.43	4.4%
Larp1	42.61646	1.22	2.9%
Larp2	2.439582	0.28	11.6%
Larp4	10.95862	0.40	3.7%
Larp5	27.49594	1.00	3.6%
Larp6	7.933612	0.42	5.4%
Larp7	3.610104	0.38	10.6%
Lars	15.87734	1.10	7.0%
Lars2	84.23366	25.32	30.1%
Las1	0.039887136	0.06	140.8%
Las1l	13.37054	0.75	5.6%
Lasp1	21.24934	1.65	7.7%
Lass1	27.07692	2.07	7.6%
Lass2	11.1066	0.65	5.8%
Lass3	0.07832012	0.03	43.4%
Lass4	11.74912	0.69	5.9%
Lass5	23.43776	1.67	7.1%
Lass6	12.95546	1.24	9.6%
Lat	0.1659978	0.06	34.3%
Lat2	0.3162862	0.06	20.1%

Lats1	8.562268	0.43	5.1%
Lats2	8.185208	3.45	42.2%
Lax1	0.08810052	0.05	51.9%
Layn	0.216502	0.05	24.9%
Lba	0.4141812	0.13	32.5%
Lba1	13.4928	1.12	8.3%
Lbh	23.28052	1.53	6.6%
Lbp	0.57174	0.23	40.3%
Lbr	4.969962	0.36	7.3%
Lbx1	2.219156	0.30	13.6%
Lbx2	0.11607524	0.09	77.2%
Lbxcor1	4.331548	0.61	14.1%
Lca5	1.84322	0.38	20.8%
Lcat	0.1421626	0.03	20.4%
Lce1a1	0	0.00	
Lce1a2	0	0.00	
Lce1b	0	0.00	
Lce1c	0	0.00	
Lce1d	0	0.00	
Lce1e	0	0.00	
Lce1f	0	0.00	
Lce1g	0.00600792	0.01	223.6%
Lce1h	0	0.00	
Lce1i	0	0.00	
Lce1l	0	0.00	
Lce1m	0	0.00	
Lce3a	0.01169358	0.03	223.6%
Lce3b	0	0.00	
Lce3c	0	0.00	
Lce3f	0	0.00	
Lck	1.0438298	0.18	17.3%
Lcmt1	24.78638	1.72	6.9%
Lcmt2	1.523888	0.33	21.6%
Lcn10	0	0.00	
Lcn11	0	0.00	
Lcn12	0	0.00	
Lcn13	0	0.00	
Lcn2	0.23619218	0.13	54.8%
Lcn3	0	0.00	
Lcn4	0	0.00	
Lcn5	0	0.00	
Lcn8	0	0.00	
Lcn9	0	0.00	
Lcor	1.52177	0.10	6.3%
Lcorl	6.481018	0.63	9.7%
Lcp1	0.3812576	0.06	15.6%
Lcp2	0.08589762	0.03	39.3%

Lcrn	0.04003606	0.04	92.7%
Lct	0.002209264	0.00	138.6%
Lctl	0.01370074	0.01	60.7%
Ldb1	46.41576	2.22	4.8%
Ldb2	8.385442	0.83	9.8%
Ldb3	0.4180248	0.14	33.8%
Ldha	63.23656	10.68	16.9%
Ldhal6b	0.07331524	0.04	54.3%
Ldhb	97.37934	2.88	3.0%
Ldhc	0.00521268	0.01	223.6%
Ldhd	0.10317968	0.03	32.1%
LDLB	2.25138	0.88	39.1%
Ldlr	14.4517	1.00	6.9%
Ldlrad2	0	0.00	
Ldlrad3	5.167666	0.53	10.2%
Ldlrap1	0.5988472	0.14	23.3%
Ldoc1	0.04715628	0.04	75.2%
Ldoc1l	12.1638	1.15	9.4%
LE51	2.934116	0.35	11.8%
Leap2	0.01540686	0.03	223.6%
Lect1	0.01120516	0.02	154.8%
Lect2	0.00738582	0.02	223.6%
Lef1	0.1280475	0.05	40.7%
Lefty1	0	0.00	
Lefty2	0.0207933	0.01	68.3%
Lelp1	0	0.00	
Lemd1	1.047168	0.19	17.7%
Lemd2	12.10574	1.21	10.0%
Lemd3	7.898792	0.33	4.2%
Lenep	0.3556816	0.08	21.8%
Leng1	1.861496	0.20	10.7%
Leng4	30.11092	1.89	6.3%
Leng8	32.72432	3.23	9.9%
Leng9	1.914138	0.21	10.9%
Leo1	8.3722	0.30	3.6%
Lep	0.001755748	0.00	223.6%
Lepr	0.2464818	0.07	27.1%
Lepre1	3.688602	0.52	14.2%
Leprel1	0.9779692	0.19	19.4%
Leprel2	5.12446	0.33	6.4%
Leprot	9.185782	1.04	11.3%
Leprotl1	34.60106	1.22	3.5%
Letm1	18.60022	1.18	6.4%
Letm2	1.483116	0.24	16.3%
Letmd1	20.53998	1.09	5.3%
Lfg	0.7935838	0.71	89.2%
Lfng	3.512628	0.30	8.5%

Lgals1	6.998612	2.03	29.0%
Lgals12	0.01592032	0.02	137.6%
Lgals2	0.03139582	0.04	140.4%
Lgals3	0.09343418	0.07	75.1%
Lgals3bp	1.456744	0.29	20.1%
Lgals4	1.4800408	0.48	32.7%
Lgals7	0	0.00	
Lgals8	19.92822	0.21	1.1%
Lgals9	1.773572	0.24	13.3%
Lgi1	3.461782	0.25	7.1%
Lgi2	24.14976	1.88	7.8%
Lgi3	26.1822	2.93	11.2%
Lgi4	0.07845248	0.05	57.7%
Lgmn	47.15904	1.21	2.6%
Lgr4	9.64505	0.56	5.9%
Lgr5	1.499088	0.19	12.7%
Lgr6	0.09444652	0.02	16.1%
Lgtn	6.43811	0.36	5.5%
Lhb	0.0249091	0.03	138.9%
Lhcgr	0.05156878	0.02	33.8%
Lhfp	8.041406	1.07	13.3%
Lhfpl1	0.04040124	0.04	110.3%
Lhfpl2	5.846598	0.65	11.2%
Lhfpl3	24.59006	0.28	1.1%
Lhfpl4	30.5671	1.70	5.6%
Lhx1	4.998076	0.68	13.6%
Lhx2	7.096192	0.85	11.9%
Lhx3	0.03686444	0.02	57.4%
Lhx4	0.07885146	0.02	30.3%
Lhx5	0.6990254	0.12	17.1%
Lhx6	3.41173	0.15	4.4%
Lhx8	0.01492354	0.02	118.5%
Lhx9	1.514636	0.17	11.0%
Lias	10.120226	0.57	5.7%
Lif	0.07499026	0.02	26.6%
Lifr	5.319612	0.59	11.1%
Lig1	9.075718	0.25	2.7%
lig-2a	1.220195	1.71	140.5%
Lig3	2.28406	0.54	23.8%
Lig4	3.7249	0.44	11.9%
Lilrb3	0	0.00	
Lilrb4	0	0.00	
Lim2	0.0064405	0.01	223.6%
Lima1	1.956634	0.27	13.9%
Limch1	11.72436	0.55	4.7%
Limd1	2.265616	0.15	6.6%
limd1	0.3229048	0.08	23.3%

Limd2	4.66451	0.37	8.0%
Lime1	7.988914	1.25	15.6%
Limk1	58.43418	1.98	3.4%
Limk2	23.89772	0.62	2.6%
Lims1	9.456394	0.17	1.8%
Lims2	0.7384716	0.12	16.3%
Lin28	0.0101586	0.02	173.5%
Lin28b	2.420328	0.16	6.7%
Lin37	5.135352	0.33	6.3%
Lin52	4.504486	0.38	8.3%
Lin54	0.5361574	0.17	32.2%
Lin7a	23.7918	0.39	1.6%
Lin7b	28.00322	4.41	15.7%
Lin7c	36.15232	3.50	9.7%
Lin9	0.945328	0.24	25.2%
Lincr	0.13029294	0.07	50.9%
Lingo1	34.62286	3.97	11.5%
Lingo2	10.299512	0.90	8.7%
Lingo4	0.6326709	0.38	59.3%
Lins2	2.662016	0.49	18.6%
Lipa	7.014246	0.36	5.1%
Lipc	0.00306464	0.01	223.6%
Lipe	2.924184	0.19	6.5%
Lipf	0.0052045	0.01	223.6%
Lipg	0.04982926	0.04	76.7%
Liph	0.06729154	0.04	63.1%
Lipi	0.01455474	0.01	96.1%
Lipk	0	0.00	
Lipl3	0	0.00	
Lipn	0	0.00	
Lipt1	1.209454	0.12	10.1%
Litaf	2.20206	0.43	19.7%
Lix1	1.0252968	0.09	9.3%
Lix1l	8.004064	0.78	9.7%
lkb1	2.788508	0.18	6.6%
Llgl1	9.587724	1.04	10.8%
Llgl2	0.2361632	0.10	41.6%
Lman1	12.51018	0.53	4.2%
Lman1l	0	0.00	
Lman2	16.79302	0.60	3.6%
Lman2l	15.47566	0.69	4.4%
Lmbr1	14.56668	1.30	9.0%
Lmbr1l	4.417126	0.43	9.7%
Lmbrd1	21.912	1.21	5.5%
Lmbrd2	26.69004	2.35	8.8%
Lmcd1	0.355566	0.05	14.2%
Lmln	4.337574	0.45	10.3%

Lmna	14.96662	0.96	6.4%
Lmnb1	6.740734	0.54	8.0%
Lmnb2	5.881468	0.58	9.8%
Lmo1	4.850624	0.48	9.9%
Lmo2	2.520628	0.29	11.4%
Lmo3	9.486932	0.26	2.7%
Lmo4	24.81634	2.58	10.4%
Lmo6	0.10348028	0.03	24.6%
Lmo7	0.3729282	0.08	22.1%
Lmod1	0.017073748	0.02	99.8%
Lmod2	0	0.00	
Lmod3	0.01413364	0.02	172.2%
Lmtk2	16.76302	0.35	2.1%
Lmtk3	33.4225	1.31	3.9%
Lmx1a	0.13007498	0.05	40.6%
Lmx1b	2.510268	0.22	8.6%
Lnp	6.810902	0.92	13.5%
Lnpep	12.48588	1.71	13.7%
Lnv	0.03286026	0.01	39.3%
Lnx1	5.572926	0.37	6.6%
Lnx2	0.5964854	0.07	11.7%
LOC100034363	1.841248	0.47	25.3%
LOC100034729	0.00291274	0.01	223.6%
LOC100036568	0	0.00	
LOC100036766	0	0.00	
LOC100037259	0	0.00	
LOC100038977	0	0.00	
LOC100039065	0	0.00	
LOC100039319	0	0.00	
LOC100039672	0.10598264	0.03	29.1%
LOC100039834	0	0.00	
LOC100039890	0	0.00	
LOC100039913	0	0.00	
LOC100039945	1.25331	0.09	7.2%
LOC100039948	0	0.00	
LOC100039968	1.934788	0.35	18.0%
LOC100040208	0	0.00	
LOC100040223	0	0.00	
LOC100040233	0	0.00	
LOC100040340	17.52382	2.10	12.0%
LOC100040606	0	0.00	
LOC100040635	0	0.00	
LOC100041488	0	0.00	
LOC100041581	14.05682	1.91	13.6%
LOC100041724	0.05311306	0.05	97.5%
LOC100042109	0	0.00	
LOC100042144	0	0.00	

LOC100042175	0	0.00	
LOC100042180	2.80366	0.23	8.4%
LOC100042254	0	0.00	
LOC100042555	0.252358	0.06	25.8%
LOC100042782	0	0.00	
LOC100043125	0	0.00	
LOC100043216	0	0.00	
LOC100043315	0	0.00	
LOC100043381	0.001843056	0.00	223.6%
LOC100043387	0.13508112	0.07	49.3%
LOC100043860	0	0.00	
LOC100043868	0	0.00	
LOC100043899	0.00489452	0.01	223.6%
LOC171266	0	0.00	
LOC195555	0	0.00	
LOC217066	0.00654506	0.01	223.6%
LOC232875	7.233572	0.32	4.4%
LOC236118	0	0.00	
LOC272350	0.8760242	0.14	16.2%
LOC277666	0	0.00	
LOC277667	0	0.00	
LOC329575	2.453466	0.23	9.5%
LOC332923	0	0.00	
LOC380728	0.4941662	0.15	31.3%
LOC380994	0	0.00	
LOC381484	0.07217802	0.07	99.2%
LOC381535	0	0.00	
LOC381536	0	0.00	
LOC381806	0.00539806	0.01	223.6%
LOC382044	0.0032456	0.01	223.6%
LOC382133	0	0.00	
LOC383678	0.00998388	0.02	223.6%
LOC384639	0	0.00	
LOC384806	0	0.00	
LOC432649	1.1006448	0.14	12.6%
LOC433486	0	0.00	
LOC433722	0	0.00	
LOC433791	0.7503668	0.10	13.3%
LOC434864	0	0.00	
LOC434960	0	0.00	
LOC435023	0	0.00	
LOC435273	0	0.00	
LOC435286	0	0.00	
LOC435815	0	0.00	
LOC435818	0	0.00	
LOC436177	0.1471443	0.08	54.7%
LOC436479	0.02838668	0.02	58.1%

LOC544988	0	0.00	
LOC545291	0.0671485	0.02	36.9%
LOC545611	0	0.00	
LOC545637	0	0.00	
LOC545667	0.05985072	0.06	103.0%
LOC545732	0	0.00	
LOC546511	0	0.00	
LOC546519	0.0136825	0.02	117.0%
LOC546840	0	0.00	
LOC546849	0.07060544	0.09	124.0%
LOC547127	0.8408066	0.19	22.5%
LOC547323	0.0278464	0.06	223.6%
LOC548597	0	0.00	
LOC554292	0.027558192	0.01	46.3%
LOC574418	0.3569256	0.08	22.3%
LOC620779	0.00479744	0.01	223.6%
LOC620807	0.01313536	0.03	223.6%
LOC621852	0	0.00	
LOC622404	11.165842	1.35	12.1%
LOC622665	0.4048924	0.17	41.3%
LOC623272	0	0.00	
LOC625360	0	0.00	
LOC625480	0	0.00	
LOC625638	5.264094	0.28	5.3%
LOC626578	0.03917682	0.04	90.1%
LOC626832	5.526694	0.62	11.3%
LOC626922	0	0.00	
LOC626943	0	0.00	
LOC626995	0	0.00	
LOC627085	0	0.00	
LOC628053	0	0.00	
LOC628100	0	0.00	
LOC628308	1.439024	0.24	16.7%
LOC631002	0	0.00	
LOC631784	0	0.00	
LOC634428	0.001658364	0.00	223.6%
LOC654842	1.9582828	1.07	54.5%
LOC664987	0.01833188	0.03	137.3%
LOC665001	0.09346468	0.06	66.7%
LOC665596	0	0.00	
LOC665622	0	0.00	
LOC665992	0	0.00	
LOC665998	0	0.00	
LOC666244	3.79506	0.51	13.4%
LOC666927	0	0.00	
LOC667250	2.07099	0.26	12.4%
LOC667370	0.4327966	0.19	43.9%

LOC670464	0	0.00	
LOC670482	0	0.00	
LOC670496	0	0.00	
LOC692132	0	0.00	
LOC751864	0	0.00	
Loh11cr2a	6.13981	0.64	10.4%
Loh12cr1	9.106754	0.72	7.9%
Lonp1	25.90276	0.40	1.5%
Lonp2	22.71776	1.04	4.6%
Lonrf1	4.845578	0.34	7.0%
Lonrf2	64.34288	1.76	2.7%
Lonrf3	0.8820898	0.22	25.2%
Lor	3.448608	0.24	7.0%
Lox	6.576528	2.00	30.4%
Loxhd1	0.00949164	0.01	123.0%
Loxl1	0.7164516	0.14	19.3%
Loxl2	3.470534	1.28	36.8%
Loxl3	0.5265938	0.11	21.1%
Loxl4	0.4108782	0.14	34.5%
Lpgat1	23.68584	2.52	10.6%
Lphn1	65.3427	1.71	2.6%
Lphn2	24.9546	0.66	2.6%
Lphn3	37.94026	1.90	5.0%
Lpin1	11.76546	0.70	6.0%
Lpin2	13.71926	1.27	9.2%
Lpin3	0.09730876	0.03	26.0%
Lpl	9.693946	0.64	6.6%
Lpla2	1.3282856	0.63	47.4%
Lpo	0.1899872	0.06	32.2%
Lpp	0.415615	0.04	8.9%
lpr	0	0.00	
Lpxn	0.170826	0.05	30.4%
Lrat	0.05375604	0.02	40.4%
Lrba	2.758604	0.25	8.9%
Lrch1	8.41435	0.25	2.9%
Lrch2	6.975212	0.55	7.9%
Lrch3	4.708472	0.29	6.2%
Lrch4	1.936162	0.32	16.6%
Lrdd	0.9369652	0.14	14.7%
Lrfn1	25.39654	1.80	7.1%
Lrfn2	6.431082	0.81	12.6%
Lrfn3	9.333484	0.73	7.8%
Lrfn4	22.77592	2.59	11.4%
Lrfn5	29.62788	1.45	4.9%
Lrg1	0.03965662	0.05	134.2%
Lrig1	9.416564	0.45	4.8%
Lrig2	6.249734	0.52	8.3%

Lrig3	1.417084	0.23	16.5%
Lrit1	0.001523868	0.00	223.6%
Lrit2	0	0.00	
Lrmp	0.13410744	0.15	112.0%
Lrp1	16.15576	1.97	12.2%
Lrp10	6.78529	0.48	7.1%
Lrp11	27.20108	1.57	5.8%
Lrp12	22.96322	0.78	3.4%
Lrp1b	2.331834	0.40	17.3%
Lrp2	0.6774238	0.29	43.2%
Lrp2bp	0.462658	0.18	39.7%
Lrp3	24.71908	0.89	3.6%
Lrp4	2.291762	0.50	21.8%
Lrp5	1.372544	0.15	10.9%
Lrp6	3.573012	0.35	9.7%
Lrp8	11.49576	1.20	10.4%
Lrpap1	40.28398	1.08	2.7%
Lrpprc	22.10782	0.86	3.9%
Lrrc1	1.313624	0.10	7.5%
Lrrc10	0.0046896	0.01	223.6%
Lrrc14	13.42998	0.96	7.2%
Lrrc15	0.002727152	0.00	138.7%
Lrrc16	8.75805	0.35	4.0%
Lrrc17	2.76052	1.21	43.9%
Lrrc18	0.02147592	0.02	75.7%
Lrrc19	0.01935398	0.02	99.1%
Lrrc2	0.1731562	0.04	23.9%
Lrrc20	3.241568	0.24	7.3%
Lrrc23	0.08070794	0.04	49.7%
Lrrc24	17.37344	0.53	3.0%
Lrrc25	0	0.00	
Lrrc26	0.8321204	0.10	12.0%
Lrrc27	4.071234	0.13	3.1%
Lrrc28	9.74434	0.87	9.0%
Lrrc29	1.2580678	0.25	20.0%
Lrrc3	3.425038	0.63	18.3%
Lrrc30	0.00334202	0.01	223.6%
Lrrc33	0.7043692	0.04	6.1%
Lrrc34	0.026138474	0.02	70.6%
Lrrc36	0.039359	0.02	55.9%
Lrrc38	0.1451786	0.03	21.0%
Lrrc39	0.2770874	0.07	23.5%
Lrrc3b	11.38634	0.68	6.0%
Lrrc4	13.83252	1.12	8.1%
Lrrc40	9.472974	0.51	5.4%
Lrrc41	18.30828	1.17	6.4%
Lrrc42	6.021284	0.51	8.5%

Lrrc43	0.00618682	0.01	138.9%
Lrrc44	0.2390044	0.07	27.4%
Lrrc45	10.135116	1.19	11.8%
Lrrc46	0.4586546	0.14	30.6%
Lrrc47	9.5911	1.03	10.8%
Lrrc48	2.426238	0.26	10.8%
Lrrc49	25.41636	1.10	4.3%
Lrrc4b	54.31934	3.59	6.6%
Lrrc4c	15.62552	0.45	2.9%
Lrrc50	0.11200316	0.05	43.2%
Lrrc51	2.49198	0.48	19.1%
Lrrc52	0	0.00	
Lrrc55	0.7810218	0.10	12.9%
Lrrc56	0.9889812	0.15	14.9%
Lrrc57	5.200798	1.15	22.1%
Lrrc58	26.35276	1.09	4.1%
Lrrc59	42.73022	2.98	7.0%
Lrrc6	0.3366476	0.08	23.1%
Lrrc61	11.4291	0.25	2.1%
Lrrc62	13.38406	0.75	5.6%
Lrrc7	2.580576	0.19	7.3%
Lrrc8a	14.25624	0.33	2.3%
Lrrc8b	7.500298	0.74	9.9%
Lrrc8c	4.185956	0.13	3.2%
Lrrc8d	11.89526	0.93	7.8%
Lrrc8e	0.00486242	0.01	223.6%
Lrrc9	0.11427814	0.03	28.5%
Lrrcc1	11.99336	0.50	4.1%
Lrrfip1	11.53878	0.72	6.2%
Lrrfip2	6.16619	0.18	2.8%
Lrriq1	0.08942896	0.02	24.5%
Lrriq2	5.807224	1.73	29.7%
Lrrk1	0.6635932	0.03	4.2%
Lrrk2	1.352176	0.21	15.6%
Lrrn1	18.9954	1.01	5.3%
Lrrn2	18.88698	0.86	4.6%
Lrrn3	19.61462	0.88	4.5%
Lrrtm1	21.18952	1.69	8.0%
Lrrtm2	7.081866	0.37	5.2%
Lrrtm3	9.024514	0.72	8.0%
Lrrtm4	6.681268	0.24	3.6%
Lrsam1	16.00494	0.26	1.7%
Lrtm1	0.00417874	0.01	223.6%
Lrtm2	8.738338	0.67	7.7%
Lsamp	49.19702	3.61	7.3%
Lsg1	4.613678	0.27	5.9%
Lsm1	7.520036	0.67	8.9%

Lsm10	8.187606	0.69	8.4%
Lsm11	22.44964	1.30	5.8%
Lsm12	12.38134	1.06	8.6%
Lsm14a	17.12308	0.81	4.7%
Lsm14b	29.3225	1.96	6.7%
Lsm2	3.606368	0.80	22.3%
Lsm3	12.35748	1.34	10.9%
Lsm4	21.28028	2.53	11.9%
Lsm5	0.9651752	0.32	33.5%
Lsm6	14.69326	0.77	5.3%
Lsm7	0.9467332	0.13	13.7%
Lsm8	9.318832	0.70	7.5%
Lsmd1	14.6905	0.74	5.0%
Lsp1	0.4530798	0.10	21.1%
Lsr	0.7947944	0.30	37.3%
Lss	20.3032	1.27	6.3%
Lst1	0.1860184	0.10	51.5%
Lta	0.02665932	0.02	73.3%
Lta4h	18.78534	0.86	4.6%
Ltb	0.08993778	0.09	99.9%
Ltb4dh	0.6986262	0.08	11.3%
Ltb4r1	0.04302974	0.02	39.7%
Ltb4r2	0.0298249	0.03	93.6%
Ltbp1	1.1075844	0.12	11.1%
Ltbp2	0.07344466	0.02	27.2%
Ltbp3	7.698036	0.72	9.3%
Ltbp-3	1.63035	0.47	29.1%
Ltbp4	3.139358	0.64	20.4%
Ltbr	1.0170352	0.18	17.7%
Ltc4s	0.05972374	0.10	169.4%
Ltf	0.00572286	0.01	144.2%
Ltk	3.165042	0.30	9.6%
Ltv1	4.334774	0.21	4.7%
Luc7l	28.75312	2.01	7.0%
Luc7l2	27.29016	1.48	5.4%
Lum	10.207628	2.24	22.0%
Luzp1	18.22184	2.05	11.2%
Luzp2	4.974998	0.16	3.2%
Lvrn	0.017452	0.02	105.2%
Lxn	5.491086	0.72	13.2%
Ly49A	0	0.00	
Ly49G	0	0.00	
Ly-49Q	0	0.00	
Ly-49W	0	0.00	
Ly6a	0.28786722	0.17	57.8%
Ly6c1	0.0756309	0.08	107.7%
Ly6c2	0.00849108	0.02	223.6%

Ly6d	0.0652604	0.04	56.8%
Ly6e	30.48124	2.55	8.4%
Ly6f	0	0.00	
Ly6g5b	0.134395	0.07	53.8%
Ly6g5c	0	0.00	
Ly6g6c	0.00651968	0.01	223.6%
Ly6g6d	0	0.00	
Ly6g6e	0	0.00	
Ly6h	176.9146	19.70	11.1%
Ly6i	0	0.00	
Ly6k	0.00631882	0.01	223.6%
Ly75	0.07856144	0.04	44.7%
Ly86	0	0.00	
Ly9	0.005572	0.01	138.6%
Ly96	0.541948	0.18	33.8%
Lyar	4.06044	0.25	6.2%
Lycat	9.445586	0.90	9.5%
Lyg1	0.00821836	0.02	223.6%
Lyg2	0.0058858	0.01	223.6%
Lyl1	0.04989134	0.03	50.4%
Lyn	1.451586	0.21	14.7%
Lynx1	107.56218	8.64	8.0%
Lypd1	48.40502	1.78	3.7%
Lypd2	0	0.00	
Lypd3	0.0056595	0.01	223.6%
Lypd4	0	0.00	
Lypd5	0	0.00	
Lypd6	13.502	0.62	4.6%
Lypla1	15.01722	1.13	7.5%
Lypla2	27.32458	2.13	7.8%
Lypla3	6.180504	0.51	8.2%
Lyplal1	8.200794	0.69	8.4%
Lyrm1	3.21818	0.56	17.3%
Lyrm2	11.79776	0.32	2.7%
Lyrm4	11.67512	0.93	8.0%
Lyrm5	9.670892	0.65	6.8%
Lyrm7	1.0146548	0.24	24.1%
Lysmd1	0.6306104	0.07	11.8%
Lysmd2	12.12586	0.85	7.0%
Lysmd3	3.260316	0.20	6.2%
Lysmd4	12.23198	0.76	6.2%
Lyst	2.558964	0.35	13.6%
Lyst2	24.7233	1.91	7.7%
Lyve1	0.0324254	0.03	81.0%
Lyz	0.006935052	0.01	170.7%
Lyzl1	0	0.00	
Lyzl4	0.01318934	0.02	138.2%

Lyzl6	0	0.00	
Lyzs	0.1337126	0.05	35.2%
Lzic	6.374712	0.45	7.0%
Lztfl1	5.477942	0.23	4.2%
Lztr1	26.31242	1.59	6.0%
Lzts1	1.139314	0.11	9.5%
Lzts2	7.179482	0.38	5.3%
M11858	0	0.00	
M11859	0	0.00	
M11860	0	0.00	
M11896	0.0335766	0.08	223.6%
M12570	0	0.00	
M13188	0	0.00	
M13677	0	0.00	
M13680	0	0.00	
M14294	0	0.00	
M15063	0	0.00	
M16678	0	0.00	
M16679	0	0.00	
M19910	0	0.00	
M20876	0	0.00	
M25937	93.5917	15.29	16.3%
M26425	0	0.00	
M26448	0	0.00	
M34214	0	0.00	
M34473	0.000420906	0.00	223.6%
M35492	0	0.00	
M36767	0	0.00	
M37285	0	0.00	
M37286	0	0.00	
M37296	0	0.00	
M37592	0	0.00	
M38681	0	0.00	
M55219	0	0.00	
M6B	0.3531762	0.10	29.2%
M6pr	20.2386	0.25	1.3%
M6prbp1	1.1804118	0.18	15.3%
M71214	0	0.00	
M75	0.2970416	0.13	44.1%
M76612	0	0.00	
M83997	1.1631854	0.45	38.7%
Mab21l1	5.230076	0.12	2.4%
Mab21l2	31.4445	4.73	15.0%
Macf	17.02332	1.16	6.8%
Macf1	20.02732	1.09	5.5%
Macrod1	6.839994	0.87	12.7%
Macrod2	7.73886	0.56	7.2%

Mad1	0.05658898	0.01	18.3%
Mad1l1	5.594208	0.69	12.4%
Mad2l1	2.752242	0.54	19.5%
Mad2l1bp	3.555694	0.38	10.6%
Mad2l2	8.3707	0.80	9.6%
mADAM33-alpha	0.004510935	0.01	215.7%
Madcam1	0.04956104	0.02	45.7%
Madd	14.475956	4.26	29.4%
MADP-1	2.156298	0.55	25.7%
Maea	56.07912	2.95	5.3%
Mael	0.3011072	0.07	22.0%
Maf	7.886878	0.59	7.4%
Maf1	22.83432	0.48	2.1%
mAF10	0.176261276	0.11	64.9%
Mafa	0.298323	0.09	31.0%
Mafb	1.292157	0.32	24.7%
Maff	1.1197374	0.17	15.4%
Mafg	16.3871	1.04	6.4%
Mafk	1.934054	0.15	7.7%
Mag	0.362373	0.31	85.2%
Magea1	0	0.00	
Magea10	0	0.00	
Magea2	0	0.00	
Magea3	0	0.00	
Magea4	0	0.00	
Magea5	0	0.00	
Magea6	0	0.00	
Magea7	0	0.00	
Magea8	0	0.00	
Magea9	0	0.00	
Mageb18	0	0.00	
Mageb2	0	0.00	
Mageb3	0.02772942	0.03	114.4%
Mageb5	0	0.00	
Maged1	281.5504	5.47	1.9%
Maged2	109.0204	3.85	3.5%
Magee1	90.23594	3.53	3.9%
Magee2	2.786384	0.48	17.2%
Mageh1	17.31976	2.15	12.4%
Magi1	25.99078	1.60	6.2%
Magi2	8.17152	0.33	4.1%
Magi3	13.59844	0.81	5.9%
Magix	0	0.00	
Magmas	0.2473752	0.12	47.5%
Magoh	18.3332	1.08	5.9%
MAIR-IIa	0	0.00	
Mak	0.08732258	0.04	49.9%

Mak10	17.22406	0.84	4.9%
Mal	0.15570442	0.08	54.1%
Mal2	19.0393	0.86	4.5%
Mall	0.00651406	0.01	137.7%
Malt1	0.825296	0.13	15.2%
Mamdc2	0.594206	0.08	14.3%
Mamdc4	0.2319832	0.02	10.6%
Maml1	1.26933	0.15	12.0%
Maml2	1.277644	0.09	7.3%
Maml3	1.491822	0.19	12.4%
Man1a	3.648494	0.43	11.9%
Man1a2	14.59144	0.69	4.7%
Man1b1	16.9316	0.33	2.0%
Man1c1	4.1725	0.22	5.4%
Man2a1	5.565382	0.32	5.8%
Man2a2	25.31636	1.89	7.5%
Man2b1	6.066762	0.29	4.8%
Man2b2	4.76381	0.34	7.1%
MAN2B2	1.109071	0.73	66.2%
Man2c1	14.32714	1.27	8.8%
Manba	2.404222	0.23	9.5%
Manbal	22.8929	1.91	8.3%
Manea	8.463402	0.51	6.0%
Mansc1	0.3856092	0.11	28.2%
Maoa	9.295094	0.63	6.8%
Maob	0.13088344	0.04	27.5%
Map1lc3a	100.55212	9.99	9.9%
Map1lc3b	82.15334	2.83	3.4%
Map2k1	40.89266	2.28	5.6%
Map2k1ip1	6.731546	0.61	9.1%
Map2k2	21.2034	1.78	8.4%
Map2k3	3.66673	0.36	9.7%
Map2k4	64.31038	1.26	2.0%
Map2k5	11.6763	0.34	2.9%
Map2k6	2.930226	0.34	11.5%
Map2k7	15.754	0.43	2.7%
Map3k1	2.244076	0.08	3.7%
Map3k10	31.84296	2.52	7.9%
Map3k11	3.653136	0.10	2.8%
Map3k12	31.51616	0.85	2.7%
Map3k13	1.966378	0.25	12.8%
Map3k14	0.3307988	0.06	19.0%
Map3k2	2.755776	0.51	18.5%
Map3k3	3.113542	0.31	9.9%
Map3k4	6.888326	0.14	2.1%
Map3k5	1.710126	0.13	7.7%
Map3k6	0.04561162	0.01	27.3%

Map3k7	6.7112	0.56	8.4%
Map3k7ip1	9.311498	0.66	7.1%
Map3k7ip2	14.04352	0.58	4.1%
Map3k7ip3	5.73865	0.40	6.9%
Map3k8	0.13784556	0.05	33.4%
Map3k9	9.87734	1.08	10.9%
Map4k1	1.1874424	0.12	9.7%
Map4k2	21.6864	2.67	12.3%
Map4k3	16.81876	0.25	1.5%
Map4k4	23.78588	0.30	1.3%
Map4k5	5.221488	0.31	6.0%
Map6d1	5.774162	0.25	4.3%
Mapbpip	31.97862	2.01	6.3%
Mapk1	69.81214	1.91	2.7%
Mapk10	42.52534	0.93	2.2%
Mapk11	5.455718	0.60	10.9%
Mapk12	2.564376	0.39	15.3%
Mapk13	0.05224362	0.03	66.0%
Mapk14	10.199192	0.71	7.0%
Mapk15	0.3320098	0.09	26.5%
Mapk1ip1	12.65946	0.60	4.7%
Mapk3	45.90352	1.53	3.3%
Mapk4	3.237734	0.21	6.4%
Mapk6	31.57382	3.37	10.7%
Mapk7	5.017542	0.61	12.1%
Mapk8	28.74144	2.00	6.9%
Mapk8ip1	121.904	9.39	7.7%
Mapk8ip2	89.49546	3.52	3.9%
Mapk8ip3	64.90246	2.45	3.8%
Mapk9	103.5036	1.98	1.9%
Mapkap1	13.79116	0.63	4.6%
Mapkapk2	7.227762	0.45	6.2%
Mapkapk3	0.5460502	0.08	14.5%
Mapkapk5	7.122566	0.97	13.5%
Mapkbp1	5.59647	0.26	4.6%
Mapre1	29.6681	2.18	7.3%
Mapre2	102.6992	5.93	5.8%
Mapre3	53.28232	2.16	4.0%
Mapt	124.2266	9.62	7.7%
Marcks	63.90784	4.36	6.8%
Marcksl1	88.36064	9.31	10.5%
Marco	0	0.00	
Mare	3.97997	0.41	10.3%
Mark1	21.86354	0.80	3.7%
Mark2	17.16286	0.51	3.0%
Mark3	25.30676	1.54	6.1%
Mark4	16.36194	1.41	8.6%

Mars	19.3073	2.22	11.5%
Mars2	2.360848	0.12	5.1%
Marveld1	2.520202	0.31	12.3%
Marveld2	0.13408258	0.05	36.7%
Marveld3	0.0530963	0.03	58.1%
Mas1	0.01514546	0.02	107.0%
Masp1	3.720012	0.30	8.1%
Masp2	2.617178	0.46	17.6%
masp3	0.07029992	0.07	95.5%
Mass1	0	0.00	
Mast1	40.0084	2.18	5.4%
Mast2	15.1401	0.57	3.7%
Mast3	0.7977922	0.11	13.8%
Mast4	9.227666	0.40	4.4%
Mastl	0.837449	0.10	11.6%
Mat1a	0.00273196	0.01	223.6%
Mat2a	48.11282	2.11	4.4%
Mat2b	15.20302	1.02	6.7%
mat-4	0.034834348	0.02	56.7%
Matk	23.51022	2.79	11.9%
Matn1	0	0.00	
Matn2	2.920548	0.31	10.4%
Matn3	0.10385532	0.07	65.5%
Matn4	0.19552058	0.09	45.8%
Matr3	114.9552	6.73	5.9%
mAWMS3	0.03383818	0.00	10.5%
Max	20.13388	1.76	8.8%
Maz	52.5799	1.53	2.9%
mazr	1.1226866	0.20	17.5%
Mb	0.00989768	0.02	223.6%
mBB1	21.10168	0.89	4.2%
Mbc2	7.938478	0.56	7.1%
Mbd1	11.18114	0.42	3.8%
Mbd2	8.581518	0.42	4.9%
Mbd3	27.25652	2.36	8.6%
Mbd3l1	0	0.00	
Mbd3l2	0.01570304	0.01	58.3%
Mbd4	0.9263692	0.09	9.3%
Mbd5	7.682834	0.68	8.8%
Mbd6	7.402126	0.60	8.1%
Mbip	3.958858	0.42	10.6%
Mbl1	0.00503312	0.01	223.6%
Mbl2	0.04361502	0.03	67.2%
Mbnl1	21.17056	1.15	5.4%
Mbnl2	30.43052	5.08	16.7%
Mbnl3	0.2068924	0.03	14.2%
Mboat1	0.2979224	0.05	17.7%

Mboat2	6.63287	0.26	4.0%
Mboat5	8.35077	0.99	11.9%
mboct	191.526	5.07	2.6%
Mbp	16.2629	2.00	12.3%
Mbtd1	2.600062	0.22	8.3%
Mbtps1	17.8786	1.02	5.7%
Mbtps2	8.950038	0.67	7.5%
Mc1r	0.02446734	0.01	51.8%
Mc2r	0.0640536	0.06	99.5%
Mc3r	0	0.00	
Mc4r	2.46976	0.34	13.8%
Mc5r	0.19893716	0.14	69.2%
Mcam	0.4727826	0.10	22.1%
Mcart1	24.2002	1.08	4.5%
Mcart6	4.952428	0.46	9.4%
Mcat	9.437992	0.35	3.7%
Mcc	1.983252	0.09	4.5%
Mccc1	2.434614	0.92	37.6%
Mccc2	7.451716	0.17	2.2%
Mcee	5.4113	0.20	3.6%
Mcf2	2.878458	0.15	5.4%
Mcf2l	27.73562	0.50	1.8%
Mcfd2	11.83494	0.82	6.9%
Mchr1	1.69337	0.43	25.6%
Mcl	0	0.00	
Mcl1	16.26784	0.62	3.8%
mCLCA1	0.0342606	0.02	50.1%
Mcm10	0.5540886	0.11	20.6%
Mcm2	1.953068	0.23	11.7%
Mcm3	1.3088306	0.21	16.3%
Mcm3ap	6.729838	0.15	2.2%
Mcm4	2.924986	0.31	10.5%
Mcm5	1.86096	0.22	11.7%
Mcm6	3.373374	0.37	11.1%
Mcm7	1.359214	0.16	12.0%
Mcm8	1.18737	0.06	4.8%
Mcm9	0.8808446	0.09	10.4%
Mcoln1	23.28148	0.83	3.5%
Mcoln2	0	0.00	
Mcoln3	0.00387158	0.01	223.6%
Mcph1	3.154102	0.23	7.3%
Mcpt1	0	0.00	
Mcpt2	0.00646526	0.01	223.6%
Mcpt4	0	0.00	
Mcpt8	0	0.00	
Mcpt9	0	0.00	
Mcrs1	3.818014	0.52	13.5%

Mctp1	10.048842	0.35	3.4%
Mctp2	0.0508647	0.02	42.9%
Mcts1	35.7854	1.45	4.1%
Mcts2	9.239202	0.82	8.9%
mD53	0.3397424	0.11	31.4%
Mdc1	3.63427	0.29	8.0%
Mdcp1	3.759886	0.37	9.8%
mdcr	1.3337548	0.45	34.0%
mDEAH9	2.935196	0.27	9.4%
Mdfi	0.7989262	0.16	19.6%
Mdfic	1.2369836	0.22	18.0%
Mdga1	24.54772	2.11	8.6%
Mdga2	8.026472	0.60	7.5%
Mdh1	325.677	2.61	0.8%
Mdh1b	0.5191766	0.11	20.8%
Mdh2	188.9218	9.90	5.2%
mDj11	14.71038	1.50	10.2%
Mdk	9.019962	1.41	15.6%
Mdl1	0.001898848	0.00	95.0%
mDLG6A	0.007873918	0.01	103.7%
Mdm1	1.374426	0.14	10.1%
Mdm2	10.7994	0.53	4.9%
Mdm4	4.568386	0.71	15.6%
Mdmx	4.492186	0.93	20.6%
Mdn1	2.374698	0.44	18.4%
Mds1	0.27501912	0.17	60.4%
Me2	5.691242	0.39	6.9%
Me3	2.05077	0.16	7.9%
Mea1	49.7679	5.88	11.8%
Mecp2	13.9059	0.71	5.1%
Mecr	10.990282	1.02	9.3%
Med1	12.7604	0.56	4.4%
Med10	14.60988	1.79	12.3%
Med11	4.016892	0.48	12.0%
Med12	4.055032	0.20	4.9%
Med12l	4.24662	0.52	12.3%
Med13	6.452848	0.23	3.6%
Med13l	5.305994	0.27	5.1%
Med14	18.54102	0.91	4.9%
Med15	9.85398	0.32	3.2%
Med16	10.67286	0.22	2.0%
Med17	4.614284	0.17	3.6%
Med18	1.667466	0.26	15.8%
Med19	18.41516	1.77	9.6%
Med20	10.872568	0.98	9.0%
Med21	17.62276	0.90	5.1%
Med22	14.43208	0.48	3.3%

Med23	6.43425	0.06	1.0%
Med24	13.0795	0.68	5.2%
Med25	15.79058	0.95	6.0%
Med26	2.669274	0.22	8.1%
Med27	11.8068	1.47	12.5%
Med28	7.631724	0.29	3.8%
Med29	2.341592	0.27	11.4%
Med30	11.24072	0.79	7.0%
Med31	5.432244	0.40	7.3%
Med4	7.120932	0.60	8.4%
Med6	6.813938	0.35	5.2%
Med7	6.876134	0.23	3.4%
Med8	6.712276	0.70	10.4%
Med9	14.75328	0.47	3.2%
Mef2a	9.456852	1.00	10.6%
Mef2b	2.595238	0.21	8.2%
Mef2c	3.04279	0.17	5.6%
Mef2d	16.73116	1.63	9.7%
Mefv	0.00150874	0.00	223.6%
Megf10	1.1828492	0.14	11.5%
Megf11	6.627546	0.42	6.3%
Megf12	0.07609286	0.02	23.1%
Megf6	0.9103458	0.13	14.3%
Megf8	14.52454	1.73	11.9%
Megf9	17.9131	1.47	8.2%
Mei1	0.01150166	0.02	141.2%
Meig1	0.1899938	0.16	84.0%
Meis1	18.15856	1.60	8.8%
Meis2	19.59664	2.45	12.5%
meis2a	4.644424	1.81	38.9%
mel1	0.004151551	0.00	92.0%
Mel13	9.670586	0.46	4.8%
Mela	0	0.00	
Melk	0.4055338	0.12	28.8%
Memo1	11.44184	0.70	6.1%
Men1	24.21426	0.93	3.8%
Meox1	0.00454748	0.01	137.0%
Meox2	0.8556204	0.12	13.8%
Mep1a	0.02267464	0.02	70.9%
Mep1b	0.00361196	0.01	223.6%
Mep-1beta	0.01256626	0.03	223.6%
mEP2e	0	0.00	
Mepce	9.16657	0.42	4.6%
Mepe	0	0.00	
Mertk	0.587178	0.10	17.6%
Mesdc1	6.34282	0.21	3.2%
Mesdc2	12.16244	0.53	4.4%

Mesp1	0.01949988	0.01	59.9%
Mesp2	0.11547254	0.04	35.7%
Mest	72.54996	10.59	14.6%
Met	0.4400422	0.11	25.8%
Metap1	15.6286	0.33	2.1%
Metap2	19.32664	1.21	6.2%
Metapl1	3.65162	0.30	8.2%
Metrn	8.361522	0.86	10.3%
Metrnl	2.22794	0.18	8.0%
Mett10d	10.74274	0.31	2.9%
Mett11d1	9.75725	0.29	2.9%
Mett5d1	1.590546	0.14	8.7%
Mettl1	5.031324	0.39	7.7%
Mettl2	6.387928	0.30	4.7%
Mettl3	6.517668	0.59	9.0%
Mettl4	2.467278	0.22	8.8%
Mettl5	5.588448	0.52	9.2%
Mettl6	7.051802	0.31	4.3%
Mettl7a	0.762191	0.10	12.6%
Mettl7b	0.12765074	0.06	48.0%
Mettl8	4.095384	0.25	6.1%
Mettl9	24.71334	1.38	5.6%
Mex3a	2.27873	0.10	4.3%
Mex3c	12.06674	0.52	4.3%
Mex3d	5.869888	0.57	9.7%
Mfap1a	3.90909	0.23	6.0%
Mfap1b	3.96474	0.20	5.1%
Mfap2	8.110646	1.01	12.5%
Mfap3	6.227424	0.44	7.1%
Mfap3l	5.714768	0.42	7.4%
Mfap4	3.96031	0.66	16.6%
Mfap5	0.233234	0.13	54.6%
Mfge8	7.714218	1.07	13.8%
Mfhas1	6.845516	0.11	1.6%
Mfi2	0.00356194	0.00	137.0%
mFKHLA	0.04679104	0.01	18.5%
mFLJ00017	0.00639975	0.01	79.0%
mFLJ00022	0.1933164	0.04	19.5%
mFLJ00025	0.06766486	0.08	111.9%
mFLJ00067	0.015710862	0.01	42.8%
mFLJ00088	0.7828168	0.21	27.4%
mFLJ00128	0.11593098	0.06	55.5%
mFLJ00150	0.3858972	0.14	36.6%
mFLJ00177	0.10452252	0.03	30.5%
mFLJ00205	0.2153254	0.06	27.0%
mFLJ00209	0.22141274	0.13	60.3%
mFLJ00251	0.7681492	0.21	27.3%

mFLJ00267	0.011815716	0.01	55.5%
mFLJ00277	1.52659	0.48	31.8%
mFLJ00298	0.5227906	0.11	21.1%
mFLJ00332	0.14667912	0.08	53.9%
mFLJ00369	1.425488	0.96	67.1%
mFLJ00377	0.4584588	0.24	52.5%
Mfn1	15.75216	0.93	5.9%
Mfn2	49.30248	0.44	0.9%
Mfng	0.06826512	0.03	37.4%
Mfrp	0.011114032	0.01	67.0%
Mfsd1	8.939046	0.28	3.1%
Mfsd11	9.804574	1.33	13.6%
Mfsd2	1.209088	0.09	7.7%
Mfsd3	6.119804	0.51	8.3%
Mfsd4	3.760934	0.44	11.8%
Mfsd7	0.02260478	0.02	81.6%
Mfsd8	2.230242	0.20	8.8%
Mfsd9	2.905574	0.18	6.1%
Mga	5.772076	0.91	15.7%
Mgam	0	0.00	
Mgat1	12.2473	0.61	5.0%
Mgat3	55.02124	1.78	3.2%
Mgat4a	3.91136	0.18	4.6%
Mgat4b	22.62168	1.94	8.6%
Mgat4c	2.88067	0.29	10.2%
Mgat5	16.43746	1.37	8.3%
Mgat5b	16.05052	1.44	9.0%
MGC107098	0	0.00	
MGC107415	4.783872	0.54	11.2%
MGC58426	0	0.00	
MGC67181	2.464272	0.17	6.8%
MGC68323	0.3829254	0.11	28.3%
Mgea5	67.4228	3.47	5.2%
Mgl1	0	0.00	
Mgl2	0	0.00	
Mgll	6.892346	0.50	7.3%
Mgmt	0.7506252	0.18	23.5%
Mgp	5.75429	1.34	23.3%
mGpi1	3.099348	0.39	12.5%
Mgrn1	60.35638	2.35	3.9%
Mgst1	9.750626	0.20	2.0%
Mgst2	0.6136666	0.37	59.7%
Mgst3	74.69664	6.51	8.7%
mHTATIP	1.87836	0.39	21.0%
Mia1	0.08018266	0.07	89.6%
Mia2	0.0355599	0.03	89.2%
Mia3	0	0.00	

Mib1	22.2462	1.30	5.9%
Mib2	20.07192	0.65	3.2%
Mical1	0.8801576	0.14	15.5%
Mical2	4.965948	0.39	7.9%
Mical3	9.430058	0.52	5.5%
Micalcl	0.0500522	0.01	26.5%
Micall1	5.646674	0.38	6.8%
Micall2	0.2287996	0.05	22.9%
Mid1	7.322802	0.87	11.9%
Mid1ip1	15.86972	1.82	11.5%
Mid2	9.821694	0.12	1.2%
Midn	18.6144	1.60	8.6%
Mier1	5.573714	0.46	8.3%
Mier2	8.175788	0.30	3.6%
Mier3	4.792342	0.21	4.4%
Mif	27.34846	1.30	4.8%
Mif1	2.945526	0.30	10.1%
Mif4gd	4.868478	1.38	28.4%
Mill1	0	0.00	
Mill2	0.0916161	0.05	56.1%
mimp3	0.1206896	0.06	48.8%
Mina	1.324484	0.24	18.4%
MINK	0.8744544	0.35	39.9%
Mink1	14.79256	0.75	5.0%
Minpp1	18.2651	1.44	7.9%
MINT	0.08578674	0.04	46.4%
Miox	0	0.00	
Mip	0.0288998	0.02	59.8%
Mipep	4.418398	0.13	3.0%
Mipol1	0.7275786	0.55	75.3%
Mir16	69.64668	0.85	1.2%
Mis12	7.687406	0.52	6.8%
Mitd1	2.67811	0.14	5.3%
Mitf	1.263864	0.17	13.4%
mitsugumin29	0.00491782	0.01	223.6%
miwi	0.00083331	0.00	223.6%
Mixl1	0.01434578	0.01	59.4%
Mizf	4.513466	0.33	7.4%
Mki67	0.9664824	0.19	19.9%
Mki67ip	15.9486	1.48	9.3%
mKIAA0003	0.04591924	0.01	25.6%
mKIAA0004	15.331104	7.45	48.6%
mKIAA0007	0.5002324	0.24	47.6%
mKIAA0008	0.05844032	0.01	15.4%
mKIAA0010	0.02946322	0.01	47.1%
mKIAA0031	3.573428	0.88	24.7%
mKIAA0044	4.273974	0.32	7.4%

mKIAA0050	0	0.00	
mKIAA0054	0.922686	0.10	11.3%
mKIAA0056	2.77564	0.46	16.7%
mKIAA0074	0.05570978	0.03	54.3%
mKIAA0076	1.449998	0.39	26.7%
mKIAA0079	4.0607	0.45	11.2%
mKIAA0083	0.110291	0.05	42.4%
mKIAA0084	0.07190858	0.02	22.4%
mKIAA0088	0.8166892	0.21	26.0%
mKIAA0097	18.1675	2.85	15.7%
mKIAA0134	0.15791392	0.09	59.2%
mKIAA0135	0.6466568	0.04	6.7%
mKIAA0136	0.519547	0.21	39.6%
mKIAA0137	1.666146	0.24	14.4%
mKIAA0139	0.1560842	0.07	46.9%
mKIAA0145	0.782617	0.37	47.3%
mKIAA0148	0.1432884	0.05	33.8%
mKIAA0154	0.8893198	0.45	50.6%
mKIAA0157	0.7555044	0.78	102.7%
mKIAA0164	9.376248	0.50	5.3%
mKIAA0168	0.02741856	0.02	72.3%
mKIAA0169	0.8181306	0.54	65.9%
mKIAA0173	0.3147756	0.15	47.0%
mKIAA0174	3.266028	0.67	20.4%
mKIAA0184	4.71206	0.44	9.2%
mKIAA0189	0.09179496	0.03	32.1%
mKIAA0191	0.11154936	0.04	38.6%
mKIAA0195	0.6966786	0.48	69.1%
mKIAA0200	0.3847956	0.08	20.4%
mKIAA0210	0.19190128	0.10	52.1%
mKIAA0215	0.6604956	0.18	26.6%
mKIAA0225	3.182366	0.57	17.9%
mKIAA0226	2.056468	0.64	31.3%
mKIAA0230	0.744072	0.51	68.1%
mKIAA0236	0.612531087	0.40	64.7%
mKIAA0237	0.639909	0.17	26.4%
mKIAA0241	0.1895214	0.06	29.2%
mKIAA0244	0.3117126	0.10	31.6%
mKIAA0245	1.0232346	0.18	17.3%
mKIAA0248	0.22017738	0.09	41.2%
mKIAA0255	2.325786	0.38	16.5%
mKIAA0259	0.0639134	0.02	27.8%
mKIAA0260	0.0374506	0.02	45.0%
mKIAA0267	3.370484	0.90	26.7%
mKIAA0281	1.33749	0.33	24.8%
mKIAA0294	0.18689002	0.10	51.8%
mKIAA0297	0.06950744	0.01	21.3%

mKIAA0306	0.3877394	0.21	54.3%
mKIAA0308	5.037516	0.48	9.5%
mKIAA0312	0.500435984	0.53	105.8%
mKIAA0318	5.1199	0.39	7.6%
mKIAA0321	0.0908332	0.04	41.4%
mKIAA0326	2.366914	0.27	11.3%
mKIAA0327	11.12244	1.33	12.0%
mKIAA0338	3.410196	1.61	47.2%
mKIAA0340	0.7305708	0.28	38.9%
mKIAA0344	0.3267782	0.06	19.1%
mKIAA0348	0.4002212	0.10	24.0%
mKIAA0350	1.3872652	0.65	46.9%
mKIAA0351	0.05314608	0.01	23.4%
mKIAA0358	17.56012	3.04	17.3%
mKIAA0367	0.223086	0.07	31.5%
mKIAA0368	1.1225944	0.29	26.0%
mKIAA0369	7.824888	3.48	44.5%
mKIAA0370	3.31555	0.87	26.4%
mKIAA0371	1.75834	0.25	14.4%
mKIAA0373	1.372466	0.16	11.9%
mKIAA0375	9.287316	0.77	8.3%
mKIAA0376	3.600242	0.11	3.0%
mKIAA0377	0.532781	0.26	48.7%
mKIAA0378	0.0398835	0.03	81.2%
mKIAA0387	0.007362318	0.00	28.6%
mKIAA0390	0.10047728	0.08	76.9%
mKIAA0397	0.7144042	0.08	11.8%
mKIAA0399	0.7900446	0.43	53.8%
mKIAA0404	0.543718	0.06	10.2%
mKIAA0419	1.258012	0.15	12.2%
mKIAA0429	0.021383016	0.01	41.2%
mKIAA0432	0.07002466	0.04	52.6%
mKIAA0433	8.339732	1.33	15.9%
mKIAA0439	0.2806154	0.07	24.5%
mKIAA0444	7.393364	0.50	6.7%
mKIAA0452	0.16174752	0.09	53.1%
mKIAA0453	0.09806204	0.02	20.2%
mKIAA0460	0.24752818	0.31	127.0%
mKIAA0470	0.14930826	0.05	31.7%
mKIAA0471	0.1208641	0.05	39.2%
mKIAA0474	1.0091642	0.80	79.1%
mKIAA0480	2.543738	0.31	12.3%
mKIAA0512	0.07457832	0.02	24.2%
mKIAA0515	33.84922	2.49	7.3%
mKIAA0528	1.3251824	0.29	22.2%
mKIAA0532	0.05865132	0.01	25.2%
mKIAA0538	0.2740106	0.11	40.1%

mKIAA0544	7.643294	1.00	13.1%
mKIAA0545	1.3904	0.22	15.7%
mKIAA0560	0.4570644	0.05	11.6%
mKIAA0561	7.602478	0.37	4.9%
mKIAA0569	0.5554892	0.26	46.7%
mKIAA0570	32.4502	1.73	5.3%
mKIAA0578	0.7402072	0.41	55.8%
mKIAA0580	3.41239	0.29	8.4%
mKIAA0585	1.812318	0.33	18.5%
mKIAA0590	0.7757344	0.24	31.6%
mKIAA0595	0.4185764	0.15	35.6%
mKIAA0599	0.0229251	0.01	45.9%
mKIAA0601	5.505826	0.95	17.2%
mKIAA0606	0.6371942	0.26	40.6%
mKIAA0610	0.05781348	0.05	85.9%
mKIAA0612	3.173932	0.57	17.9%
mKIAA0614	0.2570708	0.10	38.5%
mKIAA0621	3.014076	0.59	19.5%
mKIAA0629	34.57794	0.64	1.9%
mKIAA0634	0.01600298	0.01	72.7%
mKIAA0636	0.10953752	0.04	35.5%
mKIAA0639	0.2515194	0.06	24.5%
mKIAA0640	0.2146403	0.14	66.5%
mKIAA0641	6.958352	0.79	11.4%
mKIAA0650	1.931564	0.60	31.3%
mKIAA0651	0.4177242	0.14	32.8%
mKIAA0656	10.976306	1.34	12.2%
mKIAA0665	0.08611862	0.03	37.5%
mKIAA0666	6.35049	2.71	42.7%
mKIAA0676	5.825228	2.46	42.2%
mKIAA0694	2.730024	0.41	14.9%
mKIAA0696	2.325906	0.97	41.8%
mKIAA0701	3.812706	0.62	16.3%
mKIAA0703	0.03408226	0.01	34.9%
mKIAA0716	1.1093608	1.58	142.5%
mKIAA0720	2.670922	0.49	18.3%
mKIAA0737	0.3172612	0.05	16.6%
mKIAA0744	0.8998016	0.38	42.5%
mKIAA0750	1.2590692	0.21	16.6%
mKIAA0753	1.580024	0.29	18.3%
mKIAA0763	41.58106	2.00	4.8%
mKIAA0769	0.0299534	0.01	17.6%
mKIAA0776	2.108978	0.26	12.3%
mKIAA0783	7.12524	0.85	11.9%
mKIAA0787	1.1448576	0.29	25.0%
mKIAA0788	6.210342	0.44	7.1%
mKIAA0797	4.580164	1.19	25.9%

mKIAA0804	0.02753698	0.01	25.7%
mKIAA0806	1.2445312	0.45	36.2%
mKIAA0817	23.1813	1.34	5.8%
mKIAA0824	0.1171358	0.07	58.3%
mKIAA0833	56.6901	2.17	3.8%
mKIAA0842	2.68965	0.37	13.7%
mKIAA0847	0.22661744	0.10	45.8%
mKIAA0849	0.2373414	0.04	17.2%
mKIAA0858	0.09032978	0.08	89.5%
mKIAA0866	0.000533854	0.00	154.8%
mKIAA0876	0.6525992	0.16	24.3%
mKIAA0881	3.469786	0.15	4.4%
mKIAA0884	0.327901	0.12	35.3%
mKIAA0885	0.5351294	0.18	33.6%
mKIAA0887	0.15577392	0.06	38.2%
mKIAA0889	4.302502	0.52	12.1%
mKIAA0892	6.727112	0.66	9.8%
mKIAA0893	0.1456636	0.07	45.8%
mKIAA0895	0.0554704	0.03	58.5%
mKIAA0896	9.084194	0.52	5.7%
mKIAA0897	10.755978	0.89	8.3%
mKIAA0899	0.2753256	0.11	40.8%
mKIAA0903	0.09998436	0.02	23.4%
mKIAA0904	4.191714	0.36	8.5%
mKIAA0909	1.1350014	0.46	40.2%
mKIAA0912	0.03626458	0.02	46.9%
mKIAA0919	16.35816	0.42	2.6%
mKIAA0922	0.517296	0.24	45.8%
mKIAA0923	9.663944	0.95	9.9%
mKIAA0933	3.67731	0.42	11.5%
mKIAA0946	4.365266	0.48	11.1%
mKIAA0947	0.8467866	0.65	76.7%
mKIAA0957	0.02836582	0.01	46.4%
mKIAA0961	0.3409122	0.05	15.1%
mKIAA0966	0.08159212	0.03	41.4%
mKIAA0968	0.01995308	0.00	15.0%
mKIAA0977	0.005902049	0.00	59.8%
mKIAA0981	0.46405296	0.66	142.2%
mKIAA0993	4.920826	0.63	12.8%
mKIAA0998	2.154218	0.18	8.2%
mKIAA1016	2.610392	0.20	7.7%
mKIAA1019	14.32944	0.61	4.3%
mKIAA1026	8.329384	1.14	13.7%
mKIAA1027	10.260536	1.28	12.4%
mKIAA1028	0.9285606	0.25	26.5%
mKIAA1038	0.181975	0.07	39.1%
mKIAA1039	0.919424	0.30	32.9%

mKIAA1049	8.937392	3.58	40.1%
mKIAA1052	0.07878754	0.06	78.8%
mKIAA1054	1.7860082	1.01	56.6%
mKIAA1055	0.05482584	0.02	37.9%
mKIAA1058	2.227448	0.47	21.0%
mKIAA1064	8.847682	0.65	7.4%
mKIAA1068	7.430606	2.69	36.2%
mKIAA1070	0.058674	0.02	39.3%
mKIAA1076	4.348208	0.37	8.5%
mKIAA1080	0.2778704	0.07	24.9%
mKIAA1084	0.05360926	0.01	22.0%
mKIAA1090	2.895718	0.29	9.8%
mKIAA1102	0.11169556	0.12	104.3%
mKIAA1105	4.29837	0.37	8.7%
mKIAA1108	0.16532164	0.12	70.6%
mKIAA1109	0.16118012	0.05	32.0%
mKIAA1110	13.10668	1.05	8.0%
mKIAA1111	3.464608	0.38	10.9%
mKIAA1112	1.537002	0.13	8.6%
mKIAA1122	0.2158482	0.11	52.4%
mKIAA1124	4.352076	0.76	17.5%
mKIAA1125	0.09997182	0.05	48.5%
mKIAA1128	0.5288386	0.21	39.7%
mKIAA1146	6.619894	0.87	13.2%
mKIAA1151	3.179928	0.34	10.7%
mKIAA1158	2.385926	1.59	66.6%
mKIAA1164	0.8128786	0.26	31.4%
mKIAA1178	0.9470904	0.33	34.5%
mKIAA1180	15.44418	2.97	19.3%
mKIAA1181	0.02794652	0.01	45.8%
mKIAA1188	0.9738242	0.17	17.0%
mKIAA1205	4.613828	0.72	15.7%
mKIAA1207	0.03954028	0.02	61.1%
mKIAA1211	10.033464	0.73	7.3%
mKIAA1214	0.03086322	0.01	23.2%
mKIAA1217	5.067754	0.37	7.4%
mKIAA1232	0.0655487	0.01	19.5%
mKIAA1238	3.565454	0.69	19.4%
mKIAA1239	1.216768	0.14	11.3%
mKIAA1244	0.15121936	0.07	43.3%
mKIAA1267	4.03638	0.74	18.3%
mKIAA1272	1.343536	0.20	14.8%
mKIAA1279	0.05131792	0.01	19.9%
mKIAA1281	0.6694536	0.54	81.4%
mKIAA1291	0.2807367	0.17	59.8%
mKIAA1294	0.0984735	0.05	48.2%
mKIAA1296	1.893458	0.47	25.1%

mKIAA1300	0.5639236	0.10	17.2%
mKIAA1308	0.2486962	0.10	40.2%
mKIAA1311	2.222302	0.47	21.0%
mKIAA1317	0.4919204	0.25	51.6%
mKIAA1334	0.16041652	0.29	181.9%
mKIAA1337	0.5104954	0.08	15.3%
mKIAA1358	3.75946	0.46	12.3%
mKIAA1369	0.0895421	0.06	66.8%
mKIAA1371	3.873844	1.37	35.2%
mKIAA1376	0.9829766	0.33	33.6%
mKIAA1380	7.374308	0.43	5.9%
mKIAA1382	0.2386462	0.10	40.7%
mKIAA1386	1.4298402	0.34	23.6%
mKIAA1401	0.542923	0.36	67.0%
mKIAA1403	0.3785642	0.06	16.1%
mKIAA1417	1.655146	0.46	27.9%
mKIAA1418	0.1673308	0.03	18.6%
mKIAA1422	6.74537	1.09	16.2%
mKIAA1426	0.2824676	0.15	53.6%
mKIAA1435	5.096168	1.01	19.7%
mKIAA1439	0.05909152	0.05	82.5%
mKIAA1441	1.0286394	0.37	35.8%
mKIAA1453	5.983006	0.36	6.0%
mKIAA1456	0.6719186	0.33	48.5%
mKIAA1458	0.4080194	0.21	51.6%
mKIAA1464	0.02684736	0.01	20.0%
mKIAA1486	2.033146	0.08	3.7%
mKIAA1506	0.4154882	0.08	19.8%
mKIAA1513	5.74384	0.38	6.6%
mKIAA1520	1.0018278	0.10	10.0%
mKIAA1524	0.1827256	0.08	42.6%
mKIAA1526	0.05601194	0.04	73.1%
mKIAA1527	0.3916288	0.15	38.7%
mKIAA1549	0.7168696	0.31	43.3%
mKIAA1558	2.110424	0.31	14.5%
mKIAA1560	0.4349308	0.16	36.8%
mKIAA1565	0.367539	0.13	36.4%
mKIAA1568	0.8992386	0.41	46.1%
mKIAA1575	1.924406	0.31	16.3%
mKIAA1577	0.1239976	0.02	16.4%
mKIAA1584	0.1280699	0.06	46.5%
mKIAA1598	0.02069468	0.01	26.4%
mKIAA1601	0.1208941	0.04	33.3%
mKIAA1605	3.93454	0.86	21.8%
mKIAA1606	1.920138	0.44	22.8%
mKIAA1609	0.11561406	0.06	51.1%
mKIAA1612	0.3471858	0.08	24.0%

mKIAA1626	1.8441506	0.75	40.5%
mKIAA1627	0.3435438	0.15	44.6%
mKIAA1632	3.080088	0.46	14.9%
mKIAA1633	0.2290364	0.15	67.2%
mKIAA1635	5.458746	0.26	4.8%
mKIAA1643	0.05583826	0.04	68.4%
mKIAA1668	3.518266	0.25	7.0%
mKIAA1672	0.016209386	0.01	37.9%
mKIAA1673	27.13922	1.71	6.3%
mKIAA1680	2.1313	0.48	22.6%
mKIAA1682	0.7249972	0.28	39.0%
mKIAA1685	0.1434942	0.03	24.0%
mKIAA1686	7.630308	1.46	19.1%
mKIAA1694	27.56066	1.76	6.4%
mKIAA1705	0.2614186	0.13	49.9%
mKIAA1707	0.196927964	0.39	198.7%
mKIAA1715	7.961992	0.66	8.3%
mKIAA1721	0.6944424	0.94	134.9%
mKIAA1724	2.509592	0.30	11.8%
mKIAA1726	1.882832	0.38	20.4%
mKIAA1732	0.11220142	0.04	36.4%
mKIAA1734	7.174794	1.47	20.5%
mKIAA1737	0.3634014	0.09	23.7%
mKIAA1739	0.11392678	0.04	39.0%
mKIAA1741	1.369388	0.13	9.7%
mKIAA1744	1.412632	0.13	9.3%
mKIAA1745	0.04386864	0.04	81.4%
mKIAA1747	0.537637	0.11	20.9%
mKIAA1756	0.24627656	0.13	54.7%
mKIAA1757	6.481048	0.70	10.8%
mKIAA1766	3.521156	0.61	17.2%
mKIAA1767	1.18458	0.08	6.8%
mKIAA1781	0.0487035	0.01	23.5%
mKIAA1790	0.0438147	0.02	51.4%
mKIAA1796	0.33001412	0.66	200.8%
mKIAA1823	0.02920836	0.01	25.5%
mKIAA1830	1.546276	0.20	13.1%
mKIAA1833	30.76446	2.14	7.0%
mKIAA1835	0.2275912	0.08	36.8%
mKIAA1839	0.04769584	0.01	17.5%
mKIAA1841	1.174204	0.51	43.7%
mKIAA1849	0.252959	0.10	40.9%
mKIAA1857	0.2558456	0.06	24.4%
mKIAA1860	5.69323	1.27	22.3%
mKIAA1870	0.5950034	0.06	10.7%
mKIAA1884	1.0423874	0.10	9.9%
mKIAA1889	9.978096	1.09	10.9%

mKIAA1896	1.7354044	0.53	30.8%
mKIAA1897	1.1282894	0.30	26.5%
mKIAA1902	2.999732	0.58	19.2%
mKIAA1911	1.2515284	0.28	22.7%
mKIAA1914	0.02650884	0.01	25.3%
mKIAA1917	1.0794598	0.15	14.1%
mKIAA1921	7.553454	0.22	2.9%
mKIAA1924	0.17544216	0.13	73.0%
mKIAA1933	1.580506	0.39	24.9%
mKIAA1939	0.003489674	0.00	136.9%
mKIAA1971	0.2419628	0.13	53.7%
mKIAA1973	7.380222	0.39	5.3%
mKIAA1980	0.704134	0.25	35.5%
mKIAA1981	5.760898	0.68	11.9%
mKIAA1983	0.02675718	0.01	19.2%
mKIAA1992	0.07072966	0.02	30.4%
mKIAA1994	1.1110348	0.34	30.4%
mKIAA1997	0.1904212	0.08	41.6%
mKIAA1998	0.690486	0.40	57.2%
mKIAA2000	0.2437647	0.26	107.1%
mKIAA2006	0.4077624	0.14	35.5%
mKIAA2037	0.12070662	0.04	33.1%
mKIAA3005	18.94408	1.62	8.6%
mKIAA3014	0.5294038	0.11	20.0%
mKIAA3015	1.1370302	0.09	8.1%
mKIAA3017	4.35517	0.77	17.6%
mKIAA3020	0.6632798	0.10	15.4%
mKIAA3026	0.09712912	0.04	38.1%
mKIAA3028	0.01480072	0.01	68.4%
mKIAA4006	0.3784126	0.20	52.1%
mKIAA4009	1.0310814	0.09	9.0%
mKIAA4020	2.565352	0.52	20.4%
mKIAA4026	0.11793948	0.03	21.2%
mKIAA4029	8.593962	0.76	8.8%
mKIAA4044	0.07428206	0.01	18.8%
mKIAA4045	0.08993114	0.03	28.4%
mKIAA4046	0.590591	0.35	58.5%
mKIAA4048	0.01258082	0.01	63.9%
mKIAA4051	0.7014392	0.12	17.6%
mKIAA4052	0.13000706	0.06	49.9%
mKIAA4069	0.038361282	0.04	108.1%
mKIAA4074	0.012887288	0.01	63.9%
mKIAA4075	5.487408	1.01	18.5%
mKIAA4082	3.189306	0.37	11.5%
mKIAA4083	0.1727078	0.08	45.3%
mKIAA4089	0.6238064	0.27	42.8%
mKIAA4091	4.79137	0.15	3.1%

mKIAA4093	89.83888	4.19	4.7%
mKIAA4106	0.4124498	0.11	25.5%
mKIAA4110	0.7827924	0.33	42.5%
mKIAA4112	2.047952	0.50	24.2%
mKIAA4114	0.009859512	0.01	132.3%
mKIAA4116	1.69289	0.26	15.1%
mKIAA4118	0.332472	0.11	33.1%
mKIAA4119	48.93132	8.33	17.0%
mKIAA4123	0.2098174	0.08	37.3%
mKIAA4128	0.256112	0.06	22.1%
mKIAA4143	8.553978	0.53	6.2%
mKIAA4144	0.8205338	0.28	33.8%
mKIAA4145	0.8913346	0.34	38.7%
mKIAA4155	1.0978794	0.19	17.8%
mKIAA4159	2.04207	0.44	21.5%
mKIAA4162	2.159588	0.88	40.8%
mKIAA4163	0.7946514	0.61	76.4%
mKIAA4170	0.07845638	0.02	31.5%
mKIAA4185	3.189538	0.90	28.3%
mKIAA4187	1.448339	0.77	53.5%
mKIAA4190	0.2961592	0.13	44.4%
mKIAA4191	11.62898	0.35	3.0%
mKIAA4192	3.10573	1.08	34.7%
mKIAA4193	0.0495623	0.04	80.9%
mKIAA4196	4.016198	0.46	11.5%
mKIAA4205	0.8155326	0.11	14.0%
mKIAA4211	0.0257885	0.02	88.8%
mKIAA4216	0.3351436	0.39	117.6%
mKIAA4222	1.1318082	0.49	43.7%
mKIAA4226	0.0412608	0.01	13.0%
mKIAA4231	0.0339978	0.00	14.4%
mKIAA4237	0.3843928	0.31	81.4%
mKIAA4245	0.026218766	0.01	40.6%
mKIAA4247	0.4524208	0.42	93.8%
mKIAA4252	0.2252437	0.15	68.5%
mKIAA4253	0.40214	0.18	45.3%
mKIAA4256	9.355028	1.20	12.9%
MKK7	0.2357942	0.06	26.3%
Mkks	6.810754	1.15	16.9%
Mkl1	10.65662	0.52	4.9%
Mkl2	2.763208	0.21	7.4%
Mkln1	14.99312	0.80	5.3%
Mknk1	5.614766	0.30	5.3%
Mknk2	10.376572	1.01	9.8%
MKP-7	0.8315676	0.70	84.2%
Mkrn1	58.5516	3.22	5.5%
Mkrn2	12.30016	0.47	3.9%

Mkrn3	12.5863	2.53	20.1%
Mks1	1.68202	0.19	11.0%
Mkx	2.470754	0.44	18.0%
Mlana	0.01194008	0.03	223.6%
Mlc1	19.76408	1.15	5.8%
Mlf1	0.7584432	0.11	14.2%
Mlf1ip	1.695412	0.36	21.5%
Mlf2	123.1988	8.85	7.2%
Mlh1	2.13244	0.12	5.5%
Mlh3	3.842176	0.22	5.7%
mLIP1	2.046048	0.81	39.6%
Mlkl	0.01603318	0.02	95.5%
Mll1	4.382978	0.38	8.6%
Mll2	5.196852	0.23	4.4%
Mll3	4.15261	0.36	8.6%
Mll5	25.82674	1.52	5.9%
Mllt1	10.379866	1.06	10.2%
Mllt10	3.836828	0.14	3.6%
Mllt11	270.762	27.30	10.1%
Mllt3	5.399212	0.47	8.7%
Mllt4	11.54174	0.50	4.3%
Mllt6	5.91335	0.78	13.1%
Mllt7	0.10115874	0.04	44.1%
Mln51	13.20816	1.29	9.8%
Mlph	0.0251876	0.02	86.9%
mlr2	0.12757312	0.10	77.8%
Mlstd1	1.1468604	0.15	13.2%
Mlstd2	23.79414	1.01	4.2%
Mltk	0.3692424	0.11	30.9%
Mlx	10.357884	0.47	4.5%
Mlxip	3.060222	0.18	5.8%
Mlxipl	0.049629504	0.03	55.0%
Mlycd	3.898046	1.04	26.7%
Mlyk	0.0827594	0.02	21.5%
Mlze	0.003524862	0.01	156.0%
Mmaa	3.60113	0.36	10.1%
Mmab	20.34844	1.11	5.5%
Mmachc	6.664298	0.47	7.1%
MmCdt1	0.0729772	0.03	41.8%
Mmd	28.74326	2.95	10.3%
Mmd2	2.409764	0.27	11.3%
Mme	2.63737	0.17	6.3%
Mmel1	0.300093	0.08	26.2%
MMET-1	0.05865146	0.03	47.7%
Mmh	2.13994	0.16	7.5%
Mmip	0.2753284	0.06	21.9%
MmKIF17	1.866632	0.35	18.9%

Mmp10	0.00367998	0.01	223.6%
Mmp11	1.95391	0.24	12.4%
Mmp12	0.04015394	0.03	75.4%
Mmp13	0.00763382	0.02	223.6%
Mmp14	8.656288	1.68	19.4%
Mmp15	7.72026	0.41	5.3%
Mmp16	5.002116	0.49	9.7%
Mmp17	6.809542	0.51	7.5%
Mmp19	1.0152128	0.14	13.8%
Mmp1a	0.00374192	0.01	223.6%
Mmp1b	0.00001422	0.00	174.8%
Mmp2	10.878886	2.59	23.8%
Mmp20	0	0.00	
Mmp21	0.00733428	0.01	150.6%
Mmp23	0.0510459	0.04	70.9%
Mmp24	17.99606	1.73	9.6%
Mmp25	0.02618918	0.02	68.7%
Mmp27	0	0.00	
Mmp28	0.3445118	0.08	22.1%
Mmp3	0.02704554	0.02	69.0%
Mmp7	0.00853774	0.02	223.6%
Mmp8	0	0.00	
Mmp9	1.596024	0.15	9.5%
MMR	1.2500232	0.50	40.2%
Mmrn1	0.001272384	0.00	223.6%
Mmrn2	0.010516566	0.01	89.2%
Mms19	14.81922	0.57	3.9%
Mn1	5.189246	0.85	16.3%
mNadrin1	0.2367044	0.11	48.4%
Mnat1	4.49954	0.26	5.8%
MNCb-1706	25.39954	2.91	11.4%
Mnd1	0.1500234	0.11	71.1%
Mnda	0.03133714	0.04	139.8%
mNfs1	0.7342918	0.30	41.5%
Mnk2	0.7226788	0.36	49.2%
Mns1	0.7360708	0.05	7.1%
Mnt	12.27158	0.85	6.9%
Mnx1	.3017108	0.28	9.4%
Moap1	21.03092	1.34	6.3%
Mob2	0.7475896	0.34	45.1%
Mobk1b	8.107294	0.68	8.4%
Mobkl1a	7.24587	0.49	6.8%
Mobkl1b	5.840276	0.64	10.9%
Mobkl2a	5.936642	1.15	19.3%
Mobkl2b	0.531344	0.05	9.5%
Mobkl2c	0.6052334	0.12	19.1%
Mobkl3	41.8255	1.84	4.4%

Mobp	0.20043706	0.19	93.0%
Mocos	0.05042188	0.03	67.8%
Mocs1	2.448378	0.21	8.7%
Mocs2	23.14004	0.70	3.0%
Mod1	13.47204	0.83	6.2%
Mog	0.6748742	0.15	22.1%
Mogat1	0.01988738	0.02	92.3%
Mogat2	0.00941414	0.01	150.7%
MOK	0.30846042	0.21	69.4%
Mon1a	3.606952	0.24	6.7%
Mon1b	5.335514	0.60	11.3%
Mon2	15.88006	0.97	6.1%
Mopt	1.8580208	1.67	89.6%
MOR	0.572924104	0.69	120.3%
Morc	0.00721262	0.01	129.6%
Morc1	0.013507066	0.01	51.3%
Morc2a	9.48994	0.30	3.2%
Morc2b	0.2114558	0.02	10.9%
Morc3	5.86847	0.13	2.2%
Morc4	1.882358	0.17	9.2%
Morf4l1	9.720862	0.62	6.4%
Morf4l2	100.37252	5.55	5.5%
Morn1	0.7111248	0.08	11.7%
Morn2	7.91185	1.99	25.2%
Morn3	0.01343688	0.02	138.9%
Mos	0.03175356	0.03	91.8%
Mosc1	0.01249514	0.01	59.8%
Mosc2	8.12752	0.85	10.4%
Mospd1	4.522758	0.44	9.7%
Mospd2	5.705012	0.31	5.4%
Mospd3	19.43324	1.65	8.5%
Mov10	0.7987682	0.09	11.7%
Mov10l1	0.001547966	0.00	223.6%
Moxd1	0.2897736	0.12	40.3%
Moxd2	0	0.00	
Mpa2l	0.272386	0.06	21.0%
Mpdu1	31.8237	2.44	7.7%
Mpdz	3.189388	0.26	8.1%
Mpeg1	0.4822648	0.11	22.1%
Mpg	6.80163	0.78	11.5%
mPHLL2	0.2956038	0.09	31.5%
Mphosph1	0.751513	0.15	19.6%
Mphosph10	4.022598	0.23	5.6%
Mphosph6	3.20546	0.36	11.1%
Mphosph8	15.99114	0.66	4.2%
Mphosph9	3.435712	0.34	10.0%
Mpi	19.5697	0.48	2.5%

Mpl	0	0.00	
mPLD1	0.0448197	0.04	91.5%
Mpnd	21.54018	1.61	7.5%
Mpo	0.0104948	0.02	173.7%
Mporc-b	0.9689552	0.07	7.4%
Mpp1	17.20376	0.51	3.0%
Mpp2	25.35016	1.65	6.5%
Mpp3	9.140646	0.85	9.3%
Mpp4	0.12963476	0.03	22.3%
Mpp5	4.88665	0.92	18.8%
Mpp6	16.31804	0.75	4.6%
Mpp7	5.851428	0.50	8.5%
Mppe1	5.037776	1.28	25.4%
Mpped1	4.79698	0.25	5.2%
Mpped2	13.03964	1.08	8.3%
mpp-GalNAc-T12	0.16033566	0.10	64.6%
mpp-GalNAc-T14	0.02044028	0.00	17.2%
mpp-GalNAc-T17(O6)	0.07414866	0.02	28.4%
Mpst	8.51639	1.05	12.3%
Mpv17	13.76542	0.61	4.4%
Mpv17l	20.32318	1.73	8.5%
Mpz	0.02595506	0.02	94.1%
Mpzl1	11.145744	1.20	10.7%
Mpzl2	0.4994926	0.16	31.9%
Mpzl3	0.3747848	0.13	34.3%
Mr1	0.71699	0.13	17.7%
Mrap	0.0043244	0.01	223.6%
Mras	16.09304	0.82	5.1%
Mrc1	0.035526	0.02	42.4%
Mrc2	2.512394	0.46	18.4%
Mrcl	0.00530794	0.01	137.1%
M-rdgB_beta	0.04035684	0.01	17.1%
Mre11a	1.688714	0.23	13.5%
Mreg	2.52705	0.28	11.2%
Mrfap1	65.85826	1.65	2.5%
Mrg1	35.74448	4.98	13.9%
MRG15	1.561338	0.47	29.9%
Mrg2	11.03872	0.64	5.8%
Mrgpra1	0	0.00	
Mrgpra2	0	0.00	
Mrgpra3	0	0.00	
Mrgpra4	0	0.00	
Mrgpra6	0	0.00	
Mrgprb1	0	0.00	
Mrgprb2	0	0.00	
Mrgprb3	0	0.00	
Mrgprb4	0	0.00	

Mrgprb5	0	0.00	
Mrgprb8	0	0.00	
Mrgprd	0	0.00	
Mrgpre	6.119498	0.36	6.0%
Mrgprf	0.222691	0.09	39.9%
Mrgprg	0	0.00	
Mrgprh	0.1329542	0.03	20.0%
Mrgprx1	0	0.00	
Mrgprx2	0	0.00	
Mrm1	6.608592	0.31	4.8%
Mrmt1	2.16224	0.39	18.1%
Mro	1.580764	0.23	14.3%
Mrp63	7.321794	0.40	5.4%
Mrpl1	7.682898	0.69	9.0%
Mrpl10	29.00686	1.27	4.4%
Mrpl11	27.76644	1.37	4.9%
Mrpl12	24.54644	1.51	6.1%
Mrpl13	11.49352	1.15	10.0%
MRPL13	4.025148	0.55	13.7%
Mrpl14	14.76152	1.66	11.3%
Mrpl15	19.5741	1.51	7.7%
Mrpl16	20.75954	1.97	9.5%
Mrpl17	12.1661	1.28	10.6%
Mrpl18	15.01314	1.16	7.7%
Mrpl19	4.647648	0.38	8.2%
Mrpl2	19.13342	1.50	7.8%
Mrpl20	37.23378	3.05	8.2%
Mrpl21	8.071586	0.73	9.0%
Mrpl22	3.268958	0.48	14.8%
Mrpl23	0.2170791	0.14	65.8%
Mrpl24	13.3844	0.88	6.6%
Mrpl27	11.33702	0.84	7.4%
Mrpl28	19.90474	1.28	6.4%
Mrpl3	15.81618	1.19	7.5%
Mrpl30	16.2032	1.31	8.1%
Mrpl32	12.1796	0.91	7.5%
Mrpl33	25.66058	2.80	10.9%
MRPL33	0.182427	0.03	16.0%
Mrpl34	14.52352	0.86	5.9%
Mrpl35	12.1247	0.22	1.8%
Mrpl36	11.11406	0.73	6.6%
Mrpl37	20.38972	1.23	6.0%
Mrpl38	21.03814	0.86	4.1%
Mrpl39	15.11018	0.61	4.0%
Mrpl4	11.8481	0.57	4.8%
MRPL4	2.26804	0.41	18.1%
Mrpl40	13.24862	1.24	9.3%

Mrpl41	11.17064	0.81	7.2%
Mrpl43	32.72046	2.61	8.0%
Mrpl44	9.796996	0.60	6.2%
Mrpl45	9.813302	0.29	2.9%
Mrpl46	10.719324	1.53	14.3%
Mrpl47	8.79398	0.67	7.7%
Mrpl48	6.528572	1.64	25.1%
Mrpl49	18.0389	0.42	2.3%
Mrpl50	18.56118	1.15	6.2%
Mrpl51	9.851762	0.85	8.6%
Mrpl52	23.65104	3.41	14.4%
Mrpl53	4.997758	1.32	26.5%
Mrpl54	13.10906	1.50	11.4%
Mrpl55	9.433846	0.74	7.9%
Mrpl9	16.21372	0.61	3.8%
Mrpplf4	0	0.00	
Mrps10	11.82556	1.32	11.2%
Mrps11	6.287864	1.05	16.7%
MRPS11	1.534831	0.53	34.8%
Mrps12	18.2058	1.28	7.0%
Mrps14	11.60346	0.45	3.9%
Mrps15	19.66108	0.85	4.3%
Mrps16	17.13206	1.20	7.0%
Mrps17	16.9928	1.31	7.7%
MRPS17	1.5369368	0.50	32.6%
Mrps18a	32.8	2.27	6.9%
Mrps18b	6.569086	0.85	13.0%
Mrps18c	10.047864	1.39	13.8%
Mrps2	12.433928	1.94	15.6%
MRP-S2	2.336696	1.55	66.2%
Mrps21	11.8801	1.22	10.2%
Mrps22	13.42382	0.68	5.0%
Mrps23	15.75364	0.57	3.6%
Mrps24	12.77906	1.15	9.0%
Mrps25	10.014964	0.23	2.3%
Mrps26	17.3999	1.39	8.0%
Mrps27	16.33272	1.47	9.0%
Mrps28	8.674802	1.12	12.9%
Mrps30	9.492844	0.58	6.1%
Mrps31	10.77324	0.53	4.9%
Mrps33	2.948968	0.46	15.5%
Mrps34	13.59688	0.35	2.6%
Mrps35	14.13506	1.36	9.6%
Mrps36	2.124304	0.35	16.3%
Mrps5	7.312274	0.27	3.6%
MRPS5	6.080884	1.14	18.8%
Mrps6	31.86652	0.81	2.5%

Mrps7	19.73406	1.92	9.7%
Mrps9	12.72656	0.20	1.6%
Mrrf	8.652012	0.70	8.1%
Mrs2l	7.299664	0.85	11.7%
Mrto4	3.40246	0.32	9.4%
Mrvi1	0.4176558	0.10	23.1%
Ms4a1	0	0.00	
Ms4a10	0	0.00	
Ms4a13	0	0.00	
Ms4a2	0.08937172	0.08	88.4%
Ms4a3	0	0.00	
Ms4a4b	0	0.00	
Ms4a4c	0.00619974	0.01	223.6%
Ms4a4d	0.22656148	0.10	45.5%
Ms4a5	0	0.00	
Ms4a6b	0	0.00	
Ms4a6c	0	0.00	
Ms4a6d	0.01062658	0.01	137.0%
Ms4a7	0	0.00	
Ms4a8a	0	0.00	
Msc	1.376314	0.14	10.1%
M-Septin	0.04657602	0.01	25.0%
Msgn1	0	0.00	
Msh2	11.7606	0.37	3.1%
Msh3	5.727616	0.36	6.3%
Msh4	0.02486758	0.01	56.1%
Msh5	0.06161722	0.03	49.4%
Msh6	5.630238	0.23	4.0%
Msi1	5.374942	0.64	11.9%
Msi2	11.89854	0.80	6.7%
msi2	0.23304296	0.12	51.4%
Msi2h	0.33909242	0.28	82.7%
Msl2l1	9.113382	0.34	3.7%
Msl31	8.477574	0.39	4.6%
Msln	0.02106232	0.02	72.4%
Msmb	0	0.00	
mSMO	1.2123014	0.57	46.9%
Msn	5.625532	0.44	7.9%
mSox5L	0.17720302	0.08	42.5%
MSP58	0.3017544	0.15	50.1%
Msr1	0.01062996	0.01	61.7%
Msr2	0	0.00	
Msra	4.426048	0.50	11.3%
Msrb2	2.525028	0.37	14.5%
Msrb3	0.683337	0.09	13.6%
mSSH-2L	0.04236838	0.02	45.1%
mSSH-3L	1.151232	0.52	44.9%

Mst1	0.00483778	0.01	223.6%
Mst1r	0.09550594	0.04	39.2%
Mstn	0.00738738	0.01	138.6%
Msto1	11.9727	1.07	9.0%
Msx1	1.335082	0.10	7.5%
Msx2	0.12264248	0.07	54.3%
Msx3	0.4521314	0.07	15.4%
Msy2	0.954696	0.36	37.9%
Mt1	113.2858	6.98	6.2%
Mt2	52.76942	4.90	9.3%
Mt3	65.19134	6.40	9.8%
mt3-mmp	0.1951194	0.07	38.3%
Mt4	0	0.00	
Mta1	17.4329	0.53	3.0%
Mta2	12.06502	0.55	4.6%
Mta3	18.4235	2.69	14.6%
Mtap	8.223156	0.45	5.5%
Mtap1a	89.3444	5.10	5.7%
Mtap1b	124.9308	9.48	7.6%
Mtap1s	15.21424	1.03	6.8%
Mtap2	176.8036	6.09	3.4%
Mtap4	129.8984	4.30	3.3%
Mtap6	69.5427	3.91	5.6%
Mtap7	1.715982	0.11	6.3%
Mtap7d1	39.5731	1.30	3.3%
Mtap7d2	61.59956	2.02	3.3%
Mtap7d3	0.07315828	0.02	32.5%
Mtap9	18.21126	1.07	5.9%
Mtb	0.2901892	0.11	38.7%
Mtbp	0.6718334	0.11	17.1%
Mtch1	151.4932	6.00	4.0%
Mtch2	27.95226	1.43	5.1%
Mtcp1	5.21152	0.41	7.8%
Mtdh	20.80472	1.01	4.8%
mTdIF1	0.0796239	0.02	20.0%
Mterf	0.7426864	0.11	15.1%
Mterfd1	10.319546	2.34	22.7%
Mterfd2	4.320192	0.15	3.6%
Mterfd3	7.238458	0.28	3.9%
Mtf1	3.81903	0.50	13.0%
Mtf2	7.032682	0.37	5.2%
Mtfmt	3.385134	0.26	7.7%
Mtfr1	2.120956	0.17	8.2%
Mtg1	8.889512	0.40	4.5%
Mthfd1	8.149254	0.37	4.6%
Mthfd1l	5.83586	0.18	3.1%
Mthfd2	15.55688	2.17	14.0%

Mthfr	6.216592	0.60	9.7%
Mthfs	1.509638	0.36	23.8%
Mthfsd	6.313006	0.48	7.5%
mThy28	1.637114	0.11	6.8%
Mtif2	9.440496	0.32	3.4%
Mtif3	6.447976	0.86	13.4%
Mtl5	0.08733696	0.03	33.1%
Mtm1	2.862016	0.30	10.4%
Mtmr1	17.080496	5.49	32.1%
Mtmr10	1.912984	0.20	10.5%
Mtmr11	1.1257132	0.11	10.1%
Mtmr12	1.958358	0.23	11.8%
Mtmr14	6.367416	0.37	5.7%
Mtmr2	10.26765	0.39	3.8%
Mtmr3	17.04196	0.74	4.4%
Mtmr4	28.69722	0.50	1.8%
Mtmr6	30.09686	0.32	1.1%
Mtmr7	28.54474	2.25	7.9%
Mtmr9	27.9347	1.11	4.0%
mt-Nd4	1829.65	271.59	14.8%
Mtnr1a	0.00981036	0.01	141.4%
Mtnr1b	0	0.00	
Mto1	4.828334	0.33	6.8%
mTOP3beta	3.568848	0.30	8.4%
mTPK1	0.04562316	0.01	18.2%
Mtpn	88.28344	1.95	2.2%
MtPolB	0.71470432	0.41	57.5%
Mtr	1.582502	0.14	8.8%
Mtr1	0.0251941	0.01	30.0%
Mtrf1	2.94496	0.31	10.6%
Mtrf1l	1.476042	0.11	7.1%
mTRF2	0.6869488	0.20	29.7%
Mtrr	2.545614	0.07	2.6%
Mtss1	28.74646	1.79	6.2%
Mttp	4.221834	0.45	10.6%
Mtus1	4.069992	0.37	9.0%
Mtvr2	0.4390552	0.18	41.0%
Mtx1	12.46114	0.98	7.9%
Mtx2	19.89436	1.56	7.8%
mUBPY	1.2149468	0.54	44.7%
Muc1	0.4228416	0.07	17.7%
Muc10	0	0.00	
Muc13	0.00498516	0.01	138.1%
Muc15	0.0935859	0.03	35.8%
Muc19	0.00065985	0.00	223.6%
Muc2	0.13845744	0.03	24.6%
MUC2	0	0.00	

Muc20	0.00206978	0.00	223.6%
Muc3	0.0256687	0.03	114.3%
Muc4	0.001515518	0.00	137.3%
Muc5ac	0.3521174	0.20	58.1%
Muc5b	0.03864176	0.04	106.6%
Muc6	0.04387248	0.03	57.0%
Mug1	0.01917186	0.00	26.0%
Mug2	0.011668788	0.00	33.8%
MUG3	0.01984434	0.02	95.4%
mUGT1	0.7003172	0.25	35.3%
mUGTrel8	0.03380478	0.02	47.8%
Mule	1.4667658	0.68	46.2%
Mum1	8.467532	0.50	5.9%
Mum1l1	3.035424	0.23	7.6%
MuORC4	1.1083188	0.43	39.0%
Mup1	0	0.00	
Mup2	0	0.00	
Mup3	0	0.00	
Mup4	0	0.00	
Mup5	0	0.00	
Mupcdh	0.0090517	0.01	161.3%
Mus81	5.092966	0.11	2.1%
Musk	0.02130085	0.02	77.9%
muspaox	1.3597292	0.51	37.7%
Mustn1	0	0.00	
Mut	12.14842	0.97	8.0%
Muted	6.512988	0.72	11.1%
Mutyh	0.6117964	0.15	25.0%
mVAMP-1	1.2248904	0.74	60.1%
Mvd	27.56418	4.40	16.0%
MVD	0.10478474	0.02	14.6%
Mvk	27.6256	6.43	23.3%
Mvp	1.0786774	0.12	11.5%
Mx1	0.02178088	0.02	80.3%
Mx2	0.10119354	0.04	38.2%
Mxd1	2.668384	0.13	4.9%
Mxd3	0.5022068	0.07	14.6%
Mxd4	8.12883	1.26	15.5%
Mxi1	7.941758	0.62	7.8%
Mxra7	5.973012	0.74	12.3%
Mxra8	1.880808	0.47	25.2%
Myadm	49.60704	1.94	3.9%
Myb	0.09734838	0.02	17.2%
Mybbp1a	12.37928	0.53	4.3%
Mybl1	1.96794	0.29	14.9%
Mybl2	0.2114228	0.06	28.0%
Mybpc2	0.014575432	0.01	98.7%

Mybpc3	0.001045774	0.00	223.6%
Mybph	0.61269274	0.56	91.2%
Mybphl	0	0.00	
Myc	7.214546	0.92	12.8%
Mycbp	4.02224	0.28	6.9%
Mycbpap	0.1476736	0.05	30.8%
Mycl1	3.079354	0.26	8.5%
Mycn	1.409876	0.17	12.0%
Mycs	0	0.00	
Myct1	0.00253452	0.01	223.6%
Myd116	15.01142	0.88	5.8%
Myd88	1.1989838	0.23	19.2%
Myef2	22.14056	1.36	6.2%
Myf5	0.00367428	0.01	223.6%
Myf6	0	0.00	
Myg1	8.46337	0.70	8.2%
Myh1	0.01243263	0.01	88.1%
Myh10	34.5706	2.18	6.3%
Myh11	0.04499368	0.02	34.6%
Myh13	0.01244692	0.02	142.0%
Myh14	1.863376	0.36	19.4%
Myh2	0.04859326	0.01	26.2%
Myh3	0.004294656	0.01	129.2%
Myh4	0.002543676	0.00	94.9%
Myh6	0.02036916	0.01	50.1%
Myh7	0.08980414	0.02	17.3%
Myh7b	0.281449	0.07	24.2%
Myh8	0.03683174	0.01	38.0%
Myh9	5.26957	0.50	9.5%
Myl1	0.01413714	0.02	137.0%
Myl2	0	0.00	
Myl3	0.0318344	0.03	79.3%
Myl4	0.5423196	0.10	19.2%
Myl6	38.50686	3.26	8.5%
Myl6b	17.67224	0.63	3.6%
Myl7	0.01556174	0.03	223.6%
Myl9	1.576824	0.40	25.6%
Mylc2b	98.06442	5.25	5.4%
Mylc2pl	0	0.00	
Mylip	3.23315	0.30	9.4%
Mylk	0.8192048	0.20	24.9%
Mylk2	0.0083451	0.01	112.4%
Mylpf	0.2148586	0.08	38.2%
Myn	2.392792	0.89	37.0%
Mynn	3.66317	0.17	4.6%
myo_10	0.6572928	0.32	49.4%
Myo10	3.602882	0.37	10.2%

Myo15	0.00783115	0.01	76.1%
Myo15b	0.004716406	0.01	142.2%
Myo16	7.73359	0.56	7.2%
Myo18a	20.53564	1.28	6.2%
Myo18b	0.017544862	0.01	70.2%
Myo1a	0.10415954	0.05	47.4%
Myo1b	4.035786	0.27	6.6%
Myo1c	5.342632	0.27	5.1%
Myo1d	0.5060142	0.04	8.7%
Myo1e	0.776904	0.15	19.5%
Myo1f	0.01276933	0.01	50.3%
Myo1g	0.001424702	0.00	223.6%
Myo1h	0.04233512	0.02	56.5%
Myo3a	0.002622504	0.00	137.3%
Myo3b	0.022676874	0.02	77.1%
Myo5a	39.2577	2.58	6.6%
Myo5b	1.71533	0.16	9.4%
Myo5c	0.06560448	0.02	31.0%
Myo6	3.601084	0.16	4.5%
Myo7a	0.013429294	0.01	80.7%
Myo7b	0.006745174	0.01	94.4%
Myo9a	11.32718	1.09	9.7%
Myo9b	5.7898	0.52	9.0%
Myoc	0.08586268	0.04	42.4%
Myocd	0.011295582	0.01	83.8%
Myod1	0.0070135	0.01	139.8%
Myog	0.01656568	0.02	120.5%
Myohd1	1.639552	0.14	8.8%
Myom1	0.12458774	0.06	47.0%
Myom2	0.021162396	0.01	39.0%
Myom3	0.001240796	0.00	223.6%
Myot	0	0.00	
Myoz1	0.00401704	0.01	223.6%
Myoz2	0.01646912	0.02	92.7%
Myoz3	0	0.00	
Mypn	0.01925317	0.01	51.0%
Myrip	9.866518	0.57	5.8%
Mysm1	4.311994	0.31	7.1%
MysPDZ	9.58201	0.79	8.2%
Myst1	6.9792	0.94	13.5%
Myst2	24.98934	0.39	1.6%
Myst3	5.595422	0.35	6.2%
Myst4	5.95396	0.46	7.7%
Myt1	3.033346	0.28	9.2%
Myt1l	28.56784	1.36	4.8%
Mzf1	1.307358	0.13	9.6%
N28178	30.24794	1.12	3.7%

N30.7TCRA	0	0.00	
N4wbp5A	0.7370922	0.24	32.5%
N6amt1	4.635544	0.74	16.0%
N6amt2	12.0723	0.61	5.1%
NAAA	0.2969728	0.20	69.0%
Naalad2	0.06947728	0.02	22.7%
Naaladl1	0.05309556	0.04	76.7%
Nab1	4.139436	0.60	14.5%
Nab2	1.618308	0.17	10.6%
Nabp1	0.3434502	0.07	19.0%
Naca	8.680308	0.91	10.5%
Nact	0.057207022	0.05	83.2%
Nadk	9.520986	0.58	6.1%
Nadsyn1	1.41671	0.10	6.7%
Naga	11.85792	1.17	9.9%
Nagk	21.23482	2.70	12.7%
Naglu	3.108044	0.22	7.0%
Nagpa	1.1464612	0.15	13.5%
Nags	0.00393898	0.01	223.6%
Naip1	0.04345266	0.02	35.3%
Naip2	0.014353674	0.01	43.3%
Naip5	0.01017378	0.01	80.2%
Naip7	0.002507724	0.00	103.6%
NAKAP95	1.909598	0.35	18.4%
Nalcn	21.53206	0.74	3.4%
Nalp12	0.00468884	0.01	223.6%
Nalp1b	0	0.00	
Nalp4c	0.00137436	0.00	223.6%
Nanog	0.00192178	0.00	223.6%
Nanos1	7.806708	0.62	7.9%
Nanos2	0.1950558	0.04	18.0%
Nanos3	0.8247102	0.21	25.4%
Nanp	0.01169212	0.02	138.7%
Nans	12.7173	1.14	9.0%
Nap1l1	25.85762	1.40	5.4%
Nap1l2	87.86474	2.72	3.1%
Nap1l3	39.65446	2.46	6.2%
Nap1l4	59.551	3.78	6.3%
Nap1l5	454.8046	29.47	6.5%
Napa	58.20128	0.89	1.5%
Napb	129.5574	3.61	2.8%
Napg	50.8092	1.37	2.7%
Napor-1	4.427774	1.14	25.8%
Naprt1	0.4028144	0.13	33.1%
Napsa	0.10430136	0.05	48.5%
Narf	16.3939	1.28	7.8%
Narfl	6.551118	0.19	3.0%

Narg1	14.29712	0.85	5.9%
Narg1l	1.2253	0.11	9.3%
Narg2	2.413488	0.82	34.2%
Nars	91.69662	6.36	6.9%
Nars2	3.34707	0.16	4.6%
Nasp	11.52602	0.90	7.8%
Nat1	0.03283516	0.04	107.6%
Nat10	6.054726	0.45	7.5%
Nat11	9.64278	0.59	6.2%
Nat12	13.87892	0.81	5.9%
Nat13	24.6515	0.50	2.0%
Nat14	9.81911	0.22	2.2%
Nat2	0.1806411	0.07	39.6%
Nat3	0	0.00	
Nat5	13.1453	1.25	9.5%
Nat6	2.595244	0.12	4.7%
Nat8l	49.89884	4.01	8.0%
Nat9	4.541044	0.15	3.2%
Nav1	11.36454	1.18	10.4%
Nav3	5.440876	0.57	10.5%
nbea	19.94912	2.54	12.7%
Nbea	11.04706	0.86	7.8%
Nbeal1	1.502312	0.27	18.1%
Nbl1	4.395056	0.98	22.3%
Nbn	4.070542	0.25	6.1%
Nbr1	30.50564	1.56	5.1%
NC8	0.4706112	0.13	27.1%
Ncald	41.82994	0.65	1.6%
Ncam1	116.4502	6.26	5.4%
Ncam2	33.11438	2.81	8.5%
Ncan	11.65218	0.28	2.4%
Ncapd2	3.9308	0.44	11.1%
Ncapd3	3.580674	0.16	4.4%
Ncapg2	0.9735978	0.13	13.2%
Ncaph	0.6522552	0.12	18.6%
Ncaph2	19.73092	0.49	2.5%
Ncbp2	19.00642	0.99	5.2%
Ncdn	171.9668	12.74	7.4%
Ncf1	0.6322425	0.49	77.6%
Ncf2	0.00961241	0.01	106.8%
Ncf4	0	0.00	
Nck1	12.04132	0.46	3.8%
Nck2	5.437166	0.77	14.2%
Nckap1	102.3932	2.19	2.1%
Nckap1l	0.142337	0.01	8.9%
Nckipsd	13.9206	1.13	8.1%
Ncl	40.82992	1.78	4.4%

Ncln	16.6159	0.70	4.2%
Ncoa1	9.528342	0.56	5.9%
Ncoa2	8.81455	0.37	4.2%
Ncoa3	5.490934	0.34	6.2%
Ncoa4	0.526758	0.06	11.6%
Ncoa5	10.374558	0.93	9.0%
Ncoa6	5.207602	0.22	4.2%
Ncoa7	27.42938	0.61	2.2%
Ncor1	32.18032	2.24	7.0%
Ncor2	9.221384	1.21	13.2%
Ncr1	0	0.00	
Ncstn	22.35702	1.49	6.7%
NCX1	1.0162226	1.53	150.6%
Ndc80	0.4869914	0.16	33.6%
Nde1	5.547674	0.37	6.6%
Ndel1	31.73222	1.36	4.3%
Ndfip1	318.37	6.08	1.9%
Ndfip2	56.55822	1.67	3.0%
Ndg1	0.00703194	0.01	137.3%
Ndg2	48.4918	1.22	2.5%
Ndn	133.9048	8.59	6.4%
Ndnl2	9.311256	0.47	5.0%
Ndor1	5.48908	0.57	10.5%
Ndph	1.98758	0.04	2.0%
Ndr3	0.02679058	0.01	47.8%
Ndrg1	14.4083	0.71	4.9%
Ndrg2	36.5546	3.14	8.6%
Ndrg3	108.608	1.26	1.2%
Ndrg4	486.8336	14.28	2.9%
ndrp	0.414721	0.22	52.1%
Ndst1	25.67978	2.36	9.2%
Ndst2	3.309074	0.21	6.4%
Ndst3	3.798496	0.07	2.0%
Ndst4	3.43863	0.22	6.5%
Ndufa1	61.69252	3.99	6.5%
Ndufa10	82.26556	2.45	3.0%
Ndufa11	65.57158	4.61	7.0%
Ndufa12	21.73488	1.24	5.7%
Ndufa12l	11.98394	0.71	5.9%
Ndufa13	31.82756	2.33	7.3%
Ndufa2	52.9833	2.01	3.8%
Ndufa3	85.49536	4.28	5.0%
Ndufa4	156.381	8.55	5.5%
Ndufa4l2	0.15005126	0.07	47.2%
Ndufa5	50.08788	1.95	3.9%
Ndufa6	93.87384	2.77	2.9%
Ndufa7	32.82628	2.75	8.4%

Ndufa8	65.90974	2.93	4.4%
Ndufa9	81.02156	2.22	2.7%
Ndufab1	3.588128	1.02	28.4%
Ndufaf1	7.44971	0.90	12.1%
Ndufb10	61.70214	3.20	5.2%
Ndufb11	42.96854	1.60	3.7%
Ndufb2	51.76146	1.53	2.9%
Ndufb3	30.8271	2.20	7.1%
Ndufb4	2.281318	0.55	24.2%
Ndufb5	85.22394	2.70	3.2%
Ndufb6	65.55538	4.01	6.1%
Ndufb7	41.52748	1.41	3.4%
Ndufb8	156.1882	8.82	5.6%
Ndufb9	141.6826	3.98	2.8%
Ndufc1	40.09994	2.50	6.2%
Ndufc2	77.50728	4.41	5.7%
Ndufs1	75.11738	2.10	2.8%
Ndufs2	105.5612	2.55	2.4%
Ndufs3	20.92352	1.33	6.3%
Ndufs4	45.84352	2.47	5.4%
Ndufs5	0.17573422	0.08	43.0%
Ndufs6	19.48718	1.21	6.2%
Ndufs7	70.10538	5.59	8.0%
Ndufs8	71.615	4.33	6.0%
Ndufv1	97.813	4.36	4.5%
Ndufv2	79.00862	2.33	2.9%
Neb	0.09368844	0.03	36.1%
Nebl	0.5766822	0.13	22.4%
Necap1	90.05032	2.36	2.6%
Necap2	5.487404	0.33	6.0%
Nedd1	0.8587284	0.15	17.0%
Nedd4	162.21	3.00	1.8%
Nedd4l	8.371718	0.26	3.1%
Nedd8	81.23308	4.29	5.3%
Nedd9	2.943322	0.30	10.2%
Nedl2	0.1192454	0.03	28.7%
Nefh	79.42608	5.37	6.8%
Nefl	765.079	50.10	6.5%
Nefm	10.044324	0.75	7.5%
Negr1	60.21512	1.96	3.3%
Neil1	3.590744	0.44	12.3%
Neil2	0.5111476	0.15	29.7%
Neil3	0.1723657	0.08	44.8%
Nek1	7.320258	0.41	5.5%
Nek10	0.02379408	0.02	93.9%
Nek11	0.2668314	0.07	28.0%
Nek2	0.4567338	0.07	15.8%

Nek3	0.8370718	0.11	13.6%
Nek4	4.452336	0.32	7.3%
Nek5	0.01731638	0.02	111.5%
Nek6	6.923824	0.34	4.9%
Nek7	4.28222	0.37	8.7%
Nek8	1.134292	0.16	14.4%
Nek9	7.427284	0.47	6.4%
Nelf	31.38024	1.15	3.7%
Nelfb	15.84614	0.33	2.1%
Nell1	40.92076	1.60	3.9%
Nell2	142.1062	7.22	5.1%
Nenf	25.85208	1.71	6.6%
Neo1	19.53408	0.97	4.9%
Nep	0.3608152	0.15	41.7%
nepas	0.03187082	0.01	41.6%
Nepn	0.7359022	0.18	23.9%
Nes	2.667816	0.26	9.8%
Net1	1.908612	0.22	11.3%
Neto1	11.45752	0.16	1.4%
Neto2	22.48286	1.52	6.7%
netrin-G1	1.35686644	1.18	86.9%
Neu1	17.2594	1.11	6.4%
Neu2	0.1509452	0.02	13.2%
Neu3	1.398908	0.11	8.2%
Neu4	0.01687196	0.01	54.1%
Neud4	6.933226	0.74	10.6%
Neurabin	0.8281172	0.32	38.9%
Neurl	9.164494	0.80	8.7%
Neurl2	0.8596628	0.07	8.6%
Neurod1	0.160769	0.01	8.0%
Neurod2	0.0130685	0.01	81.0%
Neurod4	0.12358474	0.07	53.4%
Neurod6	0.04404454	0.03	67.0%
Neurog1	0.0104615	0.01	95.6%
Neurog2	0.14923818	0.08	56.3%
Neurog3	0.00451716	0.01	223.6%
Nexn	1.830034	0.25	13.8%
Nf1	16.79456	0.77	4.6%
NF1	0.3960278	0.20	50.0%
NF1GRP	0.4203522	0.27	65.3%
Nf2	17.60486	1.15	6.5%
Nfam1	0.02987728	0.01	46.7%
Nfasc	19.6892	2.61	13.3%
Nfat5	5.135774	0.95	18.5%
Nfatc1	0.5943358	0.18	30.1%
Nfatc2	10.179046	0.49	4.9%
Nfatc2ip	1.199376	0.10	8.3%

Nfatc3	1.65949	0.17	10.4%
Nfatc4	0.7120664	0.20	28.8%
Nfe2	0.02790272	0.03	121.7%
Nfe2l1	95.86566	3.46	3.6%
Nfe2l2	3.737434	0.62	16.6%
Nfe2l3	0.5948666	0.14	22.7%
Nfia	2.57336	0.88	34.1%
Nfib	2.405422	0.32	13.3%
Nfic	12.42354	1.00	8.1%
Nfil3	3.582056	0.26	7.3%
Nfix	4.234336	0.29	6.9%
Nfkb1	5.421332	0.19	3.5%
Nfkb2	4.17865	1.70	40.8%
Nfkbia	3.86952	0.34	8.7%
Nfkbib	11.055328	1.21	10.9%
Nfkbie	0.7903164	0.07	9.3%
Nfkbil1	4.156628	0.48	11.5%
Nfkbil2	4.297344	0.16	3.7%
Nfkbiz	0.8791188	0.09	10.1%
Nfrkb	5.57422	0.28	5.1%
Nfs1	12.3182	0.70	5.7%
Nfu1	10.553644	0.85	8.1%
Nfx1	20.6862	0.77	3.7%
Nfxl1	3.608914	0.39	10.7%
Nfya	4.948	0.50	10.2%
Nfyb	7.971404	0.32	4.0%
Nfyc	4.377256	0.28	6.4%
Ng23	0.03569336	0.02	57.3%
Ngb	0.3333352	0.04	13.4%
Ngdn	10.189082	1.02	10.0%
Ngef	5.586066	0.19	3.4%
Ngfb	0.1493868	0.06	40.0%
Ngfr	1.51507	0.26	16.9%
Ngfrap1	244.172	18.81	7.7%
Ngly1	7.48428	1.83	24.4%
Ngp	0.00716354	0.02	223.6%
Ngrn	8.181774	1.00	12.2%
NHE8	0.02477086	0.02	61.8%
Nhedc1	0.02064498	0.00	18.5%
Nhedc2	0.03975356	0.03	75.5%
Nhej1	1.1591334	0.21	17.9%
Nhlh1	0.00354964	0.01	223.6%
Nhlh2	0.4233506	0.08	20.0%
Nhlrc1	3.056454	0.33	10.8%
Nhlrc2	2.031378	0.16	7.8%
Nhp2l1	19.10742	1.45	7.6%
Nhs	4.172266	0.35	8.5%

Nhsl1	1.760828	0.14	7.8%
ni-2	0.1857298	0.03	15.0%
Niban	1.028939	0.13	12.7%
Nicn1	48.65764	1.23	2.5%
Nid1	19.06	3.53	18.5%
Nid2	9.13677	1.72	18.8%
Nif3l1	8.620232	2.52	29.3%
Nin	1.246222	0.13	10.5%
Ninj1	8.506944	0.22	2.6%
Ninj2	0.00736474	0.02	223.6%
Nip7	7.095466	0.65	9.2%
Nipa1	27.7477	1.96	7.1%
Nipa2	14.39914	0.37	2.5%
Nipbl	9.624862	0.41	4.2%
Nipsnap1	32.64382	1.58	4.8%
Nipsnap3a	19.16168	2.50	13.0%
NIRF	0.5791416	0.14	23.8%
Nisch	107.6754	2.13	2.0%
Nit1	9.892372	0.28	2.9%
Nit2	8.26708	0.82	10.0%
Nkap	7.646814	0.29	3.8%
Nkapl	0.14089008	0.06	43.7%
Nkd1	1.4098748	0.34	24.3%
Nkd2	3.773182	0.17	4.5%
Nkg2c	0	0.00	
Nkg7	0	0.00	
Nkiras1	19.98544	0.44	2.2%
Nkiras2	17.95416	1.92	10.7%
Nkpd1	0.1805588	0.05	25.1%
Nkrf	27.80166	0.72	2.6%
Nktr	15.24198	1.59	10.5%
Nkx-1.2	0	0.00	
Nkx1-2	0	0.00	
Nkx2-2	0.4936972	0.11	21.5%
Nkx2-3	0.05642334	0.03	55.6%
Nkx2-4	0.00988946	0.01	138.6%
Nkx2-5	0.6398192	0.07	10.2%
Nkx2-6	0.0051275	0.01	223.6%
Nkx2-9	0.01282066	0.01	95.1%
Nkx3-1	0.1501386	0.03	18.0%
Nkx3-2	0.10868298	0.03	27.6%
Nkx6-1	0.03227444	0.02	60.4%
Nkx6-2	0.1741512	0.04	23.7%
Nkx6-3	0.05627642	0.02	36.3%
Nl1	0.06559552	0.06	86.9%
Nle1	3.873336	0.30	7.8%
Nlgn1	13.44976	0.41	3.0%

Nlgn2	69.38652	3.27	4.7%
Nlgn3	18.3061	0.39	2.1%
Nlk	11.01788	1.05	9.6%
Nln	13.183	2.34	17.7%
NLP-1	0.1784856	0.05	27.0%
Nlrc3	0.011788462	0.01	48.4%
Nlrc4	0.0063083	0.01	97.5%
Nlrp10	0.0148213	0.02	114.9%
Nlrp14	0.00297942	0.01	223.6%
Nlrp1a	0.00152064	0.00	223.6%
Nlrp1b	0	0.00	
Nlrp1c	0.001611556	0.00	223.6%
Nlrp2	0.0018906	0.00	223.6%
Nlrp3	0.010787126	0.01	73.7%
Nlrp4a	0.00618012	0.01	140.0%
Nlrp4b	0	0.00	
Nlrp4c	0.001260404	0.00	223.6%
Nlrp4e	0	0.00	
Nlrp4f	0.0710962	0.04	49.3%
Nlrp4g	0	0.00	
Nlrp5	0.04433346	0.01	18.2%
Nlrp6	0.07448654	0.02	26.6%
Nlrp9a	0.00244798	0.01	223.6%
Nlrp9b	0.09316278	0.04	43.7%
Nlrp9c	0.00283612	0.01	223.6%
Nlrx1	0.11569448	0.04	37.7%
Nm23-M7	0.29574534	0.37	123.5%
Nmb	0.4409536	0.06	13.6%
Nmbr	1.26173	0.16	12.6%
Nmd3	10.471116	0.56	5.3%
Nme1	83.44146	6.58	7.9%
Nme2	14.05784	1.10	7.8%
Nme3	11.19516	0.64	5.7%
Nme4	1.378764	0.29	21.1%
Nme5	1.0102132	0.18	18.0%
Nme6	1.355662	0.23	16.7%
Nme7	23.57636	4.57	19.4%
Nmi	0.3377754	0.09	25.7%
Nmnat1	1.221386	0.11	9.4%
Nmnat2	25.12158	0.27	1.1%
Nmnat3	0.09725494	0.06	60.1%
Nmral1	2.13397	0.26	12.2%
Nms	0.3398986	0.09	25.2%
Nmt1	28.17424	1.29	4.6%
Nmt2	21.97342	1.70	7.7%
Nmu	0.16356104	0.08	51.7%
Nmur1	0.12054478	0.05	40.3%

Nmur2	0.2864554	0.13	43.6%
Nna1	1.509096	0.14	9.2%
Nnat	194.0114	36.98	19.1%
Nnmt	0.04110714	0.03	79.9%
Nnt	3.938234	0.31	7.8%
Nob1	8.41169	0.67	8.0%
Nobox	0.01032568	0.01	94.7%
Noc3l	7.090914	0.24	3.4%
Noc4l	8.362166	0.53	6.4%
Nod1	0.3177396	0.06	17.8%
Nod2	0.0266334	0.01	38.1%
Nodal	0.11020256	0.03	23.9%
Nog	3.660024	0.27	7.5%
Nol1	9.222708	0.80	8.7%
Nol10	7.833174	0.61	7.8%
Nol11	10.108544	0.63	6.3%
Nol12	7.03669	0.57	8.1%
Nol14	11.12818	0.66	6.0%
Nol3	5.104066	0.30	5.9%
Nol4	5.351866	0.28	5.2%
Nol5	16.5329	0.84	5.1%
Nol5a	36.99226	2.15	5.8%
Nol6	25.73044	0.45	1.8%
Nol7	17.78272	0.64	3.6%
Nol8	4.782416	0.24	5.1%
Nol9	7.33464	0.46	6.3%
Nola1	5.100494	0.52	10.2%
Nola2	8.84409	1.42	16.1%
Nola3	58.30586	0.95	1.6%
Nolc1	15.06124	0.58	3.8%
Nomo1	31.08292	1.34	4.3%
Nono	26.3033	1.42	5.4%
Nope	9.623632	0.31	3.2%
Nos1	0.4862372	0.07	14.0%
Nos1ap	5.717028	0.59	10.3%
Nos2	0.002467444	0.00	137.4%
Nos3	0.226577	0.05	20.1%
Nosip	16.37474	0.53	3.3%
Nostrin	0.1886728	0.05	29.1%
Notch1	6.16146	2.52	40.8%
notch1	4.64642216	2.68	57.7%
Notch2	2.071784	0.23	10.9%
Notch3	1.250456	0.06	4.8%
Notch4	0.06331728	0.02	24.4%
Noto	0	0.00	
Notum	0.04889182	0.04	84.6%
Nov	0.9982532	0.05	5.4%

Nova1	91.71542	3.54	3.9%
Nova2	10.062782	0.79	7.9%
NOX1	0.04724874	0.06	117.6%
Nox1	0.01489222	0.02	144.7%
Nox3	0	0.00	
Nox4	0.05754812	0.02	29.0%
Noxa1	0	0.00	
Noxo1	0.8146636	0.12	15.2%
Npal1	0.026856028	0.03	110.3%
Npal2	2.787	0.09	3.2%
Npal3	30.15878	2.15	7.1%
Npas1	3.174996	0.91	28.7%
Npas2	0.7631738	0.18	24.0%
Npas3	7.124592	0.60	8.4%
Npas4	0.7797098	0.11	13.7%
Npat	3.213258	0.35	11.0%
Npb	0.03258228	0.05	147.7%
Npc1	7.254774	0.30	4.1%
Npc1l1	0.10901016	0.03	25.7%
Npc2	16.07968	0.84	5.2%
Npcd	56.74714	6.64	11.7%
Npdc1	97.40944	3.09	3.2%
Npepl1	0.400575	0.08	20.8%
Npepps	42.91636	1.04	2.4%
Npff	0.226381	0.08	35.6%
Npffr2	0	0.00	
Nphp1	2.212276	0.26	11.8%
Nphp3	2.046638	0.30	14.5%
Nphp4	1.675502	0.13	7.8%
Nphs1	0.0032662	0.00	141.0%
Nphs2	0.00201964	0.00	223.6%
Npl	1.0083644	0.20	19.8%
Nploc4	22.49982	1.18	5.2%
Npm1	27.52904	1.57	5.7%
Npm2	0.2099754	0.04	18.8%
Npm3	1.387374	0.25	18.1%
Npnt	11.343058	1.46	12.9%
Nppa	0.15265938	0.10	66.8%
Nppb	0	0.00	
Nppc	4.409132	0.46	10.4%
Npps2	0.416085	0.25	59.0%
Npr1	0.5209628	0.04	7.0%
Npr2	11.714588	1.89	16.1%
Npr3	0.726121	0.13	17.8%
Npsr1	0.02527302	0.02	64.8%
Nptn	249.4432	5.33	2.1%
Nptx1	17.44934	1.25	7.2%

Nptx2	18.4087	1.94	10.5%
Nptxr	13.63156	1.06	7.8%
Npvf	0.0301081	0.03	92.6%
Npw	0.01030488	0.02	223.6%
Npy	19.0311	1.95	10.3%
Npy1r	4.283164	0.33	7.8%
Npy2r	3.576892	0.40	11.2%
Npy5r	0.346022	0.10	30.2%
Npy6r	0.00359926	0.01	223.6%
Nqo1	1.28388	0.26	20.1%
Nqo2	2.44389	0.12	4.7%
NR_000003	0.0748136	0.17	223.6%
NR_000004	0	0.00	
NR_000040	0.164248	0.04	27.2%
NR_001277	0	0.00	
NR_001460	0.0244994	0.05	223.6%
NR_001461	0.1822086	0.06	35.0%
NR_001462	0.00456056	0.01	223.6%
NR_001463	0.008357286	0.00	34.7%
NR_001570	0.008183054	0.00	32.8%
NR_001579	0.0123257	0.03	223.6%
NR_001582	0.00507292	0.01	223.6%
NR_001583	0	0.00	
NR_001584	0.00359528	0.01	223.6%
NR_001585	0.01427944	0.01	93.9%
NR_001586	0	0.00	
NR_002142	0	0.00	
NR_002167	0.4974972	0.06	13.0%
NR_002172	0	0.00	
NR_002218	17.19206	1.97	11.5%
NR_002321	0.5718628	0.10	17.3%
NR_002322	3.86032	0.41	10.6%
NR_002452	0.001646544	0.00	223.6%
NR_002702	3.80786	0.68	17.7%
NR_002839	0.00521422	0.01	223.6%
NR_002841	0	0.00	
NR_002844	0.060612598	0.04	69.3%
NR_002846	0.08439586	0.03	41.0%
NR_002848	1.9111	0.38	20.1%
NR_002849	0	0.00	
NR_002851	0	0.00	
NR_002853	0.007602904	0.00	60.7%
NR_002854	0.011635106	0.01	128.5%
NR_002855	0.719637	0.10	13.6%
NR_002857	0	0.00	
NR_002859	0	0.00	
NR_002860	0.009787874	0.01	59.5%

NR_002862	5.853616	0.36	6.1%
NR_002863	0.1258304	0.02	15.5%
NR_002864	0.3018534	0.03	8.4%
NR_002865	0	0.00	
NR_002866	0.03432274	0.03	97.3%
NR_002867	0	0.00	
NR_002868	0	0.00	
NR_002869	0	0.00	
NR_002871	0.187172	0.07	38.4%
NR_002872	0	0.00	
NR_002873	0.2550788	0.10	40.5%
NR_002874	0.16912634	0.15	88.9%
NR_002883	2.839614	0.27	9.4%
NR_002886	38.5619	3.91	10.1%
NR_002888	1.0747424	0.09	7.9%
NR_002891	5.23099	0.51	9.7%
NR_002893	0	0.00	
NR_002895	0	0.00	
NR_002896	1.64403	0.15	8.9%
NR_002897	1.372548	0.46	33.2%
NR_002899	0.250571	0.15	58.1%
NR_002900	0	0.00	
NR_002901	0.1588054	0.22	138.2%
NR_002902	0.0495422	0.11	223.6%
NR_002903	0	0.00	
NR_002928	5.286082	0.38	7.2%
NR_003100	0	0.00	
NR_003107	0.05528156	0.05	83.6%
NR_003145	7.07271	0.75	10.6%
NR_003146	0	0.00	
NR_003202	0	0.00	
NR_003243	0.000653066	0.00	223.6%
NR_003247	0	0.00	
NR_003248	1.925834	0.22	11.3%
NR_003269	0	0.00	
NR_003270	7.77866	0.92	11.8%
NR_003282	0	0.00	
NR_003283	0	0.00	
NR_003292	0.4261364	0.05	11.9%
NR_003293	1.412176	0.14	9.7%
NR_003363	14.08728	1.52	10.8%
NR_003364	0.03380966	0.01	25.0%
NR_003368	0.43704	0.14	32.7%
NR_003372	0.02421322	0.03	104.5%
NR_003376	3.940572	0.29	7.3%
NR_003519	29.8623	3.04	10.2%
NR_003523	3.69932	0.70	18.9%

NR_003547	0	0.00	
NR_003557	24.02438	2.38	9.9%
NR_003596	0	0.00	
NR_003597	0.000594626	0.00	138.5%
NR_003619	0.4211012	0.03	8.1%
NR_003620	0.004221178	0.00	63.6%
NR_003625	0.013794878	0.01	81.0%
NR_003627	0.02154201	0.01	50.3%
NR_003628	0.00552744	0.01	223.6%
NR_003629	0	0.00	
NR_003630	0.001280634	0.00	111.6%
NR_003631	0.2644128	0.04	15.2%
NR_003632	0.08642446	0.03	40.5%
NR_003681	0	0.00	
NR_003702	0	0.00	
Nr0b1	0.09423504	0.03	27.1%
Nr0b2	0	0.00	
Nr1d1	9.33065	1.19	12.7%
Nr1d2	16.59532	1.83	11.0%
Nr1h2	11.390276	0.99	8.7%
Nr1h3	0.2044676	0.04	18.2%
Nr1h4	0	0.00	
Nr1h5	0	0.00	
Nr1i2	0.00709972	0.01	142.0%
Nr1i3	0.9774574	1.93	197.1%
Nr2c1	2.746768	0.26	9.5%
Nr2c2	9.884852	0.74	7.5%
Nr2e1	0.0796918	0.04	49.5%
Nr2e3	0	0.00	
Nr2f1	10.8871	0.81	7.4%
Nr2f2	3.5026	0.35	9.9%
Nr2f6	5.68635	0.23	4.0%
Nr3c1	5.82254	2.41	41.4%
Nr3c2	3.986436	0.49	12.4%
Nr4a1	1.99324	0.35	17.5%
Nr4a2	2.126712	0.40	18.8%
Nr4a3	0.3461484	0.08	23.3%
Nr5a1	0.008803028	0.01	119.0%
Nr5a2	0.1266175	0.04	32.3%
Nr6a1	1.22656	0.15	12.3%
NR8	0.001003992	0.00	223.6%
Nradd	0.7086768	0.13	18.9%
Nramp2	1.064929	0.39	36.8%
Nrap	0.09859582	0.02	21.3%
Nrarp	0.4008986	0.08	19.3%
Nras	10.9088	0.68	6.2%
Nrbf2	2.447654	0.21	8.5%

Nrbp1	32.44304	0.65	2.0%
Nrbp2	46.17996	2.11	4.6%
Nrcam	25.2369	1.68	6.7%
NrCAM	11.38154	0.71	6.2%
Nrd1	22.35794	1.71	7.6%
Nrf1	4.137152	0.39	9.4%
Nrg1	5.867148	0.63	10.8%
Nrg3	13.3232	0.54	4.0%
Nrg4	0.839963	1.52	181.5%
Nrgn	2.542686	0.76	29.7%
Nrip1	4.146452	0.27	6.6%
Nrip2	0.6551048	0.21	31.7%
Nrip3	30.26898	0.80	2.6%
Nrk	1.1654278	0.33	28.3%
NRK	0.05739668	0.02	27.6%
Nrl	0.0672057	0.04	63.2%
Nrm	1.3132892	0.30	22.8%
Nrn1	148.0402	10.86	7.3%
Nrn1l	0.7261026	0.31	42.5%
Nrp	0.10711328	0.06	51.4%
Nrp1	13.42908	0.97	7.3%
Nrp2	19.67918	3.08	15.7%
Nrsn1	45.76546	3.38	7.4%
Nrsn2	14.59776	1.43	9.8%
Nrtn	1.321836	0.33	25.2%
Nrxn1	44.7362	1.19	2.7%
Nrxn2	111.0678	6.68	6.0%
Nrxn3	21.71548	0.77	3.6%
nS7	4.83195	0.16	3.3%
Nsbp1	1.622118	0.22	13.4%
Nsd1	18.06698	3.63	20.1%
Nsddr	0.3991218	0.18	46.2%
Nsdhl	33.96758	4.16	12.2%
Nsf	204.9144	7.17	3.5%
Nsfl1c	17.90638	1.04	5.8%
Nsg1	255.3458	23.04	9.0%
Nsg2	181.9846	4.29	2.4%
Nsl1	0.3500726	0.09	25.8%
Nsmaf	11.14274	0.27	2.4%
Nsmce1	5.49947	0.44	8.0%
Nsmce2	12.21678	0.58	4.8%
Nsmce4a	9.910602	0.41	4.1%
Nspl1	0.3432544	0.09	25.2%
NSR1	1.3426262	0.24	18.1%
nssr_1	5.001086	1.63	32.5%
Nsun2	13.5574	0.79	5.8%
Nsun3	3.231928	0.50	15.6%

Nsun4	5.287564	0.31	5.9%
Nsun5	3.566322	0.24	6.8%
Nsun6	1.829186	0.08	4.5%
Nsun7	0.2660446	0.05	17.1%
Nt5c	16.07614	0.75	4.6%
Nt5c1a	0.1868548	0.07	35.7%
Nt5c1b	0	0.00	
Nt5c2	40.26196	2.21	5.5%
Nt5c3	22.03404	1.25	5.7%
Nt5c3l	13.85622	1.44	10.4%
Nt5dc1	0.6570586	0.17	25.6%
Nt5dc2	6.998442	2.05	29.3%
Nt5dc3	21.38952	1.00	4.7%
Nt5e	0.7840206	0.21	26.7%
Nt5m	17.30034	1.13	6.5%
Ntan1	21.43974	1.54	7.2%
Ntf3	1.312142	0.15	11.3%
Ntf5	0.07744342	0.05	63.2%
Nthl1	1.0525706	0.14	13.6%
Ntn1	4.488148	0.15	3.3%
Ntn2l	1.268898	0.44	34.8%
Ntn4	3.273432	0.31	9.3%
Ntng1	16.87992	0.80	4.7%
Ntng2	1.864458	0.34	18.0%
Ntrk2	43.6906	2.22	5.1%
Ntrk3	13.00558	0.94	7.2%
Nts	0.2539794	0.07	28.5%
Ntsr1	0.1958598	0.05	26.8%
Ntsr2	1.2705122	0.29	22.5%
Ntt4	0.3860326	0.09	22.3%
Nua	0.2261696	0.18	79.1%
Nuak1	7.054434	0.35	4.9%
Nuak2	0.5682416	0.07	12.9%
Nub1	29.00282	0.44	1.5%
Nubp1	5.957276	0.20	3.4%
Nubp2	11.3869	1.87	16.4%
Nubpl	1.548148	0.16	10.1%
Nucb1	37.63036	0.84	2.2%
Nucb2	8.739092	1.04	11.9%
Nucks1	31.13764	2.37	7.6%
Nudc	31.18356	1.86	6.0%
Nudcd1	8.71644	0.32	3.7%
Nudcd2	5.257542	0.55	10.5%
Nudcd3	42.91768	2.87	6.7%
Nudt1	3.974378	1.09	27.3%
Nudt10	8.370804	0.91	10.8%
Nudt11	16.12192	0.87	5.4%

Nudt12	1.0411142	0.10	9.2%
Nudt13	1.860204	0.03	1.7%
Nudt14	5.942976	0.38	6.3%
Nudt15	0.3322892	0.04	10.8%
Nudt16	2.933992	0.30	10.3%
Nudt16l1	16.4643	1.19	7.2%
Nudt17	1.1469746	0.24	21.0%
Nudt18	9.164074	0.11	1.2%
Nudt19	9.580276	0.61	6.4%
Nudt2	10.86796	0.68	6.3%
Nudt21	2.223094	0.25	11.0%
Nudt22	3.960516	0.36	9.2%
Nudt3	81.56018	3.42	4.2%
Nudt4	42.05698	1.76	4.2%
Nudt5	6.592908	0.37	5.6%
Nudt6	0.8242052	0.11	13.3%
Nudt7	1.383574	0.11	8.2%
Nudt8	4.299692	0.71	16.5%
Nudt9	10.089046	0.66	6.5%
Nuf2	0.755071	0.29	38.5%
Nufip1	4.13813	0.26	6.3%
Nufip2	11.30936	0.58	5.1%
Numa1	7.869486	0.87	11.0%
Numb	3.678412	0.47	12.8%
Numbl	28.809	1.66	5.8%
Nup107	4.849598	0.18	3.8%
Nup133	8.707616	0.23	2.7%
Nup153	12.65046	0.56	4.4%
Nup155	8.104944	0.56	6.9%
Nup160	3.05167	0.26	8.4%
Nup188	6.096736	0.13	2.1%
Nup205	3.380622	0.26	7.8%
Nup210	3.381524	0.14	4.0%
Nup214	5.575404	0.09	1.7%
Nup35	1.2396952	0.34	27.1%
Nup37	1.885858	0.21	11.3%
Nup43	3.600568	0.32	9.0%
Nup50	10.98684	0.39	3.5%
Nup54	4.79179	0.30	6.4%
Nup62	11.086102	1.10	10.0%
Nup85	8.937186	0.31	3.5%
Nup88	19.05756	0.61	3.2%
Nup93	9.762954	0.16	1.7%
Nup98	10.93784	0.67	6.1%
Nupl1	18.7987	0.77	4.1%
Nupl2	1.774278	0.20	11.4%
Nupr1	1.205748	0.10	8.2%

Nus1	21.89886	1.31	6.0%
Nusap1	1.284556	0.20	15.5%
Nut	0.02742024	0.01	30.1%
Nutf2	8.065578	0.23	2.8%
Nvl	10.741536	0.91	8.5%
Nwd1	3.37644	0.19	5.5%
Nxf1	16.34924	1.04	6.3%
Nxf2	0.0336543	0.03	84.2%
Nxf3	0.6829306	0.10	14.9%
Nxf7	0.38922	0.19	49.0%
Nxn	3.654984	0.45	12.2%
Nxnl1	0.02050046	0.02	90.3%
Nxnl2	0.19441434	0.14	70.9%
Nxph1	20.71704	1.00	4.8%
Nxph2	0.3389282	0.09	27.7%
Nxph3	0.23047	0.04	17.5%
Nxph4	1.36097	0.19	14.0%
Nxt1	2.62228	0.18	6.9%
Nxt2	11.43744	1.10	9.6%
Nyx	0.09221316	0.02	26.5%
O/E-4S	0.6110724	0.19	30.5%
Oaf	2.29142	0.34	15.0%
Oas1a	0.0196715	0.02	78.3%
Oas1b	0.20510032	0.08	40.0%
Oas1c	0.6564568	0.12	18.7%
Oas1d	0	0.00	
Oas1e	0.03086054	0.02	50.0%
Oas1f	0	0.00	
Oas1g	0.00850788	0.01	139.8%
Oas1h	0	0.00	
Oas2	0.009548604	0.01	129.6%
Oas3	0.006109704	0.00	72.6%
oasl1	0.06883734	0.03	47.1%
Oasl1	0.01675318	0.01	71.3%
oasl10	0.00093833	0.00	223.6%
oasl11	0.001062035	0.00	207.8%
Oasl2	0.3372664	0.07	20.3%
Oat	30.24216	0.66	2.2%
Oaz1	8.792516	0.61	7.0%
Oaz2	41.39284	1.12	2.7%
Oaz3	0.10493982	0.03	32.1%
Ob1	25.50836	2.92	11.5%
Obfc1	4.855262	0.54	11.2%
Obfc2a	2.213308	0.15	6.7%
Obfc2b	29.47928	3.55	12.0%
Obox1	0.00935724	0.01	137.7%
Obox3	0	0.00	

Obox5	0.01937904	0.01	58.3%
Obox6	0	0.00	
Obp1a	0	0.00	
Obscn	0.012270114	0.01	116.2%
Obsl1	4.054936	0.41	10.2%
Oc90	0.00939616	0.02	223.6%
Ocel1	4.152286	0.17	4.1%
Ociad1	184.7576	3.68	2.0%
Ociad2	8.42206	0.46	5.5%
Ocln	0.4107526	0.06	14.8%
Ocm	0.03041024	0.03	96.9%
Ocrl	21.38204	0.87	4.1%
Odam	0	0.00	
Odc1	2.999946	0.41	13.6%
Odf1	0.01269832	0.02	137.8%
Odf2	15.26274	0.60	4.0%
Odf2l	2.070372	0.22	10.7%
Odf3	0	0.00	
Odf3l1	0.09987786	0.02	19.9%
Odf4	0.05222204	0.01	23.8%
Odz1	2.888488	0.57	19.8%
Odz2	9.892058	0.38	3.8%
Odz3	9.421692	0.49	5.2%
Odz4	5.33077	0.46	8.7%
Ofcc1	0	0.00	
Ofd1	2.451894	0.14	5.7%
Ogdh	31.78668	0.61	1.9%
Ogdhl	30.25692	0.94	3.1%
Ogfod1	16.62468	3.13	18.8%
Ogfod2	9.08144	0.56	6.1%
Ogfr	18.87746	1.45	7.7%
Ogfrl1	10.436068	0.39	3.8%
Ogg1	2.100634	0.15	7.4%
Ogn	6.730606	1.30	19.3%
Ogt	49.55912	6.67	13.5%
Oip5	0.7815462	0.28	35.4%
Oit1	0.042054	0.02	58.3%
Oit3	0.00877806	0.01	97.4%
Ola1	34.37844	1.17	3.4%
Olah	0	0.00	
Olfm1	144.18	14.72	10.2%
Olfm2	43.69386	4.87	11.2%
Olfm3	21.7653	1.26	5.8%
Olfm4	0.0744592	0.07	95.8%
Olfml1	1.3106672	0.39	29.6%
Olfml2a	0.0571574	0.02	35.5%
Olfml2b	0.4474358	0.04	9.9%

Olfml3	2.294186	0.61	26.5%
Olfr1	0	0.00	
Olfr10	0	0.00	
Olfr100	0	0.00	
Olfr1000	0	0.00	
Olfr1002	0	0.00	
Olfr1006	0	0.00	
Olfr1008	0	0.00	
Olfr1009	0	0.00	
Olfr101	0	0.00	
Olfr1010	0	0.00	
Olfr1012	0	0.00	
Olfr1013	0	0.00	
Olfr1014	0	0.00	
Olfr1015	0	0.00	
Olfr1016	0	0.00	
Olfr1018	0	0.00	
Olfr1019	0	0.00	
Olfr1020	0	0.00	
Olfr1022	0	0.00	
Olfr1023	0	0.00	
Olfr1024	0	0.00	
Olfr1026	0	0.00	
Olfr1028	0	0.00	
Olfr1029	0	0.00	
Olfr103	0	0.00	
Olfr1030	0	0.00	
Olfr1031	0	0.00	
Olfr1032	0	0.00	
Olfr1033	0.05191004	0.02	39.1%
Olfr1034	0	0.00	
Olfr1036	0	0.00	
Olfr1037	0	0.00	
Olfr1038	0	0.00	
Olfr1039	0	0.00	
Olfr104	0	0.00	
Olfr1040	0	0.00	
Olfr1042	0	0.00	
Olfr1043	0	0.00	
Olfr1044	0	0.00	
Olfr1045	0	0.00	
Olfr1046	0	0.00	
Olfr1047	0	0.00	
Olfr1048	0	0.00	
Olfr1049	0	0.00	
Olfr105	0	0.00	
Olfr1051	0	0.00	

Olfr1052	0	0.00	
Olfr1054	0	0.00	
Olfr1055	0	0.00	
Olfr1056	0	0.00	
Olfr1057	0	0.00	
Olfr1058	0	0.00	
Olfr106	0	0.00	
Olfr1061	0	0.00	
Olfr1062	0	0.00	
Olfr1065	0	0.00	
Olfr1066	0	0.00	
Olfr107	0	0.00	
Olfr1076	0	0.00	
Olfr1079	0	0.00	
Olfr108	0	0.00	
Olfr1080	0	0.00	
Olfr1082	0	0.00	
Olfr1083	0	0.00	
Olfr1084	0.01304904	0.03	223.6%
Olfr1085	0	0.00	
Olfr1086	0	0.00	
Olfr1087	0	0.00	
Olfr1089	0	0.00	
Olfr109	0	0.00	
Olfr1090	0	0.00	
Olfr1093	0	0.00	
Olfr1094	0	0.00	
Olfr1095	0	0.00	
Olfr1097	0	0.00	
Olfr1098	0	0.00	
Olfr1099	0	0.00	
Olfr11	0	0.00	
Olfr110	0	0.00	
Olfr1100	0	0.00	
Olfr1101	0	0.00	
Olfr1102	0	0.00	
Olfr1104	0	0.00	
Olfr1105	0	0.00	
Olfr1106	0	0.00	
Olfr1107	0	0.00	
Olfr1109	0	0.00	
Olfr111	0	0.00	
Olfr1110	0	0.00	
Olfr1111	0	0.00	
Olfr1112	0	0.00	
Olfr1113	0	0.00	
Olfr1115	0	0.00	

Olfr1116	0	0.00	
Olfr1118	0	0.00	
Olfr112	0	0.00	
Olfr1120	0	0.00	
Olfr1121	0	0.00	
Olfr1122	0	0.00	
Olfr1123	0	0.00	
Olfr1125	0	0.00	
Olfr1126	0	0.00	
Olfr1128	0	0.00	
Olfr1129	0	0.00	
Olfr113	0	0.00	
Olfr1130	0	0.00	
Olfr1131	0	0.00	
Olfr1132	0	0.00	
Olfr1133	0	0.00	
Olfr1134	0	0.00	
Olfr1135	0	0.00	
Olfr1136	0	0.00	
Olfr1137	0	0.00	
Olfr1138	0	0.00	
Olfr114	0	0.00	
Olfr1140	0	0.00	
Olfr1141	0	0.00	
Olfr1143	0	0.00	
Olfr1145	0	0.00	
Olfr1148	0	0.00	
Olfr115	0	0.00	
Olfr1151	0	0.00	
Olfr1152	0	0.00	
Olfr1153	0	0.00	
Olfr1154	0	0.00	
Olfr1155	0	0.00	
Olfr1156	0	0.00	
Olfr1157	0	0.00	
Olfr1158	0	0.00	
Olfr116	0	0.00	
Olfr1160	0	0.00	
Olfr1161	0	0.00	
Olfr1162	0	0.00	
Olfr1163	0	0.00	
Olfr1164	0	0.00	
Olfr1165	0	0.00	
Olfr1166	0	0.00	
Olfr1167	0	0.00	
Olfr1168	0	0.00	
Olfr117	0	0.00	

Olfr1170	0	0.00	
Olfr1173	0	0.00	
Olfr1176	0	0.00	
Olfr1178	0	0.00	
Olfr1179	0	0.00	
Olfr118	0	0.00	
Olfr1180	0	0.00	
Olfr1181	0	0.00	
Olfr1182	0	0.00	
Olfr1183	0	0.00	
Olfr1184	0	0.00	
Olfr1186	0	0.00	
Olfr1188	0	0.00	
Olfr1189	0	0.00	
Olfr119	0	0.00	
Olfr1193	0	0.00	
Olfr1195	0	0.00	
Olfr1196	0	0.00	
Olfr1197	0	0.00	
Olfr1198	0.0128426	0.03	223.6%
Olfr1199	0	0.00	
Olfr12	0	0.00	
Olfr120	0	0.00	
Olfr1200	0	0.00	
Olfr1201	0	0.00	
Olfr1202	0	0.00	
Olfr1203	0	0.00	
Olfr1204	0	0.00	
Olfr1206	0	0.00	
Olfr1208	0	0.00	
Olfr1209	0	0.00	
Olfr121	0	0.00	
Olfr1211	0	0.00	
Olfr1212	0	0.00	
Olfr1213	0	0.00	
Olfr1214	0	0.00	
Olfr1215	0	0.00	
Olfr1216	0	0.00	
Olfr1217	0	0.00	
Olfr1218	0.01208698	0.03	223.6%
Olfr1219	0	0.00	
Olfr122	0	0.00	
Olfr1220	0	0.00	
Olfr1221	0	0.00	
Olfr1222	0	0.00	
Olfr1223	0	0.00	
Olfr1225	0	0.00	

Olfr1226	0	0.00	
Olfr1228	0	0.00	
Olfr1229	0	0.00	
Olfr123	0	0.00	
Olfr1230	0	0.00	
Olfr1231	0	0.00	
Olfr1232	0	0.00	
Olfr1233	0	0.00	
Olfr1234	0	0.00	
Olfr1238	0	0.00	
Olfr1239	0	0.00	
Olfr124	0	0.00	
Olfr1240	0	0.00	
Olfr1241	0	0.00	
Olfr1242	0	0.00	
Olfr1243	0	0.00	
Olfr1245	0	0.00	
Olfr1246	0	0.00	
Olfr1247	0	0.00	
Olfr1248	0	0.00	
Olfr1249	0	0.00	
Olfr125	0	0.00	
Olfr1250	0	0.00	
Olfr1251	0	0.00	
Olfr1252	0	0.00	
Olfr1254	0	0.00	
Olfr1255	0	0.00	
Olfr1256	0	0.00	
Olfr1257	0	0.00	
Olfr1258	0	0.00	
Olfr1259	0	0.00	
Olfr126	0	0.00	
Olfr1260	0	0.00	
Olfr1261	0	0.00	
Olfr1262	0	0.00	
Olfr1263	0	0.00	
Olfr1264	0	0.00	
Olfr1265	0	0.00	
Olfr1269	0	0.00	
Olfr127	0	0.00	
Olfr1270	0	0.00	
Olfr1271	0	0.00	
Olfr1272	0	0.00	
Olfr1273	0	0.00	
Olfr1274	0	0.00	
Olfr1275	0	0.00	
Olfr1276	0	0.00	

Olfr1277	0	0.00	
Olfr1278	0	0.00	
Olfr1279	0	0.00	
Olfr128	0	0.00	
Olfr1280	0	0.00	
Olfr1281	0	0.00	
Olfr1282	0	0.00	
Olfr1283	0	0.00	
Olfr1284	0	0.00	
Olfr1286	0	0.00	
Olfr1287	0	0.00	
Olfr1288	0	0.00	
Olfr1289	0	0.00	
Olfr129	0	0.00	
Olfr1290	0	0.00	
Olfr1294	0	0.00	
Olfr1295	0	0.00	
Olfr1297	0	0.00	
Olfr1298	0	0.00	
Olfr1299	0	0.00	
Olfr13	0	0.00	
Olfr130	0	0.00	
Olfr1300-ps1	0	0.00	
Olfr1301	0	0.00	
Olfr1302	0	0.00	
Olfr1303	0	0.00	
Olfr1305	0.01249114	0.03	223.6%
Olfr1306	0	0.00	
Olfr1307	0	0.00	
Olfr1308	0	0.00	
Olfr1309	0.00765672	0.02	223.6%
Olfr131	0.01706236	0.04	223.6%
Olfr1310	0	0.00	
Olfr1311	0	0.00	
Olfr1312	0	0.00	
Olfr1313	0	0.00	
Olfr1314	0.03358884	0.03	93.8%
Olfr1316	0	0.00	
Olfr1317	0	0.00	
Olfr1318	0.00844104	0.02	223.6%
Olfr132	0	0.00	
Olfr1320	0	0.00	
Olfr1321	0	0.00	
Olfr1322	0	0.00	
Olfr1323	0	0.00	
Olfr1324	0	0.00	
Olfr1325	0	0.00	

Olfr1328	0	0.00	
Olfr1329	0	0.00	
Olfr133	0	0.00	
Olfr1330	0	0.00	
Olfr1331	0	0.00	
Olfr1333	0	0.00	
Olfr1335	0	0.00	
Olfr1336	0	0.00	
Olfr1337	0	0.00	
Olfr1338	0	0.00	
Olfr1339	0	0.00	
Olfr134	0	0.00	
Olfr1340	0	0.00	
Olfr1341	0	0.00	
Olfr1342	0	0.00	
Olfr1344	0.698644	0.17	24.1%
Olfr1346	0	0.00	
Olfr1347	0	0.00	
Olfr1348	0	0.00	
Olfr1349	0	0.00	
Olfr135	0	0.00	
Olfr1350	0.00841052	0.02	223.6%
Olfr1351	0	0.00	
Olfr1352	0	0.00	
Olfr1353	0	0.00	
Olfr1355	0	0.00	
Olfr1356	0	0.00	
Olfr1357	0	0.00	
Olfr1359	0.00949002	0.02	223.6%
Olfr136	0	0.00	
Olfr1360	0	0.00	
Olfr1361	0	0.00	
Olfr1362	0	0.00	
Olfr1364	0	0.00	
Olfr1365	0	0.00	
Olfr1366	0	0.00	
Olfr1367	0	0.00	
Olfr1368	0	0.00	
Olfr137	0	0.00	
Olfr1370	0	0.00	
Olfr1371	0.01298338	0.03	223.6%
Olfr1372-ps1	0.07039114	0.03	45.6%
Olfr1373	0	0.00	
Olfr1377	0	0.00	
Olfr1378	0	0.00	
Olfr138	0	0.00	
Olfr1380	0	0.00	

Olfr1381	0	0.00	
Olfr1382	0	0.00	
Olfr1383	0	0.00	
Olfr1384	0	0.00	
Olfr1385	0	0.00	
Olfr1386	0	0.00	
Olfr1387	0	0.00	
Olfr1388	0	0.00	
Olfr1389	0.00648112	0.01	223.6%
Olfr139	0	0.00	
Olfr1390	0	0.00	
Olfr1391	0	0.00	
Olfr1392	0.00679402	0.02	223.6%
Olfr1393	0.0087289	0.02	223.6%
Olfr1394	0	0.00	
Olfr1395	0	0.00	
Olfr1396	0	0.00	
Olfr140	0	0.00	
Olfr1402	0	0.00	
Olfr1403	0	0.00	
Olfr1404	0	0.00	
Olfr1406	0	0.00	
Olfr1408	0	0.00	
Olfr141	0.00897148	0.02	223.6%
Olfr1410	0	0.00	
Olfr1411	0	0.00	
Olfr1412	0	0.00	
Olfr1413	0	0.00	
Olfr1414	0.00619424	0.01	223.6%
Olfr1415	0	0.00	
Olfr1416	0	0.00	
Olfr1417	0.11980478	0.05	39.0%
Olfr1418	0	0.00	
Olfr1419	0.01318826	0.02	137.2%
Olfr142	0	0.00	
Olfr1420	0.01513468	0.03	223.6%
Olfr1423	0	0.00	
Olfr1424	0	0.00	
Olfr1425	0	0.00	
Olfr1426	0	0.00	
Olfr1427	0	0.00	
Olfr1428	0	0.00	
Olfr143	0	0.00	
Olfr1431	0	0.00	
Olfr1434	0	0.00	
Olfr1436	0	0.00	
Olfr1437	0	0.00	

Olfr144	0	0.00	
Olfr1440	0	0.00	
Olfr1441	0	0.00	
Olfr1442	0.01099144	0.02	223.6%
Olfr1443	0	0.00	
Olfr1444	0	0.00	
Olfr1445	0	0.00	
Olfr1446	0	0.00	
Olfr1447	0.00891396	0.02	223.6%
Olfr1448	0	0.00	
Olfr1449	0	0.00	
Olfr145	0	0.00	
Olfr1450	0	0.00	
Olfr1451	0	0.00	
Olfr1453	0	0.00	
Olfr1454	0	0.00	
Olfr1457	0	0.00	
Olfr1459	0	0.00	
Olfr146	0	0.00	
Olfr1461	0	0.00	
Olfr1462	0	0.00	
Olfr1463	0	0.00	
Olfr1465	0	0.00	
Olfr1466	0	0.00	
Olfr1467	0	0.00	
Olfr1469	0	0.00	
Olfr147	0.00846608	0.02	223.6%
Olfr1471	0	0.00	
Olfr1472	0	0.00	
Olfr1474	0	0.00	
Olfr1475	0	0.00	
Olfr1477	0	0.00	
Olfr148	0.00851548	0.02	223.6%
Olfr1480	0	0.00	
Olfr1484	0	0.00	
Olfr1487	0	0.00	
Olfr1489	0	0.00	
Olfr149	0	0.00	
Olfr1490	0	0.00	
Olfr1491	0	0.00	
Olfr1494	0	0.00	
Olfr1495	0	0.00	
Olfr1496	0	0.00	
Olfr1497	0	0.00	
Olfr1499	0	0.00	
Olfr15	0	0.00	
Olfr1500	0	0.00	

Olfr1501	0	0.00	
Olfr1502	0	0.00	
Olfr1504	0	0.00	
Olfr1505	0	0.00	
Olfr1507	0	0.00	
Olfr1508	0	0.00	
Olfr1509	0	0.00	
Olfr151	0	0.00	
Olfr1510	0	0.00	
Olfr1511	0	0.00	
Olfr1512	0	0.00	
Olfr1513	0	0.00	
Olfr1514	0	0.00	
Olfr1518	0	0.00	
Olfr152	0	0.00	
Olfr153	0.0072187	0.02	223.6%
Olfr154	0	0.00	
Olfr155	0	0.00	
Olfr156	0	0.00	
Olfr157	0	0.00	
Olfr159	0.00834438	0.02	223.6%
Olfr16	0	0.00	
Olfr160	0	0.00	
Olfr161	0	0.00	
Olfr164	0	0.00	
Olfr165	0	0.00	
Olfr166	0	0.00	
Olfr167	0	0.00	
Olfr168	0	0.00	
Olfr169	0	0.00	
Olfr17	0	0.00	
Olfr170	0	0.00	
Olfr171	0	0.00	
Olfr172	0	0.00	
Olfr173	0	0.00	
Olfr174	0	0.00	
Olfr177	0	0.00	
Olfr178	0	0.00	
Olfr18	0.03841274	0.03	68.9%
Olfr180	0	0.00	
Olfr181	0	0.00	
Olfr183	0	0.00	
Olfr186	0	0.00	
Olfr187	0	0.00	
Olfr19	0	0.00	
Olfr190	0	0.00	
Olfr191	0	0.00	

Olfr192	0	0.00	
Olfr193	0	0.00	
Olfr194	0	0.00	
Olfr195	0	0.00	
Olfr196	0	0.00	
Olfr197	0	0.00	
Olfr198	0	0.00	
Olfr199	0	0.00	
Olfr2	0	0.00	
Olfr201	0	0.00	
Olfr202	0	0.00	
Olfr203	0	0.00	
Olfr204	0	0.00	
Olfr205	0	0.00	
Olfr206	0	0.00	
Olfr209	0	0.00	
Olfr211	0	0.00	
Olfr212	0.03334294	0.03	101.1%
Olfr213	0	0.00	
Olfr214	0.01522282	0.02	137.3%
Olfr215	0	0.00	
Olfr216	0	0.00	
Olfr218	0	0.00	
Olfr222	0	0.00	
Olfr223	0	0.00	
Olfr225	0	0.00	
Olfr228	0	0.00	
Olfr23	0	0.00	
Olfr232	0	0.00	
Olfr234	0	0.00	
Olfr239	0.00785052	0.02	223.6%
Olfr24	0	0.00	
Olfr244	0	0.00	
Olfr247	0	0.00	
Olfr25	0	0.00	
Olfr250	0	0.00	
Olfr251	0	0.00	
Olfr257	0	0.00	
Olfr259	0	0.00	
Olfr26	0	0.00	
Olfr262	0	0.00	
Olfr266	0.01131586	0.03	223.6%
Olfr267	0.00848176	0.02	223.6%
Olfr27	0	0.00	
Olfr270	0	0.00	
Olfr272	0	0.00	
Olfr273	0	0.00	

Olfr275	0	0.00	
Olfr281	0	0.00	
Olfr282	0	0.00	
Olfr283	0	0.00	
Olfr284	0	0.00	
Olfr288	0.07690562	0.03	38.3%
Olfr290	0.00916102	0.02	223.6%
Olfr291	0.00771228	0.02	223.6%
Olfr292	0	0.00	
Olfr293	0	0.00	
Olfr294	0	0.00	
Olfr295	0	0.00	
Olfr297	0	0.00	
Olfr298	0	0.00	
Olfr299	0	0.00	
Olfr3	0.00819758	0.02	223.6%
Olfr30	0	0.00	
Olfr301	0.00953832	0.02	223.6%
Olfr303	0	0.00	
Olfr304	0	0.00	
Olfr305	0	0.00	
Olfr307	0	0.00	
Olfr308	0	0.00	
Olfr309	0	0.00	
Olfr31	1.0545692	0.28	26.7%
Olfr310	0	0.00	
Olfr311	0	0.00	
Olfr312	0.00951402	0.02	223.6%
Olfr313	0	0.00	
Olfr314	0	0.00	
Olfr315	0	0.00	
Olfr316	0.00925486	0.02	223.6%
Olfr317	0	0.00	
Olfr318	0	0.00	
Olfr319	0	0.00	
Olfr32	0	0.00	
Olfr320	0	0.00	
Olfr323	0	0.00	
Olfr324	0	0.00	
Olfr325	0	0.00	
Olfr328	0	0.00	
Olfr329	0	0.00	
Olfr33	0	0.00	
Olfr330	0.05867272	0.05	82.9%
Olfr331	0	0.00	
Olfr332	0	0.00	
Olfr335	0	0.00	

Olfr338	0	0.00	
Olfr339	0	0.00	
Olfr340	0	0.00	
Olfr341	0	0.00	
Olfr342	0	0.00	
Olfr344	0	0.00	
Olfr345	0	0.00	
Olfr346	0	0.00	
Olfr347	0	0.00	
Olfr348	0	0.00	
Olfr350	0	0.00	
Olfr351	0	0.00	
Olfr352	0	0.00	
Olfr353	0	0.00	
Olfr354	0	0.00	
Olfr355	0	0.00	
Olfr356	0	0.00	
Olfr357	0	0.00	
Olfr358	0	0.00	
Olfr360	0	0.00	
Olfr361	0	0.00	
Olfr362	0	0.00	
Olfr365	0	0.00	
Olfr366	0	0.00	
Olfr367	0	0.00	
Olfr368	0	0.00	
Olfr370	0	0.00	
Olfr371	0	0.00	
Olfr372	0	0.00	
Olfr373	0	0.00	
Olfr374	0	0.00	
Olfr376	0	0.00	
Olfr378	0	0.00	
Olfr38	0	0.00	
Olfr380	0	0.00	
Olfr381	0	0.00	
Olfr382	0	0.00	
Olfr385	0	0.00	
Olfr386	0	0.00	
Olfr389	0	0.00	
Olfr39	0	0.00	
Olfr390	0	0.00	
Olfr392	0	0.00	
Olfr393	0	0.00	
Olfr394	0	0.00	
Olfr395	0	0.00	
Olfr397	0	0.00	

Olfr398	0	0.00	
Olfr399	0	0.00	
Olfr401	0	0.00	
Olfr402	0	0.00	
Olfr403	0	0.00	
Olfr406	0	0.00	
Olfr410	0	0.00	
Olfr411	0	0.00	
Olfr412	0	0.00	
Olfr414	0	0.00	
Olfr415	0	0.00	
Olfr417	0	0.00	
Olfr419	0	0.00	
Olfr42	0	0.00	
Olfr420	0	0.00	
Olfr421	0.0115479	0.03	223.6%
Olfr424	0	0.00	
Olfr427	0	0.00	
Olfr429	0	0.00	
Olfr43	0	0.00	
Olfr430	0	0.00	
Olfr432	0.00803762	0.02	223.6%
Olfr433	0	0.00	
Olfr434	0	0.00	
Olfr435	0	0.00	
Olfr437	0	0.00	
Olfr438	0	0.00	
Olfr44	0	0.00	
Olfr441	0	0.00	
Olfr444	0	0.00	
Olfr446	0	0.00	
Olfr447	0	0.00	
Olfr448	0	0.00	
Olfr449	0	0.00	
Olfr45	0	0.00	
Olfr450	0	0.00	
Olfr452	0	0.00	
Olfr453	0	0.00	
Olfr456	0.01306784	0.02	137.2%
Olfr457	0	0.00	
Olfr458	0	0.00	
Olfr459	0	0.00	
Olfr46	0	0.00	
Olfr460	0.00854522	0.02	223.6%
Olfr461	0.11764934	0.10	89.0%
Olfr462	0	0.00	
Olfr463	0.00725174	0.02	223.6%

Olfr464	0.0208881	0.03	150.7%
Olfr466	0	0.00	
Olfr467	0	0.00	
Olfr469	0	0.00	
Olfr47	0	0.00	
Olfr470	0	0.00	
Olfr472	0	0.00	
Olfr473	0.01156854	0.03	223.6%
Olfr474	0	0.00	
Olfr476	0	0.00	
Olfr477	0	0.00	
Olfr478	0	0.00	
Olfr479	0	0.00	
Olfr48	0	0.00	
Olfr480	0	0.00	
Olfr481	0	0.00	
Olfr482	0	0.00	
Olfr483	0	0.00	
Olfr484	0	0.00	
Olfr485	0	0.00	
Olfr486	0	0.00	
Olfr487	0	0.00	
Olfr488	0	0.00	
Olfr49	0	0.00	
Olfr490	0	0.00	
Olfr491	0	0.00	
Olfr492	0	0.00	
Olfr493	0	0.00	
Olfr494	0	0.00	
Olfr495	0	0.00	
Olfr497	0	0.00	
Olfr498	0	0.00	
Olfr5	0	0.00	
Olfr50	0	0.00	
Olfr502	0	0.00	
Olfr503	0	0.00	
Olfr504	0	0.00	
Olfr506	0	0.00	
Olfr507	0	0.00	
Olfr508	0	0.00	
Olfr509	0	0.00	
Olfr51	0	0.00	
Olfr510	0	0.00	
Olfr512	0	0.00	
Olfr513	0	0.00	
Olfr514	0	0.00	
Olfr516	0	0.00	

Olfr517	0	0.00	
Olfr518	0	0.00	
Olfr519	0	0.00	
Olfr52	0	0.00	
Olfr520	0	0.00	
Olfr521	0	0.00	
Olfr522	0	0.00	
Olfr523	0	0.00	
Olfr524	0	0.00	
Olfr525	0	0.00	
Olfr527	0	0.00	
Olfr53	0	0.00	
Olfr530	0	0.00	
Olfr531	0	0.00	
Olfr532	0	0.00	
Olfr533	0	0.00	
Olfr535	0	0.00	
Olfr536	0.006788002	0.01	93.8%
Olfr538	0	0.00	
Olfr539	0	0.00	
Olfr54	0	0.00	
Olfr541	0	0.00	
Olfr543	0.01510262	0.02	138.7%
Olfr544	0	0.00	
Olfr545	0.00896114	0.02	223.6%
Olfr547	0	0.00	
Olfr549	0	0.00	
Olfr55	0	0.00	
Olfr550	0	0.00	
Olfr551	0	0.00	
Olfr552	0	0.00	
Olfr553	0	0.00	
Olfr554	0	0.00	
Olfr555	0	0.00	
Olfr556	0	0.00	
Olfr557	0	0.00	
Olfr558	0	0.00	
Olfr559	0	0.00	
Olfr56	0.0044474	0.01	223.6%
Olfr560	0	0.00	
Olfr561	0	0.00	
Olfr564	0	0.00	
Olfr566	0	0.00	
Olfr568	0	0.00	
Olfr569	0	0.00	
Olfr57	0	0.00	
Olfr570	0	0.00	

Olfr571	0	0.00	
Olfr572	0	0.00	
Olfr574	0	0.00	
Olfr575	0	0.00	
Olfr576	0	0.00	
Olfr577	0	0.00	
Olfr578	0	0.00	
Olfr58	0.00736842	0.02	223.6%
Olfr582	0	0.00	
Olfr583	0	0.00	
Olfr584	0	0.00	
Olfr585	0	0.00	
Olfr586	0	0.00	
Olfr589	0	0.00	
Olfr59	0	0.00	
Olfr591	0	0.00	
Olfr592	0	0.00	
Olfr593	0	0.00	
Olfr594	0	0.00	
Olfr597	0	0.00	
Olfr598	0	0.00	
Olfr599	0	0.00	
Olfr6	0	0.00	
Olfr60	0	0.00	
Olfr600	0	0.00	
Olfr601	0	0.00	
Olfr604	0	0.00	
Olfr605	0	0.00	
Olfr606	0	0.00	
Olfr608	0	0.00	
Olfr609	0	0.00	
Olfr61	0	0.00	
Olfr610	0	0.00	
Olfr611	0	0.00	
Olfr614	0	0.00	
Olfr615	0	0.00	
Olfr616	0	0.00	
Olfr617	0	0.00	
Olfr618	0.00982582	0.02	223.6%
Olfr619	0	0.00	
Olfr62	0	0.00	
Olfr620	0	0.00	
Olfr622	0	0.00	
Olfr623	0	0.00	
Olfr624	0	0.00	
Olfr627	0	0.00	
Olfr628	0	0.00	

Olfr629	0	0.00	
Olfr63	0	0.00	
Olfr630	0	0.00	
Olfr631	0	0.00	
Olfr632	0	0.00	
Olfr633	0.00665428	0.01	223.6%
Olfr635	0	0.00	
Olfr638	0	0.00	
Olfr639	0	0.00	
Olfr64	0	0.00	
Olfr640	0	0.00	
Olfr641	0	0.00	
Olfr642	0	0.00	
Olfr643	0	0.00	
Olfr644	0	0.00	
Olfr645	0	0.00	
Olfr646	0	0.00	
Olfr648	0	0.00	
Olfr649	0	0.00	
Olfr65	0	0.00	
Olfr651	0	0.00	
Olfr652	0	0.00	
Olfr653	0	0.00	
Olfr654	0	0.00	
Olfr655	0	0.00	
Olfr656	0	0.00	
Olfr657	0	0.00	
Olfr658	0.00308126	0.01	223.6%
Olfr659	0	0.00	
Olfr66	0	0.00	
Olfr661	0	0.00	
Olfr663	0	0.00	
Olfr665	0	0.00	
Olfr666	0	0.00	
Olfr667	0	0.00	
Olfr668	0.01115622	0.02	223.6%
Olfr669	0	0.00	
Olfr67	0	0.00	
Olfr670	0	0.00	
Olfr671	0	0.00	
Olfr672	0	0.00	
Olfr675	0	0.00	
Olfr676	0	0.00	
Olfr677	0	0.00	
Olfr678	0	0.00	
Olfr679	0	0.00	
Olfr68	0	0.00	

Olfr681	0	0.00	
Olfr683	0	0.00	
Olfr684	0	0.00	
Olfr685	0	0.00	
Olfr686	0	0.00	
Olfr688	0	0.00	
Olfr689	0.01065284	0.02	223.6%
Olfr69	0	0.00	
Olfr690	0.06981494	0.06	85.3%
Olfr691	0.03273982	0.05	140.5%
Olfr692	0.14063052	0.12	83.4%
Olfr693	0	0.00	
Olfr694	0	0.00	
Olfr695	0	0.00	
Olfr697	0	0.00	
Olfr698	0	0.00	
Olfr699	0	0.00	
Olfr700	0	0.00	
Olfr701	0	0.00	
Olfr702	0	0.00	
Olfr703	0	0.00	
Olfr704	0	0.00	
Olfr705	0	0.00	
Olfr706	0	0.00	
Olfr707	0	0.00	
Olfr708	0	0.00	
Olfr71	0	0.00	
Olfr710	0	0.00	
Olfr711	0	0.00	
Olfr713	0	0.00	
Olfr714	0	0.00	
Olfr715	0	0.00	
Olfr716	0	0.00	
Olfr720	0	0.00	
Olfr722	0	0.00	
Olfr723	0	0.00	
Olfr724	0	0.00	
Olfr725	0	0.00	
Olfr726	0	0.00	
Olfr727	0	0.00	
Olfr728	0	0.00	
Olfr729	0	0.00	
Olfr73	0	0.00	
Olfr730	0	0.00	
Olfr731	0	0.00	
Olfr732	0	0.00	
Olfr733	0	0.00	

Olfr734	0	0.00	
Olfr735	0	0.00	
Olfr736	0	0.00	
Olfr738	0	0.00	
Olfr739	0	0.00	
Olfr74	0	0.00	
Olfr740	0	0.00	
Olfr741	0	0.00	
Olfr742	0	0.00	
Olfr744	0	0.00	
Olfr745	0	0.00	
Olfr746	0	0.00	
Olfr747	0	0.00	
Olfr748	0	0.00	
Olfr749	0	0.00	
Olfr750	0	0.00	
Olfr76	0.00954246	0.02	223.6%
Olfr761	0	0.00	
Olfr763	0	0.00	
Olfr765	0	0.00	
Olfr767	0	0.00	
Olfr768	0	0.00	
Olfr769	0	0.00	
Olfr77	0	0.00	
Olfr770	0	0.00	
Olfr771	0	0.00	
Olfr772	0	0.00	
Olfr773-ps1	0	0.00	
Olfr774	0	0.00	
Olfr776	0	0.00	
Olfr777	0	0.00	
Olfr78	0.00798072	0.01	91.7%
Olfr780	0	0.00	
Olfr781	0	0.00	
Olfr782	0	0.00	
Olfr784	0	0.00	
Olfr786	0	0.00	
Olfr787	0.01157498	0.03	223.6%
Olfr788	0	0.00	
Olfr790	0	0.00	
Olfr791	0	0.00	
Olfr792	0	0.00	
Olfr794	0	0.00	
Olfr796	0	0.00	
Olfr798	0	0.00	
Olfr799	0	0.00	
Olfr8	0	0.00	

Olfr800	0	0.00	
Olfr801	0	0.00	
Olfr802	0	0.00	
Olfr803	0	0.00	
Olfr804	0	0.00	
Olfr805	0	0.00	
Olfr806	0	0.00	
Olfr807	0	0.00	
Olfr808	0	0.00	
Olfr809	0	0.00	
Olfr810	0	0.00	
Olfr811	0	0.00	
Olfr812	0	0.00	
Olfr813	0	0.00	
Olfr814	0	0.00	
Olfr815	0	0.00	
Olfr816	0	0.00	
Olfr818	0	0.00	
Olfr820	0	0.00	
Olfr821	0	0.00	
Olfr822	0	0.00	
Olfr823	0	0.00	
Olfr824	0	0.00	
Olfr825	0	0.00	
Olfr826	0	0.00	
Olfr827	0	0.00	
Olfr828	0	0.00	
Olfr829	0	0.00	
Olfr830	0	0.00	
Olfr832	0	0.00	
Olfr834	0	0.00	
Olfr835	0	0.00	
Olfr836	0	0.00	
Olfr837	0	0.00	
Olfr843	0	0.00	
Olfr845	0	0.00	
Olfr846	0	0.00	
Olfr847	0	0.00	
Olfr849	0	0.00	
Olfr850	0	0.00	
Olfr851	0	0.00	
Olfr853	0	0.00	
Olfr854	0	0.00	
Olfr855	0	0.00	
Olfr857	0.0102241	0.02	223.6%
Olfr859	0.0090394	0.02	223.6%
Olfr860	0	0.00	

Olfr862	0.00864116	0.02	223.6%
Olfr866	0	0.00	
Olfr867	0	0.00	
Olfr868	0	0.00	
Olfr869	0	0.00	
Olfr870	0	0.00	
Olfr871	0	0.00	
Olfr872	0	0.00	
Olfr873	0	0.00	
Olfr874	0.0085395	0.02	223.6%
Olfr875	0	0.00	
Olfr876	0	0.00	
Olfr877	0	0.00	
Olfr878	0	0.00	
Olfr881	0	0.00	
Olfr883	0	0.00	
Olfr884	0	0.00	
Olfr885	0	0.00	
Olfr887	0	0.00	
Olfr888	0	0.00	
Olfr889	0	0.00	
Olfr890	0	0.00	
Olfr891	0	0.00	
Olfr893	0	0.00	
Olfr894	0	0.00	
Olfr895	0	0.00	
Olfr898	0	0.00	
Olfr9	0.00991006	0.02	223.6%
Olfr90	0.00603348	0.01	223.6%
Olfr901	0	0.00	
Olfr902	0	0.00	
Olfr904	0	0.00	
Olfr905	0	0.00	
Olfr906	0	0.00	
Olfr907	0	0.00	
Olfr908	0	0.00	
Olfr909	0	0.00	
Olfr91	0	0.00	
Olfr910	0	0.00	
Olfr912	0	0.00	
Olfr913	0	0.00	
Olfr914	0	0.00	
Olfr915	0	0.00	
Olfr916	0	0.00	
Olfr917	0	0.00	
Olfr918	0	0.00	
Olfr919	0	0.00	

Olfr92	0	0.00	
Olfr920	0.05920446	0.08	128.0%
Olfr921	0	0.00	
Olfr922	0	0.00	
Olfr923	0	0.00	
Olfr924	0	0.00	
Olfr926	0.00864466	0.02	223.6%
Olfr93	0	0.00	
Olfr930	0	0.00	
Olfr933	0	0.00	
Olfr934	0	0.00	
Olfr935	0	0.00	
Olfr937	0	0.00	
Olfr938	0	0.00	
Olfr94	0.02736178	0.03	112.5%
Olfr943	0	0.00	
Olfr944	0	0.00	
Olfr945	0	0.00	
Olfr948	0	0.00	
Olfr95	0	0.00	
Olfr951	0	0.00	
Olfr952	0	0.00	
Olfr954	0	0.00	
Olfr955	0	0.00	
Olfr957	0	0.00	
Olfr958	0	0.00	
Olfr959	0	0.00	
Olfr96	0	0.00	
Olfr960	0	0.00	
Olfr961	0	0.00	
Olfr963	0	0.00	
Olfr965	0	0.00	
Olfr967	0	0.00	
Olfr968	0	0.00	
Olfr969	0	0.00	
Olfr97	0	0.00	
Olfr970	0	0.00	
Olfr971	0	0.00	
Olfr972	0	0.00	
Olfr973	0	0.00	
Olfr974	0	0.00	
Olfr975	0	0.00	
Olfr976	0	0.00	
Olfr978	0	0.00	
Olfr979	0	0.00	
Olfr98	0	0.00	
Olfr980	0	0.00	

Olfr981	0	0.00	
Olfr982	0	0.00	
Olfr983	0	0.00	
Olfr984	0	0.00	
Olfr985	0	0.00	
Olfr986	0	0.00	
Olfr987	0	0.00	
Olfr988	0	0.00	
Olfr99	0	0.00	
Olfr992	0	0.00	
Olfr993	0	0.00	
Olfr994	0	0.00	
Olfr995	0	0.00	
Olfr996	0	0.00	
Olfr998	0	0.00	
Olig1	39.23698	5.54	14.1%
Olig2	9.044424	1.06	11.8%
Olig3	0.2646404	0.07	27.1%
Olr1	0.01865392	0.03	151.7%
Oma1	1.80896	0.21	11.6%
Omd	0.03795018	0.04	118.1%
Omg	35.42784	3.53	10.0%
Omp	0.05376042	0.03	49.1%
Omt2b	0	0.00	
Onecut1	0.6410818	0.17	26.9%
Onecut2	0.4867004	0.05	11.0%
Onecut3	0.3986066	0.06	16.1%
Oog1	0	0.00	
Oog2	0	0.00	
Oog3	0	0.00	
Oog4	0	0.00	
Oosp1	0	0.00	
Opa1	33.36716	1.68	5.0%
Opa3	15.3277	1.41	9.2%
Opcml	25.10126	0.84	3.3%
Ophn1	9.88491	0.67	6.8%
Oplah	0.7794984	0.05	6.5%
Opn1mw	0.08920682	0.04	39.4%
Opn1sw	0	0.00	
Opn3	5.52417	0.46	8.4%
Opn4	0.029248	0.01	17.4%
Opn5	0	0.00	
oppo_1	0.04501856	0.01	27.0%
Oprd1	0.9108476	0.13	14.5%
Oprk1	0.5481206	0.22	40.6%
Oprl1	8.592422	1.69	19.7%
Oprm	0.010715288	0.02	157.0%

Oprm1	2.775726	0.87	31.2%
Oprs1	29.6739	4.04	13.6%
Optc	0.590794	0.14	24.4%
Optn	8.886704	0.41	4.7%
Orai1	2.683944	0.27	10.2%
Orai2	11.07448	0.35	3.2%
Orai3	2.305922	0.20	8.9%
Oraov1	6.012144	0.29	4.8%
Orc1l	0.169724	0.02	10.5%
Orc2l	8.036302	2.97	37.0%
Orc3l	15.28064	0.81	5.3%
Orc4l	16.97532	1.00	5.9%
Orc5l	14.26006	0.90	6.3%
Orc6	0.3704924	0.23	61.5%
Orc6l	10.243418	0.90	8.8%
ORF19	7.903666	0.43	5.4%
ORF28	1.994692	0.42	21.0%
ORF3	1.536272	0.44	28.6%
ORF34	10.220854	0.62	6.1%
ORF37	0.0918074	0.03	29.5%
ORF61	33.74524	1.05	3.1%
ORF63	0.00519594	0.01	223.6%
ORF9	0.10482766	0.09	87.1%
Orm1	0.00817554	0.02	223.6%
Orm2	0.0350537	0.02	58.1%
Orm3	0.01439982	0.03	223.6%
Ormdl1	4.085984	0.58	14.2%
Ormdl2	0.7956782	0.20	24.6%
Ormdl3	15.53942	0.54	3.5%
Orp1	0.4850541	0.50	102.8%
Os9	40.49588	1.59	3.9%
Osbp	29.45846	1.11	3.8%
Osbp2	13.31736	1.49	11.2%
Osbpl10	5.8167	0.35	6.1%
Osbpl11	3.691952	0.25	6.8%
Osbpl1a	22.56432	0.75	3.3%
Osbpl2	24.52062	1.39	5.7%
Osbpl3	1.516942	0.21	13.8%
Osbpl5	8.686558	0.31	3.6%
Osbpl6	13.07388	0.31	2.3%
Osbpl7	4.75702	0.25	5.2%
Osbpl8	10.300558	0.63	6.1%
Osbpl9	38.24118	2.20	5.7%
Oscar	0.11223414	0.04	33.2%
OSCP1	0.12102538	0.03	28.7%
Osf2/Cbfa1	0.1726982	0.04	25.5%
Osgep	1.482014	0.13	8.5%

Osgepl1	6.204	0.76	12.3%
Osgin1	0.05671408	0.04	70.0%
Osgin2	5.83477	0.19	3.2%
Osm	0	0.00	
Osmr	0.8755908	0.18	20.7%
Osr1	12.4387	0.92	7.4%
Osr2	0.7936374	0.21	27.0%
Osta	0.01267934	0.01	92.7%
Ostb	0	0.00	
Ostf1	6.05118	0.41	6.8%
Ostm1	18.00266	0.79	4.4%
Ostn	0.04820018	0.05	110.8%
Otc	0	0.00	
Otoa	0.06128332	0.03	54.4%
Otof	0.020886126	0.01	44.6%
Otog	0.000784452	0.00	223.6%
Otop1	0.10652198	0.05	48.5%
Otop2	0.02279922	0.02	91.4%
Otop3	0.01075436	0.01	59.4%
Otor	0.0207222	0.05	223.6%
Otos	0.01554902	0.02	138.6%
Otp	0.8215004	0.17	21.2%
Ott	0	0.00	
OTTMUSG00000000231	0	0.00	
OTTMUSG00000000281	0	0.00	
OTTMUSG00000000421	1.519408	0.18	11.5%
OTTMUSG00000000480	0	0.00	
OTTMUSG00000000494	0	0.00	
OTTMUSG00000000514	0	0.00	
OTTMUSG00000000541	0	0.00	
OTTMUSG00000000651	0	0.00	
OTTMUSG00000000712	0.00995358	0.01	139.2%
OTTMUSG00000000720	0.00384904	0.01	223.6%
OTTMUSG00000000934	0.07880852	0.03	37.5%
OTTMUSG00000000971	0.01590782	0.04	223.6%
OTTMUSG00000000990	0	0.00	
OTTMUSG00000000997	0.0096669	0.01	140.5%
OTTMUSG00000001246	0	0.00	
OTTMUSG00000002038	0.0218687	0.03	151.4%
OTTMUSG00000002177	0	0.00	
OTTMUSG00000002180	0	0.00	
OTTMUSG00000002196	0	0.00	
OTTMUSG00000004461	8.229142	0.82	9.9%
OTTMUSG00000004966	0	0.00	
OTTMUSG00000005065	0.0390718	0.02	48.8%
OTTMUSG00000005148	0.0675679	0.04	59.5%
OTTMUSG00000005491	0.00532464	0.01	138.5%

OTTMUSG00000005523	0.001794236	0.00	223.6%
OTTMUSG00000005737	0.00643494	0.01	223.6%
OTTMUSG00000007485	0	0.00	
OTTMUSG00000007655	0	0.00	
OTTMUSG00000008540	0	0.00	
OTTMUSG00000008561	0.43404	0.12	26.8%
OTTMUSG00000008911	0	0.00	
OTTMUSG00000010009	0	0.00	
OTTMUSG00000010105	0.799842	0.12	15.3%
OTTMUSG00000010173	0.00575598	0.01	137.3%
OTTMUSG00000010207	0	0.00	
OTTMUSG00000010328	0	0.00	
OTTMUSG00000010432	0	0.00	
OTTMUSG00000010433	0	0.00	
OTTMUSG00000010537	0	0.00	
OTTMUSG00000010657	0.2287236	0.03	14.3%
OTTMUSG00000010671	0.04907168	0.05	108.4%
OTTMUSG00000010673	0.4403338	0.06	13.1%
OTTMUSG00000010694	0.0592254	0.03	55.7%
OTTMUSG00000010965	0.06041698	0.02	34.3%
OTTMUSG00000011070	0.13214106	0.04	31.9%
OTTMUSG00000011268	0	0.00	
OTTMUSG00000011269	0	0.00	
OTTMUSG00000011272	0	0.00	
OTTMUSG00000011275	0	0.00	
OTTMUSG00000012686	0.311327	0.09	29.3%
OTTMUSG00000013918	0.02319638	0.03	111.3%
OTTMUSG00000014964	4.720418	0.57	12.1%
OTTMUSG00000015529	0.0029153	0.01	223.6%
OTTMUSG00000015643	0.00812198	0.02	223.6%
OTTMUSG00000015730	0.8427628	0.21	24.6%
OTTMUSG00000015743	0.5248954	0.19	36.2%
OTTMUSG00000015750	17.32532	0.29	1.7%
OTTMUSG00000015762	0.3033724	0.06	19.7%
OTTMUSG00000015859	0	0.00	
OTTMUSG00000015915	0.00393682	0.01	223.6%
OTTMUSG00000016293	0	0.00	
OTTMUSG00000016406	0	0.00	
OTTMUSG00000016407	0	0.00	
OTTMUSG00000016411	0	0.00	
OTTMUSG00000016420	0	0.00	
OTTMUSG00000016437	0	0.00	
OTTMUSG00000016453	0	0.00	
OTTMUSG00000016571	2.10683	0.13	6.3%
OTTMUSG00000016703	0	0.00	
OTTMUSG00000016790	0	0.00	
OTTMUSG00000017155	0	0.00	

OTTMUSG00000017156	0	0.00	
OTTMUSG00000017866	0	0.00	
OTTMUSG00000019138	0	0.00	
OTTMUSG00000020946	0	0.00	
OTTMUSP00000006088	0.2399672	0.08	32.0%
Otub1	179.8294	11.00	6.1%
Otub2	3.875046	0.13	3.5%
Otud4	7.37928	0.38	5.2%
Otud5	15.83354	0.25	1.6%
Otud6b	11.73314	0.41	3.5%
Otud7a	6.245934	0.17	2.8%
Otud7b	1.960376	0.21	11.0%
Otx1	0.00967092	0.01	91.9%
Otx2	0.04598858	0.04	93.6%
Ovca2	0.9273828	0.08	8.8%
Ovch2	0.00611218	0.01	137.6%
Ovgp1	1.6827192	0.63	37.6%
Ovol1	0.08560758	0.03	33.2%
Ovol2	0.05016234	0.02	39.4%
Oxa1l	22.5448	1.13	5.0%
Oxct1	192.1646	9.14	4.8%
Oxct2a	0	0.00	
Oxct2b	0.02830264	0.03	110.0%
Oxgr1	0.00235958	0.01	223.6%
Oxnad1	5.308488	0.24	4.6%
Oxr1	26.69282	2.55	9.6%
Oxsm	4.62747	1.16	25.0%
Oxsr1	8.043018	0.41	5.1%
Oxt	0.06304074	0.04	68.4%
Oxtr	0.3129566	0.03	9.8%
p	0.2904376	0.05	18.8%
p130Cas	0.3036208	0.07	23.4%
P140	40.80074	1.32	3.2%
P2P-R	0.9821318	0.35	35.8%
P2rx1	0.00571132	0.01	137.0%
P2rx2	0.06030338	0.03	42.1%
P2rx3	0.9559836	0.10	10.2%
P2rx4	3.835084	0.51	13.2%
P2rx5	0.887761	0.09	9.7%
P2rx7	0.07478684	0.02	25.6%
P2rxl1	0.7136144	0.01	1.1%
P2ry1	3.61597	0.10	2.8%
P2ry10	0.00462888	0.01	223.6%
P2ry12	0.00612238	0.01	223.6%
P2ry13	0	0.00	
P2ry14	0.04972292	0.05	99.4%
P2ry2	0.0910002	0.03	32.0%

P2ry4	0.01581352	0.02	100.6%
P2ry5	0.7445002	0.06	7.5%
P2ry6	0.01374398	0.02	116.9%
P42pop	14.20166	2.12	14.9%
P4ha1	9.548194	1.70	17.8%
P4ha2	7.94296	1.39	17.5%
P4ha3	1.29521	0.07	5.2%
P4hb	70.9532	4.00	5.6%
p53	0.1674052	0.07	41.3%
p62	0.3512766	0.17	48.0%
P7	0.18607716	0.14	73.1%
p73H	0.000669048	0.00	223.6%
pa	3.056214	0.93	30.4%
Pa2g4	17.73926	1.13	6.4%
Pabpc1	47.59164	3.53	7.4%
Pabpc2	0	0.00	
Pabpc4	3.562082	0.18	5.1%
Pabpc5	1.0883542	0.17	15.6%
Pabpn1	40.04522	3.57	8.9%
Pabpnl1	0.00797168	0.01	137.4%
Pacrg	6.723572	0.43	6.4%
Pacs1	40.0459	2.63	6.6%
Pacs2	26.1762	0.62	2.4%
Pacsin1	54.8153	5.01	9.1%
Pacsin2	10.145054	0.38	3.7%
Pacsin3	1.548342	0.33	21.2%
Padi1	0	0.00	
Padi2	0.2508426	0.05	19.1%
Padi3	0.00748842	0.01	96.5%
Padi4	0	0.00	
Padi6	0	0.00	
Paf1	15.87272	0.80	5.0%
Pafah1b1	164.9274	2.96	1.8%
Pafah1b2	92.42458	3.57	3.9%
Pafah1b3	21.7937	2.70	12.4%
Pafah2	3.36764	0.26	7.8%
Pag1	4.181578	0.34	8.2%
Pah	0.0485038	0.03	60.5%
Paics	21.74242	0.81	3.7%
Paip1	21.9576	0.82	3.7%
Paip2	115.1646	3.37	2.9%
Paip2b	24.91364	1.00	4.0%
Pak1	92.19654	3.36	3.6%
Pak1ip1	11.69328	2.30	19.7%
Pak2	8.907708	0.42	4.8%
Pak3	33.8744	3.79	11.2%
Pak4	1.212872	0.11	9.1%

Pak6	6.32378	0.55	8.7%
Pak7	2.46316	0.35	14.2%
Paka	1.4431482	0.60	41.4%
Palb2	0.3103464	0.06	19.0%
Palld	1.360528	0.10	7.1%
Palm	41.65088	3.04	7.3%
Palm2	6.309846	0.59	9.4%
Palmd	0.1815222	0.02	10.1%
Pals1	0.463238	0.08	17.7%
Pam	23.10958	2.06	8.9%
Pamci	0.00529788	0.01	137.4%
Pan3	7.620298	0.18	2.4%
Pank1	11.61752	0.66	5.7%
Pank2	9.04396	0.33	3.6%
Pank3	27.53698	1.19	4.3%
Pank4	8.791322	0.58	6.6%
Panx1	13.81014	2.04	14.8%
Panx2	39.6257	2.79	7.0%
Panx3	0	0.00	
Paox	1.209378	0.12	10.1%
Pap	0	0.00	
Papd1	10.79846	0.20	1.8%
Papd4	1.947588	0.15	7.6%
Papd5	9.05624	0.39	4.3%
PAPK-A	10.575894	1.06	10.0%
Papln	0.011332218	0.01	64.9%
Papola	22.93538	0.79	3.4%
Papolb	0.349275	0.03	9.4%
Papolg	6.6605	0.35	5.2%
Pappa	0.5575828	0.11	19.3%
Pappa2	0.16159756	0.17	105.0%
Papss1	26.26652	2.40	9.1%
Papss2	0.599771	0.12	19.9%
Paqr11	1.3107142	0.36	27.1%
Paqr3	8.838216	1.00	11.3%
Paqr4	16.3183	0.91	5.6%
Paqr5	0.0205976	0.02	73.4%
Paqr6	0.03745366	0.03	77.4%
Paqr7	11.25144	0.65	5.8%
Paqr8	14.49432	0.87	6.0%
Paqr9	10.57386	0.44	4.1%
Par3	0.630475	0.45	70.6%
Parc	4.63036	0.24	5.1%
Pard3	5.967582	0.21	3.5%
Pard3b	1.17891	0.12	10.1%
Pard6a	13.95952	0.79	5.6%
Pard6b	1.510396	0.26	17.2%

Pard6g	4.289408	0.45	10.6%
Parg	10.07937	0.26	2.5%
Park2	4.23652	0.43	10.0%
Park7	67.2289	4.07	6.1%
Parl	8.17536	0.71	8.7%
Parn	11.40696	0.28	2.5%
Parp1	15.75544	0.16	1.0%
Parp11	12.44272	1.54	12.4%
Parp12	1.612532	0.11	7.0%
Parp14	0.06716366	0.02	22.8%
Parp16	1.539188	0.16	10.6%
Parp2	5.58173	0.38	6.9%
Parp3	0.335423	0.03	9.1%
Parp4	0.3998484	0.07	17.8%
Parp6	42.3534	3.00	7.1%
Parp8	9.965396	0.42	4.2%
Parp9	0.3224998	0.09	28.8%
Pars2	2.474478	0.12	4.9%
Parva	23.30874	1.02	4.4%
Parvb	0.87511	0.09	10.4%
Parvg	0.0144225	0.01	75.1%
Pask	0.6425106	0.08	13.0%
PAT4	3.155264	1.33	42.0%
Patz1	8.06965	0.53	6.5%
Pawr	0.8778968	0.08	9.4%
Pax1	0.6687334	0.12	18.3%
Pax2	7.306668	0.28	3.9%
Pax3	0.4296958	0.10	23.3%
Pax4	0.03881164	0.04	93.9%
Pax5	0.2041036	0.05	25.4%
Pax6	1.0117684	0.21	20.3%
Pax7	0.006101302	0.01	97.5%
Pax8	1.63045	0.09	5.3%
Pax9	0.1718938	0.03	20.0%
Paxip1	7.004978	0.42	6.0%
Pb1	10.61545	0.97	9.1%
Pbef1	25.1437	0.71	2.8%
Pbk	0.79781	0.26	32.0%
Pbld	0.03266398	0.02	71.9%
Pbp2	0.0435349	0.04	82.3%
Pbrm1	13.70046	1.24	9.0%
Pbsn	0	0.00	
Pbx1	21.2086	1.33	6.3%
PBX1a	0.04471788	0.03	67.1%
Pbx2	13.8685	0.95	6.8%
Pbx3	56.30298	6.11	10.9%
PBX3	0.05109588	0.04	77.1%

Pbx4	5.700192	0.38	6.7%
Pbxip1	3.494806	0.32	9.1%
Pcaf	1.952754	0.14	7.2%
Pcbd1	0.9438948	0.28	29.9%
Pcbd2	4.506486	0.53	11.8%
Pcbp1	57.20816	5.55	9.7%
Pcbp2	95.3726	5.75	6.0%
Pcbp3	22.40488	1.12	5.0%
Pcbp4	42.25562	2.74	6.5%
Pcca	10.44494	0.80	7.7%
Pccb	19.53958	0.66	3.4%
Pcdh1	18.32994	0.78	4.3%
Pcdh10	26.26274	1.42	5.4%
Pcdh11x	10.409458	0.58	5.6%
Pcdh12	0.0437581	0.02	43.0%
Pcdh15	1.88879	0.19	10.2%
Pcdh17	17.70384	1.25	7.0%
Pcdh18	5.197954	0.37	7.2%
Pcdh19	7.176522	0.83	11.6%
Pcdh20	2.236122	0.24	10.8%
Pcdh21	0.5490102	0.12	21.3%
Pcdh7	27.76064	1.37	4.9%
Pcdh8	13.56662	1.12	8.3%
Pcdh9	23.88472	0.93	3.9%
Pcdha1	6.63034	1.85	28.0%
Pcdha10	1.2347206	0.29	23.2%
Pcdha11	2.74841	2.22	80.9%
Pcdha12	3.299932	0.54	16.4%
Pcdha2	4.082304	0.43	10.5%
Pcdha3	8.782036	0.96	10.9%
Pcdha4	7.92817	2.12	26.7%
Pcdha5	3.770834	0.33	8.7%
Pcdha6	0.2710006	0.10	35.4%
Pcdha7	0.3630884	0.13	36.2%
Pcdha8	0.0629176	0.02	26.6%
Pcdha9	2.128578	0.45	21.1%
Pcdhac1	0.2521512	0.09	34.3%
Pcdhac2	16.05558	0.74	4.6%
Pcdhb1	0.3337658	0.08	25.3%
Pcdhb10	3.286606	0.29	8.8%
Pcdhb11	4.500502	0.35	7.7%
Pcdhb12	0.7790816	0.13	16.6%
Pcdhb13	1.167968	0.13	10.8%
Pcdhb14	2.002424	0.15	7.4%
Pcdhb15	0.5159198	0.10	18.8%
Pcdhb16	2.036968	0.16	8.0%
Pcdhb17	2.664198	0.23	8.5%

Pcdhb18	1.653396	0.13	8.0%
Pcdhb19	2.46435	0.25	10.0%
Pcdhb2	5.535988	0.63	11.4%
Pcdhb20	3.709402	0.14	3.8%
Pcdhb21	0.8353146	0.11	13.2%
Pcdhb3	4.896458	0.35	7.1%
Pcdhb4	0.3455082	0.06	18.6%
Pcdhb5	2.716722	0.18	6.6%
Pcdhb6	3.965778	0.37	9.2%
Pcdhb7	4.954098	0.34	6.9%
Pcdhb8	2.175992	0.30	13.6%
Pcdhb9	2.701288	0.32	11.9%
Pcdhga1	1.595454	0.13	8.1%
Pcdhga10	1.306854	0.12	9.2%
Pcdhga11	1.854894	0.16	8.8%
Pcdhga12	0.5954548	0.11	18.8%
Pcdhga2	4.345996	0.23	5.2%
Pcdhga3	5.209336	0.31	5.9%
Pcdhga4	2.32067	0.24	10.5%
Pcdhga5	3.61802	0.34	9.3%
Pcdhga6	1.623116	0.33	20.6%
Pcdhga7	4.503896	0.28	6.1%
Pcdhga8	1.2801802	0.28	21.8%
Pcdhga9	1.433326	0.16	11.5%
Pcdhgb1	0.5272804	0.03	5.0%
Pcdhgb2	0.5051554	0.10	20.6%
Pcdhgb4	2.10425	0.28	13.2%
Pcdhgb5	2.698868	0.21	7.9%
Pcdhgb6	2.079068	0.29	13.8%
Pcdhgb7	1.0577344	0.22	20.8%
Pcdhgb8	0.9220836	0.28	29.9%
Pcdhgc3	11.296778	1.03	9.1%
Pcdhgc4	17.59488	0.94	5.4%
Pcdhgc5	0.9426982	0.08	8.6%
PCDHX	17.29616	2.54	14.7%
Pcf11	7.852724	1.07	13.6%
Pcgf1	3.700634	0.76	20.6%
Pcgf2	11.40342	1.10	9.7%
Pcgf3	12.10396	1.35	11.1%
Pcgf5	2.324384	0.38	16.3%
Pcgf6	9.990638	0.50	5.0%
Pcid2	9.557864	0.43	4.5%
Pck1	0	0.00	
Pck2	5.164956	0.24	4.6%
Pclo	9.378232	0.79	8.4%
Pcm1	21.37368	1.52	7.1%
pcm-1	5.97584	0.73	12.2%

Pcmt1	81.54968	3.37	4.1%
Pcmtd1	20.69748	0.45	2.2%
Pcmtd2	13.05196	1.38	10.5%
Pcna	6.875686	0.82	12.0%
Pcnp	16.59222	0.56	3.4%
Pcnt	1.1129934	0.14	12.2%
PCNT2	0.0269832	0.01	18.9%
Pcnx	8.749894	0.41	4.7%
Pcnxl2	4.266194	0.26	6.1%
Pcnxl3	17.53786	0.94	5.4%
Pcolce	6.744094	1.60	23.7%
Pcolce2	0.612433	0.13	20.4%
Pcp2	3.3930766	3.18	93.8%
Pcp4	119.1548	13.75	11.5%
Pcp4l1	23.99974	2.89	12.0%
Pcqap	0.6865598	0.21	31.0%
Pcsk1	3.625024	0.40	11.0%
Pcsk1n	181.2214	29.12	16.1%
Pcsk2	65.59596	1.98	3.0%
Pcsk4	2.62591	0.22	8.3%
Pcsk5	6.565	0.41	6.2%
Pcsk6	2.368492	0.12	5.2%
Pcsk7	4.606582	0.70	15.3%
Pcsk9	0.012381734	0.01	81.1%
Pctk1	91.75878	2.25	2.4%
Pctk2	15.51602	0.75	4.8%
Pctk3	0.5423258	0.21	38.4%
Pctp	0.1646255	0.05	32.3%
Pcx	19.57944	2.30	11.8%
Pcyox1	39.39778	1.02	2.6%
Pcyox1l	12.92836	1.31	10.2%
Pcyt1a	12.14446	0.46	3.8%
Pcyt1b	18.88454	0.92	4.8%
Pcyt2	32.17044	1.44	4.5%
Pdap1	29.06844	2.50	8.6%
Pdc	0	0.00	
Pdcd1	0	0.00	
Pdcd10	8.510392	0.40	4.7%
Pdcd11	6.79809	0.31	4.6%
Pdcd1lg2	0.00413134	0.01	223.6%
Pdcd2	7.385908	0.55	7.4%
Pdcd2l	7.817534	0.82	10.4%
Pdcd4	44.32152	3.77	8.5%
Pdcd5	0.449516	0.12	26.8%
Pdcd6	22.31106	1.03	4.6%
Pdcd6ip	20.75364	1.19	5.7%
Pdcd7	8.48648	0.58	6.9%

Pdcl	8.62866	0.31	3.6%
Pdcl2	0	0.00	
Pdcl3	16.4834	0.99	6.0%
Pddc1	15.8905	0.65	4.1%
Pde10a	11.86212	0.74	6.3%
Pde11a	0.1800108	0.06	31.0%
Pde1a	1.0658316	0.09	8.8%
Pde1b	24.82196	1.80	7.3%
Pde1c	1.31512	0.18	13.6%
Pde2a	3.126208	0.45	14.5%
Pde3a	3.162728	0.22	6.8%
Pde3b	2.335754	0.18	7.9%
Pde4a	13.2226	1.71	13.0%
Pde4b	16.73538	0.85	5.1%
Pde4c	0.01390544	0.01	64.6%
Pde4d	13.66448	0.49	3.6%
Pde4dip	19.98632	0.98	4.9%
Pde5a	0.9795288	0.22	22.6%
Pde6a	0.04100336	0.02	53.9%
Pde6b	0.0104551	0.01	137.0%
Pde6c	0.00209528	0.00	223.6%
Pde6d	24.63552	1.48	6.0%
Pde6g	0.1827674	0.03	17.1%
Pde6h	0.1271532	0.02	15.8%
Pde7a	5.219504	0.49	9.3%
Pde7b	0.6169592	0.15	24.7%
Pde8a	0.5097706	0.06	11.5%
Pde8b	8.996566	0.75	8.3%
Pde9a	2.031354	0.29	14.3%
Pdgfa	15.1619	0.47	3.1%
Pdgfb	4.215982	0.47	11.1%
Pdgfc	1.279796	0.15	11.5%
Pdgfd	0.2163778	0.04	20.4%
Pdgfra	8.022148	1.34	16.7%
Pdgfrb	3.529026	0.52	14.6%
Pdgfrl	1.537714	0.28	18.3%
Pdha1	91.53872	3.63	4.0%
Pdha2	0.10453304	0.04	39.7%
Pdhb	37.89004	1.16	3.1%
Pdhx	25.7604	1.38	5.3%
Pdia2	0.01139768	0.01	94.6%
Pdia3	93.924	4.62	4.9%
Pdia4	25.5739	1.56	6.1%
Pdia5	1.896064	0.29	15.2%
Pdia6	37.93386	1.65	4.4%
Pdik1l	6.470966	0.46	7.1%
Pdk1	10.147876	1.71	16.8%

Pdk2	14.0954	2.21	15.7%
Pdk3	7.78361	0.62	8.0%
Pdk4	0.2223936	0.04	18.6%
Pdlim1	0.8900232	0.19	21.2%
Pdlim2	0.6359272	0.19	30.5%
Pdlim3	0.2107497	0.11	53.5%
Pdlim4	1.0661626	0.16	15.4%
Pdlim5	5.08807	0.31	6.0%
Pdlim7	19.22788	1.71	8.9%
Pdpk1	18.2972	0.35	1.9%
Pdpn	6.28897	1.19	18.9%
Pdrg1	31.7768	1.16	3.6%
Pds5a	7.843834	0.59	7.5%
Pds5b	21.18524	0.72	3.4%
Pdss1	2.943992	0.23	7.7%
Pdss2	4.432872	0.42	9.5%
Pdx1	0.00933412	0.01	92.8%
Pdxdc1	25.92382	0.57	2.2%
Pdxk	35.19432	1.89	5.4%
Pdxp	71.00102	4.66	6.6%
Pdyn	0.3627074	0.06	15.9%
Pdzd11	9.357534	0.91	9.7%
Pdzd2	6.765812	0.69	10.1%
Pdzd3	0.0158059	0.01	90.9%
Pdzd4	20.5311	0.76	3.7%
Pdzd6	0.06256876	0.06	88.1%
Pdzd7	2.0863282	1.05	50.6%
Pdzd8	26.955	0.68	2.5%
Pdzk1	1.776	0.10	5.8%
Pdzk1ip1	0.02280632	0.01	59.6%
Pdzrn3	2.562542	0.26	10.3%
Pdzrn4	5.368876	0.51	9.4%
Pdzx	0	0.00	
PE1	0.408735	0.05	12.2%
Pea15a	93.1581	4.87	5.2%
Pea15b	0	0.00	
Pebp1	49.3268	2.62	5.3%
Pecam1	0.1685808	0.05	27.0%
Peci	11.22052	0.85	7.5%
Pecr	2.389742	0.21	8.6%
Pef1	23.3797	1.09	4.7%
Peg10	63.00664	3.32	5.3%
Peg12	0.540167	0.10	18.5%
Peg3	143.5834	11.98	8.3%
Peg5	1.887448	0.61	32.4%
Peli1	6.03074	0.42	6.9%
Peli2	4.463794	0.17	3.9%

Peli3	6.58544	0.48	7.3%
Pelo	5.699314	0.30	5.2%
Pelota	0.392619	0.27	69.5%
Pelp1	14.68632	1.53	10.4%
Pemt	2.233918	0.15	6.6%
Penk1	50.94656	4.69	9.2%
Peo1	5.444588	0.36	6.7%
Pepd	15.00842	0.98	6.5%
Per1	4.83118	0.58	12.0%
Per2	1.966994	0.22	11.3%
Per3	3.81863	0.41	10.8%
Perld1	2.133896	0.19	8.9%
Perp	3.266054	0.51	15.7%
Perq1	17.35484	1.57	9.1%
Pes1	6.650636	0.40	6.0%
Pet112l	2.513292	0.22	8.7%
Pet2	0	0.00	
Pex1	4.647928	1.30	27.9%
Pex10	3.881374	0.15	3.8%
Pex11a	1.554164	0.10	6.6%
Pex11b	14.80192	0.81	5.5%
Pex11c	0.22262678	0.13	57.0%
Pex11g	0.1638544	0.19	115.7%
Pex12	4.067904	0.17	4.3%
Pex13	4.8703	1.24	25.5%
Pex14	17.31994	0.67	3.9%
Pex16	7.495388	0.60	8.0%
Pex19	49.9375	2.50	5.0%
Pex2	10.680376	0.92	8.6%
Pex26	6.374894	0.57	8.9%
Pex3	7.538478	0.56	7.5%
Pex5	20.97662	0.81	3.9%
Pex6	12.65522	0.37	2.9%
Pex7	11.0968	0.44	3.9%
Pfas	2.839398	0.41	14.4%
Pfdn1	30.46608	1.64	5.4%
Pfdn2	36.29056	2.13	5.9%
Pfdn4	18.56704	1.51	8.1%
Pfdn5	30.18414	2.12	7.0%
Pfkfb1	0.4577898	0.12	26.7%
Pfkfb2	6.756178	0.50	7.4%
Pfkfb3	11.813738	2.12	17.9%
Pfkfb4	2.86128	0.19	6.7%
Pfkl	25.8053	3.66	14.2%
Pfkm	80.42926	2.69	3.4%
Pfkp	94.70442	4.02	4.2%
Pfn1	80.17346	4.82	6.0%

Pfn2	371.5392	12.39	3.3%
Pfn3	0.000382184	0.00	223.6%
Pfn4	3.781802	0.44	11.7%
Pfpl	0.0022803	0.01	223.6%
Pftk1	58.51806	3.29	5.6%
Pga5	2.99845	0.72	23.9%
Pgam1	21.30554	1.64	7.7%
Pgam2	1.799262	0.10	5.7%
Pgam5	25.06844	1.31	5.2%
Pgbd1	0.2757362	0.06	23.1%
Pgbd5	10.602556	0.55	5.1%
Pgc	3.391166	0.89	26.2%
PGC-1v	4.228704	0.71	16.8%
Pgcp	0.13962956	0.08	58.1%
Pgd	24.953	2.03	8.2%
Pgf	0.08695248	0.02	27.8%
Pggt1b	6.351188	0.41	6.4%
Pgk1	15.77962	1.10	7.0%
Pgk2	0	0.00	
Pgls	1.0282928	0.09	8.3%
Pglyrp1	0.15912214	0.06	40.4%
Pglyrp2	0	0.00	
Pglyrp3	0.0051069	0.01	223.6%
Pglyrp4	0	0.00	
Pgm1	2.99903	0.20	6.7%
Pgm2	18.09202	1.01	5.6%
Pgm2l1	47.99618	2.91	6.1%
Pgm3	4.967206	0.35	7.0%
Pgm5	2.226792	0.19	8.7%
Pgpep1	4.565736	0.33	7.2%
Pgr	5.028944	0.26	5.1%
Pgr15l	0.7923978	0.10	13.1%
Pgrmc1	180.3386	4.93	2.7%
Pgrmc2	21.65998	0.28	1.3%
Pgs1	21.84044	12.25	56.1%
Phactr1	23.88986	1.13	4.7%
Phactr2	2.637882	0.11	4.3%
Phactr3	6.291944	0.22	3.5%
Phactr4	1.505578	0.09	6.2%
Phb	10.705268	0.96	9.0%
Phb2	43.68	3.35	7.7%
Phc1	9.29286	0.37	4.0%
Phc2	9.682	0.56	5.8%
Phc3	4.730864	0.46	9.8%
Phca	5.941268	0.61	10.3%
Phemx	0.00263202	0.00	158.2%
Phex	0.05594514	0.02	30.1%

Phf1	6.084384	1.06	17.4%
Phf10	8.966056	2.40	26.7%
Phf11	0	0.00	
Phf12	9.268046	0.34	3.7%
Phf13	7.099498	0.25	3.5%
Phf14	7.278548	0.62	8.5%
Phf15	5.151232	0.34	6.5%
Phf16	0.737482	0.15	20.9%
Phf17	5.478576	0.42	7.7%
Phf19	0.3522718	0.05	13.0%
Phf2	7.403086	0.26	3.6%
Phf20	8.207762	0.37	4.5%
Phf20l1	13.53562	1.28	9.4%
Phf21a	6.802244	0.55	8.0%
Phf21b	0.7941738	0.13	16.2%
Phf23	12.1425	0.42	3.4%
Phf3	8.104614	0.28	3.5%
Phf5a	14.8129	1.78	12.0%
Phf6	9.109522	0.64	7.1%
Phf7	1.678164	0.12	7.3%
Phf8	6.197674	0.50	8.0%
Phgdh	3.337376	0.72	21.5%
Phip	8.662176	1.44	16.6%
Phka1	3.669664	0.27	7.5%
Phka2	4.071768	0.35	8.5%
Phkb	9.48656	0.38	4.0%
Phkg1	0.6875238	0.08	12.3%
Phkg2	21.7814	0.45	2.1%
Phlda1	5.311942	0.51	9.7%
Phlda2	0.02069908	0.02	95.7%
Phlda3	21.3933	0.92	4.3%
Phldb1	3.948176	0.27	6.9%
Phldb2	0.7855528	0.11	14.2%
Phldb3	0.04735906	0.04	79.6%
Phlpp	7.610326	0.19	2.4%
Phlppl	8.344658	0.88	10.5%
Phospho1	6.209658	1.15	18.6%
Phospho2	10.226682	0.33	3.2%
Phox2a	1.0333944	0.26	25.1%
Phox2b	0.04814382	0.03	59.3%
Phpt1	46.79796	3.15	6.7%
Phr1	27.99352	1.74	6.2%
Phtf1	5.031568	0.36	7.1%
Phtf2	7.799846	0.75	9.7%
Phxr1	0	0.00	
Phxr4	0	0.00	
Phyh	12.99708	0.52	4.0%

Phyhd1	1.27005	0.13	10.5%
Phyhip	17.14882	1.36	7.9%
Phyhipl	64.6304	4.63	7.2%
Pi15	0.8572968	0.27	31.6%
Pi16	0.2137364	0.05	24.7%
Pi4k2a	24.3188	0.57	2.3%
Pi4k2b	0.693234	0.13	19.1%
Pi4ka	31.2026	2.07	6.6%
Pi4kb	11.13322	0.39	3.5%
Pias1	14.87502	0.61	4.1%
Pias2	17.36578	0.64	3.7%
Pias3	4.790154	0.19	4.0%
Pias4	6.339194	0.16	2.5%
PIAS-gamma	0.3901166	0.10	26.5%
Pib5pa	10.87268	0.64	5.9%
Picalm	51.89904	1.80	3.5%
Pick1	22.62238	2.36	10.4%
PICOT	0.3335252	0.06	18.3%
Pif1	0.20231046	0.10	50.3%
Piga	1.923754	0.22	11.5%
Pigb	3.356292	0.32	9.6%
Pigc	3.947556	0.24	6.1%
Pigf	5.589148	0.40	7.1%
Pigg	5.101588	0.24	4.6%
Pigh	3.889394	0.09	2.4%
Pigk	17.27768	0.62	3.6%
Pigl	1.954896	0.18	9.4%
Pigm	2.999284	0.18	5.9%
Pign	2.652948	0.34	12.7%
Pig-n	0.05579322	0.03	46.8%
Pigo	8.15481	0.20	2.5%
Pigp	14.78398	2.67	18.1%
Pigq	39.68682	1.82	4.6%
Pigr	0.05864782	0.02	36.2%
Pigs	25.60362	1.62	6.3%
Pigt	53.52474	5.91	11.0%
Pigu	18.84736	1.56	8.3%
Pigv	3.647708	0.21	5.7%
Pigw	2.643198	0.32	12.3%
Pigx	5.647698	0.70	12.5%
Pigy	5.945018	0.15	2.5%
Pigyl	10.21325	0.69	6.8%
Pigz	1.310344	0.39	30.0%
Pih1d1	18.06812	3.65	20.2%
Pih1d2	0.2119202	0.08	39.9%
Pik3ap1	0.04186708	0.02	43.0%
Pik3c2a	3.33498	0.15	4.5%

Pik3c2b	1.42911	0.11	8.0%
Pik3c2g	0.042141994	0.03	78.1%
Pik3c3	9.116894	0.58	6.3%
Pik3ca	16.05738	1.21	7.5%
Pik3cb	8.573044	0.17	2.0%
Pik3cd	2.395678	0.22	9.2%
Pik3cg	0.001268472	0.00	223.6%
Pik3ip1	9.372722	2.11	22.5%
Pik3r1	45.4527	1.59	3.5%
Pik3r2	18.38218	1.03	5.6%
Pik3r3	17.37818	1.10	6.3%
Pik3r4	8.200754	0.50	6.1%
Pik3r5	0.0579926	0.01	20.5%
Pik4ca	9.46739	0.71	7.5%
Pilra	0.067121	0.08	114.6%
Pilrb1	0	0.00	
Pilrb2	0	0.00	
Pim1	1.717414	0.16	9.5%
Pim2	10.011158	0.68	6.8%
Pim3	12.4098	1.60	12.9%
Pin1	13.98796	1.06	7.6%
Pin4	0.427388	0.22	50.7%
Pink1	48.98274	3.65	7.5%
PINK1	0.1575996	0.07	45.2%
Pip	0	0.00	
Pip4k2a	9.342846	0.99	10.5%
Pip4k2b	32.57694	1.15	3.5%
Pip4k2c	10.572964	0.66	6.2%
Pip5k1a	11.81566	0.88	7.5%
Pip5k1b	2.596956	0.29	11.3%
Pip5k1c	46.39676	0.93	2.0%
Pip5k3	7.237466	2.49	34.4%
Pip5kl1	5.395348	0.30	5.5%
Pipox	0.6806252	0.06	8.5%
Pir	0.3711022	0.05	14.7%
Pira1	0	0.00	
Pira11	0	0.00	
Pira2	0	0.00	
Pira3	0	0.00	
Pira4	0	0.00	
Pira6	0	0.00	
Pirb	0	0.00	
Pisd	35.18242	2.38	6.8%
Pitpna	83.9069	1.63	1.9%
Pitpnb	23.40094	1.25	5.3%
Pitpnc1	14.30224	0.88	6.1%
Pitpnm1	38.77862	2.04	5.3%

Pitpnm2	11.92682	0.32	2.7%
Pitpnm3	4.797774	0.30	6.3%
Pitrm1	10.39678	0.12	1.2%
Pitx1	0.4558926	0.15	32.9%
Pitx2	0.460754	0.17	37.3%
Pitx3	0.08877874	0.06	68.9%
Piwil1	0.000796832	0.00	223.6%
Piwil2	0.2065288	0.01	7.1%
Piwil4	0.00215016	0.00	223.6%
piwiL4	0.000995334	0.00	223.6%
Pja1	71.23906	3.36	4.7%
Pja2	169.9828	4.63	2.7%
Pkd1	5.347918	1.16	21.7%
Pkd1l1	0.01926006	0.03	137.7%
Pkd1l2	0.002152734	0.00	153.0%
Pkd1l3	0.26929088	0.29	106.4%
PKD1L3	0.0099087	0.02	223.6%
Pkd2	3.13032	0.22	7.0%
Pkd2l1	0.03908444	0.01	37.1%
Pkd2l2	3.026025	4.83	159.6%
Pkdrej	0.008540908	0.01	127.3%
Pkhd1	0.01439428	0.01	35.9%
Pkhd1l1	0.011087558	0.01	97.7%
Pkia	122.587	4.49	3.7%
Pkib	0.576634	0.11	19.9%
Pkig	8.990564	1.88	20.9%
Pklr	0.1844036	0.08	45.2%
Pkm2	147.6614	12.66	8.6%
Pkmyt1	1.1656262	0.28	23.7%
Pkn1	6.256092	0.15	2.3%
Pkn2	3.547732	0.38	10.7%
Pkn3	0.7708418	0.43	55.8%
Pknox1	4.786164	0.32	6.8%
Pknox2	8.900756	0.19	2.2%
Pkp1	0.00223516	0.00	223.6%
Pkp2	0.8774964	0.09	10.6%
Pkp3	0.009826774	0.01	107.3%
Pkp4	36.39644	1.45	4.0%
Pla1a	0.014788	0.02	108.0%
Pla2g10	0.03769228	0.04	116.1%
Pla2g12a	6.520936	0.35	5.3%
Pla2g12b	0.07444824	0.04	51.8%
Pla2g1b	0	0.00	
Pla2g2a	0	0.00	
Pla2g2c	0	0.00	
Pla2g2d	0.06137398	0.03	46.0%
Pla2g2e	0	0.00	

Pla2g2f	0.01092122	0.01	111.8%
Pla2g3	0.2192504	0.05	21.7%
Pla2g4a	0.7526234	0.10	13.0%
Pla2g4b	0.6261126	0.06	10.0%
Pla2g4c	0.1570656	0.05	31.4%
Pla2g4d	0	0.00	
Pla2g4e	1.156201	0.13	11.2%
Pla2g4f	0.00697536	0.01	150.0%
Pla2g5	0.1086109	0.05	43.0%
Pla2g6	10.76868	0.42	3.9%
Pla2g7	46.39768	2.16	4.7%
Pla2r1	0.00852498	0.01	155.7%
Plaa	17.60498	1.23	7.0%
Plac1	0.06550274	0.02	22.9%
Plac1l	0	0.00	
Plac8	1.754794	0.58	32.8%
Plac8l1	0.03811252	0.05	143.2%
Plac9	0	0.00	
Plag1	0.3592586	0.06	18.0%
Plagl1	20.93396	3.46	16.5%
Plagl2	1.491138	0.16	10.5%
Plat	7.795932	0.63	8.1%
Plau	0.886553	0.21	23.3%
Plaur	0.15957888	0.12	73.0%
Plb1	0.00578444	0.01	145.2%
Plcb1	16.29814	1.24	7.6%
Plcb2	0.03741896	0.03	74.0%
Plcb3	2.382964	0.19	7.9%
Plcb4	22.0743	1.74	7.9%
Plcd	2.8280948	2.00	70.9%
Plcd1	1.282674	0.06	5.0%
Plcd3	3.817084	0.19	4.9%
Plcd4	2.39344	0.36	15.2%
Plce1	2.02144	0.11	5.6%
PLC-epsilon	0.572161	0.13	22.0%
Plcg1	15.65756	0.66	4.2%
Plcg2	0.14658608	0.05	33.3%
Plch1	3.275514	0.39	11.8%
Plch2	0.565623	0.04	7.2%
Plcl1	8.342766	0.52	6.2%
Plcl2	9.019482	0.47	5.2%
Plcl4	0.110528	0.06	53.9%
Plcxd1	2.920586	0.14	4.7%
Plcxd3	13.65154	1.45	10.6%
Plcz1	0.08331902	0.02	25.0%
Pld1	1.198508	0.15	12.7%
Pld2	2.424514	0.33	13.5%

Pld3	118.7116	3.29	2.8%
Pld4	0.00614864	0.01	138.1%
Pld5	5.172322	0.50	9.7%
Pldn	13.80722	1.27	9.2%
Plec1	8.952552	0.91	10.1%
Plek	2.543832	0.99	38.8%
Plek2	0.1709254	0.05	26.9%
Plekha1	15.1978	0.37	2.4%
Plekha2	0.8337424	0.17	20.5%
Plekha3	9.270452	0.49	5.3%
Plekha4	0.4106806	0.06	13.7%
Plekha5	6.706872	0.65	9.6%
Plekha6	27.25728	1.31	4.8%
Plekha7	0.8537162	0.11	13.0%
Plekha8	2.439766	0.45	18.6%
Plekhb1	3.901528	0.39	10.0%
Plekhb2	108.7888	3.05	2.8%
Plekhc1	9.072384	0.46	5.0%
Plekhf1	0.8128982	0.17	20.8%
Plekhf2	1.223758	0.06	4.9%
Plekhg1	1.7615	0.31	17.8%
Plekhg2	1.128535	0.13	11.7%
Plekhg3	0.4881824	0.15	30.3%
Plekhg4	0.6966322	0.39	56.2%
Plekhg5	1.45799	0.40	27.4%
Plekhg6	0.0335884	0.03	89.7%
Plekhh1	0.6524706	0.06	9.7%
Plekhh2	0.6960064	0.12	16.9%
Plekhh3	5.633038	0.58	10.4%
Plekhj1	15.65792	1.09	7.0%
Plekhk1	0.487047	0.06	13.3%
Plekhm1	4.82109	0.25	5.1%
Plekhm2	7.255788	0.65	8.9%
Plekhn1	3.369798	1.09	32.3%
Plekho1	3.242868	0.32	9.9%
Plekhq1	0.7791952	0.14	17.4%
Plg	0.01755792	0.01	81.3%
Plin	0.01572896	0.01	70.9%
Plk1	2.212772	0.43	19.3%
Plk2	19.86538	0.64	3.2%
Plk3	6.064672	0.57	9.4%
Plk4	1.621992	0.19	11.7%
Pllp	0.6854446	0.13	18.5%
Pln	0.0080211	0.01	137.0%
Plod1	5.752432	1.39	24.1%
Plod2	5.505048	0.81	14.7%
Plod3	8.09824	0.62	7.6%

Plp1	3.176942	1.42	44.8%
Plp2	0.09419034	0.06	63.2%
Plrg1	19.1455	0.64	3.4%
PLRG1	0.5080536	0.15	30.2%
Pls3	27.3175	1.90	7.0%
Plscr1	0.5452278	0.11	20.6%
Plscr2	0.2940154	0.05	16.2%
Plscr3	4.283668	0.71	16.6%
Plscr4	0.252456	0.06	23.8%
Pltp	3.673904	0.70	19.0%
Plunc	0	0.00	
Plvap	9.158682	0.47	5.1%
Plxdc1	1.83503	0.22	11.9%
Plxdc2	9.518544	1.17	12.3%
Plxna1	17.458	0.61	3.5%
Plxna2	17.10986	1.37	8.0%
Plxna3	8.214974	0.37	4.5%
Plxna4	7.722726	0.81	10.5%
Plxnb1	2.279218	0.08	3.3%
Plxnb2	11.32944	0.34	3.0%
Plxnb3	0.08745046	0.05	53.3%
Plxnc1	5.86101	0.49	8.4%
Plxnd1	0.3505958	0.05	13.1%
Pmaip1	1.840864	0.36	19.4%
PMCA2	34.13224	2.46	7.2%
Pmch	0.16639938	0.05	30.3%
Pmf1	5.04001	0.49	9.7%
Pmfbp1	0.013381734	0.01	89.6%
Pml	1.128096	0.06	5.6%
Pmm1	56.32428	3.92	7.0%
Pmm2	3.305444	0.55	16.5%
Pmp2	0	0.00	
Pmp22	5.918714	1.24	21.0%
Pmpca	32.37538	2.10	6.5%
Pmpcb	18.75246	0.62	3.3%
pmr1	0.8702678	0.58	66.7%
Pms1	1.1169072	0.12	10.4%
Pms2	3.313486	0.20	6.0%
PMUR10F	0.0039867	0.01	223.6%
Pmvk	26.16686	2.74	10.5%
Pnck	36.1345	4.52	12.5%
Pnkd	88.41104	7.34	8.3%
Pnkp	3.749806	0.20	5.3%
Pnlip	0.013742	0.01	96.8%
Pnliprp1	0.0616988	0.04	66.1%
Pnliprp2	0.03006932	0.02	74.0%
Pnma1	0.4121336	0.13	32.2%

Pnma2	17.33732	1.21	7.0%
Pnma3	0.700659	0.06	9.0%
Pnma5	0.04535924	0.03	65.7%
Pnmt	0	0.00	
Pnn	20.5464	1.04	5.1%
Pno1	11.26594	1.25	11.1%
Pnoc	1.644292	0.24	14.9%
Pnp	0.5391488	0.68	125.3%
Pnpla1	0.001607282	0.00	223.6%
Pnpla2	3.992486	0.35	8.9%
Pnpla3	4.976248	0.42	8.4%
Pnpla5	0	0.00	
Pnpla6	19.46618	0.40	2.0%
Pnpla7	0.8131188	0.04	5.2%
Pnpla8	29.38964	1.26	4.3%
Pnpo	15.7004	0.53	3.4%
Pnpt1	9.368272	0.54	5.8%
Pnr	0	0.00	
Pnrc1	8.654556	1.30	15.0%
Pnrc2	13.94796	0.64	4.6%
Podn	1.1646446	0.25	21.6%
Podnl1	0.04609088	0.02	47.9%
Podxl	4.039074	0.91	22.5%
Podxl2	90.70426	2.47	2.7%
Pof1b	0.1580242	0.04	25.5%
Pofut1	3.373156	0.18	5.4%
Pofut2	9.824612	0.69	7.1%
Pog	0.1571761	0.11	72.6%
Pogk	4.99075	0.56	11.2%
Pogz	9.338864	0.43	4.6%
Pol	0	0.00	
Pol_gamma	0.2687522	0.17	63.9%
Pola1	0.9205764	0.20	21.5%
POLA1	0.32150764	0.47	146.7%
Pola2	3.314218	0.15	4.6%
Polb	21.09392	1.32	6.2%
Pold1	0.992173	0.15	15.0%
Pold2	12.10434	0.93	7.7%
Pold3	3.81763	0.19	5.0%
Pold4	11.33236	0.28	2.5%
Poldip2	26.5817	0.61	2.3%
Poldip3	35.75128	1.59	4.4%
Pole	0.6705268	0.16	23.4%
Pole1	0.1834135	0.11	61.0%
Pole2	0.463963	0.10	22.4%
Pole3	6.24265	0.41	6.5%
Pole4	19.3447	1.73	8.9%

Polg	5.88474	0.56	9.5%
Polg2	0.9734458	0.52	53.6%
Polh	1.166276	0.13	10.8%
Poli	2.302082	0.15	6.7%
PolI	0.1589784	0.09	55.3%
Polk	2.384456	0.34	14.3%
Poll	2.921762	0.36	12.5%
Polm	1.25641	0.07	5.3%
Poln	0.370504	0.25	68.5%
Polq	0.2991684	0.04	12.3%
Polr1e	2.455642	0.26	10.5%
Polr2a	20.83264	0.94	4.5%
Polr2b	21.8555	0.65	3.0%
Polr2c	5.388874	0.16	3.0%
Polr2d	14.42608	1.43	9.9%
Polr2e	15.7884	1.20	7.6%
Polr2f	30.2751	2.86	9.4%
Polr2g	49.65874	1.27	2.6%
Polr2h	5.206554	0.47	9.1%
Polr2i	7.467654	0.42	5.6%
Polr2j	25.86388	2.31	8.9%
Polr2k	1.3691888	0.34	24.6%
Polr3a	5.637686	0.30	5.3%
Polr3b	4.912394	0.41	8.4%
Polr3c	9.225278	1.00	10.8%
Polr3d	7.737222	0.52	6.7%
Polr3e	6.832498	0.26	3.9%
Polr3f	5.384634	0.28	5.2%
Polr3g	1.799102	0.22	12.1%
Polr3gl	4.227884	0.31	7.3%
Polr3h	7.908286	0.85	10.8%
Polr3k	26.21596	1.55	5.9%
Polrmt	5.873812	0.40	6.8%
Pols	10.6121	0.59	5.5%
Pom121	14.28398	0.59	4.1%
Pomc1	0.510455	0.08	16.3%
Pomgnt1	13.8697	1.06	7.7%
Pomp	52.46446	2.75	5.2%
Pomt1	4.941354	0.27	5.4%
Pomt2	6.544412	0.29	4.5%
Pon1	0.0096217	0.01	137.6%
Pon2	5.921456	0.35	5.9%
Pon3	0.06003272	0.04	66.3%
Pop1	2.198056	0.22	10.2%
Pop4	12.5563	1.17	9.4%
Pop5	9.020156	0.52	5.8%
Pop7	10.183606	1.21	11.9%

Popdc2	0.2703074	0.09	31.9%
Popdc3	1.286982	0.45	35.0%
Por	20.35882	2.02	9.9%
Porcn	14.61238	0.46	3.2%
Postn	29.36468	5.24	17.8%
Pot1a	4.340118	0.22	5.2%
Pot1b	0.4351668	0.06	14.5%
Pou1f1	0.00610424	0.01	223.6%
Pou2af1	0.01109712	0.01	99.8%
Pou2f1	1.589834	0.25	15.9%
Pou2f2	0.2650916	0.06	22.5%
Pou2f3	0.00528218	0.01	138.0%
Pou3f1	5.276528	0.85	16.1%
Pou3f2	6.410126	0.57	9.0%
Pou3f3	8.4521	0.37	4.4%
Pou3f4	0.969407	0.13	13.2%
Pou4f1	8.906972	0.49	5.5%
Pou4f2	6.563588	0.65	9.9%
Pou4f3	1.384986	0.30	21.7%
Pou5f1	0	0.00	
Pou6f1	14.03386	0.70	5.0%
Pou6f2	0.2991644	0.09	30.8%
Pp11r	0.00506506	0.01	223.6%
PP2A_B56_gamma_3	0.4624038	0.21	46.1%
Ppa1	32.27268	2.54	7.9%
Ppa2	11.7069	0.48	4.1%
Ppan	5.364278	0.84	15.6%
Ppap2a	22.74458	1.72	7.6%
Ppap2b	12.61138	1.32	10.5%
Ppap2c	2.026504	0.34	16.7%
Ppapdc1	7.92692	0.63	8.0%
Ppapdc1a	8.470898	0.50	5.9%
Ppapdc2	14.16222	1.02	7.2%
Ppapdc3	5.06617	0.10	1.9%
Ppara	0.2123884	0.06	28.5%
Ppard	2.597682	0.38	14.8%
Pparg	0.2167136	0.09	42.3%
Ppargc1a	8.42618	0.59	7.0%
Ppargc1b	2.403914	0.24	10.1%
Ppat	4.140678	0.21	5.1%
Ppbp	0	0.00	
Ppcdc	2.278554	0.16	7.1%
Ppcs	5.533732	0.29	5.2%
Ppef1	0.1140213	0.07	57.9%
Ppef2	0.05723042	0.02	34.6%
Ppfia1	1.852786	0.50	27.1%
Ppfia2	17.68584	1.66	9.4%

Ppfia3	39.69096	2.87	7.2%
Ppfia4	23.41112	1.05	4.5%
Ppfibp1	1.896628	0.15	7.9%
Ppfibp2	1.0141356	0.07	6.5%
Pphln1	13.31638	0.78	5.9%
Ppia	0.596213	0.06	10.1%
Ppib	71.6973	3.39	4.7%
Ppic	12.72524	1.30	10.2%
Ppid	15.86304	0.39	2.5%
Ppie	3.929756	0.14	3.5%
Ppif	20.64814	0.98	4.8%
Ppig	17.07694	0.69	4.0%
Ppih	0.8361436	0.17	20.3%
Ppil1	8.085996	1.38	17.1%
Ppil2	7.700854	0.39	5.0%
Ppil3	9.860008	2.70	27.4%
Ppil4	9.929778	0.43	4.3%
Ppil5	0.157265	0.05	30.3%
Ppl	0.00840724	0.01	75.2%
Ppm1a	71.76694	2.21	3.1%
Ppm1b	18.0627	0.27	1.5%
Ppm1d	3.33599	0.20	6.1%
Ppm1e	32.23034	1.80	5.6%
Ppm1f	13.52158	1.33	9.8%
Ppm1g	30.55036	3.19	10.4%
Ppm1h	13.80704	0.65	4.7%
Ppm1j	0.1804288	0.06	33.3%
Ppm1k	8.107872	1.07	13.2%
Ppm1l	10.332974	0.79	7.6%
Ppm1m	1.565932	0.17	10.9%
Ppm2c	19.24464	0.53	2.7%
Ppme1	47.82058	1.29	2.7%
Ppnr	0.00353212	0.01	223.6%
Ppnx	0.06930262	0.03	42.0%
Ppox	2.552202	0.25	9.8%
Ppp1ca	109.6516	7.53	6.9%
Ppp1cb	128.7348	5.30	4.1%
Ppp1cc	2.070596	0.18	8.7%
Ppp1r10	10.883322	0.83	7.6%
Ppp1r11	36.82076	1.96	5.3%
Ppp1r12a	9.920746	0.52	5.2%
Ppp1r12b	6.483294	0.18	2.7%
Ppp1r12c	13.835	1.08	7.8%
Ppp1r13b	17.35754	0.95	5.5%
Ppp1r13l	0.4853488	0.10	21.4%
Ppp1r14a	0.05874178	0.07	112.9%
Ppp1r14b	4.387724	0.45	10.3%

Ppp1r14c	5.444546	0.90	16.6%
Ppp1r14d	0	0.00	
Ppp1r15b	11.96022	0.65	5.4%
Ppp1r16a	12.74932	1.08	8.5%
Ppp1r16b	6.248796	0.20	3.1%
Ppp1r1a	12.2139	1.53	12.5%
Ppp1r1b	0.2501518	0.12	47.4%
Ppp1r1c	0.217673	0.05	20.7%
Ppp1r2	3.383228	0.35	10.5%
Ppp1r3a	0	0.00	
Ppp1r3b	0.4739116	0.17	36.7%
Ppp1r3c	4.406722	0.87	19.7%
Ppp1r3d	2.601294	0.26	9.8%
Ppp1r3f	9.84616	0.49	5.0%
Ppp1r7	17.56204	1.08	6.2%
Ppp1r8	15.27788	1.07	7.0%
Ppp1r9a	14.8946	1.23	8.2%
Ppp1r9b	56.1895	1.83	3.3%
Ppp2ca	179.3636	8.99	5.0%
Ppp2cb	46.3168	2.12	4.6%
Ppp2r1a	236.4966	12.65	5.3%
Ppp2r1b	13.28614	0.83	6.3%
Ppp2r2a	17.55234	1.16	6.6%
Ppp2r2b	63.17294	4.06	6.4%
Ppp2r2c	79.9021	1.26	1.6%
Ppp2r2d	29.12832	2.35	8.1%
Ppp2r3a	0	0.00	
Ppp2r3c	8.255334	0.73	8.8%
Ppp2r4	46.1943	1.46	3.2%
Ppp2r5a	1.706552	0.17	10.1%
Ppp2r5b	69.29342	5.71	8.2%
Ppp2r5c	47.81706	2.30	4.8%
Ppp2r5d	35.08514	1.22	3.5%
Ppp2r5e	27.64208	1.05	3.8%
Ppp3ca	126.7874	5.88	4.6%
Ppp3cb	93.00618	2.60	2.8%
Ppp3cc	6.802722	0.84	12.3%
Ppp3r1	206.519	5.60	2.7%
Ppp3r2	0.04349908	0.03	61.2%
Ppp4c	14.35806	0.87	6.0%
Ppp4r1	7.198398	0.46	6.4%
Ppp4r1l	3.792186	0.26	6.8%
Ppp4r2	7.031674	0.23	3.3%
Ppp5c	39.86064	1.76	4.4%
Ppp6c	20.09794	1.18	5.9%
Pprc1	5.78714	0.55	9.5%
Pps	8.273974	0.23	2.7%

Ppt1	42.46052	1.74	4.1%
Ppt2	8.497616	0.22	2.6%
Pptc7	12.91558	0.73	5.7%
Ppwd1	3.340398	0.29	8.6%
Ppy	0.01274926	0.03	223.6%
Ppyr1	0.00783876	0.01	140.6%
Pqbp1	29.05828	1.43	4.9%
Pqlc1	6.854086	0.25	3.7%
Pqlc2	5.121404	0.38	7.5%
Pqlc3	0.8940686	0.17	19.4%
Pra1	6.729722	2.00	29.8%
Praf2	38.60972	2.04	5.3%
Pram1	3.4642936	2.54	73.2%
Pramef12	0	0.00	
Pramel1	0.01120754	0.01	99.9%
Pramel3	0	0.00	
Pramel4	0	0.00	
Pramel5	0	0.00	
Pramel6	0	0.00	
Pramel7	0	0.00	
Prap1	0	0.00	
Prb1	0	0.00	
Prc1	1.568736	0.24	15.6%
Prcc	16.65428	0.71	4.3%
Prcd	1.1491918	0.39	34.3%
Prcp	3.54409	0.27	7.6%
Prdm1	0.245205	0.06	24.7%
Prdm10	2.941442	0.23	7.9%
Prdm13	0.08817428	0.03	33.1%
Prdm14	0.00707596	0.01	145.6%
Prdm15	1.960884	0.14	7.3%
Prdm16	0.2260114	0.02	10.9%
Prdm2	4.683474	0.14	3.0%
Prdm4	7.337914	0.28	3.8%
Prdm5	0.5003604	0.13	26.5%
Prdm6	0.3635802	0.10	28.2%
Prdm8	0.719963	0.13	18.1%
Prdm9	0.4318626	0.12	28.3%
Prdx1	57.97868	3.19	5.5%
Prdx2	97.86426	8.31	8.5%
Prdx3	63.7003	4.06	6.4%
Prdx4	12.63036	1.25	9.9%
Prdx5	170.8746	8.82	5.2%
Prdx6	28.84872	2.06	7.1%
Prdx6-rs1	1.433614	0.08	5.5%
Preb	22.72678	0.24	1.0%
Prei4	14.3537	2.24	15.6%

Prelid1	30.69076	2.00	6.5%
Prelid2	0.2386906	0.14	60.3%
Prelp	2.29037	0.70	30.4%
Prep	21.74736	2.16	9.9%
PREP2	0.06712188	0.01	17.5%
Prepl	192.9118	6.48	3.4%
P-Rex2	0.10758028	0.03	27.8%
Prf1	0	0.00	
Prg2	0	0.00	
Prg3	0	0.00	
Prg4	1.24791236	0.85	68.1%
Prh1	0	0.00	
Prhoxnb	0	0.00	
Prickle1	13.019	0.28	2.2%
Prickle2	6.989738	0.75	10.7%
Prim1	2.997024	0.18	5.8%
Prim2	2.000092	0.30	15.0%
Prima1	6.46263	0.74	11.4%
Prip	0.09087138	0.04	46.3%
Prkaa1	10.729478	1.02	9.5%
Prkaa2	5.747532	0.54	9.5%
Prkab1	5.235312	0.27	5.2%
Prkab2	13.09808	0.63	4.8%
Prkaca	123.056	5.19	4.2%
Prkacb	185.978	11.44	6.2%
Prkag1	14.72152	0.90	6.1%
Prkag2	13.73284	0.65	4.7%
Prkag3	0	0.00	
Prkar1a	236.3182	2.17	0.9%
Prkar1b	197.2958	3.30	1.7%
Prkar2a	24.47482	2.11	8.6%
Prkar2b	35.57186	2.77	7.8%
Prkca	8.821484	0.29	3.3%
Prkcb1	63.1353	1.66	2.6%
Prkcbp1	9.078206	0.29	3.2%
Prkcc	3.36662	0.47	13.9%
Prkcd	1.88073	0.16	8.7%
Prkcdbp	0.5054862	0.05	9.0%
Prkce	24.99738	1.54	6.1%
Prkch	1.292504	0.11	8.3%
Prkci	17.48144	0.52	2.9%
Prkcm	1.2114344	0.21	17.3%
Prkcn	1.702666	0.20	12.0%
Prkcq	0.8239376	0.11	13.8%
Prkcsh	41.46156	1.94	4.7%
Prkcz	11.35856	0.12	1.1%
Prkd2	0.8927478	0.12	13.9%

Prkdc	1.0521954	0.06	5.5%
Prkg1	1.840492	0.14	7.7%
Prkg1b	0.03524158	0.01	20.2%
Prkg2	0.5149804	0.05	9.9%
Prkra	13.5369	0.61	4.5%
Prkrip1	4.874498	0.15	3.1%
Prkrir	14.44896	0.86	6.0%
PRKWNK4	0.08479812	0.04	43.8%
Prkx	4.94095	0.21	4.2%
Prl	0	0.00	
Prl2a1	0	0.00	
Prl2b1	0	0.00	
Prl2c2	0	0.00	
Prl2c3	0	0.00	
Prl2c5	0	0.00	
Prl3a1	0	0.00	
Prl3b1	0	0.00	
Prl3c1	0	0.00	
Prl3d1	0	0.00	
Prl3d2	0	0.00	
Prl3d3	0	0.00	
Prl4a1	0.0082849	0.02	223.6%
Prl5a1	0	0.00	
Prl6a1	0	0.00	
Prl7a1	0	0.00	
Prl7a2	0	0.00	
Prl7b1	0	0.00	
Prl7c1	0	0.00	
Prl7d1	0	0.00	
Prl8a1	0	0.00	
Prl8a2	0	0.00	
Prl8a6	0	0.00	
Prl8a8	0	0.00	
Prl8a9	0	0.00	
Prlhr	0.05499884	0.05	86.3%
Prlpc1	0	0.00	
Prlpc3	0	0.00	
Prlr	1.569276	0.23	14.7%
Prm1	0.13022	0.05	34.7%
Prm2	0.01144102	0.03	223.6%
Prm3	0	0.00	
Prmt1	49.94108	5.31	10.6%
Prmt2	38.17156	4.59	12.0%
Prmt3	12.28566	0.57	4.6%
Prmt5	24.708	1.80	7.3%
Prmt6	7.101044	0.45	6.4%
Prmt7	17.36806	1.52	8.7%

Prmt8	10.791648	1.88	17.4%
Prnd	0.03062812	0.01	43.0%
Prnp	121.4808	8.29	6.8%
Prnpip1	59.69096	2.77	4.6%
Proc	0	0.00	
Procr	0.2939372	0.14	48.7%
Prodh	0.6607412	0.04	6.6%
Prodh2	0.00764494	0.01	137.0%
Prok1	0.0817727	0.07	87.7%
Prok2	1.042911	0.24	23.0%
Prokr1	0.1970798	0.07	33.7%
Prokr2	3.60874	0.31	8.6%
Prom1	0.648644	0.12	17.9%
Prom2	0.001338982	0.00	223.6%
Prop1	0	0.00	
Pros1	4.412364	0.34	7.6%
Prosapip1	29.68506	0.89	3.0%
Prosc	20.012	1.54	7.7%
Prox1	0.660531	0.07	10.7%
Prox2	0.2190956	0.03	15.7%
Proz	0.3760152	0.08	21.2%
Prp2	0	0.00	
Prpf18	11.45996	0.29	2.6%
Prpf19	93.19468	3.52	3.8%
Prpf3	4.092352	0.24	5.8%
Prpf31	13.81282	0.72	5.2%
Prpf38a	8.289388	1.00	12.1%
Prpf38b	10.773214	1.31	12.1%
Prpf39	12.245228	2.13	17.4%
Prpf4	3.762398	0.17	4.5%
Prpf40a	11.84678	0.84	7.1%
Prpf40b	15.55318	0.92	5.9%
Prpf4b	20.55668	1.03	5.0%
Prpf6	16.35352	0.13	0.8%
Prpf8	34.74654	1.61	4.6%
Prph	0.8461014	0.15	18.3%
Prph2	0	0.00	
Prpmp5	0	0.00	
Prps1	33.22936	2.60	7.8%
Prps1l1	0	0.00	
Prps2	7.153478	0.51	7.1%
Prpsap1	19.80206	0.59	3.0%
Prpsap2	4.34374	0.28	6.4%
Prr11	0.6904064	0.07	10.7%
Prr12	9.166406	0.50	5.5%
Prr13	39.92828	2.53	6.3%
Prr14	6.474512	0.32	5.0%

Prr15	0.12721558	0.09	67.5%
Prr16	1.706086	0.33	19.2%
Prr18	2.62365	0.16	5.9%
Prr3	20.81782	0.79	3.8%
Prr6	5.098134	0.38	7.4%
Prr7	5.484456	0.88	16.0%
Prr8	7.518218	0.74	9.8%
Prrc1	7.901672	0.69	8.7%
Prrg1	1.518538	0.21	14.1%
Prrg2	1.445068	0.27	18.9%
Prrg3	4.970178	0.44	8.9%
Prrg4	0.4251768	0.11	26.0%
Prrt1	1.130454	0.08	6.7%
Prrt3	11.07638	0.57	5.1%
Prrx1	2.28812	0.44	19.1%
Prrx2	0.331204	0.14	41.5%
Prrxl1	0.0662278	0.05	75.9%
Prss1	0.00956928	0.02	223.6%
Prss12	1.725458	0.30	17.1%
Prss16	0.07121618	0.03	47.9%
Prss2	0	0.00	
Prss21	0	0.00	
Prss22	0	0.00	
Prss23	1.375022	0.16	11.4%
Prss27	0	0.00	
Prss28	0	0.00	
Prss29	0	0.00	
Prss3	0	0.00	
Prss32	0.02987638	0.04	143.5%
Prss33	0	0.00	
Prss34	0	0.00	
Prss35	1.0237674	0.16	16.0%
Prss36	4.276292	0.25	5.8%
Prss7	0	0.00	
Prss8	1.1099387	0.74	66.7%
Prssl1	0.09347136	0.02	24.5%
Prtg	0.1510174	0.02	12.9%
Prtn3	2.438142	0.73	30.1%
Prune	11.5694	1.14	9.8%
Prx	0.2215806	0.03	12.4%
Psap	463.7	8.80	1.9%
Psapl1	0	0.00	
Psat1	48.4949	10.75	22.2%
Psca	0.2017562	0.08	39.9%
Pscd1	11.85828	0.55	4.7%
Pscd2	15.48186	1.23	7.9%
Pscd3	13.01344	0.48	3.7%

Pscd4	0.04113332	0.01	27.3%
Pscdbp	0.09901648	0.06	61.0%
Psd	63.17686	2.38	3.8%
Psd2	11.6132	0.42	3.6%
Psd3	16.31564	2.17	13.3%
Psd4	0.05730572	0.03	52.6%
Psen1	19.81938	1.22	6.2%
Psen2	7.0081	0.50	7.1%
Psenen	2.28887	0.32	14.1%
Psg16	0.6499858	0.12	18.3%
Psg17	0.0098765	0.01	139.5%
Psg18	0	0.00	
Psg19	0.00428004	0.01	223.6%
Psg21	0.00277072	0.01	223.6%
Psg23	0	0.00	
Psg25	0	0.00	
Psg28	0.00325778	0.01	223.6%
Psg29	0	0.00	
Psg30	0	0.00	
Psip1	54.81826	1.99	3.6%
Pskh1	2.908654	0.19	6.4%
Psma1	57.9238	3.44	5.9%
Psma2	48.19134	2.48	5.1%
Psma3	6.485062	0.60	9.2%
Psma4	24.49122	1.70	6.9%
Psma5	5.157928	0.08	1.6%
Psma6	66.03794	2.52	3.8%
Psma7	71.80478	5.10	7.1%
Psma8	0	0.00	
Psmb1	60.43972	3.81	6.3%
Psmb10	13.69338	1.66	12.1%
Psmb2	50.55566	4.79	9.5%
Psmb3	5.94637	0.77	13.0%
Psmb4	86.16174	5.95	6.9%
Psmb5	20.43644	1.37	6.7%
Psmb6	52.21682	3.72	7.1%
Psmb7	91.14264	3.08	3.4%
Psmb8	0.3455262	0.11	31.5%
Psmb9	0.8122606	0.23	28.0%
Psmbeta	0.19022838	0.14	74.2%
Psmc1	24.792	2.49	10.0%
Psmc2	48.44596	0.64	1.3%
Psmc3	38.64546	3.17	8.2%
Psmc3ip	0.9606222	0.14	14.4%
Psmc4	40.94468	2.31	5.6%
Psmc5	60.02412	4.43	7.4%
Psmc6	60.14298	2.02	3.4%

Psmd1	62.89868	3.49	5.5%
Psmd10	6.615798	0.44	6.6%
Psmd11	7.780198	0.28	3.6%
Psmd12	42.50216	1.90	4.5%
Psmd13	38.79164	2.13	5.5%
Psmd14	34.86188	1.30	3.7%
Psmd2	47.29542	1.65	3.5%
Psmd3	44.0829	2.20	5.0%
Psmd4	51.0926	3.75	7.3%
Psmd5	25.03486	1.58	6.3%
Psmd6	50.44928	3.65	7.2%
Psmd7	47.5167	3.50	7.4%
Psmd8	45.12786	2.58	5.7%
Psmd9	7.294756	0.64	8.7%
Psme1	24.81422	1.96	7.9%
Psme2	1.546438	0.11	7.0%
Psme3	32.66612	0.98	3.0%
Psme4	16.87794	1.07	6.3%
Psmf1	14.08212	0.87	6.2%
Psmg3	8.365274	1.03	12.3%
Psors1c2	0.176383	0.07	38.6%
Psp	0.00682632	0.02	223.6%
Pspc1	19.49922	2.16	11.1%
Psph	5.151324	0.37	7.2%
Pspn	0.16219042	0.09	53.1%
Psrc1	1.90461	0.19	9.9%
Pstk	9.526182	0.12	1.3%
Pstpip1	0.01142596	0.02	156.5%
Pstpip2	1.0882274	0.14	12.5%
Ptafr	0	0.00	
Ptbp1	5.88957	0.68	11.6%
Ptbp2	54.614	1.99	3.6%
Ptcd1	5.759052	0.60	10.5%
Ptcd2	17.13684	0.76	4.4%
Ptcd3	18.10902	0.88	4.8%
PTCH	12.650622	4.71	37.2%
Ptch1	12.8297	2.14	16.7%
Ptch2	0.482339	0.16	32.6%
Ptchd1	10.53218	0.08	0.8%
Ptchd2	1.372802	0.12	8.5%
Ptchd3	0.00565534	0.01	140.7%
Ptcra	0.04108976	0.04	108.2%
Ptdsr	0.807417	0.15	18.8%
Ptdss1	30.04972	1.81	6.0%
Ptdss2	19.07062	1.64	8.6%
Pte2	1.072262	0.06	5.9%
Pten	39.81922	1.21	3.0%

Pter	1.09578	0.16	14.3%
Ptf1a	0.2203234	0.07	33.0%
Ptgdr	0.02893018	0.01	29.3%
Ptgds	0.533098	0.11	21.4%
Ptgds2	0.02799302	0.03	109.9%
Ptger1	3.414724	0.26	7.6%
Ptger2	0.01247124	0.02	152.4%
Ptger3	2.626682	0.30	11.5%
Ptger4	1.310546	0.15	11.3%
Ptges	0.06380882	0.01	21.3%
Ptges2	15.30284	1.23	8.1%
Ptges3	33.3305	1.73	5.2%
Ptgfr	0.09586382	0.03	32.9%
Ptgfrn	7.15798	0.34	4.8%
Ptgir	0.010248076	0.01	69.0%
Ptgis	5.181984	0.68	13.1%
Ptgs1	0.07061206	0.01	18.2%
Ptgs2	0.2114012	0.06	30.4%
Pth	0.0149816	0.02	137.6%
Pthb1	0.02417414	0.00	19.6%
Pthlh	3.022696	0.26	8.7%
Pthr1	2.299298	0.41	18.0%
Pthr2	0.2277582	0.05	23.1%
Ptk2	11.41718	0.62	5.5%
Ptk2b	0.298871	0.02	6.2%
Ptk6	0.05172744	0.03	67.6%
Ptk7	5.501566	0.40	7.3%
Ptma	14.91186	1.36	9.1%
Ptms	159.0674	21.37	13.4%
Ptn	93.6302	9.85	10.5%
Ptov1	62.72806	3.91	6.2%
Ptp4a1	7.266412	0.52	7.1%
Ptp4a2	25.84084	0.59	2.3%
Ptp4a3	10.756994	1.57	14.6%
ptpc1	1.4185846	0.55	38.5%
Ptpdc1	6.075478	0.62	10.2%
Ptpla	2.890268	0.45	15.6%
Ptplad1	78.27416	1.90	2.4%
Ptplad2	0.767092	0.08	10.9%
Ptplb	3.279474	0.18	5.6%
Ptpmt1	8.317346	0.19	2.3%
Ptpn1	6.455368	0.54	8.3%
Ptpn11	22.28718	0.73	3.3%
Ptpn12	8.368478	0.46	5.5%
Ptpn13	0.6938392	0.06	8.5%
Ptpn14	0.6506934	0.08	12.2%
Ptpn18	0.11357762	0.09	75.5%

Ptpn2	3.480096	0.27	7.8%
Ptpn20	0.00243442	0.01	223.6%
Ptpn21	1.308214	0.13	10.3%
Ptpn22	0.14653544	0.10	69.1%
Ptpn23	15.45164	0.82	5.3%
Ptpn3	1.561872	0.21	13.7%
Ptpn4	8.63363	1.20	13.9%
Ptpn5	40.14278	4.10	10.2%
Ptpn6	0.10577354	0.03	30.7%
Ptpn7	0.008701544	0.01	71.5%
Ptpn9	12.02546	0.72	6.0%
Ptpra	27.70354	0.88	3.2%
Ptprb	0.03378676	0.01	35.1%
Ptprc	0.00145246	0.00	223.6%
Ptprcap	0.09331794	0.05	56.5%
Ptprd	28.29826	2.35	8.3%
Ptpre	3.055802	0.23	7.5%
Ptprf	13.83398	0.47	3.4%
Ptprg	15.86192	0.77	4.8%
Ptprh	0.0496168	0.03	59.9%
Ptprj	5.673712	0.16	2.8%
Ptprk	11.90876	0.28	2.3%
Ptprm	7.45406	0.18	2.4%
Ptprn	75.25406	1.66	2.2%
Ptprn2	59.37984	2.54	4.3%
Ptpro	14.77758	1.38	9.3%
Ptprq	0	0.00	
Ptprr	5.52492	0.31	5.6%
Ptprs	68.4817	2.00	2.9%
Ptprt	14.15774	1.95	13.8%
Ptpru	3.850198	0.14	3.7%
Ptprv	0.5971602	0.09	14.6%
Ptprz1	15.15744	1.29	8.5%
Ptrf	1.67581	0.31	18.7%
Ptrh1	4.52861	0.56	12.3%
Ptrh2	4.10899	0.41	10.0%
Pts	6.62069	0.72	10.9%
Pttg1	0.8165988	0.25	31.1%
Pttg1ip	11.63048	0.90	7.7%
Ptx3	0.0787272	0.01	9.6%
Puf60	62.15232	5.12	8.2%
Pum1	15.8808	0.61	3.8%
Pum2	25.92326	1.40	5.4%
Punc	0.6849206	0.06	9.3%
Pura	9.568272	1.23	12.9%
Purb	13.83684	0.58	4.2%
Purg	6.570882	0.61	9.2%

Pus1	7.804492	0.38	4.9%
Pus3	4.412988	0.24	5.5%
Pus7	1.967788	0.26	13.2%
Pus7l	1.3485	0.22	16.4%
Pusl1	6.8766	0.41	6.0%
Pvalb	5.773684	1.59	27.5%
Pvr	1.238654	0.10	7.7%
Pvrl1	19.80626	2.53	12.8%
Pvrl2	8.902372	0.90	10.1%
Pvrl3	7.036862	0.44	6.2%
Pvrl4	0.06506958	0.02	26.0%
Pwp1	6.984474	0.65	9.3%
Pwp2	4.334014	0.21	4.9%
Pwwp2	1.780544	0.21	11.7%
Pxdn	9.654206	0.63	6.5%
Pxk	13.29438	0.28	2.1%
Pxmp2	0.9880712	0.17	17.2%
Pxmp3	15.5972	1.25	8.0%
Pxmp4	5.93818	0.67	11.2%
Pxn	3.010902	0.26	8.8%
Pxt1	0.0052473	0.01	223.6%
Pycard	0.04063648	0.02	47.8%
Pycr1	2.147162	0.57	26.4%
Pycr2	7.551522	0.29	3.8%
Pycrl	14.93132	1.58	10.5%
Pygb	54.96342	2.18	4.0%
Pygl	0.11055574	0.04	35.1%
Pygm	0.1806258	0.05	26.1%
Pygo1	10.022964	1.07	10.7%
Pygo2	13.5696	0.51	3.8%
Pyhin1	0.00753164	0.01	139.3%
Pyroxd1	3.879176	0.19	4.9%
Pyy	0.01364534	0.03	223.6%
Pzp	0.03605852	0.02	62.5%
Qars	16.803	1.71	10.2%
qbrick	0.06553942	0.02	23.2%
Qdpr	53.75714	3.34	6.2%
Qil1	0.11510488	0.02	20.3%
Qk	24.90942	1.14	4.6%
Qpct	8.430994	0.73	8.7%
Qpctl	6.696598	0.28	4.2%
Qprt	0.0133417	0.01	92.8%
Qrfp	0.02086912	0.01	66.1%
Qrich1	23.37084	0.22	1.0%
Qrich2	0.00628188	0.01	155.5%
Qrsl1	6.666792	0.58	8.7%
Qscn6	0.2775072	0.11	39.2%

Qscn6l1	3.6127	0.27	7.6%
Qser1	2.98973	0.20	6.8%
Qsox1	11.01254	0.74	6.7%
Qsox2	2.58621	0.30	11.6%
Qtrt1	3.222228	0.32	10.0%
Qtrtd1	3.100022	0.37	11.9%
R3hcc1	16.97468	1.90	11.2%
R3hdm1	26.5594	0.80	3.0%
R3hdm2	23.95266	1.51	6.3%
R74862	5.898838	1.23	20.9%
Rab1	102.89388	4.12	4.0%
Rab10	62.58832	4.70	7.5%
Rab11a	96.23746	3.24	3.4%
Rab11b	12.76214	0.96	7.5%
Rab11fip1	0.5173014	0.02	3.9%
Rab11fip2	18.73306	0.93	5.0%
Rab11fip3	11.95216	0.40	3.4%
Rab11fip4	24.06136	1.82	7.5%
Rab11fip5	34.71466	0.63	1.8%
Rab12	31.18938	1.83	5.9%
Rab13	1.666354	0.28	16.6%
Rab14	84.07046	4.62	5.5%
Rab15	26.73942	1.42	5.3%
Rab17	0.0375126	0.02	61.0%
Rab18	88.6038	1.40	1.6%
Rab19	0.02219026	0.02	86.4%
Rab1A	0.24396674	0.09	35.7%
Rab1b	48.42856	3.20	6.6%
Rab2	87.56754	1.90	2.2%
Rab20	0.08476156	0.06	75.6%
Rab21	32.45436	0.78	2.4%
Rab22a	8.249962	0.79	9.5%
Rab22A	1.347722	0.35	26.2%
Rab22B	0.2667702	0.08	30.0%
Rab23	6.295366	0.31	5.0%
Rab24	13.03502	0.27	2.1%
Rab25	0.04459474	0.04	98.7%
Rab26	1.515752	0.26	17.3%
Rab27a	1.603594	0.17	10.4%
Rab27b	6.773604	0.57	8.4%
Rab28	20.74706	0.82	4.0%
Rab29	0.271298	0.08	28.8%
Rab2A	1.331908	0.71	53.6%
Rab2b	16.2253	0.74	4.6%
Rab2B	0.6197944	0.27	43.3%
Rab30	8.957292	0.99	11.1%
Rab31	17.72632	1.27	7.2%

Rab32	0.4053594	0.08	18.8%
Rab33a	13.06406	2.81	21.5%
Rab33b	6.846068	0.70	10.3%
Rab34	7.222946	0.48	6.6%
Rab35	26.54464	1.42	5.3%
Rab36	5.045152	0.23	4.5%
Rab37	0.13153466	0.03	19.8%
Rab38	0.3676256	0.11	30.8%
Rab39	0.76691	0.09	11.1%
Rab39b	22.6995	0.73	3.2%
Rab3a	284.787	8.13	2.9%
Rab3b	10.584106	1.68	15.9%
Rab3c	58.7761	5.34	9.1%
Rab3d	4.736842	0.72	15.2%
Rab3gap1	14.6174	0.40	2.7%
Rab3gap2	17.35518	1.02	5.9%
Rab3il1	2.788928	0.33	11.7%
Rab3ip	6.837496	0.34	5.0%
Rab40b	1.869714	0.10	5.4%
Rab40c	11.93754	0.39	3.2%
Rab40C	0.2192032	0.07	31.7%
Rab41	0.2815292	0.06	22.7%
Rab42	0.004900795	0.01	118.6%
Rab43	4.21234	0.12	2.9%
Rab4a	20.84306	0.90	4.3%
Rab4b	40.27932	3.18	7.9%
Rab4B	0.17525614	0.10	56.4%
Rab5a	35.15032	1.12	3.2%
Rab5b	29.53284	1.08	3.7%
Rab5c	46.86576	2.27	4.8%
Rab6	164.2664	7.62	4.6%
Rab6b	201.4122	8.92	4.4%
Rab6ip1	36.70084	0.39	1.1%
Rab7	25.3578	1.40	5.5%
Rab7b	0.01294156	0.01	65.4%
Rab7l1	0.4115958	0.07	18.1%
Rab8a	13.05148	1.11	8.5%
Rab8A	0.2708118	0.11	39.7%
Rab8b	5.197196	0.62	12.0%
Rab8B	2.475186	0.30	12.3%
Rab9	16.54828	0.79	4.7%
Rab9b	21.2312	1.07	5.1%
Rabac1	101.918	11.74	11.5%
Rabep1	37.51048	1.68	4.5%
Rabep2	1.919038	0.31	16.0%
Rabepk	6.368044	0.43	6.7%
Rabgap1	25.11026	1.08	4.3%

Rabgap1l	29.45602	1.23	4.2%
Rabgef1	10.416984	0.44	4.2%
Rabggta	10.7442	0.59	5.5%
Rabggtb	33.01908	1.22	3.7%
Rabif	20.39146	1.10	5.4%
Rabj	0.1382036	0.04	32.5%
Rabl2a	13.05054	0.55	4.2%
Rabl3	5.644896	0.47	8.3%
Rabl4	14.29884	2.17	15.2%
Rabl5	20.44682	0.35	1.7%
Rac1	84.35034	1.98	2.3%
Rac2	0.2174334	0.05	24.4%
Rac3	47.71828	7.88	16.5%
Racgap1	4.75801	0.29	6.1%
Rad1	4.365178	0.55	12.6%
Rad17	3.397006	0.26	7.6%
Rad18	2.01978	0.12	6.0%
Rad18sc	0.11098134	0.03	28.2%
Rad21	49.79336	0.58	1.2%
Rad23a	21.69072	1.24	5.7%
Rad23b	61.21148	3.18	5.2%
Rad50	2.559766	0.16	6.1%
Rad51	0.9472184	0.05	5.3%
Rad51ap1	1.0555786	0.19	17.5%
Rad51c	0.7211334	0.04	6.0%
Rad51l1	0.08610516	0.05	61.3%
Rad51l3	1.4528794	1.09	75.3%
Rad52	3.756952	0.09	2.4%
Rad54l	2.63286	0.52	19.8%
Rad54l2	4.246324	0.14	3.4%
Rad9	6.421758	0.28	4.4%
Rad9b	3.48696	0.51	14.6%
radical-fringe	0.7061228	0.36	50.9%
Rae1	12.67252	0.73	5.7%
Raet1a	0.051878	0.02	33.4%
Raet1c	0.04626352	0.06	132.7%
Raet1d	0.06678196	0.04	67.3%
Raet1e	0.169625	0.04	25.0%
Raf1	29.21838	0.95	3.2%
Rag1	0.01642146	0.02	93.3%
Rag1ap1	5.29066	0.65	12.3%
Rag2	0	0.00	
Rage	1.940522	0.23	11.8%
Rai1	8.590804	1.84	21.4%
Rai12	26.52432	2.09	7.9%
Rai14	1.497064	0.22	14.9%
Rai16	11.54696	0.56	4.8%

Rai2	5.13674	0.21	4.0%
Rala	44.48698	6.70	15.1%
Ralb	12.6531	1.00	7.9%
Ralbp1	16.87588	0.48	2.8%
Ralgds	18.42852	2.60	14.1%
Ralgps1	9.042826	0.69	7.6%
Ralgps2	8.223068	0.61	7.4%
Raly	38.09456	2.25	5.9%
Ralyl	70.96528	2.33	3.3%
ramp	0.15446	0.03	18.9%
Ramp1	0.3048392	0.09	28.8%
RAMP1	0.08850588	0.04	43.7%
Ramp2	6.187886	0.76	12.3%
Ramp3	16.0218	2.29	14.3%
Ran	30.61876	0.51	1.7%
Ranbp1	27.71656	1.75	6.3%
Ranbp10	6.929944	0.16	2.3%
Ranbp17	2.795498	0.39	13.9%
Ranbp2	11.13037	0.85	7.6%
Ranbp3	21.22336	1.74	8.2%
Ranbp3l	0.01733714	0.01	69.9%
RanBP4	0.498639	0.13	26.7%
Ranbp5	18.98452	0.83	4.4%
Ranbp6	34.21396	0.80	2.3%
Ranbp9	21.3452	0.68	3.2%
Rangap1	65.479	1.44	2.2%
Rangrf	0.3696626	0.09	23.1%
RANK	0.10265274	0.06	53.8%
Rap1a	3.991508	0.29	7.4%
Rap1b	29.05664	2.28	7.9%
Rap1gap	47.18726	2.36	5.0%
Rap1gds1	90.8246	1.66	1.8%
Rap2a	23.91488	0.80	3.3%
Rap2b	6.868698	0.19	2.8%
Rap2c	13.04884	0.69	5.3%
Rapgef1	17.79572	0.45	2.5%
Rapgef2	8.581324	0.21	2.4%
Rapgef3	0.711127	0.06	8.9%
Rapgef4	33.59446	0.91	2.7%
Rapgef5	7.40494	0.47	6.3%
Rapgef6	13.523	0.72	5.3%
Rapgefl1	20.39398	0.55	2.7%
Raph1	14.2594	1.15	8.1%
Rapsn	0.00741928	0.01	141.7%
raptor	1.5079736	0.49	32.2%
Raptor	0.08023742	0.05	65.2%
Rara	2.499712	0.41	16.6%

Rarb	9.883884	2.43	24.5%
Rarg	1.873218	0.29	15.5%
Rarres1	0.1401017	0.07	48.7%
Rarres2	0.3531754	0.19	54.4%
Rars	22.05	1.33	6.1%
Rars2	9.951818	0.90	9.0%
Rasa1	16.20468	1.08	6.7%
Rasa2	4.524546	0.50	11.2%
Rasa3	8.40734	0.27	3.3%
Rasa4	0.3206738	0.11	35.6%
Rasal1	0.1179242	0.01	11.6%
Rasal2	4.250506	0.56	13.3%
Rasd1	0.2695314	0.07	26.5%
Rasd2	6.409198	0.85	13.2%
Rasef	0.05808422	0.02	39.1%
Rasgef1a	7.378876	0.52	7.1%
Rasgef1b	5.756292	0.26	4.5%
Rasgef1c	2.469232	0.26	10.4%
Rasgrf1	71.10814	7.27	10.2%
Rasgrf2	11.939742	1.49	12.5%
Rasgrp	0.0491199	0.01	15.5%
Rasgrp1	0.3629016	0.09	24.4%
Rasgrp2	46.36914	2.25	4.8%
Rasgrp3	0.012984132	0.01	112.9%
Rasgrp4	3.0499098	3.77	123.5%
Rasip1	0.8507542	0.19	21.9%
Rasl10a	0.9543738	0.31	33.0%
Rasl10b	54.3468	2.63	4.8%
Rasl11a	0.193808	0.02	10.3%
Rasl11b	1.218448	0.22	18.0%
Rasl12	0.03443316	0.03	75.9%
Rasl2-9	1.671336	0.18	10.9%
Rassf1	4.832766	0.46	9.5%
Rassf2	4.874316	0.25	5.2%
Rassf3	2.179386	0.25	11.5%
Rassf4	14.39322	0.62	4.3%
Rassf5	23.4453	0.95	4.1%
Rassf6	0.3457208	0.12	35.8%
Rassf7	2.465682	0.12	4.9%
Rassf8	8.212456	0.36	4.4%
Raver1	6.161278	0.60	9.7%
Raver2	2.407724	0.25	10.5%
Rax	0.02279812	0.02	77.1%
Rb1	4.641014	0.72	15.6%
Rb1cc1	11.95192	0.95	8.0%
Rbak	2.196872	0.14	6.5%
Rbbp4	20.17146	1.38	6.9%

Rbbp5	6.44307	0.27	4.2%
Rbbp6	6.767988	0.27	4.1%
Rbbp7	43.15344	4.16	9.6%
Rbbp8	1.506262	0.25	16.5%
Rbbp9	5.551128	0.40	7.2%
Rbck1	16.88834	0.79	4.7%
Rbed1	2.629624	0.15	5.8%
Rbj	35.7882	3.55	9.9%
Rbks	0.5379336	0.14	25.6%
Rbl1	0.5439646	0.13	24.4%
Rbl2	6.29776	0.48	7.6%
Rbm10	3.961742	0.25	6.2%
Rbm11	5.454704	0.55	10.1%
Rbm12	8.466324	0.70	8.3%
Rbm12b	1.211906	0.15	12.3%
Rbm13	8.266294	0.60	7.2%
Rbm14	10.640082	1.31	12.3%
Rbm15	1.59578	0.24	14.8%
Rbm15b	8.09941	0.55	6.8%
Rbm16	15.21638	0.77	5.1%
Rbm17	10.36247	0.48	4.6%
Rbm18	21.50902	0.60	2.8%
Rbm19	3.54817	0.35	10.0%
Rbm20	0.2111846	0.05	25.5%
Rbm22	8.589002	0.72	8.3%
Rbm24	0.7367634	0.04	5.1%
Rbm25	14.56064	1.38	9.5%
Rbm26	22.60756	0.81	3.6%
Rbm27	4.434798	0.42	9.5%
Rbm28	6.30113	0.27	4.2%
Rbm3	8.212106	1.00	12.2%
Rbm34	7.227622	0.43	5.9%
Rbm35a	0.14406426	0.05	32.6%
Rbm35b	0.09767348	0.03	32.4%
Rbm38	0.935044	0.13	13.4%
Rbm39	44.63494	1.21	2.7%
Rbm4	4.153556	0.29	7.1%
Rbm41	1.35054	0.23	17.3%
Rbm43	5.643192	0.31	5.6%
Rbm44	0.00644893	0.01	93.0%
Rbm45	8.28423	0.32	3.8%
Rbm4b	8.695246	0.68	7.8%
Rbm5	31.72028	1.62	5.1%
Rbm6	7.947246	0.33	4.1%
Rbm7	7.519962	0.53	7.0%
Rbm8a	12.80456	1.14	8.9%
Rbm9	74.32918	4.78	6.4%

Rbms1	12.60308	0.85	6.7%
Rbms2	1.755048	0.21	12.1%
Rbms3	15.10758	1.22	8.1%
Rbmx	13.37896	0.70	5.2%
Rbmx2	2.432266	0.21	8.7%
Rbmxl2	0.02246294	0.02	108.3%
Rbmxrt	6.292056	0.74	11.8%
Rbmy1a1	0	0.00	
Rbp1	0.9638986	0.16	16.5%
Rbp2	0.03855556	0.04	104.4%
Rbp3	0.001412234	0.00	223.6%
Rbp4	2.53074	0.47	18.5%
Rbp7	0.02261446	0.03	139.8%
Rbpj	3.222026	0.15	4.6%
Rbpjl	0.0757228	0.17	223.6%
Rbpms	1.566574	0.23	14.7%
Rbpms2	0.5607818	0.11	19.9%
Rbx1	53.70442	1.38	2.6%
Rc3h1	3.637102	0.36	9.9%
Rc3h2	10.640048	0.66	6.2%
Rcan1	16.37806	1.62	9.9%
Rcan2	39.89346	1.65	4.1%
Rcan3	22.14798	1.55	7.0%
Rcbtb1	9.023904	0.72	8.0%
Rcbtb2	9.016712	1.14	12.6%
Rcc1	4.782604	0.23	4.8%
Rcc2	9.691624	0.80	8.2%
Rccd1	2.59563	0.29	11.3%
Rce1	6.409728	0.40	6.3%
Rchy1	12.06004	1.23	10.2%
Rcl1	8.801574	0.51	5.8%
Rcn1	10.841908	0.80	7.4%
Rcn2	42.21152	1.49	3.5%
Rcn3	5.494038	1.41	25.7%
Rcor1	2.780282	0.48	17.3%
Rcor2	2.05864	0.18	8.9%
Rcor3	7.298628	0.94	12.9%
Rcsd1	1.561032	0.25	15.8%
Rcvrn	0.0659175	0.05	78.6%
Rdbp	4.910128	0.64	13.0%
Rdh1	0.02053424	0.02	74.4%
Rdh10	4.369442	0.34	7.7%
Rdh11	19.36132	2.18	11.3%
Rdh12	0.06432094	0.04	57.7%
Rdh13	13.58292	0.23	1.7%
Rdh14	10.9674	0.82	7.5%
Rdh16	0.01394854	0.01	79.5%

Rdh5	0.3657778	0.13	34.4%
Rdh7	0	0.00	
Rdh8	0	0.00	
Rdh9	0.2480976	0.04	17.1%
Rdhe2	0	0.00	
Rdhs	0.01551098	0.02	102.0%
Rdm1	0.4025204	0.05	11.3%
Rdx	34.16618	0.94	2.8%
Rec8	1.2591534	0.20	15.8%
Recc1	0.080861	0.04	46.2%
Reck	1.995278	0.33	16.4%
Recql	4.18211	0.30	7.1%
Recql4	1.2458034	0.32	25.4%
Recql5	3.900736	0.37	9.4%
Reep1	99.06536	3.12	3.2%
Reep2	55.41708	3.03	5.5%
Reep3	5.266746	0.30	5.7%
Reep4	0.9409996	0.07	6.9%
Reep5	240.6368	5.79	2.4%
Reep6	7.912238	0.61	7.7%
Refbp2	3.348132	0.37	11.0%
Reg1	0.00920296	0.02	223.6%
Reg2	0	0.00	
Reg3a	0	0.00	
Reg3d	0	0.00	
Reg3g	0.12926928	0.10	74.6%
Reg4	0.00587124	0.01	223.6%
Rel	0.435749	0.08	18.9%
Rela	7.052568	0.40	5.6%
Relb	1.825818	0.30	16.5%
Rell1	0.9741604	0.11	10.9%
Rell2	65.486	3.79	5.8%
Reln	41.66184	2.13	5.1%
Relt	3.47073	0.77	22.2%
Rem1	0	0.00	
Rem2	1.704712	0.49	28.5%
Ren1	0.00384482	0.01	223.6%
Renbp	2.539482	0.53	20.7%
Repin1	8.13801	0.50	6.1%
Reps1	18.40422	0.82	4.5%
Reps2	13.254688	2.52	19.0%
Rer1	34.08932	2.75	8.1%
Rere	28.67548	1.44	5.0%
Rerg	10.285432	0.72	7.0%
Resp18	27.23242	2.52	9.3%
Rest	0.8785134	0.07	8.4%
Ret	4.278404	0.25	5.8%

Retn	0.0762298	0.04	57.1%
Retnla	0	0.00	
Retnlb	0	0.00	
Retnlg	0	0.00	
Retsat	1.89036	0.24	12.8%
Rev1	5.160224	0.11	2.2%
Rev3l	13.65962	1.00	7.3%
Rex2	0.16444836	0.18	108.7%
Rexo1	6.478902	0.45	7.0%
Rexo2	18.29802	1.01	5.5%
Rexo4	9.050938	0.39	4.4%
Rfc1	6.131528	0.39	6.4%
Rfc2	12.66288	1.35	10.6%
Rfc3	4.433152	0.68	15.4%
Rfc4	2.129208	0.39	18.4%
Rfc5	2.056454	0.20	9.7%
Rfesd	7.798386	0.74	9.5%
Rffl	4.195968	1.05	25.0%
Rfg	0.5433584	0.15	28.0%
Rfk	55.78896	1.92	3.5%
Rfng	23.09874	0.90	3.9%
Rfpl4	0.0048076	0.01	223.6%
Rft1	4.43605	0.28	6.3%
Rftn1	6.388044	0.67	10.5%
Rftn2	0.6588214	0.10	14.5%
Rfwd2	2.0881	0.15	7.3%
Rfwd3	2.584716	0.09	3.5%
Rfx1	3.3839	0.21	6.1%
Rfx2	0.1662894	0.05	27.9%
Rfx3	1.76413	0.16	9.2%
Rfx4	2.307456	0.34	14.9%
Rfx5	5.932802	1.08	18.2%
Rfxank	2.330306	0.84	36.0%
Rfxap	11.52408	0.66	5.8%
Rfxdc1	0.01931306	0.02	86.4%
Rfxdc2	10.51258	0.33	3.1%
Rg9mtd1	6.476108	0.57	8.8%
Rg9mtd2	1.79171	0.35	19.3%
Rg9mtd3	5.615556	0.33	5.9%
Rgag1	1.174682	0.15	13.1%
Rgag4	6.343136	0.38	6.0%
Rgl1	7.581526	0.85	11.2%
Rgl2	8.731624	1.08	12.3%
Rgl3	0.1828452	0.04	24.3%
Rgma	9.169654	0.84	9.2%
Rgmb	25.87474	0.71	2.7%
Rgn	0.04564764	0.02	39.3%

Rgnef	1.3916638	0.34	24.2%
Rgr	0.010949498	0.01	82.7%
Rgs1	0.00457596	0.01	223.6%
Rgs10	14.46398	1.46	10.1%
Rgs11	8.843606	1.68	19.0%
Rgs12	2.392492	0.20	8.3%
Rgs13	0	0.00	
Rgs14	0.00551186	0.01	142.2%
Rgs16	0.6220456	0.17	27.3%
Rgs17	28.92034	3.23	11.2%
Rgs18	0	0.00	
Rgs19	12.11974	1.68	13.8%
Rgs2	21.45434	1.88	8.8%
Rgs20	5.903022	0.77	13.1%
Rgs3	0.7210458	0.09	12.3%
Rgs4	43.9167	2.96	6.7%
Rgs5	0.03698206	0.03	93.7%
Rgs6	4.96359	0.45	9.2%
Rgs7	20.94136	1.59	7.6%
RGS7	0.1666336	0.07	42.6%
Rgs7bp	19.00406	0.26	1.4%
Rgs8	2.932262	0.11	3.8%
Rgs9	1.815	0.27	14.8%
RGS9	0.7868334	0.03	4.1%
Rgs9bp	0.09831132	0.03	29.3%
Rhag	0.00718786	0.01	140.4%
Rhbdd1	3.19691	0.17	5.2%
Rhbdd2	43.10958	3.33	7.7%
Rhbdd3	10.435406	0.66	6.3%
Rhbdf1	4.22693	0.43	10.2%
Rhbdf2	0.427895	0.06	15.0%
Rhbdl1	32.45212	1.74	5.3%
Rhbdl2	0	0.00	
Rhbdl3	3.338818	0.46	13.7%
Rhbg	0.01706992	0.02	103.3%
Rhcg	0.01020908	0.02	159.1%
Rhd	0.03765958	0.03	81.8%
Rheb	57.93172	3.82	6.6%
Rhebl1	2.821686	0.24	8.5%
Rho	0.0655637	0.04	54.2%
Rhoa	28.0812	1.48	5.3%
Rhob	50.39342	2.43	4.8%
Rhobtb1	0.9514242	0.15	16.1%
Rhobtb2	4.465612	0.27	6.0%
Rhobtb3	5.22713	0.27	5.2%
Rhoc	4.952912	0.80	16.1%
Rhod	1.63325	0.28	17.2%

Rhof	13.11634	0.24	1.8%
Rhog	2.865746	0.43	14.9%
Rhoh	0.009923698	0.01	134.3%
Rhoj	1.3818096	0.26	18.7%
Rhoq	12.4593	1.50	12.1%
Rhot1	20.00782	1.04	5.2%
Rhot2	11.36268	0.89	7.8%
Rhou	3.619054	0.12	3.4%
Rhov	10.056356	0.34	3.3%
Rhox1	0	0.00	
Rhox10	0	0.00	
Rhox11	0	0.00	
Rhox12	0	0.00	
Rhox2	0	0.00	
Rhox3	0	0.00	
Rhox4a	0	0.00	
Rhox4b	0	0.00	
Rhox4c	0	0.00	
Rhox4d	0	0.00	
Rhox4e	0	0.00	
Rhox4g	0	0.00	
Rhox4h	0	0.00	
Rhox6	0.0142137	0.02	137.0%
Rhox7	0	0.00	
Rhox8	0.0035358	0.01	223.6%
Rhox9	0.01231516	0.02	138.9%
Rhpn1	1.315964	0.14	10.9%
Rhpn2	0.6464232	0.18	28.3%
Ribc1	0.15179128	0.04	24.0%
Ribc2	0.07614884	0.03	34.8%
Ric3	21.0539	0.48	2.3%
Ric8	14.8692	0.52	3.5%
Ric8b	17.83226	0.55	3.1%
RIE2	3.048444	2.33	76.4%
Rif1	6.052666	0.33	5.4%
Rilp	0.3203944	0.08	25.9%
Rim	0.527996	0.09	17.1%
RIM2	1.989582	0.46	23.3%
Rimbp2	5.896558	0.31	5.2%
Rims1	14.3836	0.76	5.3%
Rims2	14.80154	0.99	6.7%
Rims3	21.9256	1.84	8.4%
Rims4	11.63412	0.78	6.7%
Rin1	0.1981902	0.03	15.5%
Rin2	1.869594	0.14	7.6%
Rin3	0.284937	0.03	10.3%
Ring1	7.509094	0.78	10.4%

Rint1	3.247048	0.09	2.7%
Riok1	6.113776	0.23	3.8%
Riok2	6.779426	0.51	7.6%
Riok3	22.78052	2.15	9.5%
Rip3	0	0.00	
Ripk1	1.321264	0.08	6.2%
Ripk2	1.105913	0.14	12.3%
Ripk3	0.00287324	0.01	223.6%
Ripk4	0.4326892	0.01	3.2%
Ripk5	9.576742	0.82	8.6%
Ripply1	0.03105548	0.02	59.7%
Ripply2	0.872083	0.21	24.5%
Ripply3	0.05303468	0.03	54.7%
Rit1	19.57674	0.59	3.0%
Rit2	29.97002	1.58	5.3%
rjs	12.788	1.11	8.7%
Rkhd3	3.760218	0.35	9.2%
Rlbp1	0.2514032	0.08	33.2%
Rlbp1l1	17.5889	0.25	1.4%
Rlbp1l2	3.509466	0.47	13.3%
Rlf	7.927652	0.52	6.6%
Rln1	0.1308926	0.07	54.6%
Rln3	0	0.00	
Rmi1	4.399862	0.35	7.9%
Rmnd1	2.13389	0.14	6.7%
Rmnd5a	35.72	3.45	9.6%
Rmnd5b	9.231708	0.83	9.0%
Rn3	0.17168782	0.08	47.7%
Rnase1	0.1730162	0.05	29.5%
Rnase10	0.01261966	0.01	93.8%
Rnase11	0	0.00	
Rnase12	0	0.00	
Rnase13	0	0.00	
Rnase4	1.3804234	0.65	47.2%
Rnase6	0	0.00	
Rnase9	0	0.00	
Rnaseh1	2.66811	0.28	10.4%
Rnaseh2a	5.152996	0.11	2.1%
Rnaseh2b	2.916632	0.37	12.7%
Rnaseh2c	11.223122	1.54	13.7%
Rnasel	0.5811472	0.07	12.0%
Rnasen	29.8085	0.41	1.4%
Rnaset2	0.12202432	0.06	45.8%
Rnaset2a	3.897546	0.57	14.5%
Rnaset2b	1.092326	0.44	40.7%
Rnd1	7.9672	0.31	3.9%
Rnd2	43.77942	3.11	7.1%

Rnd3	12.15424	0.43	3.6%
Rnf10	59.13312	3.67	6.2%
Rnf103	18.72692	0.77	4.1%
Rnf11	81.28078	3.01	3.7%
Rnf111	8.120052	0.25	3.0%
Rnf113a1	3.804776	0.69	18.2%
Rnf113a2	5.009428	0.35	7.1%
Rnf12	16.60422	0.87	5.2%
Rnf121	9.344798	0.82	8.8%
Rnf122	1.0103918	0.11	10.8%
Rnf123	7.315358	1.00	13.6%
Rnf125	0.08041226	0.06	75.0%
Rnf126	17.77826	0.78	4.4%
Rnf128	9.154518	0.59	6.4%
Rnf13	27.81612	1.58	5.7%
Rnf130	21.2218	1.38	6.5%
Rnf133	0	0.00	
Rnf135	0.2157508	0.05	21.1%
Rnf138	6.097076	0.69	11.3%
Rnf139	15.65382	0.86	5.5%
Rnf14	149.0016	6.30	4.2%
Rnf141	1.826952	0.46	24.9%
Rnf144a	6.467812	0.49	7.6%
Rnf144b	0.666073	0.13	19.4%
Rnf145	28.78126	3.76	13.1%
Rnf146	23.031	1.47	6.4%
Rnf148	0.0050388	0.01	223.6%
Rnf149	9.060512	0.81	8.9%
Rnf150	3.419002	0.32	9.3%
Rnf151	0.0414141	0.03	82.8%
Rnf152	12.35366	1.16	9.4%
Rnf157	91.502	4.93	5.4%
Rnf166	5.551192	0.48	8.7%
Rnf167	25.35994	0.50	2.0%
Rnf168	7.86464	0.27	3.4%
Rnf17	0.4192648	0.09	22.5%
Rnf170	4.815216	0.35	7.2%
Rnf180	3.938204	0.18	4.7%
Rnf181	26.64512	1.36	5.1%
Rnf182	1.51365	0.22	14.5%
Rnf183	0.05246118	0.04	84.0%
Rnf185	11.73732	0.49	4.1%
Rnf186	0	0.00	
Rnf187	176.4288	13.57	7.7%
Rnf19	0.4863396	0.15	31.7%
Rnf190	0.0902302	0.04	43.8%
Rnf19a	11.53182	0.54	4.7%

Rnf19b	4.12304	0.62	15.0%
Rnf2	15.14614	1.83	12.1%
Rnf20	12.24116	0.69	5.6%
Rnf207	1.30811	0.16	12.4%
Rnf213	0.5026058	0.09	17.2%
Rnf214	21.86028	1.24	5.7%
Rnf215	7.089604	0.10	1.4%
Rnf216	8.408736	0.77	9.2%
Rnf24	8.183888	0.40	4.9%
Rnf25	19.48902	1.12	5.8%
Rnf26	2.88717	0.13	4.6%
Rnf31	6.063274	0.45	7.4%
Rnf32	2.469996	0.31	12.6%
Rnf34	17.80648	0.70	3.9%
Rnf38	10.50226	0.11	1.1%
Rnf39	0.3352968	0.10	29.7%
Rnf4	33.36224	1.71	5.1%
Rnf40	9.378182	0.23	2.5%
Rnf41	21.60348	1.39	6.4%
Rnf43	0.15878082	0.11	70.6%
Rnf44	20.89072	0.83	4.0%
Rnf5	19.96382	2.04	10.2%
Rnf6	13.0531	0.87	6.7%
Rnf7	25.61314	0.82	3.2%
Rnf8	8.369926	0.24	2.9%
Rngtt	6.304148	0.36	5.7%
Rnh1	6.035144	0.68	11.2%
Rnmt	18.6098	0.57	3.1%
Rnmtd2	0.02568728	0.02	95.1%
Rnmtl1	6.172648	0.29	4.7%
Rnpc3	13.95438	2.24	16.0%
Rnpep	8.233324	0.10	1.2%
Rnpepl1	6.041768	0.37	6.2%
Rnps1	8.242526	0.38	4.6%
Rnuxa	18.36986	1.86	10.1%
Robo1	9.3488	0.26	2.8%
Robo2	19.9738	0.93	4.6%
Robo4	0.009710002	0.01	73.3%
Rock1	2.27638	0.15	6.6%
Rock2	10.070706	0.57	5.6%
Rod1	3.202546	0.49	15.2%
Rogdi	40.6897	1.11	2.7%
Rom1	1.4877668	0.38	25.6%
Ropn1	0	0.00	
Ropn1l	0.338974	0.11	33.0%
Ror1	0.5720922	0.11	18.8%
Ror2	1.991984	0.34	17.2%

Rora	3.77553	1.24	32.8%
Rorb	0.9118962	0.08	8.3%
RORb	0.2343432	0.18	78.5%
Rorc	0.522642292	0.33	63.1%
Ros1	0.005021546	0.01	105.1%
Rp1	0	0.00	
Rp1h	0	0.00	
Rp1l1	0.001915984	0.00	223.6%
Rp2	0.14843302	0.10	69.3%
RP23-117C5.5-001	0.00470784	0.01	223.6%
RP23-121P11.3	0.08370722	0.06	70.6%
RP23-153N12.1	2.149196	0.09	4.1%
RP23-180B18.4	0.4800964	0.28	59.1%
RP23-188H3.7	0.022157908	0.02	69.4%
RP23-198E14.4	0.3328248	0.17	52.2%
RP23-257P7.3	1.371274	0.27	20.0%
RP23-286B12.2-001	0.282541	0.11	37.4%
RP23-292E3.5-003	0.5253138	0.17	32.3%
Rp23-297j14.5	0.8507464	0.13	15.0%
RP23-311O20.2-001	0.1454117	0.05	37.6%
RP23-327G1.2	2.488606	0.38	15.4%
Rp23-357i14.1	0.06091862	0.04	61.0%
RP23-382C18.2	0.329812	0.08	24.0%
RP23-391K14.2-001	1.479592	0.22	14.9%
Rp23-423l11.5	0	0.00	
Rp23-438h3.2	0	0.00	
RP23-451A6.3	0.004654112	0.01	150.2%
RP23-453B22.3	2.123078	0.30	14.1%
Rp23-45p4.1	0.008608486	0.00	40.3%
RP23-467J12.2-001	8.865114	0.39	4.4%
RP23-81E16.2-005	3.152078	0.31	9.8%
RP23-92B18.7	0.04638886	0.03	71.0%
Rp2h	4.25709	0.34	7.9%
rp9	13.20234	0.56	4.2%
Rpa1	9.834364	0.58	5.9%
Rpa2	3.77237	0.27	7.0%
Rpa3	2.313984	0.38	16.3%
Rpain	3.639276	0.41	11.2%
Rpap1	4.210604	0.12	2.9%
Rpap2	8.90869	4.20	47.1%
Rpap3	7.67177	0.35	4.6%
Rpe	8.16496	1.22	14.9%
Rpe65	0.00357382	0.01	223.6%
Rpgr	5.425292	0.64	11.7%
Rpgrip1	2.067536	0.60	28.8%
Rpgrip1l	4.782048	0.26	5.5%
Rph3a	15.45678	2.26	14.6%

Rph3al	0.22001076	0.11	52.0%
Rpia	3.563694	0.07	2.1%
Rpl10	1.38592	0.27	19.5%
Rpl10a	5.620914	1.21	21.5%
Rpl11	2.171062	0.41	18.8%
Rpl12	0.7938776	0.14	17.2%
Rpl13	3.881688	0.27	7.1%
Rpl13a	34.83064	3.05	8.7%
Rpl14	19.09304	2.53	13.2%
Rpl15	53.41992	5.08	9.5%
Rpl17	0.440181	0.09	21.4%
Rpl18	5.392466	0.33	6.0%
Rpl18a	7.180916	0.55	7.6%
Rpl19	0.750669	0.08	10.0%
Rpl21	2.39021	0.25	10.4%
Rpl22	24.3227	1.57	6.5%
Rpl22l1	23.00112	1.68	7.3%
Rpl23	5.976512	0.40	6.7%
Rpl23a	0.3324278	0.09	26.6%
Rpl24	0.6365668	0.15	23.4%
Rpl26	9.602684	0.70	7.3%
Rpl27	0.07830254	0.09	115.2%
Rpl27a	14.33054	0.91	6.3%
Rpl28	0.378612	0.16	42.1%
Rpl29	1.470726	0.17	11.6%
Rpl3	0.2558278	0.04	13.7%
Rpl30	2.573766	0.69	26.7%
Rpl31	2.338282	0.43	18.4%
Rpl32	43.00372	5.21	12.1%
Rpl34	1.703582	0.33	19.2%
Rpl35	2.906288	1.18	40.6%
Rpl35a	4.303124	0.40	9.2%
Rpl36	0.9422096	0.37	38.8%
Rpl36a	0.2538348	0.08	32.0%
Rpl36al	16.31218	1.97	12.1%
Rpl37	5.498752	0.60	11.0%
Rpl37a	6.992364	2.87	41.1%
Rpl38	4.98122	0.70	14.1%
Rpl39	26.68136	3.30	12.4%
Rpl39l	0	0.00	
Rpl3l	0.19937882	0.10	51.4%
Rpl4	121.583	9.09	7.5%
Rpl41	56.1053	10.75	19.2%
Rpl5	1.795688	0.19	10.4%
Rpl6	12.04526	1.01	8.3%
Rpl7	24.66364	2.13	8.6%
Rpl7a	13.56926	0.69	5.1%

Rpl7l1	9.575096	0.34	3.6%
Rpl8	208.6176	17.40	8.3%
Rpl9	1.368376	0.30	21.8%
Rplp1	85.22996	3.92	4.6%
Rplp2	11.23733	1.54	13.7%
Rpn1	28.9838	1.62	5.6%
Rpn2	43.70748	1.57	3.6%
Rpo1-1	13.067	0.92	7.0%
Rpo1-2	2.951858	0.08	2.7%
Rpo1-3	13.22054	2.14	16.2%
Rpo1-4	7.030008	0.41	5.8%
Rpp14	10.61492	0.14	1.3%
Rpp21	10.21589	0.99	9.7%
Rpp25	1.900946	0.25	12.9%
Rpp30	12.45834	0.69	5.5%
Rpp38	2.789376	0.39	14.0%
Rpp40	0.9466382	0.13	13.4%
Rprm	9.53467	0.67	7.0%
Rprml	0.1965788	0.03	15.9%
Rps10	2.269166	0.43	19.1%
Rps11	57.86294	6.34	11.0%
Rps12	1.881844	0.13	7.1%
Rps13	0.2486716	0.04	14.8%
Rps14	38.76626	4.35	11.2%
Rps15	92.92412	6.54	7.0%
Rps15a	15.0258	0.53	3.6%
Rps16	13.71908	1.16	8.4%
Rps17	10.372928	0.72	7.0%
Rps18	2.971298	0.46	15.4%
Rps19	12.72494	1.39	10.9%
Rps19bp1	14.65758	1.98	13.5%
Rps2	17.53584	2.94	16.7%
Rps20	40.29178	3.72	9.2%
Rps21	33.22484	2.54	7.6%
Rps23	0.0683421	0.07	96.8%
Rps24	8.74026	0.35	4.0%
Rps25	13.18066	1.42	10.8%
Rps26	22.96854	2.89	12.6%
Rps27	0.09117666	0.09	104.1%
Rps27a	0.937313	0.29	31.3%
Rps27l	8.372212	0.31	3.7%
Rps28	0.3723872	0.07	18.2%
Rps29	0.16770834	0.08	49.2%
Rps3	70.24064	4.86	6.9%
Rps3a	0.475751	0.17	35.9%
Rps4x	44.5026	2.79	6.3%
Rps5	147.5458	11.78	8.0%

Rps6	1.4652448	0.31	21.1%
Rps6ka1	5.602042	0.48	8.5%
Rps6ka2	5.00852	0.39	7.7%
Rps6ka3	9.142708	1.00	11.0%
Rps6ka4	9.272914	0.65	7.0%
Rps6ka5	3.594976	0.71	19.7%
Rps6ka6	12.07652	1.31	10.9%
Rps6kb1	0.9479772	0.21	22.3%
Rps6kb2	7.384294	0.51	6.9%
Rps6kc1	12.26832	0.39	3.2%
Rps6kl1	9.18753	0.53	5.8%
Rps7	0.7685364	0.21	27.1%
Rps9	78.51268	8.93	11.4%
Rpsa	17.78746	1.78	10.0%
Rptn	0	0.00	
Rpusd1	6.834764	0.60	8.8%
Rpusd2	1.26133	0.05	3.8%
Rpusd3	1.818764	0.21	11.3%
Rpusd4	3.184694	0.10	3.1%
Rqcd1	11.29388	0.59	5.2%
Rrad	0.257237	0.08	33.0%
Rraga	61.10846	5.06	8.3%
Rragb	66.9926	1.13	1.7%
Rragc	29.05956	1.60	5.5%
Rragd	15.80606	0.66	4.2%
Rras	2.05442	0.31	15.1%
Rras2	3.356142	0.44	13.2%
Rrbp1	13.569	2.66	19.6%
Rreb1	1.597648	0.13	8.3%
Rrh	0.00532474	0.01	223.6%
Rrm1	7.359186	0.53	7.2%
Rrm2	2.43595	0.21	8.6%
Rrm2b	6.126774	0.15	2.4%
Rrn3	21.91762	0.79	3.6%
Rrp1	101.5556	3.83	3.8%
Rrp12	12.2999	0.97	7.9%
Rrp15	2.738152	0.16	6.0%
Rrp1b	2.662736	0.25	9.3%
Rrp9	6.768102	0.77	11.4%
Rrs1	7.51046	0.64	8.5%
Rs1	0.0035701	0.01	223.6%
Rsad1	1.798484	0.08	4.4%
Rsad2	0.07447086	0.03	34.6%
Rsbn1	7.738332	0.36	4.7%
Rsbn1l	3.782824	0.26	6.9%
Rsf1	5.461636	0.53	9.7%
Rshl1	0.01984022	0.01	36.6%

Rshl2a	3.29308	0.19	5.7%
Rshl2b	2.882022	0.31	10.7%
Rshl3	0.3142932	0.04	11.7%
rsk3	0.04666334	0.03	71.0%
Rsl1	0.6899732	0.13	18.7%
Rsl1d1	23.51344	0.81	3.4%
Rsph1	0.366006	0.05	14.6%
Rspo1	3.227246	0.42	12.9%
Rspo2	1.834858	0.26	14.2%
Rspo3	2.023596	0.12	6.2%
Rspo4	0.00627902	0.01	138.6%
Rspry1	12.01756	0.45	3.7%
Rsrc1	12.76824	0.63	5.0%
Rsrc2	20.13216	0.50	2.5%
Rsu1	14.0716	1.34	9.5%
Rtbdn	1.826858	0.16	8.6%
Rtbnd	0.23761556	0.10	43.4%
Rtcd1	28.56368	0.98	3.4%
Rtdr1	0	0.00	
Rtel1	5.147808	0.26	5.1%
Rtkn	3.034246	0.36	11.8%
Rtl1	2.16664	0.21	9.5%
Rtn1	615.747	31.42	5.1%
Rtn-1A	1.8109414	1.44	79.7%
Rtn2	32.8648	3.37	10.3%
Rtn3	864.7276	16.50	1.9%
Rtn4	172.0262	7.54	4.4%
Rtn4ip1	6.045124	0.40	6.6%
Rtn4r	8.597824	1.19	13.8%
Rtn4rl1	1.58647	0.12	7.5%
Rtn4rl2	1.1423066	0.24	21.4%
Rtp1	0.00824104	0.01	94.5%
Rtp2	0.00536164	0.01	137.3%
Rtp3	0.00281244	0.01	223.6%
Rtp4	0.14186612	0.10	68.6%
Rttn	0.5584908	0.05	8.4%
Rufy1	6.565074	0.53	8.0%
Rufy2	13.1087	0.95	7.2%
Rufy3	64.48826	3.01	4.7%
Rufy4	0.05138164	0.02	42.4%
Rundc1	6.905836	0.40	5.8%
Rundc3a	62.63092	1.62	2.6%
Rundc3b	18.62344	1.22	6.5%
Runx1	0.642523	0.10	15.8%
Runx1t1	9.280292	0.89	9.6%
Runx2	0.1971272	0.05	25.7%
Runx3	2.004024	0.09	4.7%

Rusc1	56.36092	3.29	5.8%
Rusc2	22.59138	1.01	4.5%
Rutbc2	1.3243072	0.39	29.4%
Ruvbl1	2.456244	0.27	11.0%
Ruvbl2	13.31046	0.56	4.2%
Rwdd1	4.264558	0.36	8.5%
Rwdd2a	36.01742	2.22	6.2%
Rwdd2b	3.56061	0.27	7.6%
Rwdd3	1.584216	0.36	22.9%
Rwdd4a	9.515878	0.25	2.6%
Rxfp1	0.4695614	0.17	35.9%
Rxfp2	0.02899676	0.01	51.6%
Rxfp3	0.5665222	0.09	16.0%
Rxfp4	0	0.00	
Rxra	5.757922	0.53	9.3%
Rxrb	15.19032	0.58	3.8%
Rxrg	0.5814602	0.04	6.6%
Rxrip110	0.2850394	0.06	20.6%
Rya3	0	0.00	
Rybp	4.989346	0.48	9.7%
Ryk	4.959342	0.42	8.5%
Ryr1	0.1403334	0.03	19.9%
Ryr2	5.260402	0.47	9.0%
Ryr3	1.596988	0.16	10.2%
Rzf	0.703119	0.14	19.2%
S100a1	1.21646	0.06	5.3%
S100a10	6.688618	1.24	18.6%
S100a11	2.648594	0.43	16.4%
S100a13	1.381886	0.13	9.4%
S100a14	0	0.00	
S100a16	2.919854	0.38	13.0%
S100a3	0	0.00	
S100a4	0.5102704	0.26	50.6%
S100a5	0	0.00	
S100a6	5.02757	1.06	21.0%
S100a7a	0	0.00	
S100a8	0	0.00	
S100a9	0	0.00	
S100b	2.535624	0.46	18.3%
S100g	9.006342	4.77	52.9%
S100pbp	11.40052	0.27	2.4%
S100z	0	0.00	
s11-6	0.3803872	0.18	46.7%
S3-12	0.0319819	0.01	33.8%
S60130	0	0.00	
S60135	0.0520818	0.12	223.6%
S81934	0	0.00	

S90647	0	0.00	
S94460	0	0.00	
Saa1	0.0265879	0.03	94.4%
Saa2	0	0.00	
Saa3	1.850926	0.39	21.1%
Saa4	0	0.00	
Saal1	2.182134	0.14	6.5%
Sac3d1	8.734512	0.48	5.5%
Sacm1l	35.41882	1.21	3.4%
Sacs	3.769044	0.64	17.0%
Sacy	0.01198468	0.01	69.2%
Sae1	47.40476	2.80	5.9%
Sae2	28.10592	1.13	4.0%
Safb2	9.985714	0.46	4.6%
Sag	0.01721386	0.02	92.2%
Sall1	1.28293	0.10	8.1%
Sall2	19.1795	0.63	3.3%
Sall3	1.03017	0.10	10.1%
Sall4	0.179882	0.04	23.1%
SAM11	4.493098	0.85	18.8%
Samd1	5.478518	0.44	8.1%
Samd10	23.4882	3.14	13.4%
Samd12	11.21764	1.13	10.1%
Samd14	33.96732	3.29	9.7%
Samd4	5.015766	0.49	9.7%
Samd4b	10.5197	0.20	1.9%
Samd5	2.881474	0.50	17.5%
Samd7	0.013989884	0.01	103.4%
Samd8	21.8994	0.92	4.2%
Samd9l	0.3352366	0.06	18.7%
Samhd1	4.435424	0.47	10.6%
Samm50	45.83072	1.45	3.2%
Samsn1	0.00951706	0.01	93.8%
Sap130	14.28716	0.75	5.3%
Sap18	8.04825	0.47	5.8%
SAP2	1.3120948	0.29	22.1%
Sap25	0.5308274	0.18	33.1%
Sap30	1.69267	0.15	8.8%
Sap30bp	10.52182	0.40	3.8%
Sap30l	8.75999	0.55	6.3%
Sapap2	0.08411506	0.01	15.6%
Saps1	31.3693	0.66	2.1%
Saps2	24.31128	0.89	3.7%
Saps3	19.72058	0.91	4.6%
Sar1a	52.49034	1.65	3.1%
Sar1b	32.5834	3.76	11.5%
Sardh	0.8538144	0.14	16.4%

Sarm1	5.275458	0.69	13.2%
Sars	110.1176	5.80	5.3%
Sars2	4.650418	0.47	10.1%
Sart1	12.41716	0.52	4.2%
Sart3	7.2843	0.24	3.3%
Sash1	3.436036	0.21	6.1%
SASH1	0.04046452	0.01	25.3%
Sass6	2.07655	0.20	9.6%
Sat1	7.38796	0.48	6.5%
Sat2	2.817312	0.44	15.5%
Satb1	6.802648	0.46	6.8%
Satb2	0.3236708	0.07	21.0%
Satl1	0.00523922	0.01	223.6%
Sav1	4.893236	0.41	8.5%
Sb1	0.5843302	0.08	13.9%
Sbds	11.77406	0.61	5.2%
Sbf1	25.70952	1.01	3.9%
Sbf2	10.263798	1.07	10.4%
Sbk1	25.41102	3.62	14.3%
Sbno1	20.64076	1.13	5.5%
Sbno2	2.536878	0.54	21.3%
Sbp	0	0.00	
Sbpl	0.00802564	0.02	223.6%
Sbsn	3.380406	0.18	5.3%
Sc4mol	78.53948	8.29	10.6%
Sc5d	33.76192	1.02	3.0%
Scaf1	34.48246	1.47	4.3%
Scamp1	91.38928	1.25	1.4%
Scamp2	4.392658	0.35	7.9%
Scamp3	37.45006	2.46	6.6%
Scamp4	11.8911	1.03	8.7%
Scamp5	94.34392	3.83	4.1%
Scand1	7.909762	1.51	19.1%
Scap	16.20284	0.98	6.1%
Scaper	9.275526	0.80	8.6%
Scara3	0.12140112	0.03	26.8%
Scara5	0.414641	0.14	33.7%
Scarb1	9.179324	1.14	12.4%
Scarb2	34.0998	1.65	4.8%
Scarf1	0.04961766	0.02	49.5%
Scarf2	1.3412452	0.57	42.2%
Sccpdh	51.7943	1.92	3.7%
Scd1	45.4453	4.50	9.9%
Scd2	452.876	31.67	7.0%
Scd3	0.3045246	0.02	5.0%
Scd4	1.754982	0.19	10.9%
Scel	0.09268536	0.03	37.5%

Scfd1	18.37216	1.74	9.5%
Scfd2	3.766838	0.19	5.2%
Scg2	84.934	3.65	4.3%
Scg3	50.95674	3.24	6.4%
Scg5	98.99942	3.95	4.0%
Scgb1a1	0	0.00	
Scgb1c1	0	0.00	
Scgb3a1	0.038028	0.09	223.6%
Scgb3a2	0	0.00	
Scgn	0.4710396	0.07	14.1%
Schip1	38.95082	2.19	5.6%
Scin	0.00888088	0.01	92.6%
Sclt1	2.062344	0.39	18.7%
Scly	6.961942	0.59	8.5%
Scmh1	9.502652	0.46	4.8%
scmh1	3.142396	0.49	15.5%
Scml2	0.3047802	0.05	16.1%
Scml4	1.1254752	0.19	16.6%
Scn10a	0.005953226	0.00	62.9%
Scn11a	0.004344158	0.01	157.2%
Scn1a	29.7139	3.05	10.3%
Scn1b	86.58392	4.03	4.7%
Scn2a1	24.8485	1.71	6.9%
Scn2b	60.75688	6.44	10.6%
Scn3a	10.857716	1.40	12.9%
Scn3b	36.61484	3.26	8.9%
Scn4a	0.004225342	0.01	138.7%
Scn4b	8.032002	1.79	22.2%
Scn5a	0.1748108	0.01	4.6%
Scn7a	0.5266142	0.06	12.2%
Scn8a	33.37398	2.61	7.8%
Scn9a	0.8115278	0.06	7.6%
Scnm1	8.063686	0.43	5.4%
Scnn1a	0.2037402	0.06	27.1%
Scnn1b	0.00228912	0.01	223.6%
Scnn1g	0.0026015	0.01	223.6%
Sco1	2.698754	0.27	10.0%
Scoc	35.96666	1.36	3.8%
sco-spondin	0.019034534	0.01	58.7%
Scotin	20.4404	0.88	4.3%
Scp2	10.020678	0.64	6.4%
Scpep1	20.38408	0.83	4.1%
Scrg1	0.606254	0.17	28.4%
Scrib	10.51088	0.29	2.7%
Scrn1	73.32678	1.98	2.7%
Scrn2	0.7134088	0.12	16.6%
Scrn3	10.007374	0.53	5.3%

Scrt1	38.74388	2.82	7.3%
Scs	0.1952822	0.16	80.1%
Sct	0.17103686	0.06	36.8%
Sctr	0.12930778	0.05	41.5%
Scube1	2.453568	0.16	6.5%
Scube2	0.416845	0.11	25.8%
Scube3	0.2216554	0.02	10.2%
Scx	1.264632	0.06	4.8%
Scyb10	0.00282344	0.01	223.6%
Scye1	23.06504	0.89	3.9%
Scyl1	25.3912	0.85	3.3%
Scyl1bp1	4.90652	0.57	11.6%
Scyl2	13.90632	0.86	6.2%
Scyl3	4.385118	0.82	18.8%
Sdad1	8.567086	0.28	3.2%
Sdc1	2.130632	0.23	10.9%
Sdc2	25.66894	1.21	4.7%
Sdc3	47.53952	5.79	12.2%
Sdc4	4.471962	0.63	14.2%
Sdcbp	24.69964	2.10	8.5%
Sdcbp2	0.3180678	0.10	30.7%
Sdccag1	14.1776	0.82	5.8%
Sdccag10	5.326024	0.29	5.5%
Sdccag3	25.07518	0.88	3.5%
Sdccag8	4.42035	0.61	13.7%
Sdf2	29.16304	1.25	4.3%
Sdf2l1	15.43	0.97	6.3%
Sdf4	45.78428	2.71	5.9%
Sdha	125.0716	2.49	2.0%
Sdhb	78.61734	3.92	5.0%
Sdhc	83.55778	3.27	3.9%
Sdhd	79.0766	2.39	3.0%
Sdk1	3.058754	0.20	6.4%
Sdk2	2.870794	0.20	6.8%
Sdpr	1.939726	0.53	27.3%
Sdro	0.00321484	0.01	223.6%
Sds	0.13604562	0.06	47.2%
Sdsl	2.014914	0.24	11.8%
Sebox	0	0.00	
Sec1	0.02753418	0.02	68.6%
Sec11a	22.14854	2.34	10.5%
Sec11c	26.35958	0.80	3.0%
Sec13	22.62624	1.15	5.1%
Sec14l1	6.97929	0.22	3.2%
Sec14l2	2.902806	0.13	4.3%
Sec14l3	0.00342696	0.01	223.6%
Sec14l4	0	0.00	

Sec15l2	0.6221382	0.43	69.0%
Sec16a	10.72874	0.37	3.5%
Sec16b	0.0913422	0.03	38.3%
Sec20	0.556904	0.11	19.4%
Sec22a	3.529302	0.19	5.4%
Sec22b	29.31866	0.45	1.5%
Sec22c	6.099106	0.63	10.3%
Sec23a	41.54462	1.13	2.7%
Sec23b	13.0937	0.38	2.9%
Sec23ip	16.88248	1.14	6.7%
Sec24a	7.122506	0.48	6.8%
Sec24b	14.55704	0.43	2.9%
Sec24c	25.40924	0.82	3.2%
Sec24d	6.200786	0.28	4.5%
Sec31a	2.414654	0.34	14.2%
Sec31b	0.05004572	0.01	18.7%
Sec31l1	51.26798	1.06	2.1%
Sec61a1	42.86256	4.56	10.6%
Sec61a2	44.23348	2.65	6.0%
Sec61b	8.222726	0.94	11.5%
Sec61g	0.2255302	0.17	76.6%
Sec63	12.59124	0.47	3.7%
Secisbp2	3.050162	0.22	7.2%
Sectm1a	0.01514468	0.02	153.2%
Sectm1b	0	0.00	
Sef	0.02398354	0.01	31.5%
Seh1l	14.61818	0.43	3.0%
Sel1h	0.6528978	0.30	45.6%
Sel1l	33.68884	1.65	4.9%
Sel1l2	0	0.00	
Sele	0.00220246	0.00	223.6%
Selenbp1	0.4754826	0.15	30.5%
Selenbp2	0.09424008	0.10	105.3%
Selk	34.64794	2.93	8.4%
Sell	0	0.00	
Selm	55.07076	3.67	6.7%
Selp	0.00270662	0.01	223.6%
Selpl	0.001387556	0.00	223.6%
Selplg	0.00154574	0.00	223.6%
Sema3a	5.228714	0.50	9.5%
Sema3b	0.21167	0.05	23.0%
Sema3c	3.329864	0.26	7.7%
Sema3d	0.6434898	0.08	11.9%
Sema3e	1.314458	0.03	2.5%
Sema3f	2.259454	0.17	7.7%
Sema3g	0.0562816	0.02	34.1%
Sema4a	8.52321	0.96	11.3%

Sema4b	3.761986	0.30	8.0%
Sema4c	2.718238	0.15	5.4%
Sema4d	7.845624	0.36	4.6%
Sema4f	8.173662	0.44	5.4%
Sema4g	27.25826	0.84	3.1%
Sema5a	31.85062	2.20	6.9%
Sema5b	3.034466	0.22	7.2%
Sema6a	12.18062	0.56	4.6%
Sema6b	20.37368	0.49	2.4%
Sema6c	7.827888	0.64	8.2%
Sema6d	25.39422	0.80	3.2%
Sema7a	11.25232	0.83	7.3%
Semcap2	0.04923498	0.02	35.7%
Semcap3	0.038945	0.01	16.0%
Senp1	4.31942	0.28	6.5%
Senp2	20.4263	0.41	2.0%
Senp3	14.02234	1.11	7.9%
Senp5	12.89744	0.77	5.9%
Senp6	14.36144	0.64	4.5%
Senp7	13.8341	2.16	15.6%
Senp8	4.29316	0.41	9.6%
Sephs1	8.805182	0.67	7.6%
Sephs2	18.4902	0.66	3.6%
Sepn1	6.950868	0.60	8.6%
Sepp1	48.77016	4.35	8.9%
Sepsecs	2.211934	0.39	17.6%
Sepw1	171.818	27.01	15.7%
Sepx1	14.27308	1.87	13.1%
Serac1	4.518646	0.86	19.0%
serase-1B	0.04957128	0.02	48.6%
Serbp1	57.59702	4.34	7.5%
Serf1	12.64568	0.89	7.0%
Serf2	15.41192	1.35	8.7%
Sergef	7.06202	0.39	5.5%
Serhl	6.636936	1.16	17.5%
Serinc1	358.815	10.43	2.9%
Serinc2	0.2346418	0.03	13.3%
Serinc3	82.28818	1.07	1.3%
Serinc5	8.616396	0.23	2.7%
Serpina10	0.01105408	0.01	93.9%
Serpina11	0	0.00	
Serpina12	0.001652184	0.00	223.6%
Serpina1a	0.00421776	0.01	223.6%
Serpina1b	0	0.00	
Serpina1c	0	0.00	
Serpina1d	0	0.00	
Serpina1e	0	0.00	

Serpina1f	0	0.00	
Serpina3a	0.00593882	0.01	137.3%
Serpina3b	0	0.00	
Serpina3c	0.4409588	0.12	27.4%
Serpina3f	0.8925866	0.17	18.8%
Serpina3g	0.2320222	0.05	20.8%
Serpina3h	0.1567086	0.03	21.8%
Serpina3k	0.06280456	0.04	56.7%
Serpina3m	0.2293918	0.09	40.6%
Serpina3n	20.7181	1.92	9.3%
Serpina4-ps1	0	0.00	
Serpina5	0	0.00	
Serpina6	0.0051868	0.01	223.6%
Serpina7	0	0.00	
Serpina9	0.08527262	0.01	15.2%
Serpinb10	0	0.00	
Serpinb11	0	0.00	
Serpinb12	0.00721344	0.02	223.6%
Serpinb13	0	0.00	
Serpinb1a	0.2699046	0.04	16.2%
Serpinb1b	13.59534	2.05	15.1%
Serpinb1c	0.10042198	0.06	63.7%
Serpinb2	0.021179	0.02	100.9%
Serpinb3a	0.01048192	0.02	223.6%
Serpinb3b	0	0.00	
Serpinb3c	0	0.00	
Serpinb3d	0	0.00	
Serpinb5	0.04350324	0.02	57.4%
Serpinb6a	8.10331	0.47	5.8%
Serpinb6b	0.1889258	0.07	36.6%
Serpinb6c	0.1748908	0.05	26.6%
Serpinb7	0.0037393	0.01	223.6%
Serpinb8	0.07076986	0.04	55.8%
Serpinb9	3.59859	0.41	11.3%
Serpinb9b	0.15258828	0.05	31.4%
Serpinb9c	0.039645	0.03	63.6%
Serpinb9d	0	0.00	
Serpinb9e	0.02430022	0.02	91.0%
Serpinb9f	0	0.00	
Serpinb9g	0	0.00	
Serpinc1	0.00245736	0.01	223.6%
Serpind1	0.00801608	0.01	144.4%
Serpine1	0.04610028	0.05	108.8%
Serpine2	131.9286	10.33	7.8%
Serpinf1	9.782934	0.74	7.6%
Serpinf2	0.00220528	0.00	223.6%
Serping1	1.93935	0.58	29.7%

Serpinh1	26.04582	5.28	20.3%
Serpini1	154.8006	3.71	2.4%
Serpini2	0.00428202	0.01	223.6%
SerRSmt	0.406847	0.04	11.0%
Sertad1	0.8716214	0.11	12.6%
Sertad2	2.583868	0.14	5.5%
Sertad3	0.6250848	0.05	8.1%
Sertad4	6.729202	0.50	7.5%
Sesn1	11.79848	0.89	7.6%
Sesn2	19.66428	0.84	4.3%
Sesn3	20.4794	2.02	9.9%
Sestd1	6.554886	0.60	9.1%
Set	19.02396	1.68	8.8%
Setbp1	2.148468	0.30	13.8%
Setd1a	6.697804	0.53	7.9%
Setd1b	3.096588	0.11	3.7%
Setd2	11.89742	0.43	3.6%
Setd3	19.33892	0.51	2.7%
Setd4	1.537768	0.17	11.0%
Setd5	19.01402	0.37	2.0%
Setd6	3.075562	0.61	20.0%
Setd7	4.306074	0.47	11.0%
Setd8	8.415378	0.63	7.5%
Setdb1	10.41978	0.11	1.1%
Setdb2	0.6888016	0.05	6.6%
Setmar	1.766674	0.16	9.2%
Setx	11.2068	0.46	4.1%
Sez4	0.5524124	0.13	24.0%
Sez6	21.69768	1.85	8.5%
Sez6l	25.32596	0.99	3.9%
Sez6l2	64.14068	2.59	4.0%
Sf1	46.2581	1.00	2.2%
Sf3a1	18.88816	0.81	4.3%
Sf3a2	13.12924	0.96	7.3%
Sf3a3	8.323288	0.62	7.5%
Sf3b1	48.28922	1.99	4.1%
Sf3b2	78.65164	1.37	1.7%
Sf3b3	20.36326	1.07	5.3%
Sf3b4	7.603918	0.73	9.6%
Sf3b5	14.48352	1.06	7.3%
Sf4	12.54514	0.64	5.1%
Sfi1	33.5675	2.76	8.2%
Sfmbt1	5.55896	0.51	9.2%
Sfmbt2	6.106002	1.30	21.3%
Sfn	0.2727544	0.06	21.6%
Sfpi1	0	0.00	
Sfpq	39.9992	4.07	10.2%

Sfrp1	9.59658	0.60	6.2%
Sfrp2	3.908704	0.35	9.0%
Sfrp4	0.4296566	0.04	9.4%
Sfrp5	0.07568734	0.02	30.6%
Sfrs1	36.52548	0.91	2.5%
Sfrs10	9.544482	0.38	4.0%
Sfrs11	44.2133	4.11	9.3%
Sfrs12	12.05242	0.99	8.2%
Sfrs14	30.31724	1.49	4.9%
Sfrs15	8.710244	0.56	6.4%
Sfrs16	2.833344	0.84	29.7%
Sfrs17b	6.250554	0.22	3.5%
Sfrs2	43.0481	3.08	7.2%
Sfrs2ip	10.390788	0.51	4.9%
Sfrs3	22.05554	0.78	3.5%
Sfrs4	13.68392	1.22	8.9%
Sfrs5	29.30978	0.71	2.4%
Sfrs6	31.89954	0.50	1.6%
Sfrs7	29.87606	2.09	7.0%
Sfrs8	13.78152	0.46	3.3%
Sfrs9	11.25818	0.70	6.2%
Sft2d1	1.9233776	1.21	62.9%
Sft2d2	1.452096	0.17	12.0%
Sft2d3	1.544646	0.17	11.2%
Sftpa1	0.00767948	0.01	91.8%
Sftpb	0.00721924	0.01	139.8%
Sftpc	0.02989488	0.03	99.5%
Sftpd	0.0133627	0.01	99.8%
Sfxn1	25.59938	1.37	5.3%
Sfxn2	1.536818	0.21	13.4%
Sfxn3	41.77814	2.02	4.8%
Sfxn4	7.526214	0.24	3.2%
Sfxn5	34.71516	3.62	10.4%
Sgca	0.05123548	0.04	78.5%
Sgcb	29.98362	1.81	6.0%
Sgcd	1.2325724	0.40	32.1%
Sgce	12.61142	0.81	6.4%
Sgcg	0.04448172	0.01	32.1%
Sgcz	1.294466	0.15	12.0%
sgigsf	0.0577442	0.04	71.0%
Sgip1	36.5596	4.27	11.7%
SGIP1alpha	8.569484	4.22	49.3%
Sgk	5.465068	0.46	8.4%
Sgk2	0.0512171	0.03	53.1%
Sgk269	2.943648	0.30	10.3%
Sgk3	0.4740794	0.09	18.0%
Sgms1	8.091782	0.23	2.8%

Sgms2	0.4020214	0.11	27.6%
Sgo1	0.1211182	0.27	223.6%
Sgol1	0.2595978	0.10	38.5%
Sgol2	0.5613734	0.06	10.9%
Sgpl1	8.536728	0.30	3.5%
Sgpp1	13.07184	0.88	6.8%
Sgpp2	3.126964	1.03	33.0%
Sgsh	0.8838504	0.11	12.2%
Sgsm1	18.24102	1.58	8.7%
Sgsm2	17.96586	1.14	6.4%
Sgsm3	20.44942	0.50	2.5%
Sgta	54.56468	4.36	8.0%
Sgtb	24.24634	1.08	4.5%
Sh2b1	11.088708	1.51	13.6%
Sh2b2	3.327704	0.26	7.7%
Sh2b3	2.591186	0.34	13.2%
Sh2bpsm1	7.919538	1.58	19.9%
Sh2d1a	0.0058008	0.01	223.6%
Sh2d1b1	0.01493224	0.01	93.8%
Sh2d1b2	0	0.00	
Sh2d2a	0	0.00	
Sh2d3c	10.410812	0.74	7.1%
Sh2d4a	0.09798658	0.02	21.7%
Sh2d4b	0.0472029	0.02	49.2%
Sh2d5	9.84012	0.56	5.7%
Sh3bgr	1.479408	0.24	16.2%
Sh3bgrl	38.1769	1.54	4.0%
Sh3bgrl2	12.46872	1.41	11.3%
Sh3bgrl3	55.65294	6.98	12.5%
Sh3bp1	2.739704	0.59	21.7%
Sh3bp2	1.596522	0.32	20.0%
Sh3bp4	8.188316	0.34	4.1%
Sh3bp5	17.5408	2.18	12.4%
Sh3bp5l	17.3423	0.25	1.5%
Sh3d19	4.510386	0.45	10.0%
SH3d19	1.479318	0.43	29.1%
Sh3gl1	10.61792	0.85	8.0%
Sh3gl2	82.0416	2.43	3.0%
Sh3gl3	11.85902	0.30	2.5%
Sh3glb1	11.48138	1.16	10.1%
Sh3glb2	41.53588	2.17	5.2%
Sh3kbp1	12.12684	0.68	5.6%
Sh3md4	2.852928	0.29	10.0%
Sh3px3	0.8701394	0.19	22.0%
Sh3pxd2a	4.965126	0.73	14.8%
Sh3pxd2b	3.715502	0.18	4.8%
Sh3rf1	5.957548	0.38	6.5%

Sh3rf2	0.001702764	0.00	223.6%
Sh3tc1	0.02574324	0.01	25.9%
Sh3tc2	0.08243042	0.06	70.1%
Sh3yl1	5.637996	0.57	10.1%
Shag1	0.0783712	0.02	29.2%
Shank2	5.891272	0.23	4.0%
Shank3	17.05286	0.43	2.5%
Sharpin	13.88148	0.74	5.3%
Shb	4.579842	0.60	13.1%
Shbg	0	0.00	
Shc1	2.98758	0.38	12.6%
Shc2	10.111196	0.60	5.9%
Shc3	8.281078	0.97	11.8%
Shc4	1.0410532	0.15	14.5%
Shcbp1	1.0081914	0.16	15.4%
Shd	15.04402	1.42	9.5%
She	0.19308	0.05	27.6%
Shf	7.624726	0.50	6.5%
Shfm1	49.58576	2.03	4.1%
Shh	1.53634	0.20	12.8%
Ship	0.005240088	0.00	73.3%
Shkbp1	2.469766	0.47	19.0%
Shmt1	0.645802	0.14	21.4%
Shmt2	8.88795	1.08	12.2%
Shoc2	38.25654	2.43	6.4%
Shoca-1	0.02248308	0.02	68.3%
Shox2	1.383598	0.23	16.7%
Shprh	10.349024	0.47	4.6%
Shq1	1.401206	0.06	4.1%
Shroom1	0.09276558	0.01	11.3%
Shroom2	57.88776	4.13	7.1%
Shroom3	0.7676732	0.07	8.6%
Shroom4	0.1562488	0.01	9.4%
Shyc	0.2493546	0.06	25.5%
Si	0.08745846	0.03	33.6%
Siae	7.002494	0.42	6.0%
Siah1a	13.02862	0.81	6.2%
Siah1b	2.44034	0.25	10.1%
Siah2	6.686924	0.48	7.2%
sid2895	0.0711864	0.05	70.1%
Sid470	1.53669	0.67	43.4%
Sidt1	6.104522	0.21	3.5%
Sidt2	23.71546	0.51	2.2%
Sigirr	0.02232038	0.02	79.3%
Siglec1	0	0.00	
Siglec15	0	0.00	
Siglec5	0.004640482	0.01	151.9%

Siglece	0	0.00	
Siglecf	0.000879104	0.00	223.6%
Siglecg	0.0050447	0.01	138.1%
Siglech	0	0.00	
SIK2	1.706714	0.76	44.4%
Sil1	8.562894	0.61	7.1%
Sim1	0.002400478	0.00	140.5%
Sim2	0.03402238	0.01	40.1%
Sin3a	5.735026	0.26	4.5%
Sin3b	28.88548	1.89	6.5%
Sip1	2.018982	0.10	4.8%
Sipa1	0.9416612	0.07	7.6%
Sipa1l1	7.142004	0.50	6.9%
Sipa1l2	9.548048	0.68	7.2%
Sipa1l3	6.048478	0.23	3.8%
Sirpa	25.89752	1.89	7.3%
Sirpb1	0.00971616	0.01	138.6%
SIRP-beta	0	0.00	
Sirt1	2.638522	0.13	5.0%
Sirt2	23.19174	0.71	3.1%
Sirt3	25.79026	1.39	5.4%
Sirt4	4.36588	0.46	10.6%
Sirt5	3.509098	0.16	4.6%
Sirt6	9.454932	0.77	8.1%
Sirt7	7.005908	0.40	5.7%
Sit1	0	0.00	
Siva	0.53134	0.10	19.2%
Siva1	1.962108	0.49	24.8%
Six1	0.5541982	0.07	12.1%
Six2	0.2096024	0.04	20.1%
Six3	0.08210076	0.04	49.5%
Six4	0.272546	0.06	22.8%
Six5	0.479012	0.13	26.4%
Six6	0.1211056	0.02	15.2%
Six6os1	1.38505	0.18	13.3%
Skap1	0.18035634	0.07	36.4%
Skap2	7.857358	0.34	4.4%
Ski	11.95538	0.70	5.8%
Skil	12.0096	0.35	2.9%
Skint1	0	0.00	
Skint10	0	0.00	
Skint5	0.00207872	0.00	223.6%
Skint6	0	0.00	
Skint7	0.0123931	0.02	138.6%
Skiv2l	11.27462	0.49	4.4%
Skiv2l2	19.5747	0.91	4.6%
Skp1a	175.1768	4.27	2.4%

Skp2	2.15107	0.16	7.4%
SKRP1	1.537962	0.24	15.3%
Sla	1.19615	0.11	9.0%
Sla2	0.36807432	0.48	130.4%
Slain1	4.51493	0.32	7.0%
Slain2	5.815384	0.34	5.9%
Slamf1	0.00303452	0.01	223.6%
Slamf6	0	0.00	
Slamf7	0	0.00	
Slamf8	0	0.00	
Slamf9	0.0581654	0.03	56.4%
Slbp	12.71808	0.38	3.0%
Slc10a1	0.05178984	0.02	43.2%
Slc10a2	0	0.00	
Slc10a3	1.499548	0.10	7.0%
Slc10a4	0.0591908	0.03	47.5%
Slc10a5	0.04068452	0.01	32.3%
Slc10a6	0.0136661	0.02	116.7%
Slc10a7	1.916072	0.24	12.3%
Slc11a1	0.04012622	0.03	63.9%
Slc11a2	12.88548	0.21	1.6%
Slc12a1	0.04020036	0.02	50.0%
Slc12a2	5.433442	0.16	3.0%
Slc12a3	0.01617516	0.01	70.4%
Slc12a4	2.165908	0.37	17.0%
Slc12a5	98.30882	4.32	4.4%
Slc12a6	19.03412	1.39	7.3%
Slc12a7	1.229522	0.08	6.8%
Slc12a8	0.35608	0.08	21.8%
Slc12a9	7.561264	0.39	5.2%
Slc13a1	0	0.00	
Slc13a2	0	0.00	
Slc13a3	0.2173744	0.03	12.9%
Slc13a4	0.0131529	0.02	139.1%
Slc13a5	0.10761916	0.07	63.4%
Slc14a1	0.4527194	0.10	22.2%
Slc14a2	0.6853846	0.04	6.4%
Slc15a1	0.01152024	0.01	119.4%
Slc15a2	2.131466	0.21	9.7%
Slc15a3	0.4447852	0.51	115.1%
Slc15a4	7.50346	0.41	5.4%
Slc16a1	9.368726	0.59	6.3%
Slc16a10	0.368455	0.05	13.3%
Slc16a11	0.8899376	0.17	19.1%
Slc16a12	0.5347006	0.10	18.2%
Slc16a13	3.112066	0.17	5.3%
Slc16a14	4.363236	0.45	10.3%

Slc16a2	4.80443	0.20	4.1%
Slc16a3	13.785166	3.40	24.6%
Slc16a4	0.15392636	0.06	41.9%
Slc16a5	0.1684916	0.04	23.6%
Slc16a6	4.335214	1.94	44.8%
Slc16a7	5.79362	0.55	9.5%
Slc16a8	0.14696112	0.06	41.3%
Slc16a9	1.82587	0.26	14.5%
Slc17a1	0.0045395	0.01	223.6%
Slc17a2	0.00261706	0.01	223.6%
Slc17a3	0.00530208	0.01	137.0%
Slc17a4	0.00285446	0.01	223.6%
Slc17a5	4.316486	0.62	14.5%
Slc17a6	100.8006	3.29	3.3%
Slc17a7	0.13309144	0.03	24.8%
Slc17a8	0.11618276	0.04	34.7%
Slc18a1	0.2486644	0.06	22.9%
Slc18a2	1.837598	0.19	10.6%
Slc18a3	.11136772	0.20	17.7%
Slc19a1	6.817478	0.31	4.5%
Slc19a2	8.552812	0.21	2.5%
Slc19a3	0.10208024	0.08	74.9%
Slc1a1	28.22234	0.80	2.8%
Slc1a2	15.21236	1.87	12.3%
Slc1a3	27.60352	2.59	9.4%
Slc1a4	33.21738	2.21	6.7%
Slc1a5	4.01419	0.50	12.5%
Slc1a6	14.83118	1.00	6.7%
Slc1a7	0	0.00	
Slc20a1	40.46036	0.65	1.6%
Slc20a2	12.29564	0.42	3.4%
Slc21a7	0.03790398	0.02	55.1%
Slc22a1	0.00809182	0.02	223.6%
Slc22a12	0.03778912	0.01	16.1%
Slc22a13	0.03012802	0.03	94.3%
Slc22a14	0.01791736	0.02	117.6%
Slc22a15	1.2422554	0.92	74.3%
Slc22a16	0	0.00	
Slc22a17	109.1208	5.09	4.7%
Slc22a18	0.1810446	0.12	67.8%
Slc22a2	0.00310658	0.01	223.6%
Slc22a20	0	0.00	
Slc22a21	1.274554	0.17	13.3%
Slc22a3	0.1521016	0.03	20.3%
Slc22a4	0.4686322	0.09	18.4%
Slc22a5	2.5238	0.18	7.3%
Slc22a6	0.00343378	0.01	223.6%

Slc22a7	0.03953986	0.02	52.3%
Slc22a8	0.00494134	0.01	137.0%
Slc22a9	0	0.00	
Slc23a1	0.01927442	0.01	65.8%
Slc23a2	22.32812	1.26	5.7%
Slc23a3	0.00707444	0.01	138.4%
Slc24a1	0.001429818	0.00	223.6%
Slc24a2	22.51468	1.93	8.6%
Slc24a3	57.52554	2.13	3.7%
Slc24a4	1.1934586	0.14	12.1%
Slc24a5	0.451272	0.17	36.6%
Slc24a6	0.2312102	0.04	15.4%
Slc25a1	45.51886	2.55	5.6%
Slc25a10	5.050398	0.38	7.5%
Slc25a11	68.6164	1.23	1.8%
Slc25a12	68.11988	0.99	1.5%
Slc25a13	0.8154248	0.13	15.6%
Slc25a14	14.13176	0.45	3.2%
Slc25a15	3.31774	0.11	3.3%
Slc25a16	9.813074	0.65	6.6%
Slc25a17	23.24848	1.96	8.4%
Slc25a18	2.664706	0.27	10.1%
Slc25a19	8.55165	0.65	7.6%
Slc25a2	0.20835418	0.07	36.0%
Slc25a20	9.450778	0.65	6.9%
Slc25a21	0.06797092	0.03	37.4%
Slc25a22	33.87524	1.48	4.4%
Slc25a23	52.0449	3.20	6.1%
Slc25a24	1.0088124	0.10	10.0%
Slc25a25	18.7282	0.92	4.9%
Slc25a26	3.650614	0.30	8.2%
Slc25a27	11.35424	0.56	4.9%
Slc25a28	11.8438	0.54	4.5%
Slc25a29	3.439206	0.13	3.8%
Slc25a3	216.9346	5.48	2.5%
Slc25a30	2.150112	0.33	15.2%
Slc25a31	0.06477354	0.02	36.5%
Slc25a32	4.992884	0.65	13.1%
Slc25a33	16.28822	0.69	4.2%
Slc25a34	0.08404336	0.04	43.0%
Slc25a35	1.924886	0.38	19.6%
Slc25a36	14.40818	0.64	4.5%
Slc25a37	5.64791	1.05	18.5%
Slc25a38	12.26042	0.28	2.3%
Slc25a39	49.86926	2.19	4.4%
Slc25a4	485.6796	20.64	4.2%
Slc25a40	2.558716	0.89	34.7%

Slc25a41	0.374495	0.03	7.8%
Slc25a42	4.664064	0.80	17.2%
Slc25a43	0.00975844	0.01	137.0%
Slc25a44	28.93902	1.02	3.5%
Slc25a45	0.14246396	0.09	63.7%
Slc25a46	33.55222	0.99	2.9%
Slc25a5	89.92608	4.86	5.4%
Slc26a1	1.285328	0.17	13.2%
Slc26a11	0.8362924	0.09	10.3%
Slc26a2	1.491272	0.08	5.6%
Slc26a3	0.0059442	0.01	140.6%
Slc26a4	0.0071326	0.01	94.1%
Slc26a5	0.02527446	0.01	25.0%
Slc26a6	4.815716	0.20	4.1%
Slc26a7	0.06365958	0.05	71.8%
Slc26a8	0.5866196	0.09	15.3%
Slc26a9	0.05149594	0.03	50.9%
Slc27a1	9.136008	0.62	6.8%
Slc27a2	3.833168	0.24	6.3%
Slc27a3	4.424466	0.30	6.7%
Slc27a4	52.71188	2.70	5.1%
Slc27a5	0.11884436	0.03	29.3%
Slc27a6	0.14495052	0.04	27.7%
Slc28a1	0.0262064	0.02	84.4%
Slc28a2	0.16479274	0.06	35.9%
Slc28a3	0	0.00	
Slc29a1	2.28911	0.23	10.2%
Slc29a2	14.6404	1.19	8.2%
Slc29a3	1.925098	0.13	6.8%
Slc29a4	12.8746	0.65	5.0%
Slc2a1	7.191206	1.52	21.1%
Slc2a10	0.06798004	0.03	46.3%
Slc2a12	0.2089918	0.04	19.2%
Slc2a13	13.83864	0.79	5.7%
Slc2a2	0.01598786	0.02	95.9%
Slc2a3	61.9302	2.93	4.7%
Slc2a4	0.1792968	0.04	24.6%
Slc2a5	0.01268772	0.01	105.8%
Slc2a6	16.91776	0.67	4.0%
Slc2a8	3.759872	0.73	19.4%
Slc2a9	0.13017336	0.03	26.6%
Slc30a1	5.90243	0.22	3.8%
Slc30a10	2.259614	0.19	8.4%
Slc30a2	0.04842134	0.03	63.3%
Slc30a3	4.612816	0.59	12.7%
Slc30a4	9.321496	0.76	8.2%
Slc30a5	16.40474	0.49	3.0%

Slc30a6	4.39576	0.31	7.0%
Slc30a7	3.112688	0.25	7.9%
Slc30a8	0.01920038	0.01	61.7%
Slc30a9	51.71028	0.95	1.8%
Slc31a1	16.25862	1.43	8.8%
Slc31a2	2.41473	0.30	12.5%
Slc32a1	11.57476	1.34	6.2%
Slc33a1	12.94528	0.73	5.6%
Slc34a1	0.00460192	0.01	223.6%
Slc34a2	0.00588628	0.01	99.6%
Slc34a3	0	0.00	
Slc35a1	5.90763	0.30	5.0%
Slc35a2	12.12992	0.77	6.4%
Slc35a3	5.9028	0.35	6.0%
Slc35a4	9.545976	0.48	5.0%
Slc35a5	9.755968	0.37	3.8%
Slc35b1	31.42848	1.72	5.5%
Slc35b2	11.43648	0.91	8.0%
Slc35b3	3.564454	0.45	12.7%
Slc35b4	15.69358	0.75	4.8%
Slc35c1	6.141746	0.37	6.0%
Slc35c2	15.88572	1.02	6.4%
Slc35d1	2.237402	0.21	9.6%
Slc35d2	0.0526234	0.01	25.5%
Slc35d3	0.7947252	0.09	10.9%
Slc35e1	19.20186	0.88	4.6%
Slc35e3	6.837384	0.46	6.7%
Slc35e4	7.49716	0.69	9.2%
Slc35f1	31.87654	1.51	4.7%
Slc35f2	2.018568	0.40	19.7%
Slc35f3	9.221624	0.30	3.2%
Slc35f4	4.432036	0.40	9.0%
Slc35f5	10.94638	0.45	4.1%
Slc36a1	20.04682	1.13	5.7%
Slc36a2	0.00622832	0.01	138.8%
Slc36a3	0	0.00	
Slc36a4	20.87088	1.32	6.3%
Slc37a1	0.947053	0.03	3.1%
Slc37a2	0.4789678	0.07	14.9%
Slc37a3	14.14686	2.62	18.5%
Slc37a4	8.757724	0.30	3.4%
Slc38a1	63.2884	3.64	5.8%
Slc38a2	32.81884	2.08	6.3%
Slc38a3	2.881952	0.68	23.8%
Slc38a4	0.9335442	0.17	17.8%
Slc38a5	0.02582388	0.02	66.9%
Slc38a6	0.232719	0.09	36.9%

Slc39a1	9.1244	1.27	14.0%
Slc39a10	17.90802	1.07	6.0%
Slc39a11	5.936796	0.72	12.2%
Slc39a12	3.772586	0.15	4.1%
Slc39a13	6.336878	0.22	3.5%
Slc39a14	6.397716	0.17	2.7%
Slc39a2	0.7864096	0.18	22.9%
Slc39a3	19.7388	0.60	3.0%
Slc39a4	0.10224082	0.07	67.8%
Slc39a5	0.00582732	0.01	137.1%
Slc39a6	26.70224	1.65	6.2%
Slc39a7	27.26762	1.39	5.1%
Slc39a8	0.337836	0.14	42.1%
Slc39a9	16.85554	0.74	4.4%
Slc3a1	0.200997	0.03	14.8%
Slc3a2	42.4674	2.67	6.3%
Slc40a1	4.527822	0.77	17.1%
Slc41a1	6.173932	0.59	9.5%
Slc41a2	9.648398	0.78	8.1%
Slc41a3	3.994666	0.28	7.1%
Slc43a1	0.4166782	0.09	21.5%
Slc43a2	14.86946	1.04	7.0%
slc43a2	0.3555758	0.05	12.9%
Slc43a3	0.14181238	0.06	44.9%
Slc44a1	11.053356	0.76	6.9%
Slc44a2	12.89884	1.01	7.9%
Slc44a3	0.241093	0.09	38.0%
Slc44a4	0.11609768	0.03	27.5%
Slc44a5	6.04359	0.72	12.0%
Slc45a1	26.01292	1.78	6.8%
Slc45a2	0.06583014	0.03	45.6%
Slc45a3	0.2325786	0.09	38.4%
Slc45a4	16.69028	1.75	10.5%
Slc46a1	5.008762	0.44	8.8%
Slc46a2	0.00222808	0.00	223.6%
Slc46a3	1.1009604	0.24	21.4%
Slc47a1	0.004707852	0.01	149.8%
Slc47a2	0.0125441	0.01	59.4%
Slc4a1	0.003030684	0.00	140.9%
Slc4a10	17.61902	0.98	5.6%
Slc4a11	0.1751148	0.03	17.6%
Slc4a1ap	10.93282	0.38	3.5%
Slc4a2	5.648694	0.44	7.8%
Slc4a3	32.4103	0.72	2.2%
Slc4a4	11.47568	0.74	6.5%
Slc4a7	7.764244	0.27	3.5%
Slc4a8	13.80796	0.70	5.0%

Slc4a9	0.021319548	0.02	87.0%
Slc5a1	0.009280942	0.01	111.4%
Slc5a10	0.1187821	0.09	78.2%
Slc5a11	0.00598004	0.01	137.8%
Slc5a12	0.00904368	0.01	97.4%
Slc5a2	0.02777494	0.01	25.3%
Slc5a3	4.206	0.36	8.6%
Slc5a4a	0.00347626	0.01	223.6%
Slc5a4b	0.0033999	0.01	223.6%
Slc5a5	0.10416388	0.03	31.0%
Slc5a6	6.289086	0.45	7.1%
Slc5a7	0.4323396	0.10	22.4%
Slc5a8	0.03302666	0.02	59.4%
Slc5a9	0.01548445	0.01	80.8%
Slc6a1	29.97576	1.93	6.4%
Slc6a11	18.27442	1.20	6.6%
Slc6a12	0.02873704	0.02	58.5%
Slc6a13	0.14927276	0.06	39.4%
Slc6a14	0.01641318	0.01	83.7%
Slc6a15	25.04166	0.91	3.6%
Slc6a17	36.26916	1.13	3.1%
Slc6a18	0	0.00	
Slc6a19	0	0.00	
Slc6a2	0.04421634	0.03	68.0%
Slc6a20	0	0.00	
Slc6a3	0.0290507	0.02	63.0%
Slc6a4	0.00506272	0.01	140.8%
Slc6a5	24.2581	0.85	3.5%
Slc6a6	16.17474	1.22	7.6%
Slc6a7	3.965752	0.36	9.0%
Slc6a8	21.65032	1.45	6.7%
Slc6a9	11.35406	1.07	9.4%
Slc7a1	18.01716	1.96	10.9%
Slc7a10	0.5529588	0.12	21.4%
Slc7a11	5.089438	0.46	9.0%
Slc7a12	0	0.00	
Slc7a13	0	0.00	
Slc7a14	18.51448	1.69	9.1%
Slc7a15	0	0.00	
Slc7a2	1.776216	0.19	10.4%
Slc7a3	12.687578	4.16	32.8%
Slc7a4	7.472322	0.10	1.3%
Slc7a5	29.19494	3.00	10.3%
Slc7a6	6.83175	0.45	6.5%
Slc7a6os	9.227348	0.35	3.8%
Slc7a7	2.27728	0.48	21.2%
Slc7a8	3.49043	0.48	13.7%

Slc7a9	0	0.00	
Slc8a1	40.83216	3.80	9.3%
Slc8a2	20.54632	2.42	11.8%
Slc8a3	13.62154	0.61	4.4%
Slc9a1	14.49846	0.91	6.3%
Slc9a10	0	0.00	
Slc9a2	0.496977	0.15	30.2%
Slc9a3	0.03114312	0.02	59.0%
Slc9a3r1	2.075178	0.24	11.7%
Slc9a3r2	12.835	0.73	5.7%
Slc9a4	0.0267017	0.02	64.1%
Slc9a5	4.828426	0.31	6.4%
Slc9a6	36.78184	1.67	4.5%
Slc9a7	17.78956	2.07	11.7%
Slc9a8	10.198948	0.55	5.4%
Slc9a9	1.703242	0.13	7.6%
Slco1a1	0.0025474	0.01	223.6%
Slco1a4	0.2046974	0.07	35.2%
Slco1a5	0.03976842	0.02	58.3%
Slco1a6	0.00377198	0.01	223.6%
Slco1b2	0.02933656	0.02	78.3%
Slco1c1	0.07943992	0.03	35.7%
Slco2a1	0.1813614	0.05	29.9%
Slco2b1	0.08331052	0.03	36.7%
Slco3a1	10.54576	0.56	5.3%
Slco4a1	0.5927268	0.06	9.3%
Slco4c1	0.7632502	0.19	24.7%
Slco5a1	0.7854086	0.11	14.2%
Slco6b1	0	0.00	
Slco6c1	0.01393286	0.01	105.8%
Slco6d1	0	0.00	
Slfn1	0	0.00	
Slfn10	0.00443275	0.01	138.1%
Slfn2	0.01428856	0.01	59.1%
Slfn3	0	0.00	
Slfn4	0	0.00	
Slfn5	0	0.00	
Slfn8	0.00215154	0.00	223.6%
Slfn9	0.00233468	0.01	223.6%
Slfnl1	0.01471278	0.02	110.2%
Slirp	1.3324456	0.73	54.9%
Slit1	11.38008	0.24	2.1%
Slit2	10.123024	0.28	2.7%
Slit3	1.474238	0.27	18.6%
Slitrk1	15.58946	0.42	2.7%
Slitrk2	4.003502	0.37	9.2%
Slitrk3	10.110712	0.32	3.2%

Slitrk4	13.1223	0.94	7.1%
Slitrk5	11.5863	0.56	4.8%
Slitrk6	0.9407436	0.14	14.4%
Slk	10.87342	0.77	7.1%
Slm1	0.396027	0.14	35.4%
Slmap	20.99106	0.95	4.5%
Slmo1	16.97138	0.58	3.4%
Slmo2	14.9691	0.81	5.4%
Sln	0	0.00	
Slo	4.7084	0.95	20.3%
Slo3	0.1410378	0.03	19.1%
slp1	0.01902272	0.01	74.6%
slp2-a	0.009608754	0.01	100.0%
Slpi	0	0.00	
Sltm	10.859112	0.79	7.2%
Slu7	11.16436	0.30	2.7%
Slurp1	0	0.00	
Sly	0	0.00	
Smad1	11.07352	0.38	3.4%
Smad2	11.9098	0.95	8.0%
Smad3	1.776714	0.24	13.3%
Smad4	9.935822	0.42	4.3%
Smad5	12.02462	1.02	8.5%
Smad6	0.07089436	0.04	57.6%
Smad7	1.391508	0.09	6.8%
Smad9	5.177584	0.60	11.6%
Smap1	43.03198	3.67	8.5%
Smap1l	43.89344	0.79	1.8%
Smarca1	10.70736	0.58	5.4%
Smarca2	45.65092	2.02	4.4%
Smarca4	35.8607	0.70	2.0%
Smarca5	4.969818	0.12	2.3%
Smarcad1	6.429996	0.13	2.0%
Smarcal1	15.41396	0.71	4.6%
Smarcb1	25.86778	2.20	8.5%
Smarcc1	14.89138	0.47	3.1%
Smarcc2	41.65764	1.36	3.3%
Smarcd1	31.1435	1.10	3.5%
Smarcd2	3.014922	0.39	13.0%
Smarcd3	17.90186	1.00	5.6%
Smarce1	5.044202	0.31	6.1%
smarp	0.01855172	0.03	163.2%
Smc1a	14.25194	0.55	3.8%
Smc1b	0.08763344	0.02	20.9%
Smc2	2.038818	0.29	14.4%
Smc3	12.78346	0.44	3.4%
Smc4	2.951278	0.39	13.3%

SMC4	0.10349996	0.03	25.1%
Smc5	7.471942	0.81	10.8%
Smc6	12.31442	0.69	5.6%
SMC6	0.7991118	0.39	48.7%
SmcB	0.07322518	0.02	25.4%
Smchd1	5.517452	0.40	7.2%
Smcp	0	0.00	
Smcr7	6.272394	0.24	3.8%
Smcr7l	16.22396	0.45	2.8%
Smcr8	7.433826	0.46	6.2%
Smcy	0.000581326	0.00	223.6%
Smek1	8.47688	0.46	5.4%
Smek2	14.25338	0.83	5.8%
Smg1	1.77325164	2.40	135.5%
Smg5	18.57996	0.82	4.4%
Smg6	7.605496	0.90	11.8%
Smg7	19.95426	0.23	1.1%
Smgc	0	0.00	
SMIF	0.02620538	0.00	10.6%
SMN	0.970297	0.60	61.8%
Smn1	6.728204	0.94	13.9%
Smndc1	10.63352	0.73	6.8%
Smo	4.474974	0.50	11.2%
Smoc1	4.313514	0.34	7.8%
Smoc2	3.284958	1.02	30.9%
smoc2	1.9700612	1.01	51.4%
Smok(Tcr)	0	0.00	
Smok3b	0	0.00	
Smox	7.556516	1.09	14.5%
Smpd1	31.27524	2.07	6.6%
Smpd2	1.338618	0.17	12.6%
Smpd3	9.075684	1.65	18.2%
Smpd4	9.023568	0.57	6.3%
Smpdl3a	0.5906336	0.10	17.4%
Smpdl3b	0.09035024	0.04	46.6%
Smpx	0	0.00	
Smr1	0	0.00	
Smr2	0.01103474	0.02	223.6%
smrp1	0.157379	0.03	21.7%
Smrte	3.735034	0.33	8.8%
Sms	37.47654	1.31	3.5%
Smt3ip2	0.9857706	0.19	19.6%
Smtn	1.1658144	0.28	23.8%
Smtnl1	0	0.00	
Smtnl2	1.649456	0.26	15.7%
Smu1	23.38576	0.40	1.7%
Smug1	7.408624	0.61	8.3%

Smurf1	11.1772	0.44	4.0%
Smurf2	6.249344	0.27	4.3%
SmX5	1.6588984	0.80	48.4%
Smyd1	0.2312956	0.03	14.2%
Smyd2	34.3835	1.17	3.4%
Smyd3	11.82052	1.04	8.8%
Smyd4	2.524002	0.14	5.6%
Smyd5	14.18324	1.17	8.2%
Snag1	3.611768	0.28	7.8%
Snai1	1.1784388	0.37	31.2%
Snai2	0.4048822	0.06	15.6%
Snai3	0.00782592	0.01	138.9%
Snap23	2.398246	0.21	8.8%
Snap25	706.9992	37.24	5.3%
Snap29	13.81612	1.15	8.3%
Snap91	131.248	1.24	0.9%
Snapap	20.38016	1.13	5.6%
Snapc1	4.856684	0.21	4.2%
Snapc2	8.541522	0.75	8.8%
Snapc3	7.394388	1.23	16.7%
Snapc4	6.906126	0.36	5.2%
Snapc5	10.294362	0.99	9.6%
Snca	65.51082	6.64	10.1%
Sncaip	2.70405	0.28	10.4%
Sncb	249.5244	14.77	5.9%
Sncg	14.03992	1.45	10.3%
Snd1	25.22702	1.57	6.2%
Sned1	0.4613728	0.12	26.7%
Snep	0.0339361	0.01	43.3%
Snf1lk	3.004474	0.24	8.1%
Snf1lk2	0.4880742	0.29	59.7%
Snf2l	0.16529246	0.08	46.1%
Snf8	10.25843	0.63	6.2%
Snip1	2.883208	0.20	6.8%
Snn	40.48944	2.77	6.9%
Sno	0.6264174	0.13	20.5%
Snph	24.15096	1.47	6.1%
Snrk	20.31768	1.24	6.1%
Snrp70	70.13074	5.94	8.5%
Snrpa	14.95956	1.50	10.1%
Snrpa1	10.984936	0.84	7.6%
Snrpb	25.76134	1.56	6.1%
Snrpb2	13.89222	0.96	6.9%
Snrpc	3.522224	0.28	8.0%
Snrpd1	11.19758	0.55	4.9%
Snrpd2	21.2595	2.34	11.0%
Snrpd3	30.15538	1.57	5.2%

Snrpe	8.344352	0.57	6.9%
Snrpf	0.6960272	0.36	51.6%
Snrpg	2.474424	0.24	9.9%
Snrpn	18.55878	2.07	11.1%
Snta1	3.351776	0.15	4.4%
Sntb1	2.076774	0.22	10.5%
Sntb2	1.371456	0.26	18.9%
Sntg1	10.534608	0.52	5.0%
Sntg2	1.0152692	0.13	12.6%
Snupn	5.832618	0.55	9.4%
Snurf	260.9588	10.30	3.9%
Snw1	11.907	1.06	8.9%
Snx1	17.154	0.50	2.9%
Snx10	33.06056	0.52	1.6%
Snx11	6.181886	0.59	9.5%
Snx12	16.34034	0.69	4.2%
Snx13	17.77436	0.79	4.4%
Snx14	26.11698	1.33	5.1%
Snx15	9.556904	0.75	7.8%
Snx16	22.0115	1.75	7.9%
Snx17	26.8237	1.97	7.3%
Snx19	14.82584	0.62	4.2%
Snx2	24.11162	0.83	3.4%
Snx21	2.524476	0.35	13.8%
Snx22	1.262742	0.11	8.7%
Snx24	4.693242	0.31	6.7%
Snx25	15.44368	0.59	3.8%
Snx26	25.21008	3.23	12.8%
Snx27	34.48358	1.00	2.9%
Snx28	0.3188452	0.11	33.6%
Snx29	0.04101466	0.01	20.7%
Snx3	80.1066	5.69	7.1%
Snx30	15.95952	0.78	4.9%
Snx4	15.34492	1.54	10.0%
Snx5	19.16298	0.88	4.6%
Snx6	18.24068	1.38	7.5%
Snx7	3.220866	0.43	13.3%
Snx8	15.6093	1.17	7.5%
Snx9	2.26541	0.21	9.4%
Soat1	3.63496	0.47	12.9%
Soat2	0.10999252	0.05	48.0%
Sobp	6.317376	0.52	8.2%
Socs1	0.7169206	0.17	24.2%
Socs2	1.68122	0.52	30.8%
Socs3	0.7937312	0.25	31.0%
Socs4	2.689916	0.39	14.7%
Socs5	15.15818	1.26	8.3%

Socs6	2.53456	0.21	8.4%
Socs7	9.629622	0.62	6.4%
Sod1	37.67126	3.04	8.1%
Sod2	56.3916	3.10	5.5%
Sod3	1.746018	0.40	22.8%
Sohlh1	0	0.00	
Sohlh2	0.01874582	0.01	74.9%
Solh	8.310892	0.65	7.8%
Solt	0.06607116	0.04	56.6%
Son	24.63616	1.06	4.3%
Sorbs1	16.60988	0.51	3.1%
Sorbs2	5.905352	0.42	7.1%
Sorbs3	1.1335042	0.17	14.7%
SorCS	0.8213092	0.37	44.7%
Sorcs1	8.195824	0.29	3.5%
Sorcs2	5.753464	0.73	12.8%
Sorcs3	12.54066	0.39	3.1%
Sord	7.75943	0.56	7.2%
Sorl1	7.311602	0.85	11.6%
Sort1	25.62196	0.39	1.5%
Sos1	15.7424	0.76	4.9%
Sos2	9.305812	0.35	3.7%
Sost	0.0243475	0.04	156.4%
Sostdc1	1.636576	0.35	21.3%
Sox1	0.527763	0.14	27.4%
Sox10	1.5867	0.53	33.6%
Sox11	7.88428	0.32	4.0%
Sox12	4.952982	0.42	8.6%
Sox13	0.4098234	0.12	30.3%
sox-13	0.2826776	0.12	44.1%
Sox15	0.19855248	0.07	35.3%
Sox17	0.7954558	0.22	28.1%
Sox18	0.14773426	0.08	55.0%
Sox2	4.292308	0.51	11.8%
Sox21	2.130438	0.29	13.8%
Sox3	0.9805572	0.15	15.2%
Sox30	0.022136582	0.02	94.8%
Sox4	14.31274	0.17	1.2%
Sox5	0.533016	0.08	14.8%
Sox6	2.550702	0.05	2.1%
Sox7	0.09938638	0.03	32.8%
Sox8	2.536144	0.28	11.2%
Sox9	2.928194	0.37	12.6%
SP1	5.919352	0.90	15.1%
Sp1	4.541562	0.18	4.0%
Sp100	0.00368766	0.01	223.6%
Sp110	0	0.00	

Sp140	0.0137502	0.01	91.8%
Sp2	2.282884	0.16	7.0%
Sp3	10.318414	0.54	5.2%
Sp4	3.533694	0.32	9.0%
Sp5	0.0192973	0.02	103.6%
Sp6	0.1671154	0.02	9.5%
Sp7	0.0022464	0.01	223.6%
Sp8	0.05859352	0.02	34.1%
Sp9	0.10290596	0.04	35.3%
Spa17	1.77759	0.13	7.5%
Spaca1	0	0.00	
Spaca3	0.0157142	0.02	139.4%
Spaca4	0	0.00	
Spaca5	0.01225892	0.03	223.6%
Spag1	2.820212	0.41	14.6%
Spag11	0	0.00	
Spag16	0.00921256	0.02	223.6%
Spag17	0.0064112	0.01	83.3%
Spag4	1.0751518	0.07	6.4%
Spag4l	0.00419794	0.01	223.6%
Spag5	0.8396494	0.15	17.9%
Spag6	1.415258	0.18	12.5%
Spag7	25.1565	1.65	6.6%
Spag8	0.2010818	0.05	24.9%
Spag9	51.11486	1.94	3.8%
Spam1	0	0.00	
Sparc	90.92488	13.10	14.4%
Sparcl1	131.3782	10.31	7.8%
Spast	16.99416	1.01	5.9%
Spata1	0.5133872	0.10	18.9%
Spata13	0.2086662	0.09	44.1%
Spata16	0	0.00	
Spata17	0.06138684	0.05	88.9%
Spata18	0	0.00	
Spata19	0.0795871	0.05	67.8%
Spata2	11.14676	0.69	6.2%
Spata20	0.02078172	0.02	76.8%
Spata21	0.010848238	0.01	92.5%
Spata22	0.03987146	0.03	69.6%
Spata2L	2.854884	0.28	9.7%
Spata3	0.00499616	0.01	223.6%
Spata4	0	0.00	
Spata5	5.575676	0.53	9.5%
Spata5l1	1.226646	0.13	10.5%
Spata6	3.693014	0.34	9.2%
Spata7	7.148546	0.48	6.8%
Spata9	1.718542	0.28	16.1%

Spatc1	0	0.00	
Spatial	0	0.00	
Spats1	1.0688904	0.20	18.7%
Spats2	12.16368	0.68	5.6%
Spbc25	1.548102	0.21	13.6%
Spc24	0.8503016	0.09	11.1%
Spc25	0.2729568	0.13	46.6%
Spcs1	56.25104	3.76	6.7%
Spcs2	21.94444	1.02	4.6%
Spcs3	44.29044	3.47	7.8%
Spdef	0.1555601	0.05	34.7%
Spdya	2.6553246	2.14	80.4%
Spdyb	0.01605466	0.02	116.8%
Specc1	5.283828	1.10	20.9%
Specc1l	8.148146	0.24	3.0%
Speer2	0	0.00	
Speer3	0	0.00	
Speer4a	0.02788666	0.01	50.6%
Speer4b	0.09909284	0.05	50.8%
Speer4c	0	0.00	
Speer4d	0	0.00	
Speer4f	0	0.00	
Speer6-ps1	0	0.00	
Spef1	2.302228	0.27	11.6%
Spef2	0.05743946	0.02	27.6%
Speg	5.225044	0.28	5.4%
Spem1	0.1637608	0.07	41.2%
Spen	6.043434	0.24	3.9%
Spert	0	0.00	
Spesp1	0.007344748	0.01	94.7%
Spg20	4.139666	0.35	8.5%
Spg21	9.928676	0.43	4.4%
Spg3a	45.30018	1.39	3.1%
Spg7	20.20866	0.25	1.2%
Sphk1	0.5326004	0.11	21.5%
SPHK1a	0.05426504	0.03	47.2%
Sphk2	6.397908	0.40	6.3%
Spib	0.00291768	0.01	223.6%
Spic	0	0.00	
Spin1	67.27674	2.21	3.3%
Spin2	4.086348	0.44	10.8%
Spin4	0.6542008	0.10	14.7%
Spink10	3.002414	0.68	22.6%
Spink11	0	0.00	
Spink12	0	0.00	
Spink2	0.02732504	0.04	139.1%
Spink3	0.4677086	0.19	40.5%

Spink4	0.18423954	0.11	57.6%
Spink5	0.003309992	0.00	138.8%
Spink7	0	0.00	
Spink8	0.01945468	0.02	97.0%
Spinlw1	0	0.00	
Spint1	0.420641	0.09	21.2%
Spint2	9.241384	0.86	9.4%
Spint4	0	0.00	
Spire1	19.79062	0.62	3.1%
Spire2	11.92414	0.64	5.4%
Spn	0.003646742	0.01	138.6%
Spna1	0.026116616	0.01	50.3%
Spna2	145.6314	3.48	2.4%
Spnb1	10.187686	0.33	3.2%
Spnb2	112.3522	3.75	3.3%
Spnb3	27.5221	1.42	5.2%
Spnb4	17.2452	2.86	16.6%
Spns1	27.03534	1.51	5.6%
Spns3	0.01526314	0.01	94.6%
Spo11	0.00357792	0.01	223.6%
Spock1	75.7922	2.13	2.8%
Spock2	139.3038	7.54	5.4%
Spock3	53.00884	1.65	3.1%
Spon1	16.97456	1.45	8.6%
Spon2	0.05920096	0.02	31.6%
Spop	39.25658	0.70	1.8%
Spp1	1.2274184	0.28	22.7%
Spp2	0	0.00	
Sppl2b	4.749188	0.79	16.6%
Sppl3	38.9619	2.90	7.4%
Spr	7.560788	0.11	1.5%
Spred1	27.60474	4.20	15.2%
Spred2	22.1629	1.07	4.8%
Spred-2	0.09591938	0.04	39.1%
Spred3	9.327006	1.07	11.5%
Spred-3	0.24141252	0.09	36.5%
Sprn	2.952024	0.15	5.1%
Sprr1a	0.22415994	0.12	52.0%
Sprr1b	0	0.00	
Sprr2a	0	0.00	
Sprr2b	0	0.00	
Sprr2d	0	0.00	
Sprr2e	0	0.00	
Sprr2f	0	0.00	
Sprr2g	0	0.00	
Sprr2h	0	0.00	
SPRR2H	0	0.00	

Sprr2i	0	0.00	
Sprr2j	0	0.00	
Sprr2k	0	0.00	
Sprr3	0	0.00	
Sprr4	0	0.00	
Spry1	7.797362	1.16	14.9%
Spry2	11.210348	1.39	12.4%
Spry3	0	0.00	
Spry4	10.457	0.17	1.6%
Spryd3	86.05726	3.63	4.2%
Spryd4	4.310928	0.41	9.6%
Spsb1	1.540904	0.20	13.0%
Spsb2	1.295618	0.15	11.4%
Spsb3	20.47916	5.42	26.5%
Spsb4	3.36084	0.53	15.9%
Spt1	0	0.00	
Spt2	0	0.00	
Sptlc1	7.70214	0.31	4.1%
Sptlc2	11.43478	0.67	5.8%
Sptlc3	0.01537224	0.02	146.7%
Spty2d1	3.59621	0.18	5.0%
Spz1	0	0.00	
Sqle	108.72824	17.40	16.0%
Sqrdl	0.5693888	0.10	16.7%
Sqstm1	218.3232	4.28	2.0%
sr528	14.6614	0.52	3.5%
Sra1	15.12814	1.21	8.0%
Srbd1	2.074076	0.20	9.7%
Src	13.55078	0.33	2.5%
Srcrb4d	0.4629964	0.05	9.8%
Srd5a1	4.609578	1.13	24.5%
Srd5a2	0.13467036	0.08	58.9%
Srd5a2l	6.486954	0.48	7.4%
Srd5a2l2	0.04394868	0.01	32.3%
Srebf1	12.36232	1.69	13.7%
Srebf2	74.92268	9.26	12.4%
Srf	3.921132	0.19	4.9%
Srfbp1	4.89099	0.24	4.9%
Srgap1	4.027926	0.49	12.0%
Srgap2	17.34898	0.51	2.9%
Srgap3	39.61746	1.24	3.1%
Srgn	4.57158	1.79	39.1%
Sri	16.1837	0.32	2.0%
Srl	2.414052	0.25	10.6%
Srm	15.9492	1.93	12.1%
Srms	0.007088426	0.01	94.8%
Srp14	57.24654	3.54	6.2%

Srp19	23.28908	2.26	9.7%
Srp54a	0.232621846	0.32	137.7%
Srp54b	0.25913328	0.31	121.2%
Srp54c	16.88118	0.52	3.1%
Srp68	23.6782	0.68	2.9%
Srp72	34.14074	1.43	4.2%
Srp9	27.14122	0.71	2.6%
Srpk1	10.924552	0.72	6.6%
SRPK1	2.569954	1.02	39.9%
Srpk2	44.70708	1.36	3.0%
Srpk3	0.06014094	0.04	64.9%
Srpr	19.14738	0.99	5.2%
Srprb	13.6795	0.67	4.9%
Srpx	1.660656	0.36	21.4%
Srpx2	1.2078478	0.22	18.0%
Srr	28.99732	1.23	4.3%
Srrm1	27.4349	1.23	4.5%
Srrm2	46.10144	4.25	9.2%
Srrp	16.82736	0.95	5.6%
Srxn1	28.37002	0.72	2.5%
Ss18	2.819548	0.30	10.6%
Ss18l1	19.48358	0.97	5.0%
Ssb	58.15984	2.71	4.7%
Ssbp1	4.362722	0.37	8.6%
Ssbp2	11.13568	0.58	5.3%
Ssbp3	26.30582	1.55	5.9%
Ssbp4	21.5626	0.92	4.3%
Ssfa2	2.649724	0.27	10.2%
Ssh1	2.61596	0.18	7.0%
Ssh2	4.881602	0.36	7.3%
Ssh3	7.390452	0.62	8.4%
Ssna1	14.23694	0.85	6.0%
Sspn	1.540182	0.08	5.2%
Ssr1	47.91536	1.68	3.5%
Ssr2	49.75378	3.77	7.6%
Ssr3	63.04384	4.10	6.5%
Ssr4	29.93212	2.67	8.9%
Ssrp1	23.20428	0.61	2.6%
Sssca1	16.2938	1.83	11.2%
Sst	29.39772	4.70	16.0%
Sstr1	14.12698	0.63	4.4%
Sstr2	2.919584	0.31	10.5%
Sstr3	0.373493	0.05	12.8%
Sstr4	0.6302732	0.16	24.6%
Sstr5	0.00477514	0.01	137.0%
Ssty1	0	0.00	
Ssty2	0	0.00	

Ssu72	34.90142	2.88	8.3%
Ssx2ip	22.80348	0.76	3.3%
Ssx9	0	0.00	
Ssxa1	0.01434084	0.03	223.6%
Ssxb1	0	0.00	
Ssxb10	0	0.00	
Ssxb2	0	0.00	
Ssxb3	0	0.00	
Ssxb5	0	0.00	
Ssxb9	0	0.00	
St13	91.0912	1.50	1.6%
St14	0.1614442	0.03	16.8%
St18	0.08950192	0.02	21.4%
St3gal1	9.363624	0.61	6.5%
St3gal2	13.88462	0.50	3.6%
St3gal3	13.40418	0.66	4.9%
St3gal4	3.924798	0.13	3.4%
St3gal5	18.57128	0.67	3.6%
St3gal6	2.33511	0.37	15.8%
St5	3.385462	0.31	9.0%
St6gal1	1.581572	0.14	8.8%
St6gal2	1.999514	0.09	4.4%
ST6GalNAc_VI	9.607268	0.42	4.4%
St6galnac1	0	0.00	
St6galnac2	0.06955974	0.05	66.2%
St6galnac3	2.475014	0.21	8.4%
St6galnac4	6.928694	0.25	3.6%
St6galnac5	6.785518	0.46	6.7%
St6galnac6	15.6823	1.62	10.4%
St7	7.304232	0.52	7.1%
St7l	7.18144	0.24	3.3%
St8sia1	12.68302	0.43	3.4%
St8sia2	5.32002	0.40	7.5%
St8sia3	24.93602	0.81	3.2%
St8sia4	8.981962	0.54	6.0%
St8sia5	6.710706	0.61	9.1%
St8sia6	1.697428	0.22	12.7%
ST8SiaIV	0.1895488	0.05	25.3%
Stab1	0.41514118	0.50	121.5%
Stab2	0.024010974	0.01	57.6%
Stac	1.473802	0.45	30.2%
Stac2	12.10386	1.39	11.5%
Stac3	0.3780668	0.15	38.7%
Stag1	7.379434	0.08	1.1%
Stag2	13.50984	0.44	3.3%
Stag3	2.902354	0.25	8.6%
Stam	25.11228	0.98	3.9%

Stam2	8.442646	0.80	9.4%
Stambp	15.28366	1.23	8.0%
Stambpl1	7.618454	0.51	6.7%
Stap1	0	0.00	
stap-1	0.00306186	0.01	223.6%
Stap2	0.3101904	0.07	24.1%
Star	1.92836	0.13	6.6%
Stard10	15.13456	1.61	10.7%
Stard13	1.45216	0.22	15.5%
Stard3	4.976398	0.31	6.2%
Stard3nl	36.83536	1.33	3.6%
Stard4	26.53836	0.76	2.9%
Stard5	3.337146	0.29	8.6%
Stard6	0.0423001	0.03	73.9%
Stard7	31.16988	0.74	2.4%
Stard8	0.4623298	0.04	8.2%
Stard9	0.4172284	0.18	42.3%
Stat1	5.602708	0.32	5.7%
Stat2	6.264508	0.31	5.0%
Stat3	14.2895	0.51	3.6%
Stat4	0.019692132	0.02	92.3%
Stat5a	0.516961	0.05	9.8%
Stat5A	0.008311678	0.00	33.2%
Stat5b	7.186688	0.32	4.4%
Stat6	0.10730482	0.02	17.5%
Stau1	12.58446	11.58	92.1%
Stau2	72.30674	1.54	2.1%
Stbd1	1.2144912	0.21	17.5%
Stc1	6.727796	0.87	13.0%
Stc2	2.547554	0.62	24.3%
Stch	23.45124	0.81	3.5%
Steap1	0.369227	0.11	30.9%
Steap2	7.188288	0.90	12.5%
Steap3	0.611147	0.11	17.5%
Steap4	1.0775864	0.35	32.8%
Stef	0.0370572	0.02	62.4%
Stfa1	0	0.00	
Stfa2	0	0.00	
Stfa2l1	0	0.00	
Stfa3	0	0.00	
Stil	0.3238312	0.07	21.2%
Stim1	17.8721	0.98	5.5%
Stim2	8.344824	0.23	2.7%
Stip1	88.46684	1.77	2.0%
Stk10	0.6393682	0.11	17.0%
Stk11	21.06462	1.09	5.2%
Stk11ip	5.278954	0.19	3.5%

Stk16	11.88896	0.66	5.5%
Stk17b	1.1333144	0.15	13.4%
Stk19	4.616718	0.38	8.3%
Stk22s1	0.02611778	0.01	34.2%
Stk24	18.43282	0.90	4.9%
Stk25	50.9115	0.97	1.9%
Stk3	6.223352	0.16	2.6%
Stk31	0.05055698	0.02	40.9%
Stk32a	1.1535642	0.10	9.0%
Stk32b	13.50778	2.54	18.8%
Stk32c	10.087216	0.29	2.9%
Stk33	0.3561692	0.13	36.2%
Stk35	4.22338	0.23	5.5%
Stk36	1.50186	0.16	10.6%
Stk38	5.406066	0.46	8.5%
Stk38l	2.512446	0.10	4.1%
Stk39	27.9646	1.62	5.8%
Stk4	6.4238	0.35	5.5%
Stk40	7.355898	0.30	4.0%
Stmn1	10.506192	1.80	17.1%
Stmn2	398.1574	37.09	9.3%
Stmn3	453.6074	37.43	8.3%
Stmn4	53.47852	6.64	12.4%
Stom	3.096348	0.21	6.8%
Stoml1	22.30966	0.48	2.2%
Stoml2	10.87152	0.35	3.2%
Stoml3	0.00530688	0.01	223.6%
Ston2	1.0274986	0.06	5.9%
Stox2	17.0481	1.87	11.0%
Stra13	9.343788	1.18	12.6%
Stra6	1.769092	0.45	25.6%
Stra8	0.00288262	0.01	223.6%
Strap	67.43212	1.28	1.9%
Strbp	25.78574	1.08	4.2%
Strc	0.029986972	0.03	101.3%
Strn	8.008848	0.73	9.1%
Strn3	17.34354	0.42	2.4%
Strn4	27.67044	0.86	3.1%
Stt3a	29.26122	1.15	3.9%
Stt3b	33.4198	2.57	7.7%
Stub1	51.43798	2.12	4.1%
Stx11	0.10938086	0.04	38.5%
Stx12	50.47376	1.35	2.7%
Stx16	13.16784	1.59	12.1%
Stx17	8.935652	0.18	2.1%
Stx18	10.74362	0.42	4.0%
Stx19	0.01075814	0.02	140.5%

Stx1a	16.5539	1.95	11.8%
Stx1b2	137.9928	6.66	4.8%
Stx2	2.26214	0.18	8.0%
Stx3	6.236902	0.41	6.6%
Stx4a	8.168714	0.60	7.4%
Stx5	12.61652	0.90	7.2%
Stx5a	11.101046	0.93	8.4%
Stx6	13.87332	0.66	4.8%
Stx7	77.96604	5.72	7.3%
Stx8	5.282314	0.37	7.0%
Stxbp1	368.7822	13.20	3.6%
Stxbp2	2.451412	0.32	13.2%
Stxbp3a	1.273108	0.15	11.5%
Stxbp3b	0.7298708	0.12	16.0%
Stxbp4	2.98174	0.36	12.1%
Stxbp5	18.8195	0.54	2.9%
Stxbp5l	7.796934	1.53	19.6%
Stxbp6	23.55128	0.53	2.2%
Styk1	0.3085272	0.08	26.6%
Styx	0.305707	0.10	34.0%
Styxl1	0.05337634	0.08	141.7%
Sub1	40.02364	3.48	8.7%
Sucla1	0.03515402	0.01	29.7%
Sucla2	93.50432	4.86	5.2%
Suclg1	57.53332	2.56	4.4%
Suclg2	1.271418	0.10	8.2%
Sucnr1	0.01628672	0.03	158.3%
Suds3	21.51614	1.17	5.4%
Sufu	2.968296	0.15	5.1%
Sugt1	21.6278	1.28	5.9%
Suhw3	0.3270626	0.14	44.1%
Suhw4	8.975254	0.66	7.3%
Sulf1	2.878136	0.22	7.7%
Sulf2	26.38954	2.27	8.6%
Sult1a1	1.053537	0.29	27.6%
Sult1b1	0	0.00	
Sult1c1	0	0.00	
Sult1c2	0.05386734	0.02	32.5%
Sult1d1	0.00775988	0.01	137.2%
Sult1e1	0	0.00	
Sult2b1	0.00415982	0.01	223.6%
Sult3a1	0	0.00	
Sult4a1	158.0354	7.14	4.5%
Sult5a1	0.12521762	0.04	34.6%
Sumf1	8.402208	0.52	6.1%
Sumf2	6.136006	0.57	9.3%
Sumo1	20.98736	0.66	3.2%

Sumo2	0.08730908	0.04	48.2%
Sumo3	34.3261	1.49	4.3%
Sunc1	0.5083744	0.07	13.0%
Suox	3.188906	0.29	9.1%
Supl15h	2.012216	0.67	33.2%
Supt16h	18.43136	0.72	3.9%
Supt3h	11.63772	1.23	10.6%
Supt4h1	1.5668302	0.41	26.1%
Supt4h2	7.838964	0.55	7.0%
Supt5h	33.65338	0.80	2.4%
Supt6h	21.49258	0.81	3.8%
Supt7l	6.400214	0.46	7.2%
Supv3l1	8.36682	0.50	6.0%
Sur2	1.1005332	0.52	47.7%
Surf1	30.0839	1.27	4.2%
Surf2	14.98956	4.03	26.9%
Surf4	26.10412	2.01	7.7%
Surf6	6.485758	0.22	3.3%
Susd2	7.020436	0.79	11.3%
Susd3	0.00791444	0.01	138.9%
Susd4	40.01998	1.12	2.8%
Suv39h1	8.612998	1.40	16.2%
Suv39h2	0.4743762	0.07	14.7%
Suv420h1	10.64052	0.39	3.7%
Suv420h2	7.036636	0.45	6.4%
Suz12	8.930564	0.53	5.9%
Sv2a	145.4704	4.15	2.9%
Sv2b	14.71162	0.20	1.4%
Sv2c	24.58828	0.67	2.7%
Sva	0	0.00	
Sval1	0	0.00	
Sval2	0	0.00	
Sval3	0	0.00	
svct2	0.4504212	0.22	48.7%
Svep1	2.185076	0.61	28.1%
Svil	6.053976	0.75	12.4%
Svop	41.30576	1.38	3.3%
Svopl	0.04471512	0.03	66.2%
Svs1	0	0.00	
Svs2	0	0.00	
Svs3a	0.00484608	0.01	223.6%
Svs3b	0	0.00	
Svs4	0	0.00	
Svs5	0	0.00	
Svs6	0	0.00	
Svs7	0.0553664	0.08	137.0%
Swap70	1.593314	0.10	6.2%

Syap1	11.41134	0.46	4.0%
Sybl1	0	0.00	
Syce1	0.193164	0.05	23.6%
Syce2	2.062402	0.21	10.2%
Sycn	0	0.00	
Sycp1	0.4242042	0.07	16.6%
Sycp2	0.2327174	0.07	30.1%
Sycp3	4.388682	0.85	19.3%
Syde1	2.258358	0.34	15.0%
Syde2	0.7249822	0.08	10.8%
Syf2	10.254968	0.48	4.7%
Syk	0.1436594	0.04	28.8%
Sympk	20.10112	0.41	2.0%
Syn1	140.7648	7.37	5.2%
Syn2	109.52746	7.58	6.9%
Syn3	12.37332	1.39	11.2%
Sync	3.651292	1.33	36.4%
Syncrip	19.4243	1.17	6.0%
Syne1	8.547792	0.31	3.6%
Syne2	1.797798	0.16	8.7%
Syngr1	150.5436	7.83	5.2%
Syngr2	1.87301	0.38	20.5%
Syngr3	128.5664	5.07	3.9%
Syngr4	0.03597854	0.03	95.2%
Synj1	48.23858	2.11	4.4%
Synj2	1.963838	0.24	12.3%
Synj2bp	21.23816	2.02	9.5%
Synpo	2.320354	0.36	15.3%
Synpo2l	0.09094956	0.03	36.4%
Synpr	16.04598	0.58	3.6%
Syp	413.217	4.65	1.1%
Sypl	8.600524	0.35	4.0%
Sypl2	0.02763568	0.02	68.3%
Sys1	8.938042	0.81	9.0%
syt_II	9.05018	1.88	20.8%
syt_XV-a	0.0113659	0.03	223.6%
Syt1	122.8554	4.10	3.3%
Syt10	0.4298022	0.13	29.8%
Syt11	140.9614	3.05	2.2%
Syt12	14.8733	0.33	2.2%
Syt13	26.2365	0.96	3.7%
Syt14	1.555946	0.17	11.2%
Syt15	0.0290545	0.02	81.9%
Syt16	16.66398	1.66	10.0%
Syt17	6.655648	0.44	6.7%
Syt2	58.58404	6.23	10.6%
Syt3	34.08048	2.19	6.4%

Syt4	60.20982	2.18	3.6%
Syt5	12.29398	1.81	14.7%
Syt6	3.401822	0.33	9.6%
Syt7	25.68704	3.08	12.0%
Syt8	0.00609614	0.01	223.6%
Syt9	5.801288	0.32	5.5%
Sytl1	0.0173604	0.01	33.0%
Sytl2	0.8126322	0.16	19.5%
Sytl3	0.00402448	0.01	223.6%
Sytl4	0.07939456	0.01	14.1%
Sytl5	0.08836	0.06	67.2%
sytXIV	0.01980152	0.00	20.5%
Syvn1	25.29068	0.90	3.6%
T	0.01173126	0.01	106.0%
T2	0.03422682	0.02	64.5%
T2bp	0.8129864	0.13	16.0%
Taar1	0	0.00	
Taar2	0.02434118	0.02	92.7%
Taar3	0	0.00	
Taar4	0.02822412	0.04	149.4%
Taar5	0	0.00	
Taar6	0	0.00	
Taar7a	0	0.00	
Taar7b	0	0.00	
Taar7d	0	0.00	
Taar7e	0	0.00	
Taar7f	0	0.00	
Taar8a	0	0.00	
Taar8b	0	0.00	
Taar8c	0	0.00	
Taar9	0	0.00	
Tac1	38.24288	2.72	7.1%
Tac2	0.07652368	0.05	70.9%
Tac4	0.00515716	0.01	223.6%
Tacc1	11.4064	0.79	6.9%
Tacc2	18.88476	0.56	3.0%
Tacc3	3.154218	0.25	8.1%
Tacr1	8.85355	0.20	2.3%
Tacr2	0	0.00	
Tacr3	3.022146	0.13	4.4%
Tacstd1	0.5935186	0.20	34.2%
Tacstd2	0.0075244	0.01	142.2%
Tada1l	14.01208	0.71	5.0%
Tada2l	5.750512	0.40	6.9%
Tada3l	8.851642	0.54	6.1%
Taf1	18.94094	1.10	5.8%
Taf10	3.143954	0.07	2.1%

Taf11	6.84615	0.28	4.1%
Taf12	5.68985	0.20	3.5%
Taf13	9.171914	0.96	10.5%
Taf15	23.23466	1.25	5.4%
TAF170	0.02384402	0.00	20.7%
Taf1a	0	0.00	
Taf1b	8.37354	0.65	7.7%
Taf1c	2.887238	0.03	0.9%
Taf2	11.55892	0.39	3.4%
Taf3	4.176718	0.37	8.9%
Taf4a	2.936398	0.17	5.9%
Taf4b	1.25358	0.25	19.7%
Taf5	2.584384	0.10	3.8%
Taf5l	4.30926	0.14	3.3%
Taf6	9.12881	0.12	1.3%
Taf6l	4.528028	0.27	5.9%
Taf7	5.62742	0.58	10.3%
Taf7l	0.2846722	0.09	30.9%
Taf8	2.117302	0.13	6.3%
Taf9	28.53246	2.23	7.8%
Taf9b	10.290252	1.05	10.2%
Tagap	0	0.00	
Tagap1	3.332196	0.21	6.2%
tagL	0	0.00	
Tagln	0.7120328	0.10	14.7%
Tagln2	3.04183	0.53	17.3%
Tagln3	56.11136	2.22	4.0%
Tal1	0.6174098	0.04	6.1%
Tal2	0.0795676	0.07	91.8%
Taldo1	12.77812	2.11	16.5%
Tanc1	3.431522	0.36	10.4%
Tanc2	12.32714	0.73	6.0%
Tank	5.436526	0.28	5.1%
Taok1	15.28996	0.95	6.2%
Taok2	30.2535	0.51	1.7%
Taok3	8.016208	0.40	4.9%
Tap1	2.820746	0.35	12.3%
Tap2	1.414884	0.17	12.1%
Tapbp	11.11582	0.74	6.6%
Tapbpl	1.58096	0.08	4.8%
TAPL	0.224176	0.05	20.1%
Tapt1	14.47226	0.48	3.3%
Tarbp2	3.935762	0.19	4.9%
Tardbp	23.31202	0.94	4.0%
TARDBP	0.470575	0.35	74.3%
Tars	18.39134	1.15	6.3%
Tars2	9.691028	0.63	6.5%

TARSH	0.006120188	0.01	95.0%
Tarsl2	14.90094	1.23	8.3%
Tas1r1	1.181784	0.06	4.7%
Tas1r2	0.009936664	0.01	109.1%
Tas1r3	0.12414	0.01	5.5%
Tas2r102	0	0.00	
Tas2r104	0	0.00	
Tas2r105	0	0.00	
Tas2r106	0	0.00	
Tas2r107	0	0.00	
Tas2r108	0	0.00	
Tas2r109	0	0.00	
Tas2r110	0	0.00	
Tas2r113	0	0.00	
Tas2r114	0	0.00	
Tas2r115	0	0.00	
Tas2r116	0	0.00	
Tas2r117	0	0.00	
Tas2r118	0	0.00	
Tas2r119	0	0.00	
Tas2r120	0	0.00	
Tas2r121	0	0.00	
Tas2r122	0	0.00	
Tas2r123	0	0.00	
Tas2r124	0	0.00	
Tas2r125	0.01250448	0.03	223.6%
Tas2r126	0	0.00	
Tas2r129	0	0.00	
Tas2r130	0	0.00	
Tas2r131	0	0.00	
Tas2r134	0	0.00	
Tas2r135	0.0108957	0.02	223.6%
Tas2r136	0	0.00	
Tas2r137	0.01759344	0.02	140.8%
Tas2r138	0	0.00	
Tas2r139	0	0.00	
Tas2r140	0	0.00	
Tas2r143	0.01917676	0.03	140.9%
Tas2r144	0	0.00	
Tasp1	8.552282	0.47	5.5%
Tat	0	0.00	
Tatdn1	8.97488	0.99	11.0%
Tatdn2	11.61786	0.72	6.2%
Tatdn3	4.238728	0.50	11.8%
Tax1bp1	43.59154	1.92	4.4%
Tax1bp3	0.8601462	0.88	101.9%
Taz	10.8808	0.62	5.7%

Tbc1d1	4.332194	0.21	5.0%
Tbc1d10a	5.65692	0.62	11.0%
Tbc1d10b	22.11664	1.16	5.2%
Tbc1d10c	0.3033472	0.11	34.9%
Tbc1d12	7.736018	0.48	6.2%
Tbc1d13	17.39674	0.43	2.5%
Tbc1d14	13.40318	0.61	4.5%
Tbc1d15	7.589716	0.69	9.1%
Tbc1d16	6.582988	0.57	8.7%
Tbc1d17	13.51668	1.01	7.5%
Tbc1d19	14.09768	1.24	8.8%
Tbc1d2	0.1985146	0.05	25.3%
Tbc1d20	13.06542	0.81	6.2%
Tbc1d21	0	0.00	
Tbc1d22a	7.68479	0.51	6.7%
Tbc1d22b	16.57732	0.84	5.1%
Tbc1d23	11.51492	0.67	5.8%
Tbc1d24	22.31976	1.64	7.3%
Tbc1d25	4.010346	0.35	8.8%
Tbc1d2b	3.612724	0.37	10.2%
Tbc1d4	3.400216	0.29	8.6%
Tbc1d5	5.136944	0.22	4.3%
Tbc1d7	12.94614	0.51	3.9%
Tbc1d8	2.75215	0.19	7.0%
Tbc1d8b	0.5039474	0.07	14.7%
Tbc1d9	33.27882	0.98	3.0%
Tbc1d9b	28.82946	1.19	4.1%
Tbca	20.98608	1.65	7.9%
Tbcb	55.51008	6.82	12.3%
Tbcc	6.132322	0.43	7.0%
Tbccd1	3.476226	0.19	5.4%
Tbcd	21.39114	0.32	1.5%
Tbce	13.9259	1.27	9.1%
Tbcel	10.388204	0.79	7.6%
Tbk1	10.785968	0.64	5.9%
Tbkbp1	9.428704	0.72	7.6%
Tbl1x	15.91556	1.23	7.7%
Tbl1xr1	7.906706	0.42	5.3%
Tbl2	7.503682	0.55	7.4%
Tbl3	8.854838	0.63	7.1%
Tbn	0.5696418	0.15	25.6%
Tbp	8.047964	0.41	5.1%
Tbpl1	21.94576	0.73	3.3%
Tbpl2	0	0.00	
Tbr1	0.10862814	0.05	42.2%
Tbrg1	15.92746	1.27	8.0%
Tbrg4	12.4823	0.22	1.7%

Tbx1	0.1094976	0.06	54.3%
Tbx10	0.00365168	0.01	223.6%
Tbx15	1.99416	0.10	5.2%
Tbx18	0.6063392	0.08	12.7%
Tbx19	0	0.00	
Tbx2	1.1099886	0.19	16.8%
Tbx20	0.139281	0.03	24.2%
Tbx21	0.02968912	0.03	93.2%
Tbx22	0.00239726	0.01	223.6%
Tbx3	0.5784016	0.10	17.0%
Tbx4	0	0.00	
Tbx5	0.05774112	0.02	27.0%
Tbx6	0.1629394	0.04	26.2%
Tbxa2r	0.0245181	0.02	74.7%
Tbxas1	0.1476778	0.03	19.1%
Tc2n	0.03181948	0.02	51.0%
Tca	0	0.00	
Tcam1	0.10340148	0.03	32.9%
Tcap	0.05282498	0.04	75.4%
Tcb	0	0.00	
Tcba1	7.985178	0.40	5.0%
Tcea1	5.831032	0.33	5.6%
Tcea2	10.089226	0.87	8.7%
Tcea3	0.02727514	0.01	45.8%
Tceal1	14.36582	0.21	1.5%
Tceal3	20.39806	0.71	3.5%
Tceal5	18.26034	0.82	4.5%
Tceal6	12.27106	0.29	2.4%
Tceal8	17.1376	1.49	8.7%
Tceb1	48.8391	2.31	4.7%
Tceb2	27.81958	1.31	4.7%
Tceb3	7.201072	0.18	2.5%
Tcerg1	13.04764	0.41	3.2%
Tcerg1l	18.81542	2.11	11.2%
Tcf12	12.8666	0.91	7.1%
Tcf15	0.0694984	0.07	94.3%
Tcf19	0.989858	0.16	15.9%
Tcf20	16.24924	0.87	5.4%
Tcf21	0.0337449	0.03	96.9%
Tcf23	0.07731798	0.04	54.7%
Tcf25	225.9016	8.36	3.7%
Tcf3	1.62007	0.35	21.7%
Tcf4	0.22068456	0.21	93.7%
Tcf7	0.2887664	0.06	19.7%
Tcf7l2	3.151036	0.76	24.3%
TCF9	0.1295958	0.02	17.2%
Tcfap2a	5.781764	0.83	14.4%

Tcfap2b	3.965948	0.49	12.3%
Tcfap2c	0.225749	0.05	23.8%
Tcfap2d	0.02806398	0.02	86.5%
Tcfap2e	0.03914256	0.02	54.0%
Tcfap4	1.950354	0.26	13.5%
Tcfcp2	8.61894	0.46	5.3%
Tcfcp2l1	0.224464	0.04	19.4%
Tcfe2a	3.142084	0.35	11.3%
Tcfe3	5.105448	1.30	25.4%
Tcfeb	1.0784844	0.17	15.6%
Tcfec	0.03574168	0.04	110.5%
Tcfl5	1.647108	0.29	17.5%
Tchhl1	0	0.00	
Tchp	2.892248	0.19	6.5%
Tcirg1	2.754846	0.42	15.3%
Tcl1	0	0.00	
Tcl1b1	0	0.00	
Tcl1b2	0.00658426	0.01	223.6%
Tcl1b3	0	0.00	
Tcl1b4	0	0.00	
Tcl1b5	0	0.00	
Tcn2	3.551722	0.33	9.2%
Tcof1	3.522698	0.25	7.1%
Tcp1	73.39976	2.86	3.9%
Tcp10a	0.001393713	0.00	183.0%
Tcp10b	0.02844862	0.02	72.8%
Tcp10c	0.04934034	0.03	64.1%
Tcp11	0.05727984	0.02	40.8%
Tcp11l1	3.937658	0.60	15.1%
Tcp11l2	4.583442	0.97	21.2%
tcr	0	0.00	
TCR_[alpha]	0	0.00	
TCR_AV1S1Jalpha10	0	0.00	
TCR_BV10S1A2Dbeta1Jbeta2.1	0	0.00	
TCRA	0	0.00	
Tcra	0	0.00	
TCR-alpha_chain	0.09058512	0.10	108.1%
TCRB	0	0.00	
Tcrb	0	0.00	
TCR-beta_chain	0	0.00	
Tcrb-V13	0	0.00	
TCRBVbeta5.1/Jbeta1.5	0	0.00	
Tcrg	0	0.00	
Tcrg-C	0	0.00	
TCR-V[alpha]new	0	0.00	
TCRV-alpha_22.1	0	0.00	
Tcstv3	0	0.00	

Tcta	15.69564	1.56	9.9%
Tcte1	0.1525766	0.02	16.0%
Tcte2	1.4629464	1.01	69.4%
Tcte3	0.05624242	0.06	111.2%
Tctex1d1	0.0546124	0.07	137.0%
Tctex-3	7.271172	1.39	19.2%
Tctn2	7.134396	0.36	5.0%
Tctn3	5.630494	0.49	8.6%
Tdg	0.07264346	0.03	43.2%
Tdgf1	0.02146498	0.03	148.6%
Tdh	0.03344718	0.03	99.6%
Tdo2	0.02136042	0.01	61.7%
Tdp1	3.32166	0.48	14.6%
Tdpoz1	0	0.00	
Tdpoz2	0	0.00	
Tdpoz3	0	0.00	
Tdpoz4	0	0.00	
Tdpoz5	0	0.00	
Tdrd1	0.12691034	0.04	28.8%
Tdrd3	5.520854	0.31	5.6%
Tdrd5	0.3226222	0.10	31.3%
Tdrd6	0.1639812	0.02	14.2%
Tdrd7	8.58178	0.62	7.2%
Tdrd9	0.02018156	0.01	71.4%
Tdrkh	14.70184	0.28	1.9%
Tead1	1.697638	0.24	14.2%
Tead2	2.219278	0.36	16.1%
Tead3	1.091558	0.14	12.9%
Tead4	0.0412484	0.02	47.9%
Tec	0.5024736	0.11	21.6%
TECK	2.73811	0.25	9.2%
Tect1	5.202334	0.20	3.7%
Tect2	0.7188822	0.61	84.8%
Tect3	0.22804236	0.09	40.9%
Tecta	0.0414035	0.01	22.5%
Tectb	0.00455178	0.01	137.3%
Teddm1	0.00541768	0.01	223.6%
Tef	41.932	8.60	20.5%
Tegt	112.5974	7.26	6.5%
Tek	0.265558	0.08	30.3%
Tekt1	0.0538667	0.02	34.5%
Tekt2	0.11721566	0.04	37.5%
Tekt3	0.00304668	0.01	223.6%
Tekt4	0.01002842	0.01	92.7%
Tekt5	0.1854206	0.08	45.4%
Telo2	5.555862	0.31	5.5%
Tem8	1.0394078	0.32	31.2%

Tenc1	4.20515	1.49	35.4%
ten-m1	2.9871092	1.77	59.1%
ten-m2	0.6849166	0.34	50.4%
ten-m3	0.26987232	0.16	57.5%
ten-m4	0.1757864	0.07	38.0%
Tep1	0.4177998	0.09	22.0%
Tera	0.12711582	0.04	31.7%
Terf1	5.009978	0.38	7.5%
Terf2	10.89216	0.52	4.8%
Terf2ip	10.83168	0.43	3.9%
Tert	0.10816522	0.03	25.6%
Tes	6.395714	0.86	13.5%
Tesc	1.470546	0.28	19.2%
Tesk1	16.06072	1.75	10.9%
Tesk2	0.4313864	0.10	24.0%
Tesp1	0	0.00	
Tesp2	0	0.00	
Tessp2	0.00528904	0.01	223.6%
Tessp3	0	0.00	
testican-2	0.3135536	0.28	88.5%
Tex10	7.294426	0.60	8.2%
Tex101	0	0.00	
Tex11	0.1370286	0.03	20.0%
Tex12	0.0235694	0.03	138.2%
Tex13	0.4890162	0.07	15.0%
Tex14	0.21961774	0.07	33.7%
Tex15	1.48575	0.20	13.7%
Tex16	0.02441148	0.01	59.7%
Tex18	0	0.00	
Tex19	0.15957574	0.10	62.7%
Tex2	21.45094	1.69	7.9%
Tex21	0.00608898	0.01	138.6%
Tex22	0.01182808	0.03	223.6%
Tex24	0.0027331	0.01	223.6%
Tex261	11.1733	0.30	2.7%
Tex264	28.6741	1.85	6.5%
Tex9	0.1375866	0.04	30.6%
Tfam	9.36823	0.33	3.6%
Tfb1m	2.103232	0.50	23.6%
Tfb2m	2.425276	0.20	8.1%
Tfdp1	4.36548	0.44	10.1%
Tfdp2	2.970048	0.39	13.1%
Tfe3	4.861006	1.14	23.5%
Tff1	0	0.00	
Tff2	0.640876	0.52	81.1%
Tff3	0.0574305	0.06	108.2%
Tfg	47.66706	2.03	4.3%

Tfip11	11.26306	0.41	3.6%
Tfpi	1.836224	0.32	17.3%
Tfpi2	0.00501612	0.01	223.6%
Tfpt	7.56479	0.47	6.2%
Tfrc	113.9936	3.23	2.8%
Tg	0.018228242	0.01	49.6%
Tgds	3.37123	0.38	11.3%
Tgfa	1.59547	0.24	14.8%
Tgfb1	0.3556824	0.09	24.9%
Tgfb1i1	4.8374	1.34	27.7%
Tgfb2	3.785414	0.34	9.0%
Tgfb3	2.0988	0.30	14.5%
Tgfbi	0.2944156	0.09	29.4%
Tgfbr1	4.506456	0.39	8.6%
Tgfbr2	3.837246	0.61	15.8%
Tgfbr3	2.761522	0.63	22.8%
Tgfbrap1	8.528738	0.31	3.6%
Tgif1	0.8588008	0.17	20.0%
Tgif2	1.195474	0.17	14.2%
Tgifx1	0	0.00	
Tgm1	0.0296152	0.03	92.5%
Tgm2	1.564724	0.34	21.6%
Tgm3	0.07023916	0.02	32.1%
Tgm4	0.07435696	0.02	25.6%
Tgm5	0.00300406	0.01	223.6%
Tgm6	0.019405824	0.01	70.9%
Tgoln2	44.13556	1.34	3.0%
Tgs1	4.18683	0.28	6.6%
Tgtp	0	0.00	
Th	0.00716558	0.01	139.4%
Th1l	9.880228	0.57	5.8%
Tha1	1.221152	0.10	7.8%
Thada	2.07656	0.20	9.7%
Thap1	3.216592	0.29	9.2%
Thap11	9.093538	0.39	4.3%
Thap2	2.187998	0.19	8.5%
Thap3	5.459978	0.11	2.1%
Thap4	8.109882	0.35	4.3%
Thap7	6.366084	0.69	10.9%
Thbd	0.263292	0.08	31.5%
Thbs1	2.072334	0.37	18.0%
Thbs2	8.499788	1.08	12.7%
Thbs3	1.85783	0.15	8.3%
Thbs4	0.006071898	0.01	159.2%
Theg	0	0.00	
Them2	17.67984	1.09	6.2%
Them4	15.40616	0.58	3.8%

Them5	0.02448276	0.03	137.6%
Thex1	3.649138	0.31	8.4%
Thg1l	3.078626	0.50	16.2%
Thnsl1	7.205272	0.44	6.1%
Thnsl2	1.637238	0.27	16.8%
Thoc1	9.09688	0.71	7.8%
Thoc2	7.406092	0.52	7.0%
Thoc3	13.30362	1.16	8.7%
Thoc4	7.201162	0.47	6.6%
Thoc5	10.290514	0.52	5.1%
Thoc6	8.83613	2.35	26.6%
Thoc7	52.31254	1.81	3.5%
Thop1	8.137912	0.51	6.2%
Thpo	1.5529816	0.61	39.0%
Thra	152.0128	4.70	3.1%
Thrap2	0.354184	0.12	34.3%
Thrap3	10.352104	0.57	5.5%
Thrap4	5.276202	0.63	11.9%
Thrb	0.9959332	0.16	15.9%
Thrsp	4.15072	0.67	16.1%
Thsd1	0.364998	0.11	30.6%
Thsd4	0.7255902	0.08	10.9%
Thsd7b	4.97701	0.39	7.9%
Thtpa	10.6237	0.35	3.3%
Thumpd1	15.30082	0.60	3.9%
Thumpd2	1.1282226	0.26	23.4%
Thumpd3	8.872318	1.05	11.9%
Thy1	174.9756	11.78	6.7%
Thyn1	21.04954	2.37	11.3%
Tia1	18.49098	1.01	5.5%
Tial1	21.7389	1.58	7.3%
Tiam1	6.717722	0.18	2.7%
Tiam2	0.7648546	0.03	4.4%
Ticam1	0.6304422	0.07	10.5%
Ticam2	0.01716857	0.02	121.5%
Tid1	0.0567735	0.01	20.3%
Tie1	0	0.00	
Tifp39	0.01558924	0.03	223.6%
Tigd2	5.273862	0.38	7.3%
Tigd3	1.562126	0.11	6.8%
Tigd4	0	0.00	
Tigd5	1.643028	0.18	11.0%
Tilz3b	0.04950452	0.03	51.6%
Tim9b	8.860318	0.55	6.2%
Timd2	0	0.00	
Timd4	0	0.00	
Timeless	0.9582492	0.05	5.1%

Timm10	17.40996	2.33	13.4%
Timm13	24.17514	1.99	8.2%
Timm17a	21.15802	1.19	5.6%
Timm17b	8.037986	0.88	11.0%
Timm22	11.30498	0.41	3.6%
Timm23	2.24557	0.20	9.0%
Timm44	15.36362	0.99	6.5%
Timm50	15.80976	1.68	10.6%
Timm8a1	0.7514236	0.10	12.8%
Timm8a2	0.00501058	0.01	223.6%
Timm8b	50.66366	3.33	6.6%
Timm9	19.27712	1.63	8.4%
Timp1	1.052438	0.40	38.5%
Timp2	73.2074	1.78	2.4%
Timp3	8.793072	0.69	7.9%
Timp4	9.642564	1.00	10.3%
Tinag	0	0.00	
Tinagl	0.14827464	0.07	46.2%
Tinagl1	0.372162	0.03	6.8%
Tinf2	2.905032	0.32	10.9%
TIP5	0.41992742	0.39	93.2%
Tiparp	5.076766	0.46	9.1%
Tipin	3.23574	0.31	9.5%
TIPR	0.149307	0.06	37.2%
Tiprl	10.9871	0.79	7.2%
Tirap	0.4491808	0.06	13.9%
Titf1	0.00675354	0.01	137.0%
Tjap1	4.43627	0.44	10.0%
Tjp1	8.021982	0.30	3.7%
Tjp2	2.075662	0.27	12.8%
Tjp3	1.868558	0.11	5.9%
Tk1	0.4635328	0.08	17.3%
Tk2	16.71948	0.60	3.6%
Tkt	64.03532	3.80	5.9%
Tktl1	0.0348263	0.02	48.4%
Tktl2	0.015163654	0.01	69.8%
Tlcd1	2.281354	0.14	6.2%
Tle1	7.071462	0.92	13.0%
Tle2	2.411822	0.26	10.6%
Tle3	6.600028	0.54	8.2%
Tle4	18.60258	1.26	6.8%
Tle6	0.2041234	0.07	36.6%
Tlk1	12.68074	0.38	3.0%
Tlk2	16.38056	0.99	6.0%
Tll1	0.9515436	0.09	9.1%
Tll2	0.1659437	0.06	34.6%
Tln1	3.142692	0.42	13.3%

Tln2	15.52502	0.58	3.8%
Tloc1	50.5832	0.86	1.7%
Tlr1	0	0.00	
Tlr11	0	0.00	
Tlr12	0.06442678	0.01	23.0%
Tlr13	0	0.00	
Tlr2	0.2494668	0.02	9.0%
Tlr3	0.4495068	0.11	24.6%
Tlr4	0.0351555	0.05	152.2%
Tlr5	0.0421468	0.04	98.3%
Tlr6	0.016878	0.02	100.0%
Tlr7	0.00284644	0.01	223.6%
Tlr8	0	0.00	
Tlr9	0.001750422	0.00	223.6%
Tlx1	0.7306184	0.16	22.3%
Tlx2	0.00390096	0.01	223.6%
Tlx3	0.6540054	0.14	21.1%
Tm2d1	9.438086	0.63	6.6%
Tm2d2	33.7052	2.54	7.5%
Tm2d3	11.89308	0.38	3.2%
Tm4sf1	0.1596554	0.07	42.5%
Tm4sf20	0	0.00	
Tm4sf4	0	0.00	
Tm4sf5	0.2339174	0.07	31.3%
Tm6sf1	4.607614	1.15	24.9%
Tm6sf2	0.4953414	0.13	25.5%
Tm7sf1	1.450552	0.11	7.7%
Tm7sf2	41.10968	5.97	14.5%
Tm7sf3	7.95533	0.48	6.0%
Tm7sf4	0.0278291	0.02	59.6%
Tm9sf1	15.3729	1.15	7.5%
Tm9sf2	39.9308	1.25	3.1%
Tm9sf3	55.16316	1.42	2.6%
Tm9sf4	30.19744	0.50	1.7%
Tmbim1	1.845122	0.32	17.2%
Tmbim4	24.98102	1.35	5.4%
Tmc1	0.03325174	0.02	58.8%
Tmc2	0.00489416	0.01	137.0%
Tmc3	0.3482704	0.04	12.4%
Tmc4	4.04724	1.27	31.5%
Tmc5	0.004944244	0.01	142.9%
Tmc6	0.2806844	0.09	33.8%
Tmc7	1.44295	0.14	10.0%
Tmc8	0.028952608	0.02	71.2%
Tmcc1	8.506572	0.34	3.9%
Tmcc2	38.38478	1.98	5.1%
Tmcc3	3.837512	0.14	3.7%

Tmco1	7.756548	0.42	5.4%
Tmco2	0.01238346	0.03	223.6%
Tmco3	5.894202	0.89	15.1%
Tmco4	0.08444714	0.04	45.4%
Tmco5	0	0.00	
Tmco6	3.883526	0.53	13.7%
Tmco7	1.566538	0.11	7.0%
Tmed1	3.71461	0.42	11.3%
Tmed10	34.01244	1.32	3.9%
Tmed2	5.592846	0.44	7.9%
Tmed3	19.24176	1.35	7.0%
Tmed4	20.83632	0.86	4.1%
Tmed5	5.863622	0.27	4.7%
Tmed6	0	0.00	
Tmed8	13.12728	0.67	5.1%
Tmed9	40.353	2.84	7.0%
Tmeff1	57.66626	5.45	9.4%
Tmeff2	15.3992	0.94	6.1%
TMEFF2	0.05687302	0.05	84.0%
Tmem1	20.7404	1.03	4.9%
Tmem10	0	0.00	
Tmem100	0.742523	0.20	26.3%
Tmem101	13.95394	0.59	4.2%
Tmem102	0.366808	0.12	33.0%
Tmem103	4.564452	0.18	4.0%
Tmem104	3.662626	0.40	11.0%
Tmem106a	0.1087552	0.04	40.0%
Tmem106b	35.53282	4.17	11.7%
Tmem106c	9.155506	1.09	11.9%
Tmem107	3.835998	0.35	9.1%
Tmem108	5.715846	0.31	5.4%
Tmem109	12.31356	0.43	3.5%
Tmem11	12.3812	0.96	7.7%
Tmem110	4.374312	0.14	3.2%
Tmem111	25.7796	1.32	5.1%
Tmem112	4.86314	0.32	6.6%
Tmem112b	7.635216	0.29	3.7%
Tmem115	10.525776	0.69	6.6%
Tmem116	1.4690238	0.80	54.8%
Tmem117	17.56068	1.53	8.7%
Tmem118	28.98338	0.99	3.4%
Tmem119	0.7721078	0.24	31.6%
Tmem120a	7.98152	0.43	5.4%
Tmem120b	3.045412	0.23	7.5%
Tmem121	8.498754	0.78	9.2%
Tmem123	2.850378	0.38	13.2%
Tmem125	0.03142242	0.03	107.6%

Tmem126a	16.2539	1.80	11.1%
Tmem126b	15.07208	0.41	2.7%
Tmem127	49.32404	1.80	3.7%
Tmem128	26.27488	1.30	4.9%
Tmem129	9.170458	0.66	7.1%
Tmem130	183.3228	8.52	4.6%
Tmem131	12.76738	0.29	2.3%
Tmem132a	32.9914	2.23	6.7%
Tmem132b	4.713914	0.60	12.8%
Tmem132d	2.355102	0.14	6.1%
Tmem132e	6.075792	0.04	0.7%
Tmem134	7.406924	0.70	9.4%
Tmem135	25.12806	0.96	3.8%
Tmem136	5.614248	0.21	3.7%
Tmem138	8.807592	0.71	8.1%
Tmem139	0.02125222	0.02	102.6%
Tmem140	0.07475002	0.08	113.2%
Tmem141	8.108452	0.69	8.5%
Tmem143	7.138052	0.37	5.2%
Tmem144	0.4676818	0.07	15.5%
Tmem145	39.02164	1.82	4.7%
Tmem146	0.15728598	0.05	32.6%
Tmem147	52.93476	3.17	6.0%
Tmem149	1.3415646	0.26	19.6%
Tmem14a	5.728178	0.33	5.7%
Tmem14c	21.61912	1.41	6.5%
Tmem150	3.515236	0.33	9.3%
Tmem151	52.67644	4.42	8.4%
Tmem154	0.027912756	0.01	40.6%
Tmem157	17.27948	0.92	5.3%
Tmem158	4.015484	0.46	11.4%
Tmem159	1.961512	0.22	11.4%
Tmem160	22.96856	2.76	12.0%
Tmem161a	7.883132	0.43	5.5%
Tmem161b	7.554554	0.33	4.3%
Tmem162	0.03537672	0.03	93.3%
Tmem163	34.16312	2.80	8.2%
Tmem164	4.675904	0.41	8.8%
Tmem165	14.1266	1.37	9.7%
Tmem166	0.419749	0.09	22.5%
Tmem167	16.1203	0.87	5.4%
Tmem168	3.887698	0.30	7.8%
Tmem169	13.84786	0.84	6.1%
Tmem16a	0.12430872	0.04	29.6%
Tmem16b	0.04425908	0.02	56.3%
Tmem16c	0.2711762	0.12	43.2%
Tmem16d	9.330558	0.33	3.6%

Tmem16e	0.2831648	0.04	13.5%
Tmem16f	9.033254	0.62	6.9%
Tmem16g	0.00579106	0.01	150.9%
Tmem16h	15.53554	1.03	6.6%
Tmem16j	0.0043887	0.01	137.0%
Tmem16k	14.03248	0.52	3.7%
Tmem17	4.246456	0.91	21.5%
Tmem170	0.7465104	0.09	11.8%
Tmem171	0.00461426	0.01	223.6%
Tmem173	0.2068036	0.05	24.6%
Tmem174	0	0.00	
Tmem175	19.10182	4.36	22.8%
Tmem176a	10.450798	1.91	18.3%
Tmem176b	18.03568	2.47	13.7%
Tmem177	1.859724	0.11	5.9%
Tmem178	7.589758	0.31	4.1%
Tmem179	37.32004	1.48	4.0%
Tmem18	14.95028	0.63	4.2%
Tmem180	11.59478	0.84	7.3%
Tmem181	8.0368	0.56	7.0%
Tmem182	0.03900622	0.03	76.0%
Tmem183a	20.99936	0.82	3.9%
Tmem184a	0.108637	0.06	55.7%
Tmem184b	13.94994	0.50	3.6%
Tmem185b	6.393576	0.45	7.1%
Tmem186	6.172158	0.39	6.3%
Tmem188	2.11467	0.37	17.4%
Tmem189	14.38922	0.45	3.1%
Tmem19	12.61548	0.58	4.6%
Tmem2	3.47619	0.35	10.1%
Tmem20	0.5081964	0.02	4.2%
Tmem24	11.07526	0.65	5.9%
Tmem25	16.70496	1.55	9.3%
Tmem26	0.03975376	0.01	29.2%
Tmem27	0.0180565	0.03	154.1%
Tmem28	26.19832	1.35	5.1%
Tmem30a	117.6128	4.64	3.9%
Tmem30b	0.1522226	0.02	14.0%
Tmem32	18.86744	1.58	8.4%
Tmem33	29.38782	0.88	3.0%
Tmem34	24.89886	0.41	1.6%
Tmem35	26.01388	1.16	4.5%
Tmem37	0.3746766	0.05	14.2%
Tmem38a	26.54518	2.16	8.1%
Tmem38b	2.406878	0.43	17.9%
Tmem39a	4.796672	0.44	9.1%
Tmem39b	3.434672	0.35	10.3%

Tmem4	15.02082	1.12	7.5%
Tmem40	0.10262874	0.06	61.9%
Tmem41a	8.20866	0.61	7.5%
Tmem41b	11.59978	0.45	3.9%
Tmem42	4.904624	0.63	12.9%
Tmem43	14.01602	0.39	2.8%
Tmem44	4.044652	0.31	7.6%
Tmem45a	1.1051192	0.47	42.1%
Tmem45b	0.03720318	0.03	82.1%
Tmem46	2.890494	0.29	10.0%
Tmem47	36.98134	0.37	1.0%
Tmem48	6.65934	0.43	6.5%
Tmem49	26.55726	2.14	8.1%
Tmem5	14.07058	0.43	3.0%
Tmem50a	37.17318	1.59	4.3%
Tmem50b	43.12128	2.23	5.2%
Tmem51	0.456734	0.33	72.1%
Tmem52	0.02013468	0.02	99.5%
Tmem53	3.082398	0.38	12.5%
Tmem54	0.12295208	0.26	211.5%
Tmem55a	30.49778	1.77	5.8%
Tmem55b	31.3881	2.74	8.7%
Tmem56	12.638	0.65	5.2%
Tmem57	19.421	0.39	2.0%
Tmem58	21.90522	2.87	13.1%
Tmem59	95.81434	4.12	4.3%
Tmem59l	79.8842	2.95	3.7%
Tmem60	17.54494	0.60	3.4%
Tmem62	11.73342	0.53	4.5%
Tmem63a	0.5067894	0.12	23.7%
Tmem63b	51.67516	1.08	2.1%
Tmem63c	7.28094	0.11	1.5%
Tmem64	8.131264	0.60	7.4%
Tmem65	48.74952	0.43	0.9%
Tmem66	132.1126	3.10	2.3%
Tmem67	3.104174	0.27	8.6%
Tmem68	12.8309	1.09	8.5%
Tmem69	4.613212	0.27	5.8%
Tmem70	21.2711	1.38	6.5%
Tmem71	0.00636952	0.01	138.1%
Tmem74	2.4199	0.29	11.8%
Tmem77	12.3795	0.63	5.1%
Tmem79	0.380747	0.10	25.9%
Tmem8	2.861288	0.27	9.3%
Tmem80	6.407704	0.37	5.7%
Tmem81	0.2923524	0.08	27.5%
Tmem82	0.0466229	0.02	47.2%

Tmem85	67.51778	4.04	6.0%
Tmem86a	3.29638	0.40	12.3%
Tmem86b	1.953762	0.31	15.6%
Tmem87a	19.7673	1.16	5.9%
Tmem87b	14.43842	0.43	3.0%
Tmem88	0.1648672	0.02	13.2%
Tmem9	51.99374	2.42	4.7%
Tmem90a	2.84401	0.32	11.3%
Tmem91	33.67138	3.05	9.0%
Tmem92	0	0.00	
Tmem93	21.38388	1.02	4.8%
Tmem97	7.428564	0.44	5.9%
Tmem98	1.707184	0.29	16.9%
Tmem9b	24.2713	1.24	5.1%
Tmepai	6.007826	0.38	6.3%
Tmf1	10.111354	0.39	3.9%
Tmhs	1.839126	0.14	7.4%
Tmie	0.3432082	0.09	25.5%
Tmigd1	0	0.00	
Tmlhe	0	0.00	
Tmod1	0.642746	0.16	25.7%
Tmod2	64.03606	2.82	4.4%
Tmod3	4.902612	0.24	4.9%
Tmod4	0.0352293	0.03	79.1%
Tmpo	11.12714	0.66	5.9%
Tmprss11a	0	0.00	
Tmprss11b	0.00319138	0.01	223.6%
Tmprss11c	0	0.00	
Tmprss11d	0.00802118	0.01	143.3%
Tmprss11e	0.00492812	0.01	140.4%
Tmprss11f	0	0.00	
Tmprss12	0	0.00	
Tmprss13	0.0023692	0.01	223.6%
Tmprss2	0.3783644	0.11	29.5%
Tmprss3	0	0.00	
Tmprss4	0.00250802	0.01	223.6%
Tmprss5	0.014340798	0.01	70.8%
Tmprss6	0.018253216	0.01	74.8%
Tmprss7	0.08292006	0.04	45.9%
Tmprss8	0	0.00	
Tmprss9	0.07298846	0.03	34.6%
Tmsb10	67.15032	7.96	11.9%
Tmsb4x	454.668	44.17	9.7%
Tmtc1	2.95156	0.62	21.1%
Tmtc2	2.366364	0.13	5.5%
Tmtc3	2.33245	0.18	7.8%
Tmtc4	9.07245	0.28	3.1%

Tmub1	10.458684	0.88	8.4%
Tmub2	21.98432	1.41	6.4%
Tnc	0.9444064	0.21	22.6%
Tnf	0	0.00	
Tnfaip1	13.1917	0.55	4.2%
Tnfaip2	0.07969102	0.01	12.5%
Tnfaip3	0.8778108	0.11	12.4%
Tnfaip6	0.17271066	0.09	53.3%
Tnfaip8	0.51063696	0.45	88.5%
Tnfaip8l1	1.331116	0.16	12.2%
Tnfaip8l2	0.02672798	0.02	65.4%
Tnfaip8l3	1.348828	0.12	8.8%
Tnfrh1	0.06963434	0.02	27.5%
Tnfrsf10b	0.8480028	0.08	8.9%
Tnfrsf11a	0.5504776	0.05	8.3%
Tnfrsf11b	0.07948632	0.02	23.6%
Tnfrsf12a	5.925086	0.75	12.6%
Tnfrsf13b	0	0.00	
Tnfrsf13c	0.06175658	0.03	49.8%
Tnfrsf14	0.0219213	0.02	103.9%
Tnfrsf17	0.01333562	0.02	137.0%
Tnfrsf18	0.3434676	0.08	24.6%
Tnfrsf19	3.473972	0.43	12.3%
Tnfrsf1a	2.320246	0.47	20.5%
Tnfrsf1b	0.06384004	0.04	56.7%
Tnfrsf21	34.8089	1.61	4.6%
Tnfrsf22	0.7327458	0.13	17.8%
Tnfrsf23	0.08241562	0.02	29.3%
Tnfrsf25	0.4292964	0.18	41.5%
Tnfrsf26	0.02648274	0.03	123.5%
Tnfrsf4	0.08930288	0.03	37.5%
Tnfrsf8	0.01361572	0.01	60.3%
Tnfrsf9	0.01098948	0.01	94.8%
Tnfsf10	0.00877262	0.01	99.0%
Tnfsf11	0.03446992	0.02	59.2%
Tnfsf12	0.3472042	0.20	58.4%
Tnfsf12-tnfsf13	0.3852752	0.19	50.2%
Tnfsf13	0.30931824	0.24	78.0%
Tnfsf13b	0.10016662	0.04	37.7%
Tnfsf14	0.00381374	0.01	223.6%
Tnfsf15	0	0.00	
Tnfsf18	0.00814134	0.01	137.0%
Tnfsf4	0.00378874	0.01	223.6%
Tnfsf5ip1	9.995326	0.68	6.8%
Tnfsf8	0.00664528	0.01	138.6%
Tnfsf9	0.4620204	0.18	38.0%
Tnip1	8.621208	0.36	4.2%

Tnip2	2.828858	0.35	12.5%
Tnip3	0.00292136	0.01	223.6%
Tnk1	0.5666704	0.68	119.4%
Tnk2	22.34662	2.49	11.1%
Tnks	67.56442	2.68	4.0%
Tnks1bp1	5.653474	0.14	2.5%
Tnks2	74.44796	4.00	5.4%
Tnmd	0.536183	0.13	23.9%
Tnn	0.003997242	0.00	99.1%
Tnnc1	1.268702	0.11	9.0%
Tnnc2	0.03557562	0.06	174.9%
Tnni1	0.03545908	0.04	113.8%
Tnni2	0.00800834	0.02	223.6%
Tnni3	0.05297104	0.04	77.2%
Tnni3k	0.0041926	0.01	223.6%
Tnnt1	0.590163	0.15	25.8%
Tnnt2	0.1661678	0.06	37.0%
Tnnt3	0.02981278	0.01	35.3%
Tnp1	0.01620264	0.04	223.6%
Tnp2	0.00723626	0.02	223.6%
Tnpo1	10.265828	0.73	7.1%
Tnpo2	43.59248	0.92	2.1%
Tnpo3	20.16312	0.83	4.1%
Tnr	6.024824	0.77	12.9%
Tnrc15	17.50894	1.06	6.1%
Tnrc18	15.09456	2.57	17.0%
Tnrc4	5.790098	0.55	9.5%
Tnrc5	15.97922	0.64	4.0%
Tnrc6a	20.05888	0.68	3.4%
Tnrc6b	8.90796	0.69	7.7%
Tnrc6c	9.733018	0.74	7.6%
Tns1	0.6335114	0.48	76.2%
Tns3	3.536408	0.14	3.9%
Tns4	0.033161916	0.02	59.0%
Tnxb	0.02120676	0.01	32.1%
Tob1	6.787006	0.65	9.5%
Tob2	3.703008	0.21	5.8%
Toe1	4.56399	0.29	6.4%
Tollip	51.30236	0.89	1.7%
Tom1	0.05522414	0.06	102.3%
Tom1l1	4.195654	0.58	13.9%
Tom1l2	45.88278	2.25	4.9%
Tom40	0.1240372	0.02	14.2%
Tomm20	11.976448	1.55	12.9%
Tomm22	35.82968	3.81	10.6%
Tomm34	11.58194	0.73	6.3%
Tomm40	27.61156	1.10	4.0%

Tomm40l	11.83664	1.13	9.5%
Tomm7	34.2158	1.37	4.0%
Tomm70a	81.43522	2.37	2.9%
Top1	19.56012	0.41	2.1%
Top1mt	6.008926	0.31	5.2%
Top2a	2.60646	0.66	25.3%
Top2b	32.7154	2.43	7.4%
Top3a	1.216459	0.22	18.3%
Top3b	8.637002	0.66	7.6%
Topbp1	4.19674	0.11	2.6%
Topors	6.138168	0.14	2.3%
Tor1a	10.082292	0.78	7.7%
Tor1aip1	3.588846	0.68	19.0%
Tor1aip2	4.956356	0.79	16.0%
Tor1b	5.312916	0.32	6.1%
Tor2a	4.537528	0.96	21.1%
Tor3a	0.327972	0.11	34.4%
Tox	9.166734	0.25	2.7%
Tox2	9.343396	0.64	6.9%
Tox3	9.13428	0.42	4.6%
Tox4	13.4656	0.84	6.3%
Tp73l	0.004358746	0.00	102.1%
Tpbg	0.9637032	0.44	45.6%
Tpbpa	0	0.00	
Tpbpb	0	0.00	
Tpcn1	3.648572	0.59	16.2%
Tpcn2	0.546179	0.09	15.7%
Tpd52	12.59072	1.02	8.1%
Tpd52l1	5.128518	0.18	3.4%
Tpd52l2	18.51762	0.24	1.3%
Tph1	0	0.00	
Tph2	0.02332482	0.03	120.9%
Tpi1	126.6574	14.70	11.6%
Tpk1	2.388016	0.21	8.7%
Tpm1	24.07234	1.79	7.4%
Tpm2	0.983258	0.15	14.8%
Tpm3	17.1969	1.81	10.5%
Tpm4	7.45311	0.77	10.3%
Tpmt	2.568956	0.30	11.6%
Tpo	0.042172	0.03	65.2%
Tpp1	13.22514	0.37	2.8%
Tpp2	24.49248	0.72	3.0%
Tppp	41.77644	4.18	10.0%
Tppp3	44.80066	4.18	9.3%
Tpr	19.80798	1.33	6.7%
Tprkb	22.29798	0.79	3.5%
Tpsab1	0	0.00	

Tpsb2	0.00445704	0.01	223.6%
Tpsg1	0.52600502	1.04	198.1%
Tpst1	8.180056	0.38	4.6%
Tpst2	5.27773	0.32	6.1%
Tpte	0	0.00	
Tpte2	13.13054	0.84	6.4%
Tpx2	1.2214264	0.23	18.5%
Tra2a	26.49018	0.59	2.2%
Trabd	11.19396	0.76	6.8%
TRAD/RAD51L3	1.67876326	0.92	54.6%
Tradd	2.76469	0.12	4.4%
TRADV13-4	0	0.00	
TRADV14D-3	0	0.00	
TRADV15D-2	0	0.00	
TRADV16D	0	0.00	
TRADV4-4	0	0.00	
Traf1	0.01737454	0.01	75.2%
Traf2	1.705006	0.23	13.7%
Traf3	33.16236	1.75	5.3%
Traf3ip1	4.763886	0.25	5.3%
Traf3ip2	0.6123546	0.15	24.9%
Traf3ip3	0.0678047	0.04	57.9%
traf4	3.683506	0.61	16.6%
Traf4	3.142216	0.29	9.3%
Traf5	0.06953908	0.05	73.0%
Traf6	1.83601	0.18	9.6%
Traf7	11.86666	0.79	6.6%
Trafd1	11.8695	0.31	2.6%
Traip	0.4155278	0.08	20.0%
Traits	0.05113434	0.02	41.0%
Trak1	17.8373	0.73	4.1%
Trak2	14.34738	0.32	2.2%
Tram1	16.0187	0.75	4.7%
Tram1l1	17.97494	1.70	9.5%
Tram2	0.5525832	0.13	22.7%
Trap1	22.48538	1.14	5.1%
Trap1a	4.177794	0.53	12.8%
Trappc1	26.6873	2.67	10.0%
Trappc2	10.048906	0.86	8.6%
Trappc2l	37.69234	2.16	5.7%
Trappc3	35.49528	1.83	5.1%
Trappc4	30.57432	3.74	12.2%
Trappc5	10.048232	0.25	2.5%
Trappc6a	9.150966	1.15	12.6%
Trappc6b	22.69396	1.48	6.5%
Trat1	0.00778774	0.02	223.6%
TRAV10	0	0.00	

TRAV10D	0	0.00	
Trav11	0	0.00	
TRAV12-3	0	0.00	
TRAV13D-2	0	0.00	
TRAV14-1	0	0.00	
TRAV14-2	0	0.00	
TRAV17	0	0.00	
TRAV19	0.0120389	0.03	223.6%
TRAV2	0	0.00	
TRAV3-3	0.04069168	0.06	141.6%
TRAV4-2	0	0.00	
TRAV4D-3	0.01727194	0.04	223.6%
TRAV4D-4	0	0.00	
TRAV5D-4	0	0.00	
TRAV6-3	0	0.00	
TRAV6-6	0	0.00	
TRAV6-7	0	0.00	
TRAV6D-6	0	0.00	
TRAV6D-7	0	0.00	
TRAV7-5	0	0.00	
TRAV7D-4	0	0.00	
TRAV8D-2	0	0.00	
Trcg1	0.00222456	0.00	223.6%
Trdmt1	1.824282	0.10	5.4%
Trdn	0.0107846	0.02	139.3%
TRDV2-2	0	0.00	
TRDV5	0.01264386	0.03	223.6%
Treh	0	0.00	
Trem1	0	0.00	
Trem2	0	0.00	
Trem3	0.0420014	0.03	72.8%
Treml1	0	0.00	
Treml2	0	0.00	
Treml4	0.00614078	0.01	137.3%
Trerf1	3.197108	0.25	7.7%
Trex1	3.679	0.34	9.3%
Trex2	0.00538842	0.01	223.6%
Trf	0.0354257	0.02	67.8%
Trfr2	1.50873	0.13	8.9%
Trh	0.0522966	0.03	54.0%
Trhde	5.212906	0.24	4.6%
Trhr	6.875692	0.30	4.3%
Trhr2	0.6124098	0.16	25.4%
Triad2	6.790884	1.19	17.6%
Triap1	12.7782	1.13	8.9%
Trib1	0.6453394	0.07	10.2%
Trib2	33.33986	4.12	12.4%

Trib3	1.447368	0.22	15.3%
Trim10	0.00210974	0.00	223.6%
Trim11	4.39688	0.41	9.3%
Trim12	0	0.00	
Trim13	3.526794	0.58	16.6%
Trim14	0.03824044	0.04	99.5%
Trim15	0.2065726	0.08	40.7%
Trim16	0.0597758	0.04	74.4%
Trim17	2.302268	0.15	6.6%
Trim2	38.31034	0.76	2.0%
Trim21	0.4460392	0.06	13.6%
Trim23	23.96152	1.23	5.1%
Trim24	8.57886	0.45	5.3%
Trim25	0.4263052	0.17	40.9%
Trim26	9.097582	0.34	3.7%
Trim27	8.667246	0.52	6.0%
Trim28	43.82176	1.61	3.7%
Trim29	0.00844538	0.01	147.2%
Trim3	33.68254	0.71	2.1%
Trim30	0.04727242	0.02	51.0%
Trim30/Rpt1	0.05321956	0.06	118.5%
Trim31	0	0.00	
Trim32	66.39734	2.42	3.6%
Trim33	11.17256	0.12	1.1%
Trim34	0.5448426	0.13	24.3%
Trim35	60.72684	5.52	9.1%
Trim36	7.048722	0.91	12.9%
Trim37	64.17198	2.08	3.2%
Trim38	0.0038202	0.01	223.6%
Trim39	6.653914	0.34	5.1%
Trim40	0.0063301	0.01	138.0%
Trim41	9.348676	0.69	7.4%
Trim42	0.00533738	0.01	138.9%
Trim44	67.12064	1.21	1.8%
Trim45	3.167626	0.15	4.9%
Trim46	20.8175	0.70	3.3%
Trim47	0.0773463	0.03	44.3%
Trim50	0	0.00	
Trim52	0	0.00	
Trim54	0	0.00	
Trim55	0.00261666	0.01	223.6%
Trim56	0.44826	0.15	32.9%
Trim58	0	0.00	
Trim59	2.143332	0.28	13.0%
Trim6	0.03731204	0.04	107.4%
Trim60	0	0.00	
Trim61	0.0030801	0.01	223.6%

Trim62	10.80408	0.89	8.2%
Trim63	0.02090794	0.03	152.6%
Trim65	0.6830804	0.10	14.6%
Trim66	3.23752	0.25	7.6%
Trim67	2.42393	0.64	26.4%
Trim68	0.5711296	0.07	11.6%
Trim69	0.02990954	0.01	34.8%
Trim7	0.6347948	0.11	17.2%
Trim71	0.2438752	0.06	23.7%
Trim72	0.2948814	0.09	31.3%
Trim75	0.02389412	0.01	44.7%
Trim8	20.52196	1.09	5.3%
Trim9	31.87758	1.31	4.1%
Triml1	0.00548836	0.01	137.4%
Trio	18.90992	0.69	3.6%
Triobp	3.483114	0.63	18.0%
Trip10	1.2058624	0.28	22.9%
Trip11	4.411556	0.25	5.7%
Trip12	29.01262	1.27	4.4%
Trip13	0.5062656	0.11	22.2%
Trip4	6.466178	0.29	4.5%
Trip6	2.227298	0.25	11.2%
Trit1	3.6352	0.23	6.3%
trkC	3.29577	0.60	18.3%
TrkC	2.73269	0.49	17.8%
Trm6	0.464935	0.23	48.5%
Trmt1	18.59804	0.66	3.5%
Trmt11	1.902496	0.17	8.8%
Trmt12	1.97642	0.16	8.0%
Trmt5	5.137158	0.23	4.5%
Trmt6	4.611426	0.59	12.8%
Trmu	8.608306	0.33	3.9%
Trnt1	19.24758	0.60	3.1%
Tro	56.36142	2.08	3.7%
Troap	0.3059386	0.08	27.6%
Trove2	7.109494	0.61	8.6%
Trp2	1.1588854	0.27	23.0%
trp3	0.9684892	0.24	25.2%
trp4	0.355844	0.13	36.4%
Trp53	6.050204	0.53	8.7%
Trp53bp1	36.61072	3.79	10.3%
Trp53bp2	2.165814	0.15	6.8%
Trp53i11	4.537008	0.22	4.9%
Trp53i13	1.480706	0.26	17.3%
Trp53inp1	8.204874	1.04	12.7%
Trp53inp2	37.64058	1.26	3.4%
Trp53rk	0.4582276	0.10	22.5%

Trp63	0.004859878	0.01	134.0%
Trp73	0.04176648	0.02	50.7%
Trpa1	0.1797998	0.04	20.7%
Trpc1	5.304378	0.48	9.0%
TRPC1A-Beta	0.3275028	0.18	56.2%
Trpc2	2.215638	0.47	21.1%
Trpc3	6.707216	0.26	3.9%
Trpc4	1.1842508	0.18	14.9%
Trpc4ap	63.87188	1.60	2.5%
Trpc5	4.78455	0.60	12.5%
Trpc6	0.5896774	0.08	12.9%
Trpc7	0.4396146	0.11	25.8%
Trpd52l3	0	0.00	
Trpm1	0.01495088	0.03	177.4%
Trpm2	2.214952	0.39	17.6%
Trpm3	5.717262	1.06	18.6%
Trpm4	1.985354	0.23	11.4%
Trpm5	0.1571732	0.04	24.3%
Trpm6	0.165189	0.04	26.6%
Trpm7	8.56702	0.85	10.0%
Trpm8	0.02790062	0.01	36.9%
Trps1	1.272818	0.11	8.6%
Trpt1	3.262164	0.98	30.1%
Trpv1	0.01942688	0.01	36.6%
Trpv2	1.979186	0.24	12.3%
Trpv3	0.02687264	0.02	86.8%
Trpv4	0.03064849	0.02	62.5%
Trpv5	0	0.00	
Trpv6	0.2572652	0.02	9.4%
Trrap	7.721108	0.38	4.9%
Trspap1	4.986692	0.40	8.0%
Trub1	6.798426	0.50	7.4%
Trub2	9.520362	0.20	2.1%
Try10	0	0.00	
Try4	0	0.00	
Tsarg1	0	0.00	
Tsarg7	1.1867796	0.28	23.5%
Tsc1	13.49994	0.87	6.5%
Tsc2	17.98832	1.01	5.6%
Tsc22d1	141.39	10.24	7.2%
Tsc22d2	10.197622	0.38	3.7%
Tsc22d3	40.4051	2.78	6.9%
Tsc22d4	5.114984	0.58	11.3%
tsec-1	1.302024	0.17	13.3%
Tsen2	4.998768	0.33	6.6%
Tsen34	23.1162	1.44	6.2%
Tsen54	2.865992	0.36	12.7%

Tsfm	9.266498	1.06	11.4%
Tsg101	13.34522	0.84	6.3%
Tsg118	0.8525044	1.13	132.8%
Tsga10	2.393566	0.16	6.5%
Tsga10ip	0.0093703	0.02	223.6%
Tsga13	0	0.00	
Tsga14	2.296238	0.30	13.3%
Tsga8	0	0.00	
Tshb	0.058666	0.05	91.9%
Tshr	0.3345522	0.06	16.5%
Tshz1	10.045288	0.78	7.7%
Tshz2	35.0198	1.65	4.7%
Tshz3	8.126212	0.35	4.3%
Tsku	1.049387	0.21	20.1%
Tslp	0.27042584	0.12	43.1%
Tsn	37.3477	2.20	5.9%
Tsnax	0	0.00	
Tsnaxip1	0.02775878	0.02	82.5%
Tsp50	0.01476744	0.02	147.7%
Tspan1	0.13640082	0.05	34.6%
Tspan10	0	0.00	
Tspan11	0.7982	0.26	32.2%
Tspan12	13.04348	0.49	3.8%
Tspan13	105.4022	9.97	9.5%
Tspan14	5.628136	0.37	6.6%
Tspan15	0.621947	0.11	18.4%
Tspan17	40.15558	2.22	5.5%
Tspan18	8.677734	0.87	10.0%
Tspan2	18.60582	1.28	6.9%
Tspan3	149.7024	2.86	1.9%
tspan-3	0.11990326	0.05	41.3%
Tspan31	25.88182	1.52	5.9%
Tspan32	0.01141314	0.02	156.5%
Tspan33	3.629782	0.40	11.1%
Tspan4	11.212142	1.39	12.4%
Tspan5	42.20816	1.59	3.8%
Tspan6	29.36962	3.14	10.7%
Tspan7	366.6254	13.17	3.6%
Tspan8	1.555014	0.56	35.9%
Tspan9	16.04348	1.31	8.2%
Tspo	0.2764746	0.12	43.6%
Tspyl1	56.17282	2.29	4.1%
Tspyl2	45.92146	3.28	7.1%
Tspyl3	15.07334	0.21	1.4%
Tspyl4	205.8738	1.63	0.8%
Tspyl5	11.56284	0.82	7.1%
Tsr1	10.6479	0.69	6.5%

Tsr2	17.65986	0.61	3.4%
Tssc1	12.0632	0.94	7.8%
Tssc4	16.65386	0.94	5.6%
Tssk1	0.05136374	0.04	84.3%
Tssk2	0.02188314	0.02	88.0%
Tssk3	0.00460128	0.01	223.6%
Tssk4	0.0353777	0.04	114.4%
Tssk5	0	0.00	
Tssk6	1.640776	0.16	9.7%
Tst	1.993006	0.65	32.8%
Tsta3	10.87488	0.82	7.5%
Tsx	0.05610492	0.05	86.1%
Ttbk1	11.0364	0.41	3.7%
Ttbk2	23.84082	1.43	6.0%
Ttc1	19.5004	0.57	2.9%
Ttc12	0.1747892	0.01	5.4%
Ttc13	14.49112	1.26	8.7%
Ttc14	13.27944	1.56	11.7%
Ttc15	17.97238	0.65	3.6%
Ttc16	1.1912432	0.42	35.2%
Ttc17	10.253982	0.41	4.0%
Ttc19	19.85828	0.43	2.2%
Ttc21a	0.2023128	0.03	14.2%
Ttc21b	4.289178	0.23	5.5%
Ttc22	0.1741612	0.04	23.8%
Ttc23	0.7698802	0.08	10.1%
Ttc24	0	0.00	
Ttc25	0.1304426	0.03	23.8%
Ttc26	1.109752	0.06	5.2%
Ttc27	3.449526	0.12	3.4%
Ttc28	3.449258	0.27	7.7%
Ttc29	0.02147202	0.02	112.3%
Ttc3	292.7848	14.47	4.9%
Ttc30a1	1.473808	0.08	5.6%
Ttc30a2	0.10767924	0.05	45.6%
Ttc30b	2.989726	0.09	2.9%
Ttc32	1.161327	0.25	21.9%
Ttc33	25.19252	1.80	7.2%
Ttc35	28.54636	1.23	4.3%
Ttc4	15.86364	0.77	4.9%
Ttc5	4.230578	0.33	7.8%
Ttc7	0.5174594	0.06	11.5%
Ttc8	6.115968	0.37	6.1%
Ttc9	23.19594	0.79	3.4%
Ttc9b	52.83264	3.45	6.5%
Ttc9c	18.38152	0.15	0.8%
Ttf1	1.297828	0.16	12.0%

Ttf2	0.5125912	0.02	4.7%
Ttk	0.592753	0.15	25.8%
Ttl	20.03702	1.13	5.6%
Ttll1	25.2477	1.57	6.2%
Ttll10	0.008798808	0.01	98.5%
Ttll11	8.132924	0.46	5.7%
Ttll12	12.1843	0.60	4.9%
Ttll13	0.4155774	0.10	23.6%
Ttll2	0.01224086	0.01	94.7%
Ttll3	1.653722	0.47	28.4%
Ttll4	1.235528	0.11	9.2%
Ttll5	2.664768	0.19	7.3%
Ttll6	0.03447588	0.01	34.7%
Ttll7s	17.93536	1.51	8.4%
Ttll9	1.034099	0.11	10.4%
Ttn	0.032392416	0.03	87.6%
Ttpa	0.5425434	0.12	22.6%
Ttr	0.8390596	0.30	36.2%
Ttrap	6.419602	0.30	4.6%
Ttyh1	25.55486	1.08	4.2%
Ttyh2	1.07224	0.07	6.2%
Ttyh3	41.65634	2.51	6.0%
Tub	18.91156	0.63	3.3%
Tuba1a	436.6058	61.60	14.1%
Tuba1b	74.13428	5.07	6.8%
Tuba1c	12.61432	1.91	15.2%
Tuba3a	0.00300838	0.01	223.6%
Tuba3b	0.01610538	0.02	144.2%
Tuba4a	172.941	7.62	4.4%
Tuba8	0.2613252	0.05	20.6%
Tubal3	0	0.00	
Tubb1	0.00394516	0.01	223.6%
Tubb2a	191.475	10.95	5.7%
Tubb2b	135.3444	30.68	22.7%
Tubb2c	89.65968	3.67	4.1%
Tubb3	404.603	40.50	10.0%
Tubb4	115.179	6.38	5.5%
Tubb5	401.332	40.69	10.1%
Tubb6	3.212962	0.20	6.2%
Tubd1	1.540066	0.14	9.4%
Tube1	1.16935	0.18	15.7%
Tubg1	17.3413	0.81	4.7%
tubg1	0.445157	0.17	39.0%
Tubg2	10.511728	0.65	6.1%
Tubgcp2	14.15368	0.33	2.3%
Tubgcp3	11.5052	0.75	6.5%
Tubgcp4	11.367866	4.63	40.7%

Tubgcp5	10.33909	0.56	5.4%
Tubgcp6	4.795648	0.46	9.5%
Tuc1	1.926006	0.45	23.4%
Tufm	2.709524	0.18	6.6%
Tuft1	1.68789	0.13	7.6%
Tulp1	0.003834792	0.01	147.3%
Tulp2	0.01937224	0.02	122.7%
Tulp3	3.864006	0.36	9.3%
Tulp4	0.14813	0.02	16.6%
Tusc1	3.189408	0.41	12.7%
Tusc2	21.91886	1.35	6.2%
Tusc3	106.7678	4.84	4.5%
Tusc4	13.54832	0.45	3.3%
Tusc5	0.12442502	0.03	23.7%
Tut1	7.329342	0.30	4.0%
Twf1	11.83892	0.67	5.6%
Twf2	6.537182	0.55	8.4%
Twist1	5.464374	0.43	7.8%
Twist2	0.4302992	0.13	29.3%
Twistnb	5.175536	0.73	14.1%
Twsg1	9.386024	0.86	9.1%
Txk	0	0.00	
Txlna	8.402522	0.28	3.4%
Txlnb	0.1669358	0.01	4.9%
Txlng	1.11553416	0.66	59.5%
Txn1	57.48372	1.87	3.2%
Txn2	40.1292	1.24	3.1%
Txndc1	49.5309	2.19	4.4%
Txndc10	20.42234	1.71	8.4%
Txndc11	8.670316	0.36	4.1%
Txndc12	14.75654	0.57	3.9%
Txndc13	117.0414	7.59	6.5%
Txndc14	47.17986	1.22	2.6%
Txndc15	26.01738	0.97	3.7%
Txndc16	20.32532	0.71	3.5%
Txndc17	14.67814	0.95	6.5%
Txndc2	0.0178474	0.01	34.8%
Txndc3	0	0.00	
Txndc4	16.29506	1.07	6.6%
Txndc5	28.57452	1.59	5.5%
Txndc8	0	0.00	
Txndc9	5.30657	0.19	3.6%
Txnip	6.490402	1.61	24.8%
Txnl1	51.13752	3.24	6.3%
Txnl4	0.11585174	0.04	35.5%
Txnl4a	34.37806	0.85	2.5%
Txnl4b	5.849436	0.35	6.0%

Txnrd1	22.37384	1.57	7.0%
TXNRD1	1.525925	1.10	72.0%
Txnrd2	5.573918	0.55	9.8%
Txnrd3	3.34548	0.33	9.7%
Tyk2	1.472374	0.10	7.0%
Tyki	9.890874	0.21	2.2%
Tyms	0.5007214	0.04	7.8%
Tyr	0	0.00	
Tyro3	9.330962	1.08	11.6%
Tyrobp	0	0.00	
Tyrp1	0.04089552	0.04	98.7%
Tysnd1	6.063448	0.72	11.9%
Tyw1	3.618276	0.37	10.4%
Tyw3	2.878804	0.28	9.9%
U07657	0	0.00	
U07879	0	0.00	
U09772	0	0.00	
U20366	0.01751002	0.01	29.2%
U20367	0.01601312	0.01	31.9%
U20369	0.02600664	0.01	36.4%
U2af1	0.9390914	0.12	12.9%
U2af1l4	3.972816	0.85	21.5%
U2af2	18.78584	0.73	3.9%
U2af26	1.938226	0.62	32.2%
U46068	0.02247648	0.02	72.6%
U46581	0	0.00	
U69888	2.886842	0.44	15.1%
U76754	0.07101258	0.08	113.9%
U80893	1.79179	0.29	16.0%
U83174	5.845582	0.69	11.8%
U83175	1.17743146	1.08	92.1%
U86728	0	0.00	
U89428	0.06955178	0.06	93.1%
U89738	0	0.00	
U96411	0.00288205	0.00	154.5%
Uaca	0.9765034	0.16	16.7%
Uap1	16.17074	1.34	8.3%
Uap1l1	6.44768	0.28	4.3%
Uba52	0.4175114	0.08	19.8%
Ubac1	17.13294	1.19	6.9%
Ubac2	10.378574	0.33	3.2%
Ubadc1	9.591802	1.86	19.4%
Ubap1	12.57318	0.36	2.9%
Ubap2	16.0682	1.27	7.9%
Ubap2l	37.95496	1.67	4.4%
Ubash3a	0.001760086	0.00	223.6%
Ubb	36.30852	1.10	3.0%

Ubc	134.70702	48.22	35.8%
Ubc13	45.29074	1.45	3.2%
Ubce7ip1	0.0296481	0.01	18.2%
ubcM4	0.11567488	0.08	69.4%
Ubd	0	0.00	
Ube1c	26.82464	1.15	4.3%
Ube1dc1	20.46936	1.09	5.3%
Ube1l	0.13013894	0.08	64.8%
Ube1l2	9.386666	0.68	7.3%
Ube1x	109.6626	4.61	4.2%
Ube1y1	0	0.00	
Ube2a	15.30754	1.00	6.5%
Ube2b	53.80872	1.92	3.6%
Ube2c	1.833116	0.33	17.8%
Ube2d1	26.13942	2.05	7.8%
Ube2d2	22.00418	1.11	5.1%
Ube2d3	81.92084	3.17	3.9%
Ube2e1	23.42454	1.73	7.4%
Ube2e2	42.47912	1.22	2.9%
Ube2e3	24.091	1.57	6.5%
Ube2f	9.902542	0.47	4.8%
Ube2g1	18.78506	0.58	3.1%
Ube2g2	11.59806	0.50	4.3%
Ube2h	17.4352	0.82	4.7%
Ube2i	11.65278	0.36	3.1%
Ube2j1	58.0584	2.41	4.1%
Ube2j2	3.5213	0.05	1.5%
Ube2l3	39.71518	3.05	7.7%
Ube2l6	0.7574538	0.18	23.9%
Ube2m	57.50184	2.29	4.0%
Ube2n	3.90464	1.55	39.6%
Ube2o	31.50228	0.37	1.2%
Ube2q1	23.96878	0.63	2.6%
Ube2q2	11.1808	0.70	6.3%
Ube2r2	20.92708	1.05	5.0%
Ube2s	1.979128	0.38	19.1%
Ube2t	0.9325606	0.16	17.4%
Ube2u	0.00574536	0.01	223.6%
Ube2v1	1.806434	0.12	6.8%
Ube2v2	11.00088	0.40	3.6%
Ube2w	34.84324	2.02	5.8%
Ube2z	30.01228	1.37	4.6%
Ube3a	29.59422	1.96	6.6%
Ube3b	15.50818	0.64	4.1%
Ube3c	32.36502	1.05	3.2%
Ube4a	19.38642	0.91	4.7%
Ube4b	29.28742	0.73	2.5%

Ubfd1	60.6828	1.02	1.7%
Ubiad1	5.355596	0.25	4.7%
Ubie	0.144506	0.03	19.9%
Ubl3	47.20678	2.02	4.3%
Ubl4	10.64613	0.52	4.9%
Ubl4b	0	0.00	
Ubl5	10.989752	1.90	17.3%
Ubl7	25.63334	2.02	7.9%
Ublcp1	11.69405	1.23	10.5%
Ubn1	6.594346	0.57	8.6%
Ubox5	1.657722	0.22	13.4%
Ubp1	17.38678	0.44	2.5%
Ubqln1	66.35584	1.52	2.3%
Ubqln2	84.35866	2.52	3.0%
Ubqln3	0	0.00	
Ubqln4	20.93172	1.80	8.6%
Ubqlnl	0	0.00	
Ubr1	11.29466	0.52	4.6%
Ubr2	11.012384	0.75	6.8%
Ubr5	18.49596	0.84	4.5%
Ubtd1	5.182958	0.53	10.3%
Ubtd2	8.661912	0.23	2.7%
Ubtf	18.21696	0.93	5.1%
Ubxd1	20.1391	1.01	5.0%
Ubxd2	20.3434	0.95	4.7%
Ubxd3	0.02294442	0.01	61.1%
Ubxd4	21.50176	1.15	5.4%
Ubxd5	0.8577618	0.09	10.8%
Ubxd6	5.18797	0.39	7.6%
Ubxd7	11.252434	0.81	7.2%
Ubxd8	17.03404	0.88	5.2%
Uchl1	437.6558	45.20	10.3%
Uchl3	6.48497	0.38	5.9%
uchl3	0.6899384	0.29	42.1%
Uchl4	11.4935	1.71	14.9%
Uchl5	22.40512	1.59	7.1%
Uchl5ip	6.323924	0.65	10.2%
Uck1	6.054414	0.22	3.6%
Uck2	14.43374	1.53	10.6%
Uckl1	5.821524	0.30	5.1%
Ucn	0	0.00	
Ucn3	0.00307428	0.01	223.6%
Ucp1	0.05996326	0.05	89.3%
Ucp2	1.287544	0.22	17.0%
Ucp3	0.014336	0.01	82.4%
UCP4	0.749485	0.28	37.1%
Uevld	0.9158712	0.05	5.3%

Ufc1	26.698	1.94	7.3%
Ufd1l	25.54314	0.57	2.2%
Ufd2	2.0717244	1.35	65.3%
Ufm1	15.38658	0.34	2.2%
Ugcg	47.70548	2.54	5.3%
Ugcgl1	11.81204	0.57	4.8%
Ugcgl2	8.609052	0.97	11.3%
Ugdh	7.25193	0.50	6.9%
Ugp2	19.46158	0.23	1.2%
Ugt1a1	0.034326964	0.04	115.4%
Ugt1a10	0.03921778	0.02	52.1%
Ugt1a2	0.03987368	0.01	37.4%
Ugt1a5	0.04501746	0.03	57.3%
Ugt1a6a	0.07658792	0.04	58.7%
Ugt1a6b	0.028262833	0.06	200.1%
Ugt1a7c	0.020357268	0.02	94.1%
Ugt1a9	0.038785912	0.03	74.0%
Ugt2a1	0.00805598	0.01	147.7%
Ugt2a2	0.001709932	0.00	175.0%
Ugt2a3	0	0.00	
Ugt2b1	0	0.00	
Ugt2b34	0.6098184	0.22	36.0%
Ugt2b35	0.01581356	0.02	110.6%
Ugt2b36	0	0.00	
Ugt2b37	0	0.00	
Ugt2b38	0	0.00	
Ugt2b5	0	0.00	
Ugt3a1	0	0.00	
Ugt3a2	0.01194228	0.01	93.9%
Ugt8a	2.36017	0.32	13.5%
Uhmk1	32.55584	4.25	13.0%
Uhrf1	0.5832264	0.12	20.2%
Uhrf2	12.27926	0.58	4.7%
Uimc1	2.957718	0.18	5.9%
Ulbp1	0	0.00	
Ulk1	20.923	1.12	5.4%
Ulk2	22.86218	1.33	5.8%
Ulk3	4.380418	0.40	9.1%
Ulk4	2.166066	0.46	21.1%
Umod	0	0.00	
Umodl1	0.003571004	0.00	92.7%
Umps	3.862358	0.36	9.4%
Unc119	21.27256	1.03	4.9%
Unc119b	4.668906	0.19	4.1%
Unc13a	29.83048	1.78	6.0%
Unc13b	1.67191	0.12	7.0%
Unc13c	2.663264	0.14	5.2%

Unc13d	0.015655868	0.01	42.9%
Unc45a	11.83794	1.00	8.5%
Unc45b	0.155059	0.02	15.5%
Unc50	21.93394	1.03	4.7%
Unc5a	6.323092	0.62	9.8%
Unc5b	0.7745398	0.04	5.1%
Unc5c	3.266648	0.53	16.3%
Unc5cl	0.0054165	0.01	137.0%
Unc5d	6.49692	0.84	13.0%
Unc84a	12.19342	0.37	3.0%
Unc84b	5.201608	1.19	22.8%
Unc93a	0.04751908	0.05	96.2%
Unc93b1	0.50588	0.19	37.5%
Uncx4.1	1.733118	0.12	7.1%
Ung	1.1815564	0.12	10.5%
Unk	4.639708	0.12	2.7%
Unkl	2.567302	0.09	3.4%
unknown	5.951656	0.85	14.4%
Uox	0.00757924	0.01	138.7%
Upb1	0.00362262	0.01	223.6%
Upf1	11.09208	0.35	3.2%
Upf2	5.380836	0.50	9.2%
Upf3a	8.404512	0.98	11.7%
Upf3b	7.464148	0.18	2.4%
Upk1a	1.1779	0.11	9.4%
Upk1b	0.03229752	0.02	55.0%
Upk2	0.01800834	0.03	144.2%
Upk3a	0.00956834	0.01	137.3%
Upk3b	0.00314952	0.01	223.6%
Upp1	0.300649	0.10	33.7%
Upp2	3.170456	0.61	19.3%
Uprt	3.985034	0.72	18.1%
Uqcc	13.38158	0.61	4.6%
Uqcr	28.75108	11.21	39.0%
Uqcrb	21.6928	1.83	8.4%
Uqcrc1	125.2694	3.33	2.7%
Uqcrc2	112.794	2.90	2.6%
Uqcrfs1	79.4401	1.53	1.9%
Uqcrh	99.49852	5.62	5.7%
Uqcrq	42.90238	4.46	10.4%
Urm1	16.39658	0.74	4.5%
Uroc1	0	0.00	
Urod	10.407078	0.69	6.6%
Uros	9.32846	0.65	6.9%
Use1	16.03776	1.07	6.7%
Use1l	1.69638	0.73	43.0%
Usf1	11.15062	0.73	6.6%

Usf2	10.67034	0.66	6.2%
Ush1c	0.00711936	0.01	140.3%
Ush1g	0.04598434	0.02	39.3%
Ush2a	0.003109862	0.00	43.9%
Ush2A	0.000811052	0.00	223.6%
Ushbp1	0.03377748	0.03	76.9%
Usmg5	6.27224	1.48	23.6%
Uso1	22.09938	0.87	4.0%
Usp1	2.03952	0.08	4.1%
Usp10	18.87898	0.98	5.2%
Usp11	79.09078	2.31	2.9%
Usp12	9.598872	0.56	5.8%
Usp13	5.464572	0.26	4.8%
Usp14	55.17842	1.67	3.0%
Usp15	21.45404	1.57	7.3%
Usp16	5.639536	0.36	6.5%
Usp17	0	0.00	
Usp18	0.1908292	0.07	36.6%
Usp19	25.16124	0.97	3.8%
Usp2	1.973228	0.19	9.4%
Usp20	13.80224	0.51	3.7%
Usp21	10.234842	0.35	3.4%
Usp22	113.2174	5.01	4.4%
Usp24	1.969286	0.49	25.1%
Usp25	4.283314	0.14	3.2%
Usp26	0.051590096	0.06	117.2%
Usp28	4.66334	0.24	5.1%
Usp29	23.47852	0.70	3.0%
Usp3	4.366492	0.18	4.0%
Usp30	10.120074	0.20	1.9%
Usp32	20.6535	1.27	6.2%
Usp33	38.92616	1.71	4.4%
Usp34	8.04906	1.50	18.7%
Usp35	1.455802	0.32	21.8%
Usp36	0.2064442	0.06	28.7%
Usp37	4.656716	0.65	14.0%
Usp38	7.65071	0.40	5.3%
Usp39	11.53092	0.73	6.3%
Usp4	16.464	0.66	4.0%
Usp40	5.338686	2.34	43.8%
Usp42	4.667882	0.15	3.2%
Usp43	0.12513714	0.04	32.9%
Usp44	0.06358294	0.04	68.5%
Usp45	5.445494	0.69	12.6%
Usp46	20.36862	0.48	2.3%
Usp47	21.51112	0.99	4.6%
Usp48	12.51566	1.04	8.3%

Usp49	2.80665	0.05	1.7%
Usp5	62.82878	3.19	5.1%
Usp50	0.00412792	0.01	223.6%
Usp52	4.016074	0.19	4.7%
Usp53	2.30439	0.55	23.7%
Usp54	4.335116	0.20	4.6%
Usp6nl	6.999496	0.62	8.9%
Usp7	21.51338	0.57	2.7%
Usp8	21.82242	0.73	3.3%
Usp9x	36.61486	2.92	8.0%
Usp9y	0	0.00	
Uspl1	13.23574	0.53	4.0%
Ust	5.286698	0.29	5.5%
Utf1	0.01120832	0.02	145.2%
Utp11l	8.309694	0.65	7.9%
Utp14a	2.86603	0.14	4.9%
Utp14b	1.277574	0.10	7.8%
Utp15	6.953982	0.30	4.3%
Utp18	9.293944	0.80	8.6%
Utp20	1.77241	0.20	11.2%
Utp3	12.24988	0.85	6.9%
Utp6	8.135574	0.31	3.8%
Utrn	9.451224	1.80	19.1%
Uts2	0.01074432	0.02	223.6%
Uts2d	0.00838652	0.02	223.6%
Uts2r	0.02506908	0.02	72.3%
Utx	7.333004	0.41	5.6%
Uty	0.05748234	0.01	19.6%
Uvrag	7.498892	0.33	4.4%
Uxs1	4.37464	0.20	4.5%
Uxt	0.6942498	0.19	27.2%
V00757	0	0.00	
V00821	0	0.00	
V1ra1	0.00347076	0.01	223.6%
V1ra2	0	0.00	
V1ra3	0	0.00	
V1ra4	0	0.00	
V1ra5	0	0.00	
V1ra6	0	0.00	
V1ra7	0	0.00	
V1ra8	0	0.00	
V1ra9	0	0.00	
V1rb1	0	0.00	
V1rb10	0	0.00	
V1rb2	0	0.00	
V1rb3	0	0.00	
V1rb4	0	0.00	

V1rb7	0	0.00	
V1rb8	0	0.00	
V1rb9	0	0.00	
V1rc1	0	0.00	
V1rc10	0	0.00	
V1rc11	0	0.00	
V1rc12	0	0.00	
V1rc13	0	0.00	
V1rc14	0	0.00	
V1rc15	0.00920752	0.01	140.2%
V1rc16	0	0.00	
V1rc17	0	0.00	
V1rc18	0.01159456	0.03	223.6%
V1rc19	0	0.00	
V1rc2	0.0083736	0.02	223.6%
V1rc20	0	0.00	
V1rc21	0.03286282	0.02	64.0%
V1rc22	0	0.00	
V1rc23	0	0.00	
V1rc24	0	0.00	
V1rc26	0	0.00	
V1rc27	0	0.00	
V1rc28	0	0.00	
V1rc29	0	0.00	
V1rc3	0	0.00	
V1rc30	0	0.00	
V1rc32	0	0.00	
V1rc33	0	0.00	
V1rc5	0	0.00	
V1rc6	0.04838838	0.03	68.5%
V1rc7	0.00915542	0.02	223.6%
V1rc8	0	0.00	
V1rd1	0	0.00	
V1rd10	0	0.00	
V1rd11	0	0.00	
V1rd12	0	0.00	
V1rd13	0	0.00	
V1rd14	0	0.00	
V1rd15	0.06348308	0.04	63.5%
V1rd16	0	0.00	
V1rd17	0	0.00	
V1rd19	0	0.00	
V1rd20	0	0.00	
V1rd21	0	0.00	
V1rd22	0	0.00	
V1rd3	0	0.00	
V1rd4	0	0.00	

V1rd6	0	0.00	
V1rd7	0	0.00	
V1rd8	0	0.00	
V1rd9	0	0.00	
V1re1	0	0.00	
V1re10	0	0.00	
V1re11	0	0.00	
V1re12	0.00749186	0.02	223.6%
V1re13	0	0.00	
V1re2	0	0.00	
V1re3	0.00849974	0.02	223.6%
V1re4	0	0.00	
V1re5	0	0.00	
V1re6	0	0.00	
V1re7	0	0.00	
V1re8	0	0.00	
V1re9	0	0.00	
V1rf1	0	0.00	
V1rf2	0	0.00	
V1rf3	0	0.00	
V1rf4	0	0.00	
V1rf5	0.01062248	0.02	223.6%
V1rg1	0	0.00	
V1rg11	0	0.00	
V1rg12	0	0.00	
V1rg2	0	0.00	
V1rg3	0	0.00	
V1rg4	0	0.00	
V1rg5	0	0.00	
V1rg6	0	0.00	
V1rg7	0	0.00	
V1rg8	0	0.00	
V1rg9	0	0.00	
V1rh1	0	0.00	
V1rh10	0	0.00	
V1rh11	0	0.00	
V1rh12	0	0.00	
V1rh13	0	0.00	
V1rh14	0	0.00	
V1rh16	0	0.00	
V1rh17	0	0.00	
V1rh18	0	0.00	
V1rh2	0	0.00	
V1rh20	0	0.00	
V1rh21	0	0.00	
V1rh3	0	0.00	
V1rh4	0	0.00	

V1rh5	0	0.00	
V1rh6	0	0.00	
V1rh7	0.0106853	0.02	223.6%
V1rh8	0	0.00	
V1rh9	0	0.00	
V1ri1	0	0.00	
V1ri10	0	0.00	
V1ri2	0	0.00	
V1ri3	0	0.00	
V1ri4	0	0.00	
V1ri5	0	0.00	
V1ri6	0	0.00	
V1ri7	0	0.00	
V1ri8	0	0.00	
V1ri9	0	0.00	
V1rj2	0	0.00	
V1rj3	0.00775982	0.02	223.6%
V1rk1	0	0.00	
V1rl1	0	0.00	
V2r1	0	0.00	
V2r10	0.0031411	0.01	223.6%
V2r14	0	0.00	
V2r15	0	0.00	
V2r16	0	0.00	
V2r1b	0	0.00	
V2r3	0	0.00	
V2r4	0	0.00	
V2r6	0	0.00	
V2r7	0	0.00	
V2r9	0	0.00	
Vac14	14.36956	0.97	6.8%
Vamp1	275.8298	24.92	9.0%
Vamp2	465.013	13.02	2.8%
Vamp3	8.292938	0.39	4.7%
Vamp4	40.41122	2.40	5.9%
Vamp5	0.06133594	0.03	41.3%
Vamp8	1.73806	0.35	19.9%
Vangl1	2.918168	0.17	6.0%
Vangl2	5.375374	0.32	6.0%
Vapa	87.76182	5.95	6.8%
Vapb	40.53112	0.64	1.6%
variable_group_of_2-cell-stage	0	0.00	
Varp	0.4370972	0.06	13.2%
Vars	10.60044	0.28	2.6%
Vars2	5.830022	0.33	5.6%
Vash1	14.38056	1.34	9.3%
Vash2	7.006526	0.60	8.5%

Vasn	1.809942	0.17	9.2%
Vasp	6.2996	0.37	5.9%
Vat1	72.7591	9.33	12.8%
Vav1	0.003945328	0.01	137.8%
Vav2	5.932858	0.17	2.9%
Vav3	4.853952	0.37	7.5%
Vax1	0.02993064	0.02	81.5%
Vax2	7.244216	0.44	6.1%
Vb7Db1Jb1.1Cb	0	0.00	
Vbp1	48.21078	1.94	4.0%
Vcam1	1.0792316	0.20	18.8%
Vcan	2.574624	0.45	17.5%
Vcl	11.91186	0.80	6.7%
Vcp	28.09544	0.69	2.5%
Vcpip1	15.24186	0.87	5.7%
Vdac1	223.268	6.29	2.8%
Vdac2	55.9	2.85	5.1%
Vdac3	30.9861	1.72	5.6%
Vdp	1.2694224	0.39	31.1%
Vdr	0.023174894	0.02	95.3%
Vdu1	0.5409594	0.11	20.9%
Vdu2	4.177554	0.92	21.9%
Vegf	0.6269844	0.32	50.9%
Vegfa	18.35842	2.34	12.8%
Vegfb	51.9783	1.56	3.0%
Vegfc	0.9932498	0.11	11.4%
Veph1	0.01856228	0.01	45.9%
Veph-A	0	0.00	
vesl-2	0.1576676	0.03	20.4%
Vezf1	9.88239	0.29	2.9%
Vezt	16.35148	0.73	4.4%
Vgcnl1	0.03795432	0.01	33.1%
Vgf	88.58952	21.71	24.5%
Vgll1	0	0.00	
Vgll2	0.353854	0.05	13.9%
Vgll4	4.242794	0.40	9.4%
Vhlh	13.62216	0.50	3.7%
Vil1	0.012247834	0.01	54.6%
Vil2	6.418488	0.78	12.1%
Vill	0.0878375	0.06	65.5%
Vim	55.98586	4.76	8.5%
Vip	0.4445112	0.10	21.4%
Vipr1	0.0470575	0.02	45.1%
Vipr2	1.124036	0.04	3.5%
Vit	0.3548348	0.09	26.1%
Vkorc1	11.44052	0.65	5.7%
Vkorc1l1	4.48641	0.55	12.2%

Vldlr	12.98976	0.63	4.8%
Vmn2r118	0.0031764	0.01	223.6%
Vmn2r12	0	0.00	
Vmn2r13	0.0029676	0.01	223.6%
Vmn2r2	0	0.00	
Vmn2r27	0	0.00	
Vmn2r55	0	0.00	
Vmn2r76	0	0.00	
Vmn2r81	0	0.00	
Vmn2r92	0	0.00	
Vmn2r95	0	0.00	
Vmo1	0.0295468	0.07	223.6%
Vnn1	0.136497	0.06	41.0%
Vnn3	0	0.00	
Vprbp	5.805004	0.28	4.8%
Vpreb1	0	0.00	
Vpreb2	0	0.00	
Vpreb3	0.03797988	0.05	120.7%
Vps11	20.79378	0.78	3.7%
Vps13a	4.918516	0.87	17.8%
Vps13b	4.989438	0.17	3.4%
Vps13c	10.645792	0.70	6.6%
Vps13d	7.005156	0.32	4.6%
Vps16	16.16396	0.49	3.0%
Vps18	12.02246	0.42	3.5%
Vps24	23.75836	0.53	2.2%
Vps25	26.09584	2.64	10.1%
Vps26a	17.30962	0.60	3.5%
Vps26b	33.40982	1.96	5.9%
Vps28	62.16236	4.24	6.8%
Vps29	62.03986	2.43	3.9%
Vps33a	21.03104	0.54	2.6%
Vps33b	7.88477	0.38	4.8%
Vps35	87.60372	3.30	3.8%
Vps36	10.859282	1.22	11.2%
Vps37a	14.58788	0.86	5.9%
Vps37b	3.969658	0.24	5.9%
Vps37c	13.5618	0.97	7.2%
Vps37d	9.723382	0.42	4.3%
Vps39	20.15938	1.89	9.4%
Vps41	44.56144	0.93	2.1%
Vps45	8.60767	0.48	5.5%
Vps4a	37.16916	2.06	5.5%
Vps4b	9.023154	0.72	7.9%
Vps52	21.67236	0.77	3.6%
Vps53	19.84804	1.66	8.4%
Vps54	4.84296	0.27	5.5%

Vps72	8.41334	0.33	3.9%
Vps8	8.329636	0.25	3.0%
VR1	0	0.00	
VR10	0	0.00	
VR16	0	0.00	
VR3	0	0.00	
VRHO	0.4489148	0.20	45.1%
Vrk1	2.927454	0.18	6.1%
Vrk2	0.5534112	0.32	57.5%
Vrk3	5.689336	0.57	10.0%
Vsig1	0.2142534	0.06	28.8%
Vsig2	0.3770334	0.11	28.5%
Vsig4	0	0.00	
Vsig8	0.06960038	0.03	45.3%
Vsnl1	214.5166	17.98	8.4%
Vstm2a	68.75896	1.97	2.9%
Vstm2b	21.66644	2.02	9.3%
Vstm2l	37.92038	4.64	12.2%
Vsx1	0	0.00	
Vsx2	0.031714116	0.03	91.8%
Vta1	23.08906	2.03	8.8%
Vtcn1	0.02047442	0.02	104.0%
Vti1a	17.40298	0.87	5.0%
Vti1b	47.99228	1.92	4.0%
Vti1-rp2	3.549006	0.58	16.4%
Vtn	0.12970512	0.07	50.7%
Vwa1	1.1375488	0.22	19.6%
Vwa2	0.01921324	0.01	60.5%
Vwa3a	0.1151193	0.02	21.4%
Vwc2	9.80989	0.59	6.1%
Vwce	0.03813786	0.02	40.8%
Vwf	0.12501202	0.10	78.9%
Wac	22.9302	0.84	3.7%
Wap	0	0.00	
Wapal	5.640092	0.36	6.3%
Wars	49.96418	3.14	6.3%
Wars2	2.423632	0.16	6.7%
Was	0	0.00	
Wasf1	35.78292	3.30	9.2%
Wasf2	2.537352	0.29	11.3%
Wasf3	23.86528	0.77	3.2%
Wasl	21.89136	1.77	8.1%
Wbp1	12.01962	0.86	7.1%
Wbp11	27.52926	1.96	7.1%
Wbp2	120.068	4.96	4.1%
Wbp2nl	0.11031234	0.04	32.3%
Wbp4	11.16472	0.70	6.2%

Wbp5	39.01698	2.03	5.2%
Wbp7	8.914688	0.72	8.1%
Wbscr16	3.452616	0.41	11.9%
Wbscr17	18.41526	0.47	2.5%
Wbscr18	14.15018	1.42	10.0%
Wbscr21	10.837938	0.88	8.2%
Wbscr22	7.321478	1.02	14.0%
Wbscr25	0.036166	0.02	54.1%
Wbscr27	2.243862	0.29	12.7%
Wbscr28	0	0.00	
wdc146	8.36038	1.89	22.6%
Wdfy1	12.45238	0.53	4.2%
Wdfy2	3.535858	0.49	13.8%
Wdfy3	9.084732	0.96	10.6%
Wdfy4	0.01351554	0.01	94.8%
Wdhd1	1.387812	0.19	13.9%
wdp103	0.27103934	0.14	49.9%
Wdr1	65.84888	5.42	8.2%
Wdr12	4.828512	0.29	6.1%
Wdr13	38.26946	1.49	3.9%
Wdr16	0.11434518	0.02	14.7%
Wdr17	4.196974	0.37	8.8%
Wdr18	17.04152	0.28	1.7%
Wdr19	4.108866	0.34	8.4%
Wdr20a	6.042428	0.46	7.7%
Wdr20b	0.3613288	0.06	17.6%
Wdr21	6.411532	0.39	6.0%
Wdr22	17.93634	0.41	2.3%
Wdr23	16.31692	0.55	3.4%
Wdr24	7.19001	0.20	2.8%
Wdr26	29.58908	1.34	4.5%
Wdr27	0.3135844	0.10	30.6%
Wdr3	5.266072	0.24	4.5%
Wdr31	1.706476	0.33	19.1%
Wdr32	6.593334	0.26	4.0%
Wdr33	14.7755	1.34	9.0%
Wdr34	12.18472	1.05	8.6%
Wdr35	8.650286	0.50	5.8%
Wdr36	11.7194	1.04	8.9%
Wdr37	43.86426	0.94	2.2%
Wdr38	0.3272359	0.53	161.7%
Wdr4	4.407952	0.77	17.5%
wdr4	1.6374962	0.66	40.4%
Wdr40a	18.03936	0.71	3.9%
Wdr40b	12.73162	0.40	3.2%
Wdr40c	0.2329258	0.04	17.4%
Wdr41	10.285662	0.65	6.3%

Wdr42a	23.38902	0.75	3.2%
Wdr43	6.881716	0.22	3.1%
Wdr44	5.673342	0.38	6.7%
Wdr45	10.712508	0.55	5.1%
Wdr45l	18.34826	0.50	2.7%
Wdr46	2.575834	0.31	12.1%
Wdr47	32.95158	1.00	3.0%
Wdr48	17.65404	1.03	5.8%
Wdr5	12.6585	0.94	7.4%
Wdr51a	0.7934724	0.23	29.2%
Wdr51b	1.473732	0.05	3.6%
Wdr53	4.06301	0.19	4.6%
Wdr54	2.354692	0.38	16.3%
Wdr55	2.1068008	1.20	56.9%
Wdr57	7.557758	0.90	12.0%
Wdr59	6.690964	0.30	4.5%
Wdr5b	1.592976	0.22	13.9%
Wdr6	128.273	2.77	2.2%
Wdr60	1.838928	0.21	11.2%
Wdr61	10.80032	0.55	5.1%
Wdr62	0.841287	0.14	16.3%
Wdr63	0.03125008	0.03	80.8%
Wdr66	0.06805618	0.03	47.3%
Wdr67	2.106536	0.30	14.1%
Wdr68	124.0076	3.14	2.5%
Wdr69	0.00651998	0.01	144.4%
Wdr7	33.5239	1.77	5.3%
Wdr70	14.13202	0.50	3.6%
Wdr72	0.15901562	0.05	29.8%
Wdr73	5.335086	0.33	6.2%
Wdr74	14.68192	1.38	9.4%
Wdr75	12.13968	0.41	3.4%
Wdr76	0.8784196	0.07	7.7%
Wdr77	23.39018	0.73	3.1%
Wdr78	0.7531162	0.11	14.3%
Wdr79	1.71649	0.08	4.7%
Wdr8	5.515414	0.32	5.8%
Wdr81	4.564354	0.35	7.7%
Wdr82	44.2364	1.20	2.7%
Wdr85	5.305196	0.34	6.5%
Wdr86	0.00254906	0.01	223.6%
Wdr9	1.95901	0.55	27.9%
Wdr91	6.967782	0.49	7.0%
Wdr92	3.07267	0.17	5.5%
Wdsof1	14.51114	0.97	6.7%
Wdsub1	4.331394	0.53	12.2%
Wdtc1	14.43594	0.52	3.6%

Wdvcf	1.701536	0.28	16.2%
Wee1	5.954604	1.09	18.3%
Wee2	0.1282689	0.04	32.3%
Wfdc1	0.09990888	0.06	62.9%
Wfdc10	0	0.00	
Wfdc12	0	0.00	
Wfdc13	0.00787968	0.02	223.6%
Wfdc15a	0	0.00	
Wfdc15b	0	0.00	
Wfdc16	0	0.00	
Wfdc2	0.01203306	0.02	137.8%
Wfdc3	0.2034232	0.05	25.5%
Wfdc5	0.00410014	0.01	223.6%
Wfdc6a	0	0.00	
Wfdc6b	0	0.00	
Wfdc8	0	0.00	
Wfikkn2	0.10379868	0.05	45.6%
Wfs1	12.74068	0.83	6.5%
Whdc1	5.044216	0.37	7.4%
Whrn	2.648764	0.37	14.1%
Whsc1	14.23678	1.46	10.3%
Whsc1l1	18.74374	0.64	3.4%
Whsc2	6.356612	0.37	5.9%
Wibg	8.164376	1.11	13.6%
Wif1	0.2806918	0.09	33.1%
Wipf1	1.2297342	0.25	20.3%
Wipf2	10.297546	0.64	6.2%
Wipi1	4.044272	0.55	13.5%
Wipi2	24.84602	0.80	3.2%
Wisp1	1.575128	0.24	14.9%
Wisp2	0.01218574	0.01	92.3%
Wiz	13.50134	1.13	8.4%
wiz	2.388518	0.49	20.6%
Wnk1	17.4528	0.82	4.7%
Wnk2	18.23706	0.94	5.2%
Wnk3	6.789224	0.31	4.6%
Wnk4	1.34341	0.15	11.5%
Wnt1	0.1390022	0.02	15.6%
Wnt10a	0.04020144	0.03	83.2%
Wnt10b	0.3756858	0.06	14.8%
Wnt11	1.163226	0.09	8.0%
Wnt16	0.03119072	0.01	32.2%
Wnt2	0.1494487	0.03	22.6%
Wnt2b	0.8263238	0.16	19.0%
Wnt3	1.0438896	0.08	7.6%
Wnt3a	0.03409204	0.02	69.1%
Wnt4	0.371919	0.15	39.5%

Wnt5a	5.928294	0.32	5.3%
Wnt5b	1.0947698	0.16	15.1%
Wnt6	0.09076616	0.05	55.4%
Wnt7a	1.17628	0.13	10.8%
Wnt7b	2.2021	0.09	4.1%
Wnt8a	0	0.00	
Wnt8b	0.01548772	0.02	102.3%
Wnt9a	0.8502486	0.18	21.7%
Wnt9b	0.06122182	0.01	24.1%
Wrb	34.2284	2.96	8.6%
Wrn	1.96345	0.22	11.3%
Wrnip1	15.61448	0.37	2.4%
Wsb1	46.9056	3.84	8.2%
Wsb2	77.51266	7.27	9.4%
Wscd1	21.89526	1.33	6.1%
Wscd2	5.13147	0.26	5.1%
Wt1	0.1254842	0.02	12.1%
Wtap	7.765352	0.18	2.3%
wtap	6.78844	1.81	26.7%
Wtip	2.133196	0.29	13.7%
Wwc1	6.473942	0.35	5.4%
Wwc2	4.75956	0.39	8.3%
Wwox	7.961336	0.56	7.1%
Wwp1	8.116358	0.43	5.3%
Wwp2	9.498076	0.45	4.7%
Wwtr1	3.258186	0.42	12.9%
X02930	0	0.00	
X02938	0	0.00	
X02969	0	0.00	
X05012	0	0.00	
X05732	0	0.00	
X05737	0	0.00	
X06772	0	0.00	
X56719	0	0.00	
X56722	0	0.00	
X57330	0	0.00	
X57780	3967.778	516.64	13.0%
X63927	0	0.00	
X65506	0	0.00	
X83328	16.7091	3.37	20.2%
X95399	13.43002	1.21	9.0%
X98059	0	0.00	
X99384	3.782804	0.25	6.6%
Xab1	11.50394	0.67	5.8%
Xab2	9.84302	0.26	2.6%
Xbp1	24.53302	1.86	7.6%
Xcl1	0	0.00	

Xcr1	0.03722016	0.02	42.9%
Xdh	0.10521326	0.04	39.5%
Xirp1	0.005160378	0.00	87.7%
Xirp2	0.002982258	0.00	105.2%
Xk	4.684694	0.19	4.1%
Xkr4	7.245094	1.08	14.8%
Xkr5	0.1640028	0.08	50.2%
Xkr6	1.219808	0.09	7.6%
Xkr7	2.27157	0.26	11.4%
Xkr8	2.342978	0.09	3.8%
Xkr9	0.01044018	0.01	137.4%
Xkrx	0.2828498	0.09	31.8%
Xlp	0.00488672	0.01	223.6%
Xlr	0	0.00	
Xlr3a	0.07527492	0.05	61.3%
Xlr3b	0.03084886	0.03	113.4%
Xlr3c	0.00557974	0.01	223.6%
Xlr4a	0.00488822	0.01	223.6%
Xlr4b	0	0.00	
Xlr4c	0	0.00	
Xlr5a	0	0.00	
Xlr5c	0	0.00	
Xmr	0	0.00	
Xpa	4.645994	0.46	9.8%
Xpc	3.174502	0.14	4.4%
Xpnpep1	26.406	2.59	9.8%
Xpnpep2	0.03114576	0.02	62.6%
Xpnpep3	3.881756	0.43	11.0%
Xpo1	26.60946	0.99	3.7%
Xpo4	1.0997074	0.32	29.5%
Xpo5	12.17652	0.30	2.4%
Xpo6	24.68586	0.80	3.3%
Xpo7	24.46016	1.40	5.7%
Xpot	9.616604	0.28	2.9%
Xpr1	25.67796	1.71	6.7%
Xrcc1	5.11192	0.30	5.9%
Xrcc2	1.629848	0.16	9.6%
Xrcc3	2.127918	0.13	6.2%
Xrcc4	4.25746	0.63	14.9%
Xrcc5	6.854326	0.47	6.8%
Xrcc6	5.218734	0.29	5.6%
Xrcc6bp1	4.049536	0.32	7.9%
Xrn1	2.68381	0.27	10.0%
Xrn2	12.27978	0.63	5.1%
Xrra1	0.1311611	0.03	24.6%
Xtrp3s1	0.00294016	0.01	223.6%
Xylb	2.142332	0.18	8.3%

Xylt1	2.118676	0.02	1.0%
Xylt2	7.42209	0.43	5.8%
Y08382	0.070238	0.16	223.6%
YAF2	24.9425	1.18	4.7%
Yaf2	20.12414	0.94	4.7%
Yap1	3.58742	0.57	16.0%
Yars	34.40504	4.19	12.2%
Yars2	7.642404	0.50	6.5%
Ybx1	44.19376	7.70	17.4%
Ybx2	1.072644	0.16	14.8%
Yeats2	4.368448	0.10	2.4%
Yeats4	13.3683	1.10	8.2%
Yes1	3.361828	0.25	7.3%
Yif1a	12.1285	0.88	7.2%
Yif1b	23.84366	2.01	8.4%
Yipf1	14.76808	1.51	10.2%
Yipf2	11.187438	3.11	27.8%
Yipf3	21.2581	0.92	4.3%
Yipf4	23.89308	1.46	6.1%
Yipf5	16.59644	1.18	7.1%
Yipf6	16.0498	0.84	5.2%
Yipf7	0.04952104	0.06	127.7%
Ykt6	32.35676	1.39	4.3%
Ylpm1	12.56656	0.71	5.6%
Yme1l1	30.82084	1.14	3.7%
Yod1	2.220064	0.21	9.5%
Ypel1	3.679838	0.60	16.3%
Ypel2	4.218852	0.89	21.1%
Ypel3	60.25436	6.96	11.6%
Ypel4	7.598432	2.16	28.4%
Ypel5	35.65222	3.47	9.7%
Yrdc	7.225262	0.79	11.0%
Ythdc1	19.50628	1.32	6.8%
Ythdf1	23.49106	1.22	5.2%
Ythdf2	13.6857	0.91	6.6%
Ythdf3	18.63784	0.44	2.4%
Ywhab	269.2066	10.33	3.8%
Ywhae	384.6576	22.27	5.8%
Ywhag	489.3	21.13	4.3%
Ywhah	219.048	11.44	5.2%
Ywhaq	33.09686	2.52	7.6%
Ywhaz	154.725	12.05	7.8%
Yy1	9.389506	0.30	3.2%
Z12226	0	0.00	
Z22835	0	0.00	
Z22845	0	0.00	
Z83810	0.05538694	0.06	102.5%

Z83812	0.01554172	0.03	223.6%
Z83815	0	0.00	
Zac1	2.1481872	1.01	46.9%
Zadh1	8.982994	0.93	10.4%
Zadh2	10.60903	0.52	4.9%
Zan	0.012777628	0.00	33.7%
zap	4.376178	0.46	10.6%
Zap70	0.0942612	0.05	50.9%
Zar1	0.03349704	0.03	80.4%
Zbed3	4.341016	0.33	7.6%
Zbed4	0.5247532	0.03	5.0%
Zbp1	0.0033348	0.01	223.6%
Zbtb1	3.676104	0.46	12.6%
Zbtb11	0.05492426	0.02	40.1%
Zbtb12	3.206564	0.54	16.9%
Zbtb16	2.011002	0.17	8.6%
Zbtb17	4.128458	0.29	7.1%
Zbtb2	1.97398	0.17	8.8%
Zbtb20	0.2093392	0.03	14.5%
Zbtb22	7.586534	0.48	6.3%
Zbtb24	3.633654	0.24	6.5%
Zbtb25	5.124052	0.38	7.4%
Zbtb26	2.974934	0.27	9.1%
Zbtb3	1.812062	0.13	6.9%
Zbtb32	0.2538004	0.08	30.8%
Zbtb33	7.938808	0.22	2.8%
Zbtb34	2.194154	0.14	6.2%
Zbtb37	0.9632714	0.17	17.7%
Zbtb38	2.640534	0.37	13.9%
Zbtb39	2.323526	0.10	4.3%
Zbtb4	9.732502	0.28	2.9%
Zbtb40	1.344448	0.09	6.8%
Zbtb41	6.053964	0.54	8.9%
Zbtb43	3.624724	0.20	5.5%
Zbtb44	2.689462	0.50	18.7%
Zbtb45	10.242232	0.47	4.6%
Zbtb46	3.335732	0.35	10.5%
Zbtb48	2.521226	0.15	5.8%
Zbtb5	3.906196	0.19	4.9%
Zbtb6	4.434204	0.36	8.2%
Zbtb7a	9.409608	1.43	15.2%
Zbtb7b	1.828084	0.17	9.5%
Zbtb7c	3.40833	0.34	10.0%
Zbtb8	2.363048	0.06	2.7%
Zbtb8os	1.495674	0.29	19.6%
Zbtb9	4.300362	0.51	11.9%
Zc3h10	4.449974	0.36	8.1%

Zc3h11a	17.31246	1.49	8.6%
Zc3h12a	0.08744096	0.05	54.1%
Zc3h12b	1.473026	0.10	6.6%
Zc3h12c	2.576216	0.29	11.4%
Zc3h12d	0.01403942	0.01	68.5%
Zc3h13	11.21062	0.21	1.9%
Zc3h14	19.43924	0.67	3.5%
Zc3h15	39.15388	1.45	3.7%
Zc3h3	0.9892168	0.11	10.9%
Zc3h6	3.093864	0.05	1.8%
Zc3h7a	7.119956	0.81	11.4%
Zc3h7b	35.92676	0.93	2.6%
Zc3h8	2.440712	0.35	14.3%
Zc3hav1	1.171132	0.21	18.2%
Zc3hav1l	1.88493	0.22	11.6%
Zc3hc1	11.94242	0.62	5.2%
Zcchc10	3.947852	0.85	21.4%
Zcchc11	6.192146	0.33	5.3%
Zcchc12	76.163	5.89	7.7%
Zcchc14	6.650544	0.32	4.8%
Zcchc16	1.229334	0.09	7.1%
Zcchc17	24.0494	2.42	10.0%
Zcchc18	131.3996	1.86	1.4%
Zcchc2	3.508604	0.22	6.2%
Zcchc3	2.209744	0.20	9.0%
Zcchc4	2.396236	0.25	10.4%
Zcchc5	0.6705586	0.27	40.6%
Zcchc6	8.119656	0.58	7.1%
Zcchc7	0.6165102	0.17	27.8%
Zcchc8	6.072058	0.18	3.0%
Zcchc9	3.535204	0.27	7.5%
Zcrb1	13.99624	1.29	9.2%
Zcwpw1	1.831506	0.22	11.9%
Zcwpw2	0.9321156	0.17	17.8%
Zcyto7	0.00463512	0.01	223.6%
Zdhhc1	9.703036	0.32	3.3%
Zdhhc11	0.01042394	0.01	140.6%
Zdhhc12	1.322487	1.31	99.3%
Zdhhc13	9.537888	0.60	6.3%
Zdhhc14	3.811276	0.61	16.1%
Zdhhc15	3.881776	0.31	7.9%
Zdhhc16	22.87612	2.39	10.5%
Zdhhc17	23.64976	1.33	5.6%
Zdhhc18	11.96112	0.94	7.8%
Zdhhc19	0	0.00	
Zdhhc2	26.26966	1.29	4.9%
Zdhhc20	18.14278	1.76	9.7%

Zdhhc21	12.39286	1.03	8.3%
Zdhhc22	10.82604	0.57	5.3%
Zdhhc23	0.610252	0.07	11.4%
Zdhhc24	13.09766	0.52	4.0%
Zdhhc25	0	0.00	
Zdhhc3	12.98562	1.22	9.4%
Zdhhc4	8.889478	1.31	14.7%
Zdhhc5	20.45182	1.00	4.9%
Zdhhc6	8.492094	0.38	4.5%
Zdhhc7	8.68202	0.42	4.9%
Zdhhc8	13.45626	0.67	5.0%
Zdhhc9	15.3584	1.33	8.7%
Zeb1	10.92276	0.62	5.7%
Zeb2	3.773464	0.14	3.8%
Zer1	33.00592	1.66	5.0%
Zfa	0.22187	0.08	35.6%
Zfand1	2.161114	0.37	17.3%
Zfand2a	22.0673	0.80	3.6%
Zfand2b	6.390432	0.10	1.6%
Zfand3	16.8241	1.59	9.4%
Zfand5	54.73212	2.01	3.7%
Zfand6	19.72512	1.30	6.6%
Zfat1	0.9312454	0.14	14.8%
Zfhx1a	0.02919434	0.01	20.1%
Zfhx1b	0.11506722	0.04	38.7%
Zfhx2	3.361834	0.33	9.8%
Zfhx3	2.875932	0.20	6.9%
Zfhx4	4.462144	0.36	8.0%
Zfml	16.1315	0.99	6.1%
Zfp1	4.897768	0.32	6.6%
Zfp101	2.63385	0.19	7.4%
Zfp105	2.408138	0.15	6.3%
Zfp106	12.70088	1.13	8.9%
Zfp108	1.213588	0.04	3.4%
Zfp109	0.7428972	0.09	11.7%
Zfp11	1.541242	0.10	6.2%
Zfp110	2.482122	0.22	9.0%
Zfp111	0.9171668	0.09	9.9%
Zfp112	1.881958	0.28	15.0%
Zfp113	1.297558	0.08	6.1%
Zfp114	0.209793	0.04	19.0%
Zfp119	1.66879	0.22	13.2%
Zfp12	5.247338	0.16	3.1%
Zfp120	3.055802	0.31	10.0%
Zfp128	1.67291	0.15	9.1%
Zfp13	5.74709	0.12	2.2%
Zfp131	0	0.00	

Zfp14	4.121946	0.32	7.9%
Zfp142	4.145642	1.22	29.4%
Zfp143	2.255694	0.20	8.7%
Zfp146	12.7407	0.44	3.5%
Zfp148	8.28886	0.23	2.8%
Zfp157	6.259132	0.26	4.1%
Zfp160	5.542002	0.30	5.4%
Zfp161	6.504388	0.29	4.4%
Zfp167	0.642737	0.06	9.9%
Zfp169	2.640102	0.28	10.6%
Zfp174	1.20213	0.08	6.5%
Zfp179	7.065794	0.62	8.8%
Zfp180	6.718832	0.28	4.2%
Zfp182	1.60466	0.14	8.9%
Zfp184	0.8120702	0.09	10.6%
Zfp185	0.1472194	0.03	21.1%
Zfp187	12.81236	1.91	14.9%
Zfp189	1.842088	0.13	7.3%
Zfp191	7.978028	0.28	3.4%
Zfp192	3.1149	0.17	5.4%
Zfp2	7.478412	0.28	3.7%
Zfp202	1.340264	0.07	5.3%
Zfp206	0.0041564	0.01	138.9%
Zfp207	36.20264	1.10	3.0%
Zfp212	3.26238	0.17	5.1%
Zfp213	2.206374	0.12	5.4%
Zfp217	0.6872392	0.12	17.2%
Zfp219	6.862382	0.91	13.3%
Zfp235	3.46568	0.30	8.8%
Zfp236	0.2070906	0.09	45.0%
Zfp238	9.778284	0.52	5.3%
Zfp239	7.19276	0.26	3.7%
Zfp248	5.602058	0.34	6.1%
Zfp251	11.1607	0.40	3.6%
Zfp259	7.255084	0.43	5.9%
Zfp26	4.639444	0.59	12.7%
Zfp260	12.07192	0.52	4.3%
Zfp263	5.374934	0.18	3.3%
Zfp27	4.307582	0.22	5.2%
Zfp273	2.063236	0.26	12.7%
Zfp275	6.722576	0.28	4.1%
Zfp276	1.659594	0.48	29.1%
Zfp277	4.226026	0.51	12.0%
Zfp28	1.769318	0.20	11.2%
Zfp280b	5.780328	0.81	14.1%
Zfp280c	3.43058	0.33	9.6%
Zfp281	4.616608	0.29	6.3%

Zfp282	6.40153	0.62	9.6%
Zfp286	2.781318	0.18	6.6%
Zfp287	2.741526	0.17	6.4%
Zfp289	14.321426	3.06	21.4%
Zfp292	6.34921	0.34	5.3%
Zfp294	10.89696	0.29	2.7%
Zfp295	3.157356	0.23	7.3%
Zfp296	0.03586728	0.04	100.4%
Zfp3	3.792406	0.22	5.8%
Zfp30	4.368904	0.36	8.1%
Zfp300	0.76082	0.10	13.6%
Zfp31	0.677464	0.08	11.9%
Zfp313	19.8838	0.75	3.8%
Zfp316	5.410782	0.29	5.4%
Zfp318	21.98542	1.11	5.1%
Zfp319	5.342454	0.27	5.1%
Zfp322a	8.691892	0.91	10.4%
Zfp324	3.616236	0.25	6.9%
Zfp326	7.582946	0.69	9.1%
Zfp329	5.153506	0.50	9.7%
Zfp330	1.678694	0.34	20.5%
Zfp334	6.49444	0.33	5.1%
Zfp335	4.62524	0.34	7.4%
Zfp341	1.751052	0.14	8.0%
Zfp346	12.7634	1.13	8.9%
Zfp35	5.682134	0.24	4.2%
Zfp352	0.01422556	0.01	94.9%
Zfp353	0	0.00	
Zfp354a	1.66397	0.07	3.9%
Zfp354b	0.9655984	0.05	5.5%
Zfp354c	14.4114	0.55	3.8%
Zfp358	10.85564	0.51	4.7%
Zfp36	0.3288542	0.10	31.2%
Zfp364	24.50072	1.61	6.6%
Zfp365	9.379598	0.19	2.0%
Zfp366	0.00730476	0.01	147.5%
Zfp367	4.254292	0.64	15.0%
Zfp369	3.373178	0.43	12.7%
Zfp36l1	7.540344	1.17	15.5%
Zfp36l2	2.113066	0.49	23.1%
Zfp36l3	0.00198053	0.00	223.6%
Zfp37	5.442722	0.36	6.7%
Zfp371	0.00252328	0.01	223.6%
Zfp382	3.377284	0.38	11.2%
Zfp384	9.235442	0.82	8.9%
Zfp385	26.28148	1.33	5.0%
Zfp386	5.79119	0.36	6.3%

Zfp39	1.69041	0.13	7.8%
Zfp395	4.054884	0.79	19.5%
Zfp397	6.751624	0.29	4.3%
Zfp398	3.819456	0.37	9.8%
Zfp40	1.988666	0.08	4.1%
Zfp407	1.92819	0.34	17.9%
Zfp408	3.233878	0.33	10.3%
Zfp41	9.89069	0.90	9.1%
Zfp410	7.108788	0.49	6.9%
Zfp414	9.46115	4.39	46.4%
Zfp418	1.122616	0.15	13.1%
Zfp42	0.01029252	0.02	147.9%
Zfp422	9.65042	0.39	4.0%
Zfp422-rs1	4.731816	0.18	3.8%
Zfp423	4.968048	0.48	9.6%
Zfp426	11.1287	0.81	7.3%
Zfp428	17.58168	2.22	12.6%
Zfp438	1.439818	0.14	9.8%
Zfp444	11.99122	0.57	4.8%
Zfp445	43.38588	1.88	4.3%
Zfp446	2.555372	0.32	12.7%
Zfp449	1.653858	0.04	2.5%
Zfp451	10.049516	0.76	7.6%
Zfp454	1.1343946	0.24	20.9%
Zfp455	0.79702	0.15	18.3%
Zfp456	0.2409432	0.07	28.9%
Zfp457	0.7211788	0.07	9.8%
Zfp458	1.697214	0.16	9.3%
Zfp459	0.3121098	0.08	24.7%
Zfp46	8.748536	0.56	6.4%
Zfp462	5.799296	0.21	3.6%
Zfp467	4.40854	0.60	13.6%
Zfp469	12.80972	0.93	7.2%
Zfp472	1.454514	0.14	9.8%
Zfp473	0.2905282	0.04	12.8%
Zfp474	0	0.00	
Zfp488	0.06056662	0.02	30.9%
Zfp496	1.0874962	0.11	9.8%
Zfp498	4.487294	0.41	9.1%
Zfp503	5.867904	0.41	7.0%
zfp507	5.31748	0.57	10.7%
Zfp508	5.891204	0.20	3.5%
Zfp509	1.566038	0.22	14.1%
Zfp51	1.238412	0.14	11.4%
Zfp511	5.136274	0.55	10.7%
Zfp512	19.45782	0.52	2.7%
Zfp513	4.818938	0.24	5.0%

Zfp516	2.075326	0.25	11.8%
Zfp518	3.259146	0.26	7.9%
Zfp52	0.8265472	0.21	24.9%
Zfp521	9.325214	0.52	5.6%
Zfp523	13.60794	0.97	7.1%
Zfp524	1.2982	0.12	9.3%
Zfp526	1.331626	0.14	10.7%
Zfp53	1.510832	0.28	18.3%
Zfp532	10.82964	0.47	4.3%
Zfp533	4.050588	0.21	5.1%
Zfp536	2.056772	0.15	7.5%
Zfp54	0.4078234	0.06	14.9%
Zfp541	0.1953526	0.02	10.4%
Zfp551	1.967046	0.18	9.4%
Zfp553	5.184974	0.16	3.1%
Zfp560	0.825885	0.05	5.9%
Zfp563	3.801674	0.22	5.7%
Zfp566	1.278408	0.11	8.9%
Zfp568	0.620601	0.03	5.1%
Zfp57	13.76944	1.40	10.2%
Zfp574	7.012842	0.41	5.9%
Zfp575	2.565502	0.25	9.8%
Zfp579	6.892898	0.52	7.6%
Zfp583	2.32743	0.32	13.9%
Zfp59	1.645212	0.12	7.1%
Zfp592	5.498548	0.30	5.5%
Zfp593	1.960194	0.31	15.6%
Zfp595	0.3412838	0.09	26.8%
Zfp597	6.8362	0.24	3.5%
Zfp598	10.174272	0.41	4.0%
Zfp60	10.75114	0.22	2.0%
Zfp606	4.011344	0.09	2.2%
Zfp607	0.431815	0.07	17.3%
Zfp608	4.861888	0.50	10.2%
Zfp609	5.890834	0.31	5.3%
Zfp61	5.045588	0.29	5.7%
Zfp612	7.385042	0.52	7.1%
Zfp617	3.992188	0.29	7.2%
Zfp619	0.6571188	0.06	8.5%
Zfp62	8.490994	0.50	5.9%
Zfp622	12.71412	0.47	3.7%
Zfp623	5.674464	0.26	4.5%
Zfp628	2.08148	0.12	5.8%
Zfp629	4.800224	0.27	5.7%
Zfp637	11.58682	0.73	6.3%
Zfp639	9.693674	0.30	3.1%
Zfp64	3.998518	0.45	11.1%

Zfp641	2.614922	0.12	4.8%
Zfp644	16.21658	0.52	3.2%
Zfp646	2.490688	0.18	7.2%
Zfp647	2.945368	0.24	8.0%
Zfp650	45.59746	2.49	5.5%
Zfp652	2.87096	0.17	6.1%
Zfp653	2.810756	0.17	5.9%
Zfp654	4.56947	0.39	8.5%
Zfp655	11.58892	0.61	5.2%
Zfp661	1.548574	0.17	10.8%
Zfp664	16.60934	0.59	3.5%
Zfp667	5.48057	0.39	7.0%
Zfp668	3.567136	0.21	5.8%
Zfp672	7.06937	0.38	5.3%
Zfp677	0.3315438	0.05	15.9%
Zfp68	10.201896	0.33	3.3%
Zfp687	6.709142	0.60	9.0%
Zfp688	3.683378	0.35	9.4%
Zfp689	1.954972	0.16	8.0%
Zfp69	0.14039418	0.05	33.9%
Zfp690	1.039868	0.15	14.6%
Zfp691	1.121658	0.14	12.7%
Zfp692	5.19647	0.71	13.6%
Zfp694	0.848442	0.17	20.0%
Zfp697	2.0147054	1.26	62.3%
Zfp7	2.99817	0.25	8.3%
Zfp704	9.51604	1.09	11.4%
Zfp706	61.09968	2.77	4.5%
Zfp707	4.868186	0.30	6.2%
Zfp708	1.521192	0.18	11.9%
Zfp709	0.506598	0.06	12.0%
Zfp710	1.359696	0.10	7.1%
Zfp711	7.56664	0.53	7.0%
Zfp715	7.066276	0.18	2.5%
Zfp719	4.733438	0.08	1.6%
Zfp71-rs1	3.118852	0.22	7.1%
Zfp72	1.936206	0.26	13.6%
Zfp74	6.860994	0.56	8.2%
Zfp740	7.724822	1.37	17.7%
Zfp748	4.35811	0.24	5.5%
Zfp75	6.00795	0.40	6.6%
Zfp750	0.009876726	0.01	141.3%
Zfp758	1.1844192	0.27	22.6%
Zfp759	2.84218	0.24	8.6%
Zfp760	1.88456	0.10	5.1%
Zfp764	0.4020886	0.11	27.2%
Zfp768	4.104292	0.38	9.3%

Zfp770	8.261104	0.31	3.7%
Zfp771	9.509594	0.44	4.7%
Zfp775	1.763268	0.10	5.7%
Zfp777	8.809316	0.28	3.2%
Zfp78	0.621978	0.10	16.0%
Zfp780b	3.915758	0.14	3.6%
Zfp784	1.979068	0.15	7.7%
Zfp786	1.714626	0.25	14.7%
Zfp787	6.867686	1.09	15.9%
Zfp790	3.97617	0.35	8.7%
Zfp791	1.66323	0.15	9.2%
Zfp800	2.611714	0.12	4.6%
Zfp804a	6.2578	0.34	5.4%
Zfp809	2.25896	0.16	7.3%
Zfp81	4.297206	0.44	10.3%
Zfp810	4.138846	0.23	5.6%
Zfp811	5.297918	0.26	4.9%
Zfp817	4.409874	1.07	24.3%
Zfp819	0.07396744	0.03	37.4%
Zfp82	1.905752	0.20	10.3%
Zfp820	0.6255358	0.10	16.2%
Zfp84	2.982026	0.26	8.8%
Zfp85-rs1	1.806374	0.03	1.7%
Zfp87	4.646042	0.85	18.3%
Zfp9	7.04635	0.33	4.6%
Zfp90	5.688866	0.36	6.3%
Zfp91	28.6459	0.69	2.4%
Zfp91-cntf	0.03890324	0.02	60.3%
Zfp92	0.1922544	0.05	27.4%
Zfp93	1.0629868	0.09	8.4%
Zfp94	2.686428	0.28	10.4%
Zfp97	0.5601124	0.11	19.1%
Zfpl1	14.12418	0.87	6.2%
Zfpm1	1.674178	0.15	8.9%
Zfpm2	2.000574	0.17	8.5%
Zfr	68.23398	2.66	3.9%
Zfx	3.910486	0.20	5.0%
Zfy1	0	0.00	
Zfy2	0	0.00	
Zfyve1	10.36144	0.65	6.3%
Zfyve16	3.660004	0.30	8.2%
Zfyve19	5.91229	0.22	3.7%
Zfyve20	10.93076	0.42	3.8%
Zfyve21	2.349456	0.29	12.5%
Zfyve26	1.165118	0.09	7.6%
Zfyve27	25.95066	1.25	4.8%
Zfyve28	3.880376	0.38	9.7%

Zfyve9	21.2774	0.73	3.5%
Zgpat	8.97328	1.18	13.1%
Zh2c2	1.787034	0.27	14.9%
Zhx1	17.27314	0.52	3.0%
Zhx2	1.598222	0.14	8.9%
Zhx3	5.9729	0.60	10.0%
Zic1	19.01164	1.13	5.9%
Zic2	9.957194	0.79	7.9%
Zic3	2.63624	0.16	6.1%
Zic4	2.429058	0.26	10.8%
Zic5	0.4290284	0.09	20.5%
Zik1	3.788022	0.28	7.5%
Zim1	5.035404	0.64	12.8%
Zkscan1	10.004546	0.53	5.3%
Zkscan14	3.049382	0.19	6.2%
Zkscan17	3.72622	0.32	8.5%
Zkscan2	4.78966	0.88	18.3%
Zkscan3	6.187208	0.29	4.7%
Zkscan5	4.362826	0.19	4.4%
Zkscan6	2.35831	0.22	9.5%
Zmat1	3.090712	0.39	12.5%
Zmat2	30.25222	1.45	4.8%
Zmat3	23.63614	1.30	5.5%
Zmat4	15.76816	0.68	4.3%
Zmat5	6.861024	0.54	7.9%
Zmiz1	33.8755	2.42	7.1%
Zmiz2	42.29112	1.77	4.2%
Zmpste24	10.82434	0.53	4.9%
Zmym1	4.933346	0.68	13.8%
Zmym2	21.40034	1.10	5.2%
Zmym3	29.93224	1.57	5.3%
Zmym4	14.85182	0.68	4.6%
Zmym5	3.890438	0.32	8.2%
Zmym6	11.31141	1.36	12.1%
Zmynd10	0.4386646	0.09	20.0%
Zmynd11	29.70556	1.00	3.4%
Zmynd12	0.01723966	0.03	148.8%
Zmynd15	0.11582966	0.05	39.7%
Zmynd17	0.2149692	0.04	18.8%
Zmynd19	8.731896	0.78	8.9%
Znf202	0.3279974	0.08	25.0%
Znf230	0.3272478	0.16	49.1%
Znf326	0.7901234	0.20	25.4%
Znf509	0.6647238	0.28	42.3%
Znf580	12.8221	1.40	10.9%
Znf638	0.1468696	0.04	30.5%
Znf710	0.21951564	0.10	47.1%

Znf740	10.950618	0.72	6.6%
Znf746	4.609238	0.20	4.3%
Znfx1	3.84209	0.10	2.7%
Znhit1	15.90398	2.04	12.8%
Znhit2	10.25616	0.16	1.5%
Znhit3	12.32126	1.16	9.4%
Znhit4	2.720978	0.43	15.7%
Znrd1	6.85102	0.33	4.8%
Znrf1	41.75052	1.27	3.1%
Znrf2	8.442826	0.27	3.2%
Znrf3	1.426574	0.18	12.9%
Znrf4	0	0.00	
Zp1	0.0188818	0.01	79.1%
Zp2	0	0.00	
Zp3	0.0104756	0.01	137.0%
Zp3r	0.0033345	0.01	223.6%
Zpbp	1.793766	0.11	6.4%
Zpbp2	0.01991136	0.03	140.4%
Zpld1	0.00317678	0.01	223.6%
Zranb1	14.38014	0.68	4.7%
Zranb2	57.40644	2.39	4.2%
Zranb3	1.569524	0.12	7.6%
Zrsr1	55.2912	0.84	1.5%
Zrsr2	15.1627	1.31	8.6%
Zscan10	0.009655736	0.01	139.6%
Zscan12	1.0673146	0.13	12.6%
Zscan2	2.032908	0.13	6.6%
Zscan20	0.4261876	0.11	25.3%
Zscan21	6.6236	0.64	9.7%
Zscan22	2.184076	0.27	12.6%
Zscan4d	0	0.00	
Zswim1	8.102348	0.33	4.1%
Zswim2	0	0.00	
Zswim3	3.287936	0.12	3.6%
Zswim4	2.279206	0.23	10.1%
Zswim5	2.88471	0.17	5.9%
Zswim6	9.23641	0.66	7.1%
Zubr1	5.441846	1.21	22.2%
Zw10	5.560698	0.24	4.4%
Zwilch	0.9292998	0.14	15.1%
Zwint	542.0876	14.71	2.7%
Zxdb	2.122398	0.14	6.5%
Zxdc	5.44624	0.18	3.3%
Zyg11a	0.00772498	0.01	138.7%
Zyg11b	83.13448	6.20	7.5%
Zyx	19.48082	1.17	6.0%
Zzef1	7.287726	0.62	8.4%

Zzz3	5.642644	0.59	10.4%
