# Supplementary material for: High yield derivation of enriched glutamatergic neurons from suspension-cultured mouse ESCs for neurotoxicology research
Source: BMC Neurosci. 2012 Oct 24;13:127. doi: 10.1186/1471-2202-13-127 (PMC3573964; doi:10.1186/1471-2202-13-127)
Supplement: Additional file 2: Table S2 — Summary of neurotypic gene expression in DIV 14 ESNs. Representative transcripts involved in neuron function, neurogenesis or neurotoxin function are included. Highlighted sequences represent abundant transcripts, defined as an average reads per kilobase of exon per million mapped sequences (FPKM) exceeding 30. [file 1471-2202-13-127-S2.pdf]

Functional category	Function	Symbol	Average RKPM	Common Gene Name
Neuronal Subtype Markers	Glutamatergic	Slc17a7	0.133	vGluT1
		Slc17a6	100.80	vGluT2
		Slc17a8	0.12	vGluT3
		Slc17a5	4.32	Sialin
	GABAergic	Gad1	10.03	Glutamate decarboxylase 1
		Gad2	13.88	Glutamate decarboxylase 2
		Slc32a1	11.57	vGAT
	Dopaminergic	Th	0.007	Tyrosine hydroxylase
	Cholinergic and motor neuron	vAChT	0.11	Vesicular ACh Transferase
		ChAT	0.07	Choline acetyltransferase
		Mnx1	0.302	Motor neuron and pancreas homeobox 1
		Isl1	0.354	LIM homeobox 1
	Serotoninergic	Slc6a4	0.01	Serotonin transporter
		Tph1	ND	tryptophan hydroxylase 1
		Tph2	0.03	tryptophan hydroxylase 2
	Glutamate	Gria1	30.35608	Glutamate receptor, ionotropic, AMPA 1
		Gria2	60.77608	Glutamate receptor, ionotropic, AMPA 2
		Gria3	19.70934	Glutamate receptor, ionotropic, AMPA 3
		Gria4	67.54714	Glutamate receptor, ionotropic, AMPA 4
		Grik1	5.235484	Glutamate receptor, ionotropic, Kainate 1
		Grik2	9.172482	Glutamate receptor, ionotropic, Kainate 2
		Grik3	9.686086	Glutamate receptor, ionotropic, Kainate 3
		Grik4	6.51067	Glutamate receptor, ionotropic, Kainate 4
		Grik5	33.36404	Glutamate receptor, ionotropic, Kainate 5
		Grin1	122.381	Glutamate receptor, ionotropic, NMDA 1
		Grin2a	2.127506	Glutamate receptor, ionotropic, NMDA 2A
		Grin2b	2.3164	Glutamate receptor, ionotropic, NMDA 2B
		Grin2c	0.04316324	Glutamate receptor, ionotropic, NMDA 2C
		Grin2d	7.605112	Glutamate receptor, ionotropic, NMDA 2D
		Grin3a	7.380222	Glutamate receptor, ionotropic, NMDA 3A
		Grin3b	0.6538906	Glutamate receptor, ionotropic, NMDA 3B

		Grm1	2.443274	Glutamate receptor, metabotropic 1
		Grm2	0.3390048	Glutamate receptor, metabotropic 2
		Grm3	4.035222	Glutamate receptor, metabotropic 3
		Grm4	9.94198	Glutamate receptor, metabotropic 4
		Grm5	16.30998	Glutamate receptor, metabotropic 5
		Grm6	ND	Glutamate receptor, metabotropic 6
		Grm7	18.03178	Glutamate receptor, metabotropic 7
		Grm8	3.685954	Glutamate receptor, metabotropic 8
		Grina	267.7604	Glutamate receptor, ionotropic, NMDA-associated protein 1
	GABA	Gabra1	9.01074	Gamma-aminobutyric acid (GABA) A receptor, α1
		Gabra2	28.22418	Gamma-aminobutyric acid (GABA) A receptor, α2
		Gabra3	26.0508	Gamma-aminobutyric acid (GABA) A receptor, α3
		Gabra4	5.83007	Gamma-aminobutyric acid (GABA) A receptor, α4
		Gabra5	28.9894	Gamma-aminobutyric acid (GABA) A receptor, α5
		Gabra6	ND	Gamma-aminobutyric acid (GABA) A receptor, α6
		Gabrb1	3.906874	Gamma-aminobutyric acid (GABA) A receptor, β1
		Gabrb2	13.46166	Gamma-aminobutyric acid (GABA) A receptor, β2
		Gabrb3	56.24872	Gamma-aminobutyric acid (GABA) A receptor, β3
	Glycine	Glra1	12.848712	Glycine receptor, α1
		Glra2	8.761032	Glycine receptor, α2
		Glra3	5.173716	Glycine receptor, α3
		Glra4	0.00837082	Glycine receptor, α4
		Glrb	108.062	Glycine receptor, β
	Dopamine	Drd1a	0.07373384	Dopamine receptor D1
		Drd2	7.23483	Dopamine receptor D2
		Drd3	0.00864052	Dopamine receptor D3
		Drd4	0.07192894	Dopamine receptor D4
		Drd5	0.496315	Dopamine receptor D5
		Chrna1	0.0070755	Cholinergic receptor, nicotinic, α1
		Chrna2	0.02744146	Cholinergic receptor, nicotinic, α2
		Chrna3	0.275886	Cholinergic receptor, nicotinic, α3
		Chrna4	20.67094	Cholinergic receptor, nicotinic, α4
		Chrna5	0.3836258	Cholinergic receptor, nicotinic, α5
		Chrna6	0.01037758	Cholinergic receptor, nicotinic, α6

Receptors	Acetylcholine	Chrna7	8.656464	Cholinergic receptor, nicotinic, α7
		Chrna9	ND	Cholinergic receptor, nicotinic, α9
		Chrna10	0.000107139	Cholinergic receptor, nicotinic, α10
		Chrnb1	0.4118318	Cholinergic receptor, nicotinic, β1
		Chrnb2	20.07258	Cholinergic receptor, nicotinic, β2
		Chrnb3	0.005663962	Cholinergic receptor, nicotinic, β3
		Chrnb4	0.161446	Cholinergic receptor, nicotinic, β4
		Chrnd	ND	Cholinergic receptor, nicotinic, δ
		Chrne	ND	Cholinergic receptor, nicotinic, ε
		Chrng	ND	Cholinergic receptor, nicotinic, γ
		Chrm1	0.0064537	Cholinergic receptor, muscarinic 1
		Chrm2	18.50262	Cholinergic receptor, muscarinic 2
		Chrm3	8.73957	Cholinergic receptor, muscarinic 3
		Chrm4	2.234862	Cholinergic receptor, muscarinic 4
		Chrm5	0.06815074	Cholinergic receptor, muscarinic 5
	Serotonin	Htr1a	4.405996	5-HT_{1A} receptor
		Htr1b	3.4554418	5-HT_{1B} receptor
		Htr1d	0.2505624	5-HT_{1D} receptor
		Htr1f	0.03416864	5-HT_{1F} receptor
		Htr2a	6.485528	5-HT_{2A} receptor
		Htr2b	0.01619778	5-HT_{2B} receptor
		Htr2c	46.51112	5-HT_{2C} receptor
		Htr3a	0.07764886	5-HT_{3A} receptor
		Htr3b	0.02939726	5-HT_{3B} receptor
		Htr4	1.772444	5-HT_{4} receptor
		Htr5a	0.7344406	5-HT_{5A} receptor
		Htr5b	0.05585322	5-HT_{5B} receptor
		Htr6	0.7168226	5-HT_{6} receptor
		Htr7	13.4136	5-HT_{7} receptor
		Sstr1	14.12698	Somatostatin receptor 1
		Sstr1	14.12698	Somatostatin receptor 1

	Miscellaneous receptors	Sstr2	2.919584	Somatostatin receptor 2
		Sstr3	0.373493	Somatostatin receptor 3
		Sstr4	0.6302732	Somatostatin receptor 4
		Sstr5	0.00477514	Somatostatin receptor 5
		Brs3	0.00584498	Bombesin-like receptor 3
		Galr1	0.5353272	Galanin receptor 1
		Galr2	0.4362224	Galanin receptor 2
		Galr3	2.3909507	Galanin receptor 3
		Nmbr	1.26173	Neuromedin B receptor
		Nmur1	0.12054478	Neuromedin U receptor 1
		Nmur2	0.2864554	Neuromedin U receptor 2
		Adra1a	2.282118	α-1A adrenergic receptor
		Adra1b	10.276112	α-1B adrenergic receptor
		Adra1d	0.8868502	α-1D adrenergic receptor
		Adra2a	17.66096	α-2A adrenergic receptor
		Adra2b	0.007961938	α-2B adrenergic receptor
		Adra2c	0.7679106	α-2C adrenergic receptor
		Adrb1	1.9138	β-1 adrenergic receptor
		Adrb2	0.1401438	β-2 adrenergic receptor
		Adrb3	0.4855288	β-3 adrenergic receptor
		Tacr1	8.85355	Tachykinin receptor 1
		Tacr2	ND	Tachykinin receptor 2
		Tacr3	3.022146	Tachykinin receptor 3
		PTPRS	68.4817	Receptor-type tyrosine-protein phosphatase S
	Voltage-gated potassium channels	Kcna1	12.545514	Shaker-related, member 1
		Kcna2	11.830216	Shaker-related, member 2
		Kcna6	17.83032	Shaker-related, member 6
		Kcnab2	45.5189	Shaker-related, β member 2
		Kcnc1	27.47524	Shaw-related, member 1
		Kcnc3	16.22718	Shaw-related, member 3
		Kcnc4	14.66868	Shaw-related, member 4
		Kcnd2	24.68834	Shal-related, member 2
		Kcnh2	20.17306	hERG-related, member 1
		Kcnma1	10.95116	Large conductance Ca^{2+}-activated channel, subfamily M, α1

Voltage-gated Ion Channels	Voltage-dependent calcium channels	Cacna1a	18.84142	L-type α_1 subunit
		Cacna1b	10.296098	N-type α_1 subunit
		Cacna2d1	16.37378	$\alpha_2\delta$ Subunit
		Cacna2d2	18.70052	$\alpha_2\delta$ Subunit
		Cacnb1	15.89976	L-type β1 subunit
		Cacnb2	2.56098	L-type β2 subunit
		Cacnb3	15.69302	L-type β3 subunit
		Cacnb4	15.19204	L-type β4 subunit
		Cacng2	25.98072	γ2 subunit
		Cacng3	18.64872	γ3 subunit
		Cacng4	16.44202	γ4 subunit
		Cacng7	55.51874	γ7 subunit
	Voltage-gated sodium channels	Scn1a	29.7139	Type I, α subunit
		Scn1b	86.58392	Type I, β subunit
		Scn2a1	24.8485	Type II, α subunit
		Scn2b	60.75688	Type II, β subunit
		Scn3a	10.857716	Type III, α subunit
		Scn3b	36.61484	Type III, β subunit
		Scn8a	33.37398	Type VIII, α subunit
	Voltage-gated chloride channels	Clcn1	0.1915332	voltage-dependent chloride channel 1
		Clcn2	7.257858	voltage-dependent chloride channel 2
		Clcn3	52.63078	voltage-dependent chloride channel 3
		Clcn4-2	48.52664	voltage-dependent chloride channel 4-2
		Clcn5	17.83006	voltage-dependent chloride channel 5
		Clcn6	31.50824	voltage-dependent chloride channel 6
		Clcn7	14.04322	voltage-dependent chloride channel 7
		Clcnka	ND	voltage-dependent chloride channel Ka
		Clcnkb	0.01282394	voltage-dependent chloride channel Kb
		Syp	413.217	Synaptophysin
		Sv2a	145.4704	Synaptic vesicle glycoprotein 2A
		Sv2b	14.71162	Synaptic vesicle glycoprotein 2B
		Sv2c	24.58828	Synaptic vesicle glycoprotein 2C
		Synpr	16.04598	Synaptoporin

Synapse	Presynaptic vesicle-associated	Cplx1	327.2848	Complexin 1
		Stx1b2	137.9928	Syntaxin 1B
		Stxbp1	368.7822	Syntaxin binding protein
		Syt1	122.8554	Synaptotagmin 1
		Syngr1	150.5436	Synaptogyrin 1
		Syngr3	128.5664	Synaptogyrin 3
		Stxbp1	368.7822	Munc18
		Vamp2	465.013	Vesicle-associated membrane protein 2
		Snap25	706.9992	Synaptosomal-associated protein 25
		Syn1	140.7648	Synapsin 1
		Syn2	109.52746	Synapsin 2
	Presynaptic active zone	Pclo	9.378232	Piccolo
		Bsn	7.846538	Bassoon
		Cask	52.78174	CASK
		Caskin1	41.54494	CASKIN
		Erc1	16.55554	ERC 1
		Erc2	16.87308	ERC 2
		Ctbp2	9.589376	Ribeye
	Presynaptic clathrin-mediated endocytosis	Clta	110.5168	Clathrin A
		Cltb	82.91766	Clathrin B
		Cltc	159.5734	Clathrin C
		Synj1	48.23858	Synaptojanin 1
		Sh3gl2	82.0416	Endophilin A1
		Ap2a1	89.73118	Adaptor-related protein complex 2, α1
		Ap2a2	54.65592	Adaptor-related protein complex 2, α2
		Ap2b1	80.57466	Adaptor-related protein complex 2, µ1
		Ap2m1	16.8416	Adaptor-related protein complex 2, σ1
		Scamp1	91.38928	Secretory carrier membrane protein 1
		Scamp2	4.392658	Secretory carrier membrane protein 2
		Scamp3	37.45006	Secretory carrier membrane protein 3
		Scamp4	11.8911	Secretory carrier membrane protein 4
		Scamp5	94.34392	Secretory carrier membrane protein 5
		Ap2s1	63.71916	Dynamin
		Dlg2	34.96596	PSD-93

	Postsynaptic	Dlg4	80.0291	PSD-95
		Shank2	5.891272	SH3 and multiple ankyrin repeat domains 2
		Shank3	17.05286	SH3 and multiple ankyrin repeat domains 3
		Gphn	42.36262	Gephyrin
Cytoskeletal	Structural	Mapt	124.2266	Tau
		Tubb3	404.603	β-III tubulin
		Stmn2	398.1574	Stathmin-like 2
		Stmn3	453.6074	Stathmin-like 3
		Rtn1	615.747	Reticulon 1
		Rtn3	864.7276	Reticulon 3
		Nefh	79.42608	Neurofilament H
		Nefl	765.079	Neurofilament L
		Nefm	10.044324	Neurofilament M
		Ina	129.8978	α-internexin
		Mtap2	176.8036	MAP2
Metabolic	Sodium/potassium pumps	Atp1a3	974.394	Sodium/Potassium pump (α subunit)
		Atp1b1	681.843	Sodium/Potassium pump (β subunit)
	Calcium response pathways	Ncdn	171.9668	Neurochondrin
		Camk2b	107.09824	Calcium/calmodulin-dependent protein kinase 2B
		Camk2d	81.81252	Calcium/calmodulin-dependent protein kinase 2D
		Camk2g	73.6673	Calcium/calmodulin-dependent protein kinase 2G
		Camk2n1	54.82786	Calcium/calmodulin-dependent protein kinase II inhibitor 1
		Camk2n2	117.5138	Calcium/calmodulin-dependent protein kinase II inhibitor 2
	mRNA splicing	R3hdm2	23.95266	NeuN
lolecules	Synaptogenesis	Ncam1	116.4502	Neural cell adhesion molecule 1
		Lphn1	65.3427	Latrophilin 1
		Lphn2	24.9546	Latrophilin 2
		Lphn3	37.94026	Latrophilin 3
		Ncam2	33.11438	Neural cell adhesion molecule 2
		Cdh2	24.54784	N-cadherin
		Nlgn1	13.44976	Neuroligin 1
		Nlgn2	69.38652	Neuroligin 2
		Nlgn3	18.3061	Neuroligin 3
		Nrxn1	44.7362	Neurexin 1

Adhesion M		Nrxn2	111.0678	Neurexin 2
		Nrxn3	21.71548	Neurexin 3
	Neurite guidance	Cntn1	138.7748	Contactin 1
		Cntn2	9.052066	Contactin 2
		Cntn4	12.34568	Contactin 4
		Cntnap1	73.47142	Contactin-associated protein 1
		Cntnap2	25.50548	Contactin-associated protein 2
		Gprin1	20.1613	G protein regulated inducer of neurite outgrowth 1
		Gap43	299.2398	Growth associated protein 43
		Reln	41.66184	Reelin
		Pou3f3	8.4521	POU class 3 homeobox 31
