# Supplementary figures and images for: High yield derivation of enriched glutamatergic neurons from suspension-cultured mouse ESCs for neurotoxicology research
Source: BMC Neurosci. 2012 Oct 24;13:127. doi: 10.1186/1471-2202-13-127 (PMC3573964; doi:10.1186/1471-2202-13-127)

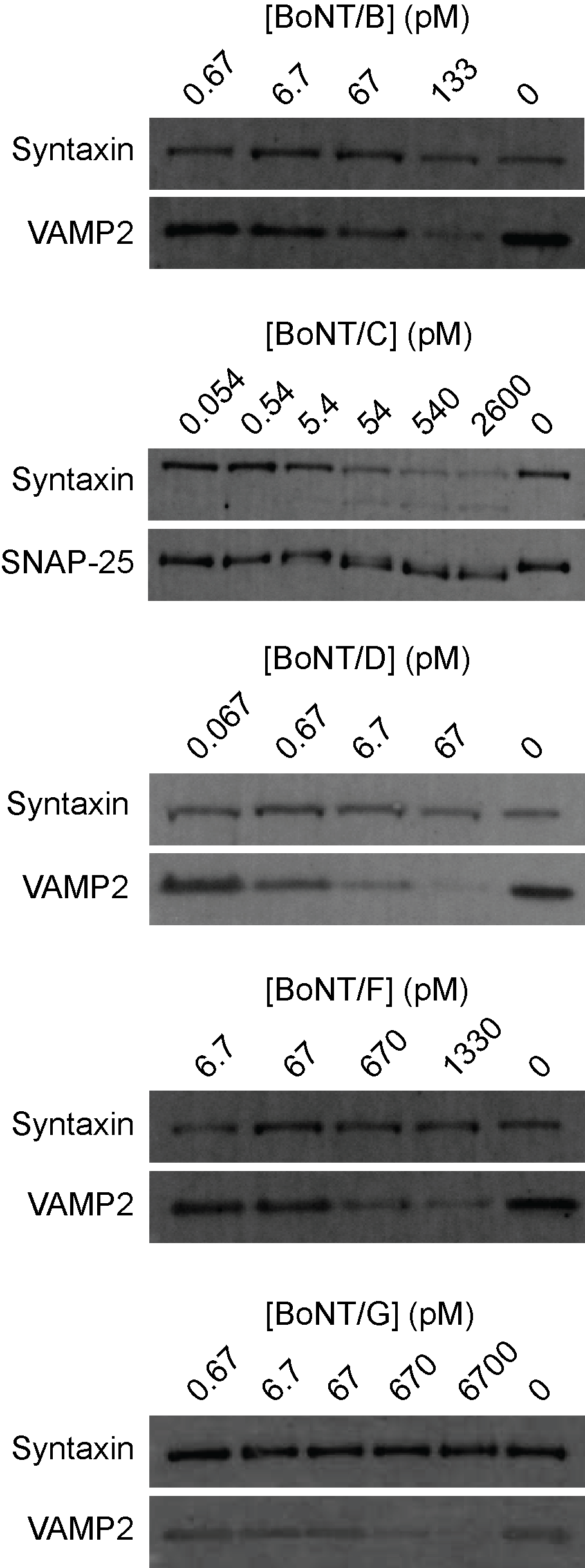

Supplement: Additional file 3: Figure S1 — Representative immunoblots demonstrating dose-dependent proteolysis of target specific SNARE proteins 24 h after exposure. [file 1471-2202-13-127-S3.png]
